# Supplementary material for: Stereospecific Si-C coupling and remote control of axial chirality by enantioselective palladium-catalyzed hydrosilylation of maleimides
Source: Nat Commun. 2020 Jun 9;11:2904. doi: 10.1038/s41467-020-16716-5 (PMC7283218; doi:10.1038/s41467-020-16716-5)
Supplement: Supplementary file 1 — Supplementary Information [file 41467_2020_16716_MOESM1_ESM.pdf]

Supplementary Information

Stereospecific Si-C Coupling and Remote Control of Axial Chirality by Enantioselective Palladium-Catalyzed Hydrosilylation of Maleimides

Xing-Wei Gu *et al*

Contents

Supplementary Methods **3**

Supplementary Tables **61**

Supplementary Discussion **67**

Supplementary Figures **71**

Supplementary References **238**

Supplementary Methods

General Information

Unless specifically stated, all reagents were commercially obtained and where appropriate, purified prior to use. Dichloromethane (DCM), toluene, were freshly distilled from CaH_2, Ether (Et_2O), tetrahydrofuran (THF) and 1, 4-dioxane were dried and distilled from metal sodium and benzophenone. Alcohol solvents were dried and distilled from metal magnesium. Other commercially available reagents and solvents were used directly without purification. Reactions were monitored by thin layer chromatography (TLC) using silica gel plates. Flash column chromatography was performed over silica (200 - 300 mesh). ^1H, ^{13}C NMR spectra were recorded on a Bruker 400 MHz or 500 MHz spectrometer in $CDCl_3$. Multiplicities were given as: s (singlet); d (doublet); dd (doublets of doublet); t (triplet); q (quartet) or m (multiplets). High resolution mass spectra (HRMS) of the products were obtained on a Bruker Daltonics micro TOF-spectrometer. HPLC was carried out with a Agilent 1260 infinity, Waters AcQuity HPLC or Waters AcQuity UPLC using a chiralcel OD column, a chiralcel Phenomenex column， a chiralcel INA column, a chiralcel INB column, a chiralcel MD column, a chiralcel IA column, and a chiralcel IB column.

General procedure for the synthesis of N-aryl maleimide substrates

N-aryl maleimide derivatives were prepared by reported methods.[1-2] Characterization of unreported N-aryl maleimide substrates is listed below.

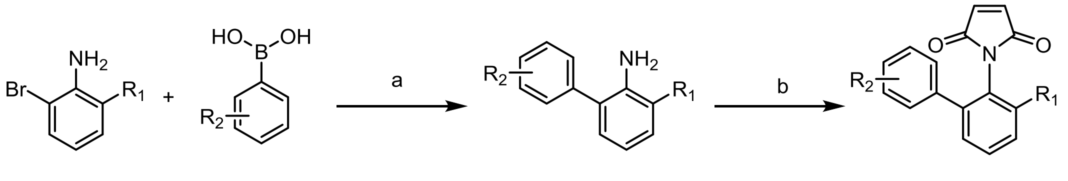

Reaction conditions:

a): To an oven-dried three necked flask, 2-bromoaniline(5 mmol), phenylboronic acid (6 mmol), K_2CO_3(22.5 mmol), DME (13 mL) and H_2O (17 ml) were added under a gentle stream of nitrogen, and the mixture was stirred for 30 min at room

temperature under N_2 atmosphere. To the stirred mixture, $PdCl_2(PPh_3)_2$ (70 mg, 0.1 mmol) was added at room temperature, and the mixture was stirred for overnight at 80 ℃, under N_2. The reaction mixture was then cooled to room temperature and diluted with EtOAc. The organic layer was washed with water and dried over Na_2SO_4. After removing the volatiles in vacuo, the residue was subjected to column chromatography on silica gel (PE/EA = 50/1) to afford 2-Aminodiphenyl;

b): Maleic anhydride (10 mmol) and primary amine (5 mmol) were stirred in acetic acid (1.5 ml per mmol of amine) until maleic anhydride dissolved completely. The reaction mixture was refluxed for 6-8 h at 115 ℃ (oil bath temperature). After completion of the reaction, the reaction mixture was then allowed to cool down to room temperature and the whole reaction mixture was transferred to a 500 mL beaker. Saturated sodium bicarbonate aqueous solution was added to the beaker containing reaction mixture until effervescence stop. The aqueous mixture was extracted with ethyl acetate (3x20 mL). The organic layer was further washed with 1(N) HCl (2x50 mL)and brine solution (30 mL) respectively. The excess solvent was removed under reduced pressure and the residue was purified by flash column chromatography using PE/EA = 3:1 to get highly pure maleimide.

Characterized data of N-aryl maleimide derivative

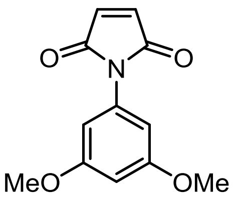

1r

1-(3,5-dimethoxyphenyl)-1H-pyrrole-2,5-dione (1r)

Yellow solid, mp 111 - 114 ^{o}C. ^{1}H NMR (400 MHz, $CDCl_3$) δ 6.74 (s, 2H), 6.47 - 6.33 (m, 3H), 3.71 (s, 6H). ^{13}C NMR (100 MHz, $CDCl_3$) δ 169.5, 161.0, 134.2, 132.7, 104.7, 100.3, 55.6. HRMS (ESI) m/z: $[M+Na]^+$ calculated for $C_{12}H_{11}NNaO_4$:

256.0580, found: 256.0587.

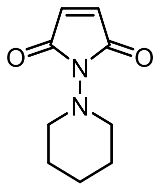

1cc

1-(piperidin-1-yl)-1H-pyrrole-2,5-dione (1cc)

Yellow solid, mp 92 - 94 °C. ^{1}H NMR (400 MHz, $CDCl_3$) δ 6.54 (s, 2H), 3.23 - 3.06 (m, 4H), 1.73 - 1.58 (m, 4H), 1.48 - 1.31 (m, 2H). ^{13}C NMR (100 MHz, $CDCl_3$) δ 169.3, 132.5, 53.4, 26.1, 23.0. HRMS (ESI) m/z: $[M+Na]^+$ calculated for $C_9H_{12}N_2NaO_2$: 203.0791, found: 203.0789.

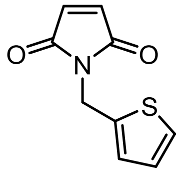

1dd

1-(thiophen-2-ylmethyl)-1H-pyrrole-2,5-dione (1dd)

White solid, mp 79 - 82 °C. ^{1}H NMR (400 MHz, $CDCl_3$) δ 7.11 (dd, J = 5.2, 0.8 Hz, 1H), 6.96 (d, J = 3.2 Hz, 1H), 6.82 (dd, J = 4.8, 3.2 Hz, 1H), 6.61 (s, 2H), 4.74 (s, 2H). ^{13}C NMR (100 MHz, $CDCl_3$) δ 169.9, 138.0, 134.2, 127.5, 126.9, 125.9, 35.5. MS (EI) m/z: 193, 164, 136, 112, 110, 97, 96, 84, 82.

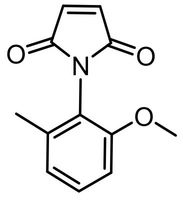

1hh

1-(2-methoxy-6-methylphenyl)-1H-pyrrole-2,5-dione (1hh)

White solid, mp 145 - 147 °C. ^{1}H NMR (400 MHz, $CDCl_3$) δ 7.20 (t, J = 8.0 Hz, 1H), 6.82 (d, J = 7.7 Hz, 1H), 6.78 - 6.71 (m, 3H), 3.66 (s, 3H), 2.07 (s, 3H). ^{13}C NMR (100 MHz, $CDCl_3$) δ 169.9, 155.8, 138.9, 134.6, 130.3, 122.6, 118.8, 109.3, 55.9, 17.7. HRMS (ESI) m/z: $[M+H]^+$ calculated for $C_{12}H_{12}NO_3$: 218.0812, found: 218.0821.

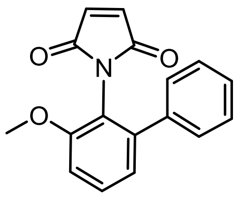

4a

1-(3-methoxy-[1,1'-biphenyl]-2-yl)-1H-pyrrole-2,5-dione (4a)

Yellow solid, mp 147 - 150 °C. ^{1}H NMR (400 MHz, $CDCl_3$) δ 7.37 (t, J = 8.1 Hz, 1H), 7.28 - 7.14 (m, 5H), 7.01 - 6.87 (m, 2H), 6.57 (s, 2H), 3.72 (s, 3H). ^{13}C NMR (100 MHz, $CDCl_3$) δ 170.1, 156.0, 144.00, 138.5, 134.4, 130.6, 128.3, 128.3, 127.7, 122.5, 117.7, 110.8, 56.1. HRMS (ESI) m/z: $[M+Na]^+$ calculated for $C_{17}H_{13}NNaO_3$: 302.0788, found: 302.0777.

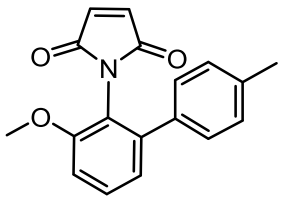

4b

1-(3-methoxy-4'-methyl-[1,1'-biphenyl]-2-yl)-1H-pyrrole-2,5-dione (4b)

White solid, mp 135 - 136 °C. ^{1}H NMR (400 MHz, $CDCl_3$) δ 7.35 (t, J = 8.1 Hz, 1H), 7.09 - 7.00 (m, 4H), 6.96 - 6.86 (m, 2H), 6.57 (s, 2H), 3.71 (s, 3H), 2.24 (s, 3H). ^{13}C NMR (100 MHz, $CDCl_3$) δ 170.1, 156.0, 143.9, 137.4, 135.6, 134.4, 130.6, 129.1, 128.1, 122.5, 117.6, 110.6, 56.1, 21.3. HRMS (ESI) m/z: $[M+Na]^+$ calculated for $C_{18}H_{15}NNaO_3$: 316.0944, found: 316.0934.

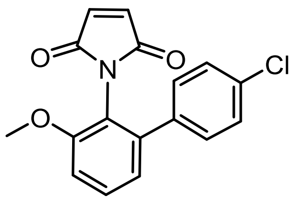

4c

1-(4'-chloro-3-methoxy-[1,1'-biphenyl]-2-yl)-1H-pyrrole-2,5-dione (4c)

Yellow solid, mp 103 - 106 °C. ^{1}H NMR (400 MHz, $CDCl_3$) δ 7.36 (t, J = 8.1 Hz, 1H), 7.24 - 7.18 (m, 2H), 7.13 - 7.07 (m, 2H), 6.96 - 6.88 (m, 2H), 6.60 (s, 2H), 3.72 (s, 3H). ^{13}C NMR (100 MHz, $CDCl_3$) δ 170.0, 156.1, 142.8, 137.0, 134.5, 133.8, 130.8,

129.7, 128.6, 122.2, 117.7, 111.1, 56.1. HRMS (ESI) m/z: $[M+H]^+$ calculated for $C_{17}H_{13}ClNO_3$: 314.0578, found: 314.0586.

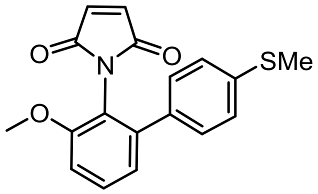

4d

1-(3-methoxy-4'-(methylthio)-[1,1'-biphenyl]-2-yl)-1H-pyrrole-2,5-dione (4d)

White solid, mp 118 - 121 ^oC. ^1H NMR (400 MHz, $CDCl_3$) δ 7.35 (t, J = 8.1 Hz, 1H), 7.09 (d, J = 1.1 Hz, 4H), 6.98 - 6.83 (m, 2H), 6.59 (s, 2H), 3.71 (s, 3H), 2.37 (s, 3H). ^{13}C NMR (100 MHz, $CDCl_3$) δ 170.1, 156.0, 143.3, 138.1, 135.1, 134.4, 130.6, 128.7, 126.0, 122.3, 117.6, 110.8, 56.1, 15.5. HRMS (ESI) m/z: $[M+Na]^+$ calculated for $C_{18}H_{15}NNaO_3S$: 348.0665, found: 348.0678.

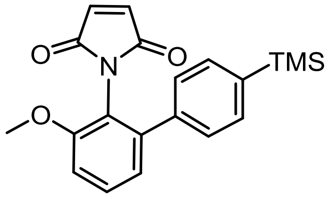

4e

1-(3-methoxy-4'-(trimethylsilyl)-[1,1'-biphenyl]-2-yl)-1H-pyrrole-2,5-dione (4e)

White solid, mp 166 - 169 ^oC. ^1H NMR (400 MHz, $CDCl_3$) δ 7.36 (dd, J = 16.8, 8.1 Hz, 3H), 7.15 (d, J = 8.0 Hz, 2H), 6.97 - 6.87 (m, 2H), 6.58 (s, 2H), 3.71 (s, 3H), 0.17 (s, 9H). ^{13}C NMR (100 MHz, $CDCl_3$) δ 170.2, 156.0, 144.0, 139.7, 138.8, 134.4, 133.3, 130.7, 127.5, 122.5, 117.6, 110.8, 56.1, -1.0. HRMS (ESI) m/z: $[M+Na]^+$ calculated for $C_{20}H_{21}NNaO_3Si$: 374.1183, found: 374.1201.

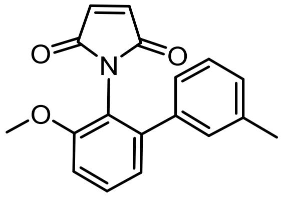

4f

1-(3-methoxy-3'-methyl-[1,1'-biphenyl]-2-yl)-1H-pyrrole-2,5-dione (4f)

Yellow solid, mp 113 - 115 °C. ^{1}H NMR (400 MHz, $CDCl_3$) δ 7.35 (t, J = 8.1 Hz, 1H), 7.11 (t, J = 7.5 Hz, 1H), 7.03 - 6.87 (m, 5H), 6.57 (s, 2H), 3.71 (s, 3H), 2.23 (s, 3H). ^{13}C NMR (100 MHz, $CDCl_3$) δ 170.1, 156.0, 144.0, 138.5, 137.9, 134.4, 130.6, 129.1, 128.4, 128.1, 125.3, 122.5, 117.6, 110.7, 56.1, 21.5. HRMS (ESI) m/z: $[M+Na]^+$ calculated for $C_{18}H_{15}NNaO_3$: 316.0944, found: 316.0955.

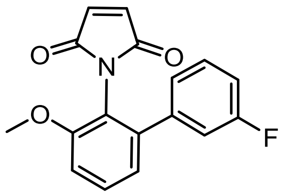

4g

1-(3'-fluoro-3-methoxy-[1,1'-biphenyl]-2-yl)-1H-pyrrole-2,5-dione (4g)

Yellow solid, mp 139 - 142 °C. ^{1}H NMR (400 MHz, $CDCl_3$) δ 7.36 (t, J = 8.1 Hz, 1H), 7.25 - 7.12 (m, 1H), 7.01 - 6.81 (m, 5H), 6.60 (s, 2H), 3.71 (s, 3H). ^{13}C NMR (100 MHz, $CDCl_3$) δ 170.0, 162.5 (d, J = 246.5 Hz), 156.1, 142.6 (d, J = 1.9 Hz), 140.6 (d, J = 8.0 Hz), 134.5, 130.8, 129.9 (d, J = 8.5 Hz), 124.1 (d, J = 2.9 Hz), 122.2, 117.6, 115.4 (d, J = 22.0 Hz), 114.7 (d, J = 21.1 Hz), 111.3, 56.1. ^{19}F NMR (471 MHz, $CDCl_3$) δ -112.96. HRMS (ESI) m/z: $[M+Na]^+$ calculated for $C_{17}H_{12}FNNaO_3$: 320.0693, found: 320.0704.

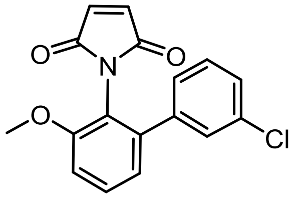

4h

1-(3'-chloro-3-methoxy-[1,1'-biphenyl]-2-yl)-1H-pyrrole-2,5-dione (4h)

Yellow solid, mp 120 - 122 °C. ^{1}H NMR (400 MHz, $CDCl_3$) δ 7.43 - 7.31 (m, 1H), 7.24 - 7.12 (m, 3H), 7.10 - 7.00 (m, 1H), 6.97 - 6.87 (m, 2H), 6.60 (s, 2H), 3.71 (s, 3H). ^{13}C NMR (100 MHz, $CDCl_3$) δ 170.0, 156.0, 142.5, 140.2, 134.5, 134.1, 130.8, 129.6, 128.5, 127.9, 126.5, 122.2, 117.6, 111.3, 56.1. HRMS (ESI) m/z: $[M+Na]^+$ calculated for $C_{17}H_{12}ClNNaO_3$: 336.0398, found: 336.0408.

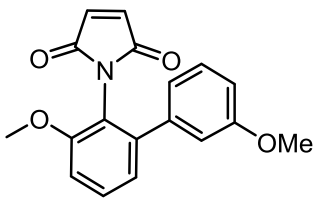

4i

1-(3,3'-dimethoxy-[1,1'-biphenyl]-2-yl)-1H-pyrrole-2,5-dione (4i)

Yellow solid, mp 90 - 92 °C. ^{1}H NMR (400 MHz, $CDCl_3$) δ 7.36 (t, J = 8.1 Hz, 1H), 7.14 (dd, J = 15.7, 7.8 Hz, 1H), 7.01 - 6.85 (m, 2H), 6.82 - 6.67 (m, 3H), 6.58 (s, 2H), 3.71 (s, 3H), 3.66 (s, 3H). ^{13}C NMR (100 MHz, $CDCl_3$) δ 170.1, 159.3, 156.0, 143.8, 139.8, 134.4, 130.6, 129.3, 122.4, 120.7, 117.6, 113.7, 113.5, 110.9, 56.1, 55.3. HRMS (ESI) m/z: $[M+H]^+$ calculated for $C_{18}H_{16}NO_4$: 310.1074, found: 310.1085.

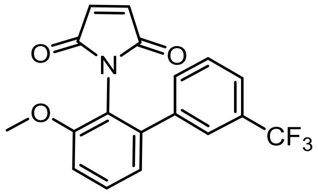

4j

1-(3-methoxy-3'-(trifluoromethyl)-[1,1'-biphenyl]-2-yl)-1H-pyrrole-2,5-dione (4j)

Yellow solid, mp 108 - 111 °C. ^{1}H NMR (400 MHz, $CDCl_3$) δ 7.53 -7.32 (m, 5H), 6.95 (dd, J = 8.1, 1.6 Hz, 2H), 6.59 (s, 2H), 3.73 (s, 3H). ^{13}C NMR (100 MHz, $CDCl_3$) δ 170.0, 156.2, 142.4, 139.3, 134.5, 131.8, 130.9, 128.9, 125.2 (d, J = 3.9 Hz), 124.5 (d, J = 3.9 Hz), 122.7, 122.2, 117.8, 111.5, 56.2. ^{19}F NMR (471 MHz, $CDCl_3$) δ -62.64. HRMS (ESI) m/z: $[M+Na]^+$ calculated for $C_{18}H_{12}F_3NNaO_3$: 370.0661, found: 370.0674.

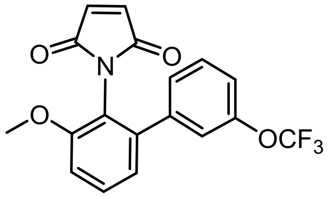

4k

1-(3-methoxy-3'-(trifluoromethoxy)-[1,1'-biphenyl]-2-yl)-1H-pyrrole-2,5-dione (4k)

White solid, mp 68 - 69 °C. ^{1}H NMR (400 MHz, CDCl$_3$) δ 7.39 (t, J = 8.1 Hz, 1H), 7.28 (t, J = 7.9 Hz, 1H), 7.14 (d, J = 7.7 Hz, 1H), 7.10 - 7.01 (m, 2H), 6.95 (d, J = 7.9 Hz, 2H), 6.60 (s, 2H), 3.73 (s, 3H). ^{13}C NMR (100 MHz, CDCl$_3$) δ 170.0, 156.2, 149.00, 142.4, 140.4, 134.5, 130.9, 129.9, 127.1, 122.2, 121.0, 120.3, 117.8, 111.5, 56.2. ^{19}F NMR (471 MHz, CDCl$_3$) δ -57.80. HRMS (ESI) m/z: [M+Na]$^+$ calculated for $C_{18}H_{12}F_3NNaO_4$: 386.0611, found: 386.0627.

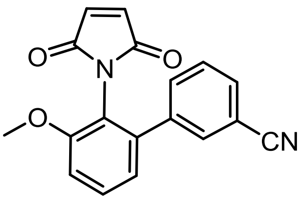

4l

2'-(2,5-dioxo-2,5-dihydro-1H-pyrrol-1-yl)-3'-methoxy-[1,1'-biphenyl]-3-carbonitrile (4l)

White solid, mp 167 - 169 °C. ^{1}H NMR (400 MHz, CDCl$_3$) δ 7.56 - 7.46 (m, 2H), 7.44 - 7.32 (m, 3H), 6.98 (d, J = 8.4 Hz, 1H), 6.91 (d, J = 7.7 Hz, 1H), 6.63 (s, 2H), 3.74 (s, 3H). ^{13}C NMR (100 MHz, CDCl$_3$) δ 169.8, 156.1, 141.6, 139.8, 134.6, 132.8, 131.8, 131.4, 131.0, 129.2, 122.0, 118.5, 117.8, 112.6, 111.8, 56.2. HRMS (ESI) m/z: [M+Na]$^+$ calculated for $C_{18}H_{12}N_2NaO_3$: 327.0740, found: 327.0750.

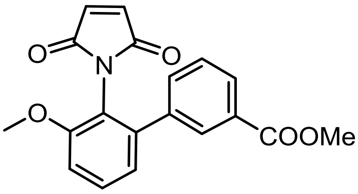

4m

Methyl 2'-(2,5-dioxo-2,5-dihydro-1H-pyrrol-1-yl)-3'-methoxy-[1,1'-biphenyl]-3-carboxylate (4m)

White solid, mp 116 - 120 °C. ^{1}H NMR (400 MHz, CDCl$_3$) δ 7.89 (d, J = 7.4 Hz, 2H), 7.44 - 7.25 (m, 3H), 7.00 - 6.90 (m, 2H), 6.58 (s, 2H), 3.80 (s, 3H), 3.72 (s, 3H). ^{13}C NMR (100 MHz, CDCl$_3$) δ 169.9, 166.8, 156.1, 142.8, 138.8, 134.4, 132.7, 130.7, 130.4, 129.6, 128.9, 128.4, 122.3, 117.7, 111.3, 56.1, 52.2 . HRMS (ESI) m/z: [M+Na]$^+$ calculated for $C_{19}H_{15}NNaO_5$: 360.0842, found: 360.0860.

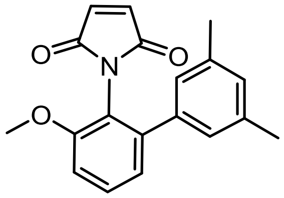

4n

1-(3-methoxy-3',5'-dimethyl-[1,1'-biphenyl]-2-yl)-1H-pyrrole-2,5-dione (4n)

Yellow solid, mp 117 - 120 °C. ^{1}H NMR (400 MHz, $CDCl_3$) δ 7.32 (t, J = 8.1 Hz, 1H), 6.97 - 6.81 (m, 3H), 6.78 (s, 2H), 6.57 (s, 2H), 3.69 (s, 3H), 2.17 (s, 6H). ^{13}C NMR (100 MHz, $CDCl_3$) δ 170.0, 156.0, 144.1, 138.4, 137.7, 134.4, 130.5, 129.3, 126.0, 122.5, 117.6, 110.6, 56.1, 21.3. HRMS (ESI) m/z: $[M+H]^+$ calculated for $C_{19}H_{18}NO_3$: 308.1281, found: 308.1291.

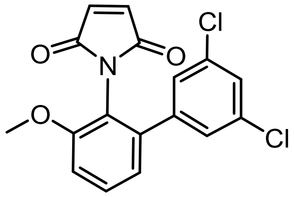

4o

1-(3',5'-dichloro-3-methoxy-[1,1'-biphenyl]-2-yl)-1H-pyrrole-2,5-dione (4o)

White solid, mp 126 - 130 °C. ^{1}H NMR (400 MHz, $CDCl_3$) δ 7.39 (t, J = 8.1 Hz, 1H), 7.31 - 7.05 (m, 3H), 6.93 (dd, J = 21.8, 8.0 Hz, 2H), 6.66 (s, 2H), 3.74 (s, 3H). ^{13}C NMR (100 MHz, $CDCl_3$) δ 169.9, 156.2, 141.4, 141.3, 134.9, 134.6, 130.9, 128.0, 127.0, 122.1, 117.7, 111.8, 56.3. HRMS (ESI) m/z: $[M+Na]^+$ calculated for $C_{17}H_{11}Cl_2NNaO_3$: 370.0008, found: 370.0020.

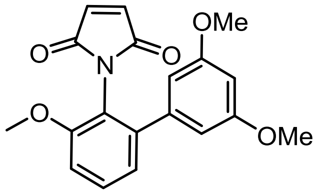

4p

1-(3,3',5'-trimethoxy-[1,1'-biphenyl]-2-yl)-1H-pyrrole-2,5-dione (4p)

White solid, mp 118 - 120 °C. ^{1}H NMR (400 MHz, $CDCl_3$) δ 7.35 (t, J = 8.1 Hz, 1H), 6.93 (dd, J = 19.3, 8.0 Hz, 2H), 6.60 (s, 2H), 6.32 (dd, J = 9.1, 2.1 Hz, 3H), 3.71 (s, 3H), 3.64 (s, 6H). ^{13}C NMR (100 MHz, $CDCl_3$) δ 170.1, 160.5, 156.1, 143.9, 140.4,

134.5, 130.6, 122.3, 117.6, 110.9, 106.3, 100.3, 56.1, 55.4. HRMS (ESI) m/z: $[M+Na]^+$ calculated for $C_{19}H_{17}NNaO_5$: 362.0999, found: 362.0988.

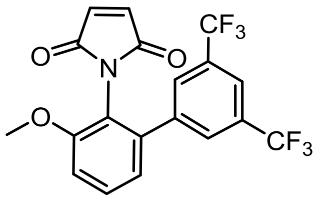

4q

1-(3-methoxy-3',5'-bis(trifluoromethyl)-[1,1'-biphenyl]-2-yl)-1H-pyrrole-2,5-dione (4q)

White solid, mp 121 - 124 °C. ^{1}H NMR (400 MHz, $CDCl_3$) δ 7.75 (s, 1H), 7.66 (s, 2H), 7.44 (t, J = 8.1 Hz, 1H), 7.00 (dd, J = 14.2, 8.1 Hz, 2H), 6.64 (s, 2H), 3.76 (s, 3H). ^{13}C NMR (100 MHz, $CDCl_3$) δ 169.8, 156.4, 140.9, 140.6, 134.6, 131.9, 131.6, 131.2, 128.7, 124.6, 122.0, 121.6 (d, J = 3.7 Hz), 118.0, 112.3, 56.3. ^{19}F NMR (471 MHz, $CDCl_3$) δ -62.86. HRMS (ESI) m/z: $[M+Na]^+$ calculated for $C_{19}H_{11}F_6NNaO_3$: 438.0535, found: 438.0537.

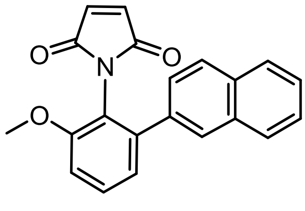

4r

1-(2-methoxy-6-(naphthalen-2-yl)phenyl)-1H-pyrrole-2,5-dione (4r)

Yellow solid, mp 120 - 122 °C. ^{1}H NMR (400 MHz, $CDCl_3$) δ 7.77 - 7.63 (m, 4H), 7.44 - 7.34 (m, 3H), 7.28 (dd, J = 8.4, 1.2 Hz, 1H), 7.03 (d, J = 7.7 Hz, 1H), 6.93 (d, J = 8.3 Hz, 1H), 6.51 (s, 2H), 3.73 (s, 3H). ^{13}C NMR (100 MHz, $CDCl_3$) δ 170.1, 156.1, 144.0, 136.1, 134.4, 133.3, 132.7, 130.7, 128.3, 127.9, 127.7, 127.4, 126.4, 126.3, 126.3, 122.8, 117.9, 111.0, 56.2. HRMS (ESI) m/z: $[M+Na]^+$ calculated for $C_{21}H_{15}NNaO_3$: 352.0944, found: 352.0962.

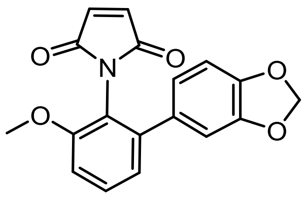

4s

1-(2-(benzo[d][1,3]dioxol-5-yl)-6-methoxyphenyl)-1H-pyrrole-2,5-dione (4s)

White solid, mp 174 - 176 °C. ^{1}H NMR (400 MHz, $CDCl_3$) δ 7.34 (t, J = 8.1 Hz, 1H), 6.90 (t, J = 8.0 Hz, 2H), 6.76 - 6.49 (m, 5H), 5.85 (s, 2H), 3.71 (s, 3H). ^{13}C NMR (100 MHz, $CDCl_3$) δ 170.1, 156.0, 147.5, 147.2, 143.5, 134.5, 132.3, 130.6, 122.5, 121.8, 117.7, 110.7, 109.0, 108.2, 101.1, 56.1. HRMS (ESI) m/z: [M+Na]$^+$ calculated for $C_{18}H_{13}NNaO_5$: 346.0686, found: 346.0700.

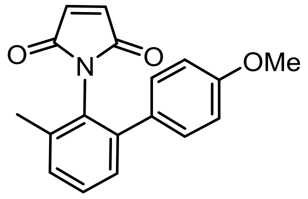

4t

1-(4'-methoxy-3-methyl-[1,1'-biphenyl]-2-yl)-1H-pyrrole-2,5-dione (4t)

White solid, mp 137 - 140 °C. ^{1}H NMR (400 MHz, $CDCl_3$) δ 7.29 (t, J = 7.6 Hz, 1H), 7.18 (dd, J = 22.3, 7.5 Hz, 2H), 7.03 (d, J = 8.6 Hz, 2H), 6.75 (d, J = 8.6 Hz, 2H), 6.55 (s, 2H), 3.70 (s, 3H), 2.10 (s, 3H). ^{13}C NMR (100 MHz, $CDCl_3$) δ 169.8, 159.0, 142.2, 137.5, 134.2, 131.5, 129.9, 129.6, 129.4, 128.6, 128.2, 113.7, 55.3, 18.2. HRMS (ESI) m/z: [M+Na]$^+$ calculated for $C_{18}H_{15}NNaO_3$: 316.0944, found: 316.0953.

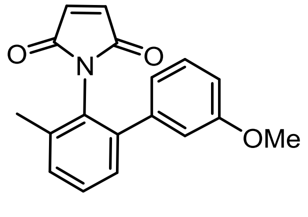

4u

1-(3'-methoxy-3-methyl-[1,1'-biphenyl]-2-yl)-1H-pyrrole-2,5-dione (4u)

White solid, mp 77 - 80 °C. ^{1}H NMR (400 MHz, $CDCl_3$) δ 7.48 - 7.20 (m, 4H), 6.90 - 6.75 (m, 3H), 6.67 (s, 2H), 3.77 (s, 3H), 2.23 (s, 3H). ^{13}C NMR (100 MHz, $CDCl_3$) δ 169.8, 159.3, 142.4, 140.5, 137.7, 134.3, 130.2, 129.6, 129.3, 128.4, 128.0, 120.7,

113.7, 113.5, 55.3, 18.1. HRMS (ESI) m/z: $[M+Na]^+$ calculated for $C_{18}H_{15}NNaO_3$: 316.0944, found: 316.0955.

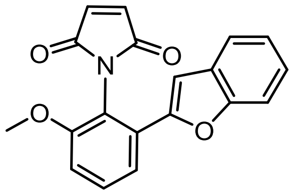

4z

1-(2-(benzofuran-2-yl)-6-methoxyphenyl)-1H-pyrrole-2,5-dione (4z)

Yellow solid, mp 146 - 148 ^oC. ^1H NMR (400 MHz, $CDCl_3$) δ 7.61 -7.48 (m, 3H), 7.42 (d, J = 8.1 Hz, 1H), 7.34 - 7.19 (m, 2H), 7.04 (d, J = 8.1 Hz, 1H), 6.94 (s, 2H), 6.87 (s, 1H), 3.85 (s, 3H). ^{13}C NMR (100 MHz, $CDCl_3$) δ 170.0, 156.7, 154.8, 152.7, 135.0, 131.4, 130.9, 128.6, 124.9, 123.1, 121.3, 120.4, 116.6, 112.0, 111.2, 105.0, 56.2. HRMS (ESI) m/z: $[M+Na]^+$ calculated for $C_{19}H_{13}NNaO_4$: 342.0737, found: 342.0752.

Preparation of chiral ligands

The chiral ligands **L14 - L18** were purchased and used without purification. **L1-L13**, **L19** were prepared by reported methods. The spectra of **L1**, **L3**, **L9**, **L10**, **L12**, **L13**[3-7] were in accordance with the reported data. **L2**, **L6**, **L7**, **L8**, **L11**, **L19** was prepared from known TADDOL **(*R,R*)-S1** by modified procedure.

Procedure for the preparation of L2, L6, L7, L8, L11[8]

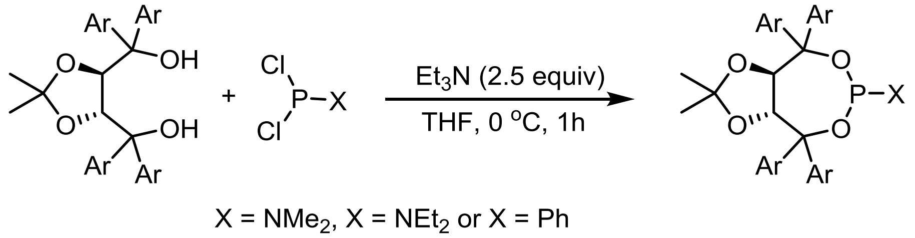

Dichloro(dimethylamino)phosphine (640 μL, 5.5 mmol) was added to a solution of TADDOL derivative (5.5 mmol) and triethylamine (1.9 mL, 13.8 mmol) in THF (38 mL) at 0 ℃. After stirring for 1 hour at 0 ℃, the reaction mixture was allowed to warm to room temperature and stirred overnight. The reaction mixture was the quenched with water, extracted with dichloromethane three times, and dried over

sodium sulfate, the volatile solvents were removed. The residue was dried on a vacuum line. Flash chromatography (90:1 hexanes:ethyl acetate) afforded (R,R)-L as a foamy solid.

Characterized data of TADDOL ligands

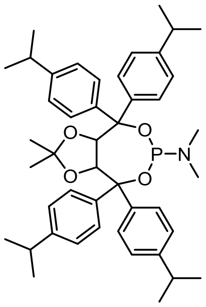

(*R,R*)-L2

White solid, mp 190 - 192 °C. ^{1}H NMR (500 MHz, CDCl$_3$) δ 7.75 (d, J = 8.1 Hz, 2H), 7.59 (d, J = 8.0 Hz, 2H), 7.42 (dd, J = 16.3, 8.1 Hz, 4H), 7.27 - 7.13 (m, 8H), 5.24 (dd, J = 8.3, 2.6 Hz, 1H), 4.83 (d, J = 8.5 Hz, 1H), 3.05 - 2.87 (m, 4H), 2.82 (d, J = 10.5 Hz, 6H), 1.40 (s, 3H), 1.30 (s, 18H), 1.24 (d, J = 6.8 Hz, 6H), 0.29 (s, 3H). ^{13}C NMR (126 MHz, CDCl$_3$) δ 147.73, 147.66, 147.53, 147.24, 144.52, 144.34, 139.70, 129.10, 128.58, 127.11, 126.21, 125.78, 125.71, 125.21, 111.51, 83.20, 81.36, 81.32, 81.26, 35.65, 35.57, 35.50, 35.42, 33.75, 27.71, 25.18, 24.09, 24.04. ^{31}P NMR (202 MHz, CDCl$_3$) δ 139.06. HRMS (ESI) m/z: [M+Na]$^+$ calculated for $C_{45}H_{58}NNaO_4P$: 730.3996, found: 730.3986.

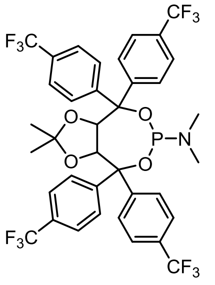

(*R,R*)-L7

White solid, mp 198 - 201 °C. ^{1}H NMR (500 MHz, CDCl$_3$) δ 7.78 (d, J = 8.3 Hz, 2H), 7.63 (d, J = 8.3 Hz, 2H), 7.56 – 7.41 (m, 12H), 5.02 (dd, J = 8.6, 3.4 Hz, 1H), 4.57 (d, J = 8.6 Hz, 1H), 2.71 (s, 3H), 2.69 (s, 3H), 1.26 (s, 3H), 0.25 (s, 3H). ^{13}C NMR (125

MHz, $CDCl_3$) : Many signals due to C-P and C-F coupling. ^{31}P NMR (202 MHz, $CDCl_3$) δ 140.13. HRMS (ESI) m/z: $[M+H]^+$ calculated for $C_{37}H_{31}F_{12}NO_4P$: 812.1794, found: 812.1784.

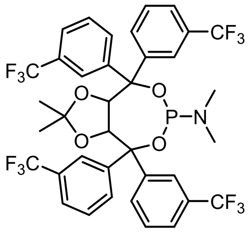

(*R*,*R*)-L8

White solid, mp 129 - 131 °C. ^1H NMR (500 MHz, $CDCl_3$) δ 8.04 (s, 1H), 7.85 - 7.77 (m, 2H), 7.72 (d, *J* = 17.5 Hz, 2H), 7.61 (s, 1H), 7.53 (d, *J* = 7.9 Hz, 1H), 7.49 - 7.29 (m, 9H), 4.97 (dd, *J* = 8.6, 3.4 Hz, 1H), 4.50 (d, *J* = 8.6 Hz, 1H), 2.72 (s, 3H), 2.70 (s, 3H), 1.30 (s, 3H), 0.21 (s, 3H). ^{13}C NMR (125 MHz, $CDCl_3$) : Many signals due to C-P and C-F coupling. ^{19}F NMR (471 MHz, $CDCl_3$) δ -62.55, -62.69, -62.82. ^{31}P NMR (202 MHz, $CDCl_3$) δ 139.88. HRMS (ESI) m/z: $[M+H]^+$ calculated for $C_{37}H_{31}F_{12}NO_4P$: 812.1794, found: 812.1784.

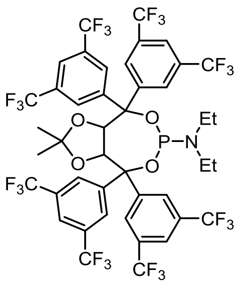

(*R*,*R*)-L11

White solid, mp 84 - 87 °C. ^1H NMR (500 MHz, $CDCl_3$) δ 8.23 (s, 2H), 7.98 (s, 2H), 7.78 (d, *J* = 13.0 Hz, 6H), 7.73 (s, 2H), 4.97 - 4.81 (m, 1H), 4.23 (dd, *J* = 8.8, 2.4 Hz, 1H), 3.43 - 3.12 (m, 4H), 1.47 (d, *J* = 1.9 Hz, 3H), 1.18 - 1.09 (m, 6H), 0.26 (d, *J* = 1.4 Hz, 3H). ^{13}C NMR (125 MHz, $CDCl_3$) : Many signals due to C-P and C-F coupling. ^{19}F NMR (471 MHz, $CDCl_3$) δ -63.20, -63.24, -63.34, -63.35, -63.38. ^{31}P NMR (202 MHz, $CDCl_3$) δ 141.13. HRMS (ESI) m/z: $[M+H]^+$ calculated for $C_{43}H_{31}F_{24}NO_4P$: 1112.1602, found: 1112.1612.

General procedure for the catalytic asymmetric hydrosilylation to the synthesis of silyl products 3 and 5

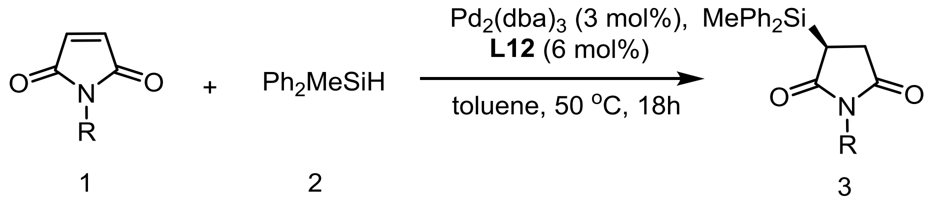

A vial was charged with N-phenyl Maleimide **1a** (52.0 mg, 0.3 mmol), $Pd_2(dba)_3$ (8.2 mg, 3.0 mol%), (*R*,*R*)-**L12** (20.1 mg, 6 mol%) and evacuated under high vacuum and backfilled with N_2. Toluene (3 mL) was next added. The mixture was stirred at 25 ^{o}C for 10 minutes, then the Ph_2MeSiH (0.6 mmol) was added to the reaction. The mixture was stirred at 50 ℃ in a preheated oil. Upon reaction completion, the mixture was filtered over a plug of silica gel (washed with 50 ml EtOAc), and the filtrate was concentrated. The crude was purified by column chromatography to give the corresponding product and was analyzed with ^{1}H NMR to determine the corresponding product ratio and recovered.

Characterized data of hydrosilylation to products

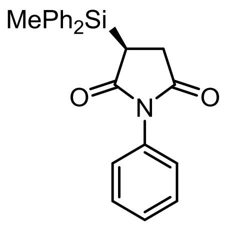

(*S*)-3a

(*S*)-3-(methyldiphenylsilyl)-1-phenylpyrrolidine-2,5-dione (3a)

Yellow oil (94.3 mg, 85% yield), purified by column chromatography (SiO_2, PE/EA= 6:1). $[\alpha]_D^{25}$= +55.5 (c=1.14, $CHCl_3$). ^{1}H NMR (400 MHz, $CDCl_3$) δ 7.55 - 7.48 (m, 1H), 7.42 - 7.37 (m, 1H), 7.36 - 7.21 (m, 2H), 6.80 - 6.74 (m, 1H), 3.06 - 2.96 (m, 1H), 2.80 - 2.68 (m, 1H), 0.72 (s, 1H). ^{13}C NMR (100 MHz, $CDCl_3$) δ 178.2, 175.9, 135.1, 134.9, 134.1, 132.9, 132.1, 132.1, 130.7, 130.5, 129.1, 128.5, 126.6, 32.0, 31.0, -4.3.

HRMS (ESI) m/z: $[M+Na]^+$ calculated for $C_{23}H_{21}NNaO_2Si$: 394.1234, found: 394.1240. The enantiomeric excess was determined by UPLC with Chiralpark INB column (hexanes:2-propanol = 90:10, 0.8 mL/min, 211 nm, 96% *ee*); major enantiomer tr = 15.916 min, minor enantiomer tr = 13.339 min.

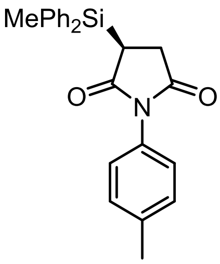

(*S*)-3b

(*S*)-3-(methyldiphenylsilyl)-1-(p-tolyl)pyrrolidine-2,5-dione (3b)

Yellow oil (91.8 mg, 79% yield), purified by column chromatography (SiO_2, PE/EA= 6:1). $[\alpha]_D^{25}$ = +79.9 (c=2.26, $CHCl_3$).^{1}H NMR (400 MHz, $CDCl_3$) δ 7.54 - 7.46 (m, 4H), 7.41 - 7.35 (m, 2H), 7.34 - 7.28 (m, 4H), 7.07 (d, *J* = 8.0 Hz, 2H), 6.63 (d, *J* = 8.4 Hz, 2H), 3.03 - 2.93 (m, 2H), 2.70 (dd, *J* = 24.0, 9.2 Hz, 1H), 2.23 (s, 3H), 0.71 (s, 3H). ^{13}C NMR (100 MHz, $CDCl_3$) δ 178.2, 176.0, 138.5, 135.0, 134.9, 132.9, 132.1, 130.6, 130.4, 129.7, 129.4, 128.4, 126.4, 31.9, 30.9, 21.2, -4.4. HRMS (ESI) m/z: $[M+Na]^+$ calculated for $C_{24}H_{23}NNaO_2Si$: 408.1390, found: 408.1399. The enantiomeric excess was determined by HPLC with Chiralpark IB column (hexanes:2-propanol = 90:10, 0.8 mL/min, 211 nm, 94% *ee*); major enantiomer tr = 21.861 min, minor enantiomer tr = 19.030 min.

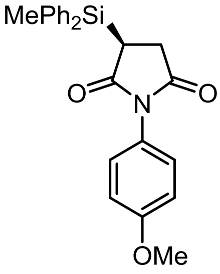

(*S*)-3c

(*S*)-1-(4-methoxyphenyl)-3-(methyldiphenylsilyl)pyrrolidine-2,5-dione (3c)

White solid (82.9 mg, 69% yield), mp 74 - 76 °C, purified by column chromatography (SiO_2, PE/EA= 4:1). $[\alpha]_D^{25}$ = +26.4 (c=0.75, $CHCl_3$).^{1}H NMR (400 MHz, $CDCl_3$) δ

7.54 - 7.46 (m, 4H), 7.42 - 7.35 (m, 2H), 7.35 - 7.28 (m, 4H), 6.78 (d, *J* = 9.2 Hz, 2H), 6.66 (d, *J* = 8.8 Hz, 2H), 3.68 (s, 3H), 3.03 - 2.92 (m, 2H), 2.76 - 2.66 (m, 1H), 0.71 (s, 3H). ^{13}C NMR (100 MHz, $CDCl_3$) δ 178.4, 176.1, 159.4, 135.1, 134.9, 132.9, 132.1, 130.6, 130.5, 128.5, 127.8, 124.7, 114.4, 55.5, 31.9, 30.9, -4.3. HRMS (ESI) m/z: $[M+Na]^+$ calculated for $C_{24}H_{23}NNaO_3Si$: 424.1339, found: 424.1338. The enantiomeric excess was determined by UPLC with Chiralpark INB column (hexanes:2-propanol = 80:20, 0.8 mL/min, 211 nm, 96% *ee*); major enantiomer tr = 13.848 min, minor enantiomer tr = 11.042 min.

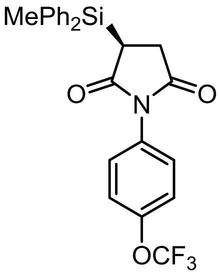

(*S*)-3d

(*S*)-3-(methyldiphenylsilyl)-1-(4-(trifluoromethoxy)phenyl)pyrrolidine-2,5-dione (3d)

White solid (75.5 mg, 55% yield), mp 95 - 98 ^{o}C, purified by column chromatography (SiO_2, PE/EA= 6:1). $[\alpha]_D^{25}$ = +54.6 (c=1.61, $CHCl_3$). ^{1}H NMR (400 MHz, $CDCl_3$) δ 7.55 - 7.46 (m, 1H), 7.42 - 7.28 (m, 2H), 7.11 (d, *J* = 8.6 Hz, 1H), 6.78 (d, *J* = 8.8 Hz, 1H), 3.08 - 2.95 (m, 1H), 2.81 - 2.68 (m, 1H), 0.72 (s, 1H). ^{13}C NMR (100 MHz, $CDCl_3$) δ 177.9, 175.6, 148.7 (d, *J* = 1.8 Hz), 135.1, 134.9, 132.7, 131.8, 130.7, 130.6, 130.5, 128.6, 128.1, 121.5, 31.9, 31.1, -4.3. ^{19}F NMR (471 MHz, $CDCl_3$) δ -57.86. HRMS (ESI) m/z: $[M+Na]^+$ calculated for $C_{24}H_{20}F_3NNaO_3Si$: 478.1057, found: 478.1056. The enantiomeric excess was determined by UPLC with Chiralpark INA column (hexanes:2-propanol = 90:10, 0.8 mL/min, 211 nm, 96% *ee*); major enantiomer tr = 7.658 min, minor enantiomer tr = 6.145 min.

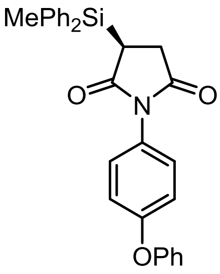

(*S*)-3e

(*S*)-3-(methyldiphenylsilyl)-1-(4-phenoxyphenyl)pyrrolidine-2,5-dione (3e)

White solid (59.3 mg, 43% yield), mp 91 - 93 ^{o}C, purified by column chromatography (SiO_2, PE/EA= 6:1). $[\alpha]_D^{25}$ = +82.5 (c=1.20, $CHCl_3$).^{1}H NMR (400 MHz, $CDCl_3$) δ 7.56 - 7.46 (m, 4H), 7.42 - 7.22 (m, 8H), 7.05 (t, *J* = 7.4 Hz, 1H), 6.94 (d, *J* = 7.6 Hz, 2H), 6.90 - 6.85 (m, 2H), 6.74 - 6.65 (m, 2H), 3.10 - 2.89 (m, 2H), 2.84 - 2.65 (m, 1H), 0.72 (s, 3H). ^{13}C NMR (100 MHz, $CDCl_3$) δ 178.3, 176.0, 157.5, 156.5, 135.1, 134.9, 132.8, 132.0, 130.7, 130.5, 130.0, 128.5, 128.1, 126.8, 124.0, 119.7, 118.8, 31.9, 31.0, -4.3. HRMS (ESI) m/z: $[M+Na]^+$ calculated for $C_{29}H_{25}NNaO_3Si$: 486.1496, found: 486.1485. The enantiomeric excess was determined by UPLC with Chiralpark INB column (hexanes:2-propanol = 90:10, 0.8 mL/min, 240 nm, 97% *ee*); major enantiomer tr = 21.009 min, minor enantiomer tr = 14.320 min.

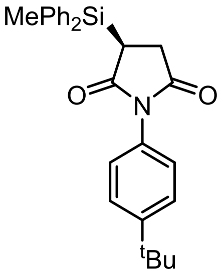

(*S*)-3f

(*S*)-1-(4-(tert-butyl)phenyl)-3-(methyldiphenylsilyl)pyrrolidine-2,5-dione (3f)

Yellow solid (88 mg, 69% yield), mp 157 - 160 ^{o}C, purified by column chromatography (SiO_2, PE/EA= 6:1). $[\alpha]_D^{25}$ = +75.6 (c=2.45, $CHCl_3$).^{1}H NMR (400 MHz, $CDCl_3$) δ 7.54 - 7.47 (m, 4H), 7.41 - 7.35 (m, 2H), 7.34 - 7.27 (m, 6H), 6.69 (d, *J* = 8.8 Hz, 2H), 2.99 (dd, *J* = 24.4, 9.6 Hz, 2H), 2.71 (dd, *J* = 24.4, 9.2 Hz, 1H), 1.20 (s, 9H), 0.71 (s, 3H). ^{13}C NMR (101 MHz, $CDCl_3$) δ 178.4, 176.1, 151.4, 135.1, 134.9, 132.9, 132.1, 130.6, 130.4, 129.3, 128.5, 126.1, 126.0, 34.7, 31.9, 31.3, 30.9, -4.4.

HRMS (ESI) m/z: $[M+Na]^+$ calculated for $C_{27}H_{29}NNaO_2Si$: 450.1860, found: 450.1875. The enantiomeric excess was determined by HPLC with Chiralpark IB column (hexanes:2-propanol = 90:10, 0.8 mL/min, 210 nm, 97% *ee*); major enantiomer tr = 17.435 min, minor enantiomer tr = 12.103 min.

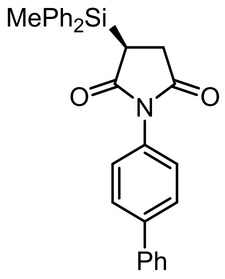

(*S*)-3g

(*S*)-1-([1,1'-biphenyl]-4-yl)-3-(methyldiphenylsilyl)pyrrolidine-2,5-dione (3g)

Yellow solid (56.9 mg, 42% yield), mp 103 - 105 °C, purified by column chromatography (SiO_2, PE/EA= 6:1). $[\alpha]_D^{25}$ = +35.2 (c=0.31, $CHCl_3$).^{1}H NMR (400 MHz, $CDCl_3$) δ 7.56 - 7.43 (m, 8H), 7.42 - 7.36 (m, 2H), 7.36 - 7.29 (m, 6H), 7.28 - 7.23 (m, 1H), 6.83 (d, *J* = 8.5 Hz, 2H), 3.07 - 2.96 (m, 2H), 2.79 - 2.69 (m, 1H), 0.73 (s, 3H). ^{13}C NMR (100 MHz, $CDCl_3$) δ 178.2, 175.9, 141.5, 140.4, 135.1, 134.9, 132.8, 132.0, 131.2, 130.7, 130.5, 128.9, 128.5, 127.9, 127.7, 127.3, 126.8, 32.0, 31.0, -4.3. HRMS (ESI) m/z: $[M+Na]^+$ calculated for $C_{29}H_{25}NNaO_2Si$: 470.1547, found: 470.1551. The enantiomeric excess was determined by UPLC with Chiralpark INB column (hexanes:2-propanol = 90:10, 0.8 mL/min, 211 nm, 98% *ee*); major enantiomer tr = 26.920 min, minor enantiomer tr = 19.601 min.

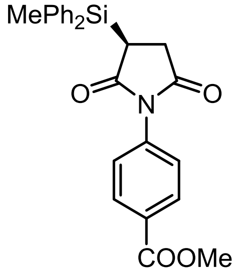

(*S*)-3h

(*S*)-ethyl 4-(3-(methyldiphenylsilyl)-2,5-dioxopyrrolidin-1-yl)benzoate (3h)

White solid (51 mg, 38% yield), mp 138 - 140 °C, purified by column chromatography (SiO_2, PE/EA= 5:1). $[\alpha]_D^{25}$ = +104.2 (c=0.71, $CHCl_3$).^{1}H NMR (400

MHz, $CDCl_3$) δ 7.95 (d, J = 8.8 Hz, 2H), 7.55 - 7.46 (m, 4H), 7.43 - 7.29 (m, 6H), 6.86 (d, J = 8.8 Hz, 2H), 4.29 (q, J = 7.2 Hz, 2H), 3.10 - 2.97 (m, 2H), 2.83 - 2.69 (m, 1H), 1.30 (t, J = 7.0 Hz, 3H), 0.73 (s, 3H). ^{13}C NMR (101 MHz, $CDCl_3$) δ 177.7, 175.4, 165.9, 136.0, 135.0, 134.9, 132.7, 131.8, 130.8, 130.6, 130.3, 130.2, 128.6, 126.3, 61.3, 32.0, 31.1, 14.4, -4.3. HRMS (ESI) m/z: $[M+Na]^+$ calculated for $C_{26}H_{25}NNaO_4Si$: 466.1445, found: 466.1435. The enantiomeric excess was determined by UPLC with Chiralpark INA column (hexanes:2-propanol = 90:10, 0.8 mL/min, 211 nm, 94% *ee*); major enantiomer tr = 14.472 min, minor enantiomer tr = 12.334 min.

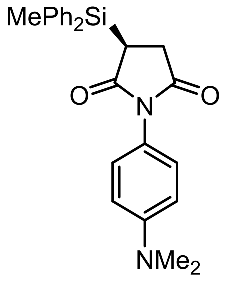

(*S*)-3i

(*S*)-1-(4-(dimethylamino)phenyl)-3-(methyldiphenylsilyl)pyrrolidine-2,5-dione (3i)

Pink solid (90.6 mg, 73% yield), mp 89 - 91 ^{o}C, purified by column chromatography (SiO_2, PE/EA= 4:1). $[\alpha]_D^{25}$ = +71.9 (c=0.90, $CHCl_3$). ^{1}H NMR (400 MHz, $CDCl_3$) δ 7.55 - 7.47 (m, 4H), 7.42 - 7.29 (m, 6H), 6.64 - 6.54 (m, 4H), 3.02 - 2.92 (m, 2H), 2.84 (s, 6H), 2.76 - 2.64 (m, 1H), 0.71 (s, 3H). ^{13}C NMR (100 MHz, $CDCl_3$) δ 178.7, 176.5, 150.4, 135.1, 134.9, 133.1, 132.2, 130.5, 130.4, 128.4, 127.2, 120.6, 112.5, 40.6, 31.8, 30.8, -4.3. HRMS (ESI) m/z: $[M+Na]^+$ calculated for $C_{25}H_{26}N_2NaO_2Si$: 437.1656, found: 437.1660. The enantiomeric excess was determined by UPLC with Chiralpark INA column (hexanes:2-propanol = 90:10, 0.8 mL/min, 211 nm, 99% *ee*); major enantiomer tr = 16.540 min, minor enantiomer tr = 19.545 min.

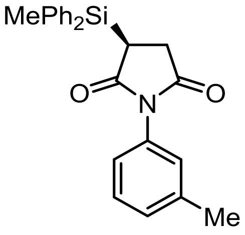

(*S*)-3j

(*S*)-3-(methyldiphenylsilyl)-1-(m-tolyl)pyrrolidine-2,5-dione (3j)

Yellow solid (100.9 mg, 87% yield), mp 95 - 97 ^{o}C, purified by column chromatography (SiO_2, PE/EA= 6:1). $[\alpha]_D^{25}$ = +97.8 (c=0.4, $CHCl_3$).^{1}H NMR (400 MHz, $CDCl_3$) δ 7.57 - 7.47 (m, 4H), 7.43 - 7.27 (m, 6H), 7.19 - 7.13 (m, 1H), 7.04 (d, *J* = 7.6 Hz, 1H), 6.57 (d, *J* = 7.6 Hz, 1H), 6.46 (s, 1H), 3.05 - 2.93 (m, 2H), 2.72 (dd, *J* = 23.2, 8.8 Hz, 1H), 2.22 (s, 3H), 0.79 – 0.67 (m, 3H). ^{13}C NMR (100 MHz, $CDCl_3$) δ 178.3, 176.0, 139.1, 135.1, 134.9, 132.9, 132.1, 132.0, 130.6, 130.5, 129.4, 128.9, 128.5, 127.3, 123.7, 32.0, 31.0, 21.3, -4.3. HRMS (ESI) m/z: $[M+Na]^+$ calculated for $C_{24}H_{23}NNaO_2Si$: 408.1390, found: 408.1391. The enantiomeric excess was determined by UPLC with Chiralpark INB column (hexanes:2-propanol = 90:10, 0.8 mL/min, 211 nm, 96% *ee*); major enantiomer tr = 12.511 min, minor enantiomer tr = 11.085 min.

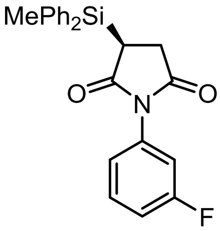

(*S*)-3k

(*S*)-1-(3-fluorophenyl)-3-(methyldiphenylsilyl)pyrrolidine-2,5-dione (3k)

Yellow oil (90.4 mg, 77% yield), purified by column chromatography (SiO_2, PE/EA= 6:1). $[\alpha]_D^{25}$= +6.5 (c=2.49, $CHCl_3$).^{1}H NMR (400 MHz, $CDCl_3$) δ 7.55 - 7.46 (m, 4H), 7.43 - 7.37 (m, 2H), 7.36 - 7.30 (m, 4H), 7.29 - 7.19 (m, 1H), 6.97 - 6.89 (m, 1H), 6.60 (d, *J* = 8.0 Hz, 1H), 6.50 - 6.43 (m, 1H), 3.07 - 2.96 (m, 2H), 2.79 - 2.67 (m, 1H), 0.72 (s, 3H). ^{13}C NMR (100 MHz, $CDCl_3$) δ 177.8, 175.4, 162.6 (d, *J* = 247.1 Hz),

135.0, 134.9, 134.1, 133.4 (d, *J* = 10.2 Hz), 132.2 (d, *J* = 84.3 Hz), 130.7 (d, *J* = 17.8 Hz), 130.1 (d, *J* = 8.9 Hz), 128.6, 128.0, 122.3 (d, *J* = 3.3 Hz), 115.5 (d, *J* = 20.9 Hz), 114.2 (d, *J* = 24.1 Hz), 31.9, 31.0, -4.3. ^{19}F NMR (471 MHz, $CDCl_3$) δ -110.92. HRMS (ESI) m/z: $[M+Na]^+$ calculated for $C_{23}H_{20}FNNaO_2Si$: 412.1140, found: 412.1148. The enantiomeric excess was determined by UPLC with Chiralpark INB column (hexanes:2-propanol = 90:10, 0.8 mL/min, 211 nm, 97% *ee*); major enantiomer tr = 12.991 min, minor enantiomer tr = 10.035 min.

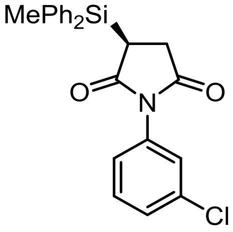

(*S*)-3l

(*S*)-1-(3-chlorophenyl)-3-(methyldiphenylsilyl)pyrrolidine-2,5-dione (3l)

White solid (87.2 mg, 72% yield), mp 96 - 98 ^{o}C, purified by column chromatography (SiO_2, PE/EA= 6:1). $[\alpha]_D^{25}$ = +61.5 (c=0.61, $CHCl_3$).^{1}H NMR (400 MHz, $CDCl_3$) δ 7.54 - 7.46 (m, 4H), 7.43 - 7.37 (m, 2H), 7.36 - 7.30 (m, 4H), 7.21 - 7.15 (m, 2H), 6.79 - 6.63 (m, 2H), 3.07 - 2.95 (m, 2H), 2.78 - 2.67 (m, 1H), 0.72 (s, 3H). ^{13}C NMR (100 MHz, $CDCl_3$) δ 177.7, 175.4, 135.1, 134.9, 134.5, 133.1, 132.6, 131.8, 130.8, 130.6, 129.9, 128.7, 128.6, 128.6, 126.9, 124.8, 31.9, 31.1, -4.3. HRMS (ESI) m/z: $[M+Na]^+$ calculated for $C_{23}H_{20}ClNNaO_2Si$: 428.0844, found: 428.0845. The enantiomeric excess was determined by UPLC with Chiralpark INB column (hexanes:2-propanol = 90:10, 0.8 mL/min, 211 nm, 97% *ee*); major enantiomer tr = 13.183 min, minor enantiomer tr = 10.570 min.

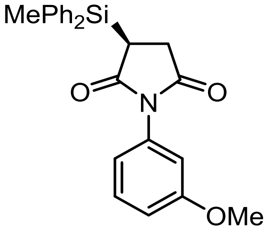

(*S*)-3m

(*S*)-1-(3-methoxyphenyl)-3-(methyldiphenylsilyl)pyrrolidine-2,5-dione (3m)

Yellow oil (87.1 mg, 72% yield), purified by column chromatography (SiO_2, PE/EA= 4:1). $[\alpha]_D^{25}$ = +68.1 (c=2.59, $CHCl_3$).^{1}H NMR (400 MHz, $CDCl_3$) δ 7.54 - 7.46 (m, 4H), 7.40 - 7.28 (m, 6H), 7.17 (t, *J* = 8.0 Hz, 1H), 6.77 (dd, *J* = 8.4, 2.4 Hz, 1H), 6.35 (d, *J* = 8.0 Hz, 1H), 6.26 (t, *J* = 2.0 Hz, 1H), 3.63 (s, 3H), 3.03 - 2.93 (m, 2H), 2.76 - 2.66 (m, 1H), 0.71 (s, 3H). ^{13}C NMR (100 MHz, $CDCl_3$) δ 178.1, 175.8, 160.0, 135.0, 134.9, 133.1, 132.8, 132.0, 130.6, 130.5, 129.7, 128.4, 118.9, 114.7, 112.2, 55.4, 31.9, 30.9, -4.3. HRMS (ESI) m/z: $[M+Na]^+$ calculated for $C_{24}H_{23}NNaO_3Si$: 424.1339, found: 424.1320. The enantiomeric excess was determined by UPLC with Chiralpark INB column (hexanes:2-propanol = 75:25, 0.8 mL/min, 211 nm, 95% *ee*); major enantiomer tr = 14.848 min, minor enantiomer tr = 13.787 min.

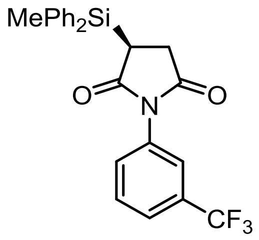

(*S*)-3n

(*S*)-3-(methyldiphenylsilyl)-1-(3-(trifluoromethyl)phenyl)pyrrolidine-2,5-dione (3n)

Yellow oil (74.7 mg, 57% yield), purified by column chromatography (SiO_2, PE/EA= 6:1). $[\alpha]_D^{25}$ = +31.4 (c=1.82, $CHCl_3$).^{1}H NMR (400 MHz, $CDCl_3$) δ 7.55 - 7.46 (m, 2H), 7.43 - 7.27 (m, 2H), 6.98 (d, *J* = 8.0 Hz, 1H), 6.87 (s, 1H), 3.11 - 2.97 (m, 1H), 2.82 - 2.68 (m, 1H), 0.73 (s, 1H). ^{13}C NMR (100 MHz, $CDCl_3$) δ 177.7, 175.4, 135.1, 134.9, 134.1, 132.6, 132.5, 131.7 (d, *J* = 6.0 Hz), 131.4, 130.0 (d, *J* = 5.8 Hz), 129.5, 128.6, 128.6, 128.0, 125.2 (d, *J* = 3.7 Hz), 123.8 (d, *J* = 3.9 Hz), 32.0, 31.2, -4.2. ^{19}F NMR (471 MHz, $CDCl_3$) δ -62.63. HRMS (ESI) m/z: $[M+Na]^+$ calculated for $C_{24}H_{20}F_3NNaO_2Si$: 462.1108, found: 462.1117. The enantiomeric excess was determined by UPLC with Chiralpark INB column (hexanes:2-propanol = 93:7, 0.8 mL/min, 211 nm, 96% *ee*); major enantiomer tr = 13.976 min, minor enantiomer tr = 10.815 min.

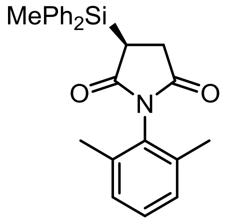

(*S*)-3o

(*S*)-1-(2,6-dimethylphenyl)-3-(methyldiphenylsilyl)pyrrolidine-2,5-dione (3o)

Yellow oil (118.8 mg, 99% yield), purified by column chromatography (SiO_2, PE/EA= 6:1). $[\alpha]_D^{25}$ = +59.2 (c=2.96, $CHCl_3$).^{1}H NMR (400 MHz, $CDCl_3$) δ 7.59 - 7.53 (m, 2H), 7.51 - 7.46 (m, 2H), 7.37 - 7.25 (m, 6H), 7.04 (t, *J* = 7.6 Hz, 1H), 6.97 (d, *J* = 7.6 Hz, 1H), 6.89 (d, *J* = 7.6 Hz, 1H), 3.16 - 2.94 (m, 2H), 2.78 (dd, *J* = 18.4, 4.4 Hz, 1H), 1.97 (s, 3H), 1.37 (s, 3H), 0.72 (s, 3H). ^{13}C NMR (100 MHz, $CDCl_3$) δ 177.8, 175.9, 135.9, 135.6, 135.3, 134.9, 133.4, 132.3, 130.5, 130.3, 129.2, 128.4, 128.3, 31.8, 30.7, 17.9, 17.1, -4.1. HRMS (ESI) m/z: $[M+Na]^+$ calculated for $C_{25}H_{25}NNaO_2Si$: 422.1547, found: 422.1545. The enantiomeric excess was determined by UPLC with Chiralpark INB column (hexanes:2-propanol = 90:10, 0.8 mL/min, 211 nm, 93% *ee*); major enantiomer tr = 15.127 min, minor enantiomer tr = 8.502 min.

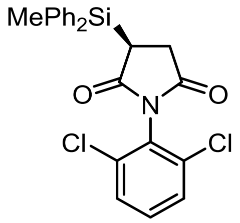

(*S*)-3p

(*S*)-1-(2,6-dichlorophenyl)-3-(methyldiphenylsilyl)pyrrolidine-2,5-dione (3p)

Yellow solid (115.8 mg, 88% yield), mp 98 - 100 ^{o}C, purified by column chromatography (SiO_2, PE/EA= 6:1). $[\alpha]_D^{25}$ = +39.5 (c=3.31, $CHCl_3$).^{1}H NMR (400 MHz, $CDCl_3$) δ 7.53 (t, *J* = 6.5 Hz, 4H), 7.40 - 7.19 (m, 8H), 7.14 (t, *J* = 8.0 Hz, 1H), 3.16 (dd, *J* = 10.1, 4.6 Hz, 1H), 3.02 (dd, *J* = 18.8, 10.1 Hz, 1H), 2.78 (dd, *J* = 18.8, 4.6 Hz, 1H), 0.73 (s, 3H). ^{13}C NMR (100 MHz, $CDCl_3$) δ 176.5, 174.6, 135.2, 135.0,

134.6, 134.4, 133.3, 132.3, 131.1, 130.5, 130.3, 128.8, 128.6, 128.5, 128.4, 128.3, 32.1, 30.8, -4.7. HRMS (ESI) m/z: $[M+Na]^+$ calculated for $C_{23}H_{19}Cl_2NNaO_2Si$: 462.0454, found: 462.0443.The enantiomeric excess was determined by UPLC with Chiralpark INB column (hexanes:2-propanol = 85:15, 0.8 mL/min, 230 nm, 94% *ee*); major enantiomer tr = 10.914 min, minor enantiomer tr = 8.393 min.

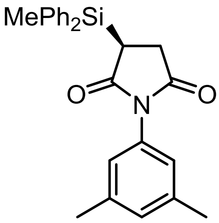

(*S*)-3q

(*S*)-1-(3,5-dimethylphenyl)-3-(methyldiphenylsilyl)pyrrolidine-2,5-dione (3q)

Yellow oil (87.3 mg, 73% yield), purified by column chromatography (SiO_2, PE/EA= 6:1). $[\alpha]_D^{25}$ = +72.6 (c=2.52, $CHCl_3$).^{1}H NMR (400 MHz, $CDCl_3$) δ 7.58 - 7.45 (m, 4H), 7.42 - 7.25 (m, 6H), 6.85 (s, 1H), 6.26 (s, 2H), 3.06 - 2.89 (m, 2H), 2.77 - 2.64 (m, 1H), 2.16 (s, 6H), 0.71 (s, 3H). ^{13}C NMR (100 MHz, $CDCl_3$) δ 178.3, 176.1, 138.8, 135.1, 134.9, 132.9, 132.1 131.8, 130.5, 130.4, 128.4, 128.4, 124.4, 31.9, 31.0, 21.2, -4.3. HRMS (ESI) m/z: $[M+Na]^+$ calculated for $C_{25}H_{25}NNaO_2Si$: 422.1547, found: 422.1549. The enantiomeric excess was determined by UPLC with Chiralpark INB column (hexanes:2-propanol = 90:10, 0.8 mL/min, 211 nm, 96% *ee*); major enantiomer tr = 11.187 min, minor enantiomer tr = 9.381 min.

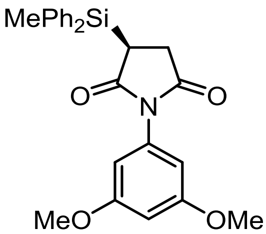

(*S*)-3r

(*S*)-1-(3,5-dimethoxyphenyl)-3-(methyldiphenylsilyl)pyrrolidine-2,5-dione (3r)

Yellow oil (78.4 mg, 61% yield), purified by column chromatography (SiO_2, PE/EA= 3:1). $[\alpha]_D^{25}$ = +76.4 (c=2.00, $CHCl_3$).^{1}H NMR (400 MHz, $CDCl_3$) δ 7.56 - 7.45 (m, 4H),

7.41 - 7.26 (m, 6H), 6.33 (t, *J* = 2.2 Hz, 1H), 5.88 (d, *J* = 2.0 Hz, 2H), 3.62 (s, 6H), 3.04 - 2.93 (m, 2H), 2.79 - 2.65 (m, 1H), 0.72 (s, 3H). ^{13}C NMR (100 MHz, $CDCl_3$) δ 176.9, 174.6, 159.9, 134.0, 133.8, 132.5, 131.8, 130.9, 129.5, 129.4, 127.4, 127.4, 104.0, 100.2, 54.4, 30.8, 29.8, -5.4. HRMS (ESI) m/z: $[M+Na]^+$ calculated for $C_{25}H_{25}NNaO_4Si$: 454.1445, found: 454.1444. The enantiomeric excess was determined by HPLC with Chiralpark phenomenex column (hexanes:2-propanol = 75:25, 0.8 mL/min, 222 nm, 96% *ee*); major enantiomer tr = 24.828 min, minor enantiomer tr = 22.911 min.

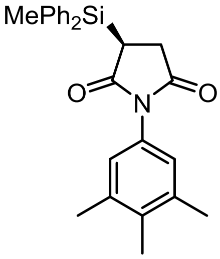

(*S*)-3s

(*S*)-3-(methyldiphenylsilyl)-1-(3,4,5-trimethylphenyl)pyrrolidine-2,5-dione (3s)

Yellow oil (92.2 mg, 74% yield), purified by column chromatography (SiO_2, PE/EA= 6:1). $[\alpha]_D^{25}$ = +87.4 (c=2.50, $CHCl_3$). ^{1}H NMR (400 MHz, $CDCl_3$) δ 7.69 (dd, *J* = 8.0, 1.2 Hz, 2H), 7.64 (dd, *J* = 8.0, 1.2 Hz, 2H), 7.58 - 7.44 (m, 6H), 6.44 (s, 2H), 3.18 - 3.08 (m, 2H), 2.91 - 2.80 (m, 1H), 2.28 (s, 6H), 2.18 (s, 3H), 0.87 (s, 3H). ^{13}C NMR (100 MHz, $CDCl_3$) δ 178.5, 176.2, 137.4, 136.0, 135.1, 134.9, 133.0, 132.1, 130.5, 130.4, 128.9, 128.5, 128.4, 125.6, 31.9, 31.0, 20.6, 15.3, -4.3. HRMS (ESI) m/z: $[M+Na]^+$ calculated for $C_{26}H_{27}NNaO_2Si$: 436.1703, found: 436.1706. The enantiomeric excess was determined by UPLC with Chiralpark INB column (hexanes:2-propanol = 90:10, 0.8 mL/min, 211 nm, 96% *ee*); major enantiomer tr = 12.083 min, minor enantiomer tr = 11.007 min.

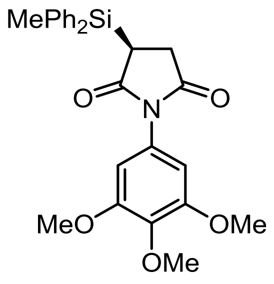

(*S*)-3t

(*S*)-3-(methyldiphenylsilyl)-1-(3,4,5-trimethoxyphenyl)pyrrolidine-2,5-dione (3t)

White solid (62.9 mg, 45% yield), mp 94 - 97 ^{o}C, purified by column chromatography (SiO_2, PE/EA= 6:1). $[\alpha]_D^{25}$ = +64.0 (c=1.42, $CHCl_3$). ^{1}H NMR (400 MHz, $CDCl_3$) δ 7.61 - 7.47 (m, 4H), 7.44 - 7.28 (m, 6H), 5.89 (s, 2H), 3.79 - 3.71 (m, 3H), 3.68 (s, 6H), 3.08 - 2.98 (m, 3H), 2.83 - 2.73 (m, 1H), 0.75 (s, 3H). ^{13}C NMR (100 MHz, $CDCl_3$) δ 178.3, 176.0, 153.5, 138.1, 135.2, 134.9, 132.8, 132.0, 130.6, 130.5, 128.5, 128.5, 127.6, 104.3, 60.8, 56.3, 31.9, 31.0, -4.1. HRMS (ESI) m/z: $[M+Na]^+$ calculated for $C_{26}H_{27}NNaO_5Si$: 484.1551, found: 484.1551. The enantiomeric excess was determined by HPLC with Chiralpark phenomenex column (hexanes:2-propanol = 80:20, 0.8 mL/min, 222 nm, 98% *ee*); major enantiomer tr = 36.002 min, minor enantiomer tr = 46.466 min.

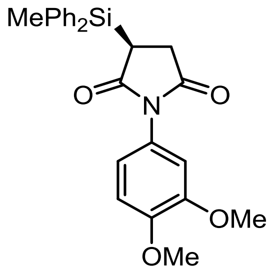

(*S*)-3u

(*S*)-1-(3,4-dimethoxyphenyl)-3-(methyldiphenylsilyl)pyrrolidine-2,5-dione (3u)

Yellow solid (66.1 mg, 51% yield), mp 66 - 68 ^{o}C, purified by column chromatography (SiO_2, PE/EA= 3:1). $[\alpha]_D^{25}$ = +74.3 (c=1.76, $CHCl_3$). ^{1}H NMR (400 MHz, $CDCl_3$) δ 7.58 - 7.45 (m, 4H), 7.42 - 7.27 (m, 6H), 6.75 (d, *J* = 8.8 Hz, 1H), 6.33 (dd, *J* = 8.4, 2.4 Hz, 1H), 6.17 (d, *J* = 2.4 Hz, 1H), 3.77 (s, 3H), 3.69 (s, 3H), 3.04 - 2.95 (m, 2H), 2.79 - 2.69 (m, 1H), 0.73 (s, 3H). ^{13}C NMR (100 MHz, $CDCl_3$) δ 178.4, 176.1, 149.3, 149.1, 135.1, 134.9, 132.9, 132.1, 130.6, 130.5, 128.5, 128.4,

124.8, 119.2, 111.2, 110.0, 31.9, 30.9, -4.2. HRMS (ESI) m/z: $[M+Na]^+$ calculated for $C_{25}H_{25}NNaO_4Si$: 454.1445, found: 454.1441. The enantiomeric excess was determined by HPLC with Chiralpark IA column (hexanes:2-propanol = 75:25, 0.8 mL/min, 220 nm, 94% *ee*); major enantiomer tr = 22.506 min, minor enantiomer tr = 29.012 min.

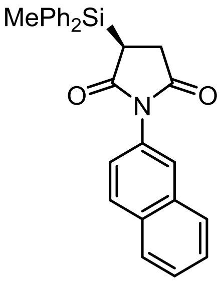

(*S*)-3v

(*S*)-3-(methyldiphenylsilyl)-1-(naphthalen-2-yl)pyrrolidine-2,5-dione (3v)

Yellow solid (69.6 mg, 55% yield), mp 115 - 117 ^oC, purified by column chromatography (SiO_2, PE/EA= 6:1). $[\alpha]_D^{25}$ = +63.4 (c=1.76, $CHCl_3$). ^1H NMR (400 MHz, $CDCl_3$) δ 7.76 - 7.69 (m, 2H), 7.68 - 7.64 (m, 1H), 7.58 - 7.48 (m, 4H), 7.45 - 7.29 (m, 8H), 7.19 (d, J = 2.0 Hz, 1H), 6.85 (dd, J = 8.8, 2.4 Hz, 1H), 3.14 - 2.97 (m, 2H), 2.85 - 2.71 (m, 1H), 0.74 (s, 3H). ^{13}C NMR (100 MHz, $CDCl_3$) δ 178.3, 176.0, 135.2, 134.9, 133.2, 132.9, 132.0, 130.7, 130.5, 128.9, 128.6, 128.5, 128.2, 127.8, 126.8, 126.5, 125.7, 124.1, 32.0, 31.1, -4.2. HRMS (ESI) m/z: $[M+Na]^+$ calculated for $C_{27}H_{23}NNaO_2Si$: 444.1390, found: 444.1390. The enantiomeric excess was determined by UPLC with Chiralpark INB column (hexanes:2-propanol = 90:10, 0.8 mL/min, 211 nm, 93% *ee*); major enantiomer tr = 23.613 min, minor enantiomer tr = 18.473 min.

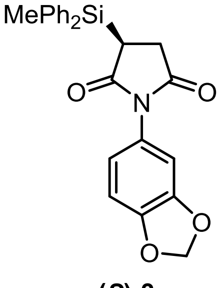

(*S*)-3w

(*S*)-1-(benzo[*d*][1,3]dioxol-5-yl)-3-(methyldiphenylsilyl)pyrrolidine-2,5-dione

(3w)

Yellow solid (84.2 mg, 68% yield), mp 103 - 105 °C, purified by column chromatography (SiO_2, PE/EA= 4:1). $[\alpha]_D^{25}$ = +77.6 (c=2.15, $CHCl_3$). ^1H NMR (400 MHz, $CDCl_3$) δ 7.57 - 7.46 (m, 4H), 7.43 - 7.37 (m, 2H), 7.33 (t, J = 7.2 Hz, 4H), 6.68 (d, J = 8.4 Hz, 1H), 6.20 (dd, J = 8.0, 2.0 Hz, 1H), 6.12 (d, J = 2.0 Hz, 1H), 5.86 (s, 2H), 3.04 - 2.92 (m, 2H), 2.79 - 2.65 (m, 1H), 0.72 (s, 3H). ^{13}C NMR (100 MHz, $CDCl_3$) δ 178.3, 176.0, 148.0, 147.7, 135.1, 134.9, 132.8, 132.0, 130.7, 130.5, 128.5 (d, J = 0.7 Hz), 125.6, 120.5, 108.4, 107.8, 101.8, 31.8, 30.9, -4.3. HRMS (ESI) m/z: $[M+Na]^+$ calculated for $C_{24}H_{21}NNaO_4Si$: 438.1132, found: 438.1139. The enantiomeric excess was determined by UPLC with Chiralpark INB column (hexanes:2-propanol = 80:20, 0.8 mL/min, 211 nm, 93% *ee*); major enantiomer tr = 14.654 min, minor enantiomer tr = 12.223 min.

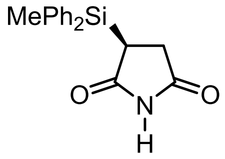

(*S*)-3x

(*S*)-3-(methyldiphenylsilyl)pyrrolidine-2,5-dione (3x)

White solid (61.5 mg, 69% yield), mp 119 - 121 °C, purified by column chromatography (SiO_2, PE/EA= 6:1). $[\alpha]_D^{25}$ = +20.1 (c=1.85, $CHCl_3$). ^1H NMR (400 MHz, $CDCl_3$) δ 8.13 (s, 1H), 7.56 - 7.43 (m, 4H), 7.41 - 7.26 (m, 6H), 2.99 - 2.79 (m, 2H), 2.55 (dd, J = 18.2, 3.1 Hz, 1H), 0.66 (s, 3H). ^{13}C NMR (100 MHz, $CDCl_3$) δ 179.5, 177.4, 134.9, 134.8, 133.0, 132.3, 130.6, 130.4, 128.5, 128.4, 33.1, 32.1, -4.7. HRMS (ESI) m/z: $[M+Na]^+$ calculated for $C_{17}H_{17}NNaO_2Si$: 318.0921, found: 318.0921. The enantiomeric excess was determined by UPLC with Chiralpark INB column (hexanes:2-propanol = 85:15, 0.8 mL/min, 211 nm, 81% *ee*); major enantiomer tr = 12.421 min, minor enantiomer tr = 11.704 min.

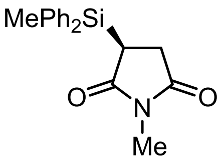

(*S*)-3y

(*S*)-1-methyl-3-(methyldiphenylsilyl)pyrrolidine-2,5-dione (3y)

Colorless oil (65.9 mg, 71% yield), purified by column chromatography (SiO_2, PE/EA= 6:1). $[\alpha]_D^{25}$ = +34.3 (c=1.72, $CHCl_3$).^{1}H NMR (400 MHz, $CDCl_3$) δ 7.49 - 7.45 (m, 2H), 7.45 - 7.40 (m, 2H), 7.39 - 7.32 (m, 3H), 7.32 - 7.26 (m, 3H), 2.90 - 2.75 (m, 2H), 2.68 (s, 3H), 2.51 (dd, J = 18.0, 2.4 Hz, 1H), 0.65 (s, 3H). ^{13}C NMR (100 MHz, $CDCl_3$) δ 179.2, 177.0, 134.8, 134.8, 133.0, 132.3, 130.5, 130.4, 128.4, 128.3, 31.7, 30.8, 24.7, -4.7. HRMS (ESI) m/z: $[M+Na]^+$ calculated for $C_{18}H_{19}NNaO_2Si$: 332.1087, found: 331.1082. The enantiomeric excess was determined by HPLC with Chiralpark IB column (hexanes:2-propanol = 90:10, 0.8 mL/min, 254 nm, 91% *ee*); major enantiomer tr = 13.579 min, minor enantiomer tr = 12.419 min.

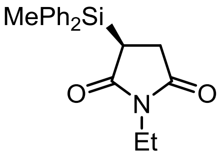

(*S*)-3z

(*S*)-1-ethyl-3-(methyldiphenylsilyl)pyrrolidine-2,5-dione (3z)

Yellow solid (86.4 mg, 89% yield), mp 86 - 89 ^{o}C, purified by column chromatography (SiO_2, PE/EA= 6:1). $[\alpha]_D^{25}$ = +31.6 (c=2.60, $CHCl_3$).^{1}H NMR (400 MHz, $CDCl_3$) δ 7.48 - 7.42 (m, 4H), 7.37 - 7.25 (m, 6H), 3.27 (q, J = 7.2 Hz, 2H), 2.85 - 2.73 (m, 2H), 2.52 (dd, J = 17.6, 2.4 Hz, 1H), 0.77 (t, J = 7.2 Hz, 3H), 0.65 (s, 3H). ^{13}C NMR (101 MHz, $CDCl_3$) δ 178.9, 176.8, 134.9, 134.8, 133.1, 132.3, 130.4, 130.3, 128.3, 128.3, 33.6, 31.6, 30.4, 12.7, -4.6. HRMS (ESI) m/z: $[M+Na]^+$ calculated for $C_{19}H_{21}NNaO_2Si$: 346.1234, found: 346.1244. The enantiomeric excess was determined by UPLC with Chiralpark INB column (hexanes:2-propanol = 90:10, 0.8 mL/min, 211 nm, 96% *ee*); major enantiomer tr = 7.436 min, minor enantiomer tr = 6.279 min.

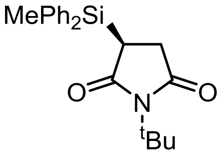

(*S*)-3aa

(*S*)-1-(tert-butyl)-3-(methyldiphenylsilyl)pyrrolidine-2,5-dione (3aa)

Colorless oil (79.4 mg, 75% yield), purified by column chromatography (SiO_2, PE/EA= 10:1). $[\alpha]_D^{25}$= +29.6 (c=1.66, $CHCl_3$.^{1}H NMR (400 MHz, $CDCl_3$) δ 7.50 - 7.43 (m, 4H), 7.38 - 7.33 (m, 2H), 7.33 - 7.28 (m, 4H), 2.77 - 2.66 (m, 2H), 2.50 - 2.39 (m, 1H), 1.27 (s, 9H), 0.65 (s, 3H). ^{13}C NMR (100 MHz, $CDCl_3$) δ 180.2, 178.0, 135.1, 134.9, 133.5, 132.6, 130.4, 130.3, 128.3, 58.2, 32.1, 30.6, 28.3, -4.4. HRMS (ESI) m/z: $[M+Na]^+$ calculated for $C_{21}H_{25}NNaO_2Si$: 374.1547, found: 374.1555. The enantiomeric excess was determined by UPLC with Chiralpark INA column (hexanes:2-propanol = 97:3, 0.8 mL/min, 211 nm, 92% *ee*); major enantiomer tr = 5.897 min, minor enantiomer tr = 5.316 min.

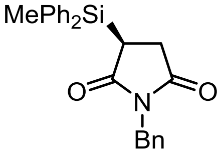

(*S*)-3bb

(*S*)-1-benzyl-3-(methyldiphenylsilyl)pyrrolidine-2,5-dione (3bb)

Colorless oil (99.0 mg, 86% yield), purified by column chromatography (SiO_2, PE/EA= 6:1). $[\alpha]_D^{25}$= +56.6 (c=2.91, $CHCl_3$).^{1}H NMR (400 MHz, $CDCl_3$) δ 7.44 - 7.38 (m, 2H), 7.36 - 7.23 (m, 6H), 7.19 (t, *J* = 7.3 Hz, 2H), 7.15 (s, 5H), 4.39 (s, 2H), 2.80 (ddd, *J* = 27.7, 13.8, 6.3 Hz, 2H), 2.57 – 2.44 (m, 1H), 0.56 (s, 3H). ^{13}C NMR (101 MHz, $CDCl_3$) δ 178.7, 176.6, 135.9, 134.8, 133.1, 132.3, 130.4, 130.3, 128.8, 128.6, 128.3, 127.8, 42.4, 31.6, 30.4, -4.7. HRMS (ESI) m/z: $[M+Na]^+$ calculated for $C_{24}H_{23}NNaO_2Si$: 408.1390, found: 408.1381. The enantiomeric excess was determined by UPLC with Chiralpark INA column (hexanes:2-propanol = 90:10, 0.8 mL/min, 211 nm, 91% *ee*); major enantiomer tr = 9.771 min, minor enantiomer tr = 8.997 min.

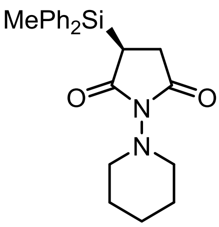

(*S*)-3cc

(*S*)-3-(methyldiphenylsilyl)-1-(piperidin-1-yl)pyrrolidine-2,5-dione (3cc)

Yellow oil (95.8 mg, 84% yield), purified by column chromatography (SiO_2, PE/EA= 6:1). $[\alpha]_D^{25}$= +45.3 (c=2.73, $CHCl_3$).^{1}H NMR (400 MHz, $CDCl_3$) δ 7.51 - 7.42 (m, 4H), 7.38 - 7.26 (m, 6H), 2.93 - 2.68 (m, 6H), 2.54 - 2.44 (m, 1H), 1.57 - 1.46 (m, 4H), 1.32 - 1.24 (m, 2H), 0.66 (s, 3H). ^{13}C NMR (101 MHz, $CDCl_3$) *δ* 177.8, 175.2, 135.0, 134.8, 133.0, 132.0, 130.4, 130.3, 128.3, 51.8, 29.9, 28.6, 25.9, 23.1, -4.6. HRMS (ESI) m/z: $[M+Na]^+$ calculated for $C_{22}H_{26}N_2NaO_2Si$: 401.1656, found: 401.1659. The enantiomeric excess was determined by UPLC with Chiralpark INB column (hexanes:2-propanol = 90:10, 0.8 mL/min, 211 nm, 96% *ee*); major enantiomer tr = 9.606 min, minor enantiomer tr = 6.852 min.

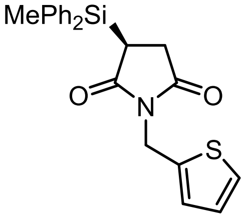

(*S*)-3dd

(*S*)-3-(methyldiphenylsilyl)-1-(thiophen-2-ylmethyl)pyrrolidine-2,5-dione (3dd)

Yellow oil (98.5 mg, 84% yield), purified by column chromatography (SiO_2, PE/EA= 6:1). $[\alpha]_D^{25}$= +44.0 (c=2.78, $CHCl_3$).^{1}H NMR (400 MHz, $CDCl_3$) *δ* 7.42 (dd, *J* = 8.0, 1.2 Hz, 2H), 7.37 - 7.24 (m, 6H), 7.19 (t, *J* = 7.2 Hz, 2H), 7.08 (dd, *J* = 5.2, 1.2 Hz, 1H), 6.89 (d, *J* = 2.4 Hz, 1H), 6.80 (dd, *J* = 5.4, 3.6 Hz, 1H), 4.56 (s, 2H), 2.88 - 2.69 (m, 2H), 2.58 - 2.42 (m, 1H), 0.57 (s, 3H). ^{13}C NMR (100 MHz, $CDCl_3$) *δ* 178.3, 176.1, 137.4, 134.8, 134.7, 133.0, 132.3, 130.4, 130.3, 128.3, 128.2, 128.0, 126.8, 125.9, 36.3, 31.7, 30.4, -4.9. HRMS (ESI) m/z: $[M+Na]^+$ calculated for $C_{22}H_{21}NNaO_2SSi$: 414.0954, found: 414.0951. The enantiomeric excess was determined by UPLC with Chiralpark INA column (hexanes:2-propanol = 93:7, 0.8

mL/min, 211 nm, 96% *ee*); major enantiomer tr = 7.886 min, minor enantiomer tr = 7.037 min.

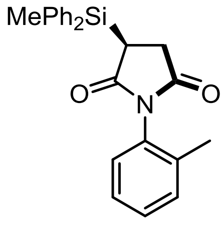

(*P*,*S*)-3ee

(*P,S*)-3-(methyldiphenylsilyl)-1-(o-tolyl)pyrrolidine-2,5-dione (3ee)

Yellow oil (94.3 mg, 82% yield), purified by column chromatography (SiO_2, PE/EA= 6:1). $[\alpha]_D^{25}$ = +70.4 (c=2.82, $CHCl_3$).^{1}H NMR (400 MHz, $CDCl_3$) δ 7.59 - 7.47 (m, 7H), 7.44 - 7.27 (m, 10H), 7.20 - 6.90 (m, 6H), 6.04 (d, *J* = 7.7 Hz, 1H), 3.11 - 2.93 (m, 3H), 2.82 - 2.71 (m, 2H), 2.02 (s, 3H), 1.44 (s, 2H), 0.73 (d, *J* = 1.9 Hz, 5H). ^{13}C NMR (100 MHz, $CDCl_3$) δ 178.2, 178.1, 176.0, 175.9, 135.8, 135.3, 135.2, 135.0, 134.9, 133.4, 132.9, 132.3, 132.0, 131.3, 131.1, 131.0, 130.7, 130.6, 130.5, 130.4, 129.4, 129.3, 128.5, 128.5, 128.4, 128.1, 127.8, 126.8, 126.7, 32.1, 31.8, 31.2, 30.8, 17.8, 17.1, -4.2, -4.2. HRMS (ESI) m/z: $[M+Na]^+$ calculated for $C_{24}H_{23}NNaO_2Si$: 408.1390, found: 408.1387. The enantiomeric excess was determined by HPLC with Chiralpark IB column (hexanes:2-propanol = 90:10, 0.8 mL/min, 211 nm, 96% *ee*, *d.r.* = 58:42); major enantiomer tr = 24.852 min, minor enantiomer tr = 17.386 min.

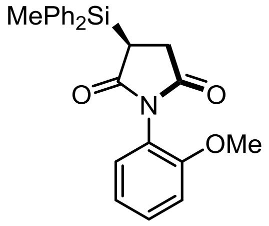

(*P*,*S*)-3ff

(*P,S*)-1-(2-methoxyphenyl)-3-(methyldiphenylsilyl)pyrrolidine-2,5-dione (3ff)

Yellow oil (115.5 mg, 96% yield), purified by column chromatography (SiO_2, PE/EA= 4:1). $[\alpha]_D^{25}$ = +68.4 (c=2.88, $CHCl_3$).^{1}H NMR (400 MHz, $CDCl_3$) δ 7.58 - 7.46 (m, 7H), 7.42 - 7.19 (m, 12H), 7.06 - 6.74 (m, 4H), 6.19 (dd, *J* = 7.7, 1.6 Hz, 1H), 3.65 (s, 3H), 3.60 (s, 2H), 3.10 - 2.85 (m, 3H), 2.76 - 2.63 (m, 2H), 0.71 (s, 3H), 0.69

(s, 2H). ^{13}C NMR (100 MHz, $CDCl_3$) δ 178.0, 177.9, 176.1, 175.9, 154.9, 154.7, 135.1, 135.1, 135.0, 134.9, 133.1, 133.1, 132.2, 130.6, 130.5, 130.4, 130.2, 130.1, 129.4, 129.0, 128.4, 128.3, 128.1, 121.0, 120.9, 120.8, 120.8, 112.1, 112.0, 55.8, 55.6, 32.1, 31.1, 30.7, 29.8, -4.3, -5.6. HRMS (ESI) m/z: $[M+Na]^+$ calculated for $C_{24}H_{23}NNaO_3Si$: 424.1339, found: 424.1350. The enantiomeric excess was determined by UPLC with Chiralpark INB column (hexanes:2-propanol = 92:8, 0.7 mL/min, 211 nm, 94% *ee*, *d.r.* = 59:41); major enantiomer tr = 28.084 min, minor enantiomer tr = 24.162 min.

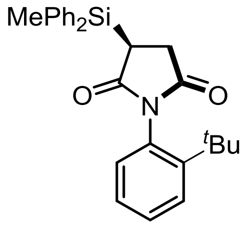

(*P,S*)-3gg

(*P,S*)-1-(2-(tert-butyl)phenyl)-3-(methyldiphenylsilyl)pyrrolidine-2,5-dione (3gg)

White solid (96.4 mg, 75% yield), mp 103 - 105 °C. purified by column chromatography (SiO_2, PE/EA= 6:1). $[\alpha]_D^{25}$ = +45.2 (c=4.03, $CHCl_3$). ^{1}H NMR (400 MHz, $CDCl_3$) δ 7.62 - 7.50 (m, 4H), 7.44 - 7.22 (m, 8H), 7.19 - 7.13 (m, 1H), 6.72 (dd, *J* = 7.7, 1.4 Hz, 1H), 3.11 (dd, *J* = 9.8, 6.4 Hz, 1H), 2.93 (dd, *J* = 18.6, 9.8 Hz, 1H), 2.78 (dd, *J* = 18.6, 6.4 Hz, 1H), 0.96 (s, 9H), 0.74 (s, 3H). ^{13}C NMR (100 MHz, $CDCl_3$) δ 179.09, 177.87, 148.39, 135.45, 135.08, 133.91, 132.91, 131.40, 131.10, 130.50, 130.27, 129.63, 128.47, 128.33, 127.48, 35.25, 32.33, 31.34, 31.25, -4.11. HRMS (ESI) m/z: $[M+Na]^+$ calculated for $C_{27}H_{29}NNaO_2Si$: 450.1860, found: 450.1864. The enantiomeric excess was determined by UPLC with Chiralpark INA column (hexanes:2-propanol = 90:10, 0.8 mL/min, 211 nm, 99% *ee*, *d.r.* = >99:1); major enantiomer tr = 11.332 min, minor enantiomer tr = 6.986 min.

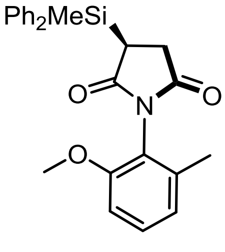

(*P,S*)-3hh

(*P,S*)-1-(2-methoxy-6-methylphenyl)-3-(methyldiphenylsilyl)pyrrolidine-2,5-dione (3hh)

Colorless oil (73 mg, 59% yield), purified by column chromatography (SiO_2, PE/EA= 6:1). $[\alpha]_D^{25}$ = +45.0 (c=2.19, $CHCl_3$). ^1H NMR (400 MHz, $CDCl_3$) δ 7.61 - 7.48 (m, 4H), 7.41 - 7.25 (m, 6H), 7.17 (t, *J* = 7.6 Hz, 1H), 6.79 (d, *J* = 7.6 Hz, 1H), 6.71 (d, *J* = 8.4 Hz, 1H), 3.58 (s, H), 3.09 (dd, *J* = 10.0, 4.4 Hz, 1H), 2.95 (dd, *J* = 18.8, 10.0 Hz, 1H), 2.70 (dd, *J* = 18.8, 4.4 Hz, 1H), 2.03 (s, 3H), 0.71 (s, 3H). ^{13}C NMR (100 MHz, $CDCl_3$) δ 178.0, 176.2, 154.9, 137.7, 135.1, 135.0, 133.9, 133.2, 130.3, 130.2, 130.1, 128.4, 128.1, 122.6, 120.1, 109.2, 55.6, 32.2, 30.7, 17.7, -5.8. HRMS (ESI) m/z: $[M+Na]^+$ calculated for $C_{25}H_{25}NNaO_3Si$: 438.1496, found: 438.1486. The enantiomeric excess was determined by UPLC with Chiralpark INB column (hexanes:2-propanol = 90:10, 0.8 mL/min, 211 nm, 93% *ee*, *d.r.* = 78:22); major enantiomer tr = 17.922 min, minor enantiomer tr = 12.075 min.

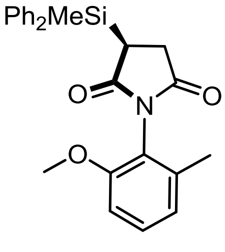

(*M,S*)-3hh'

(*M,S*)-1-(2-methoxy-6-methylphenyl)-3-(methyldiphenylsilyl)pyrrolidine-2,5-dion e (3hh')

Colorless oil (21.8 mg, 17% yield), purified by column chromatography (SiO_2, PE/EA= 6:1). $[\alpha]_D^{25}$ = +50.4 (c=0.59, $CHCl_3$). ^1H NMR (400 MHz, $CDCl_3$) δ 7.63 - 7.48 (m, 4H), 7.43 - 7.28 (m, 6H), 7.15 (dd, *J* = 16.4, 8.4 Hz, 1H), 6.69 (d, *J* = 8.0 Hz,

2H), 3.66 (s, 3H), 3.20 - 2.98 (m, 2H), 2.79 (dd, J = 18.3, 4.0 Hz, 1H), 1.45 (s, 3H), 0.74 (s, 3H). ^{13}C NMR (100 MHz, $CDCl_3$) δ 178.2, 176.4, 154.9, 137.8, 135.3, 135.0, 133.6, 132.5, 130.6, 130.3, 130.2, 128.5, 128.4, 122.6, 120.2, 109.1, 56.0, 32.0, 30.8, 16.9, -4.1. HRMS (ESI) m/z: $[M+Na]^+$ calculated for $C_{25}H_{25}NNaO_3Si$: 438.1496, found: 438.1479. The enantiomeric excess was determined by UPLC with Chiralpark INB column (hexanes:2-propanol = 90:10, 0.8 mL/min, 211 nm, 95% *ee, d.r.* = 78:22); major enantiomer tr = 18.939 min, minor enantiomer tr = 15.009 min.

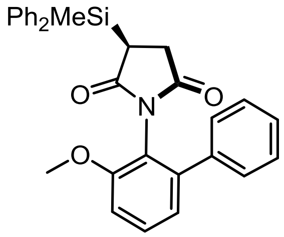

(*P*,*S*)-5a

(*P*,*S*)-1-(3-methoxy-[1,1'-biphenyl]-2-yl)-3-(methyldiphenylsilyl)pyrrolidine-2,5-dione (5a)

Colorless oil (117.3 mg, 82% yield), purified by column chromatography (SiO_2, PE/EA= 6:1). $[\alpha]_D^{25}$ = +38.6 (c=3.87, $CHCl_3$). ^{1}H NMR (400 MHz, $CDCl_3$) δ 7.58 - 7.42 (m, 4H), 7.39 - 7.16 (m, 12H), 6.95 - 6.86 (m, 2H), 3.64 (s, 3H), 2.73 (dd, J = 8.4, 5.6 Hz, 1H), 2.63 - 2.47 (m, 2H), 0.67 (s, 3H). ^{13}C NMR (100 MHz, $CDCl_3$) δ 178.2, 176.5, 155.2, 143.1, 138.6, 135.2, 135.0, 133.9, 133.1, 130.5, 130.2, 130.1, 128.3, 128.3, 128.1, 127.7, 122.4, 119.0, 110.8, 55.8, 32.0, 30.5, -5.7. HRMS (ESI) m/z: $[M+Na]^+$ calculated for $C_{30}H_{27}NNaO_3Si$: 500.1652, found: 500.1650.The enantiomeric excess was determined by UPLC with Chiralpark INB column (hexanes:2-propanol = 85:15, 0.8 mL/min, 254 nm, 94% *ee, d.r.* = 95:5); major enantiomer tr = 6.264 min, minor enantiomer tr = 7.068 min.

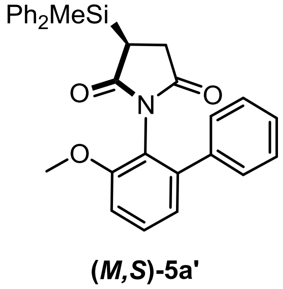

(*M*,*S*)-5a'

(*M,S*)-1-(3-methoxy-[1,1'-biphenyl]-2-yl)-3-(methyldiphenylsilyl)pyrrolidine-2,5-dione (5a')

Yellow oil (6.1 mg, 4% yield), purified by column chromatography (SiO_2, PE/EA= 4:1). $[\alpha]_D^{25}$= +28.2 (c=0.27, $CHCl_3$). ^1H NMR (400 MHz, $CDCl_3$) δ 7.45 (dd, *J* = 7.9, 1.4 Hz, 2H), 7.39 - 7.27 (m, 7H), 7.26 - 7.19 (m, 7H), 6.90 (dd, *J* = 8.1, 2.8 Hz, 2H), 3.72 (s, 3H), 3.04 (dd, *J* = 10.1, 5.3 Hz, 1H), 2.80 (dd, *J* = 18.7, 10.1 Hz, 1H), 2.37 (dd, *J* = 18.7, 5.3 Hz, 1H), -0.00 (s, 3H). ^{13}C NMR (100 MHz, $CDCl_3$) δ 178.23, 177.48, 155.00, 143.33, 138.76, 135.18, 134.76, 134.18, 133.61, 130.40, 130.19, 129.97, 128.60, 128.46, 128.40, 127.97, 127.91, 122.84, 119.36, 110.74, 56.25, 32.26, 30.37, -7.33. HRMS (ESI) m/z: $[M+Na]^+$ calculated for $C_{30}H_{27}NNaO_3Si$: 500.1652, found: 500.1671.The enantiomeric excess was determined by UPLC with Chiralpark INB column (hexanes:2-propanol = 85:15, 0.8 mL/min, 211 nm, 68% *ee*, *d.r.* = 95:5); major enantiomer tr = 8.868 min, minor enantiomer tr = 8.103 min.

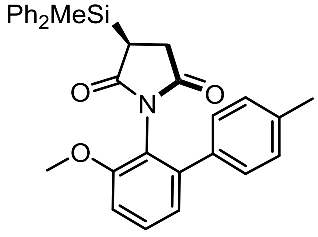

(*P,S*)-5b

(*P,S*)-1-(3-methoxy-4'-methyl-[1,1'-biphenyl]-2-yl)-3-(methyldiphenylsilyl) pyrrolidine-2,5-dione (5b)

Colorless oil (125.5 mg, 85% yield), purified by column chromatography (SiO_2, PE/EA= 6:1). $[\alpha]_D^{25}$= +43.4 (c=4.16, $CHCl_3$). ^1H NMR (400 MHz, $CDCl_3$) δ 7.53 - 7.42 (m, 4H), 7.36 - 7.22 (m, 7H), 7.09 - 7.00 (m, 4H), 6.92 - 6.81 (m, 2H), 3.62 (s, 3H), 2.75 (dd, *J* = 9.2, 4.8 Hz, 1H), 2.65 - 2.49 (m, 2H), 2.23 (s, 3H), 0.67 (s, 3H). ^{13}C NMR (100 MHz, $CDCl_3$) δ 178.1, 176.5, 155.1, 143.0, 137.3, 135.7, 135.0, 135.0, 133.9, 133.1, 130.4, 130.2, 130.1, 129.0, 128.3, 128.1, 122.4, 119.0, 110.6, 55.8, 32.0, 30.5, 21.3, -5.8. HRMS (ESI) m/z: $[M+Na]^+$ calculated for $C_{31}H_{29}NNaO_3Si$: 514.1809, found: 514.1805. The enantiomeric excess was determined by UPLC with Chiralpark

INB column (hexanes:2-propanol = 85:15, 0.8 mL/min, 211 nm, 92% *ee*, *d.r.* = 95:5); major enantiomer tr = 6.803 min, minor enantiomer tr = 8.145 min.

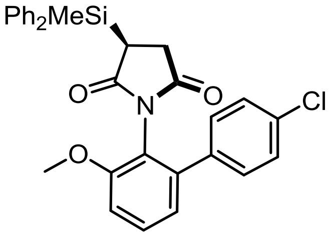

(*P,S*)-5c

(*P,S*)-1-(4'-chloro-3-methoxy-[1,1'-biphenyl]-2-yl)-3-(methyldiphenylsilyl) pyrrolidine-2,5-dione (5c)

Colorless oil (141.4 mg, 92% yield), purified by column chromatography (SiO_2, PE/EA= 6:1). $[\alpha]_D^{25}$ = +33.9 (c=4.18, $CHCl_3$). ^1H NMR (400 MHz, $CDCl_3$) δ 7.52 - 7.40 (m, 4H), 7.38 - 7.24 (m, 7H), 7.23 - 7.08 (m, 4H), 6.92 - 6.82 (m, 2H), 3.63 (s, 3H), 2.81 - 2.70 (m, 1H), 2.67 - 2.50 (m, 2H), 0.67 (s, 3H). ^{13}C NMR (100 MHz, $CDCl_3$) δ 178.1, 176.4, 155.2, 141.9, 137.1, 135.0, 134.9, 133.8, 133.7, 133.0, 130.6, 130.3, 130.1, 129.7, 128.5, 128.4, 128.3, 128.2, 128.1, 122.2, 119.0, 111.2, 55.8, 32.0, 30.5, -5.8. HRMS (ESI) m/z: $[M+Na]^+$ calculated for $C_{30}H_{26}ClNNaO_3Si$: 534.1263, found: 534.1255. The enantiomeric excess was determined by UPLC with Chiralpark INB column (hexanes:2-propanol = 85:15, 0.8 mL/min, 254 nm, 86% *ee*, *d.r.* = 94:6); major enantiomer tr = 5.873 min, minor enantiomer tr = 6.551 min.

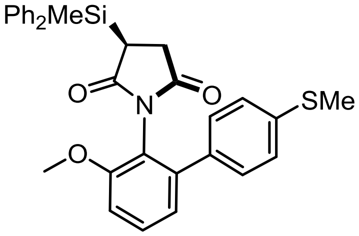

(*P,S*)-5d

(*P,S*)-1-(3-methoxy-4'-(methylthio)-[1,1'-biphenyl]-2-yl)-3-(methyldiphenylsilyl) pyrrolidine-2,5-dione (5d)

Yellow oil (47.6 mg, 30% yield), purified by column chromatography (SiO_2, PE/EA= 5:1). $[\alpha]_D^{25}$ = +27.4 (c=1.26, $CHCl_3$). ^1H NMR (400 MHz, $CDCl_3$) δ 7.55 - 7.44 (m, 4H),

7.39 - 7.24 (m, 7H), 7.11 (s, 4H), 6.94 - 6.86 (m, 2H), 3.65 (s, 3H), 2.79 (dd, *J* = 9.2, 4.8 Hz, 1H), 2.68 - 2.49 (m, 2H), 2.39 (s, 3H), 0.68 (s, 3H). ^{13}C NMR (100 MHz, $CDCl_3$) δ 178.2, 176.6, 155.2, 142.5, 138.1, 135.3, 135.1, 135.0, 133.9, 133.1, 130.6, 130.2, 130.1, 128.7, 128.3, 128.1, 126.0, 122.4, 119.0, 110.8, 55.9, 32.0, 30.6, 15.6, -5.7. HRMS (ESI) m/z: $[M+Na]^+$ calculated for $C_{31}H_{29}NNaO_3SSi$: 546.1530, found: 546.1529. The enantiomeric excess was determined by UPLC with Chiralpark INB column (hexanes:2-propanol = 85:15, 0.8 mL/min, 230 nm, 87% *ee*, *d.r.* = 81:19); major enantiomer tr = 10.855 min, minor enantiomer tr = 17.930 min.

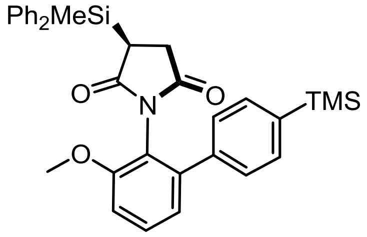

(*P,S*)-5e

(*P,S*)-1-(3-methoxy-4'-(trimethylsilyl)-[1,1'-biphenyl]-2-yl)-3-(methyldiphenylsilyl) pyrrolidine-2,5-dione (5e)

Colorless oil (141.3 mg, 86% yield), purified by column chromatography (SiO_2, PE/EA= 6:1). $[\alpha]_D^{25}$ = +33.5 (c=4.28, $CHCl_3$). ^{1}H NMR (400 MHz, $CDCl_3$) δ 7.54 - 7.43 (m, 4H), 7.42 - 7.22 (m, 9H), 7.17 (d, *J* = 7.6 Hz, 2H), 6.88 (dd, *J* = 11.2, 7.6 Hz, 2H), 3.62 (s, 3H), 2.77 (dd, *J* = 9.2, 5.2 Hz, 1H), 2.66 - 2.49 (m, 2H), 0.67 (s, 3H), 0.17 (s, 9H). ^{13}C NMR (100 MHz, $CDCl_3$) δ 178.2, 176.5, 155.1, 143.0, 139.7, 138.9, 135.1, 135.0, 133.9, 133.3, 133.1, 130.5, 130.2, 130.1, 128.3, 128.1, 127.5, 122.5, 118.9, 110.8, 55.8, 32.0, 30.5, -1.0, -5.8. HRMS (ESI) m/z: $[M+Na]^+$ calculated for $C_{33}H_{35}NNaO_3Si_2$: 572.2048, found: 572.2032. The enantiomeric excess was determined by UPLC with Chiralpark INB column (hexanes:2-propanol = 85:15, 0.8 mL/min, 254 nm, 93% *ee*, *d.r.* = 97:3); major enantiomer tr = 4.858 min, minor enantiomer tr = 6.336 min.

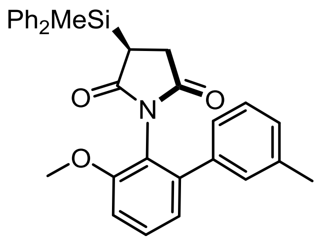

(*P*,*S*)-5f

(*M*,*S*)-1-(3-methoxy-3'-methyl-[1,1'-biphenyl]-2-yl)-3-(methyldiphenylsilyl) pyrrolidine-2,5-dione (5f)

Colorless oil (139.7 mg, 95% yield), purified by column chromatography (SiO_2, PE/EA= 6:1). $[\alpha]_D^{25}$ = +38.5 (c=4.07, $CHCl_3$). ^{1}H NMR (400 MHz, $CDCl_3$) δ 7.52 - 7.42 (m, 4H), 7.37 - 7.22 (m, 7H), 7.13 - 7.07 (m, 1H), 6.98 (dd, *J* = 12.4, 6.8 Hz, 3H), 6.91 - 6.83 (m, 2H), 3.62 (s, 3H), 2.73 (dd, *J* = 8.8, 5.2 Hz, 1H), 2.63 - 2.44 (m, 2H), 2.23 (s, 3H), 0.66 (s, 3H). ^{13}C NMR (100 MHz, $CDCl_3$) δ 178.0, 176.5, 155.1, 143.1, 138.5, 137.9, 135.1, 134.9, 133.9, 133.1, 130.4, 130.2, 130.1, 129.1, 128.4, 128.3, 128.1, 128.0, 125.2, 122.4, 119.0, 110.7, 55.8, 32.0, 30.5, 21.6, -5.8. HRMS (ESI) m/z: $[M+Na]^+$ calculated for $C_{31}H_{29}NNaO_3Si$: 514.1809, found: 514.1814. The enantiomeric excess was determined by UPLC with Chiralpark INB column (hexanes:2-propanol = 85:15, 0.8 mL/min, 211 nm, 94% *ee*, *d.r.* = 99:1); major enantiomer tr = 5.591 min, minor enantiomer tr = 7.092 min.

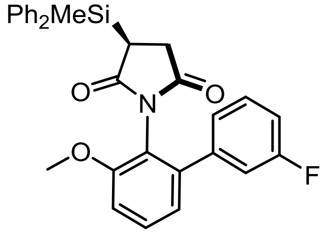

(*P*,*S*)-5g

(*P*,*S*)-1-(3'-fluoro-3-methoxy-[1,1'-biphenyl]-2-yl)-3-(methyldiphenylsilyl) pyrrolidine-2,5-dione (5g)

Colorless oil (136.3 mg, 92% yield), purified by column chromatography (SiO_2, PE/EA= 6:1). $[\alpha]_D^{25}$ = +39.9 (c=2.00, $CHCl_3$). ^{1}H NMR (400 MHz, $CDCl_3$) δ 7.53 - 7.41 (m, 4H), 7.37 - 7.11 (m, 8H), 6.98 - 6.84 (m, 5H), 3.62 (s, 3H), 2.77 (dd, *J* = 9.2, 5.2 Hz, 1H), 2.66 - 2.50 (m, 2H), 0.67 (s, 3H). ^{13}C NMR (100 MHz, $CDCl_3$) δ 178.0,

176.4, 162.5 (d, J = 244.9 Hz), 155.2, 141.7 (d, J = 1.9 Hz), 140.7 (d, J = 7.9 Hz), 135.0, 134.9, 133.7, 133.0, 130.6, 130.2, 130.1, 129.8 (d, J = 8.3 Hz), 128.3, 128.1, 124.1 (d, J = 2.9 Hz), 122.2, 118.9, 115.4 (d, J = 21.8 Hz), 114.6 (d, J = 20.8 Hz), 111.3, 55.8, 32.0, 30.5, -5.8. ^{19}F NMR (471 MHz, $CDCl_3$) δ -112.75. HRMS (ESI) m/z: $[M+Na]^+$ calculated for $C_{30}H_{26}FNNaO_3Si$: 518.1558, found: 518.1555. The enantiomeric excess was determined by UPLC with Chiralpark INB column (hexanes:2-propanol = 85:15, 0.8 mL/min, 254 nm, 94% *ee*, *d.r.* = 96:4); major enantiomer tr = 6.114 min, minor enantiomer tr = 7.110 min.

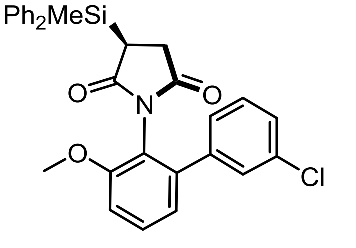

(*P*,*S*)-5h

(*P*,*S*)-1-(3'-chloro-3-methoxy-[1,1'-biphenyl]-2-yl)-3-(methyldiphenylsilyl) pyrrolidine-2,5-dione (5h)

Colorless oil (124.0 mg, 81% yield), purified by column chromatography (SiO_2, PE/EA= 6:1). $[\alpha]_D^{25}$ = +40.7 (c=0.76, $CHCl_3$). ^{1}H NMR (400 MHz, $CDCl_3$) δ 7.52 - 7.41 (m, 4H), 7.36 - 7.10 (m, 10H), 7.09 - 7.03 (m, 1H), 6.92 - 6.83 (m, 2H), 3.61 (s, 3H), 2.77 (dd, J = 9.6, 4.8 Hz, 1H), 2.69 - 2.49 (m, 2H), 0.66 (s, 3H). ^{13}C NMR (100 MHz, $CDCl_3$) δ 177.9, 176.3, 155.2, 141.5, 140.3, 135.0, 134.9, 134.0, 133.7, 133.0, 130.6, 130.2, 130.1, 129.5, 128.3, 128.2, 128.1, 127.8, 126.5, 122.1, 118.9, 111.3, 55.8, 32.0, 30.5, -5.8. HRMS (ESI) m/z: $[M+Na]^+$ calculated for $C_{30}H_{26}ClNNaO_3Si$: 534.1263, found: 534.1263. The enantiomeric excess was determined by UPLC with Chiralpark INB column (hexanes:2-propanol = 85:15, 0.8 mL/min, 254 nm, 95% *ee*, *d.r.* = 94:6); major enantiomer tr = 6.050 min, minor enantiomer tr = 7.056 min.

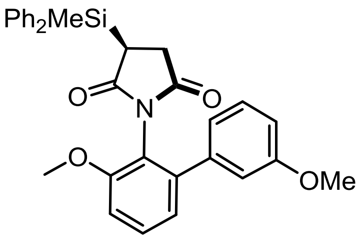

(*P*,*S*)-5i

(*P*,*S*)-1-(3,3'-dimethoxy-[1,1'-biphenyl]-2-yl)-3-(methyldiphenylsilyl)pyrrolidine-2,5-dione (5i)

Colorless oil (102.9 mg, 68% yield), purified by column chromatography (SiO_2, PE/EA= 5:1). $[\alpha]_D^{25}$ = +42.0 (c=2.97, $CHCl_3$). ^1H NMR (400 MHz, $CDCl_3$) δ 7.54 - 7.42 (m, 4H), 7.37 - 7.20 (m, 7H), 7.17 - 7.09 (m, 1H), 6.95 - 6.84 (m, 2H), 6.81 - 6.71 (m, 3H), 3.65 (s, 3H), 3.62 (s, 3H), 2.74 (dd, *J* = 9.2, 4.8 Hz, 1H), 2.66 - 2.49 (m, 2H), 0.66 (s, 3H). ^{13}C NMR (100 MHz, $CDCl_3$) δ 178.0, 176.5, 159.3, 155.1, 142.9, 139.9, 135.0, 134.9, 133.8, 133.1, 130.5, 130.2, 130.1, 129.2, 128.3, 128.1, 122.3, 120.6, 118.9, 113.7, 113.5, 110.9, 55.8, 55.2, 32.0, 30.5, -5.7. HRMS (ESI) m/z: $[M+Na]^+$ calculated for $C_{31}H_{29}NNaO_4Si$: 530.1758, found: 530.1766. The enantiomeric excess was determined by UPLC with Chiralpark INB column (hexanes:2-propanol = 85:15, 0.8 mL/min, 211 nm, 93% *ee*, *d.r.* = 91:9); major enantiomer tr = 10.098 min, minor enantiomer tr = 18.990 min.

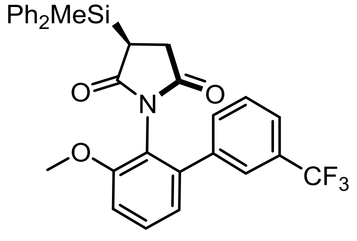

(*P*,*S*)-5j

(*P*,*S*)-1-(3-methoxy-3'-(trifluoromethyl)-[1,1'-biphenyl]-2-yl)-3-(methyldiphenylsilyl)pyrrolidine-2,5-dione (5j)

Colorless oil (149.0 mg, 91% yield), purified by column chromatography (SiO_2, PE/EA= 6:1). $[\alpha]_D^{25}$ = +33.4 (c=4.16, $CHCl_3$). ^1H NMR (400 MHz, $CDCl_3$) δ 7.54 - 7.42 (m, 6H), 7.42 - 7.22 (m, 9H), 6.91 (dd, *J* = 8.8, 2.4 Hz, 2H), 3.63 (s, 3H), 2.82 - 2.67 (m, 1H), 2.63 - 2.50 (m, 2H), 0.66 (s, 3H). ^{13}C NMR (100 MHz, $CDCl_3$) δ 178.0, 176.3, 155.3, 141.4, 139.4, 135.0, 134.9, 133.7, 133.0, 131.9, 130.8, 130.4, 130.3, 130.2, 129.0, 128.3, 128.1, 125.0 (d, *J* = 3.7 Hz), 124.5 (d, *J* = 3.7 Hz), 122.1, 119.1, 111.5, 55.9, 31.9, 30.5, -5.7. ^{19}F NMR (471 MHz, $CDCl_3$) δ -62.43. HRMS (ESI) m/z: $[M+Na]^+$ calculated for $C_{31}H_{26}F_3NNaO_3Si$: 568.1526, found: 568.1531. The enantiomeric excess was determined by UPLC with Chiralpark INA column

(hexanes:2-propanol = 85:15, 0.8 mL/min, 254 nm, 93% *ee*, *d.r.* = 99:1); major enantiomer tr = 4.209 min, minor enantiomer tr = 4.582 min.

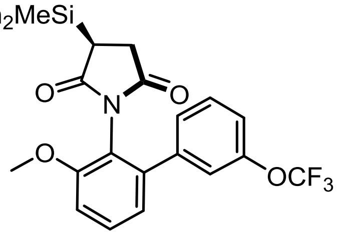

(*P,S*)-5k

(*P,S*)-1-(3-methoxy-3'-(trifluoromethoxy)-[1,1'-biphenyl]-2-yl)-3-(methyldiphenylsilyl)pyrrolidine-2,5-dione (5k)

Colorless oil (139.2 mg, 83% yield), purified by column chromatography (SiO_2, PE/EA= 6:1). $[\alpha]_D^{25}$ = +31.6 (c=3.31, $CHCl_3$). ^1H NMR (400 MHz, $CDCl_3$) δ 7.53 - 7.42 (m, 4H), 7.38 - 7.23 (m, 8H), 7.15 (d, *J* = 8.0 Hz, 1H), 7.11 - 7.04 (m, 2H), 6.90 (dd, *J* = 8.4, 4.4 Hz, 2H), 3.62 (s, 3H), 2.75 (dd, *J* = 8.4, 5.6 Hz, 1H), 2.66 - 2.49 (m, 2H), 0.66 (s, 3H). ^{13}C NMR (100 MHz, $CDCl_3$) δ 178.0, 176.4, 155.2, 148.9 (d, *J* = 1.5 Hz), 141.4, 140.6, 135.1, 135.0, 133.8, 133.0, 130.7, 130.3, 130.2, 130.0, 128.3, 128.1, 127.1, 122.2, 120.9, 120.3, 119.0, 111.5, 55.9, 31.9, 30.5, -5.7. ^{19}F NMR (471 MHz, $CDCl_3$) δ -57.70. HRMS (ESI) m/z: $[M+Na]^+$ calculated for $C_{31}H_{26}F_3NNaO_4Si$: 584.1475, found: 584.1466. The enantiomeric excess was determined by UPLC with Chiralpark INB column (hexanes:2-propanol = 93:7, 0.8 mL/min, 254 nm, 93% *ee*, *d.r.* = 99:1); major enantiomer tr = 5.928 min, minor enantiomer tr = 6.484 min.

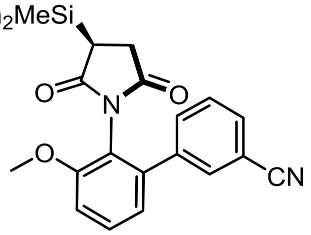

(*P,S*)-5l

(*P,S*)-3'-methoxy-2'-(3-(methyldiphenylsilyl)-2,5-dioxopyrrolidin-1-yl)-[1,1'-biphenyl]-3-carbonitrile (5l)

Colorless oil (89.4 mg, 59% yield), purified by column chromatography (SiO_2, PE/EA= 6:1). $[\alpha]_D^{25}$ = +37.7 (c=2.74, $CHCl_3$). ^1H NMR (400 MHz, $CDCl_3$) δ 7.59 - 7.24 (m, 15H), 6.95 (d, *J* = 8.0 Hz, 1H), 6.86 (d, *J* = 7.6 Hz, 1H), 3.64 (s, 3H), 2.77

(dd, *J* = 8.4, 5.6 Hz, 1H), 2.68 - 2.51 (m, 2H), 0.68 (s, 3H). ^{13}C NMR (100 MHz, $CDCl_3$) δ 177.9, 176.2, 155.2, 140.7, 139.9, 135.0, 134.9, 133.6, 132.9, 132.8, 131.8, 131.4, 130.8, 130.3, 130.2, 129.1, 128.3, 128.1, 122.0, 118.9, 118.6, 112.5, 111.8, 55.9, 31.9, 30.5, -5.8. HRMS (ESI) m/z: $[M+Na]^+$ calculated for $C_{31}H_{26}N_2NaO_3Si$: 525.1605, found: 525.1608. The enantiomeric excess was determined by UPLC with Chiralpark INA column (hexanes:2-propanol = 95:5, 0.8 mL/min, 211 nm, 91% *ee*, *d.r.* = 94:6); major enantiomer tr = 10.761 min, minor enantiomer tr = 12.994 min.

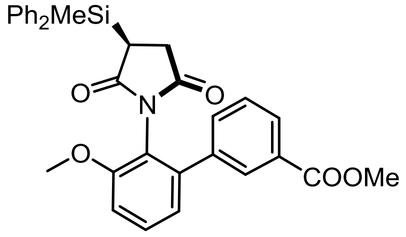

(*P*,*S*)-5m

(*P*,*S*)-1-(3-methoxy-3'-methyl-[1,1'-biphenyl]-2-yl)-3-(methyldiphenylsilyl) pyrrolidine-2,5-dione compound with carbon dioxide (1:1) (5m)

Colorless oil (104.4 mg, 65% yield), purified by column chromatography (SiO_2, PE/EA= 5:1). $[\alpha]_D^{25}$ = +39.7 (c=3.00, $CHCl_3$). ^{1}H NMR (400 MHz, $CDCl_3$) δ 7.89 (d, *J* = 8.4 Hz, 2H), 7.55 - 7.42 (m, 4H), 7.41 - 7.20 (m, 9H), 6.92 (t, *J* = 7.2 Hz, 2H), 3.79 (s, 3H), 3.64 (s, 3H), 2.75 (dd, *J* = 8.8, 5.2 Hz, 1H), 2.67 - 2.46 (m, 2H), 0.66 (s, 3H). ^{13}C NMR (100 MHz, $CDCl_3$) δ 178.0, 176.4, 167.0, 155.2, 141.8, 138.8, 135.0, 134.9, 133.8, 133.0, 132.7, 130.7, 130.4, 130.2, 130.1, 129.5, 128.9, 128.4, 128.3, 128.1, 122.2, 119.0, 111.3, 55.9, 52.3, 32.0, 30.5, -5.7. HRMS (ESI) m/z: $[M+Na]^+$ calculated for $C_{32}H_{29}NNaO_5Si$: 558.1707, found: 558.1717. The enantiomeric excess was determined by UPLC with Chiralpark INB column (hexanes:2-propanol = 80:20, 0.8 mL/min, 211 nm, 91% *ee*, *d.r.* = 96:4); major enantiomer tr = 7.533 min, minor enantiomer tr = 8.893 min.

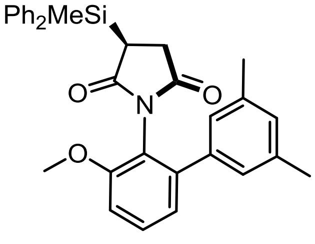

(*P*,*S*)-5n

(*P*,*S*)-1-(3-methoxy-3',5'-dimethyl-[1,1'-biphenyl]-2-yl)-3-(methyldiphenylsilyl) pyrrolidine-2,5-dione (5n)

Colorless oil (141.1 mg, 93% yield), purified by column chromatography (SiO_2, PE/EA= 6:1). $[\alpha]_D^{25}$ = +37.5 (c=4.19, $CHCl_3$). ^1H NMR (400 MHz, $CDCl_3$) δ 7.54 - 7.43 (m, 4H), 7.37 - 7.22 (m, 7H), 6.93 - 6.78 (m, 5H), 3.63 (s, 3H), 2.73 (dd, *J* = 8.8, 5.2 Hz, 1H), 2.64 - 2.49 (m, 2H), 2.19 (s, 6H), 0.67 (s, 3H). ^{13}C NMR (100 MHz, $CDCl_3$) *δ* 177.9, 176.5, 155.1, 143.1, 138.5, 137.6, 135.1, 135.0, 133.9, 133.1, 130.3, 130.2, 130.1, 129.3, 128.3, 128.1, 126.0, 122.5, 118.9, 110.6, 55.8, 32.0, 30.5, 21.4, -5.8. HRMS (ESI) m/z: $[M+Na]^+$ calculated for $C_{32}H_{31}NNaO_3Si$: 528.1965, found: 528.1967. The enantiomeric excess was determined by UPLC with Chiralpark INB column (hexanes:2-propanol = 85:15, 0.8 mL/min, 254 nm, 93% *ee*, *d.r.* = >99:1); major enantiomer tr = 4.747 min, minor enantiomer tr = 6.141 min.

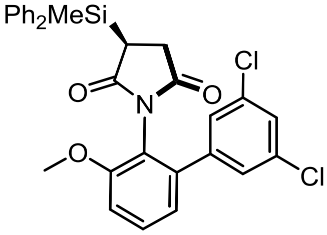

(*P*,*S*)-5o

(*P*,*S*)-1-(3',5'-dichloro-3-methoxy-[1,1'-biphenyl]-2-yl)-3-(methyldiphenylsilyl) pyrrolidine-2,5-dione (5o)

Colorless oil (141.1 mg, 86% yield), purified by column chromatography (SiO_2, PE/EA= 6:1). $[\alpha]_D^{25}$ = +33.0 (c=1.75, $CHCl_3$). ^1H NMR (400 MHz, $CDCl_3$) *δ* 7.54 - 7.43 (m, 4H), 7.36 - 7.19 (m, 8H), 7.08 (d, *J* = 2.0 Hz, 2H), 6.88 (dd, *J* = 22.0, 8.4 Hz, 2H), 3.62 (s, 3H), 2.82 (dd, *J* = 9.6, 4.4 Hz, 1H), 2.76 - 2.50 (m, 2H), 0.67 (s, 3H). ^{13}C

NMR (100 MHz, $CDCl_3$) δ 177.8, 176.3, 155.3, 141.4, 140.1, 135.0, 134.9, 134.8, 134.0, 133.6, 132.9, 130.8, 130.3, 130.2, 128.3, 128.1, 127.9, 126.8, 118.9, 111.8, 55.9, 32.0, 30.6, -5.8. HRMS (ESI) m/z: $[M+Na]^+$ calculated for $C_{30}H_{25}Cl_2NNaO_3Si$: 568.0873, found: 568.0875. The enantiomeric excess was determined by HPLC with Chiralpark IB column (hexanes:2-propanol = 97:3, 0.8 mL/min, 211 nm, 93% *ee*, *d.r.* = 97:3); major enantiomer tr = 14.081 min, minor enantiomer tr = 18.689 min.

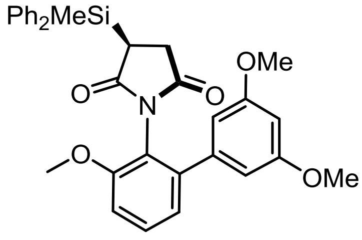

(*P,S*)-5p

(*P,S*)-3-(methyldiphenylsilyl)-1-(3,3',5'-trimethoxy-[1,1'-biphenyl]-2-yl) pyrrolidine-2,5-dione (5p)

Colorless oil (150.2 mg, 93% yield), purified by column chromatography (SiO_2, PE/EA= 3:1). $[\alpha]_D^{25}$= +35.3 (c=4.43, $CHCl_3$). ^1H NMR (400 MHz, $CDCl_3$) δ 7.54 - 7.42 (m, 4H), 7.38 - 7.20 (m, 7H), 6.96 - 6.84 (m, 2H), 6.35 (d, *J* = 2.0 Hz, 2H), 6.30 (t, *J* = 2.4 Hz, 1H), 3.62 (s, 6H), 3.61 (s, 3H), 2.76 (dd, *J* = 9.6, 4.8 Hz, 1H), 2.69 - 2.52 (m, 2H), 0.66 (s, 3H). ^{13}C NMR (100 MHz, $CDCl_3$) δ 177.9, 176.5, 160.4, 155.1, 142.9, 140.5, 135.1, 134.9, 133.8, 133.1, 130.5, 130.2, 130.1, 128.3, 128.1, 122.2, 118.9, 110.9, 106.2, 100.2, 55.8, 55.4, 32.1, 30.6, -5.8. HRMS (ESI) m/z: $[M+Na]^+$ calculated for $C_{32}H_{31}NNaO_5Si$: 560.1864, found: 560.1864. The enantiomeric excess was determined by UPLC with Chiralpark INB column (hexanes:2-propanol = 75:25, 0.8 mL/min, 211 nm, 92% *ee*, *d.r.* = >99:1); major enantiomer tr = 14.553 min, minor enantiomer tr = 27.246 min.

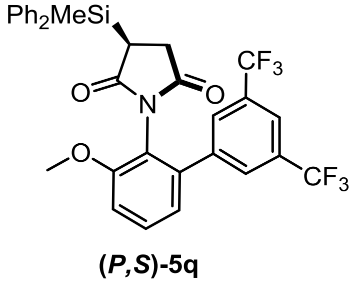

(*P,S*)-5q

(*P*,*S*)-1-(3-methoxy-3',5'-bis(trifluoromethyl)-[1,1'-biphenyl]-2-yl)-3-(methyldiphenylsilyl)pyrrolidine-2,5-dione (5q)

Colorless oil (168.1 mg, 91% yield), purified by column chromatography (SiO_2, PE/EA= 10:1). $[\alpha]_D^{25}$= +23.4 (c=5.13, $CHCl_3$). ^1H NMR (400 MHz, $CDCl_3$) δ 7.76 (s, 1H), 7.68 (s, 2H), 7.53 – 7.22 (m, 11H), 6.94 (dd, *J* = 10.4, 8.8 Hz, 2H), 3.63 (s, 3H), 2.74 (t, *J* = 7.2 Hz, 1H), 2.59 (d, *J* = 7.2 Hz, 2H), 0.66 (s, 3H). ^{13}C NMR (100 MHz, $CDCl_3$) δ 177.8, 176.2, 155.5, 140.7, 139.7, 135.0, 134.9, 133.6, 132.9, 131.7 (d, *J* = 33.2 Hz), 131.1, 130.3, 130.2, 128.7, 128.4, 128.2, 124.6, 121.9 (d, *J* = 5.2 Hz), 121.5 (d, *J* = 3.8 Hz), 119.1, 112.3, 56.0, 31.9, 30.5, -5.7. ^{19}F NMR (471 MHz, $CDCl_3$) δ -62.67. HRMS (ESI) m/z: $[M+Na]^+$ calculated for $C_{32}H_{25}F_6NNaO_3Si$: 636.1400, found: 636.1411. The enantiomeric excess was determined by UPLC with Chiralpark INA column (hexanes:2-propanol = 95:5, 0.8 mL/min, 254 nm, 91% *ee, d.r.* = >99:1); major enantiomer tr = 3.736 min, minor enantiomer tr = 4.226 min.

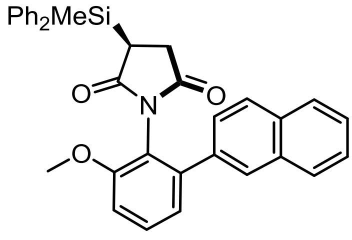

(*P*,*S*)-5r

(*P*,*S*)-1-(2-methoxy-6-(naphthalen-2-yl)phenyl)-3-(methyldiphenylsilyl) pyrrolidine-2,5-dione (5r)

Colorless oil (94.9 mg, 60% yield), purified by column chromatography (SiO_2, PE/EA= 6:1). $[\alpha]_D^{25}$= +50.8 (c=1.15, $CHCl_3$). ^1H NMR (400 MHz, $CDCl_3$) δ 7.78 - 7.64 (m, 4H), 7.52 - 7.21 (m, 14H), 7.00 (dd, *J* = 7.6, 0.8 Hz, 1H), 6.92 (d, *J* = 8.4 Hz, 1H), 3.66 (s, 3H), 2.67 (t, *J* = 7.2 Hz, 1H), 2.50 (d, *J* = 6.8 Hz, 2H), 0.65 (s, 3H). ^{13}C NMR (100 MHz, $CDCl_3$) δ 178.1, 176.6, 155.2, 143.0, 136.1, 135.1, 135.0, 133.8, 133.3, 133.1, 132.7, 130.6, 130.2, 130.1, 128.4, 128.3, 128.1, 127.8, 127.7, 127.3, 126.4, 126.3, 126.2, 122.7, 119.2, 110.9, 55.9, 32.0, 30.5, -5.7. HRMS (ESI) m/z: $[M+Na]^+$ calculated for $C_{34}H_{29}NNaO_3Si$: 550.1809, found: 550.1791. The enantiomeric excess was determined by UPLC with Chiralpark INB column

(hexanes:2-propanol = 85:15, 0.8 mL/min, 254 nm, 89% *ee*, *d.r.* = 92:8); major enantiomer tr = 8.263 min, minor enantiomer tr = 11.186 min.

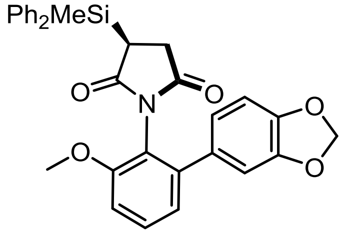

(*P*,*S*)-5s

(*P,S*)-1-(2-(benzo[*d*][1,3]dioxol-5-yl)-6-methoxyphenyl)-3-(methyldiphenylsilyl) pyrrolidine-2,5-dione (5s)

Colorless oil (152.3 mg, 97% yield), purified by column chromatography (SiO_2, PE/EA= 4:1). $[\alpha]_D^{25}$= +34.5 (c=1.41, $CHCl_3$). ^1H NMR (400 MHz, $CDCl_3$) δ 7.55 - 7.41 (m, 4H), 7.38 - 7.20 (m, 7H), 6.94 - 6.79 (m, 2H), 6.73 - 6.58 (m, 3H), 5.81 (s, 2H), 3.61 (s, 3H), 2.80 (dd, *J* = 9.6, 4.4 Hz, 1H), 2.71 - 2.50 (m, 2H), 0.67 (s, 3H). ^{13}C NMR (100 MHz, $CDCl_3$) δ 178.1, 176.5, 155.1, 147.5, 147.1, 142.6, 135.0, 134.9, 133.8, 133.0, 132.4, 130.4, 130.2, 130.1, 128.3, 128.1, 122.4, 121.6, 119.0, 110.6, 109.0, 108.1, 101.1, 55.8, 32.0, 30.5, -5.8. HRMS (ESI) m/z: $[M+Na]^+$ calculated for $C_{31}H_{27}NNaO_5Si$: 544.1551, found: 544.1552. The enantiomeric excess was determined by UPLC with Chiralpark INB column (hexanes:2-propanol = 85:15, 0.8 mL/min, 220 nm, 94% *ee*, *d.r.* = 99:1); major enantiomer tr = 15.545 min, minor enantiomer tr = 27.544 min.

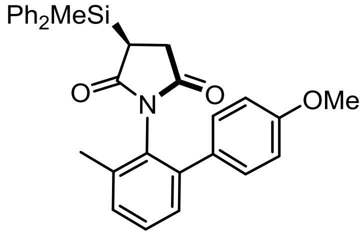

(*P*,*S*)-5t

(*P,S)*-1-(4'-methoxy-3-methyl-[1,1'-biphenyl]-2-yl)-3-(methyldiphenylsilyl) pyrrolidine-2,5-dione (5t)

Colorless oil (133.7 mg, 91% yield), purified by column chromatography (SiO_2, PE/EA= 6:1). $[\alpha]_D^{25}$= +22.0 (c=4.16, $CHCl_3$). ^1H NMR (400 MHz, $CDCl_3$) δ 7.54 - 7.41 (m, 4H), 7.39 - 7.26 (m, 6H), 7.20 (dd, *J* = 15.2, 7.6 Hz, 1H), 7.12 - 7.00 (m, 4H),

6.84 - 6.76 (m, 2H), 3.74 (s, 3H), 2.73 - 2.58 (m, 3H), 1.45 (s, 3H), 0.68 (s, 3H). ^{13}C NMR (100 MHz, $CDCl_3$) δ 178.1, 176.5, 159.0, 141.2, 136.6, 135.4, 135.0, 133.5, 132.3, 132.0, 130.6, 130.3, 130.0, 129.7, 129.5, 129.3, 128.5, 128.4, 113.7, 55.3, 31.8, 30.5, 17.3, -4.0. HRMS (ESI) m/z: $[M+Na]^+$ calculated for $C_{31}H_{29}NNaO_3Si$: 514.1809, found: 514.1811. The enantiomeric excess was determined by UPLC with Chiralpark INB column (hexanes:2-propanol = 95:5, 0.8 mL/min, 211 nm, 94% *ee*, *d.r.* = 95:5); major enantiomer tr = 8.250 min, minor enantiomer tr = 6.755 min.

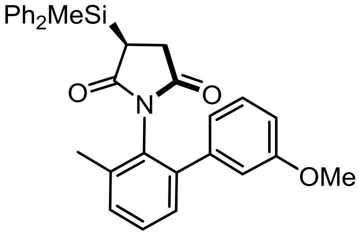

(*P*,*S*)-5u

(*P*,*S*)-1-(3'-methoxy-3-methyl-[1,1'-biphenyl]-2-yl)-3-(methyldiphenylsilyl) pyrrolidine-2,5-dione (5u)

Colorless oil (133.4 mg, 90% yield), purified by column chromatography (SiO_2, PE/EA= 6:1). $[\alpha]_D^{25}$= +29.6 (c=4.16, $CHCl_3$). ^{1}H NMR (400 MHz, $CDCl_3$) δ 7.51 (dd, *J* = 8.0, 1.2 Hz, 2H), 7.43 (dd, *J* = 7.6, 1.2 Hz, 2H), 7.39 - 7.07 (m, 10H), 6.80 - 6.75 (m, 1H), 6.73 - 6.67 (m, 2H), 3.68 (s, 3H), 2.72 - 2.55 (m, 3H), 1.45 (s, 3H), 0.67 (s, 3H). ^{13}C NMR (100 MHz, $CDCl_3$) δ 178.0, 176.5, 159.3, 141.4, 140.7, 136.7, 135.3, 134.9, 133.5, 132.3, 130.6, 130.3, 130.2, 129.5, 129.4, 129.2, 128.5, 128.3, 128.1, 120.7, 113.7, 113.6, 55.3, 31.8, 30.5, 17.2, -4.0. HRMS (ESI) m/z: $[M+Na]^+$ calculated for $C_{31}H_{29}NNaO_3Si$: 514.1809, found: 514.1809. The enantiomeric excess was determined by UPLC with Chiralpark INB column (hexanes:2-propanol = 95:5, 0.8 mL/min, 211 nm, 93% *ee, d.r.* = >99:1); major enantiomer tr = 6.595 min, minor enantiomer tr = 8.168 min.

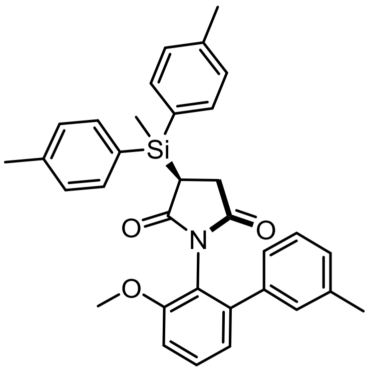

(*P,S*)-5v

(*P,S*)-1-(3-methoxy-3'-methyl-[1,1'-biphenyl]-2-yl)-3-(methyldi-p-tolylsilyl) pyrrolidine-2,5-dione (5v)

Colorless oil (148.3 mg, 95% yield), purified by column chromatography (SiO_2, PE/EA= 6:1). $[\alpha]_D^{25}$ = +36.8 (c=4.46, $CHCl_3$). ^1H NMR (400 MHz, $CDCl_3$) δ 7.42 - 7.26 (m, 5H), 7.14 - 7.05 (m, 5H), 7.03 - 6.94 (m, 3H), 6.88 (dd, *J* = 11.6, 7.6 Hz, 2H), 3.63 (s, 3H), 2.69 (dd, *J* = 8.4, 5.6 Hz, 1H), 2.61 - 2.48 (m, 2H), 2.26 (s, 3H), 2.23 (s, 6H), 0.63 (s, 1H). ^{13}C NMR (100 MHz, $CDCl_3$) δ 178.2, 176.6, 155.1, 143.1, 140.1, 140.0, 138.5, 137.8, 135.1, 135.0, 130.4, 130.3, 129.6, 129.1, 129.0, 128.9, 128.4, 128.0, 125.2, 122.4, 119.0, 110.7, 55.7, 32.0, 30.7, 21.6, 21.5, 21.5, -5.7. HRMS (ESI) m/z: $[M+Na]^+$ calculated for $C_{33}H_{33}NNaO_3Si$: 542.2122, found: 542.2111. The enantiomeric excess was determined by UPLC with Chiralpark INB column (hexanes:2-propanol = 85:15, 0.8 mL/min, 254 nm, 95% *ee*, *d.r.* = >99:1); major enantiomer tr = 4.799 min, minor enantiomer tr = 5.732 min.

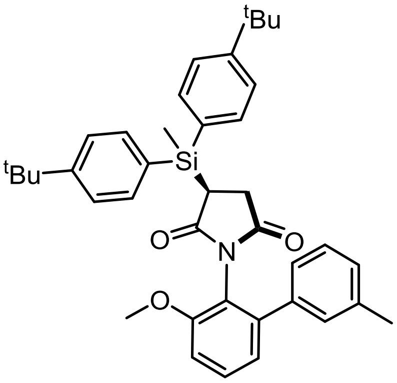

(*P,S*)-5w

(*P,S*)-3-(bis(4-(tert-butyl)phenyl)(methyl)silyl)-1-(3-methoxy-3'-methyl-[1,1'-biph

enyl]-2-yl)pyrrolidine-2,5-dione (5w)

Colorless oil (67.1 mg, 37% yield), purified by column chromatography (SiO_2, PE/EA= 6:1). $[\alpha]_D^{25}$= +30.2 (c=2.02, $CHCl_3$). ^1H NMR (400 MHz, $CDCl_3$) δ 7.43 (dd, J = 14.8, 8.0 Hz, 4H), 7.36 - 7.27 (m, 5H), 7.12 (dd, J = 18.0, 10.2 Hz, 1H), 7.04 - 6.86 (m, 5H), 3.67 (s, 3H), 2.73 (t, J = 6.4 Hz, 1H), 2.59 - 2.53 (m, 2H), 2.25 (s, 3H), 1.24 (s, 9H), 1.22 (s, 9H), 0.65 (s, 3H). ^{13}C NMR (100 MHz, $CDCl_3$) δ 178.3, 176.8, 155.2, 153.1, 153.0, 143.2, 138.6, 137.9, 135.0, 134.9, 130.6, 130.4, 129.7, 129.1, 128.4, 128.1, 125.3, 125.1, 122.5, 119.1, 110.7, 55.8, 34.9, 34.8, 32.2, 31.3, 31.2, 30.8, 21.6, -5.7. HRMS (ESI) m/z: $[M+Na]^+$ calculated for $C_{39}H_{45}NNaO_3Si$: 626.3061, found: 626.3050. The enantiomeric excess was determined by UPLC with Chiralpark INB column (hexanes:2-propanol = 85:15, 0.8 mL/min, 254 nm, 92% *ee, d.r.* = >99:1); major enantiomer tr = 3.661 min, minor enantiomer tr = 4.748 min.

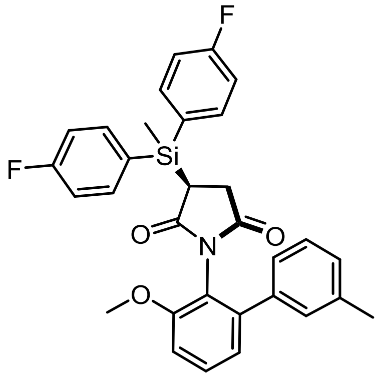

(*P*,*S*)-5x

(*P*,*S*)-3-(bis(4-fluorophenyl)(methyl)silyl)-1-(3-methoxy-3'-methyl-[1,1'-biphenyl] -2-yl)pyrrolidine-2,5-dione (5x)

Colorless oil (139.0 mg, 88% yield), purified by column chromatography (SiO_2, PE/EA= 6:1). $[\alpha]_D^{25}$= +33.9 (c=3.81, $CHCl_3$). ^1H NMR (400 MHz, $CDCl_3$) δ 7.51 - 7.38 (m, 4H), 7.31 (t, J = 8.4 Hz, 1H), 7.11 (dd, J = 15.2, 7.2 Hz, 1H), 7.05 - 6.83 (m, 9H), 3.64 (s, 3H), 2.70 (dd, J = 9.6, 4.4 Hz, 1H), 2.59 (dd, J = 18.4, 9.6 Hz, 1H), 2.47 (dd, J = 18.4, 4.4 Hz, 1H), 2.24 (s, 3H), 0.64 (s, 3H). ^{13}C NMR (100 MHz, $CDCl_3$) δ 177.8, 176.2, 165.6 (d, J = 4.8 Hz), 163.1 (d, J = 4.6 Hz), 155.1, 143.1, 138.5, 137.9, 137.2 (d, J = 7.6 Hz), 137.0 (d, J = 7.8 Hz), 130.5, 129.1 (d, J = 3.9 Hz), 129.0, 128.5 (d, J = 3.8 Hz), 128.4, 128.0, 125.1, 122.5, 118.8, 115.7 (d, J = 19.9 Hz), 115.4 (d, J =

19.8 Hz), 110.7, 55.8, 31.8, 30.5, 21.5, -5.6. ^{19}F NMR (471 MHz, $CDCl_3$) δ -109.45, -109.80. HRMS (ESI) m/z: $[M+Na]^+$ calculated for $C_{31}H_{27}F_2NNaO_3Si$: 550.1620, found: 550.1610. The enantiomeric excess was determined by UPLC with Chiralpark INA column (hexanes:2-propanol = 90:10, 0.8 mL/min, 211 nm, 91% *ee*, *d.r.* = 97:3); major enantiomer tr = 5.808 min, minor enantiomer tr = 6.696 min.

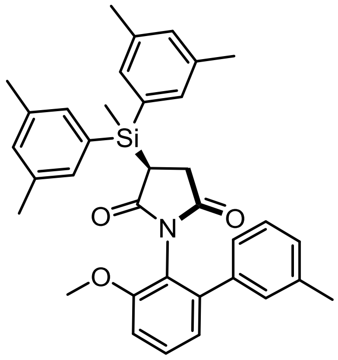

(*P*,*S*)-5y

(*P*,*S*)-3-(bis(3,5-dimethylphenyl)(methyl)silyl)-1-(3-methoxy-3'-methyl-[1,1'-biphenyl]-2-yl)pyrrolidine-2,5-dione (5y)

Colorless oil (155.0 mg, 94% yield), purified by column chromatography (SiO_2, PE/EA= 6:1). $[\alpha]_D^{25}$ = +34.1 (c=4.88, $CHCl_3$). ^{1}H NMR (400 MHz, $CDCl_3$) δ 7.29 (t, *J* = 8.0 Hz, 1H), 7.16 - 7.08 (m, 3H), 7.07 - 6.83 (m, 9H), 3.61 (s, 3H), 2.74 (dd, *J* = 8.4, 5.6 Hz, 1H), 2.62 - 2.48 (m, 2H), 2.24 (s, 3H), 2.20 (s, 6H), 2.19 (s, 6H), 0.63 (s, 3H). ^{13}C NMR (100 MHz, $CDCl_3$) δ 178.2, 176.6, 155.2, 143.1, 138.6, 137.8, 137.5, 137.3, 133.9, 133.1, 132.7, 132.6, 131.9, 131.8, 130.3, 129.1, 128.4, 128.0, 125.2, 122.4, 119.1, 110.7, 55.7, 32.3, 30.6, 21.6, 21.5, 21.44, -5.9. HRMS (ESI) m/z: $[M+Na]^+$ calculated for $C_{35}H_{37}NNaO_3Si$: 570.2435, found: 570.2419. The enantiomeric excess was determined by UPLC with Chiralpark INA column (hexanes:2-propanol = 95:5, 0.8 mL/min, 211 nm, 95% *ee, d.r.* = >99:1); major enantiomer tr = 3.900 min, minor enantiomer tr = 4.450 min.

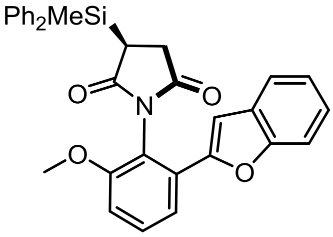

(*P*,*S*)-5z

(*P*,*S*)-1-(2-(benzofuran-2-yl)-6-methoxyphenyl)-3-(methyldiphenylsilyl) pyrrolidine-2,5-dione (5z)

Colorless oil (135.0 mg, 87% yield), purified by column chromatography (SiO_2, PE/EA= 5:1). $[\alpha]_D^{25}$= +33.6 (c=2.15, $CHCl_3$). ^1H NMR (400 MHz, $CDCl_3$) δ 7.59 - 7.48 (m, 4H), 7.43 (d, *J* = 7.6 Hz, 1H), 7.39 - 7.22 (m, 9H), 7.19 - 7.05 (m, 2H), 6.86 (dd, *J* = 8.0, 1.2 Hz, 1H), 6.77 (s, 1H), 3.60 (s, 3H), 3.13 - 2.92 (m, 2H), 2.74 (dd, *J* = 18.8, 4.0 Hz, 1H), 0.71 (s, 3H). ^{13}C NMR (100 MHz, $CDCl_3$) δ 178.1, 176.4, 155.8, 154.9, 153.1, 135.1, 135.0, 133.8, 133.1, 130.7, 130.4, 130.3, 130.1, 128.5, 128.3, 128.1, 124.8, 123.1, 121.3, 120.5, 118.0, 111.9, 111.0, 105.0, 55.9, 32.3, 30.8, -5.7. HRMS (ESI) m/z: $[M+Na]^+$ calculated for $C_{32}H_{27}NNaO_4Si$: 540.1602, found: 540.1626. The enantiomeric excess was determined by UPLC with Chiralpark INB column (hexanes:2-propanol = 80:20, 0.8 mL/min, 211 nm, 95% *ee*, *d.r.* = 92:8); major enantiomer tr = 13.981 min, minor enantiomer tr = 12.556 min.

Transformations of silyl products

Gram reaction

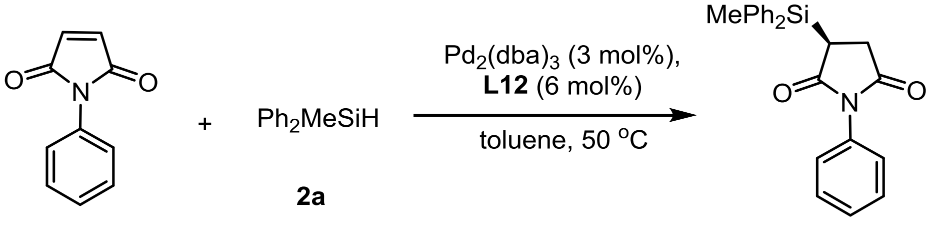

1a, 0.87g, 5 mmol

3a, 1.37g,
74% yield, 96% *ee*

A vial was charged with N-phenyl maleimide **1a** (0.87 g, 5.0 mmol), $Pd_2(dba)_3$ (137.4 mg, 3.0 mol%), (*R*,*R*)-**L12** (332.9 mg, 6 mol%) and evacuated under high vacuum and backfilled with N_2. Toluene (50 mL) was next added. The mixture was stirred at 25 °C for 10 minutes, then the mixture was stirred at 50 ℃ in a preheated oil. Upon reaction completion, the mixture was filtered over a plug of silica gel (washed with 50 ml EtOAc), and the filtrate was concentrated. The filtrate was concentrated and the residue was purified by SiO_2 chromatography to afford 1.37 g (74% yield, 96% *ee*) of (*S*)-3-(methyldiphenylsilyl)-1-phenylpyrrolidine-2,5-dione.

Procedure for the synthesis of 6.[9]

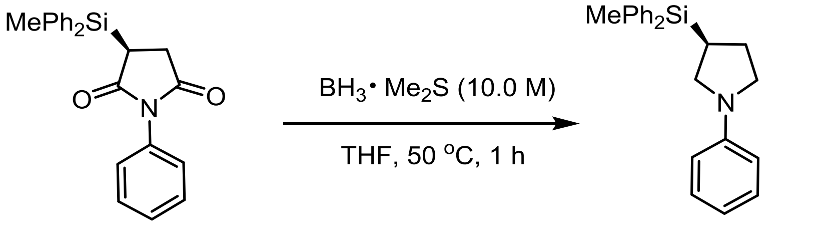

3a
96% *ee*

6
66% yield, 95% *ee*

3a (74 mg, 0.2 mmol, 1.0 equiv.) was dissolved in 1 mL of dry THF in an oven-dried tube. Borane dimethyl sulfide complex (0.2 mL of a 10.0 M solution) was added and the reaction was heated at 50 ℃ for 1 h. .The reaction was cooled in an ice bath. Methanol (10 mL) was slowly added, followed by 15 mL of water. This mixture was extracted with EtOAc (2×5) and the combined organics were dried over Na_2SO_4 and concentrated. The residue was taken up in dichloromethane and the resulting

suspension was filtered. The filtrate was concentrated and the residue was purified by SiO_2 chromatography to afford 45.3 mg (66% yield, 95% *ee*) of 3-(methyldiphenylsilyl)-1-phenylpyrrolidine.

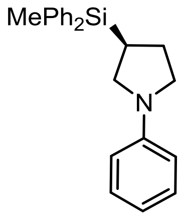

6

(*S*)-3-(methyldiphenylsilyl)-1-phenylpyrrolidine (6)

Colorless oil (45.3 mg, 66% yield), purified by column chromatography (SiO_2, PE/EA= 100:1). $[\alpha]_D^{25}$= +12.3 (c=1.39, $CHCl_3$). ^{1}H NMR (400 MHz, $CDCl_3$) δ 7.48 (dd, *J* = 9.2, 3.6 Hz, 4H), 7.38 - 7.24 (m, 6H), 7.12 (dd, *J* = 13.4, 5.6 Hz, 2H), 6.68 - 6.33 (m, 3H), 3.43 (t, *J* = 8.5 Hz, 1H), 3.31 - 3.07 (m, 3H), 2.23 - 1.72 (m, 3H), 0.53 (s, 3H). ^{13}C NMR (100 MHz, $CDCl_3$) δ 147.7, 135.9, 135.7, 134.8, 134.8, 129.7, 129.6, 129.2, 128.1, 128.1, 115.5, 111.8, 50.1, 48.8, 28.1, 24.8, -5.9. HRMS (ESI) m/z: $[M+H]^+$ calculated for $C_{23}H_{26}NSi$: 344.1829, found: 344.1828. The enantiomeric excess was determined by HPLC with Chiralpark OD column (hexanes:2-propanol = 99:1, 0.8 mL/min, 254 nm, 95% *ee*); major enantiomer tr = 11.441 min, minor enantiomer tr = 10.605 min.

Procedure for the synthesis of 7.[10]

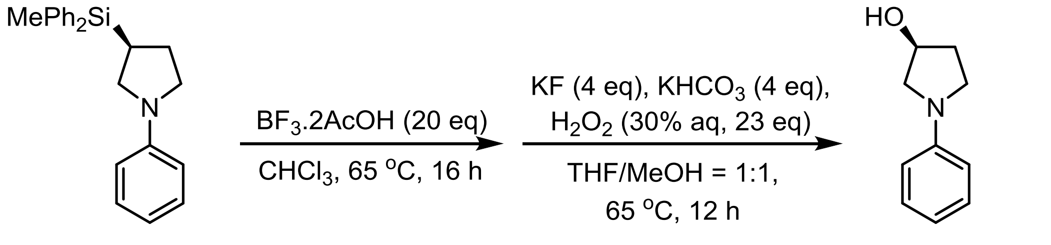

6
95% *ee*

7
70% yield, 96% *ee*

To a solution of 6 (0.2 mmol, 1.0 equiv.) in $CHCl_3$ (2.4 mL) was added trifluoroborane-acetic acid complex (0.56 mL, 4.0 mmol, 20.0 equiv.) and allowed to reflux for 16 h at 65 ^{o}C. The reaction mixture was cooled to room temperature and

then quenched with saturated aqueous $NaHCO_3$ solution (10 mL). The aqueous phase was extracted with CH_2Cl_2 (3 × 10 mL). The combined organic layers were dried over $MgSO_4$, filtered, and concentrated under reduced pressure to afford dark orange oil which was used immediately in the next step. To a crude product solution in THF and MeOH (2 mL / 2 mL) were added potassium fluoride (46.4 mg, 0.8 mmol, 4.0 equiv.), potassium hydrogen carbonate (80.0 mg, 0.8 mmol, 4.0 equiv.), and hydrogen peroxide (30% w/w solution in water, 0.47 mL, 4.6 mmol, 23.0 equiv.). The reaction was allowed to reflux for 12 h at 65 ^{o}C. The mixture was cooled to room temperature and quenched with saturated aqueous $Na_2S_2O_3$ solution (15 mL). The resulting aqueous layer was extracted with EtOAc (15mL × 3). The combined organic layers were dried over $MgSO_4$, filtered, concentrated under reduced pressure. The crude product was purified by column chromatography on silica gel with n-hexane/EtOAc to give the desired diol.

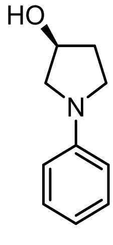

7

(*S*)-1-phenylpyrrolidin-3-ol (7)

White solid (22.7 mg, 70% yield), mp 61 - 63 ^{o}C. purified by column chromatography (SiO_2, PE/EA= 3:1). $[\alpha]_D^{25}$ = -4.2 (c=0.61, $CHCl_3$). ^{1}H NMR (400 MHz, $CDCl_3$) δ 7.25 - 7.11 (m, 2H), 6.62 (t, J = 7.3 Hz, 1H), 6.50 (d, J = 8.0 Hz, 2H), 4.51 - 4.44 (m, 1H), 3.48 - 3.35 (m, 2H), 3.32 - 3.21 (m, 1H), 3.17 (d, J = 10.4 Hz, 1H), 2.24 (dd, J = 32.7, 25.2 Hz, 1H), 2.14 - 2.02 (m, 1H), 2.00 - 1.91 (m, 1H). ^{13}C NMR (100 MHz, $CDCl_3$) δ 147.81, 129.31, 116.23, 112.01, 71.42, 56.38, 45.72, 34.26. HRMS (ESI) m/z: $[M+H]^+$ calculated for $C_{10}H_{14}NO$: 164.1070, found: 164.1076. The enantiomeric excess was determined by UPLC with Chiralpark MD column (hexanes:2-propanol = 80:20, 0.8 mL/min, 254 nm, 96% *ee*); major enantiomer tr = 4.896 min, minor enantiomer tr = 6.293 min.

Procedure for the synthesis of 8.[11]

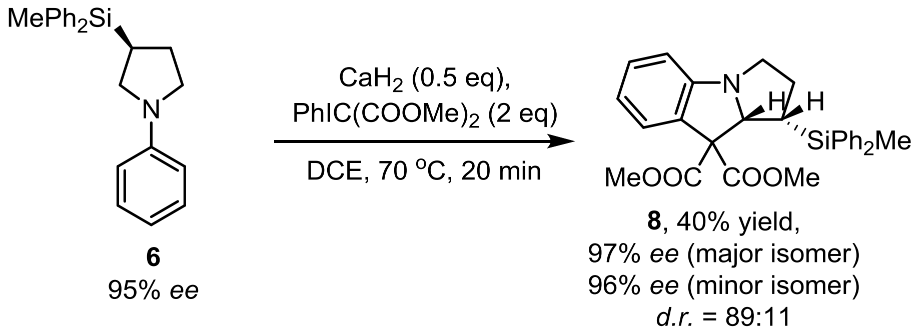

To a reaction mixture of iodonium ylide (0.6 mmol, 2.0 equiv) and CaH_2 (0.15 mmol, 0.5 equiv) in 1.0 mL freshly distilled dry DCE under a nitrogen atmosphere was added tertiary arylamine 6 (0.3 mmol, 1.0 equiv). Then the reaction mixture was stirred at 70 ^{o}C for several minutes. After the completion of the reaction as judged by TLC analysis (the reaction generally completed when the solid iodonium ylide disappeared), the solvent was evaporated and the reaction mixture was purified by flash chromatography on silica gel to give the desired products **8**. The determination of the structure of product 8 by NOESY spectrum (**Supplementary Figure 1**).

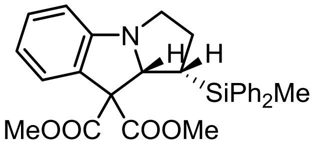

8

dimethyl (1*S*,9a*S*)-1-(methyldiphenylsilyl)-1,2,3,9a-tetrahydro-9H-pyrrolo[1,2-a] indole-9,9-dicarboxylate (8)

Yellow oil (55.9 mg, 40% yield), purified by column chromatography (SiO_2, PE/EA= 10:1). $[\alpha]_D^{25}$= +102.9 (c=1.39, $CHCl_3$).^{1}H NMR (400 MHz, $CDCl_3$) δ 7.44 - 7.36 (m, 5H， major + minor), 7.35 - 7.23 (m, 7H， major + minor), 7.16 (dd, *J* = 8.6, 6.6 Hz, 1H， major + minor), 6.84 (t, *J* = 7.5 Hz, 1H， major), 6.79 (t, *J* = 7.6 Hz, 1H， minor), 6.64 (d, *J* = 7.9 Hz, 1H， major), 6.48 (d, *J* = 7.8 Hz, 1H， minor), 4.75 - 4.65 (m, 1H， minor), 4.64 - 4.47 (m, 1H， major), 3.67 (s, 3H， minor), 3.66 (s, 3H， major), 3.63 (s, 3H， major), 3.60 (s, 3H， minor), 3.59 - 3.50 (m, 1H， major), 3.49 - 3.42 (m, 1H， minor), 3.13 (t, *J* = 11.4 Hz, 1H， major), 2.90 (t, *J* = 10.9 Hz, 1H， minor), 1.91 - 1.79 (m, 1H， major + minor), 1.72 - 1.52 (m, 2H， major + minor),

0.50 (s, 3H， major), 0.37 (s, 3H， minor). ^{13}C NMR (100 MHz, $CDCl_3$) δ 169.57, 168.98, 135.55, 135.48, 134.75, 134.72, 134.56, 134.06, 130.36, 129.67, 128.13, 128.10, 127.69, 69.94, 66.40, 53.44, 52.83, 30.13, 22.82, -5.77. HRMS (ESI) m/z: $[M+Na]^+$ calculated for $C_{28}H_{29}NNaO_4Si$: 494.1758, found: 494.1768. The enantiomeric excess was determined by UPLC with Chiralpark MD column (hexanes:2-propanol = 95:5, 0.8 mL/min, 211 nm, 97% major *ee*, 96% minor *ee*, *d.r.* = 89:11); major enantiomer tr = 7.594 min, major enantiomer tr = 12.059 min, minor enantiomer tr = 6.231 min, minor enantiomer tr = 14.531 min.

Procedure for the synthesis of 9.[12]

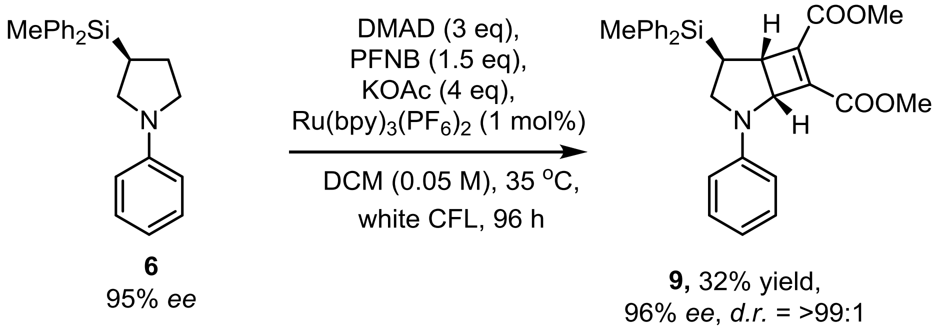

dimethyl (4*S*)-4-(methyldiphenylsilyl)-2-phenyl-2-azabicyclo[3.2.0]hept-6-ene-6,7-dicarboxylate (9)

$Ru(bpy)_3(PF_6)_2$ (0.85 mg, 0.001mmol, 1 mol%) and KOAc (39.2 mg, 0.4mmol, 4.0 equiv) were weighed into an oven-dried 8 mL vial with a magnetic stirring bar, and then DCM (0.05M, 2 mL) was added. The corresponding alkynester (0.3mmol, 3.0 equiv), 1,2,3,4,5-pentafluoro-6-nitrobenzene (PFNB, 0.15 mmol, 18.8 μL, 1.5 equiv) and amine (0.1 mmol, 1.0 equiv) were successively added into the mixture via syringes. The reaction mixture was degassed by three cycles of freeze-pump-thaw. After the mixture was thoroughly degassed, the vial was placed beside a 23-W compact fluorescent light (CFL, 1-2 centimeters from the vial), allowing the temperature to rise due to its proximity to the lights. After 96h, the crude mixture was concentrated and purified by flash chromatography (silica gel, mixtures of PE/EA)

to afford the pure product 9. The determination of the structure of product 9 by NOESY spectrum (**Supplementary Figure 2**).

Yellow oil (31.0 mg, 32% yield), purified by column chromatography (SiO_2, PE/EA= 10:1). $[\alpha]_D^{25}$ = +202.2 (c=0.08, $CHCl_3$). ^1H NMR (400 MHz, $CDCl_3$) δ 7.52 (dd, *J* = 7.5, 1.7 Hz, 2H), 7.46 (dd, *J* = 7.7, 1.5 Hz, 2H), 7.32 - 7.25 (m, 6H), 7.14 - 7.08 (m, 2H), 6.68 (d, *J* = 8.7 Hz, 3H), 4.81 (d, *J* = 3.8 Hz, 1H), 3.74 (s, 3H), 3.68 (d, *J* = 10.6 Hz, 1H), 3.63 (d, *J* = 2.9 Hz, 1H), 3.49 (s, 3H), 3.40 (dd, *J* = 9.9, 7.7 Hz, 1H), 2.17 (d, *J* = 7.3 Hz, 1H), 0.48 (s, 3H). ^{13}C NMR (100 MHz, $CDCl_3$) δ 162.35, 161.56, 146.18, 142.48, 135.62, 135.40, 134.92, 134.85, 129.66, 128.96, 128.14, 128.08, 118.02, 114.37, 60.78, 52.15, 51.93, 47.33, 21.81, -5.73. HRMS (ESI) m/z: $[M+Na]^+$ calculated for $C_{29}H_{29}NNaO_4Si$: 506.1758, found: 506.1744. The enantiomeric excess was determined by HPLC with Chiralpark AD column (hexanes:2-propanol = 95:5, 0.8 mL/min, 254 nm, 96% *ee*， *d.r.* = >99:1); major enantiomer tr = 8.794 min, minor enantiomer tr = 12.407 min.

Supplementary Tables

I. Optimizing of reaction condition

Supplementary Table 1. The effect of P-ligands L1-L13 on the palladium-catalyzed hydrosilylation reaction.[a]

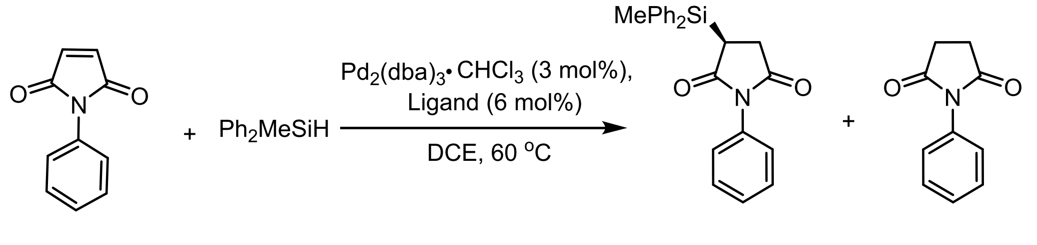

1a **2a** **3a** **4a**

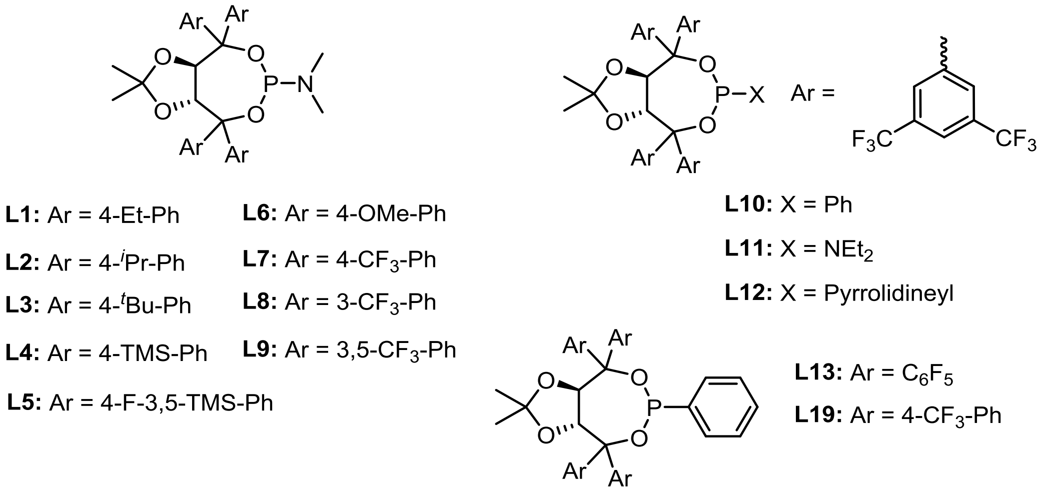

Entry	Ligand	Yield of **3a** (%)[b]	Yield of **4a** (%)[b]	*ee* of **3a** (%)[c]
1	**L1**	24	73	70
2	**L2**	16	79	69
3	**L3**	30	70	68
4	**L4**	29	71	76
5	**L5**	nr	nr	/
6	**L6**	8	92	62
7	**L7**	59	41	79
8	**L8**	47	46	79
9	**L9**	41	59	93
10	**L10**	4	86	91
11	**L11**	4	96	86
12	**L12**	68	32	94
13	**L13**	4	91	31

[a]All the reactions were run on a 0.1 mmol scale in 1.0 mL solvents for 18 h. [b]Determined by ^1H NMR using dibromomethane as an internal standard. [c]Determined by HPLC.

Supplementary Table 2. Screen of palladium metal salts for hydrosilylation reaction.[a]

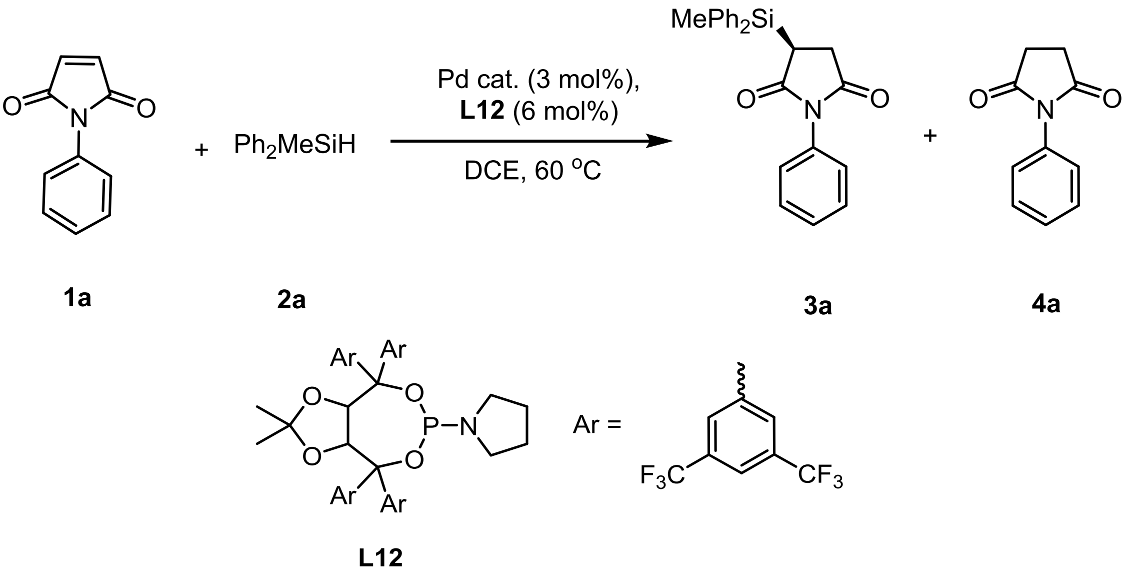

Entry	Pd cat.	Yield of **3a** (%)[b]	Yield of **4a** (%)[b]	*ee* of **3a** (%)[c]
1	[PdCl(η-cinnamoyl)]$_2$	82	18	94
2	[PdCl(allyl)]$_2$	37	63	93
3	$PdCl_2$(dtbpf)	nr	nr	/
4	$PdCl_2$ (MeCN)$_2$	28	57	92
5	$PdBr_2$	nr	nr	/
6	Pd(TFA)$_2$	47	53	90
7	Pd(acetylacetonato)$_2$	34	66	93
8	[PdCl(2-methallyl)$_2$]$_2$	40	46	93
9	PdOAc	44	38	94
10	$PdCl_2$(nbe)	9	30	93
11	Pd_2(dba)$_3$	85	15	94
12	Pd_2(dba)$_3$.$CHCl_3$	68	32	94

[a]All the reactions were run on a 0.1 mmol scale in 1.0 mL solvents for 18 h. [b]Determined by ^{1}H NMR using dibromomethane as an internal standard. [c]Determined by HPLC.

Supplementary Table 3. Screen of solvents for hydrosilylation reaction.[a]

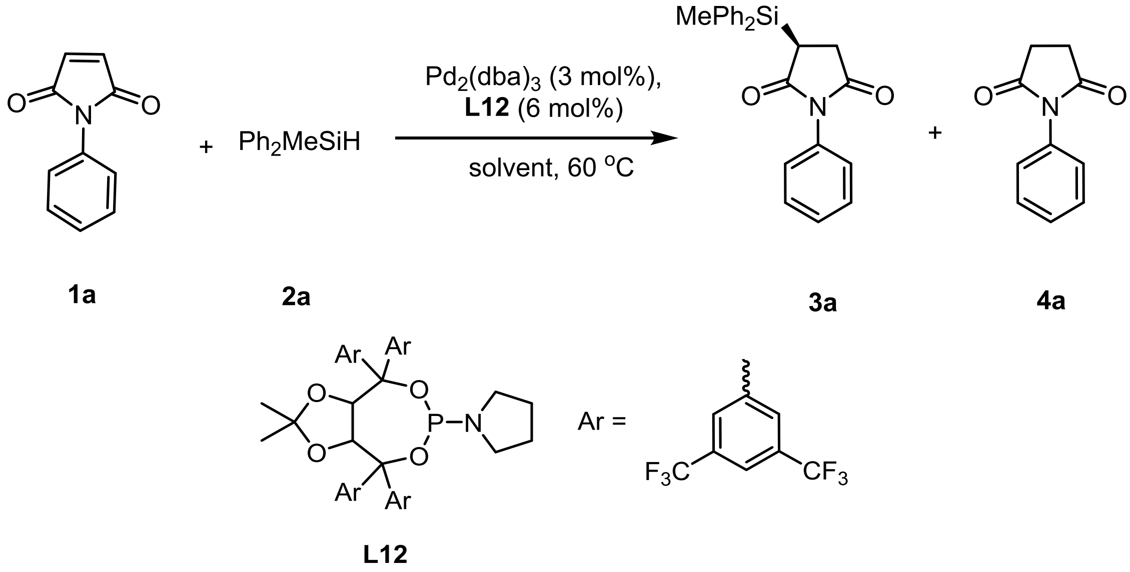

Entry	Solvent	Yield of **3a** (%)[b]	Yeld of **4a** (%)[b]	*ee* of **3a** (%)[c]
1	dioxane	67	32	92
2	toluene	93	7	94
3	Et_2O	33	67	92
4	DCE	85	15	94
5	THF	4	96	70
6	DCM	96	4	93
7	EtOAc	26	74	81
8	hexane	43	57	94

[a]All the reactions were run on a 0.1 mmol scale in 1.0 mL solvents for 18 h. [b]Determined by ^{1}H NMR using dibromomethane as an internal standard. [c]Determined by HPLC.

Supplementary Table 4. Screen of temp for hydrosilylation reaction.[a]

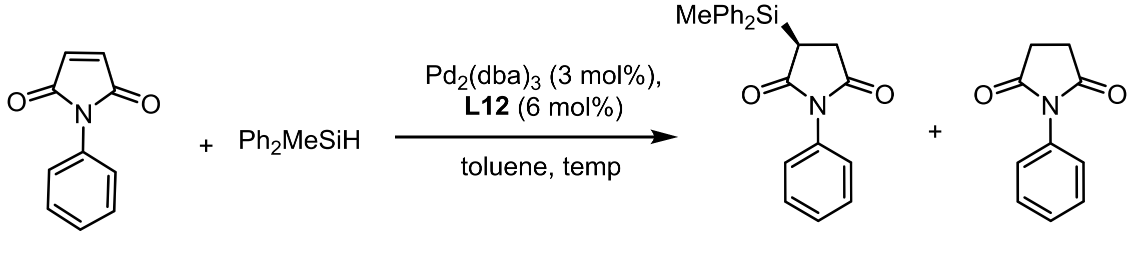

1a 2a 3a 4a

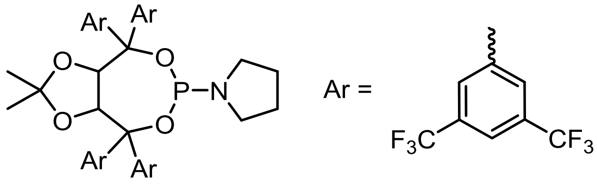

L12

Entry	Temp (°C)	Yield of **3a** (%)^b	Yield of **4a** (%)^b	*ee* of **3a** (%)^c
1	40	57	22	97
2	50	93	7	96
3	60	93	7	94
4	70	96	4	91

[a]All the reactions were run on a 0.1 mmol scale in 1.0 mL solvents for 18 h. [b]Determined by ^{1}H NMR using dibromomethane as an internal standard. [c]Determined by HPLC.

Supplementary Table 5. The effect of P-ligands L14-L18 on the optimal condition hydrosilylation reaction. [a]

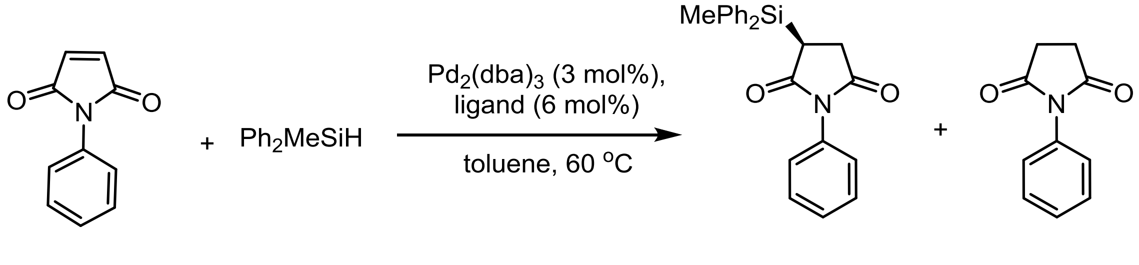

1a **2a** **3a** **4a**

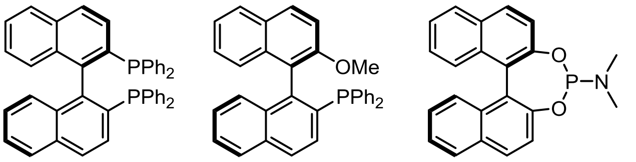

L14 **L15** **L16**

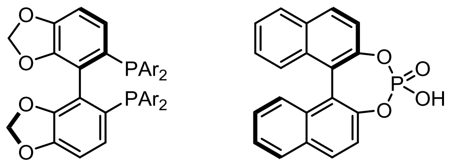

L17 (*R*)-DTBM-SEGPHOS **L18**

Ar = 3,5-(*t*-Bu)$_2$-4-MeO-C_6H_2

Entry	Ligand	Silane	Yield of **3a** (%)[b]	Yield of **4a** (%)[b]
1	**L14**	Ph_2MeSiH	nd	14
2	**L15**	Ph_2MeSiH	nd	100
3	**L16**	Ph_2MeSiH	nd	28
4	**L17**	Ph_2MeSiH	nd	29
5	**L18**	Ph_2MeSiH	nd	100
6	**L12**	Ph_2SiH_2	nd	100
7	**L12**	$PhSiH_3$	nd	100

[a]All the reactions were run on a 0.1 mmol scale in 1.0 mL solvents for 18 h. [b]Determined by ^{1}H NMR using dibromomethane as an internal standard.

Supplementary Table 6. Optimization of axial chiral hydrosilylation reaction.[a]

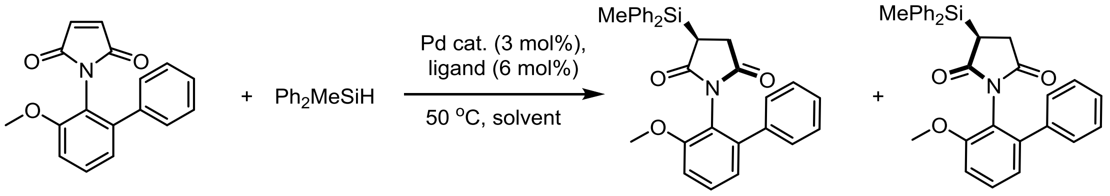

4a 2a 5a 5a'

Entry	Pd cat.	Ligand	Solvent	Yield of **5a** (%)[b]	*ee* of **5a** (%)[c]	*dr* of **5a** (%)[d]
1	$Pd_2(dba)_3$	**L19**	toluene	73	80	80:20
2	$Pd_2(dba)_3$	**L10**	toluene	62	95	91:9
3	$Pd_2(dba)_3$	**L12**	toluene	82	94	95:5
4	$Pd_2(dba)_3$	**L12**	DCE	81	93	95:5
5	$Pd_2(dba)_3.CHCl_3$	**L12**	toluene	91	90	97:3

[a]All the reactions were run on a 0.3 mmol scale in 3.0 mL solvents for 18 h. [b]Yield of isolated product. [c]Determined by HPLC. [d]Determined by isolated yield, the structure **5a** and **5a'** is determined by ^{1}H NMR.

Supplementary Discussion

Controlled experiment for the proposed mechanism

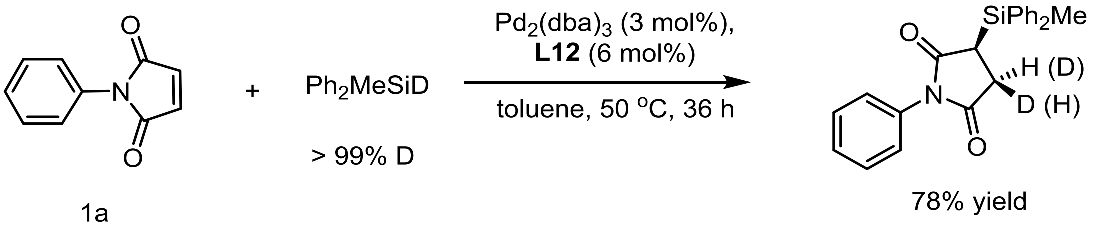

1a

A vial was charged with N-phenyl maleimide (52.0 mg, 0.3 mmol), $Pd_2(dba)_3$ (8.2 mg, 3.0 mol%), (*R,R*)-**L12** (20.1 mg, 6 mol%) and evacuated under high vacuum and backfilled with N_2. Toluene (3 mL) was next added. The mixture was stirred at 25 °C for 10 minutes, then the Ph_2MeSiD (0.6 mmol) was added to the reaction. The mixture was stirred at 50 ℃ in a preheated oil. Upon reaction completion, the mixture was filtered over a plug of silica gel (washed with 50 ml EtOAc), and the filtrate was concentrated. The crude was purified by column chromatography to give the corresponding product and was analyzed with ^1H NMR to determine the corresponding product ratio and recovered.

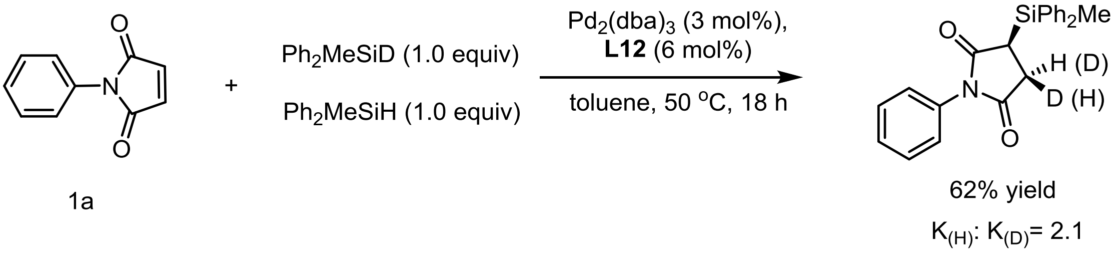

1a

A vial was charged with N-phenyl maleimide (52.0 mg, 0.3 mmol), $Pd_2(dba)_3$ (8.2 mg, 3.0 mol%), (*R,R*)-**L12** (20.1 mg, 6 mol%) and evacuated under high vacuum and backfilled with N_2. Toluene (3 mL) was next added. The mixture was stirred at 25 °C for 10 minutes, then the Ph_2MeSiD (0.3 mmol) and Ph_2MeSiH (0.3 mmol) was added to the reaction. The mixture was stirred at 50 ℃ in a preheated oil. Upon reaction completion, the mixture was filtered over a plug of silica gel (washed with 50 ml

EtOAc), and the filtrate was concentrated. The crude was purified by column chromatography to give the corresponding product and was analyzed with ^{1}H NMR to determine the corresponding product ratio (**Supplementary Figure 3**) and recovered.

Computational studies by DFT

Calculated energy parameters vibration

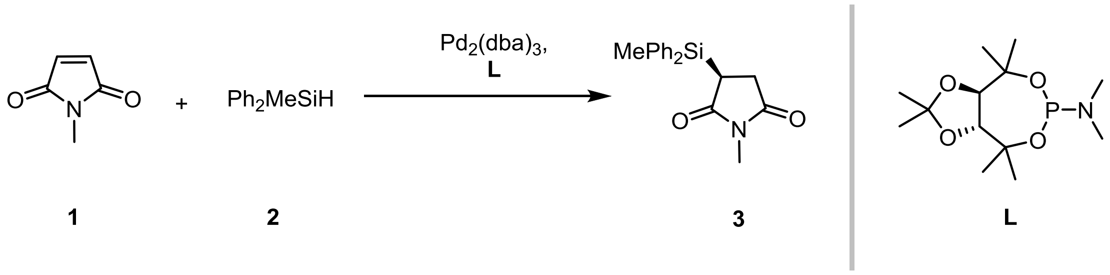

Supplementary Table 7. Reaction parameters for N-Methylmaleimide **1** and diphenylmethylsilane **2** catalyzed by the monoligated Pd^0 catalyst [Pd^0L] calculated at M06L/LANL2DZ(Pd), 6-311G(d,p) (C, H, O, N, P, Si) level of theory at 298.15 K in gas phase. [a,b]

System[c]	ΔE[d]	ΔH[d]	ΔS[e]	ΔG[d]
**B+D**	0	0	0	0
**C**	-22.9	-23.0	-56.3	-6.2
**TSCF**	-3.3	-3.5	-57.4	13.6
**F**	-22.7	-22.8	-55.4	-6.2
**TSFG**	-6.9	-7.3	-57.3	9.7
**G**	-26.6	-26.6	-51.8	-11.2
**I**	-22.4	-22.1	-48.5	-7.6
**TSIJ**	34.6	34.5	-47.3	48.6
**J**	6.6	6.6	-51.0	21.8
**K**	-27.9	-27.6	-47.7	-13.3

[a] Basis set (BS) refers to the standard 6-311G(d,p) basis for C, H, O, N, P and Si atoms and LANL2DZ valence basis set in combination with the corresponding effective core potential for Pd and I. [b] Relative activation energy and reaction parameters were calculated based on those of free **B + D**. [c] Parameters for all the transition state (TSs) should read as those with double dagger like $\Delta E^{\neq}$, $\Delta H^{\neq}$, $\Delta S^{\neq}$, and $\Delta G^{\neq}$. [d] In kcal/mol. [e] In kcal/(mol K).

Computational Details

All calculations were performed with the Gaussian09 suites of programs[13]. The geometries of all stationary points and transition states were optimized by using the M06L functional in conjunction with the standard 6-311G(d,p) basis for all atoms (C,

H, O, N, P, Si) except for palladium, which were described by the LANL2DZ valence basis set in combination with the corresponding effective core potential. We label this basis set combination as BS. Geometries were fully optimized, normally without symmetry constraints. Frequencies calculations were carried out at the same level of theory to verify the stationary points as minima or saddle points. The connectivity of the stationary points to transition state was verified by intrinsic reaction coordinate (IRC) runs or the vibrational mode of the imaginary frequencies. Solvent effects were included with fully optimization calculations at the same level of theory by using the integral equation formalism variant of the polarizable continuum model (IEFPCM) in toluene with those geometries optimized in gas phase as initial structures. The frequencies calculated at M06L/BS level were used to obtain the reported energies as zero-point energy-corrected energies, enthalpies, and free energies.

Reaction mechanism of silyl atropisomer.

As shown in Supplementary Figure 9, on the basis of experimental results and the absolute configuration of the single crystal **5f** as P,S, the origin of the remote control of axial chirality is also consistent with above reaction mechanism. In this case, because of the steric hindrance, the smaller group (such as methoxy or methyl group) is more likely to be coated in the cavity of the TADDOL-derived ligand with Pd complex, whereas the larger group (such as aromatic rings) is exposed to the outside of palladium catalyst. Therefore, the hydrosilylation occurs easily via attacking the carbon-carbon double bond on the side of the methoxy group.

Theoretical calculations on the rotation barrier

To better understand the effect of substituent effects in 2,6-position of the N-arylmaleimides on racemization barrier, density functional theory (DFT) studies have been utilized using M06L method and 6-311G (d,p) basis set with Gaussian 09 program. All geometries were optimized by relaxed scan method with M06L/6-311G (d). Harmonic vibrational frequency calculations at 298.15K showed that the

stationary points located to be either minima (with zero imaginary frequencies) or transition states (with one imaginary frequency).

As shown in Supplementary Figure 10, there are two transition states account for the interconversion of the two enantiomers according to the rotation direction of the chiral axis.[14-18] In this study, both TS-Rot-syn and TS-Rot-anti have been considered.

As listed in Supplementary Figure 10, the rotation barriers increased sharply with the size of the 2,6-substituents. In detail, the rotation barrier of **3ff** was very low, which explained the phenomenon with low diastereoselectivity for the catalytic synthesis of such compound under the same reaction conditions. On the contrary, the rotation barrier of **5a** was quite high (44.2 kcal/mol), which supported our hypothesis that the large size of aryl group would generate a strong steric congestion around the C-N axis of maleimides.[19] The increasing of the rotation barriers ensures the conformational stability of the generated axial chirality in the remote control process. In addition, the rotation barrier of **3hh** was slightly lower than **5a**, which explained the observed moderate diastereoselectivity of **3hh**. The structures of **3ff**, **3hh**-TS-Rot-anti and **5a**-TS-Rot-syn are shown in Supplementary Figure 10 and Figure 11.

Supplementary Figures

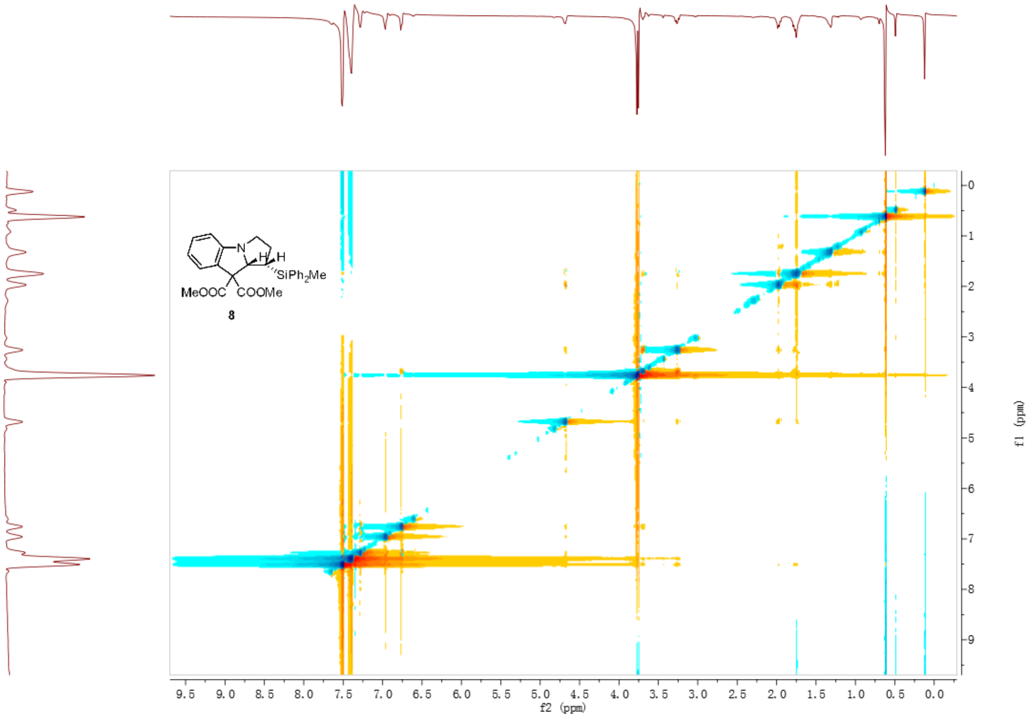

Supplementary Figure 1. NOESY spectrum of 8. The determination of the structure of product 8.

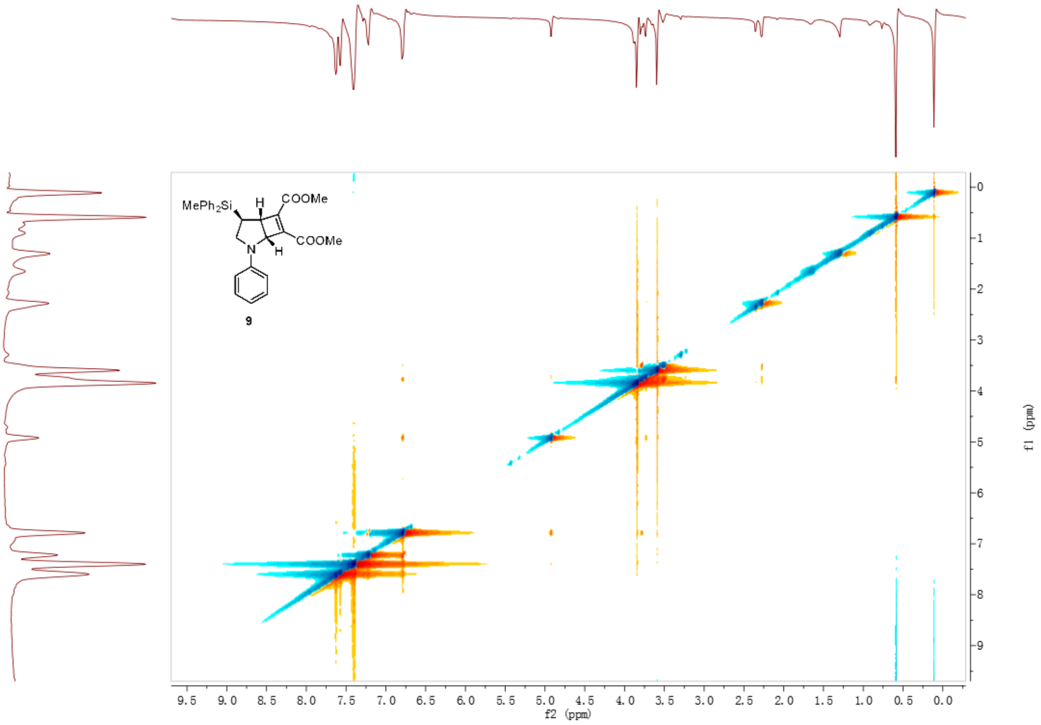

Supplementary Figure 2. NOESY spectrum of 9. The determination of the structure of product 9.

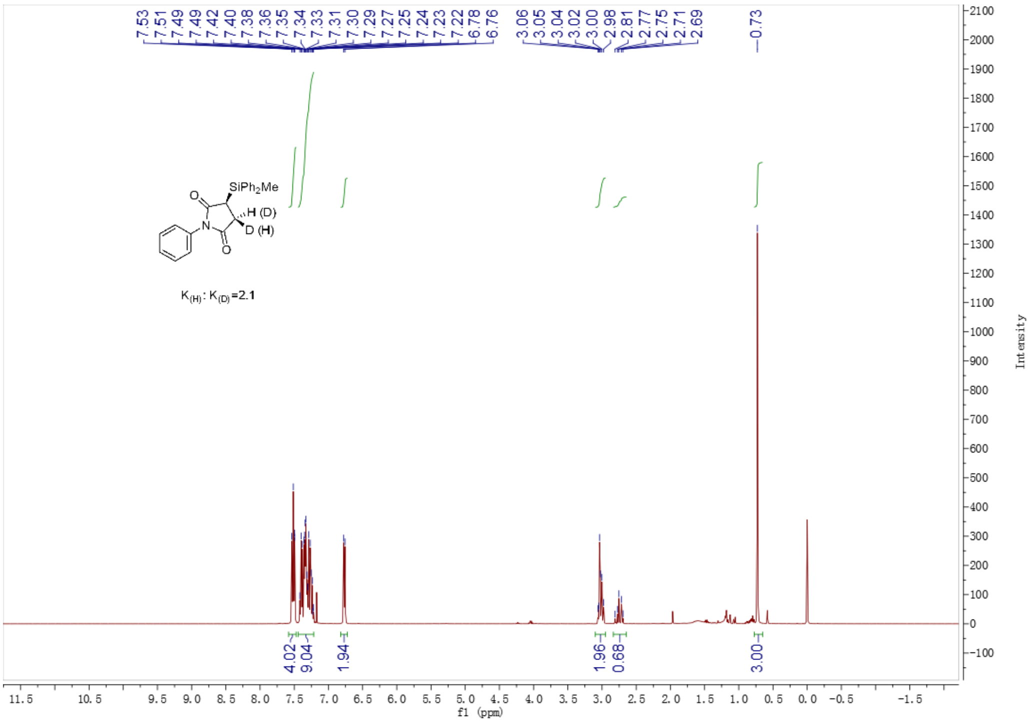

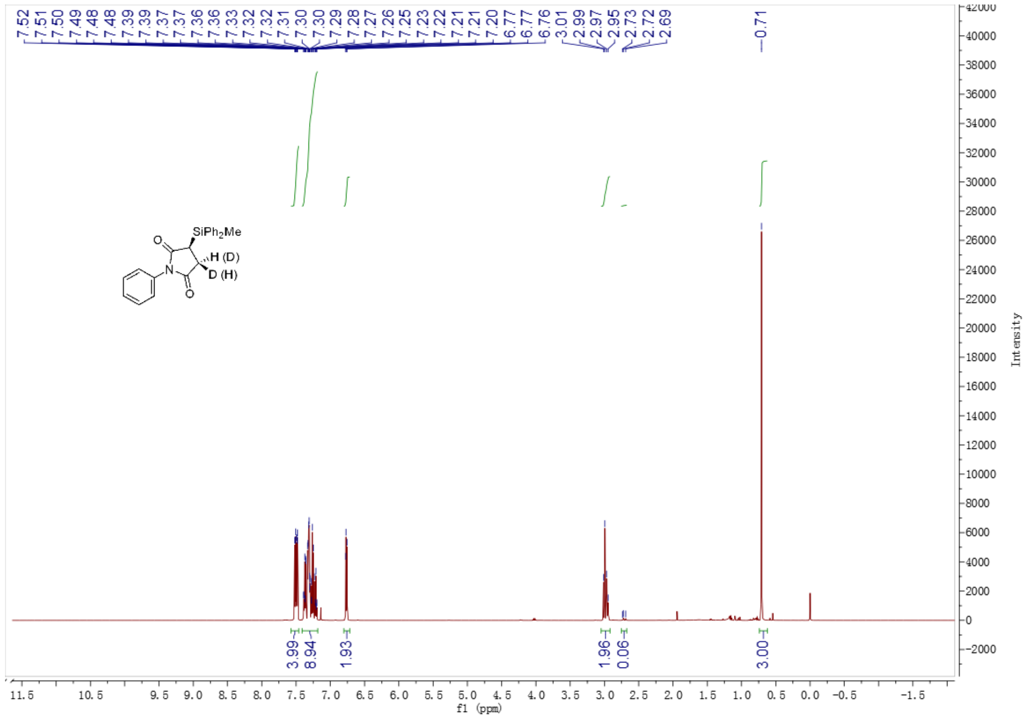

Supplementary Figure 3. Kinetic Study for the KIE. The determination of the Si-H action as a key step in this reaction.

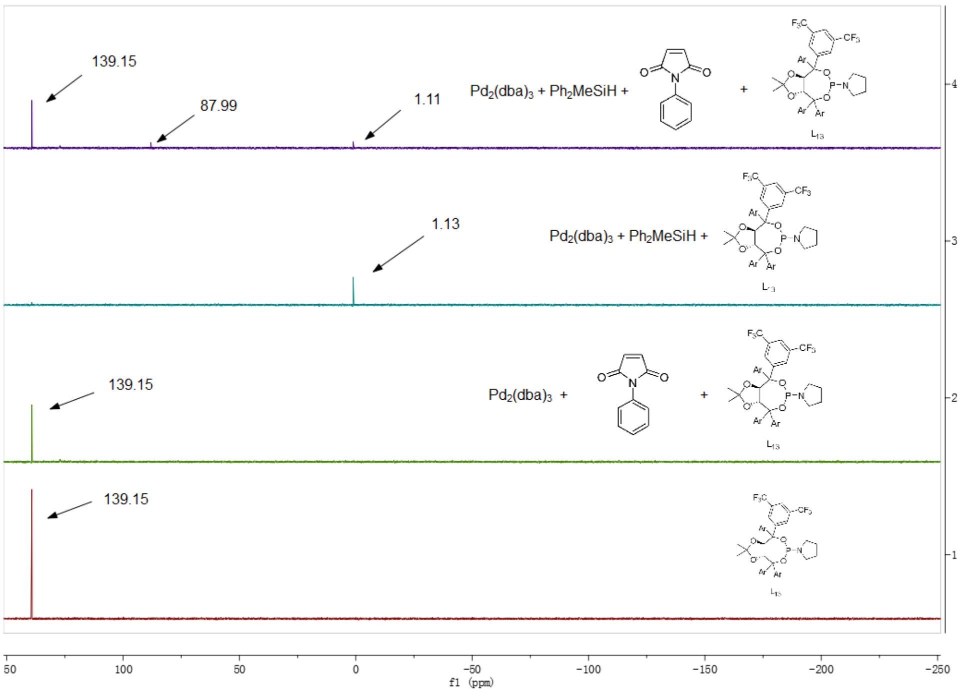

Supplementary Figure 4. Comparison of ^{31}P NMR of ligand and palladium complex in the presence of additive. The determination of the substrate effect on the palladium catalyst system.

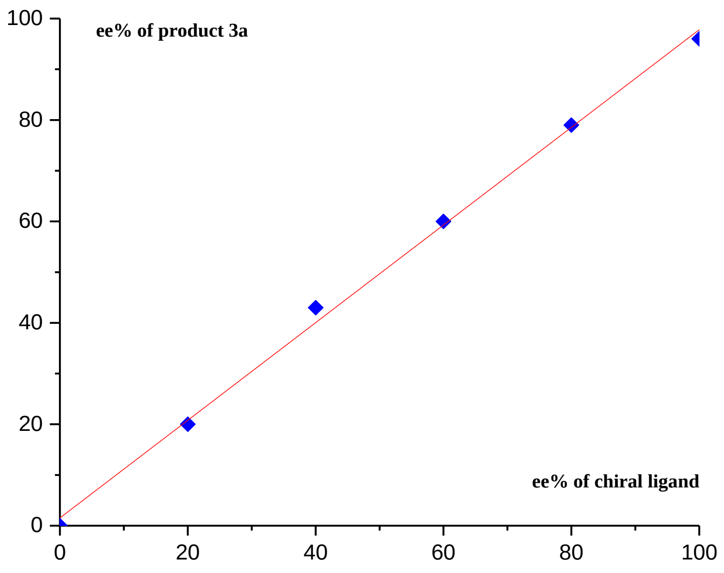

Supplementary Figure 5. The study of possible NLE in the Pd-catalyzed hydrosilylation of 1a. The determination of palladium catalyst as PdL complex because of its linear effect. Source data are provided as a Source Data file.

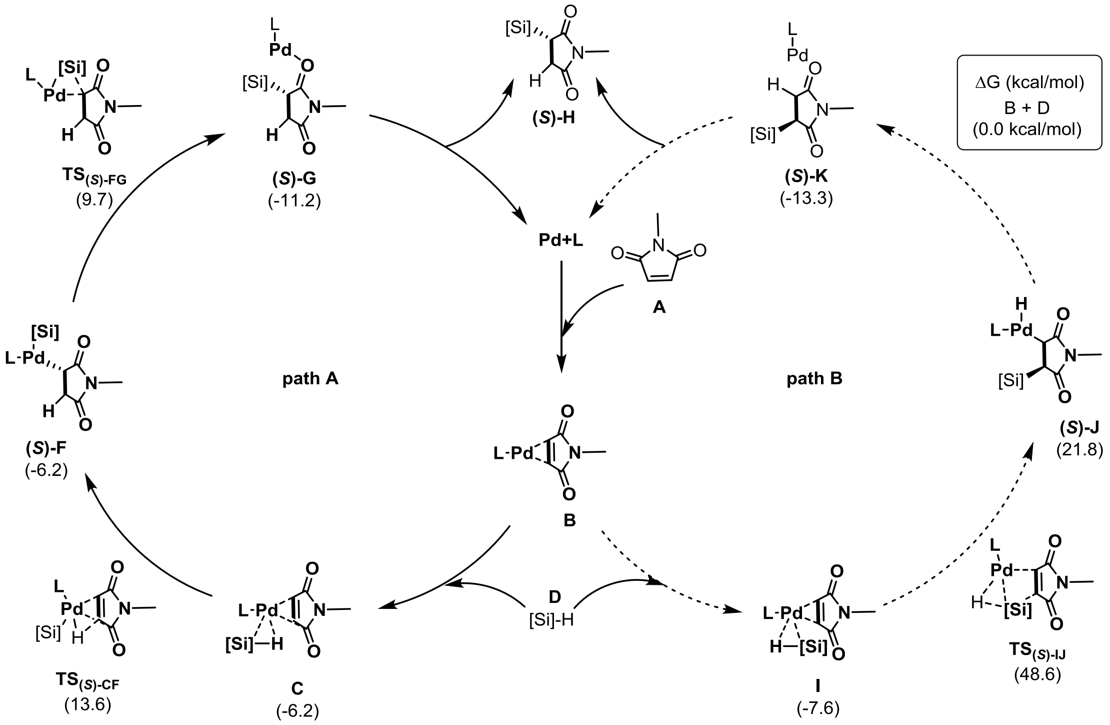

Supplementary Figure 6. Proposed catalytic cycles for the model reaction system.

Source data are provided as a Source Data file.

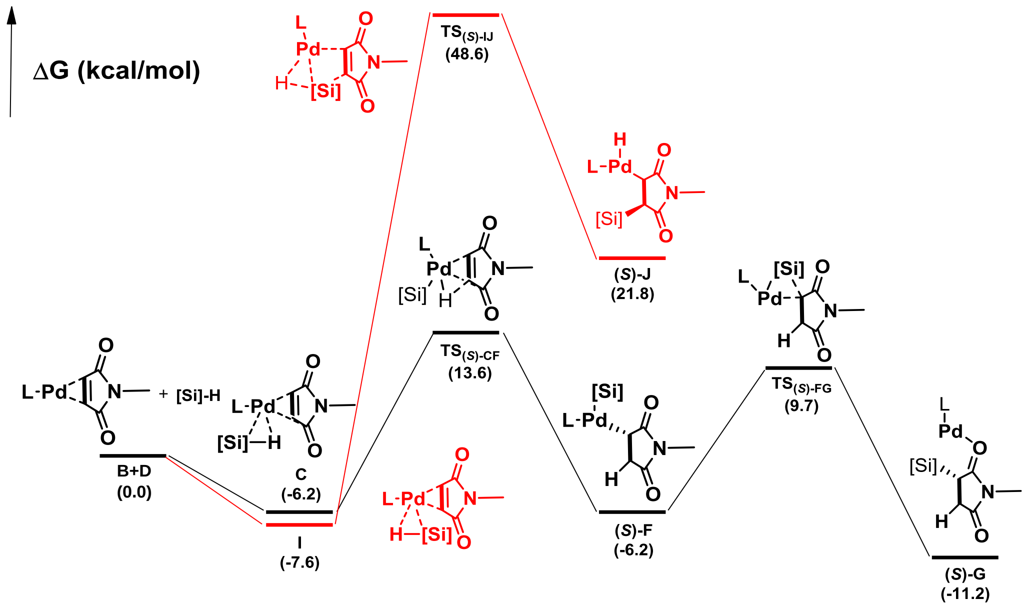

Supplementary Figure 7. The free-energy profile [kcal/mol] for Pd-catalyzed of reaction. The free energy of monoligated palladium complex (B + D) is set to be relative zero reference. Source data are provided as a Source Data file.

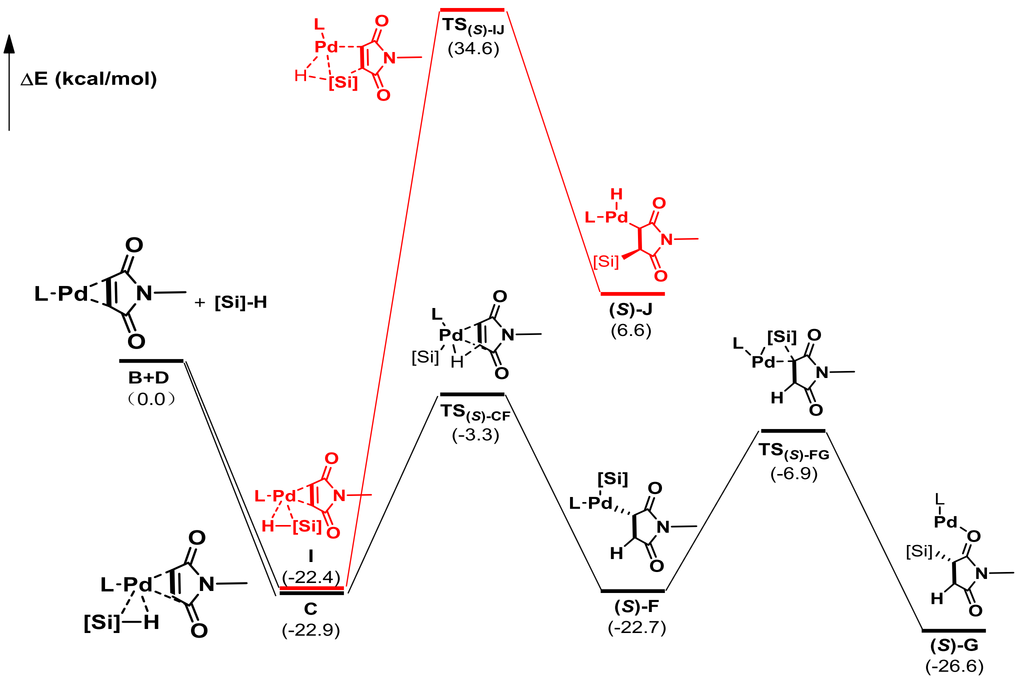

Supplementary Figure 8. The free-energy profile [kcal/mol] for Pd-catalyzed of reaction. The free energy of monoligated palladium complex (B + D) is set to be relative zero reference. Source data are provided as a Source Data file.

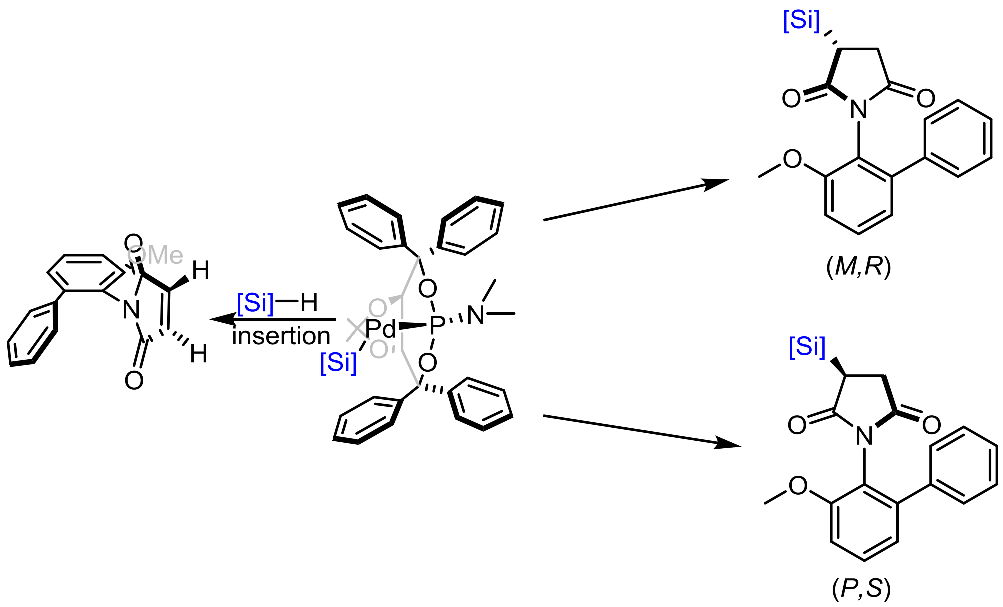

Supplementary Figure 9. Reaction mechanism of silyl atropisomer.

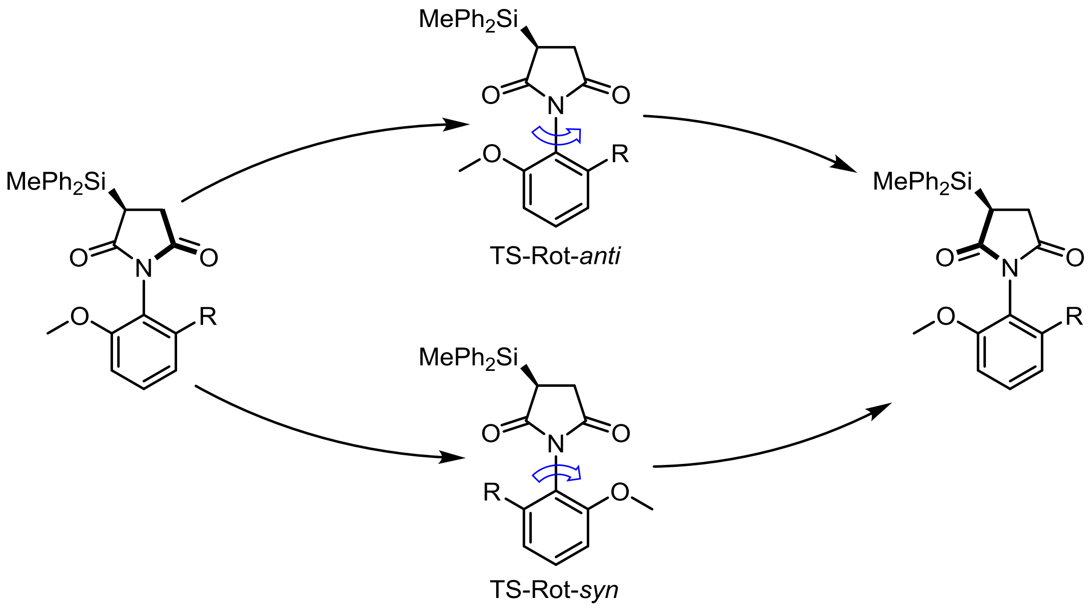

Supplementary Figure 10. The pathways for rotation barrier. Two transition states account for the racemization.

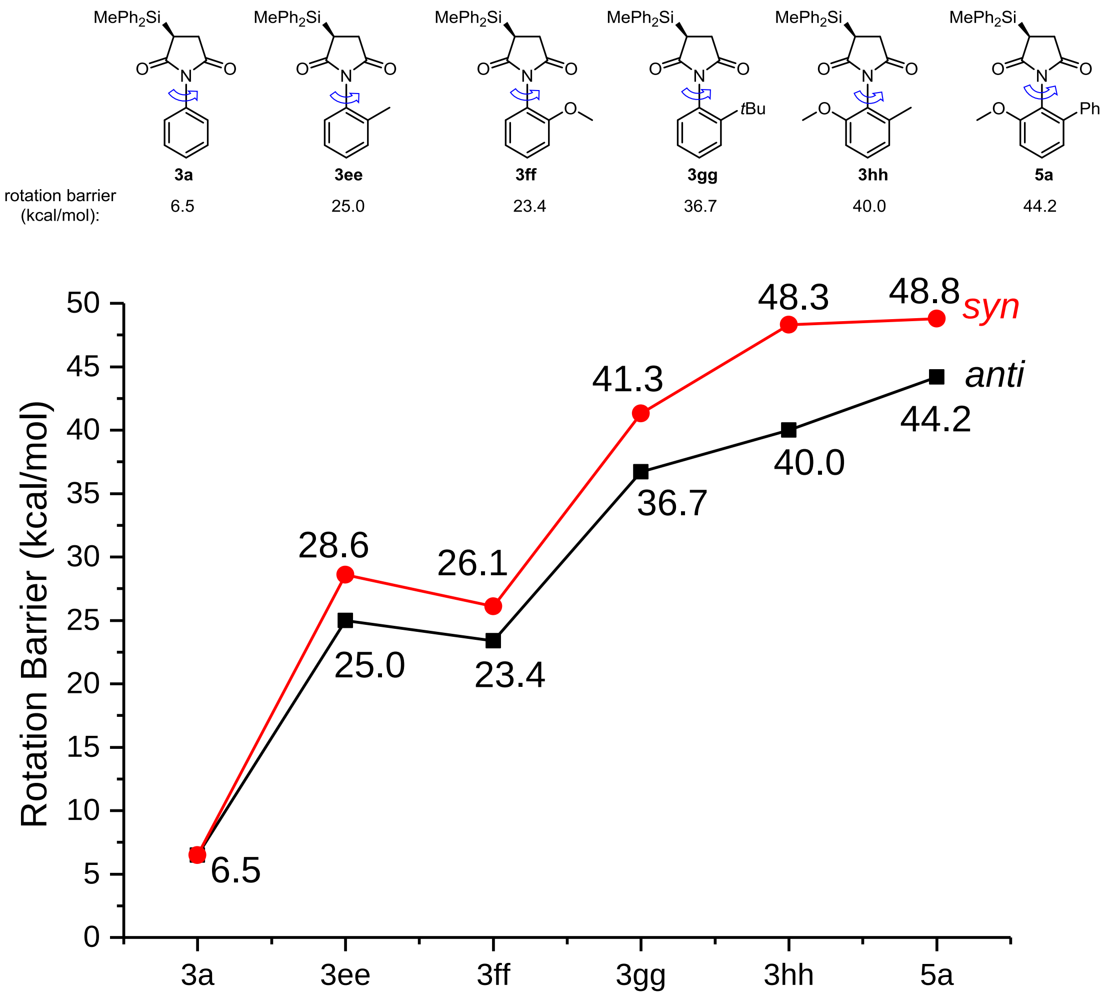

Supplementary Figure 11. Theoretical data on the rotation barrier. The relationship between the energy barriers of racemization and the bulkiness of substituents in *ortho*-position of the maleimides. Source data are provided as a Source Data file.

The confirmation of the absolute configuration of chiral product

X-ray structures of 3l (CCDC 1967248)

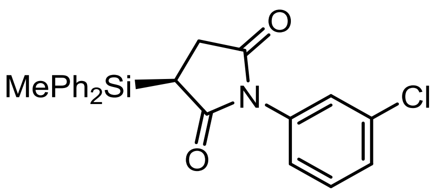

3l

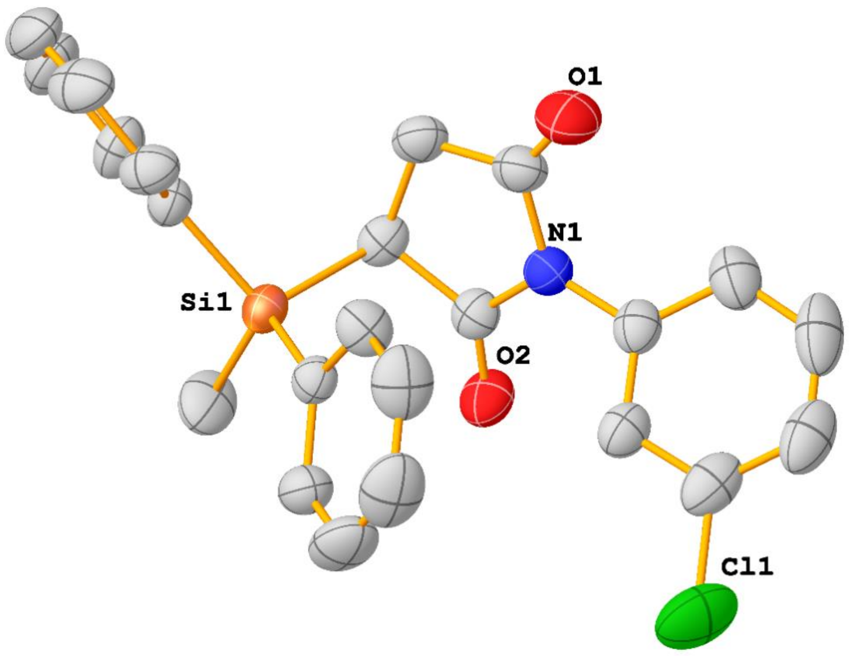

Supplementary Figure 12. X-ray structures of chiral product 3l. Crystal data and structure refinement for the product **3l** can be obtained free of charge from The Cambridge Crystallographic Data Centre via www.ccdc.cam.ac.uk/data_request/cif.

X-ray structures of 5f (CCDC 1994220)

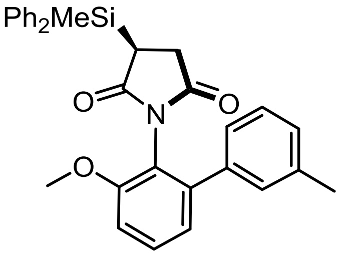

(*P*,*S*)-5f

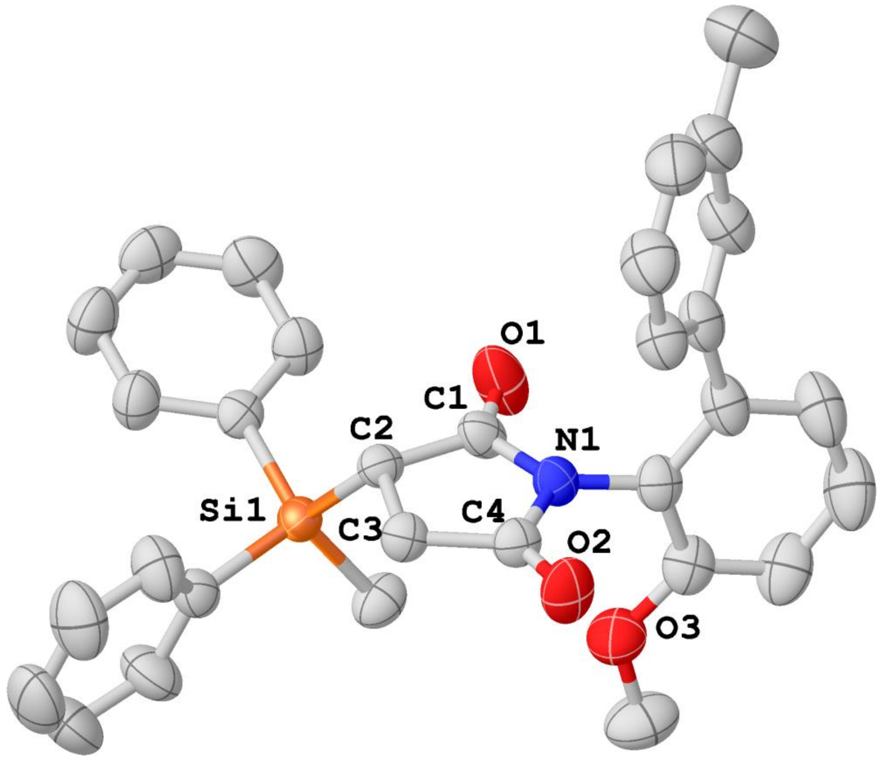

Supplementary Figure 13. X-ray structures of chiral product 5f. Crystal data and structure refinement for the product **5f** can be obtained free of charge from The Cambridge Crystallographic Data Centre via www.ccdc.cam.ac.uk/data_request/cif.**.**

NMR spectra

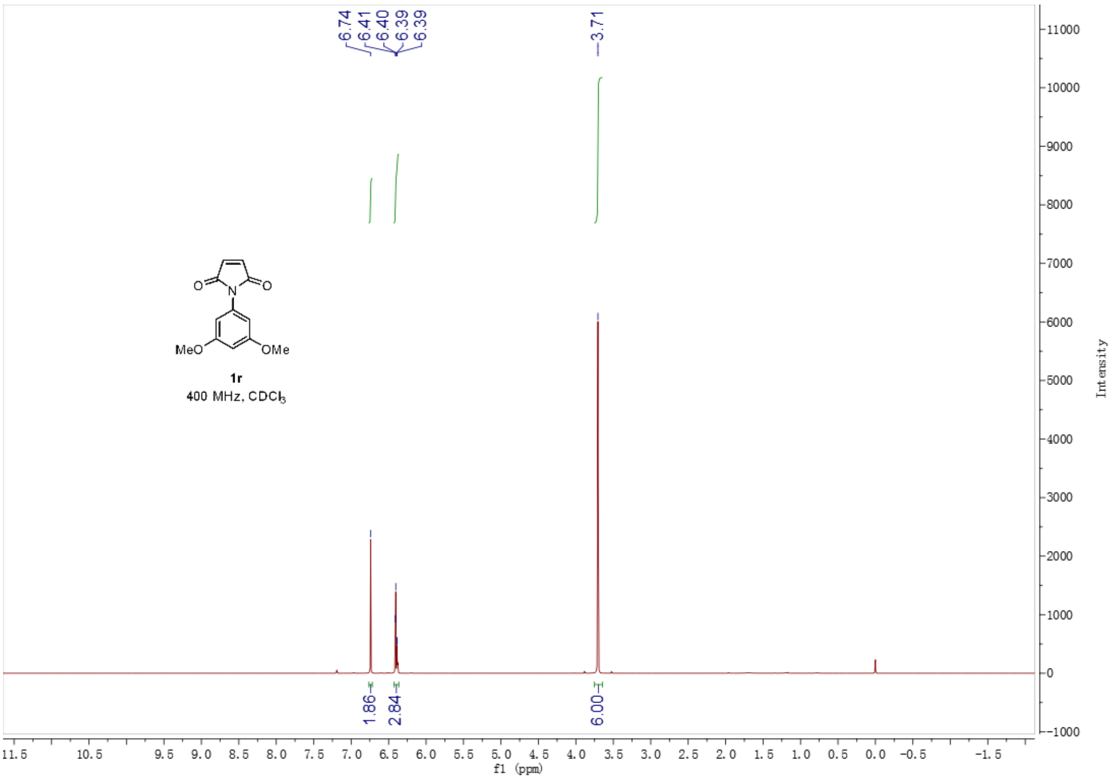

Supplementary Figure 14. ^{1}H NMR spectrum for **1r**

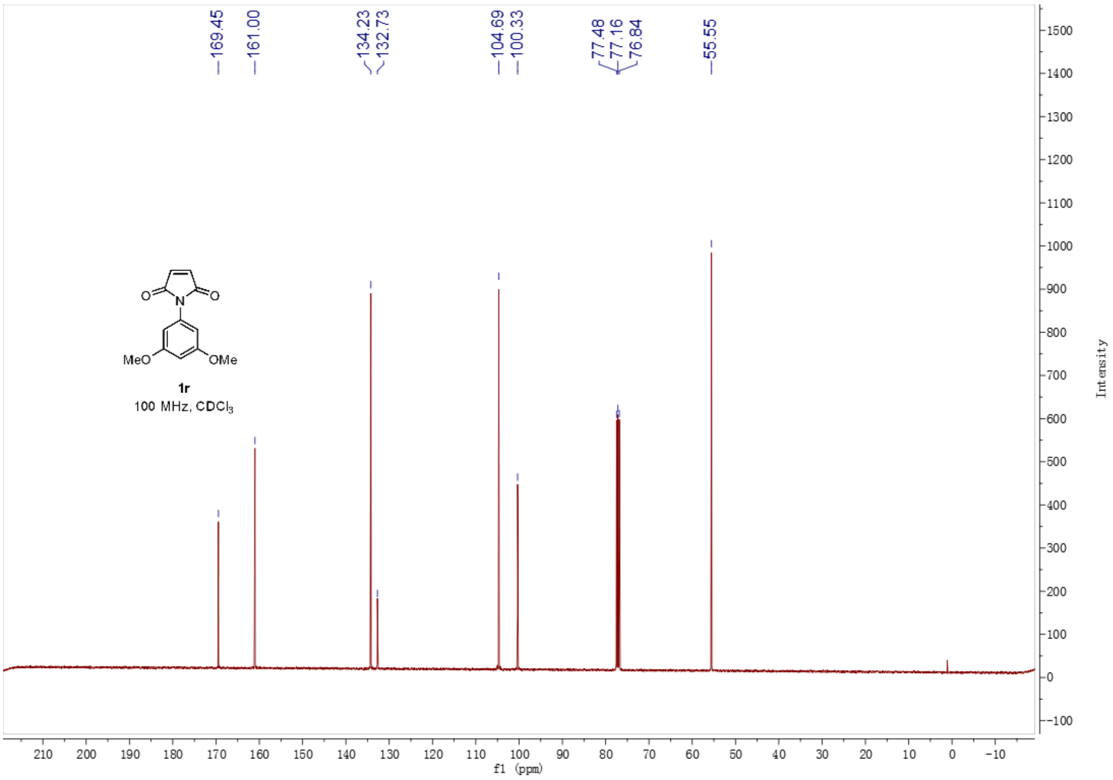

Supplementary Figure 15. ^{13}C NMR spectrum for **1r**

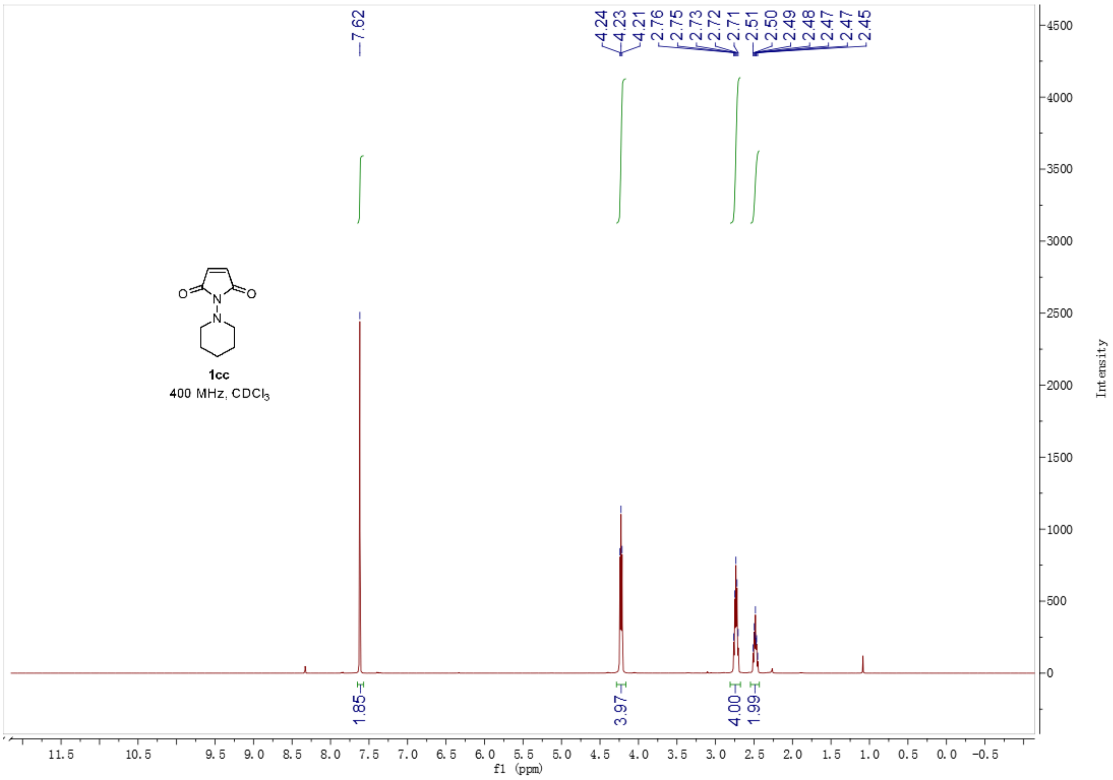

Supplementary Figure 16. ^{1}H NMR spectrum for **1cc**

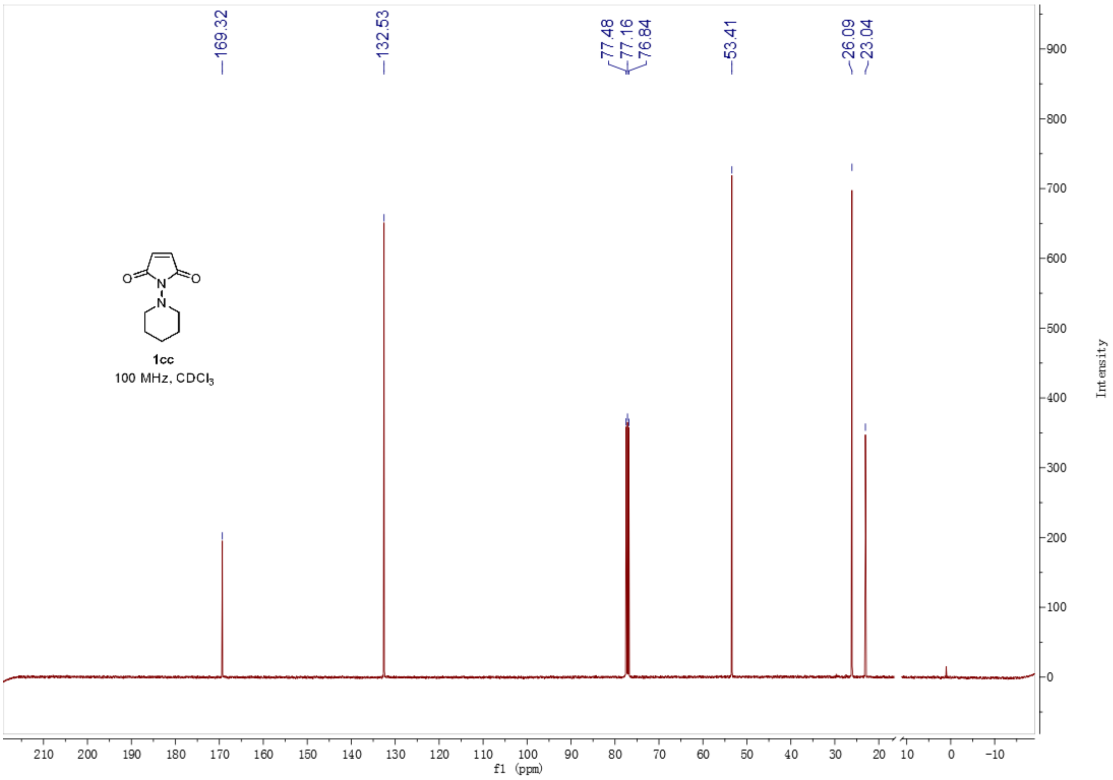

Supplementary Figure 17. ^{13}C NMR spectrum for **1cc**

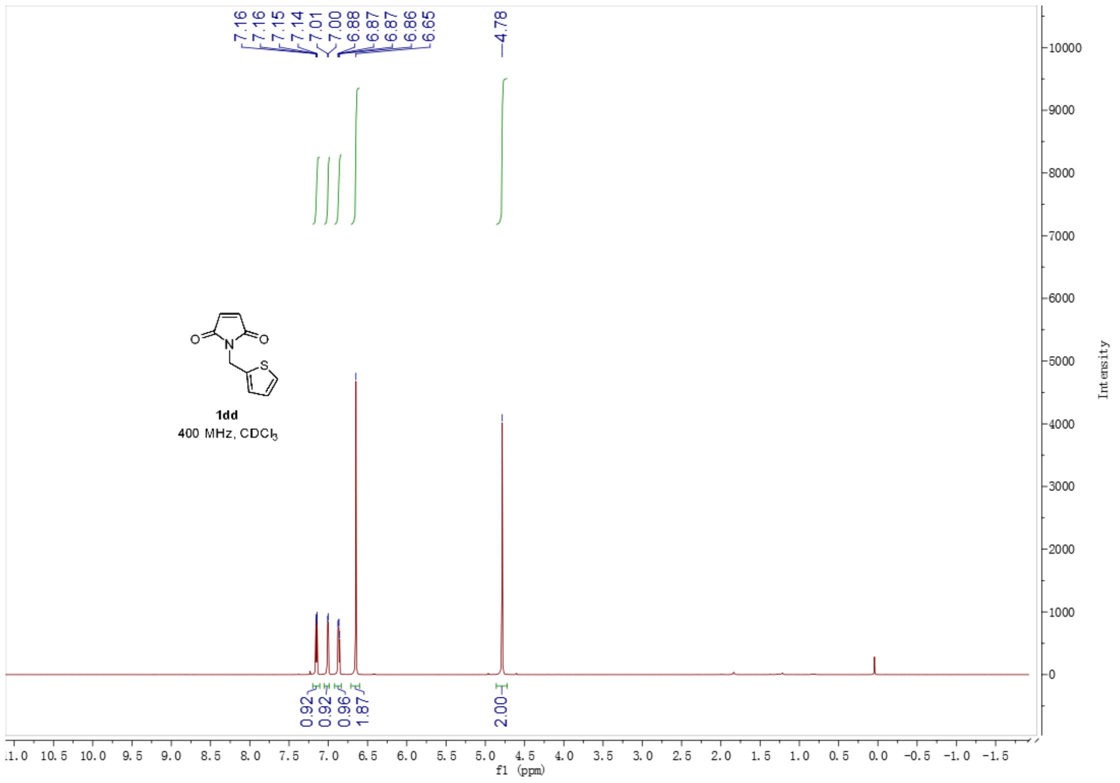

Supplementary Figure 18. ^{1}H NMR spectrum for **1dd**

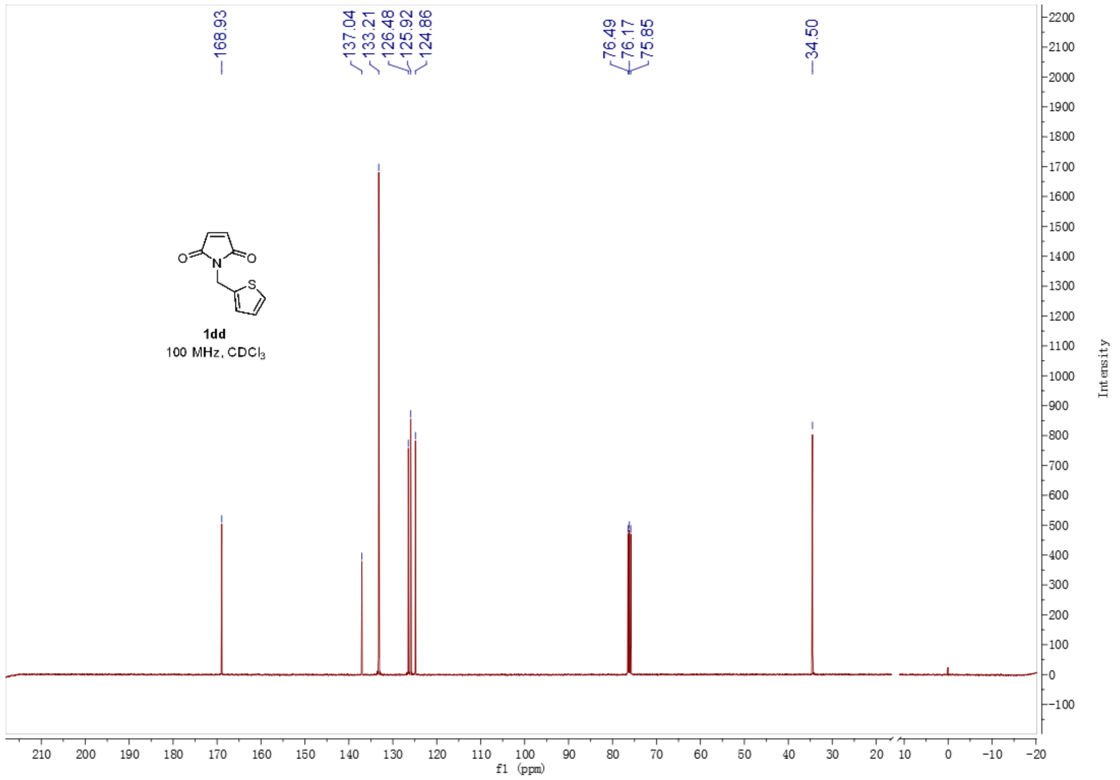

Supplementary Figure 19. ^{13}C NMR spectrum for **1dd**

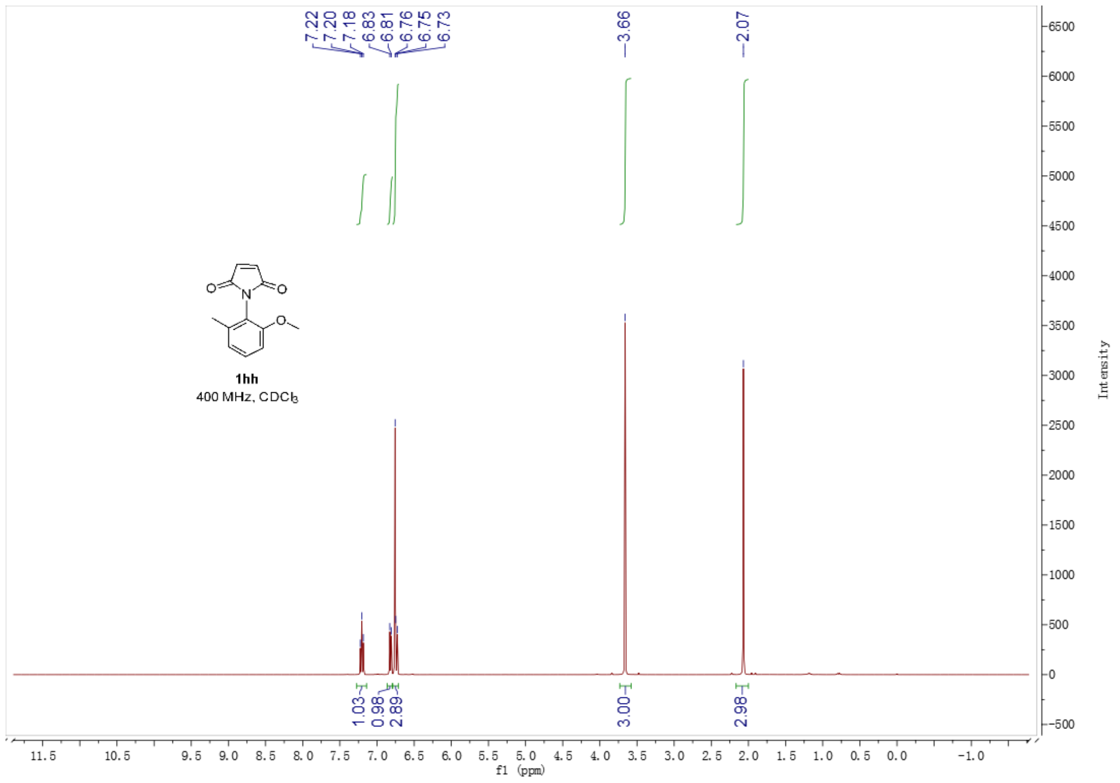

Supplementary Figure 20. ^{1}H NMR spectrum for **1hh**

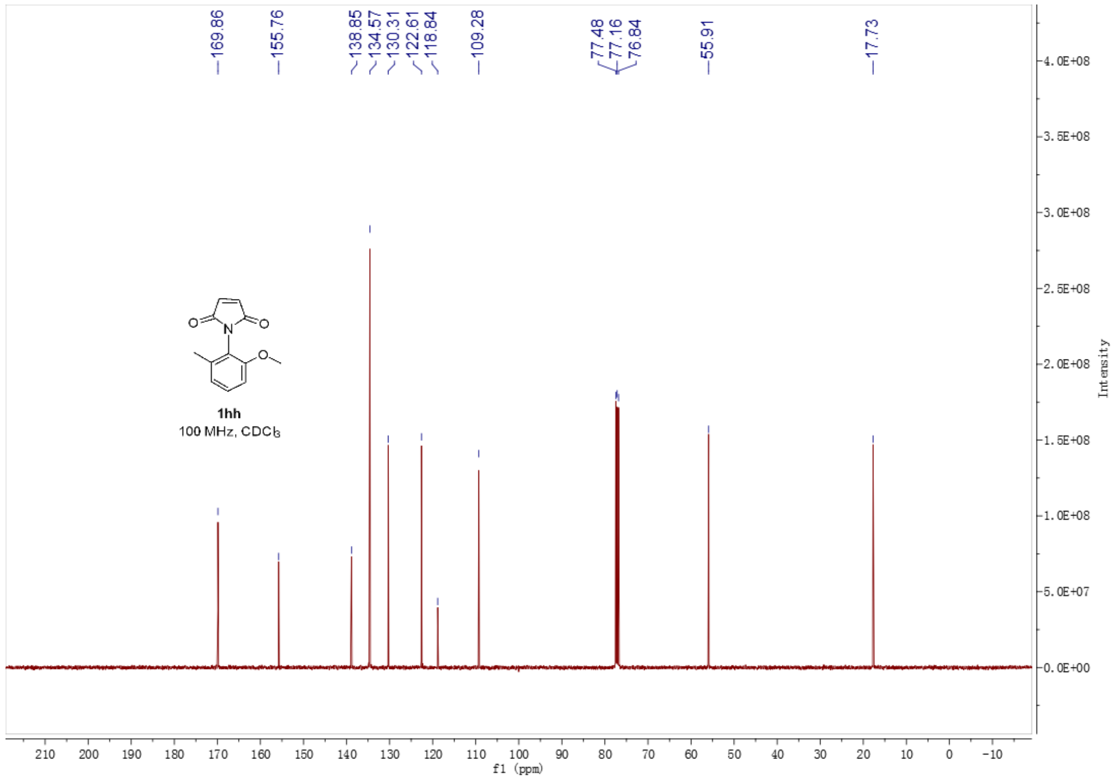

Supplementary Figure 21. ^{13}C NMR spectrum for **1hh**

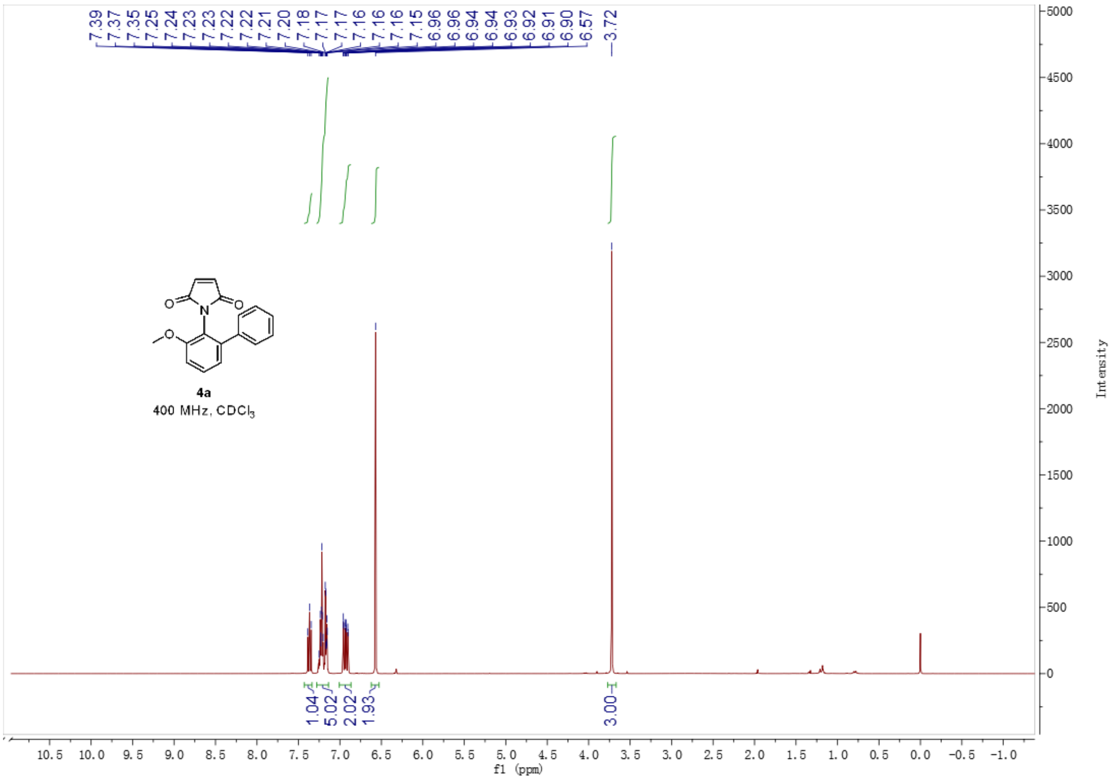

Supplementary Figure 22. ^{1}H NMR spectrum for **4a**

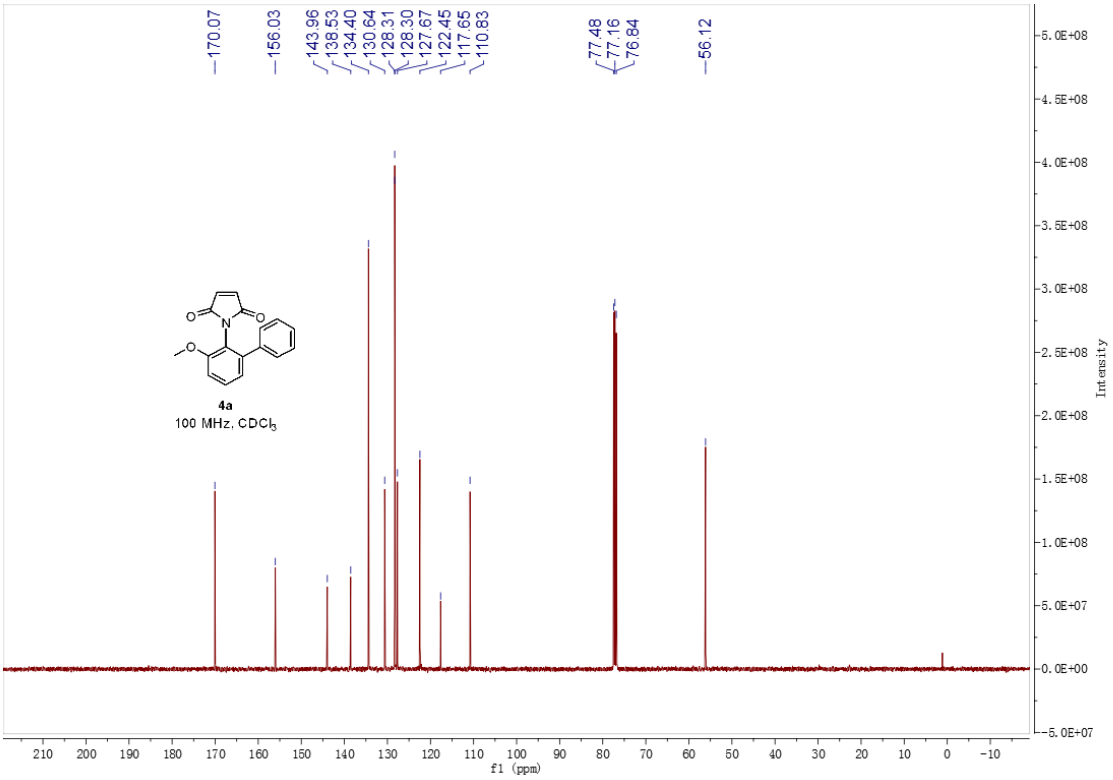

Supplementary Figure 23. ^{13}C NMR spectrum for **4a**

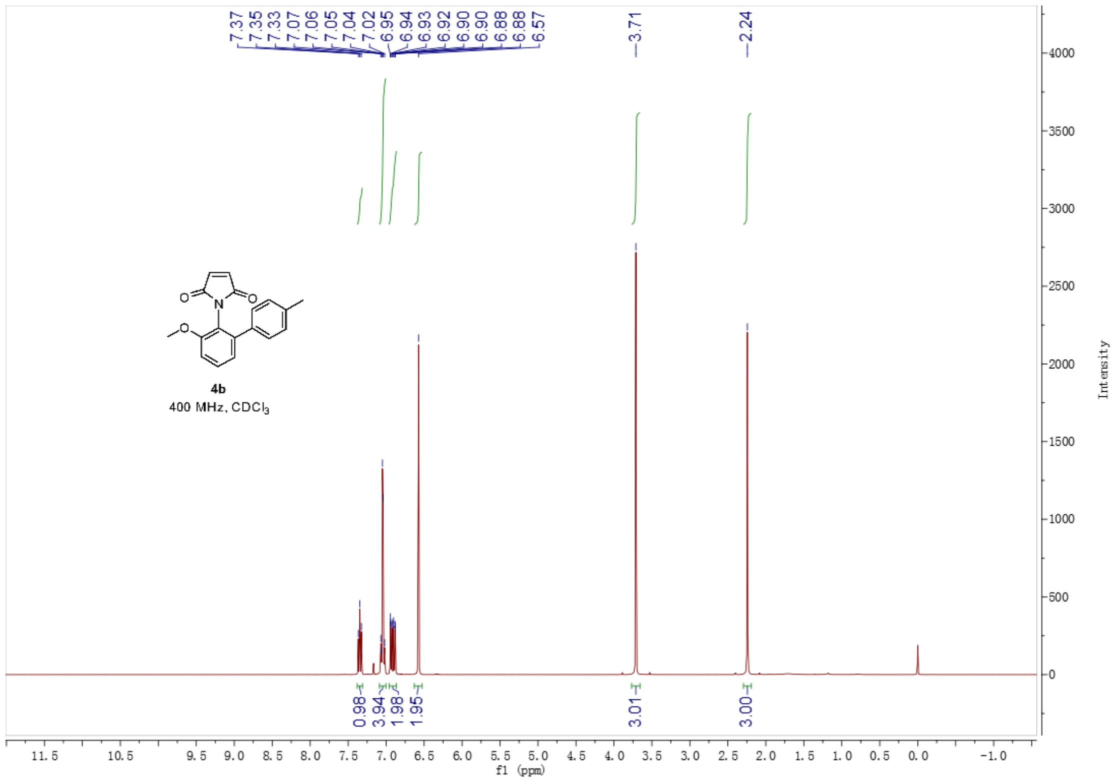

Supplementary Figure 24. ^{1}H NMR spectrum for **4b**

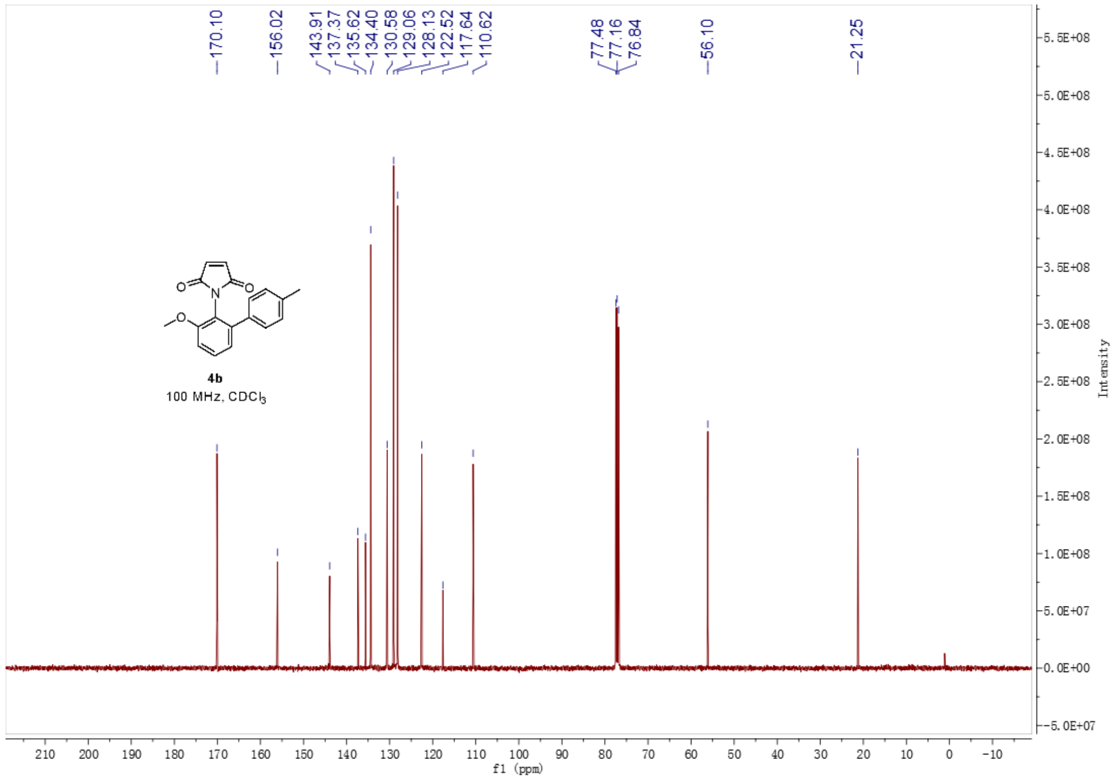

Supplementary Figure 25. ^{13}C NMR spectrum for **4b**

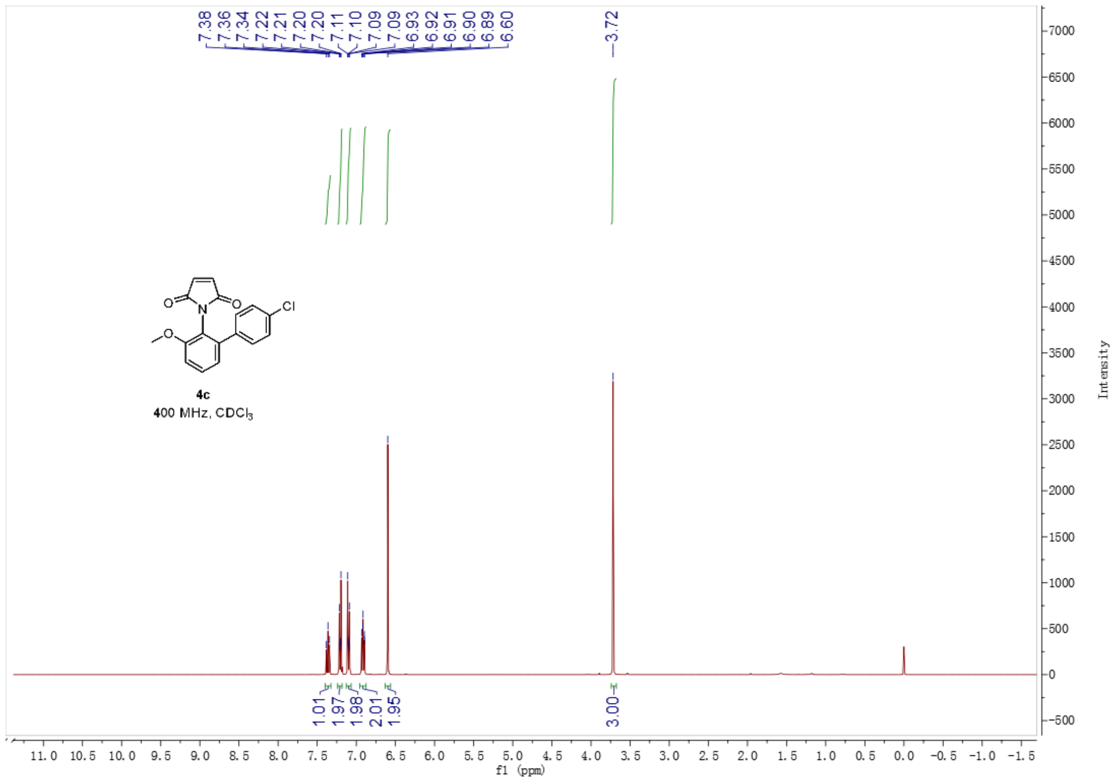

Supplementary Figure 26. ^{1}H NMR spectrum for **4c**

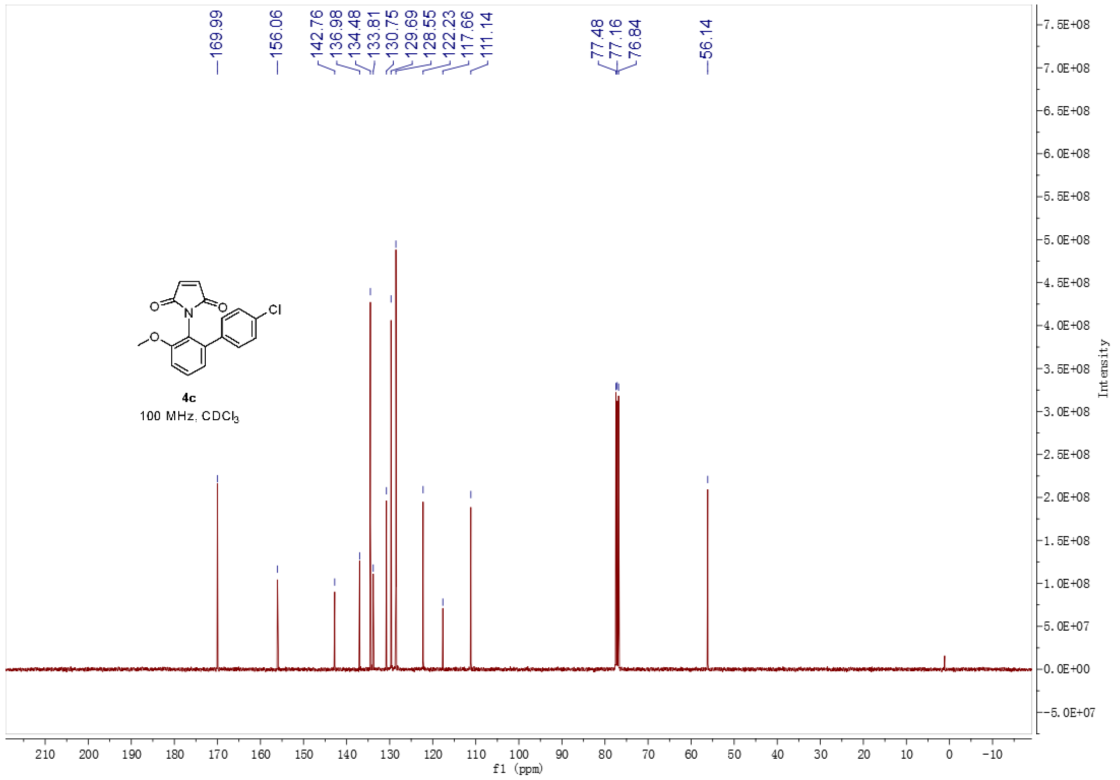

Supplementary Figure 27. ^{13}C NMR spectrum for **4c**

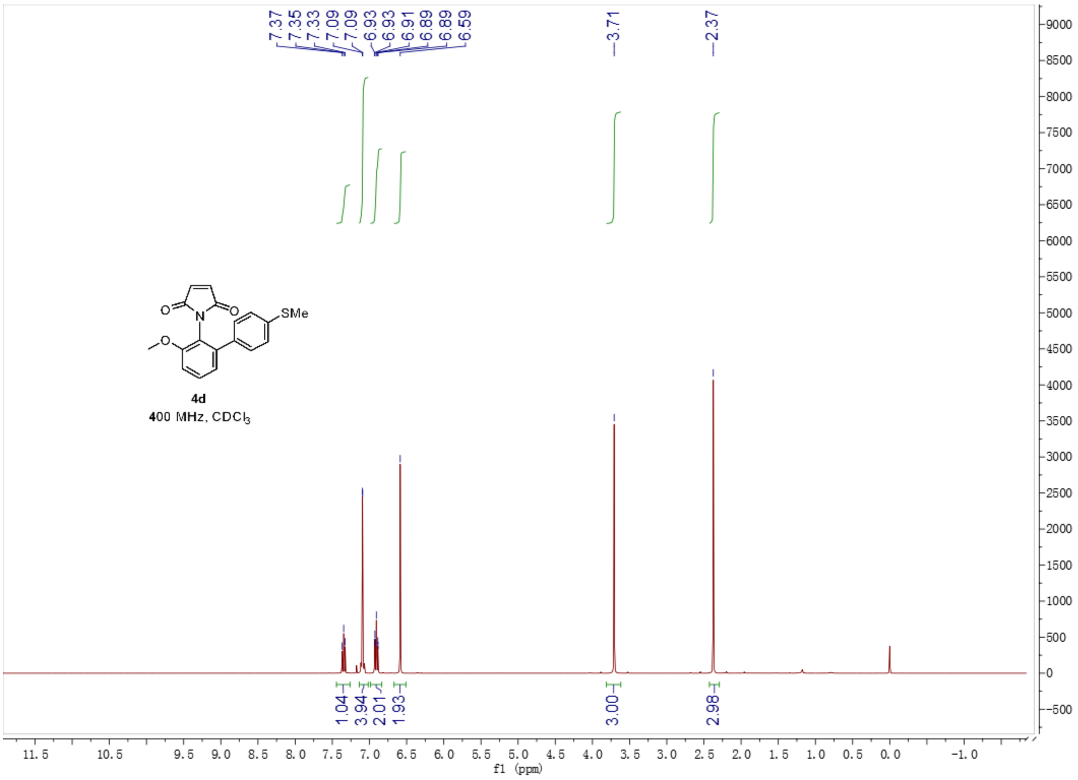

Supplementary Figure 28. ^{1}H NMR spectrum for **4d**

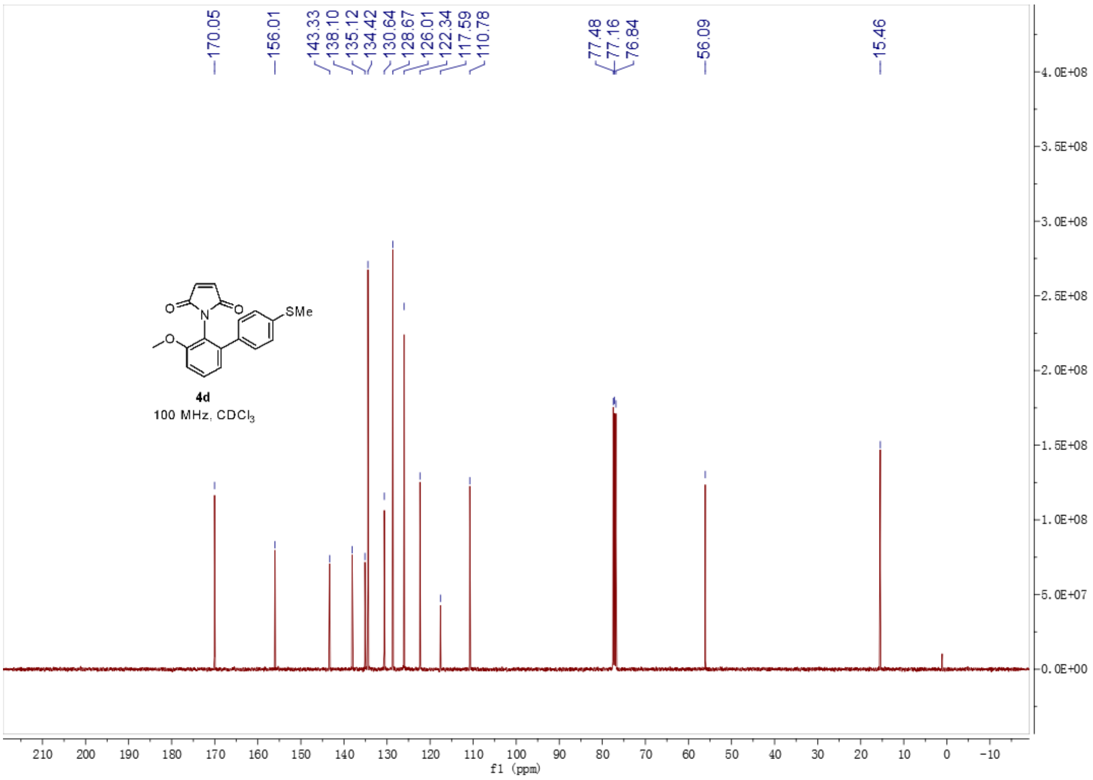

Supplementary Figure 29. ^{13}C NMR spectrum for **4d**

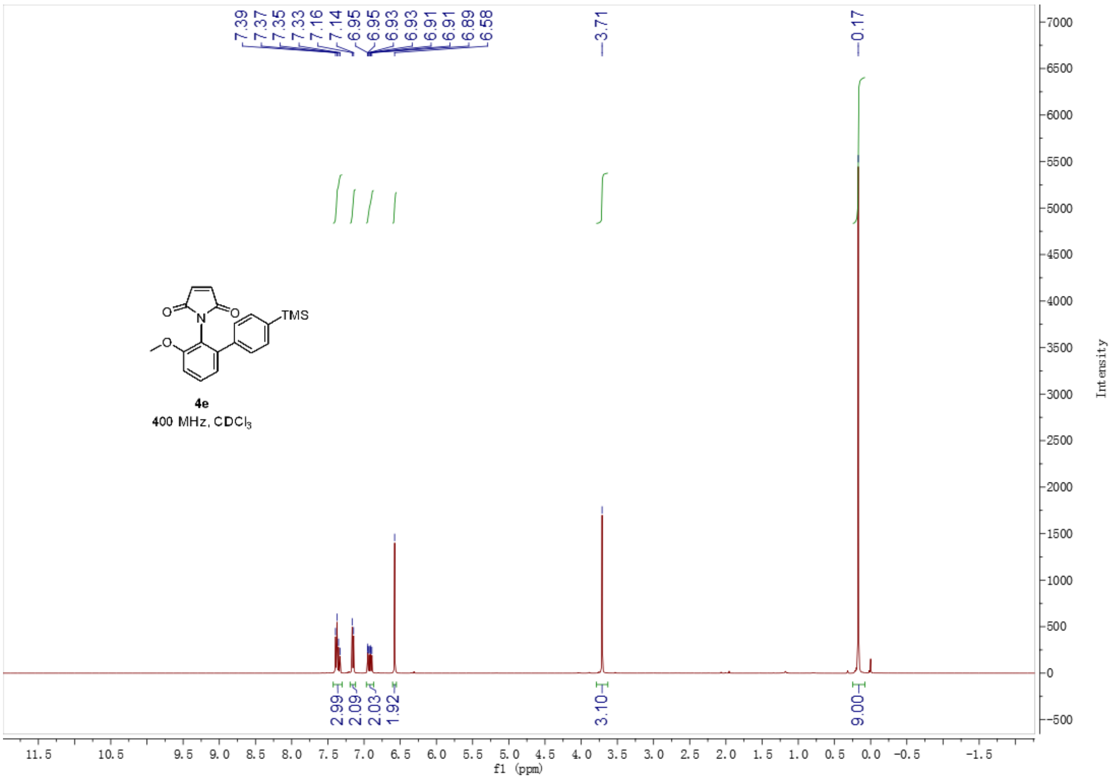

Supplementary Figure 30. ^{1}H NMR spectrum for **4e**

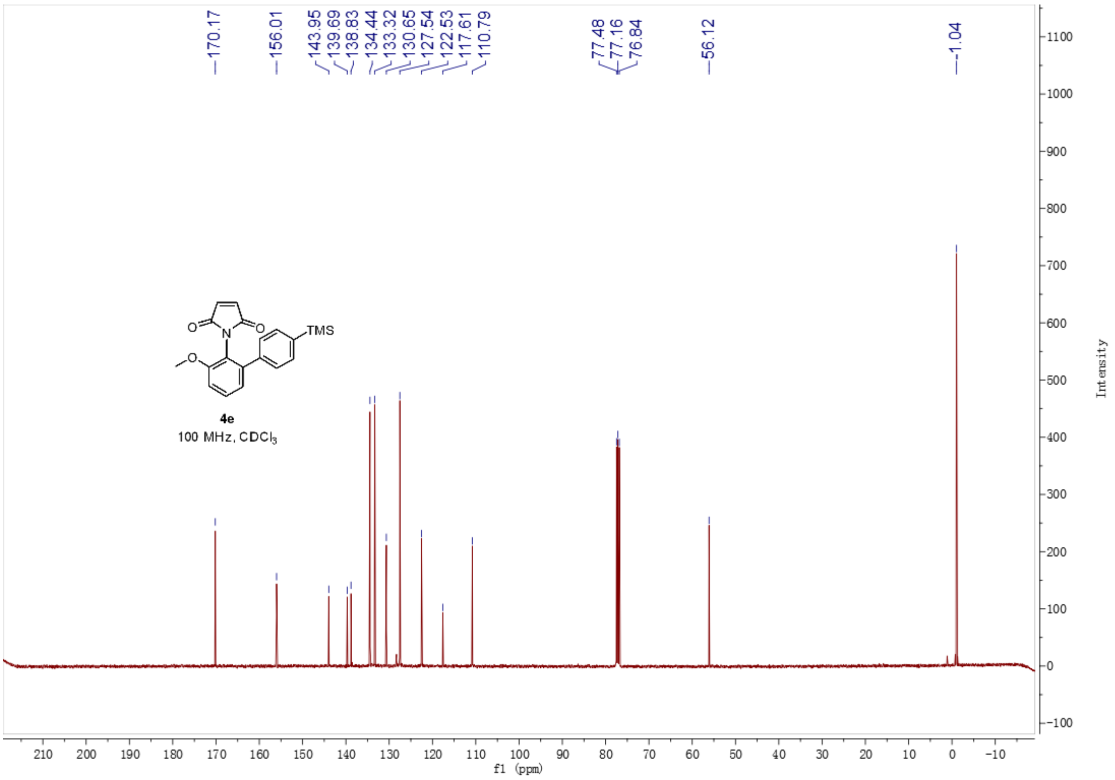

Supplementary Figure 31. ^{13}C NMR spectrum for **4e**

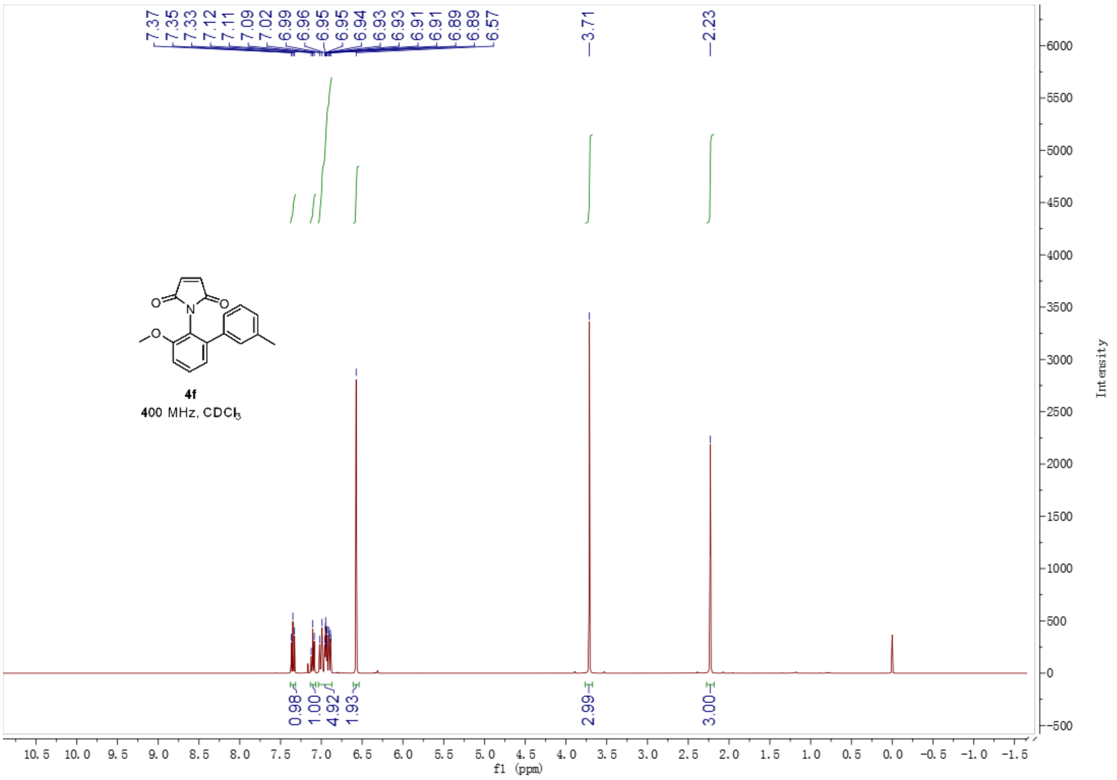

Supplementary Figure 32. ^{1}H NMR spectrum for **4f**

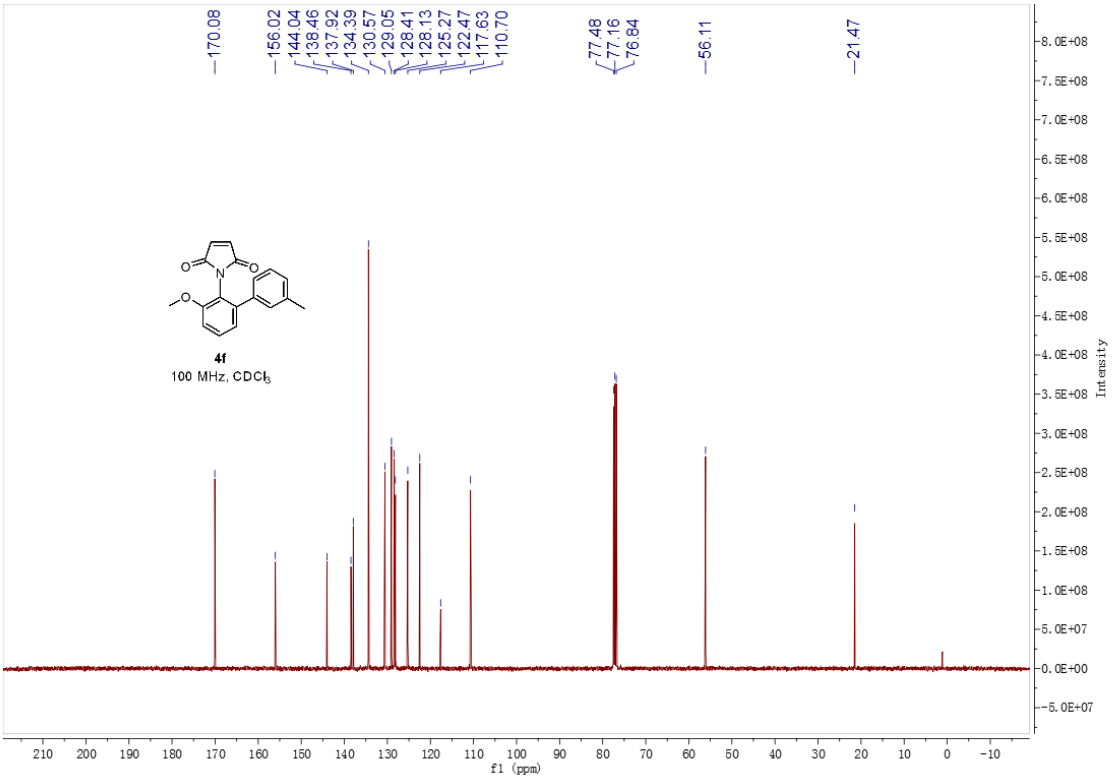

Supplementary Figure 33. ^{13}C NMR spectrum for **4f**

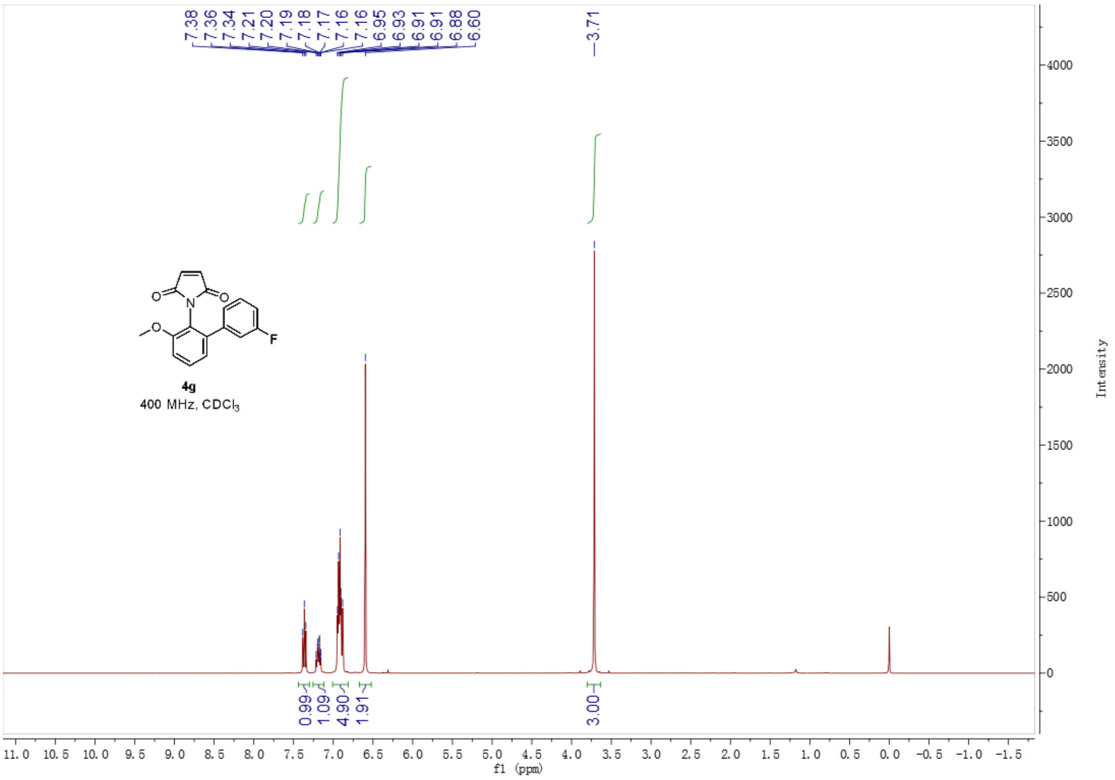

Supplementary Figure 34. ^{1}H NMR spectrum for **4g**

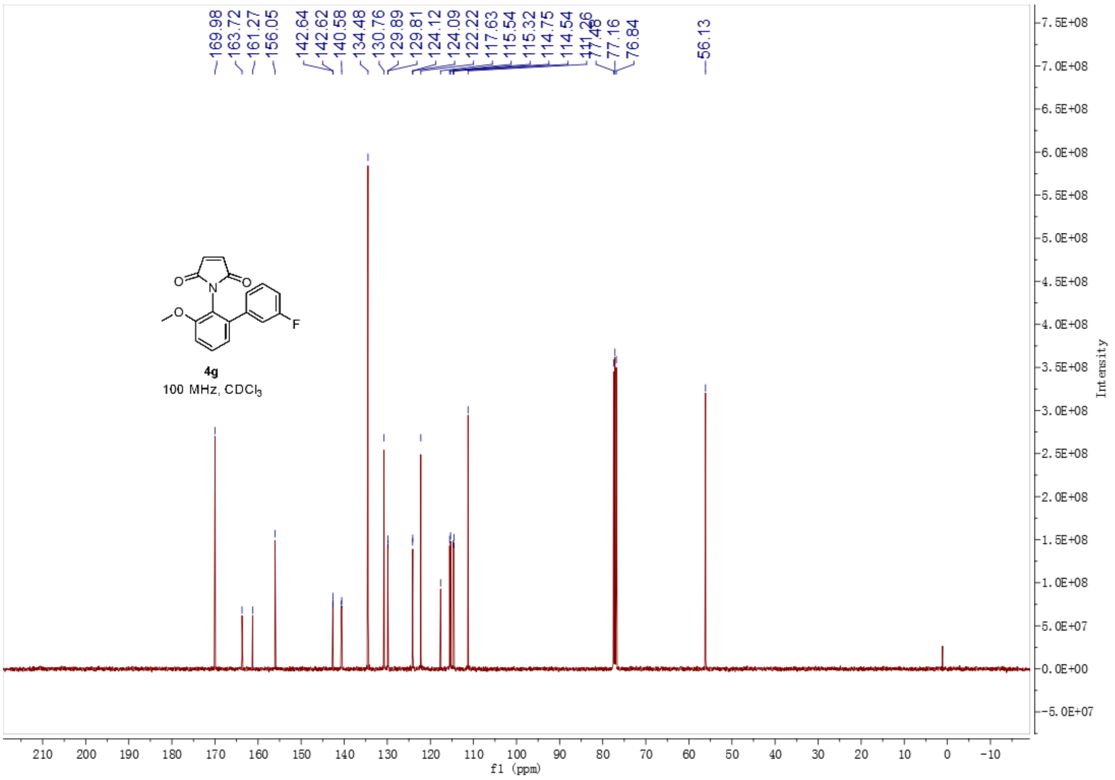

Supplementary Figure 35. ^{13}C NMR spectrum for **4g**

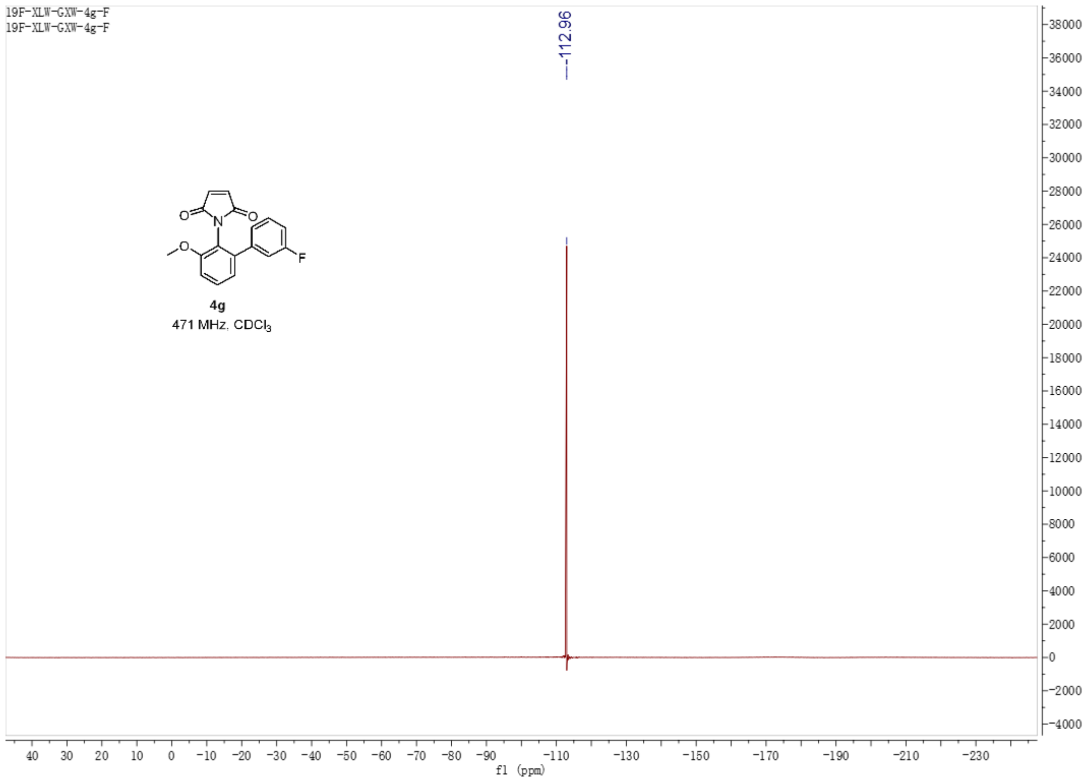

Supplementary Figure 36. ^{19}F NMR spectrum for **4g**

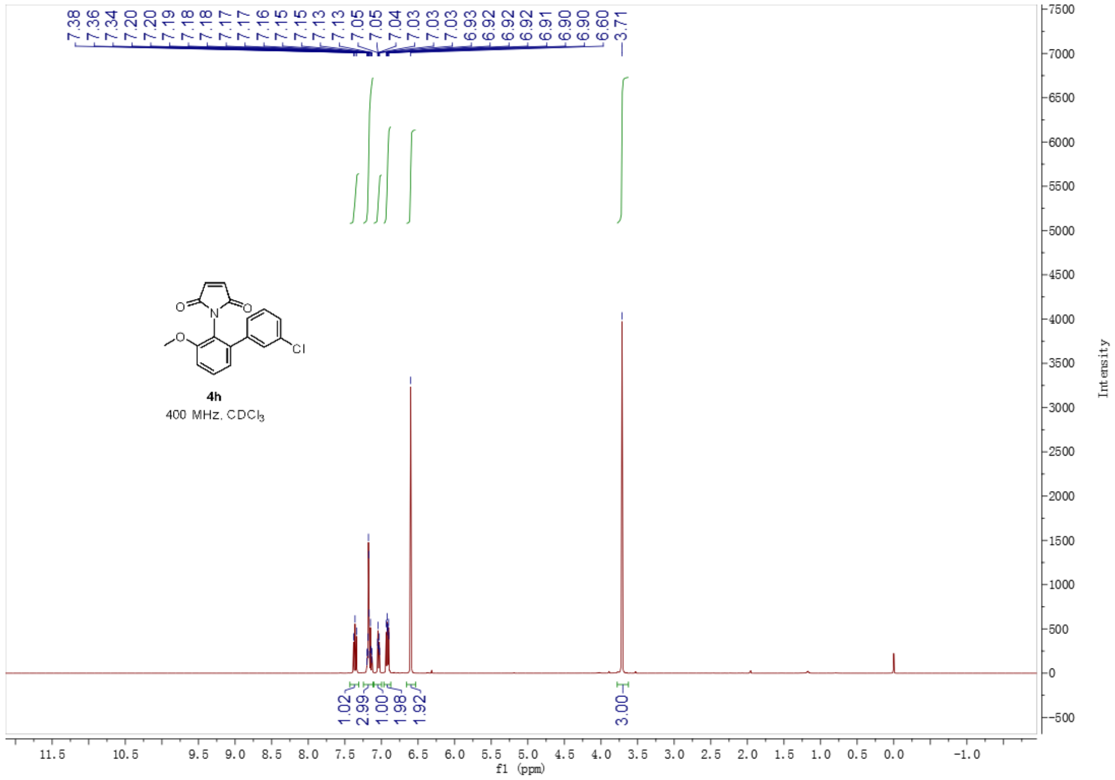

Supplementary Figure 37. ^{1}H NMR spectrum for **4h**

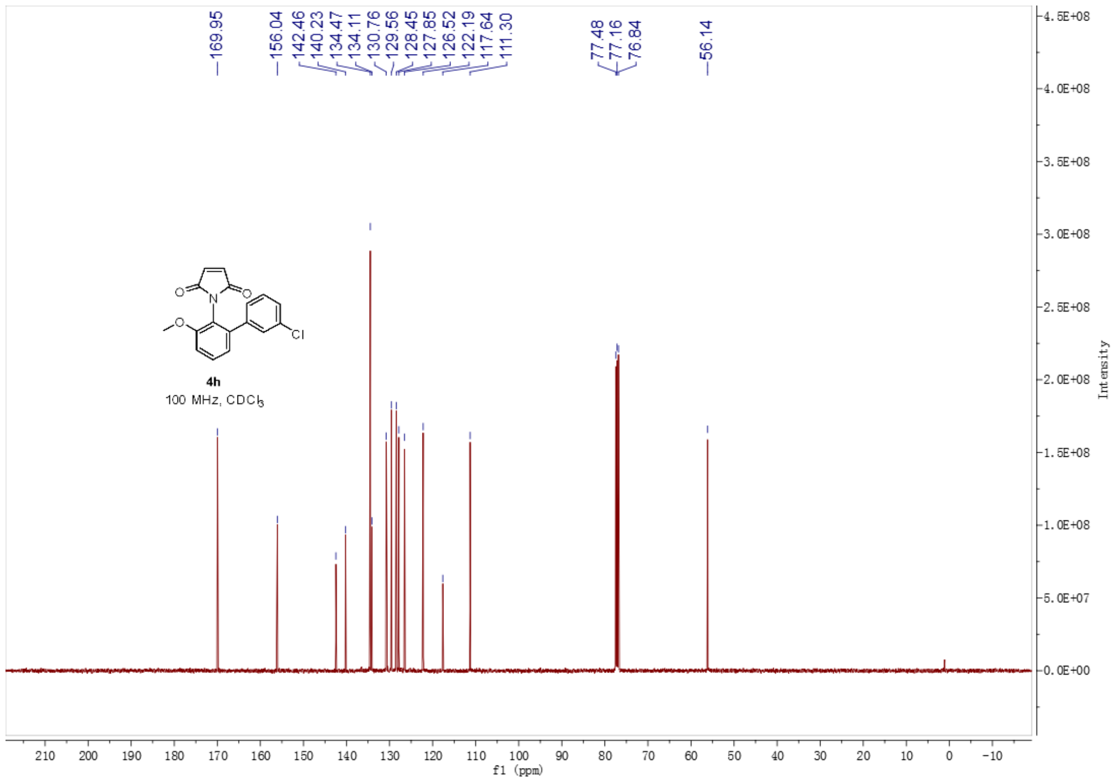

Supplementary Figure 38. ^{13}C NMR spectrum for **4h**

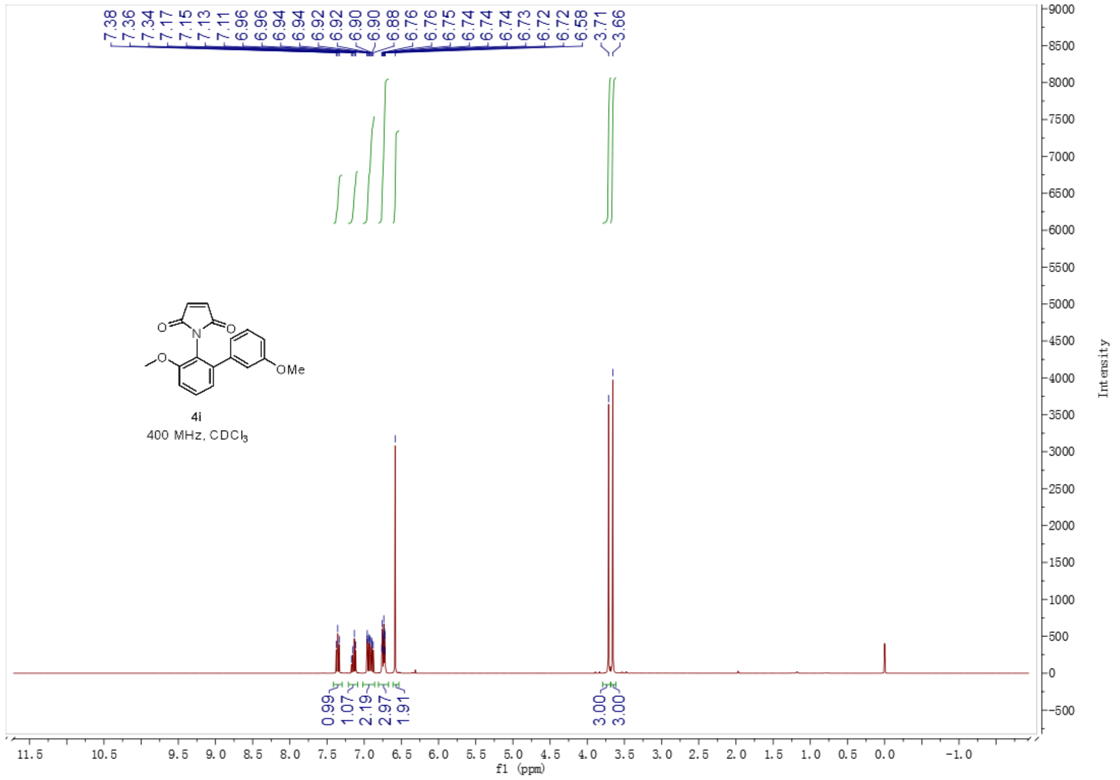

Supplementary Figure 39. ^{1}H NMR spectrum for **4i**

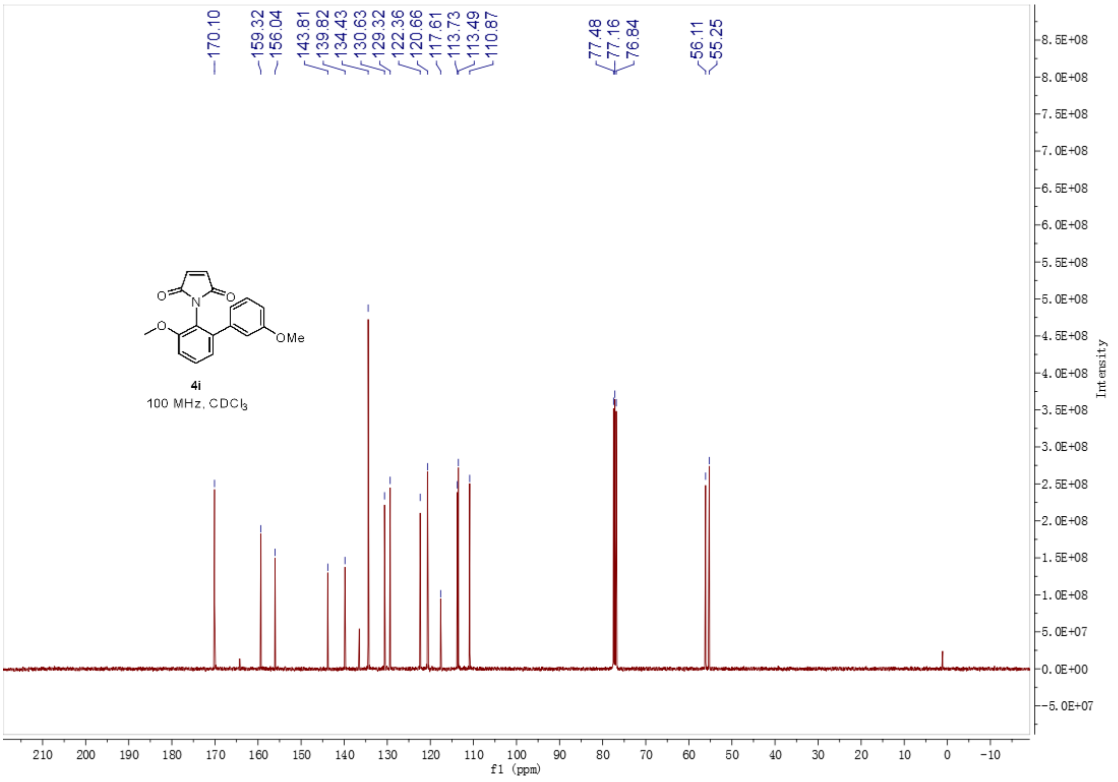

Supplementary Figure 40. ^{13}C NMR spectrum for **4i**

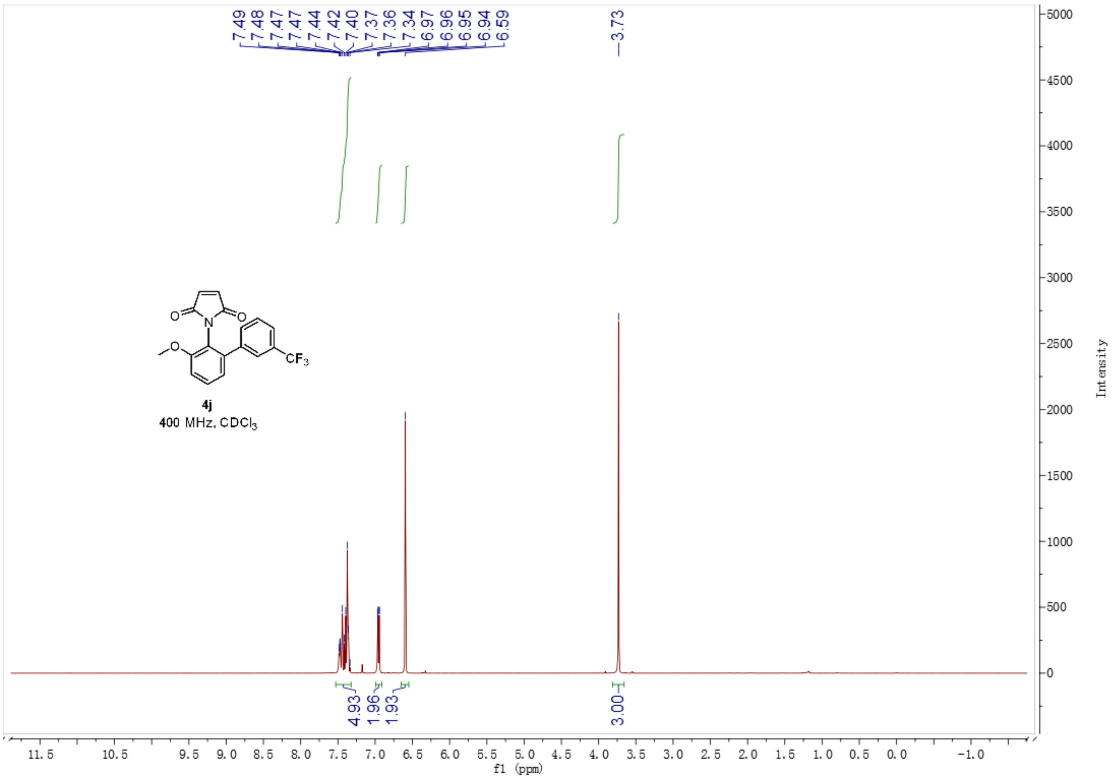

Supplementary Figure 41. ^{1}H NMR spectrum for **4j**

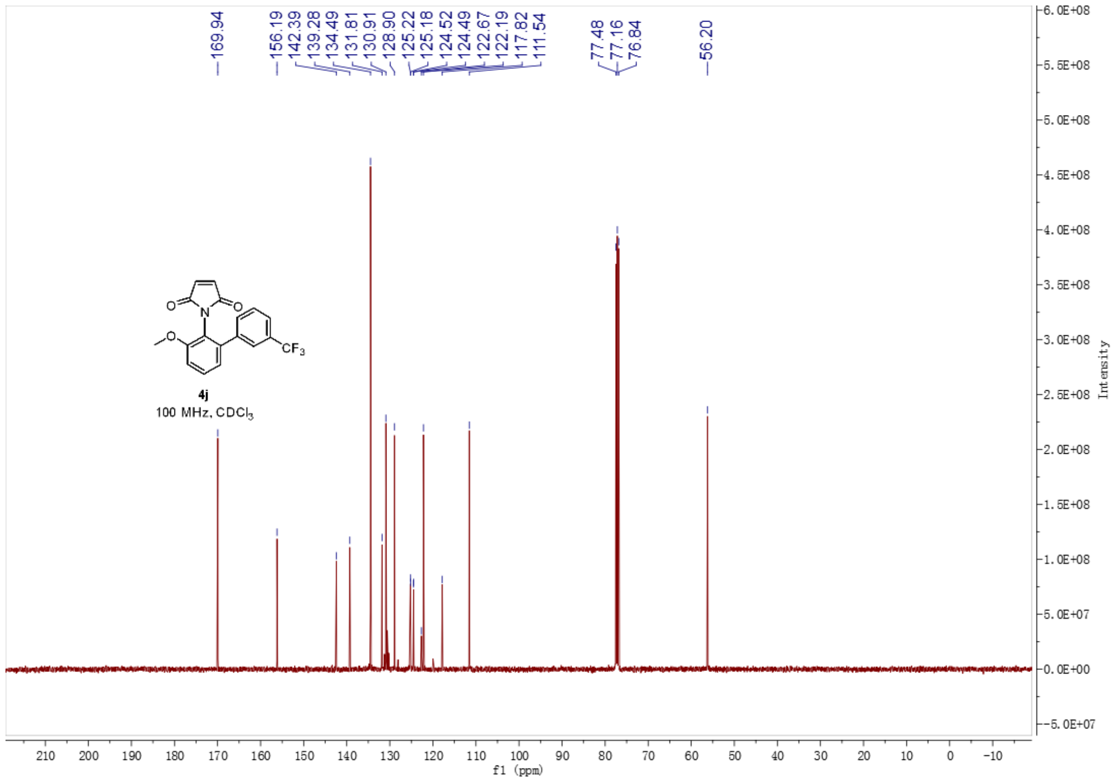

Supplementary Figure 42. ^{13}C NMR spectrum for **4j**

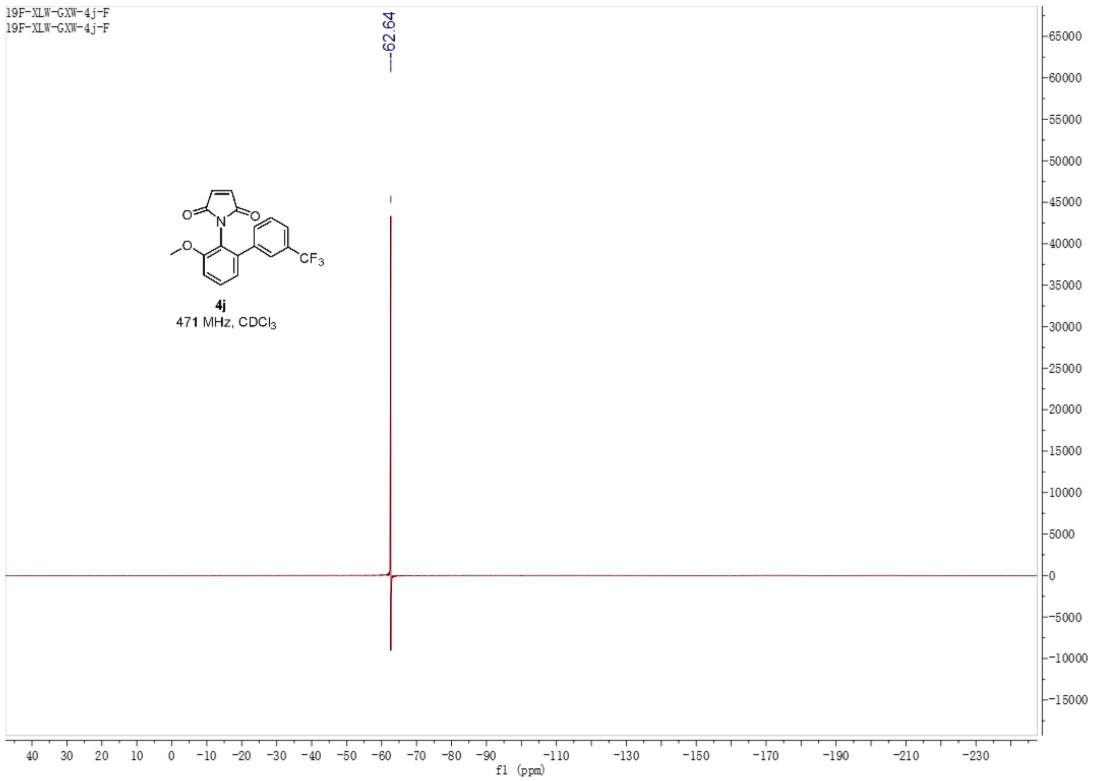

Supplementary Figure 43. ^{19}F NMR spectrum for **4j**

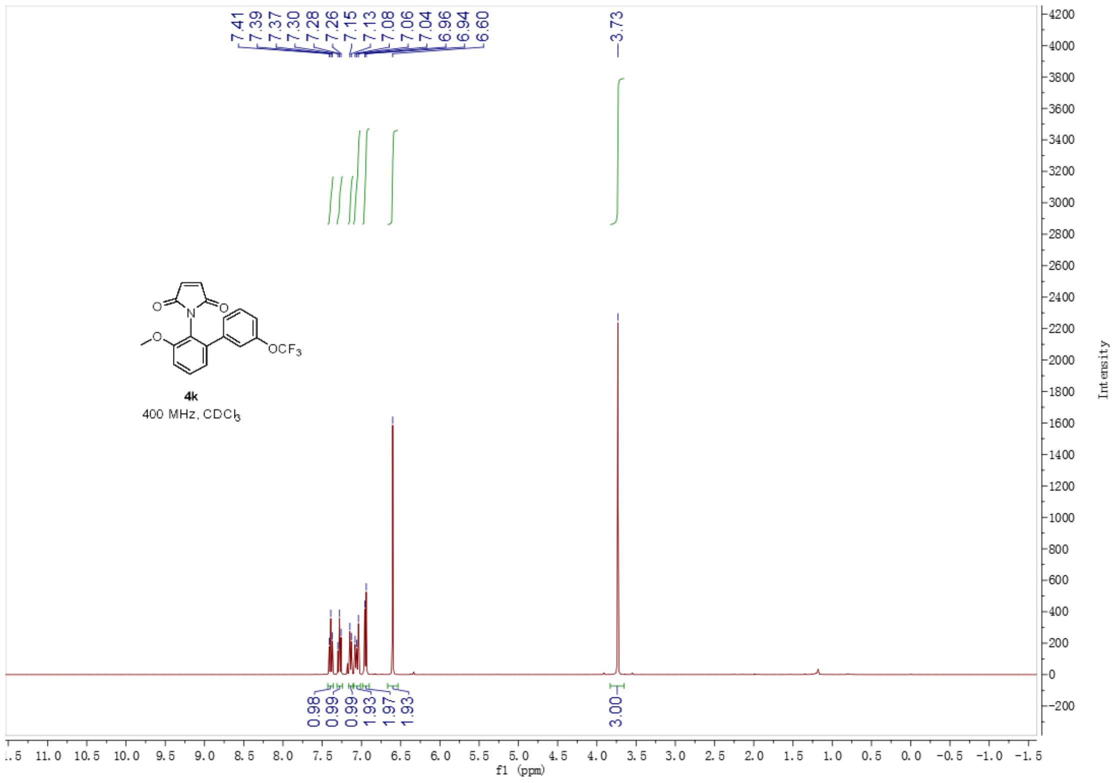

Supplementary Figure 44. ^{1}H NMR spectrum for **4k**

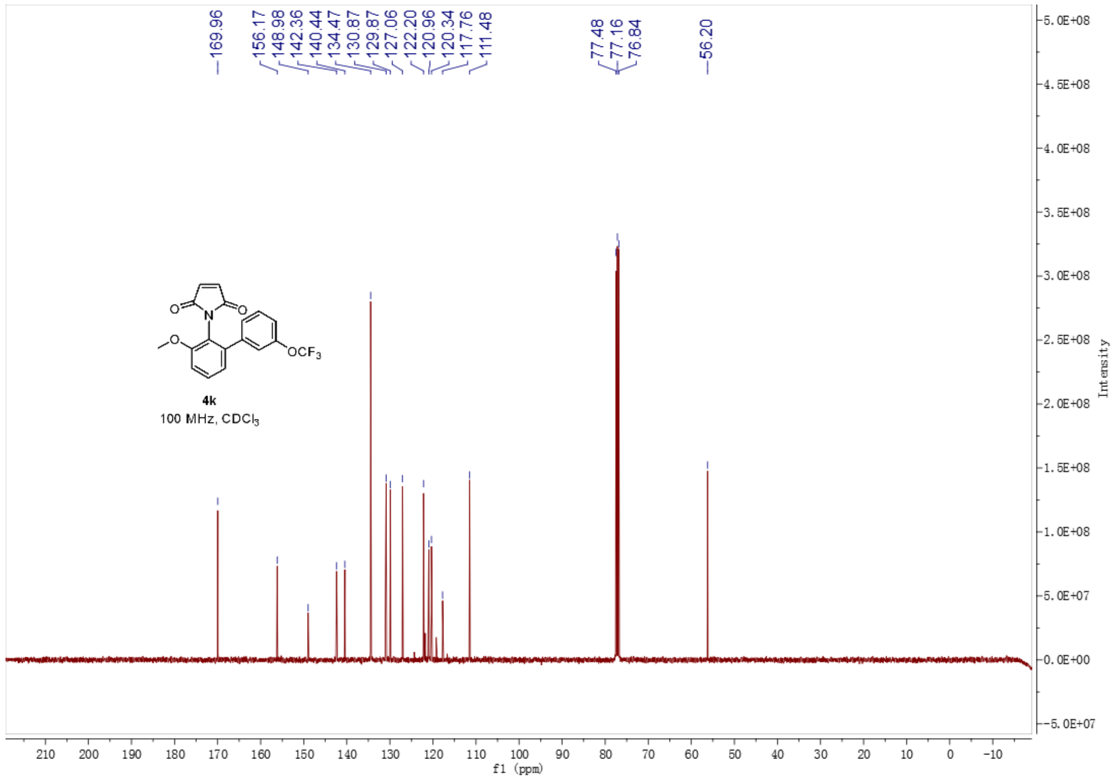

Supplementary Figure 45. ^{13}C NMR spectrum for **4k**

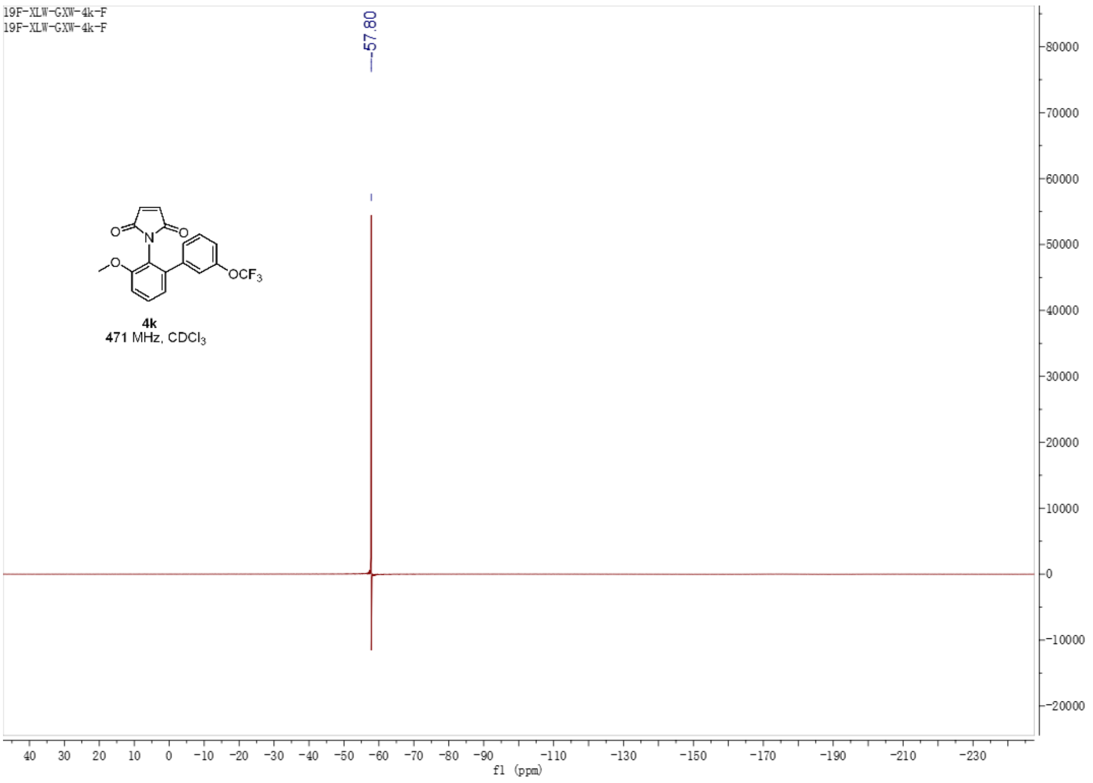

Supplementary Figure 46. ^{19}F NMR spectrum for **4k**

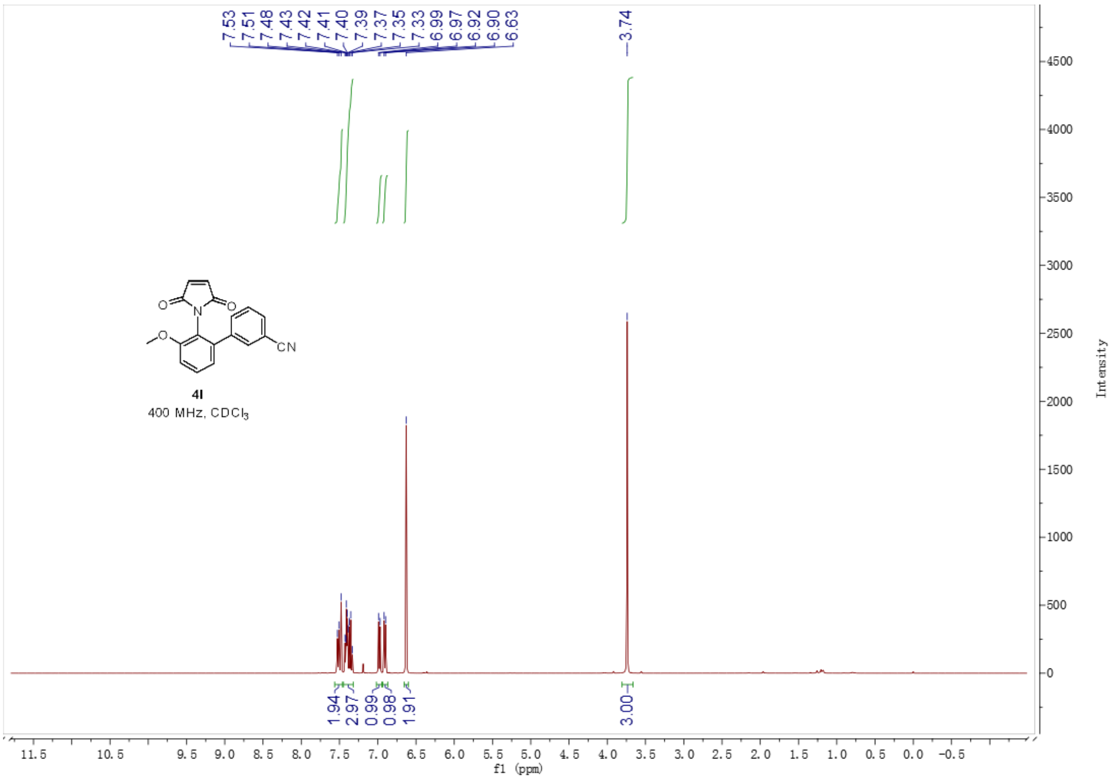

Supplementary Figure 47. ^{1}H NMR spectrum for **4l**

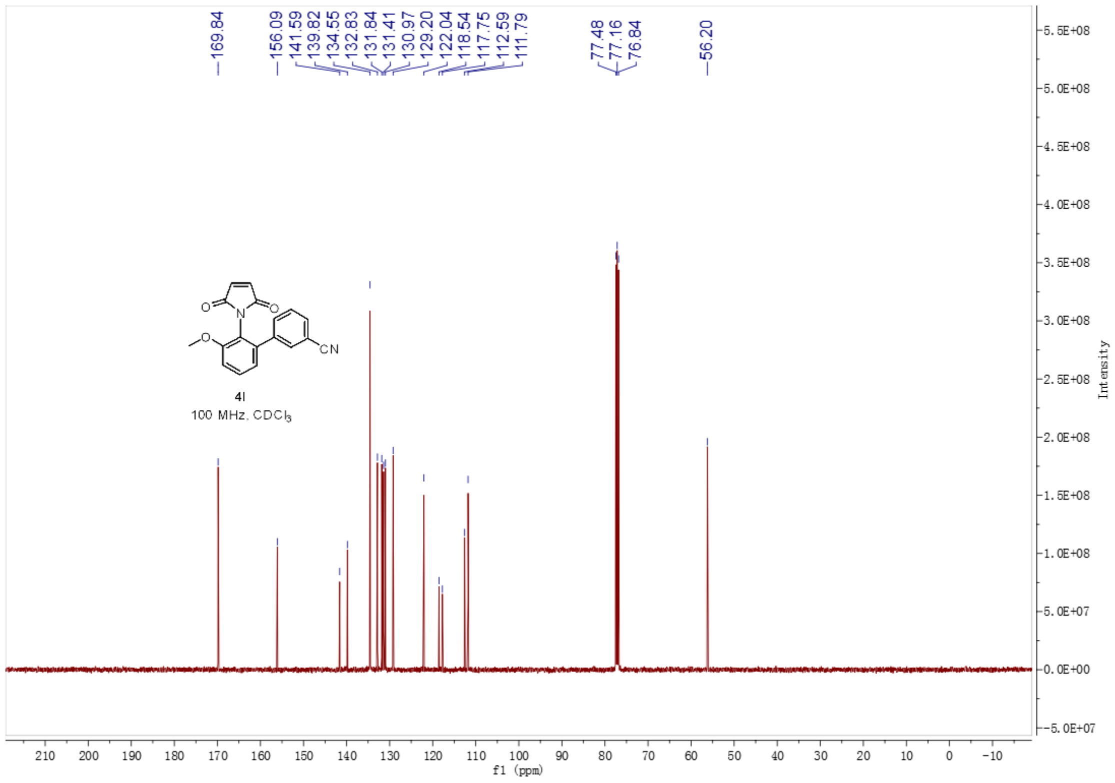

Supplementary Figure 48. ^{13}C NMR spectrum for **4l**

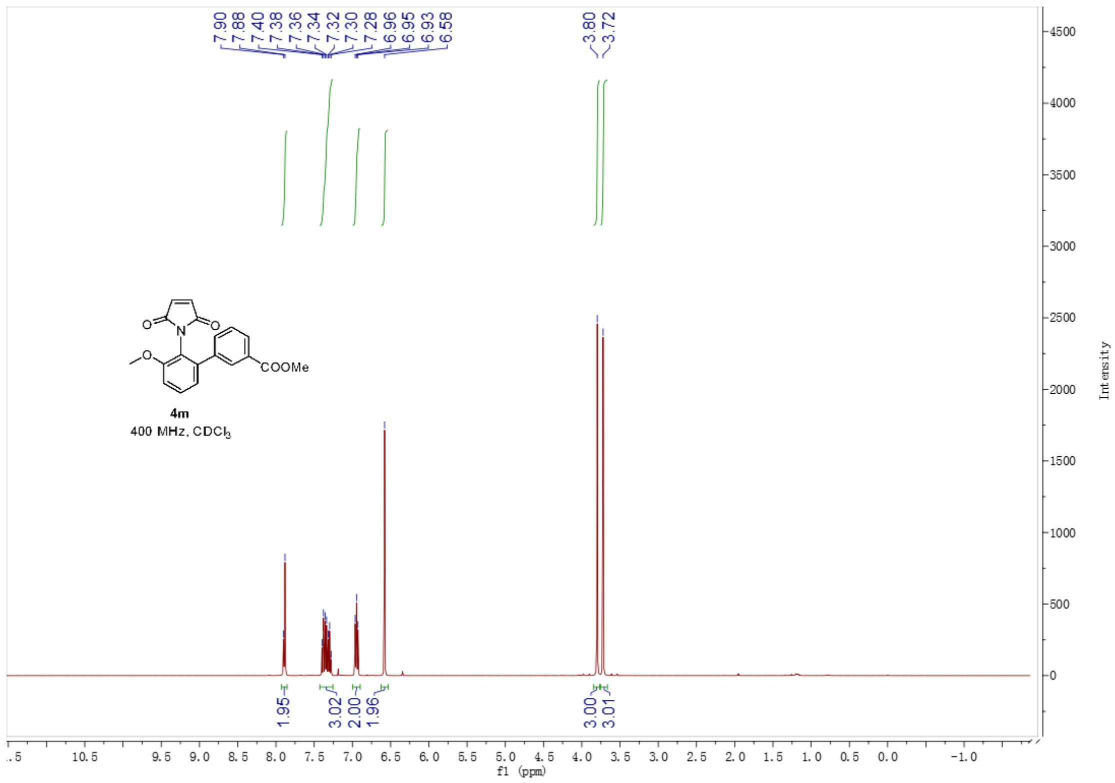

Supplementary Figure 49. ^{1}H NMR spectrum for **4m**

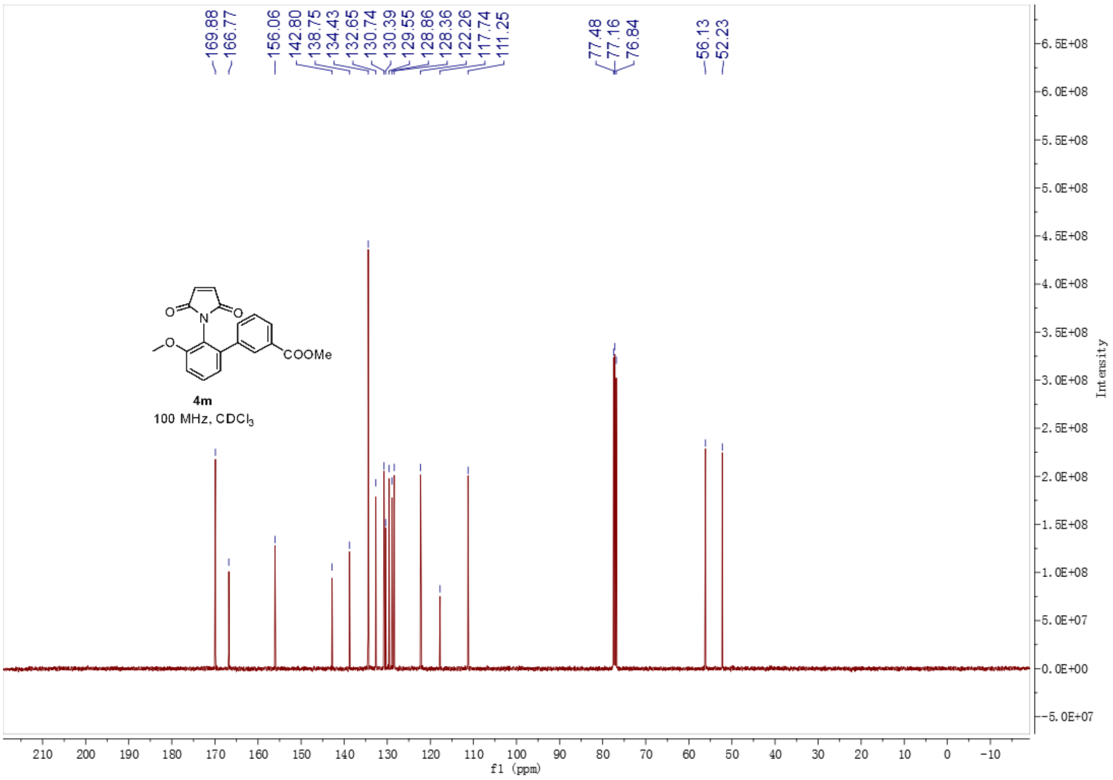

Supplementary Figure 50. ^{13}C NMR spectrum for **4m**

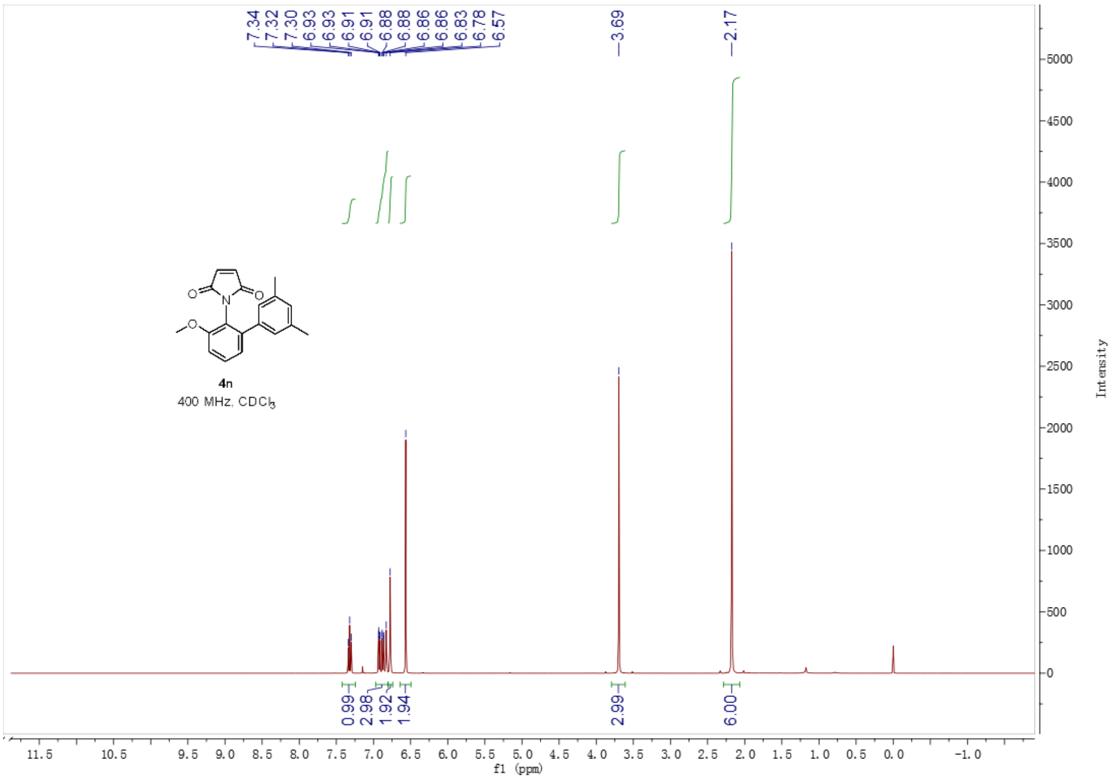

Supplementary Figure 51. ^{1}H NMR spectrum for **4n**

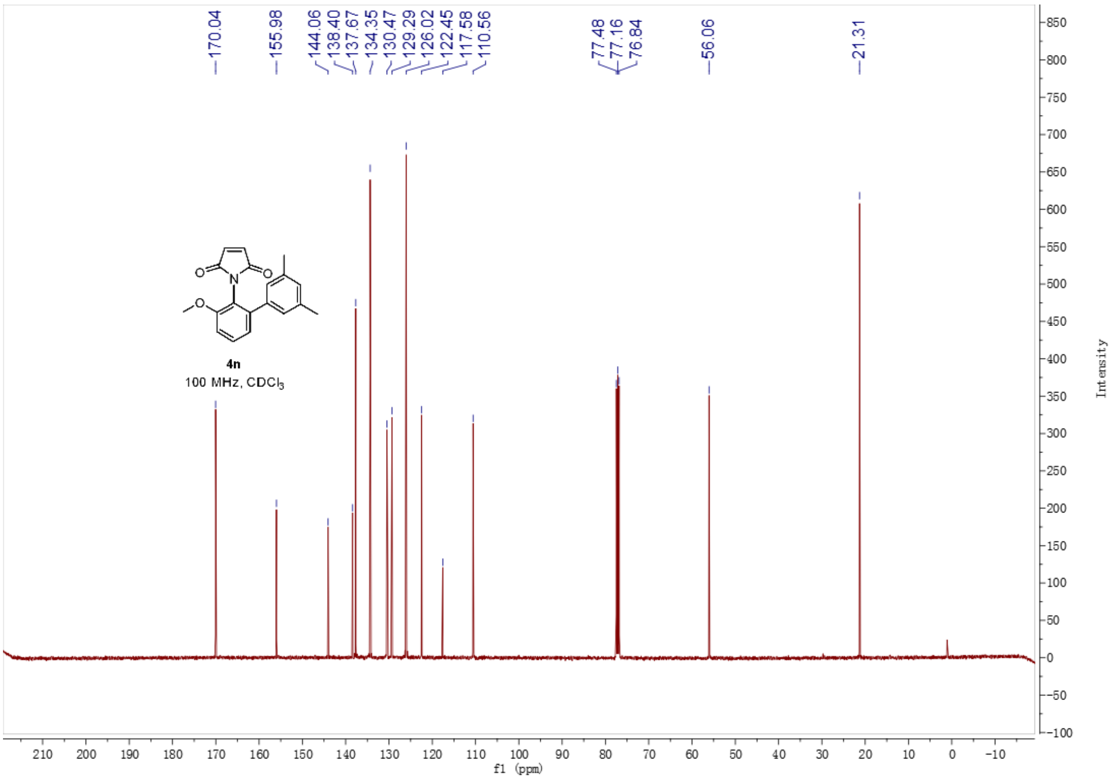

Supplementary Figure 52. ^{13}C NMR spectrum for **4n**

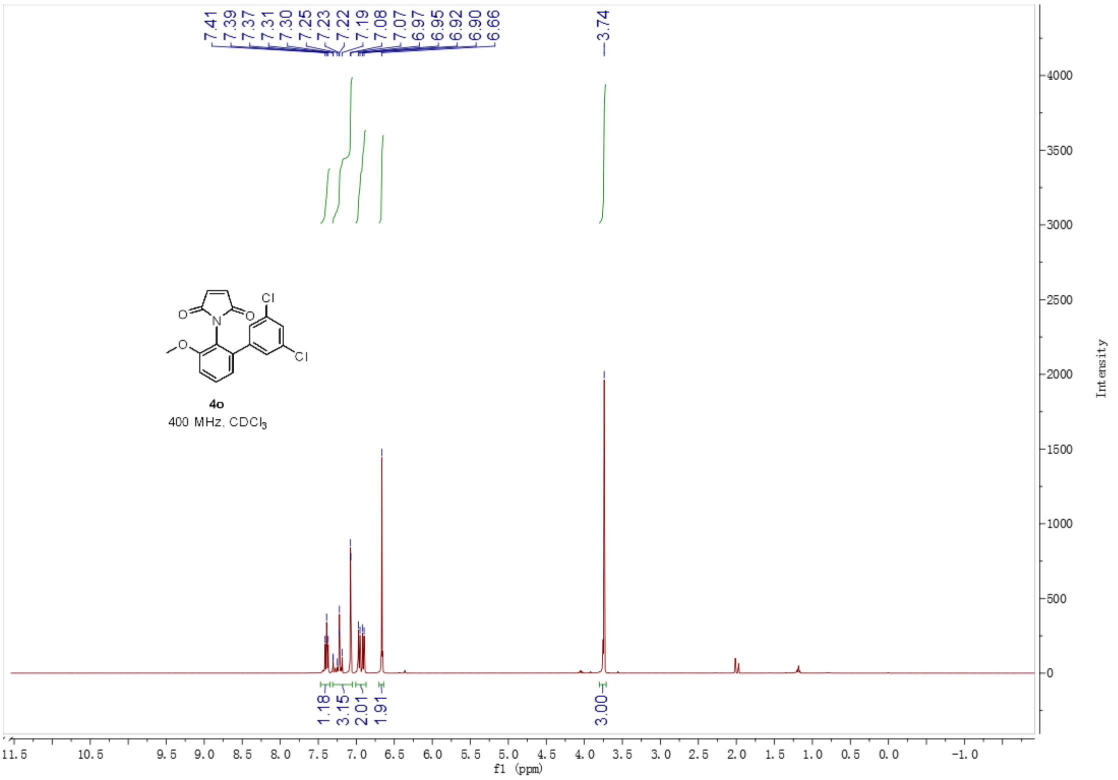

Supplementary Figure 53. ^{1}H NMR spectrum for **4o**

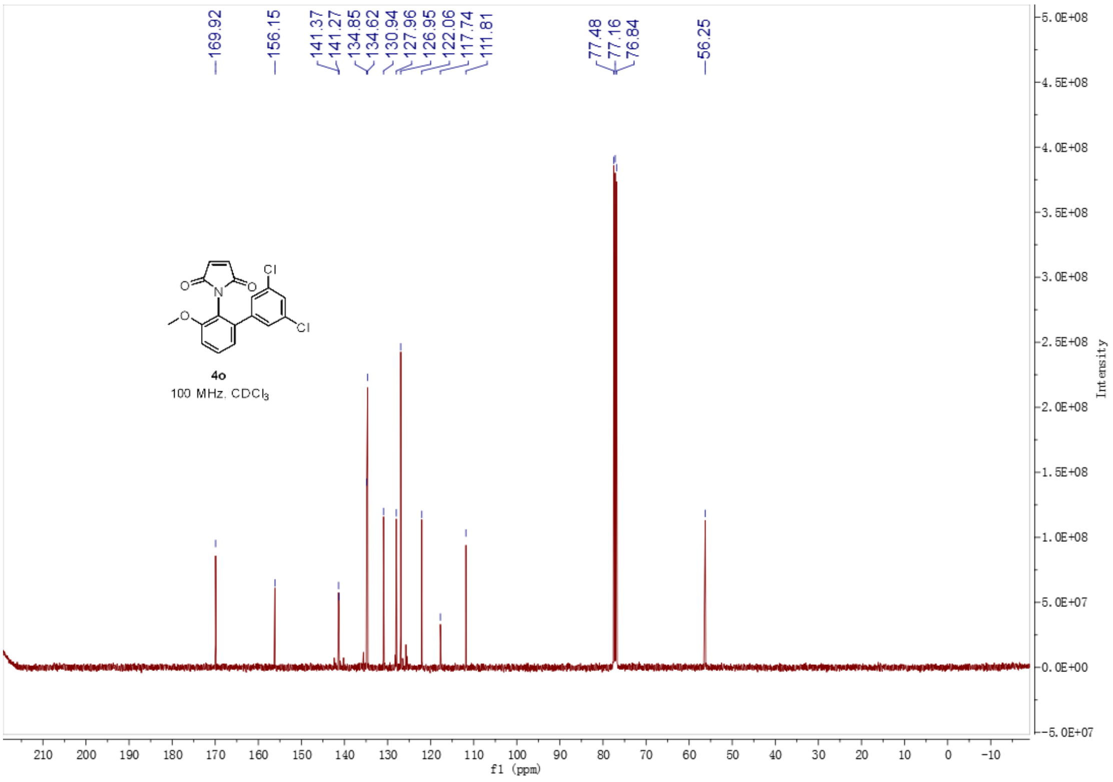

Supplementary Figure 54. ^{13}C NMR spectrum for **4o**

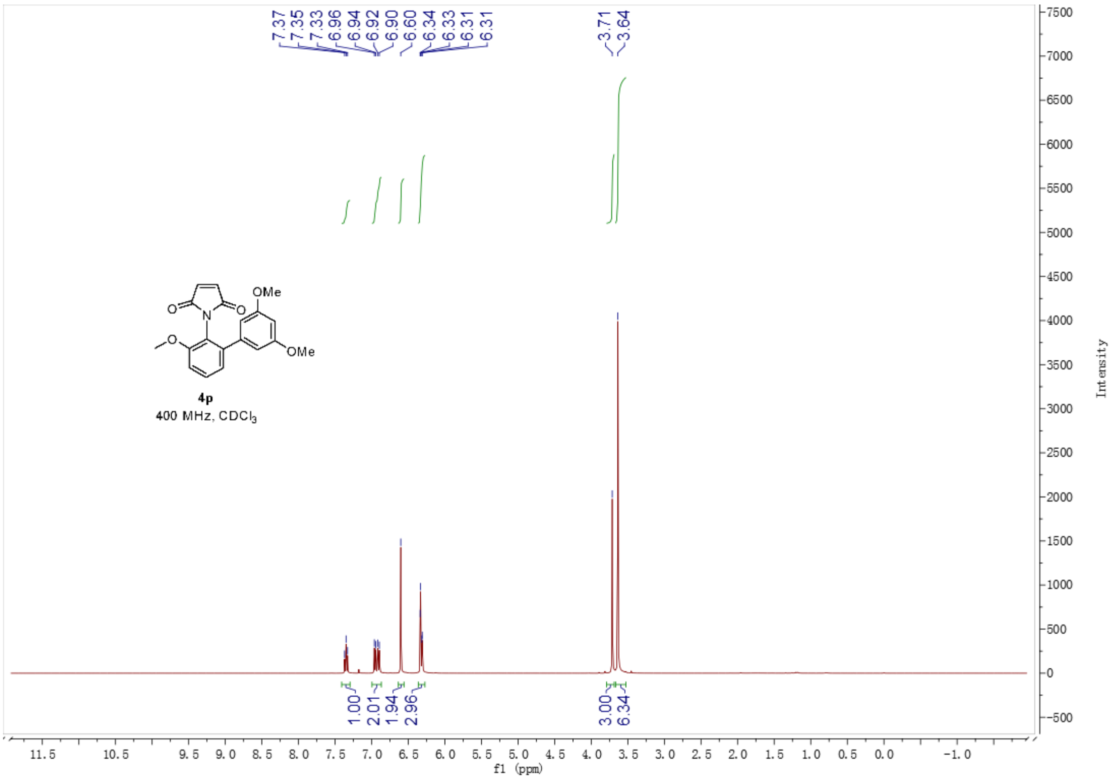

Supplementary Figure 55. ^{1}H NMR spectrum for **4p**

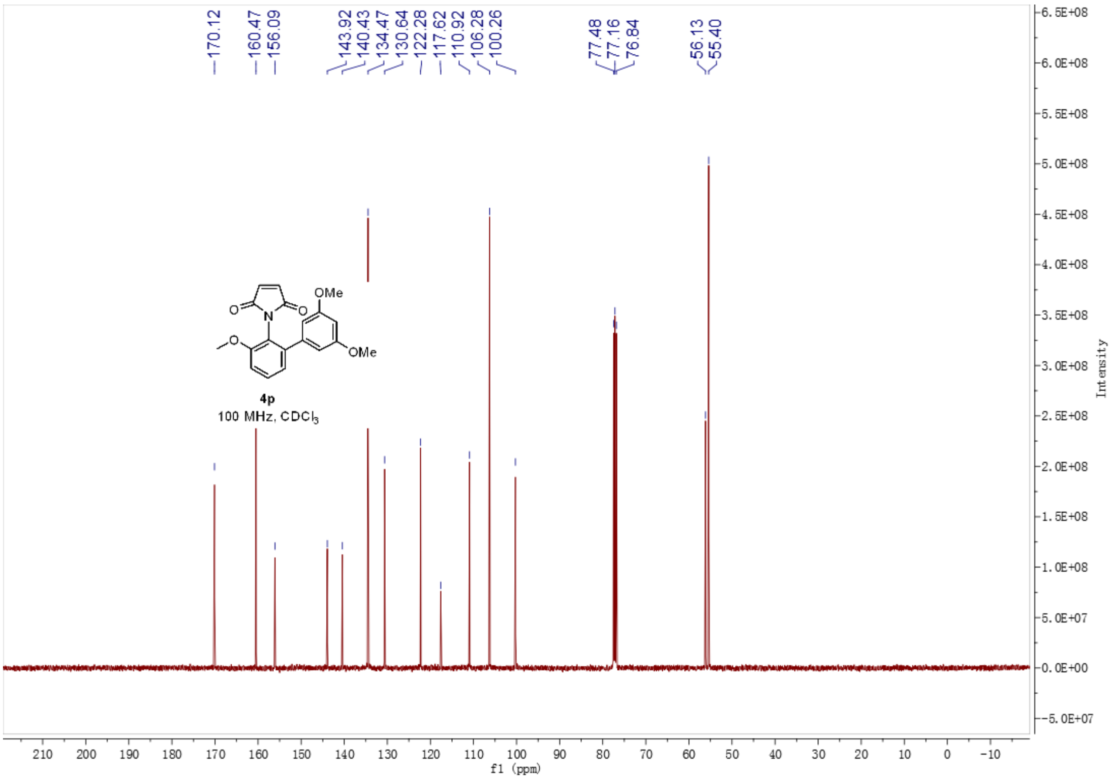

Supplementary Figure 56. ^{13}C NMR spectrum for **4p**

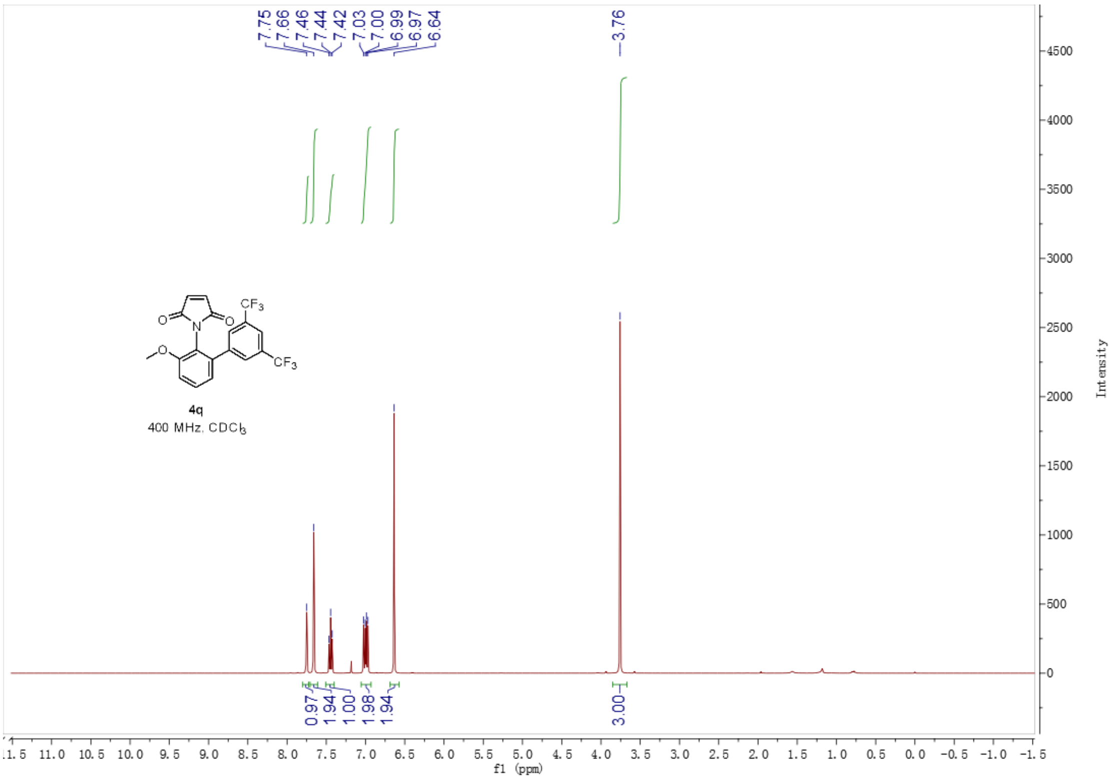

Supplementary Figure 57. ^{1}H NMR spectrum for **4q**

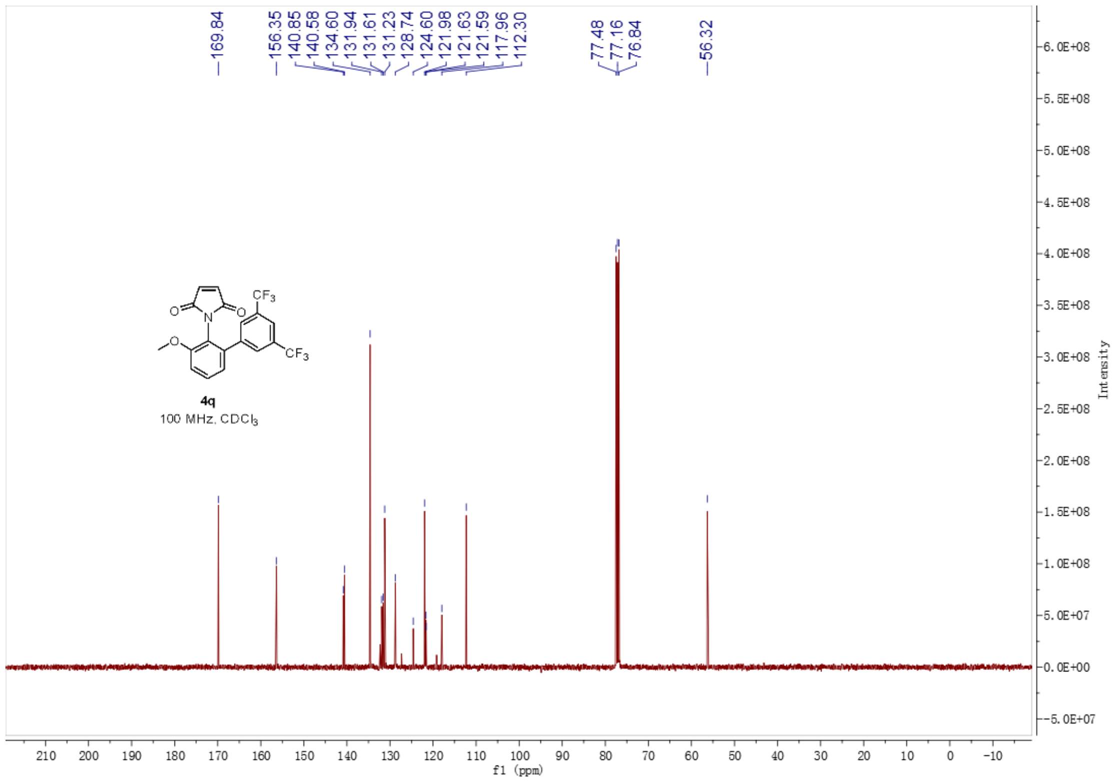

Supplementary Figure 58. ^{13}C NMR spectrum for **4q**

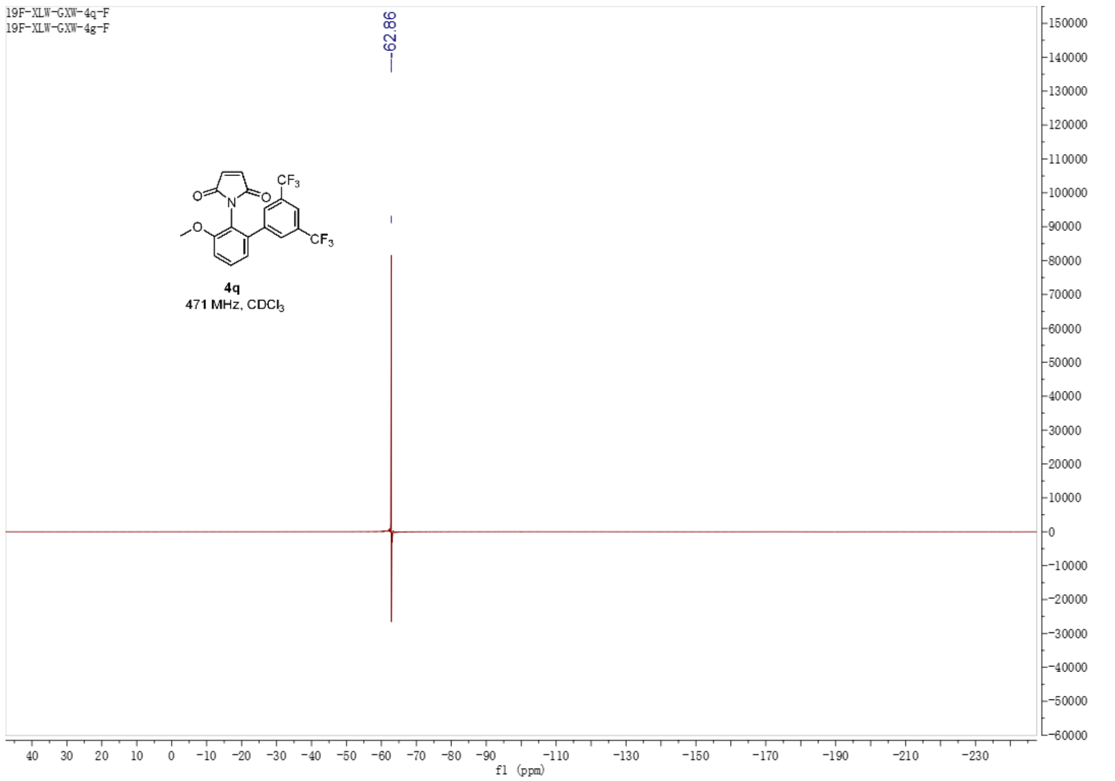

Supplementary Figure 59. ^{19}F NMR spectrum for **4q**

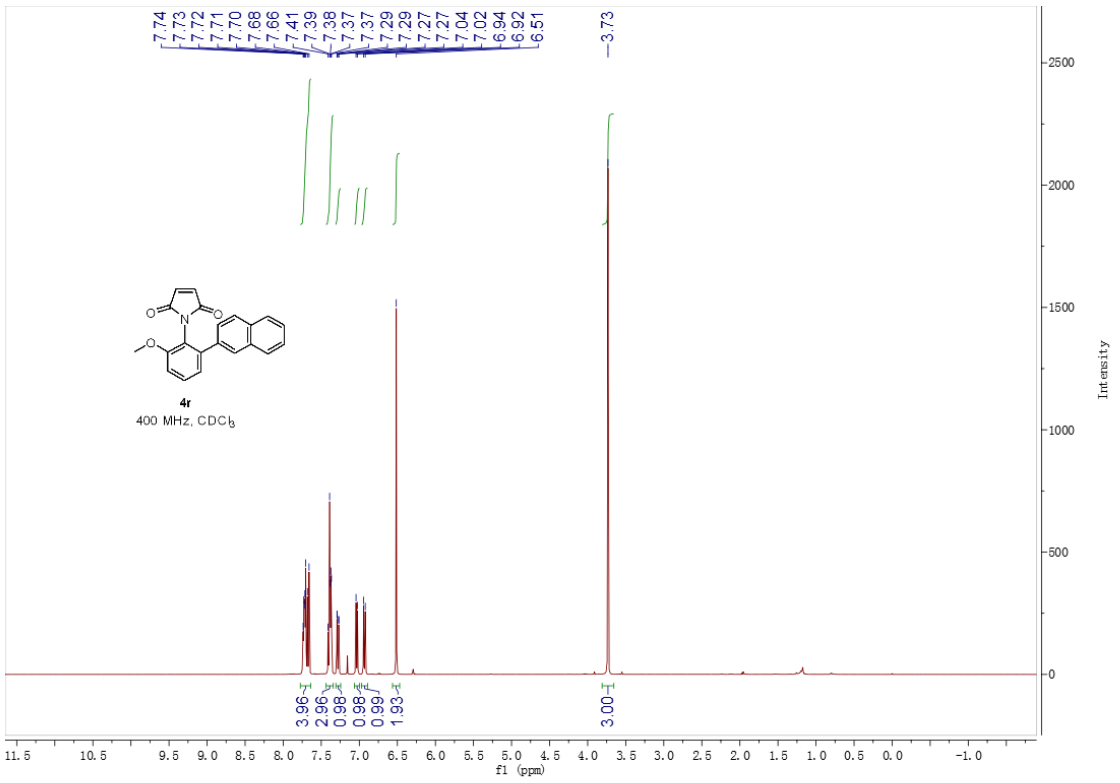

Supplementary Figure 60. ^{1}H NMR spectrum for **4r**

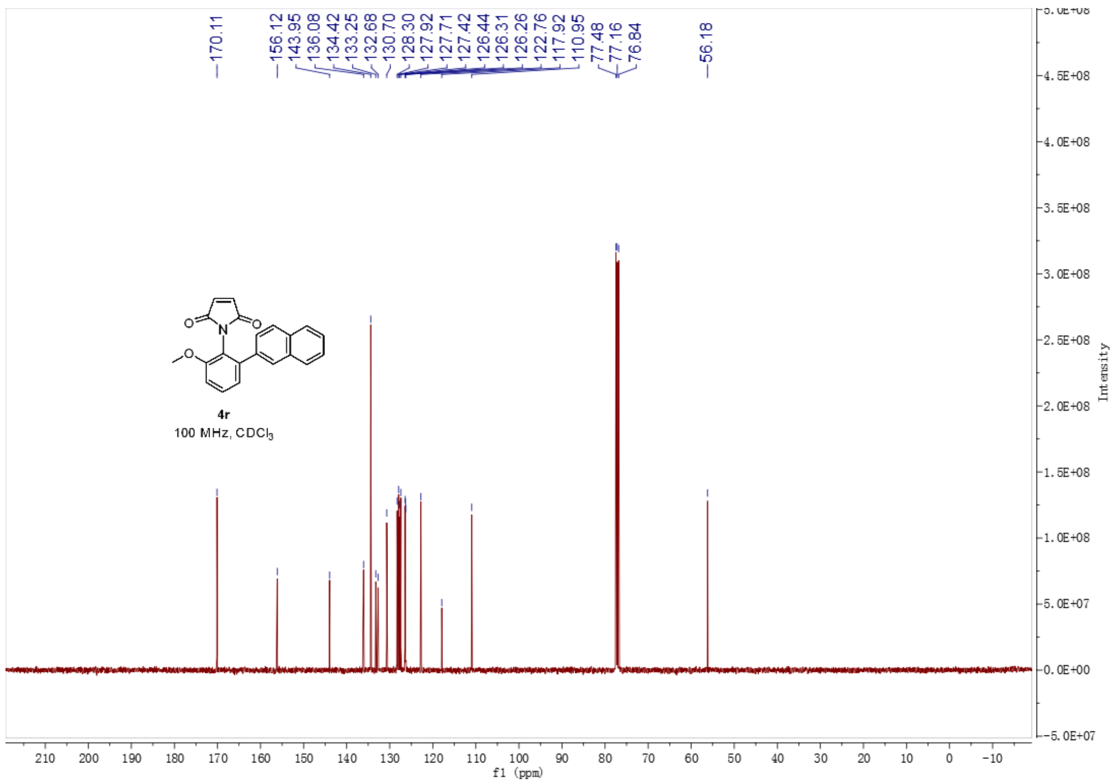

Supplementary Figure 61. ^{13}C NMR spectrum for **4r**

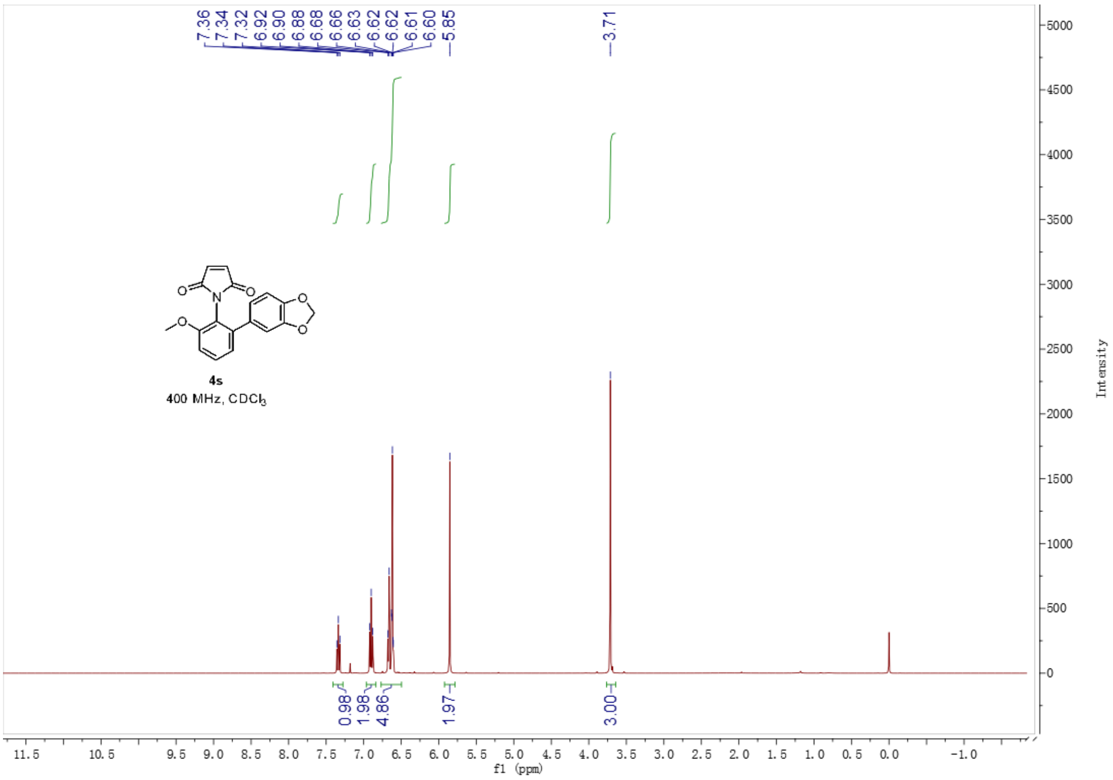

Supplementary Figure 62. ^{1}H NMR spectrum for **4s**

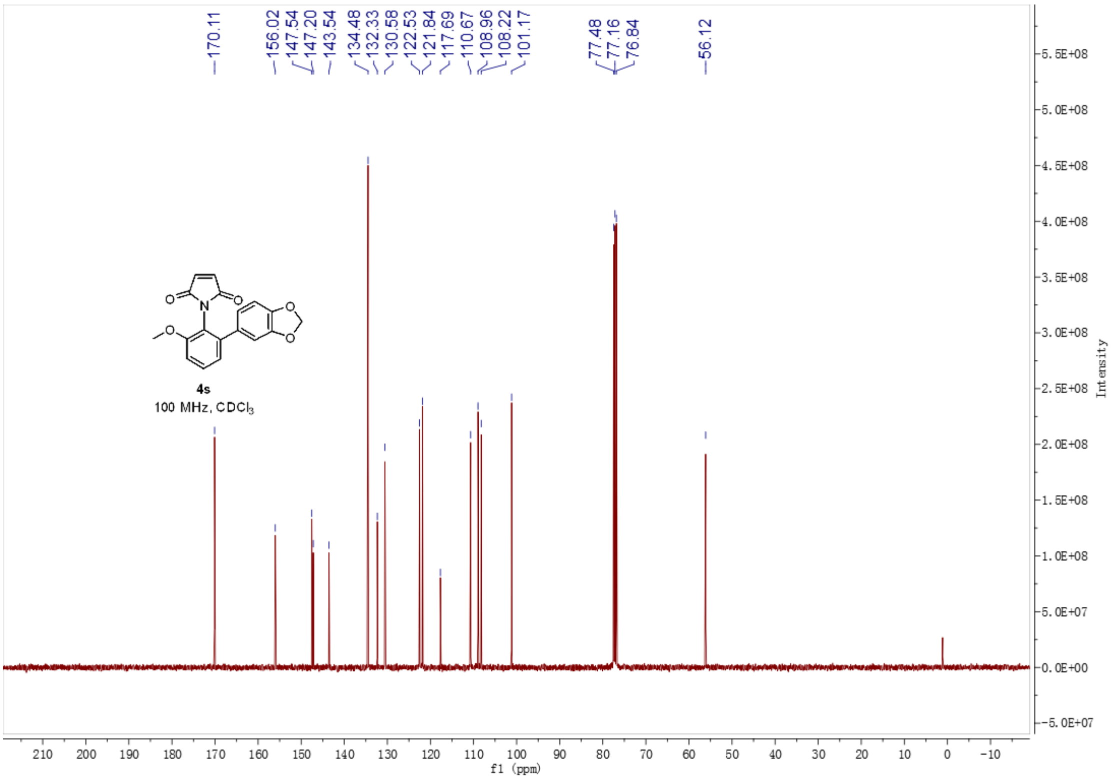

Supplementary Figure 63. ^{13}C NMR spectrum for **4s**

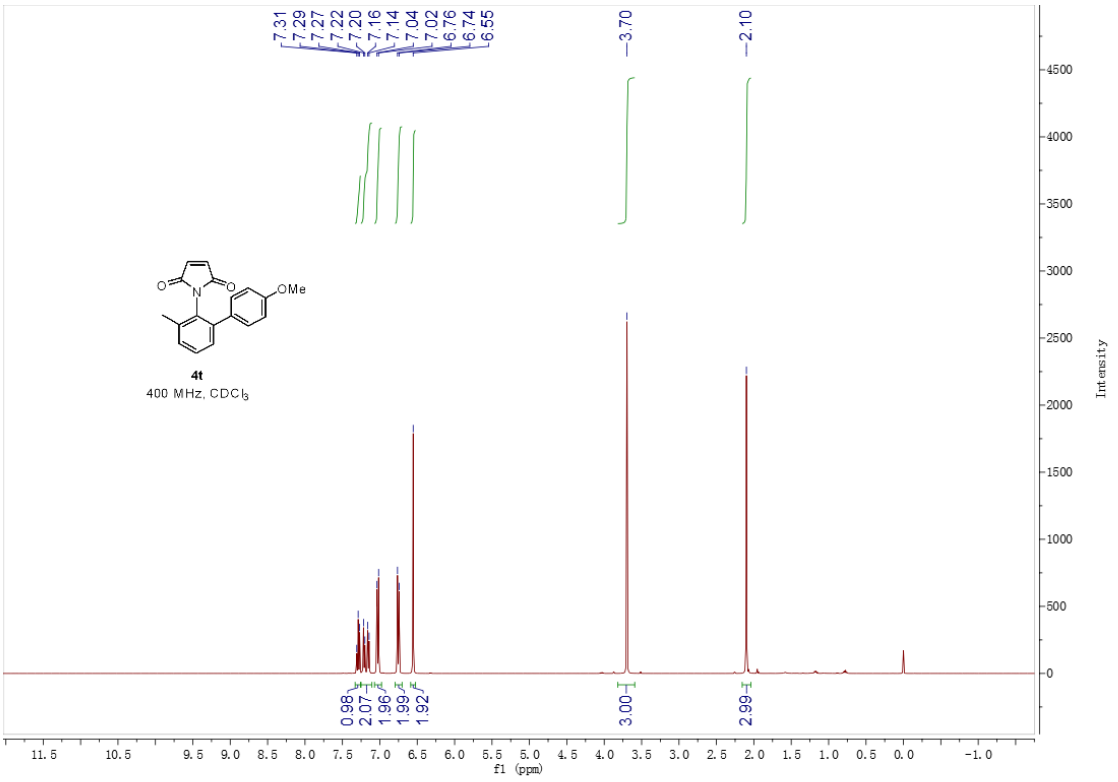

Supplementary Figure 64. ^{1}H NMR spectrum for **4t**

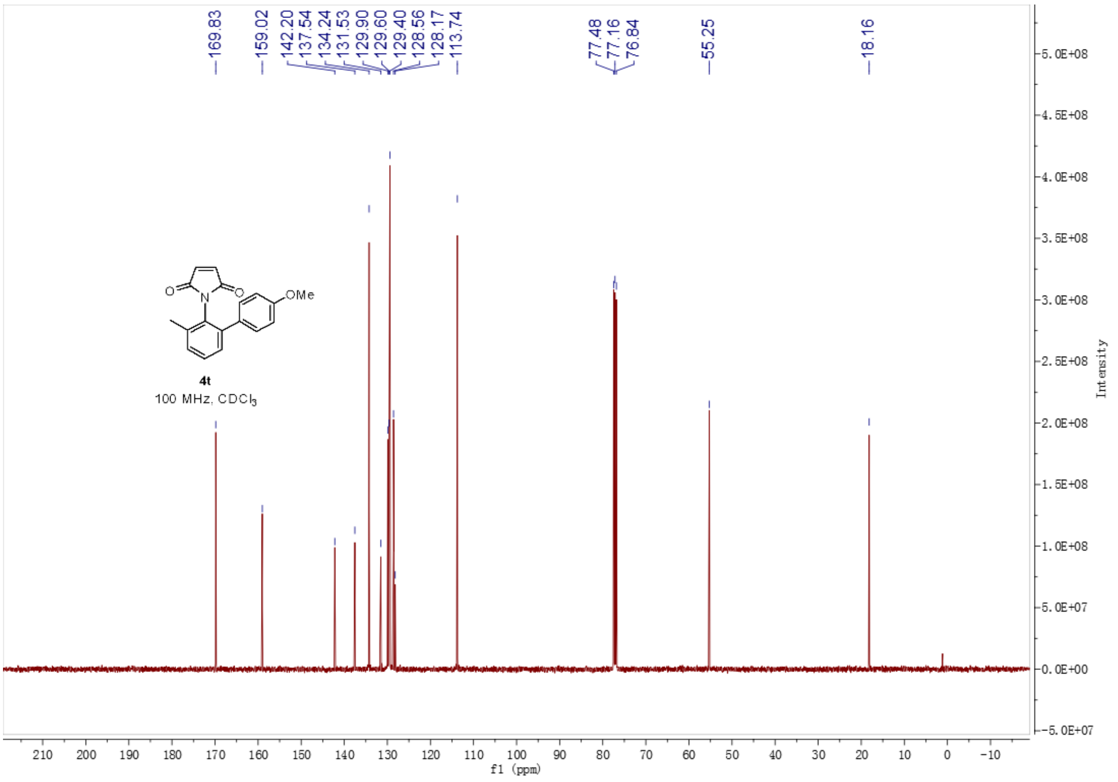

Supplementary Figure 65. ^{13}C NMR spectrum for **4t**

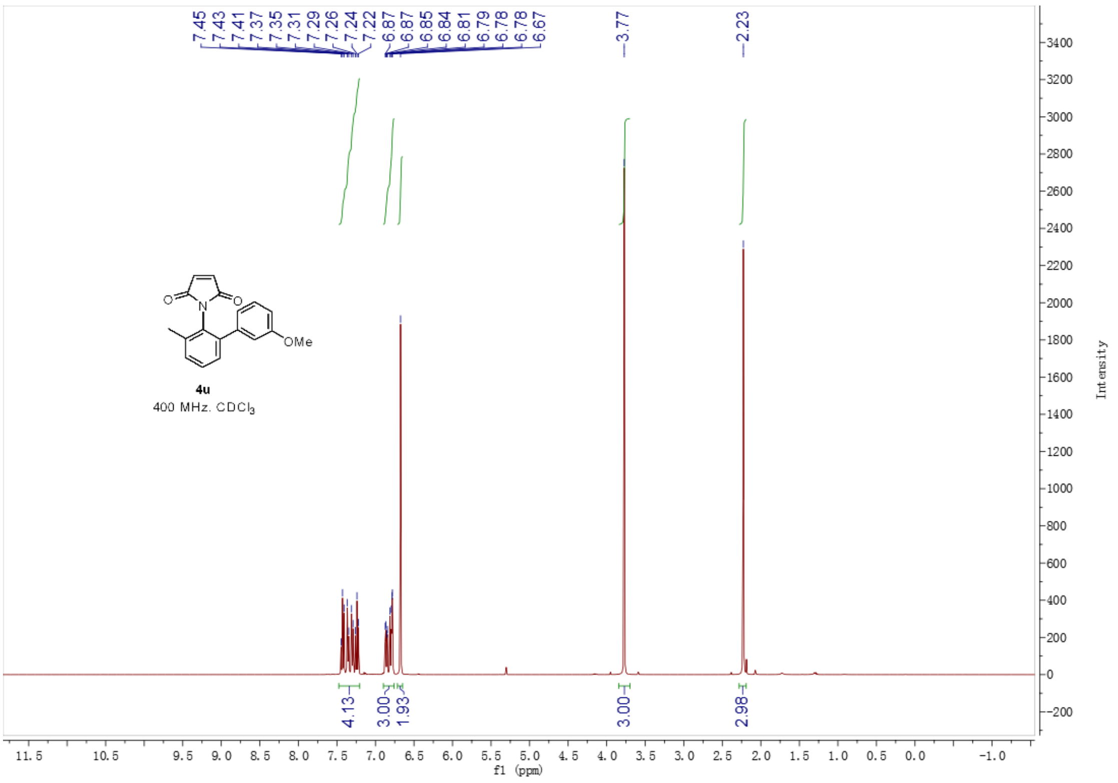

Supplementary Figure 66. ^{1}H NMR spectrum for **4u**

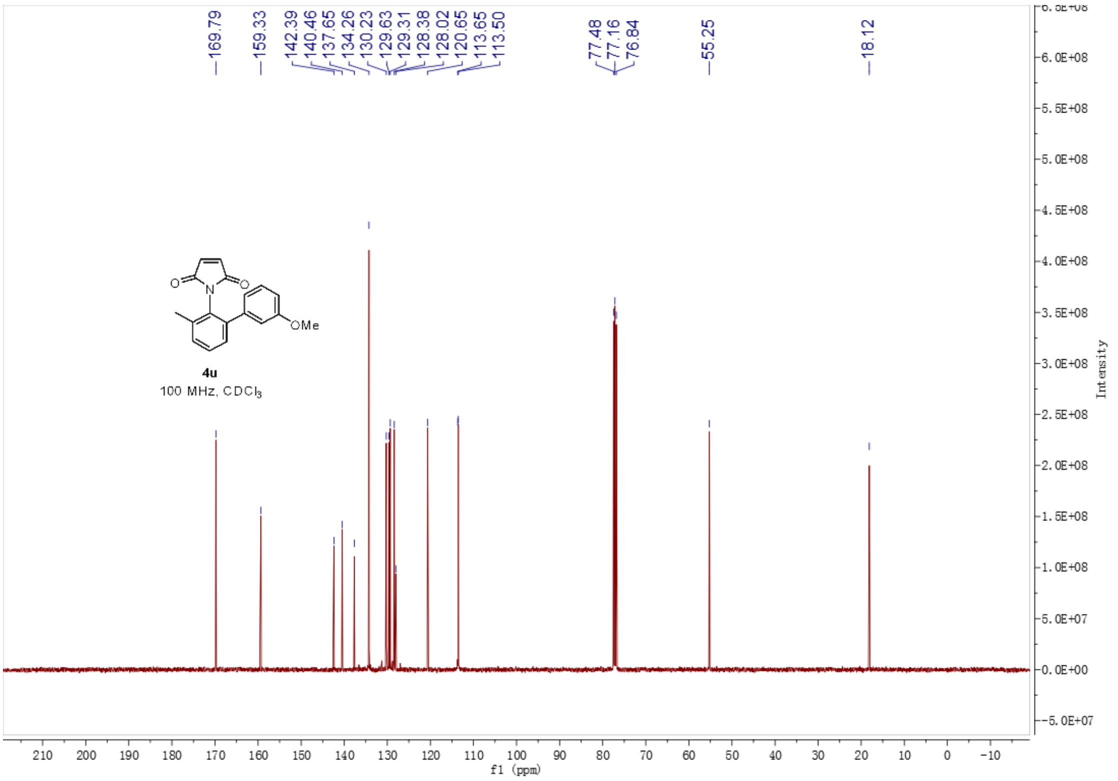

Supplementary Figure 67. ^{13}C NMR spectrum for **4u**

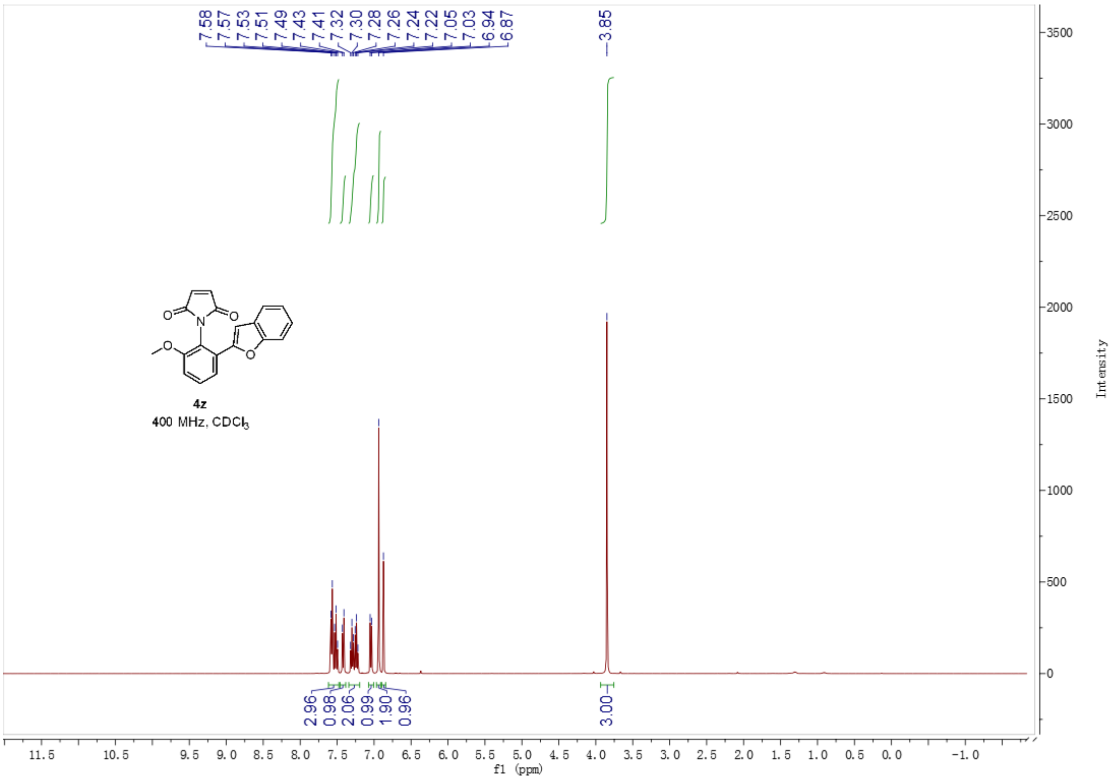

Supplementary Figure 68. ^{1}H NMR spectrum for **4z**

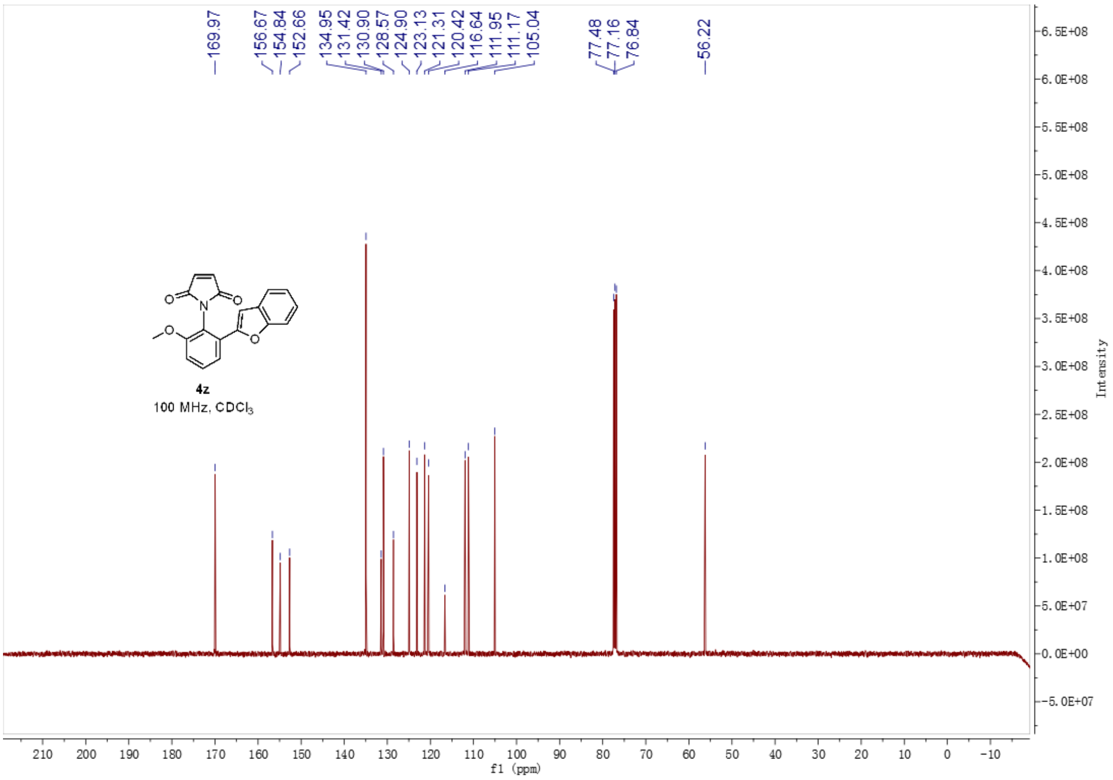

Supplementary Figure 69. ^{13}C NMR spectrum for **4z**

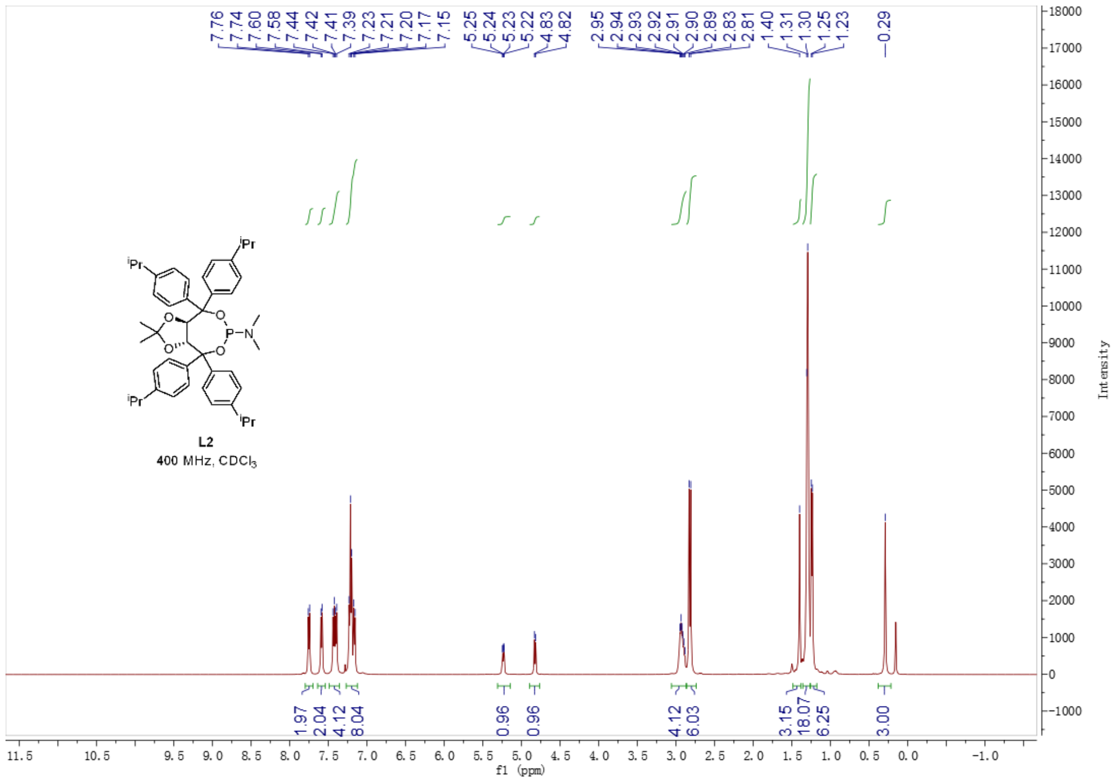

Supplementary Figure 70. ^{1}H NMR spectrum for **L2**

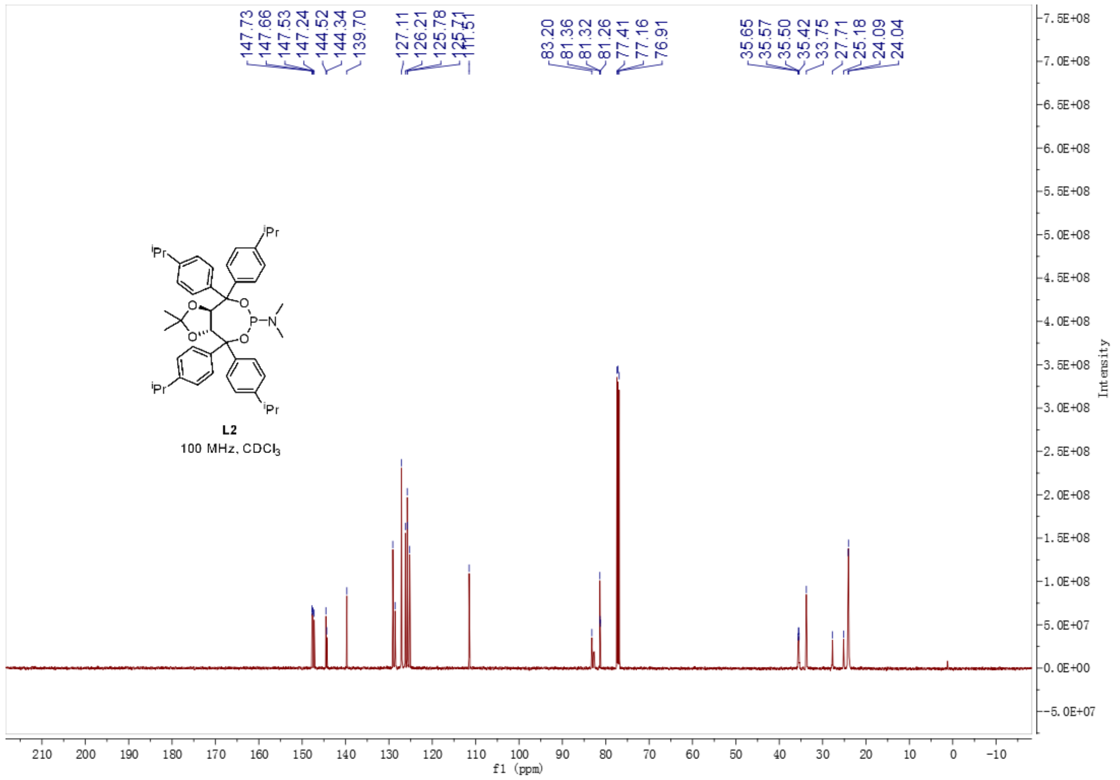

Supplementary Figure 71. ^{13}C NMR spectrum for **L2**

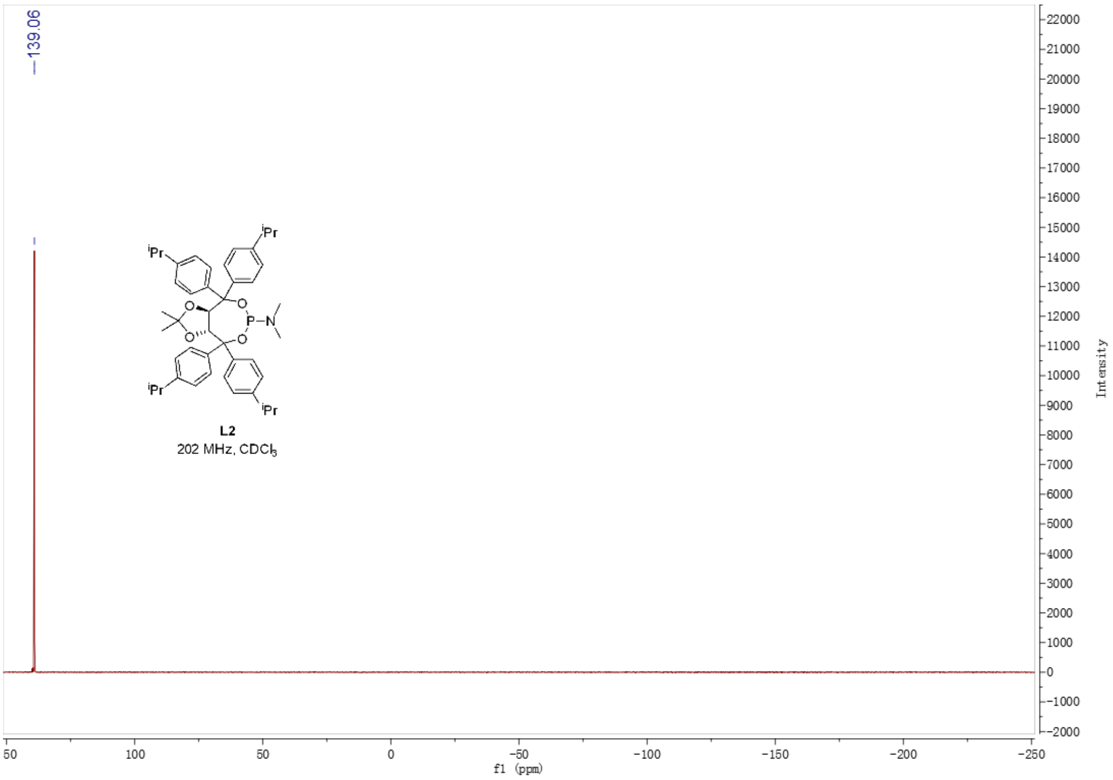

Supplementary Figure 72. ^{39}P NMR spectrum for **L2**

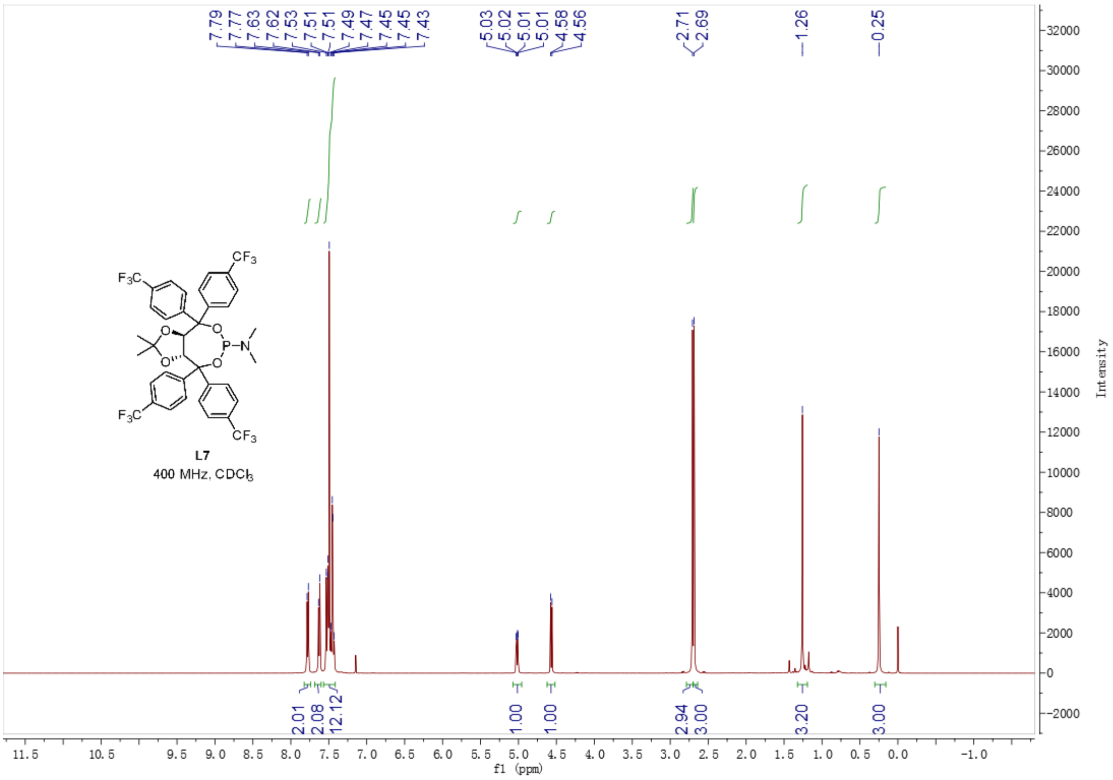

Supplementary Figure 73. ^{1}H NMR spectrum for **L7**

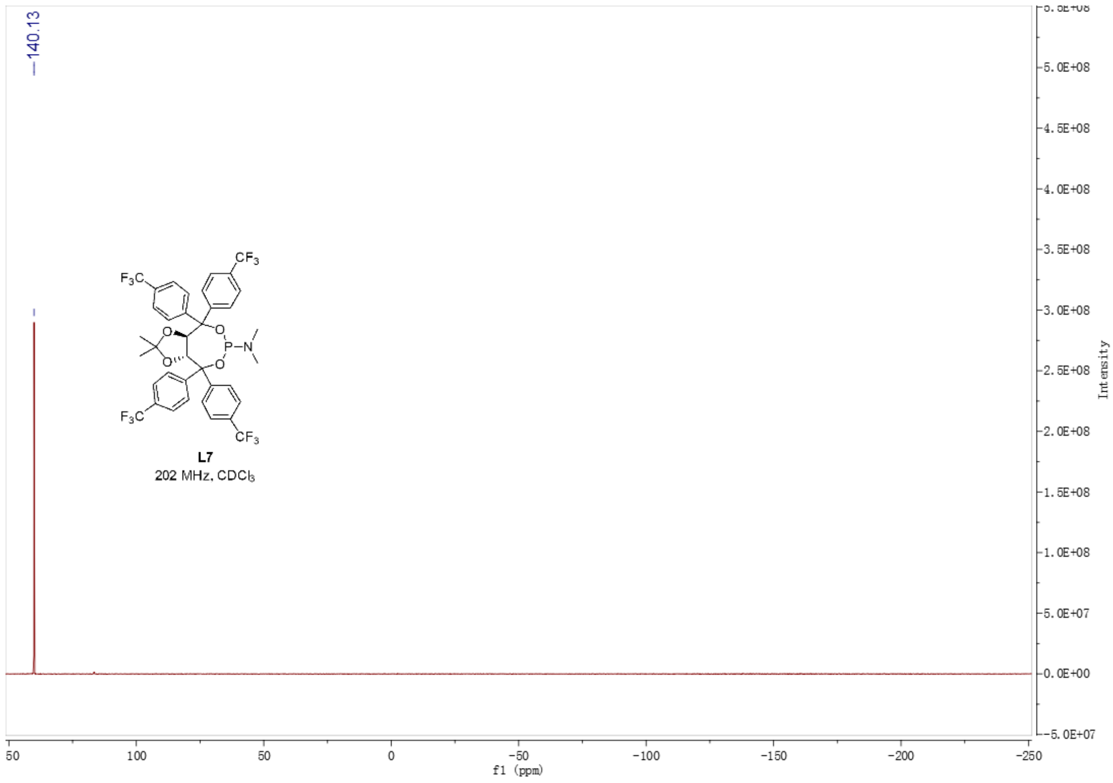

Supplementary Figure 74. ^{39}P NMR spectrum for **L7**

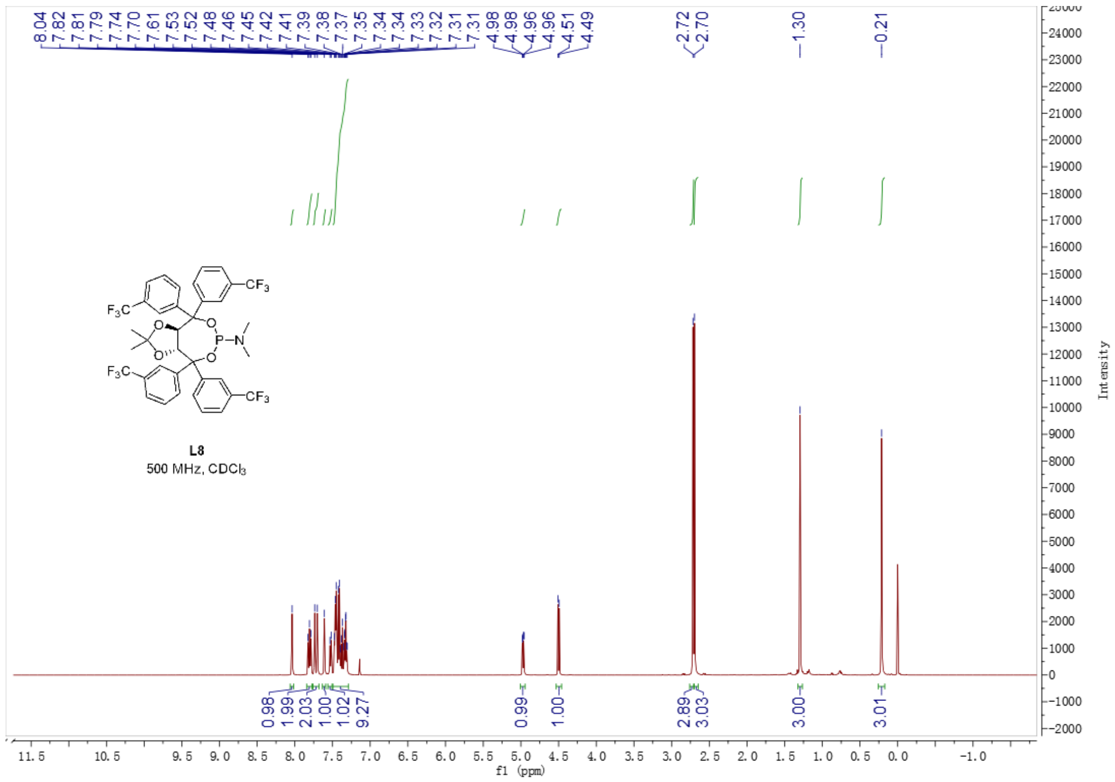

Supplementary Figure 75. ^{1}H NMR spectrum for **L8**

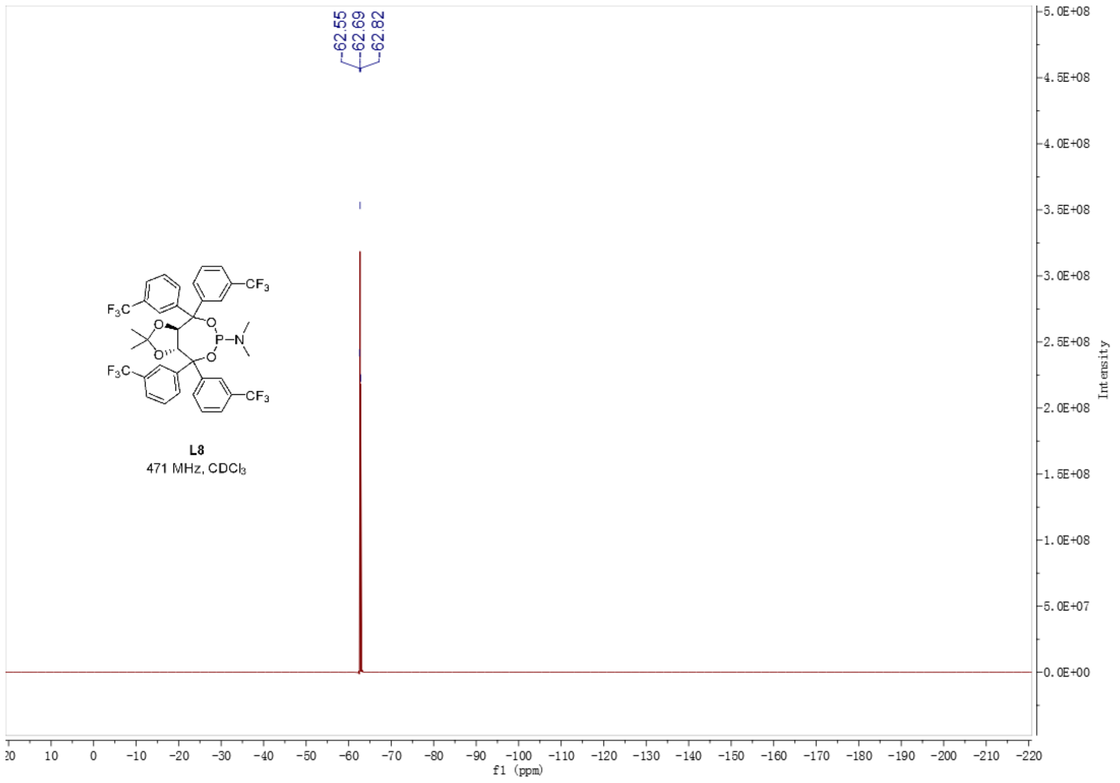

Supplementary Figure 76. ^{19}F NMR spectrum for **L8**

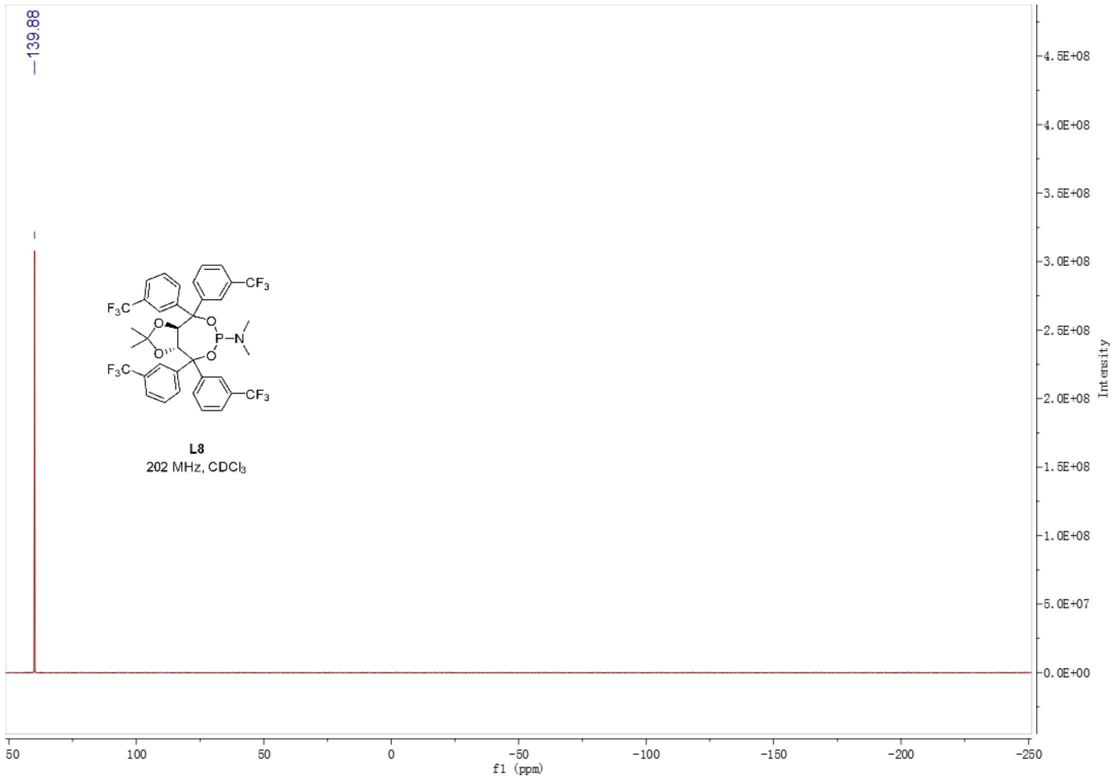

Supplementary Figure 77. ^{39}P NMR spectrum for **L8**

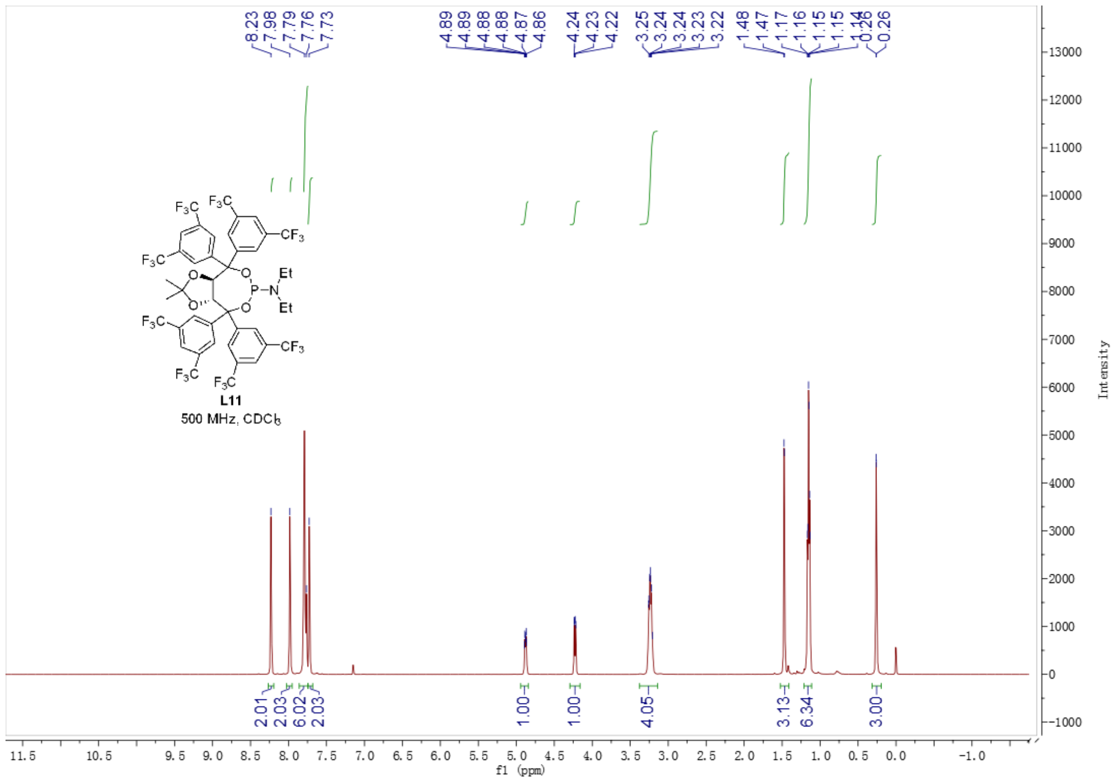

Supplementary Figure 78. ^{1}H NMR spectrum for **L11**

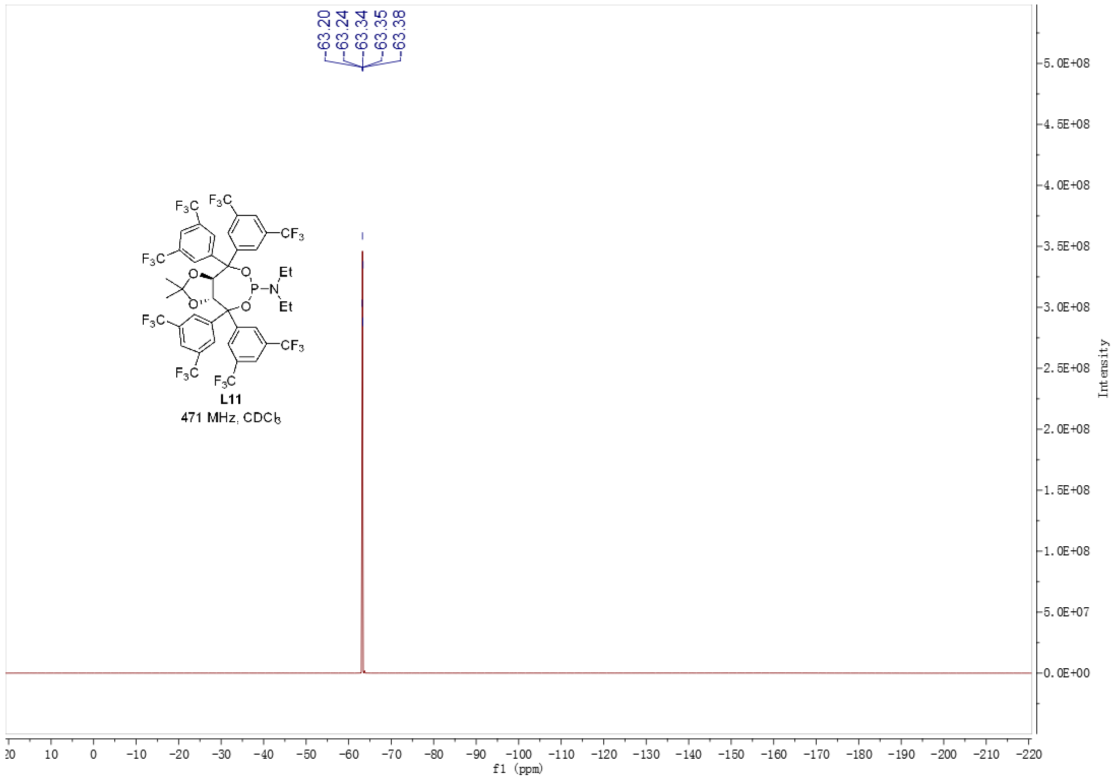

Supplementary Figure 79. ^{19}F NMR spectrum for **L11**

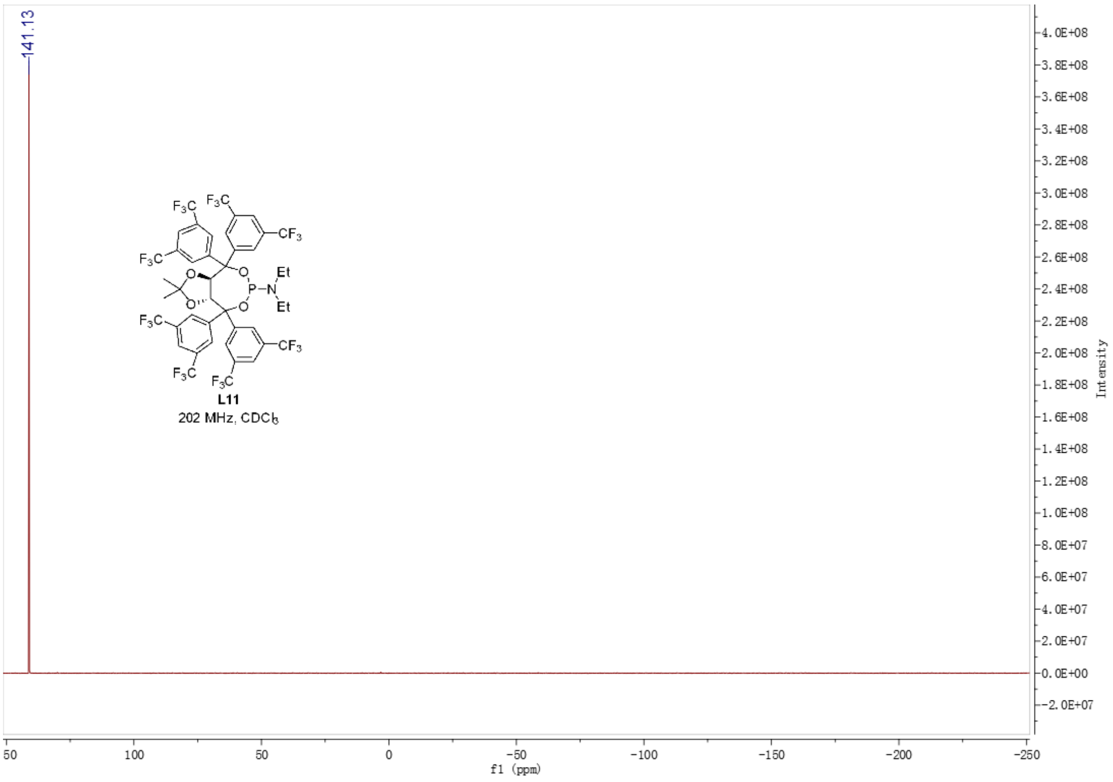

Supplementary Figure 80. ^{39}P NMR spectrum for **L11**

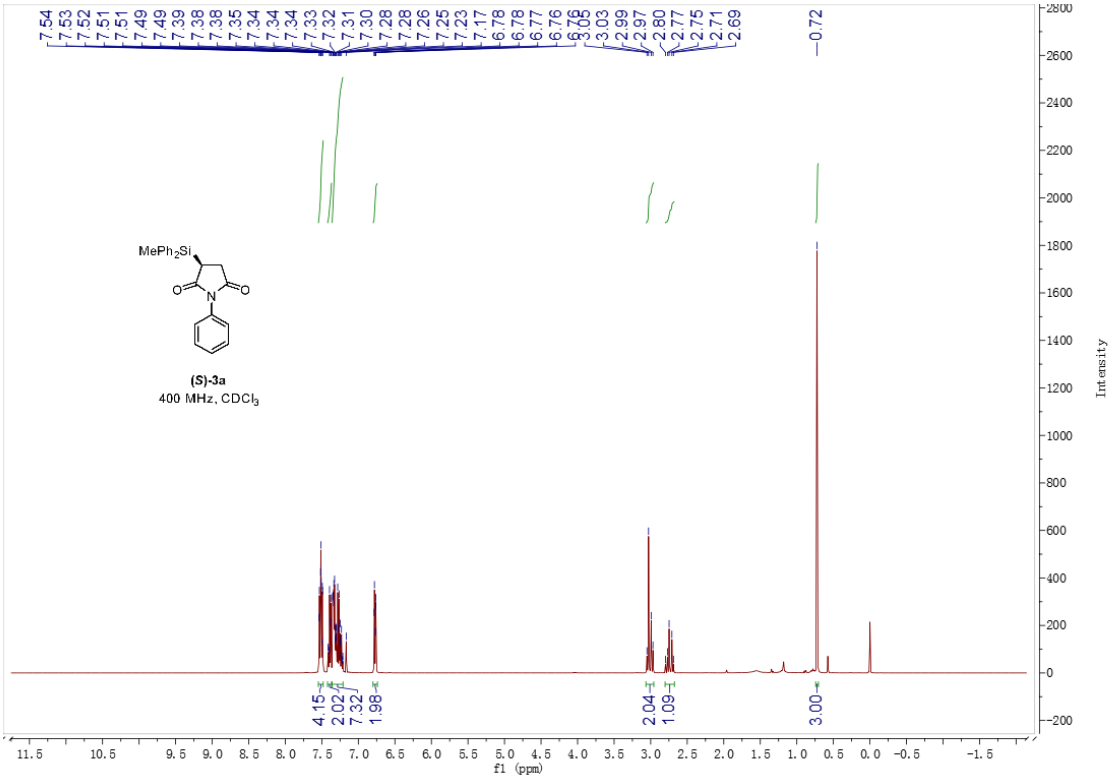

Supplementary Figure 81. ^{1}H NMR spectrum for **3a**

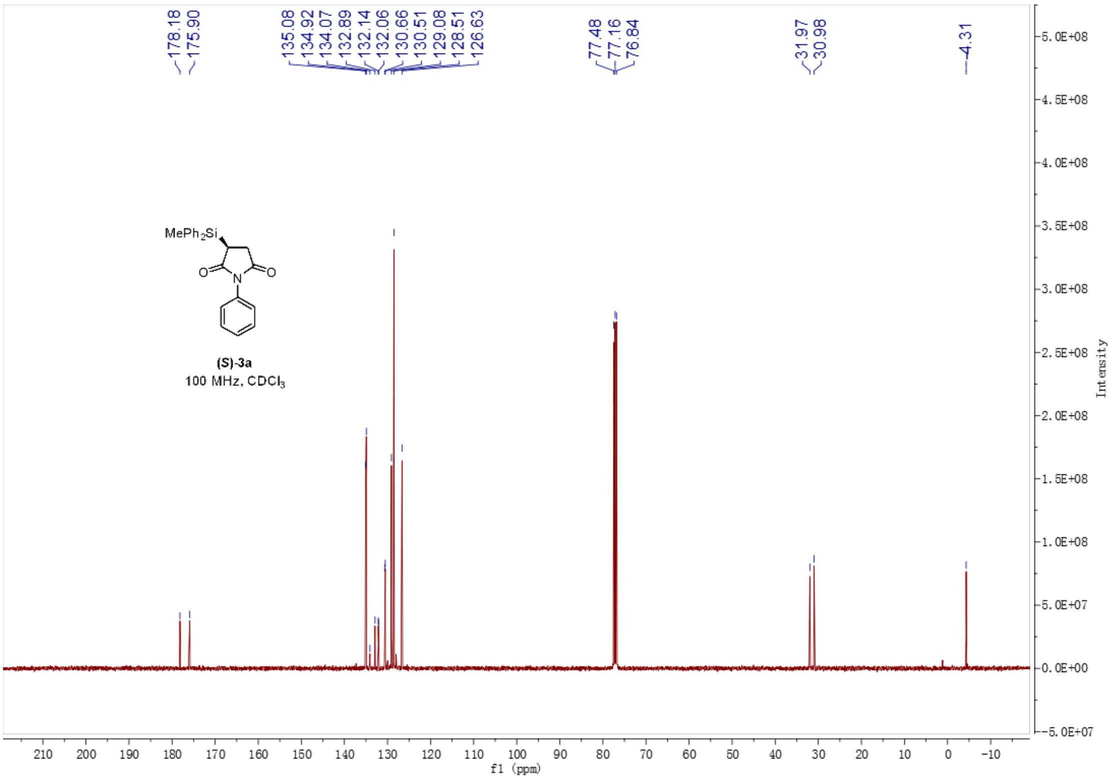

Supplementary Figure 82. ^{13}C NMR spectrum for **3a**

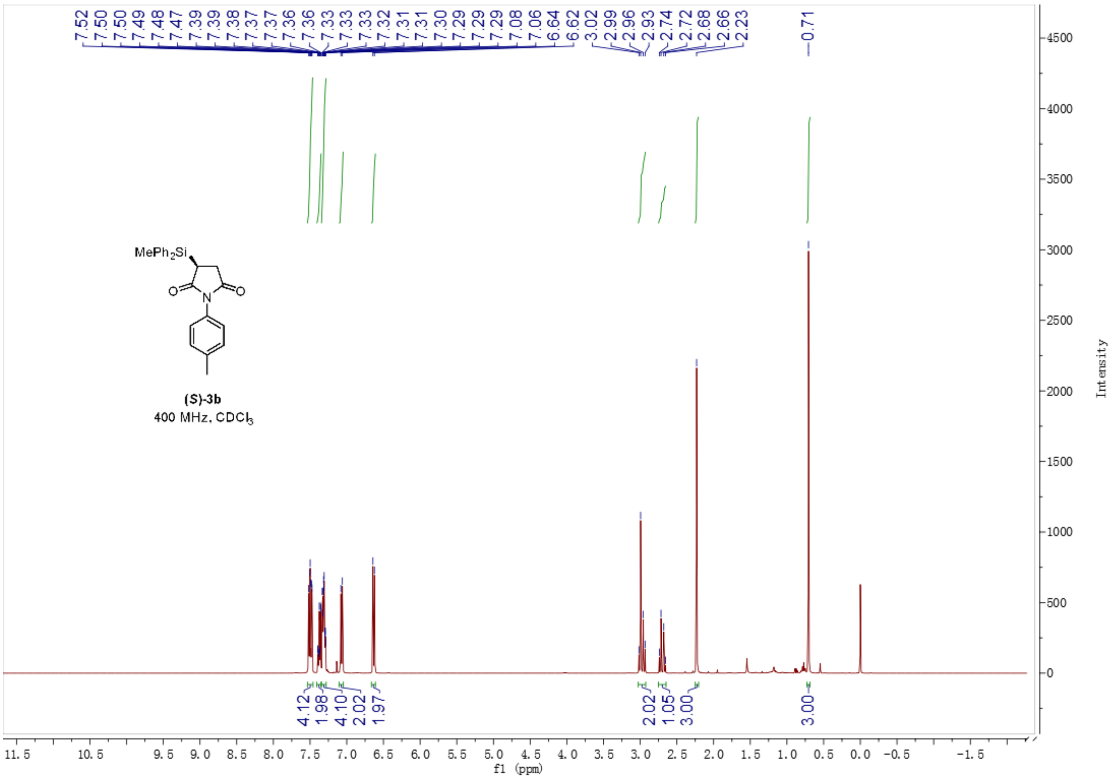

Supplementary Figure 83. ^{1}H NMR spectrum for **3b**

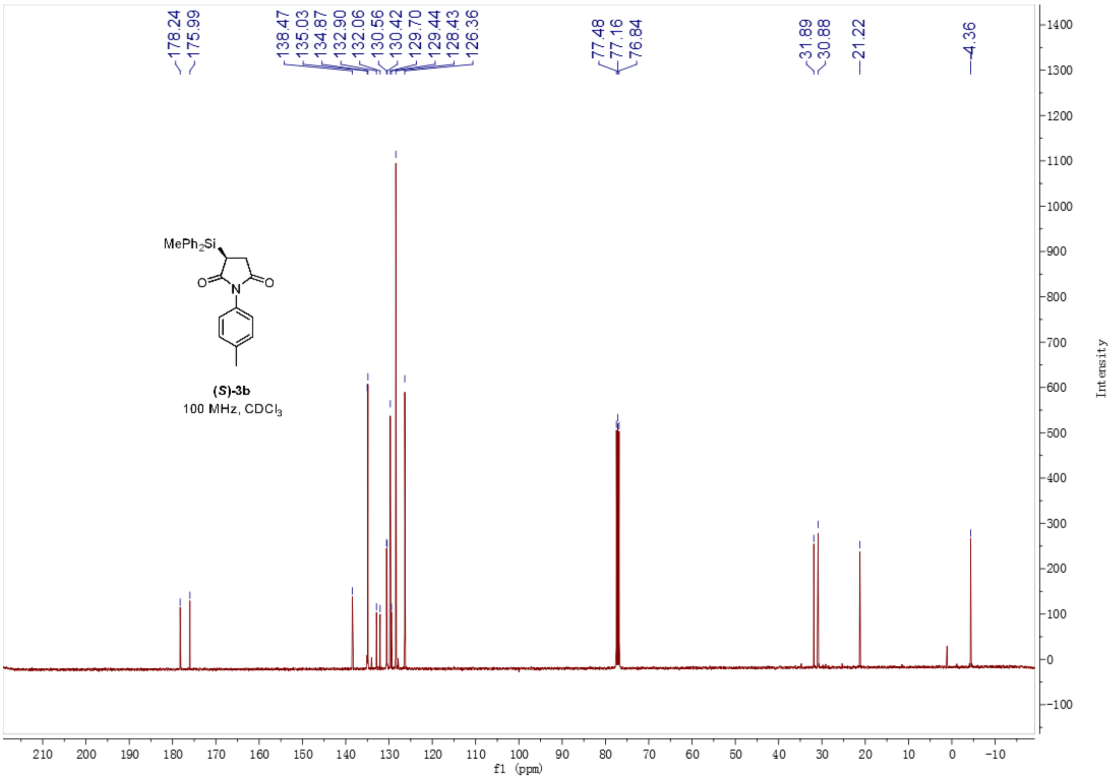

Supplementary Figure 84. ^{13}C NMR spectrum for **3b**

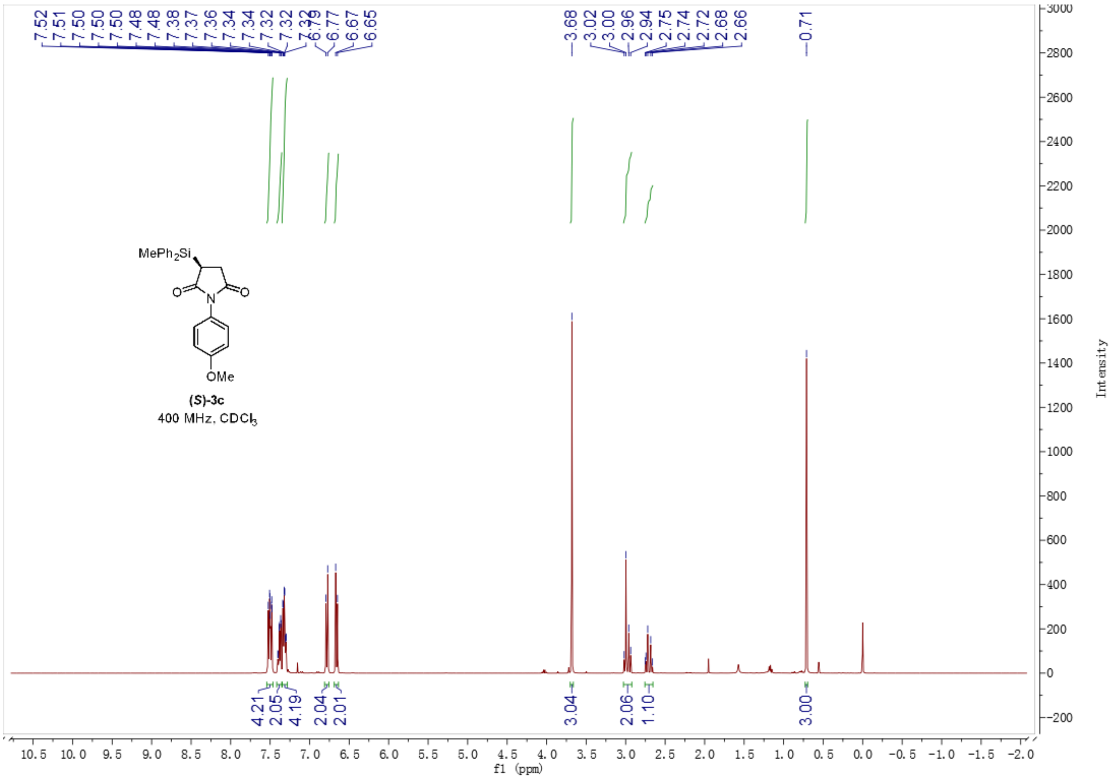

Supplementary Figure 85. ^{1}H NMR spectrum for **3c**

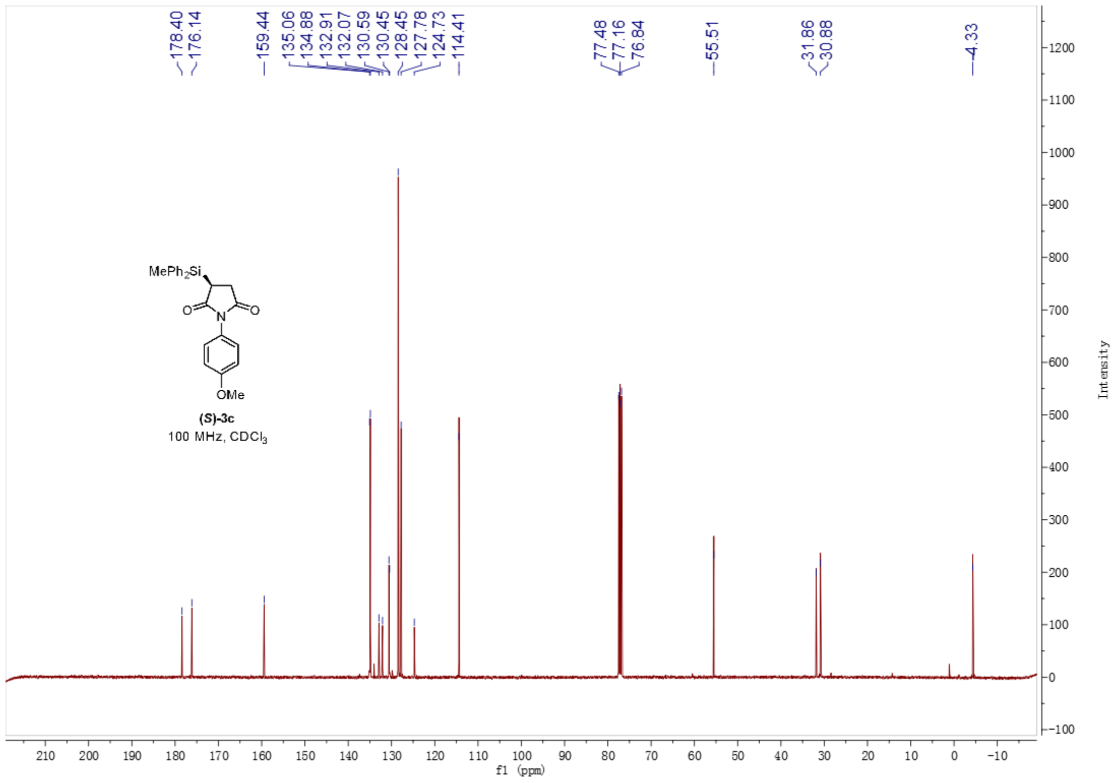

Supplementary Figure 86. ^{13}C NMR spectrum for **3c**

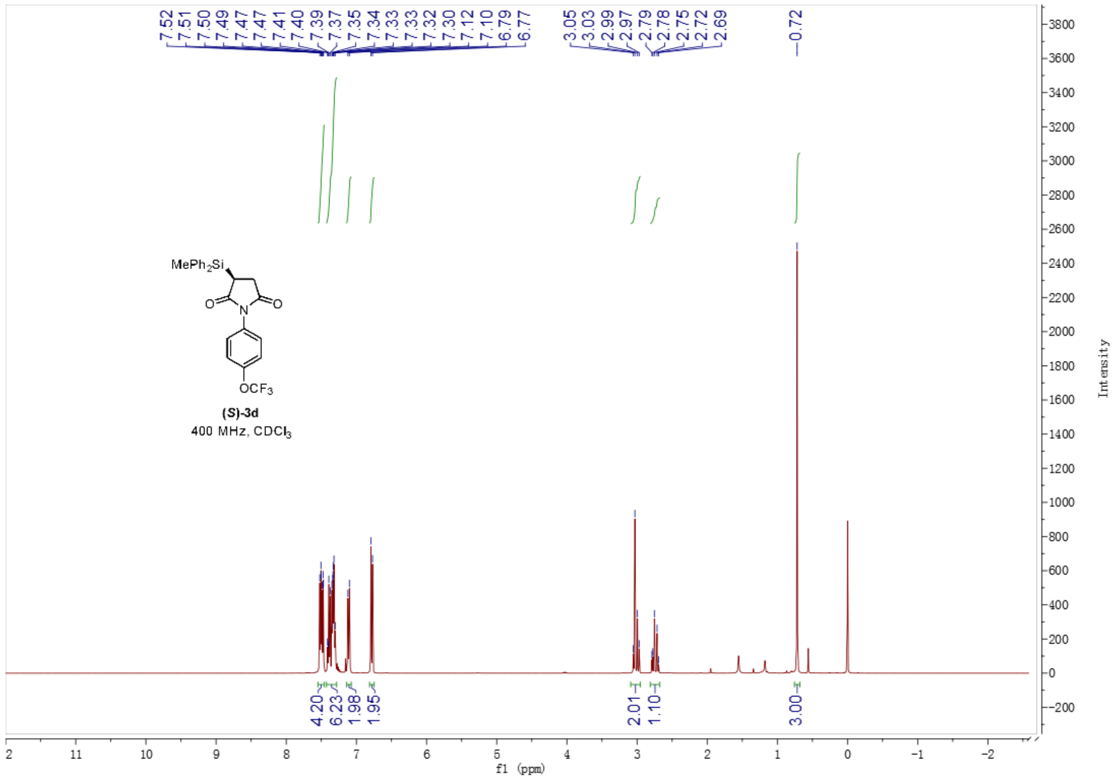

Supplementary Figure 87. ^{1}H NMR spectrum for **3d**

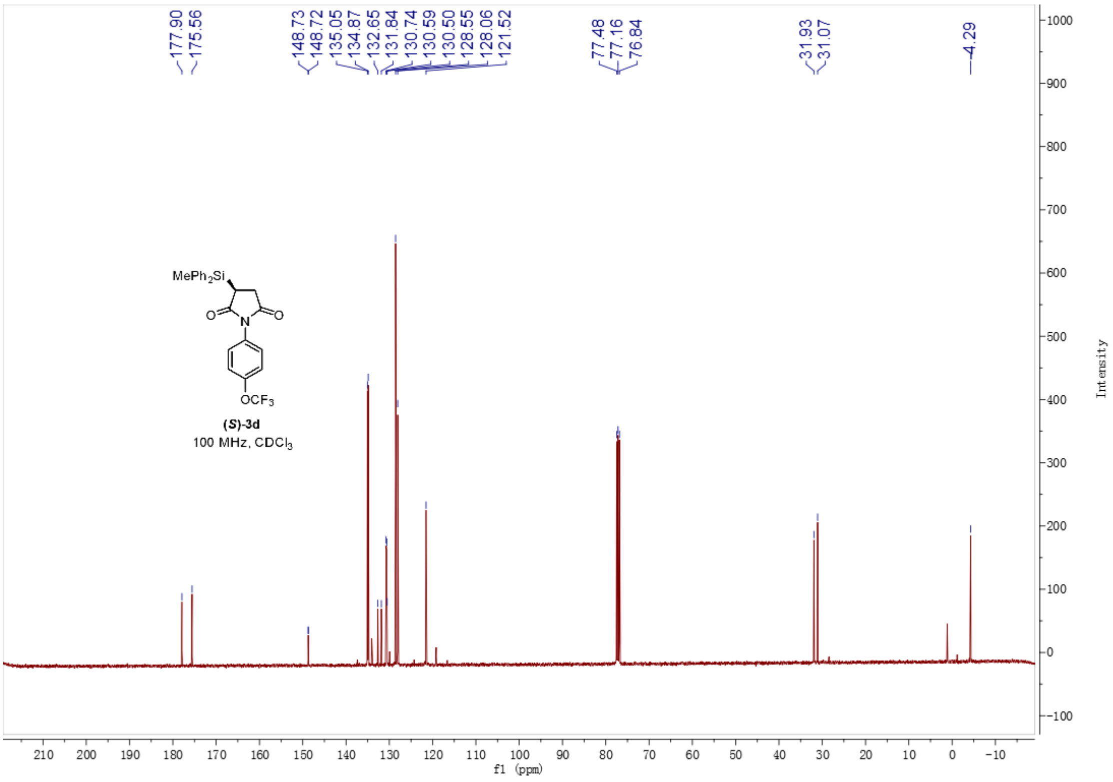

Supplementary Figure 88. ^{13}C NMR spectrum for **3d**

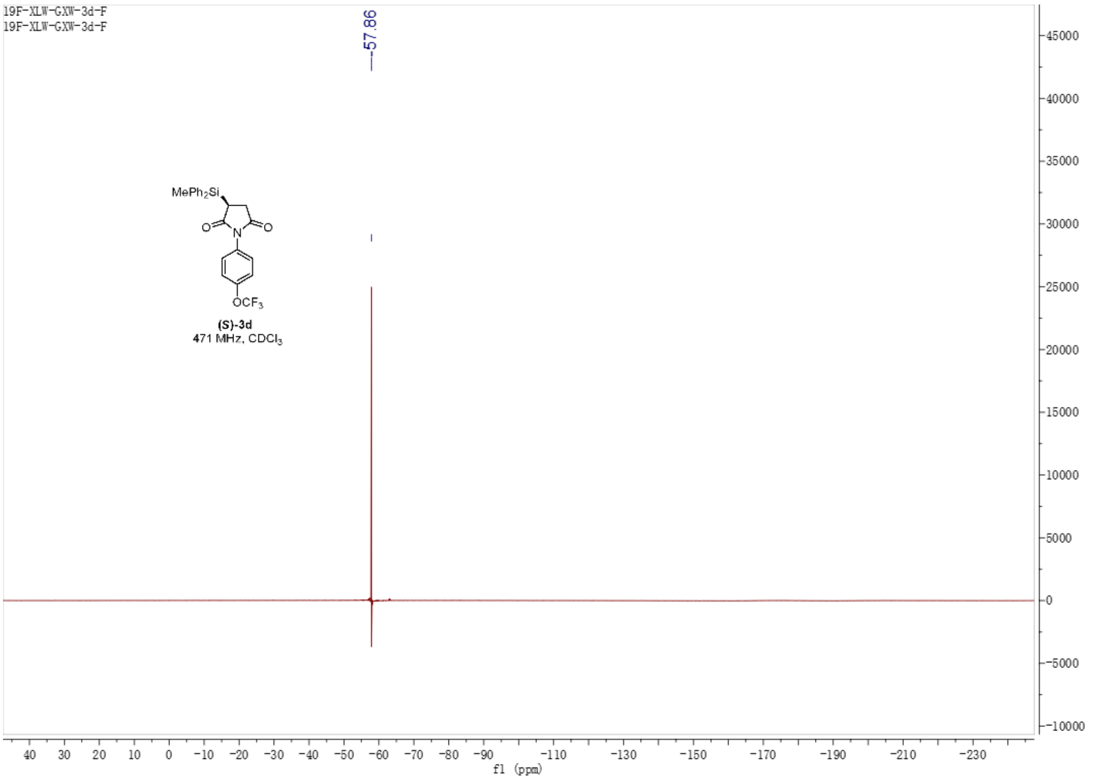

Supplementary Figure 89. ^{19}F NMR spectrum for **3d**

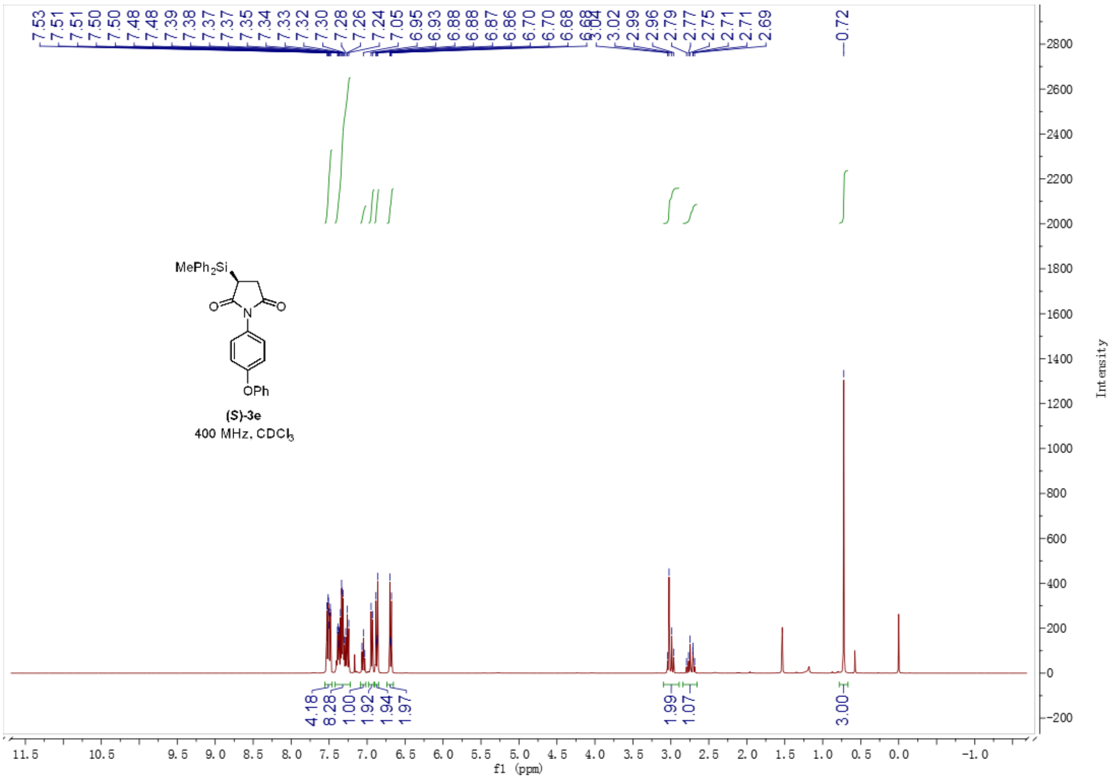

Supplementary Figure 90. ^{1}H NMR spectrum for **3e**

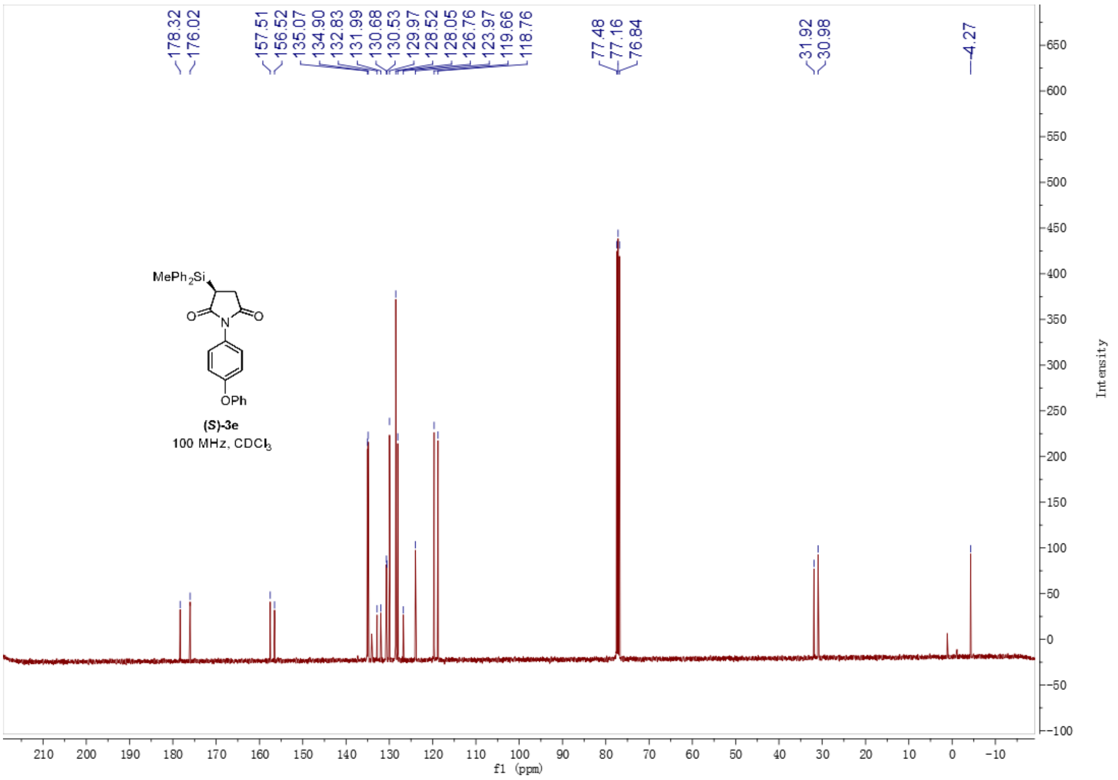

Supplementary Figure 91. ^{13}C NMR spectrum for **3e**

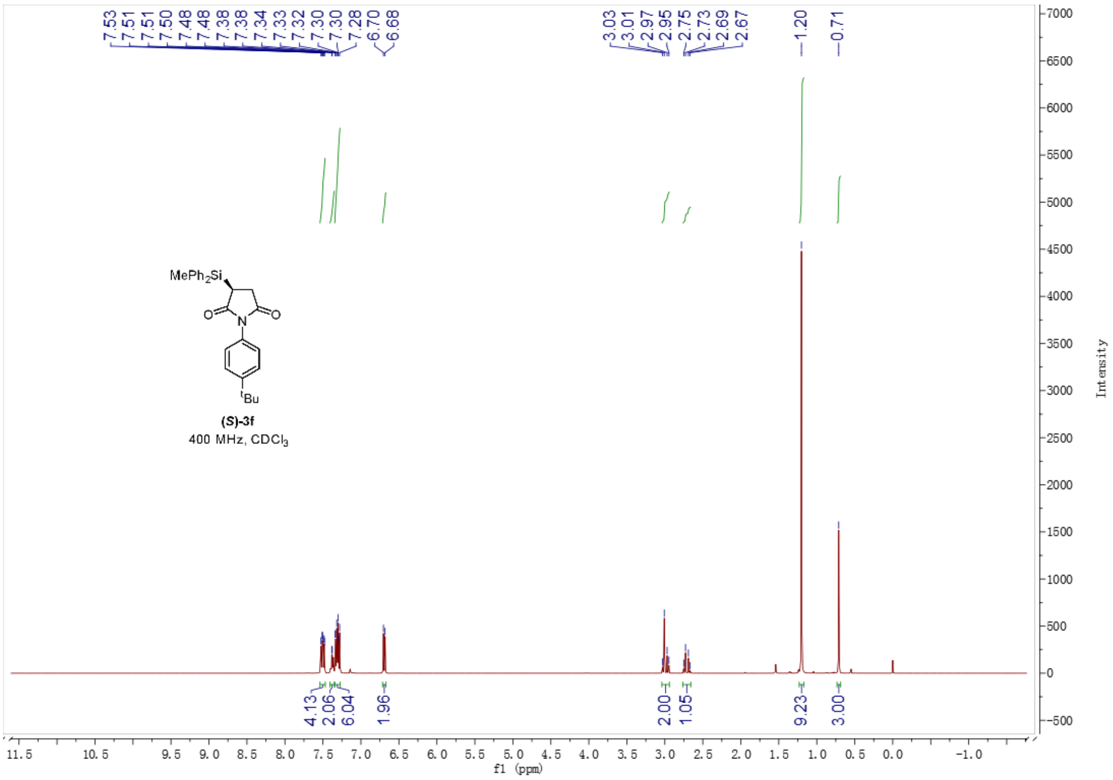

Supplementary Figure 92. ^{1}H NMR spectrum for **3f**

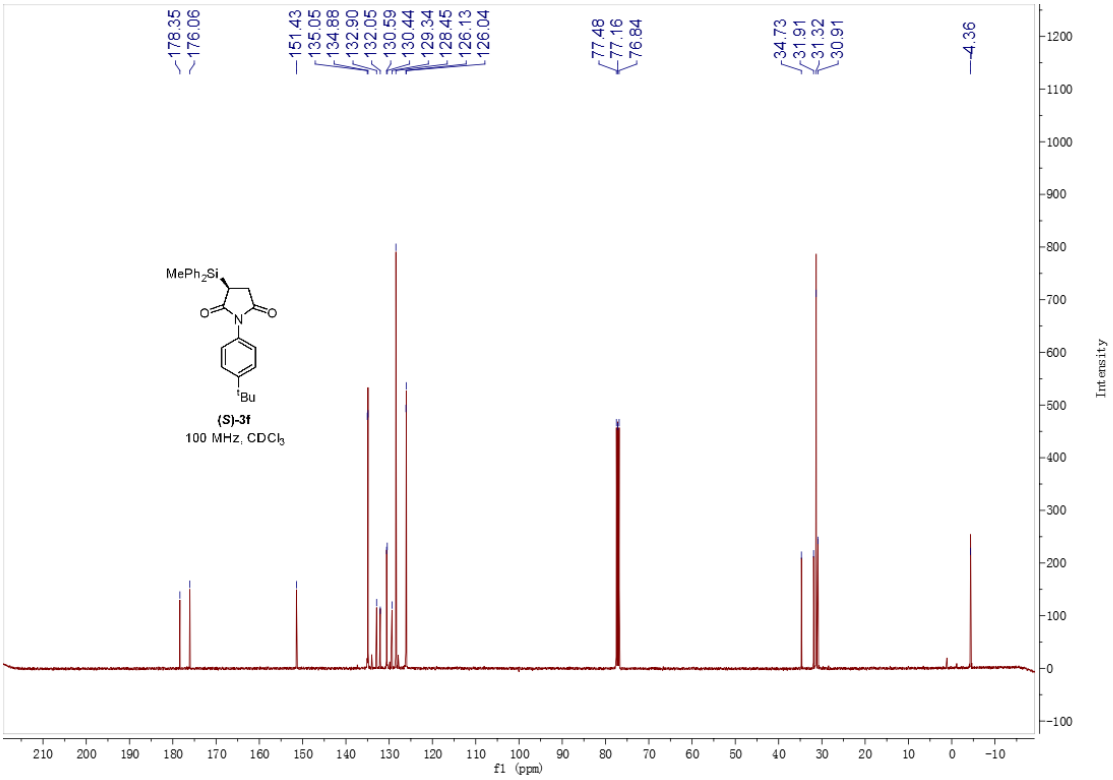

Supplementary Figure 93. ^{13}C NMR spectrum for **3f**

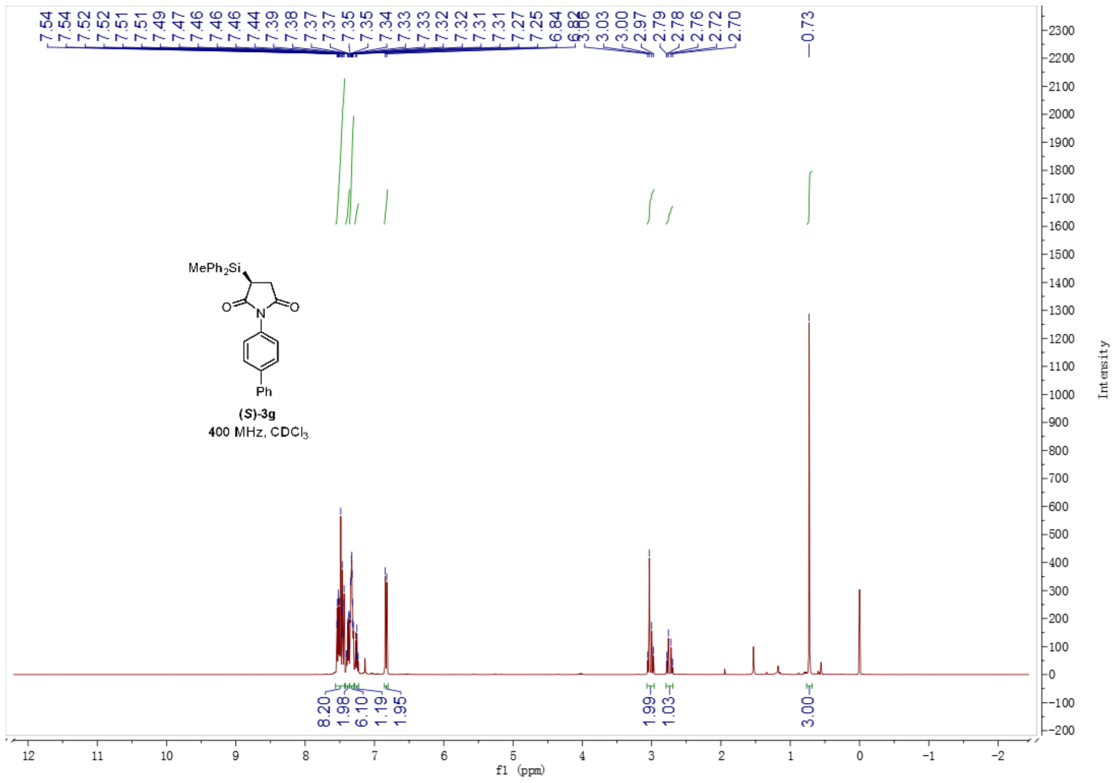

Supplementary Figure 94. ^{1}H NMR spectrum for **3g**

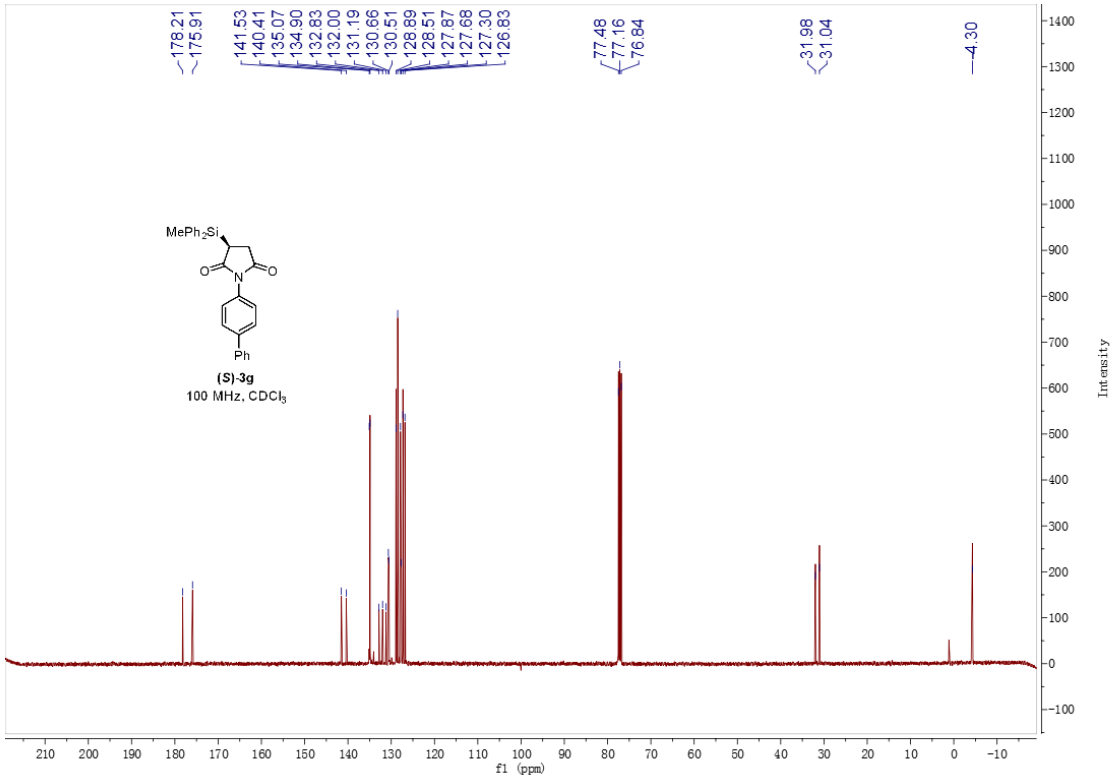

Supplementary Figure 95. ^{13}C NMR spectrum for **3g**

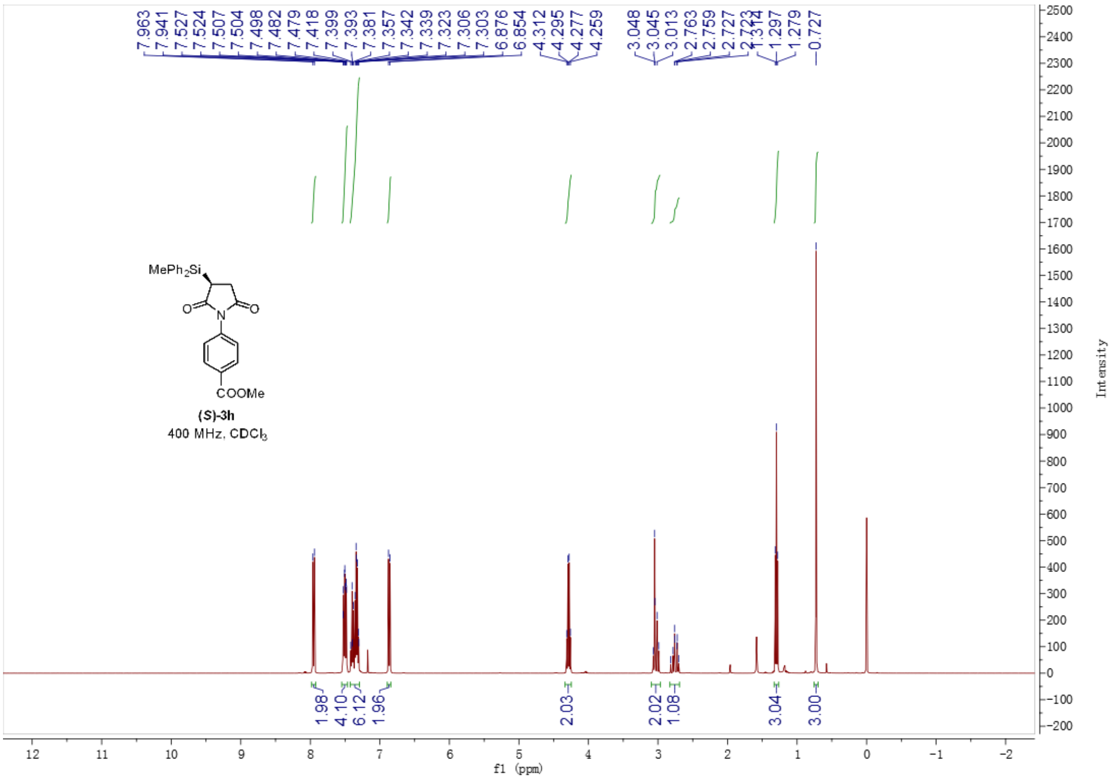

Supplementary Figure 96. ^{1}H NMR spectrum for **3h**

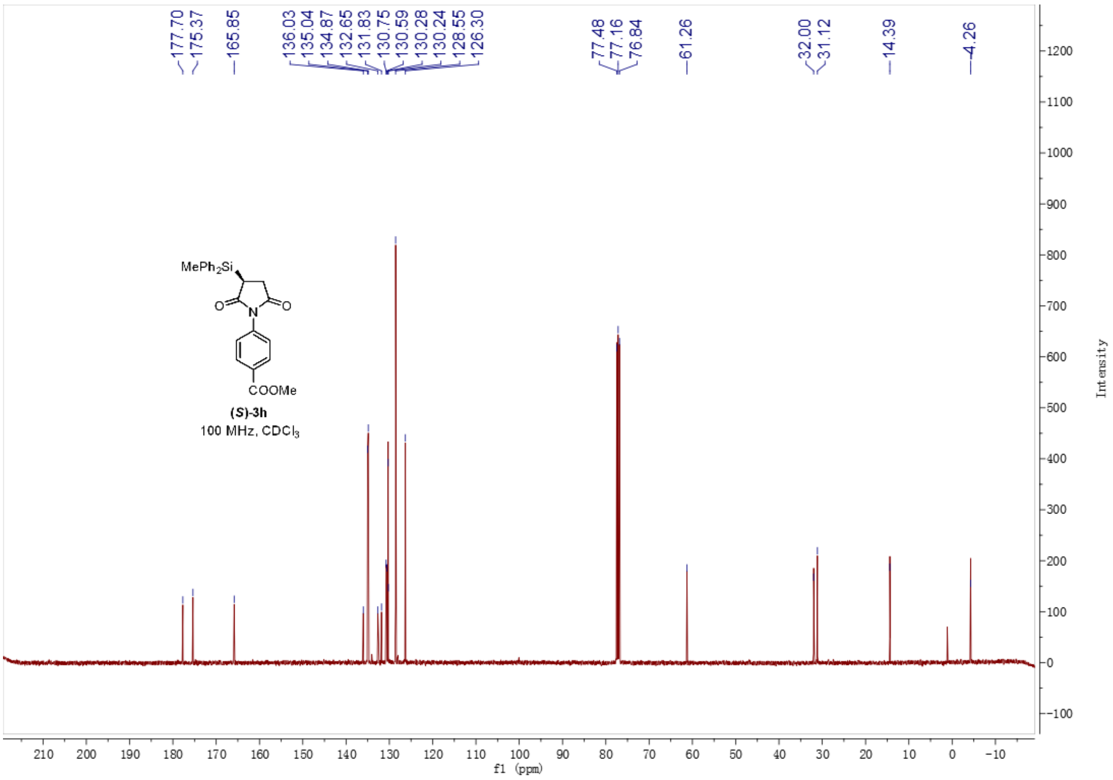

Supplementary Figure 97. ^{13}C NMR spectrum for **3h**

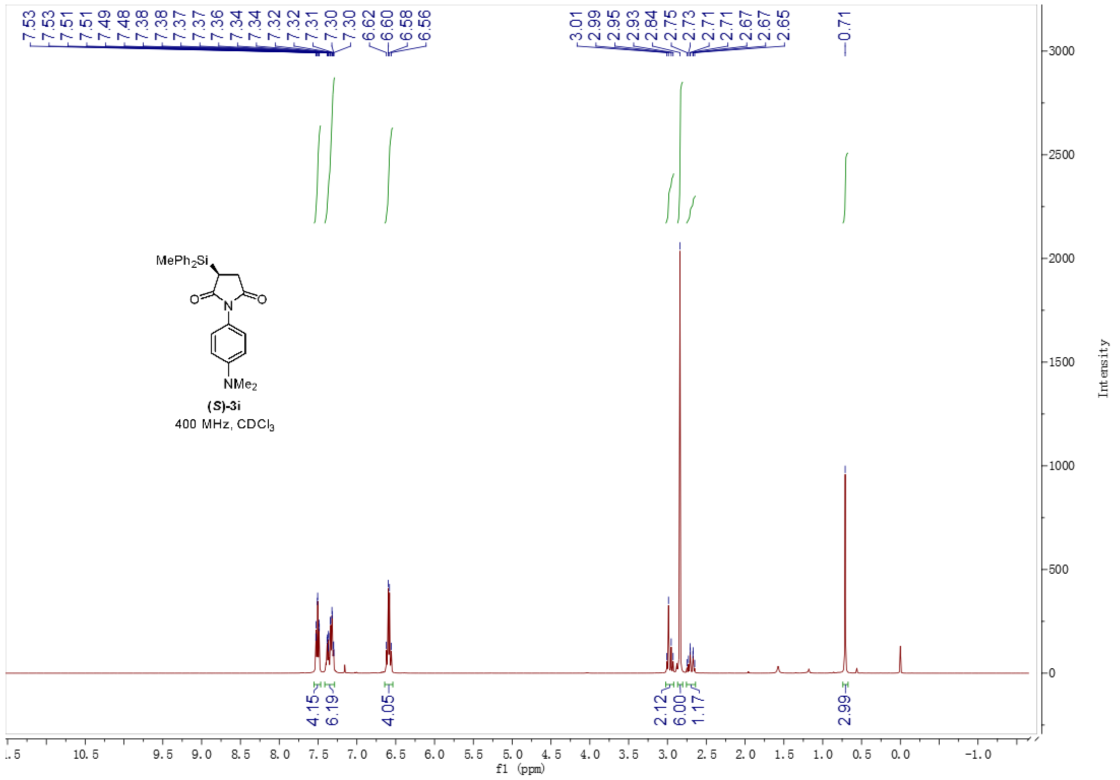

Supplementary Figure 98. ^{1}H NMR spectrum for **3i**

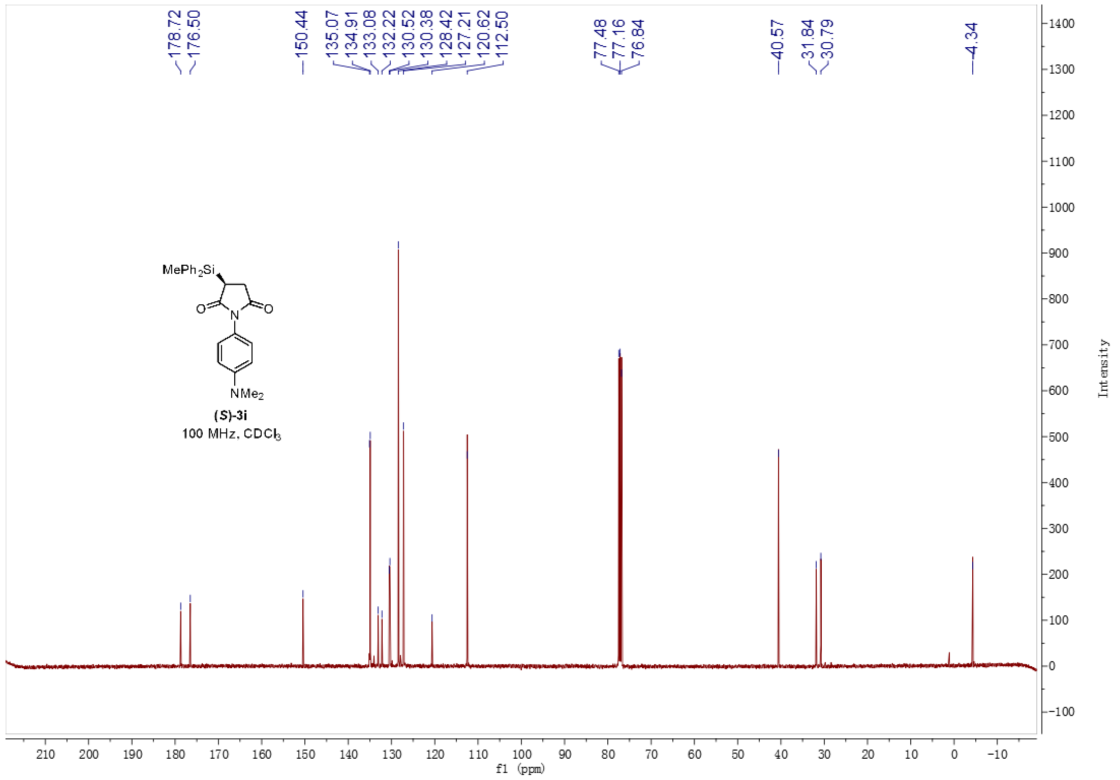

Supplementary Figure 99. ^{13}C NMR spectrum for **3i**

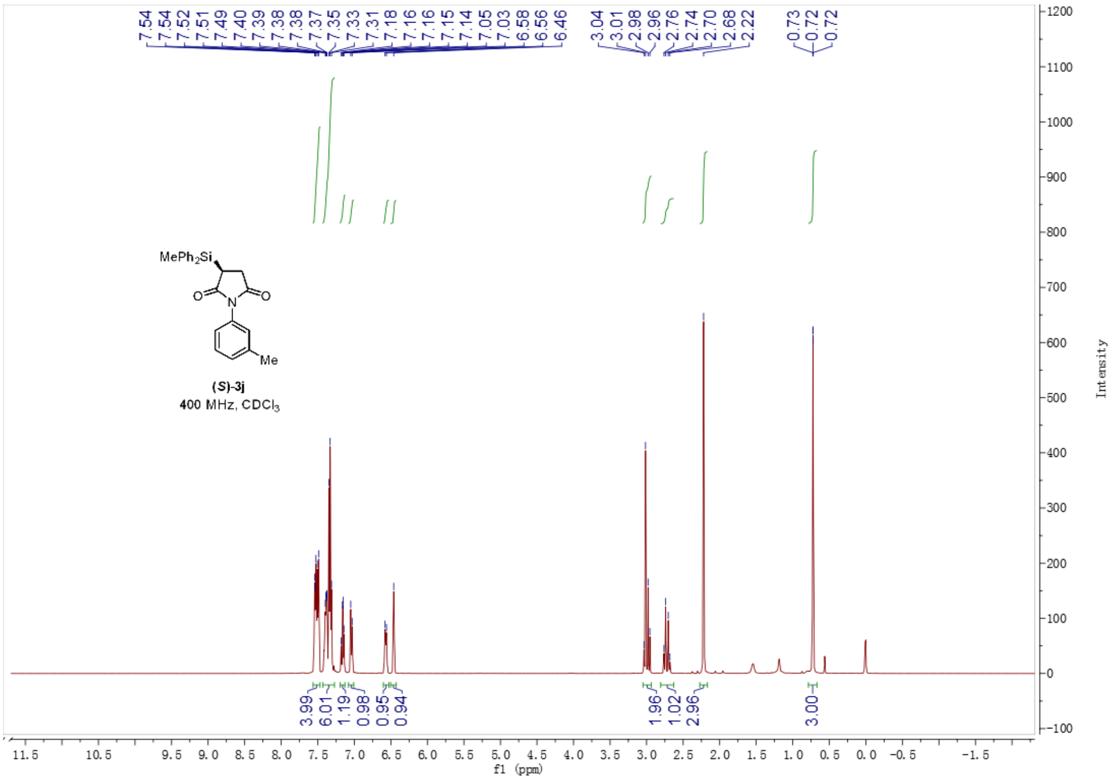

Supplementary Figure 100. ^{1}H NMR spectrum for **3j**

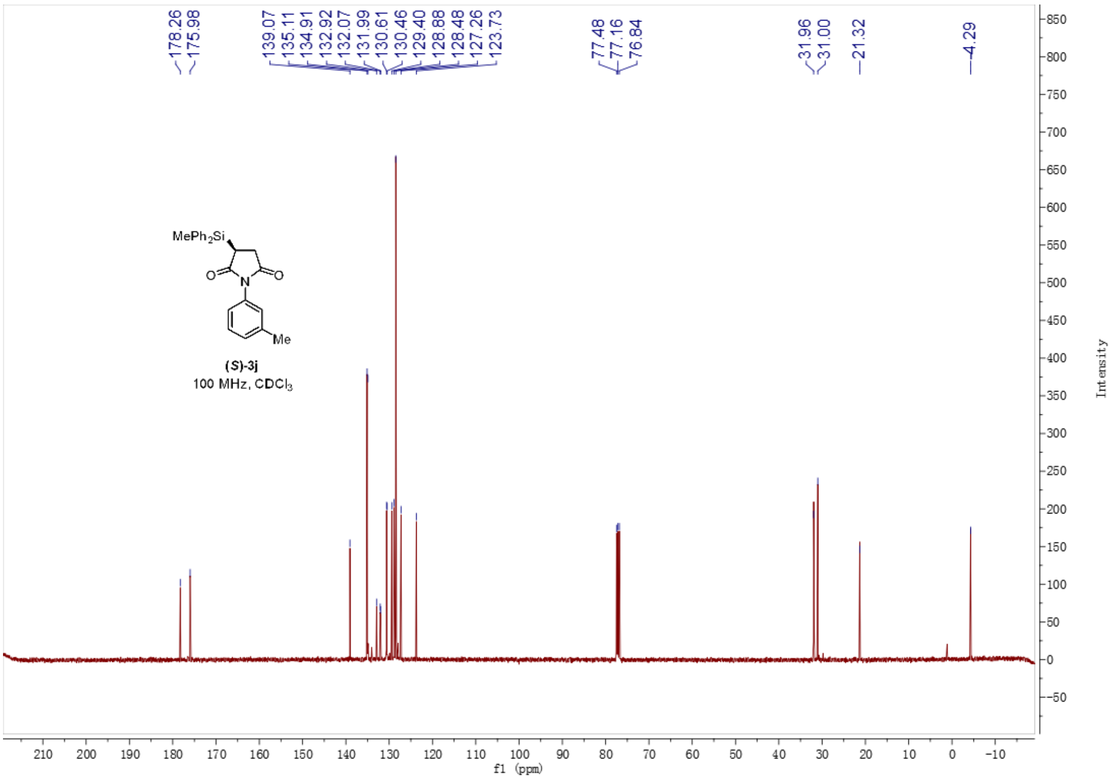

Supplementary Figure 101. ^{13}C NMR spectrum for **3j**

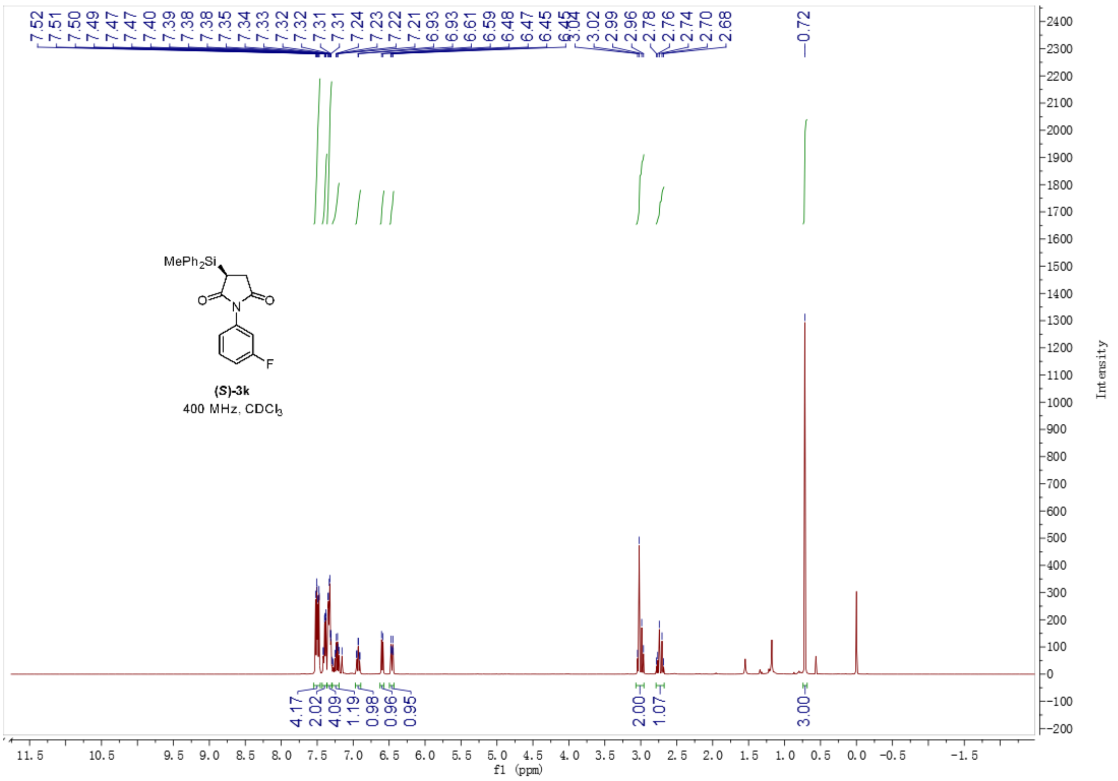

Supplementary Figure 102. ^{1}H NMR spectrum for **3k**

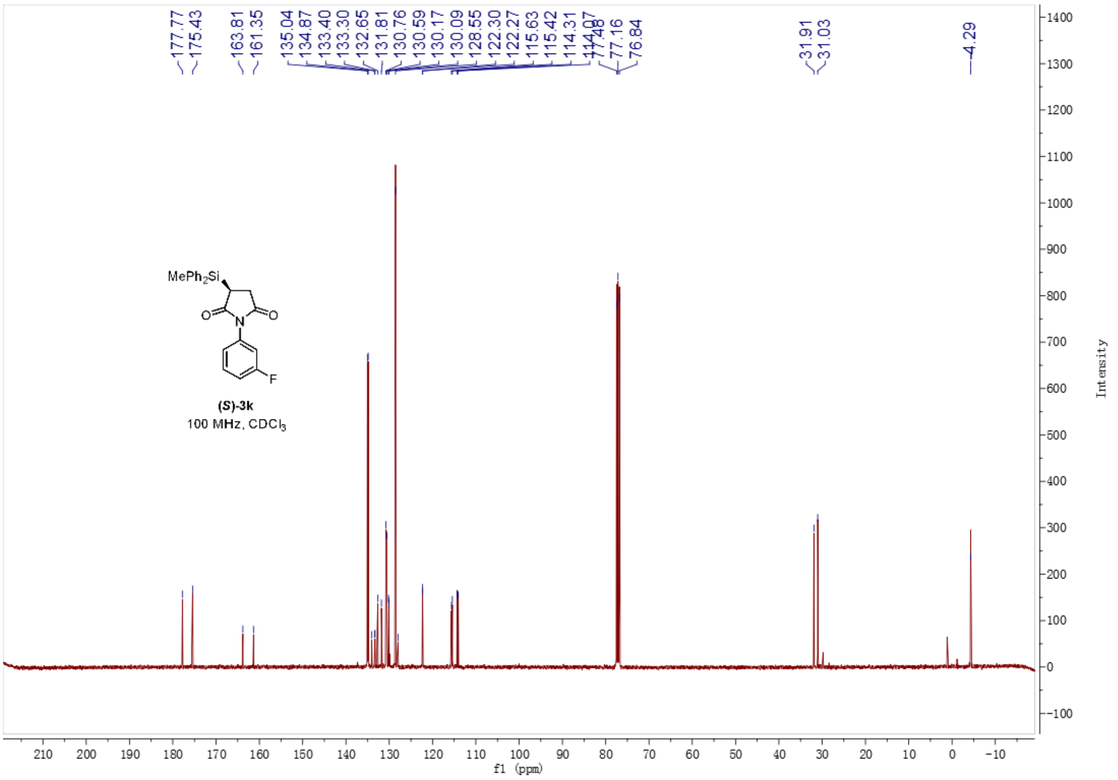

Supplementary Figure 103. ^{13}C NMR spectrum for **3k**

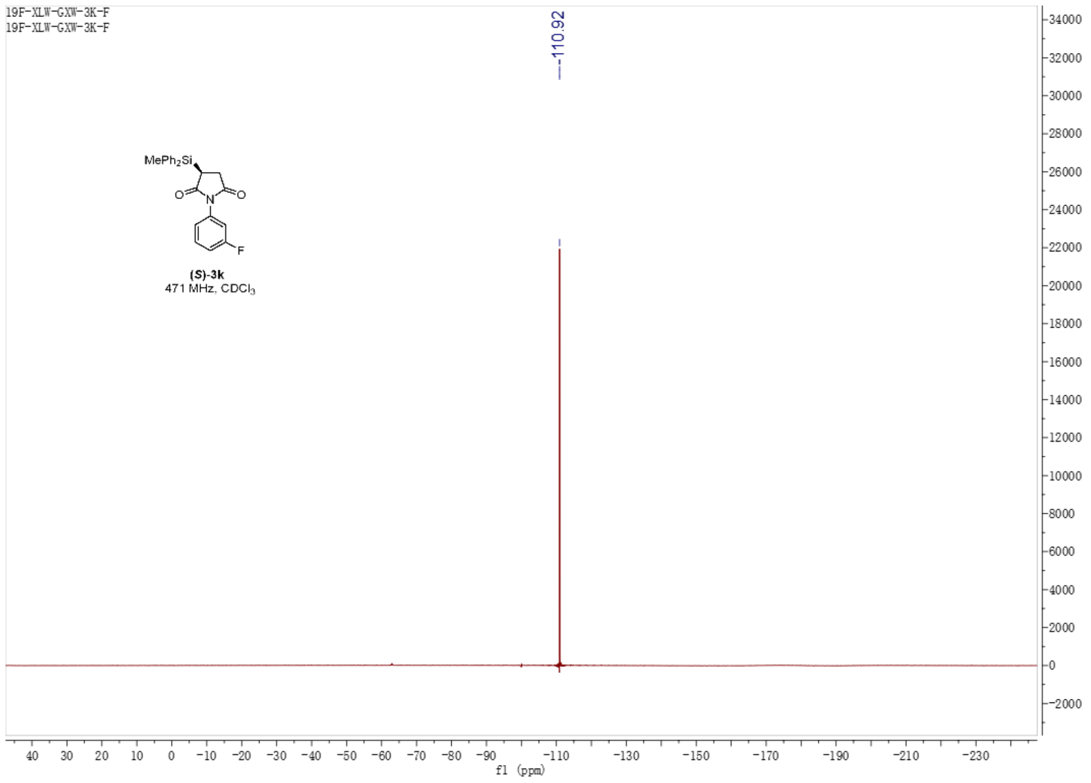

Supplementary Figure 104. ^{19}F NMR spectrum for **3k**

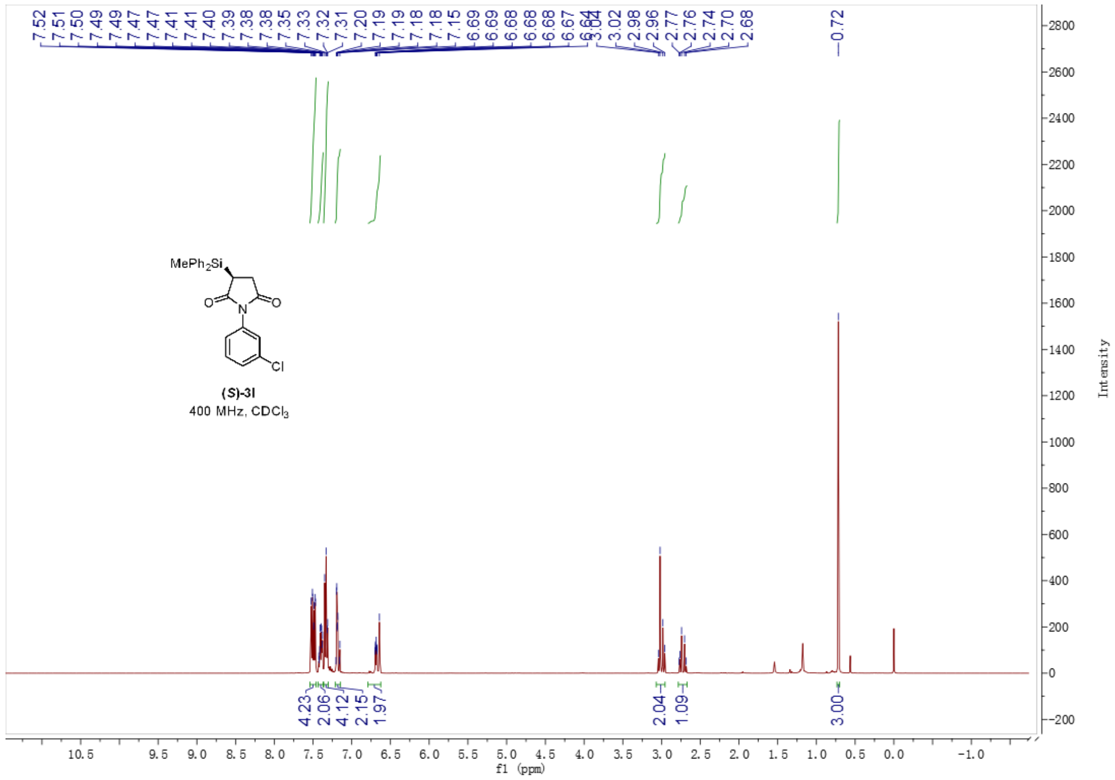

Supplementary Figure 105. ^{1}H NMR spectrum for **3l**

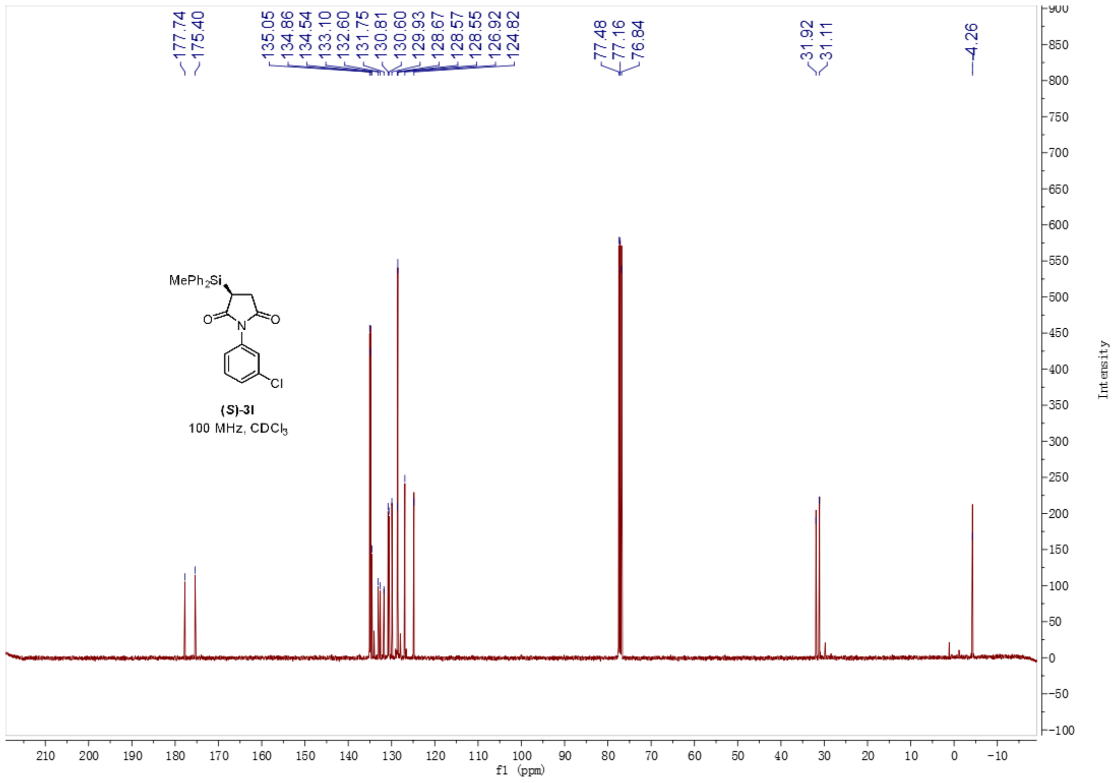

Supplementary Figure 106. ^{13}C NMR spectrum for **3l**

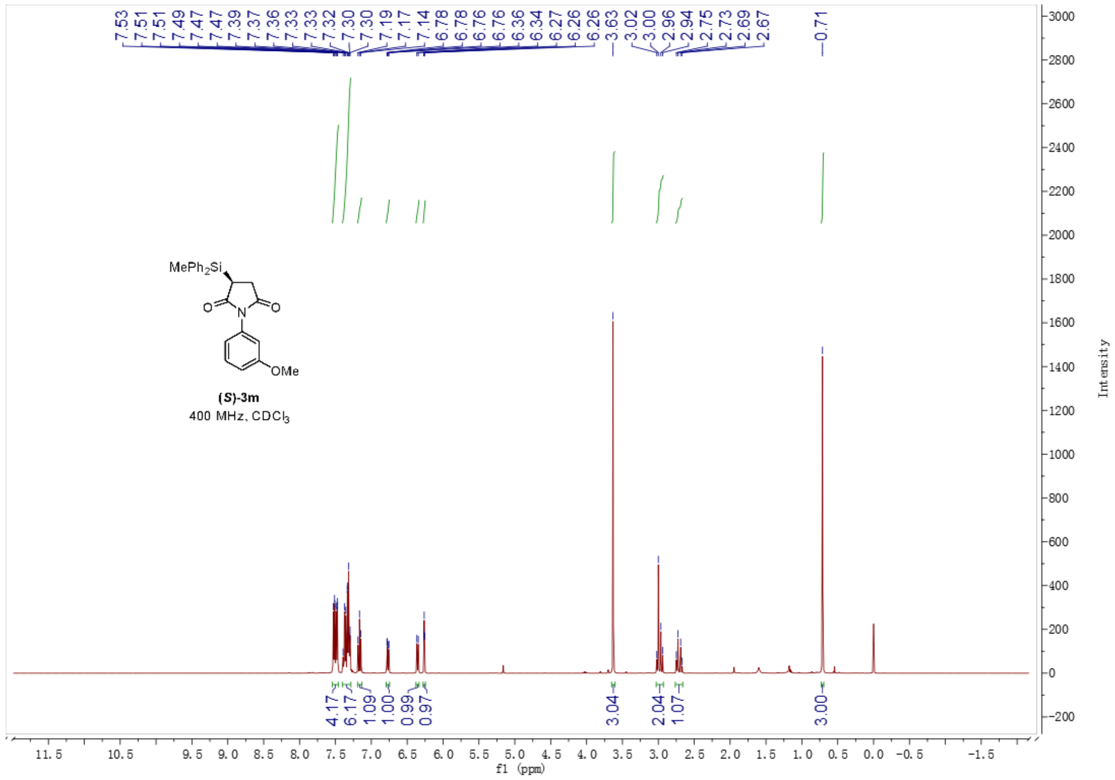

Supplementary Figure 107. ^{1}H NMR spectrum for **3m**

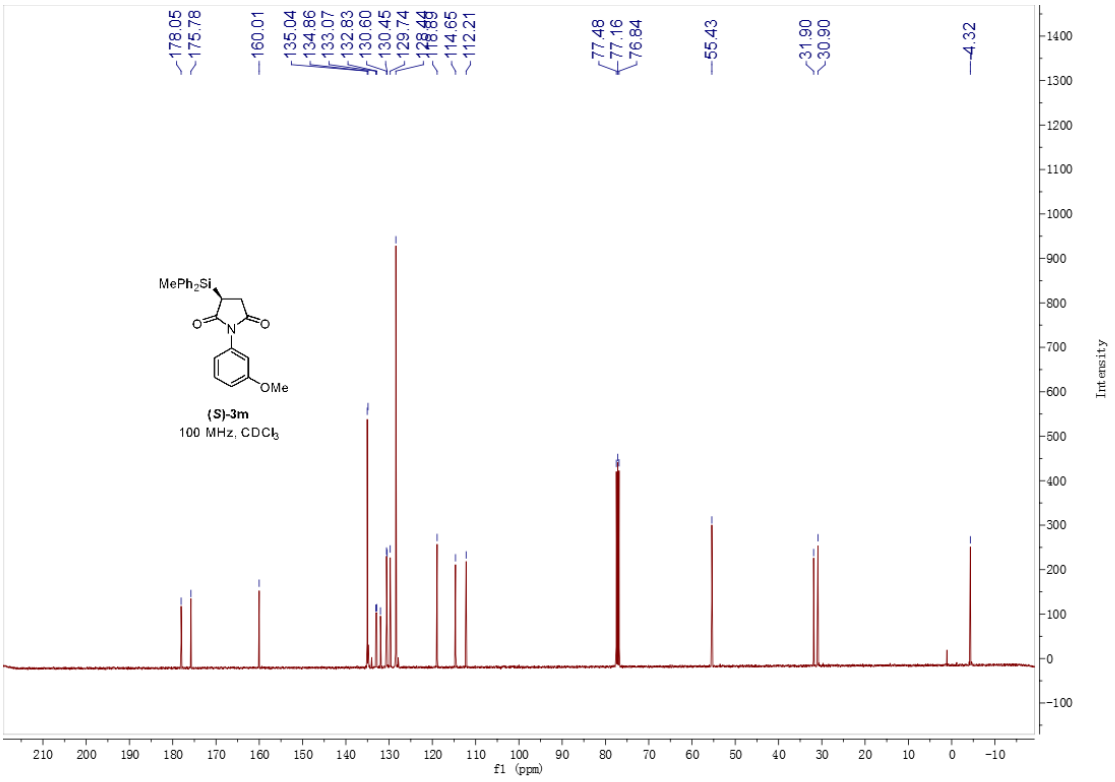

Supplementary Figure 108. ^{13}C NMR spectrum for **3m**

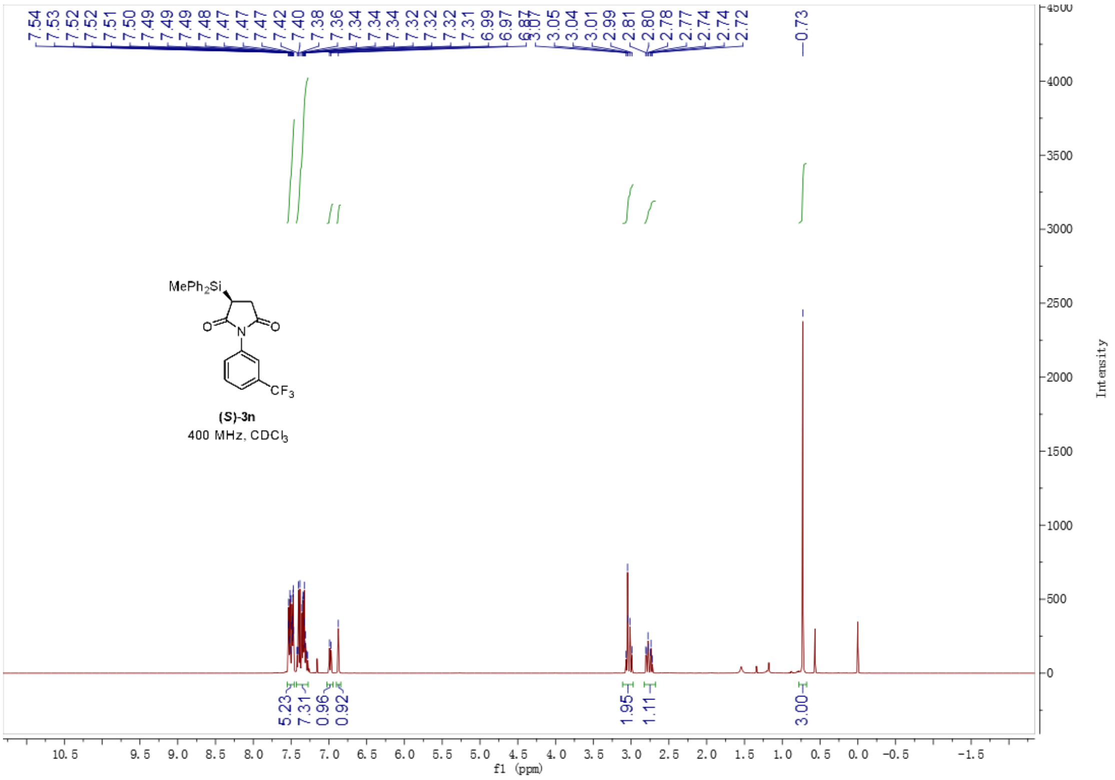

Supplementary Figure 109. ^{1}H NMR spectrum for **3n**

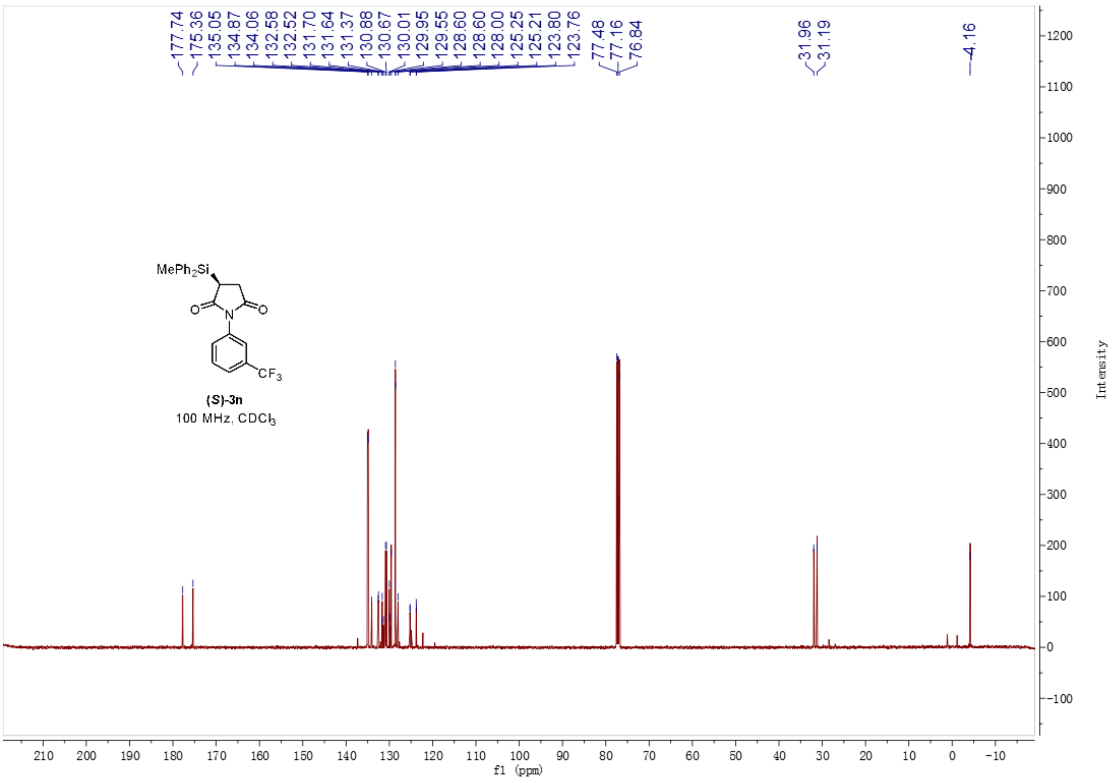

Supplementary Figure 110. ^{13}C NMR spectrum for **3n**

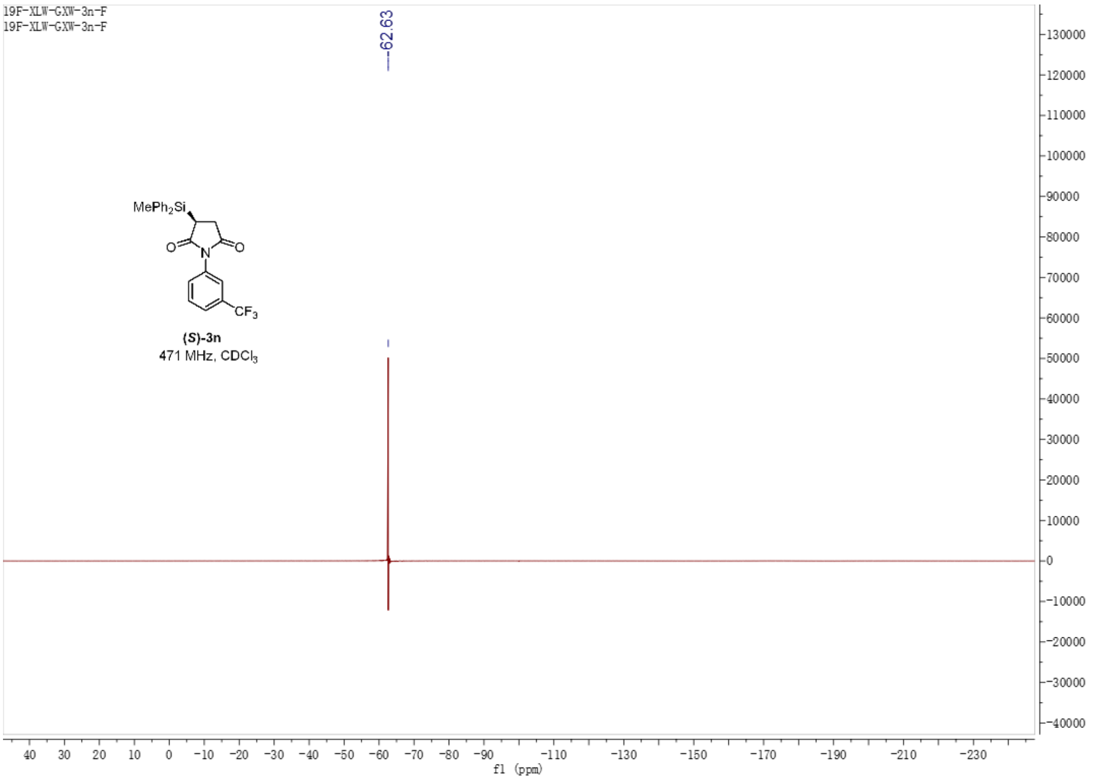

Supplementary Figure 111. ^{19}F NMR spectrum for **3n**

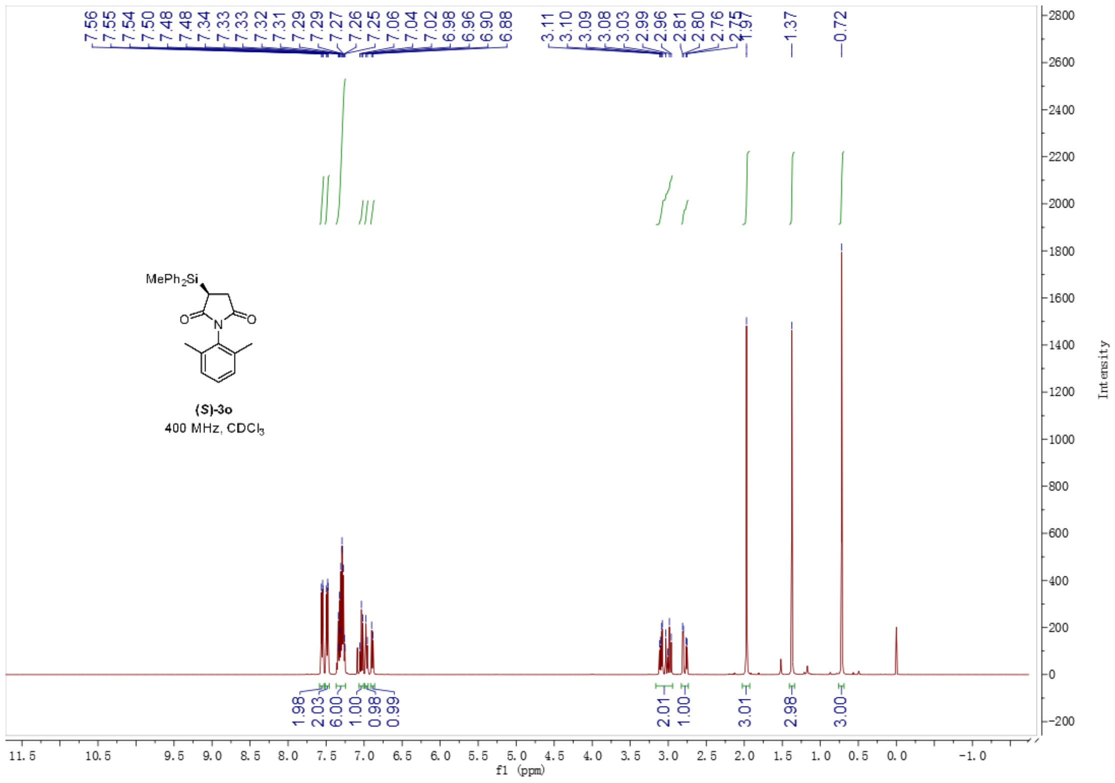

Supplementary Figure 112. ^{1}H NMR spectrum for **3o**

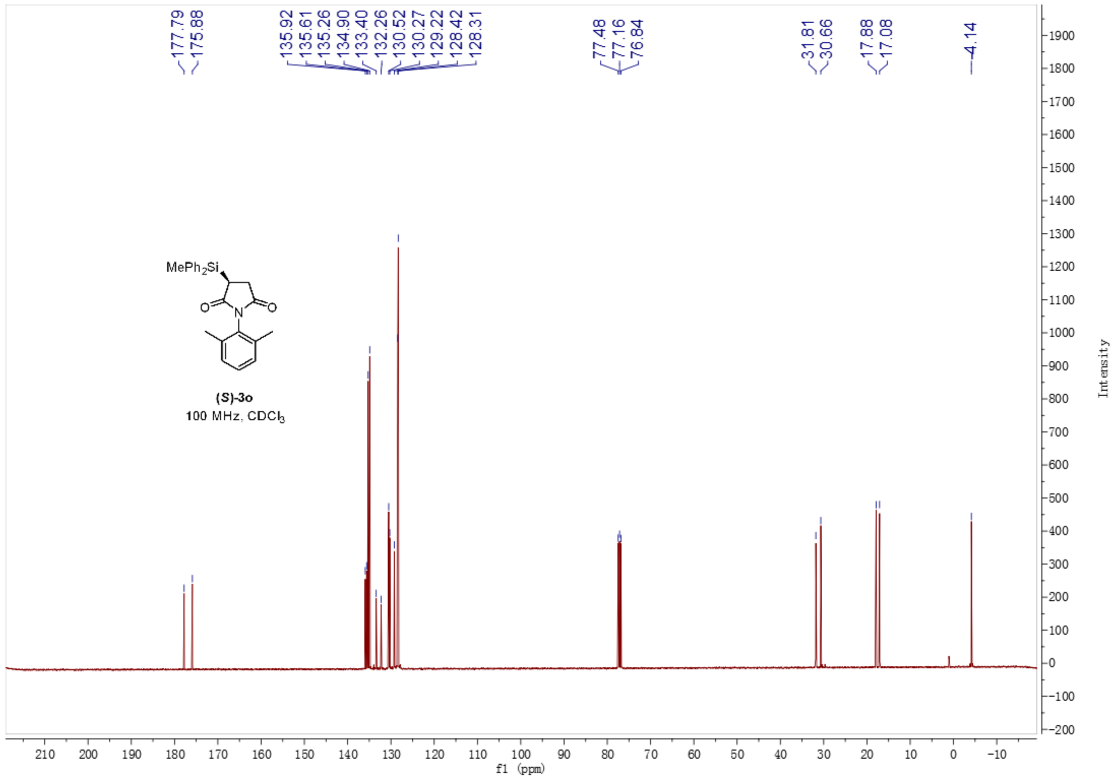

Supplementary Figure 113. ^{13}C NMR spectrum for **3o**

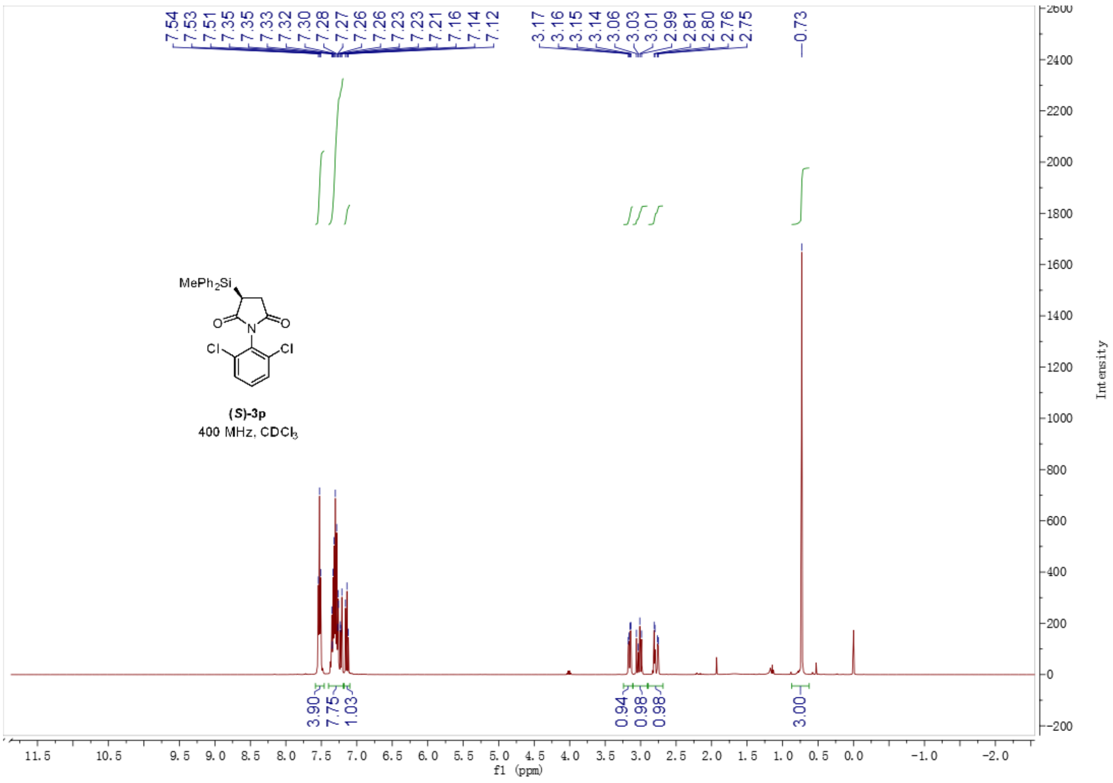

Supplementary Figure 114. ^{1}H NMR spectrum for **3p**

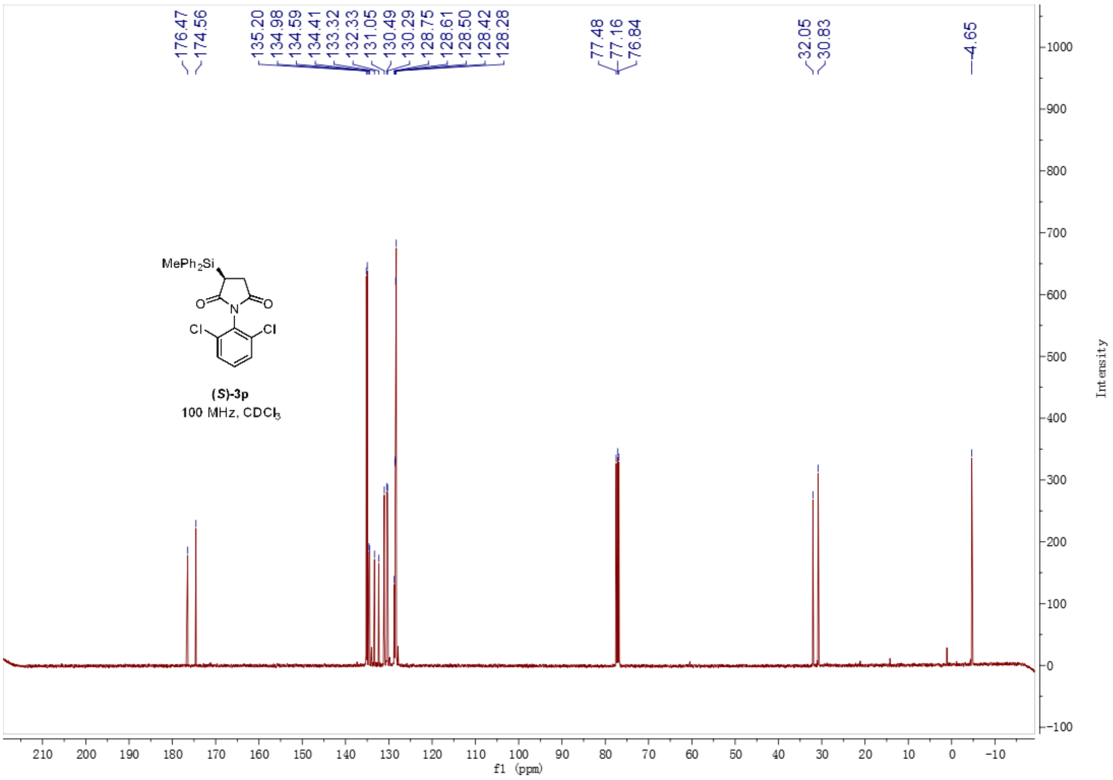

Supplementary Figure 115. ^{13}C NMR spectrum for **3p**

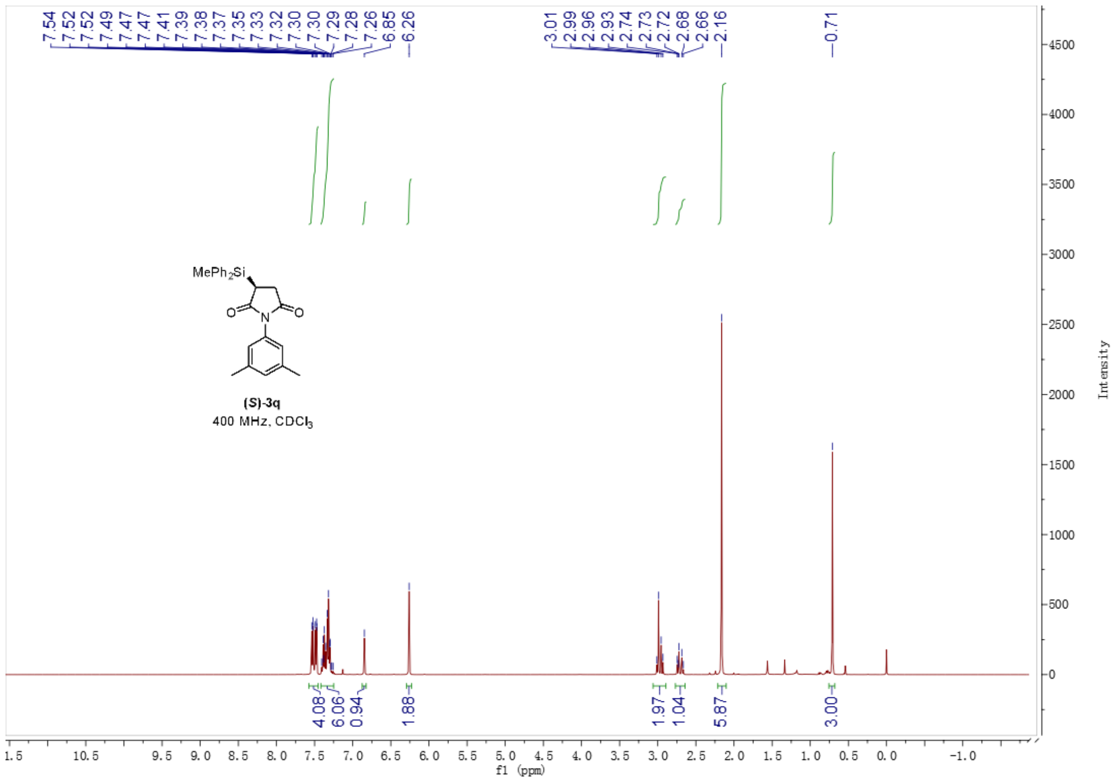

Supplementary Figure 116. ^{1}H NMR spectrum for **3q**

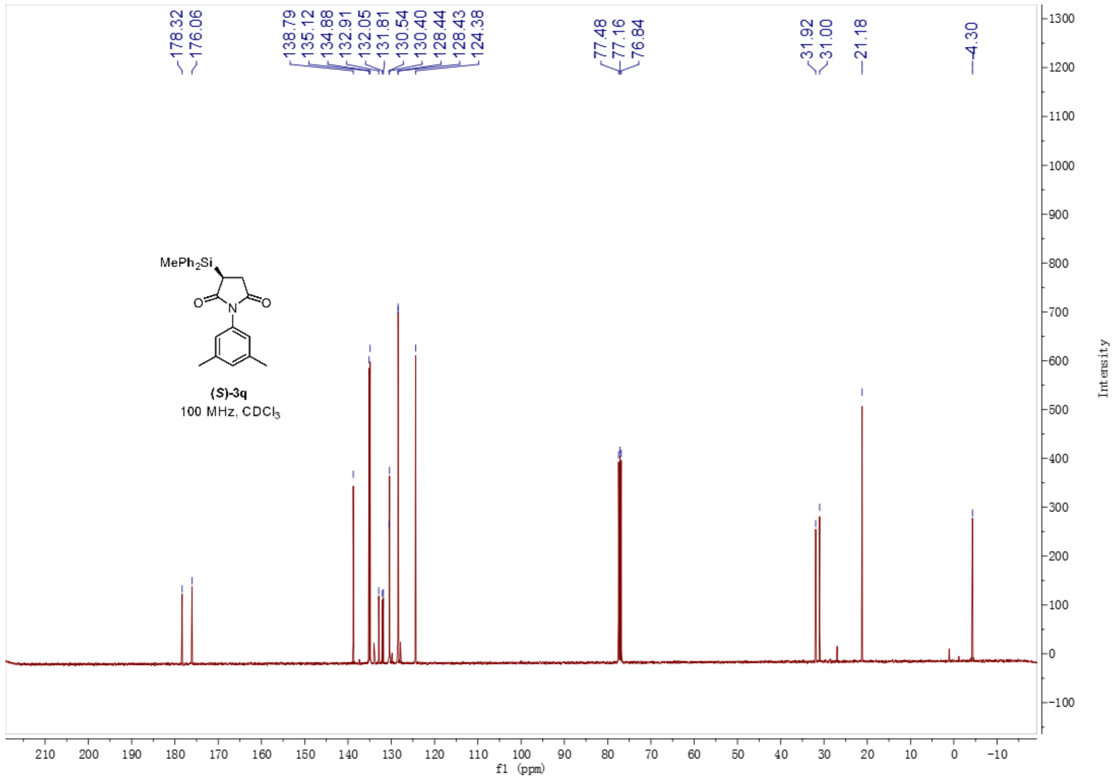

Supplementary Figure 117. ^{13}C NMR spectrum for **3q**

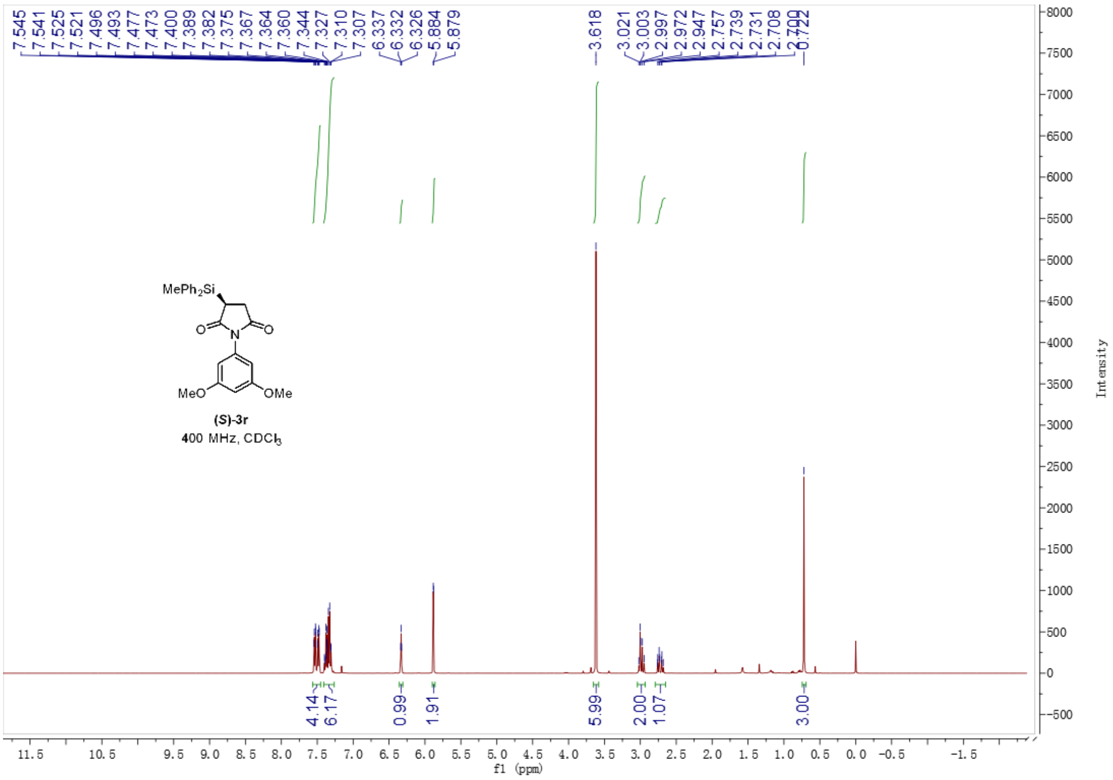

Supplementary Figure 118. ^{1}H NMR spectrum for **3r**

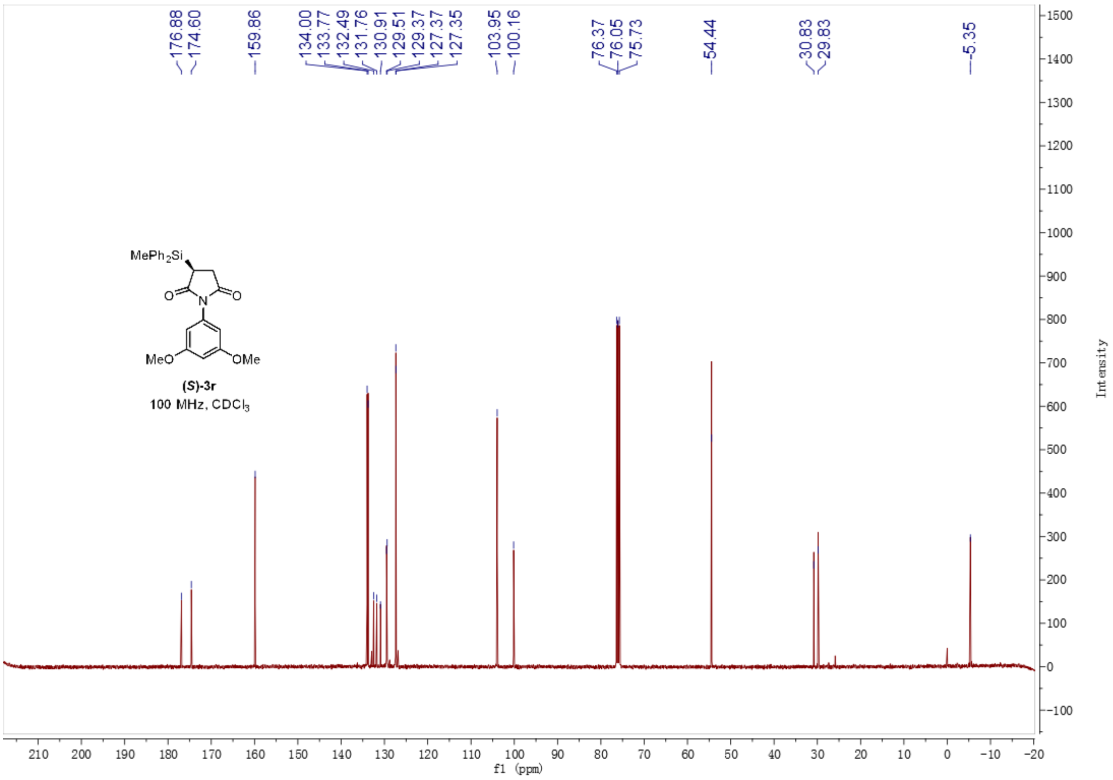

Supplementary Figure 119. ^{13}C NMR spectrum for **3r**

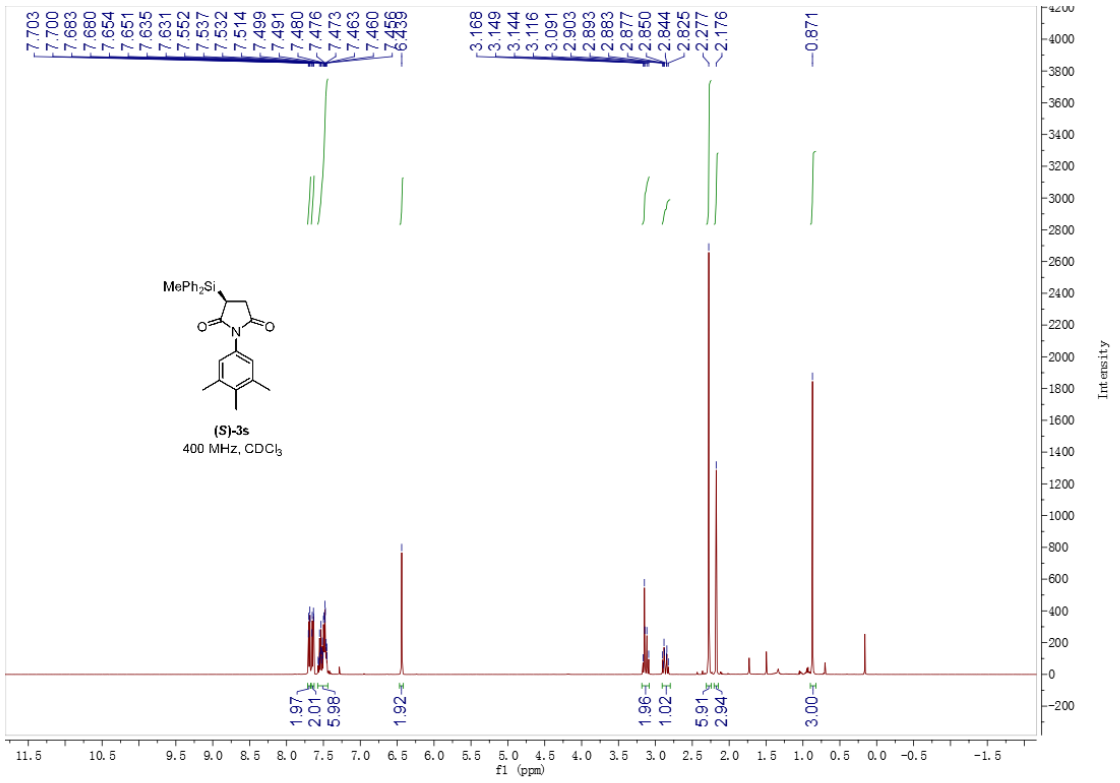

Supplementary Figure 120. ^{1}H NMR spectrum for **3s**

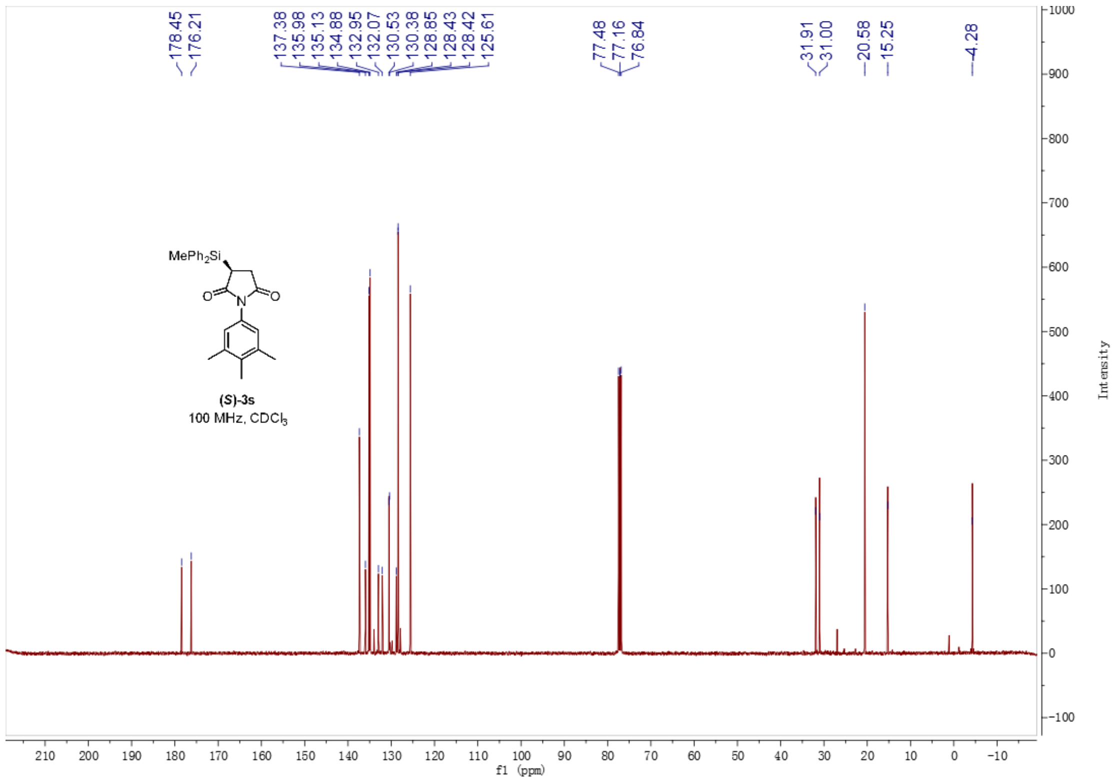

Supplementary Figure 121. ^{13}C NMR spectrum for **3s**

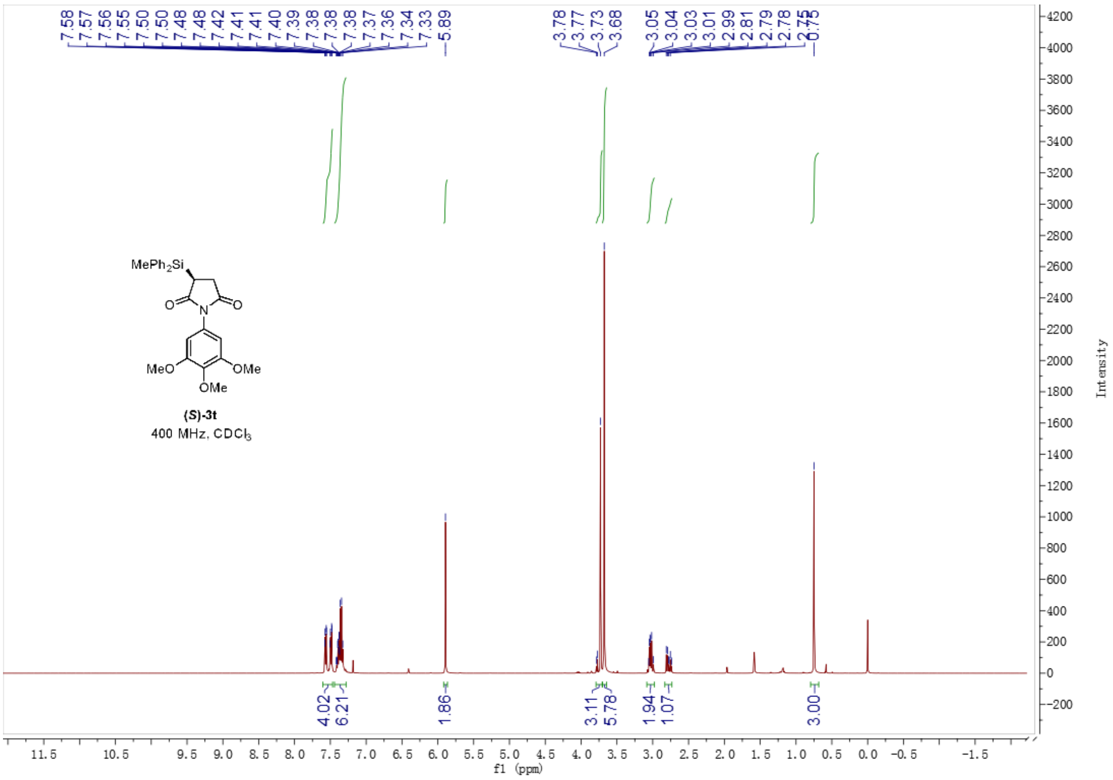

Supplementary Figure 122. ^{1}H NMR spectrum for **3t**

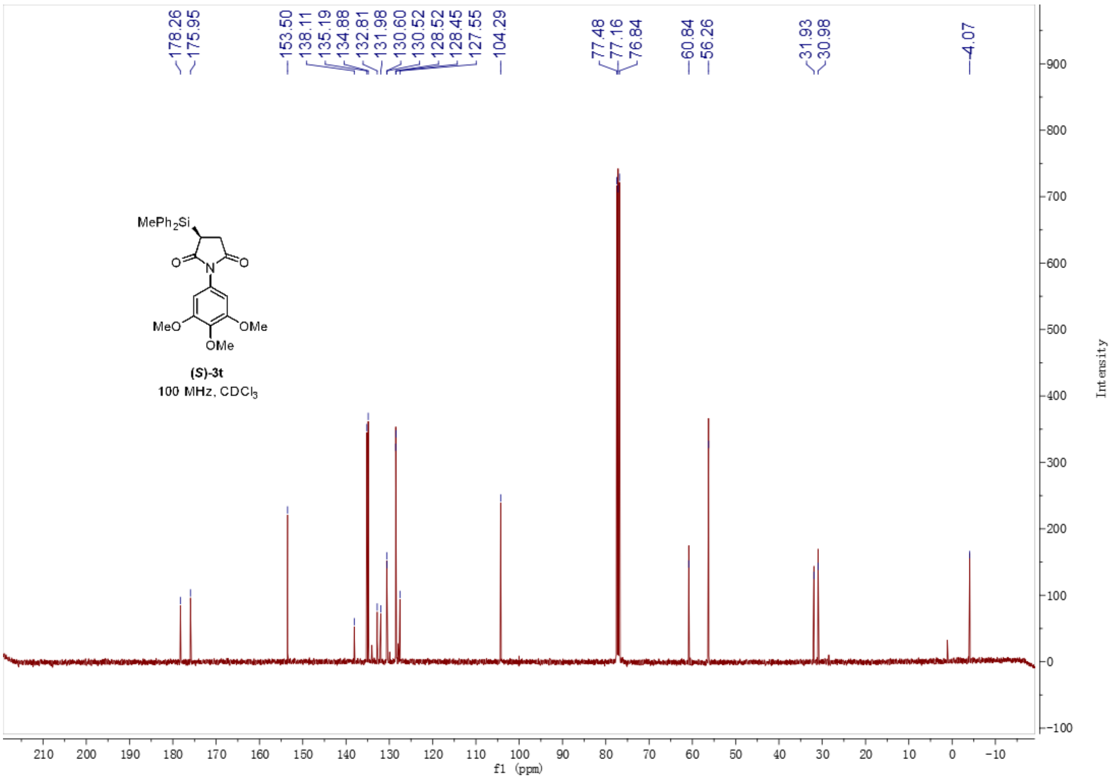

Supplementary Figure 123. ^{13}C NMR spectrum for **3t**

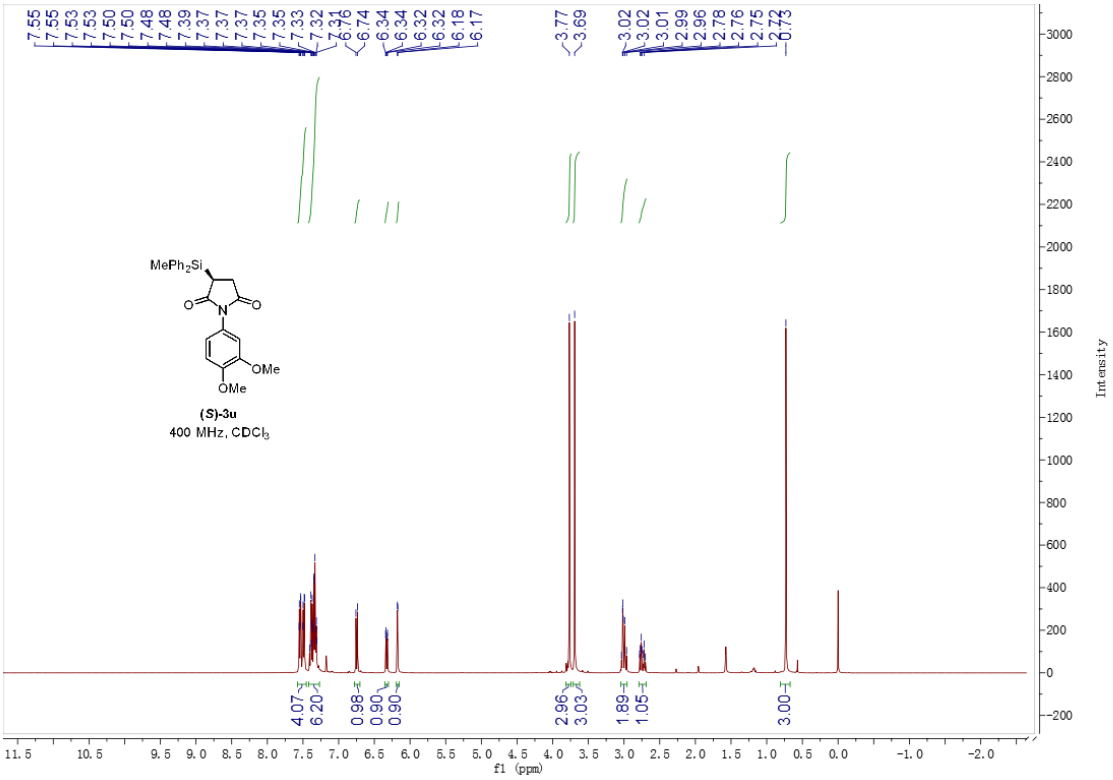

Supplementary Figure 124. ^{1}H NMR spectrum for **3u**

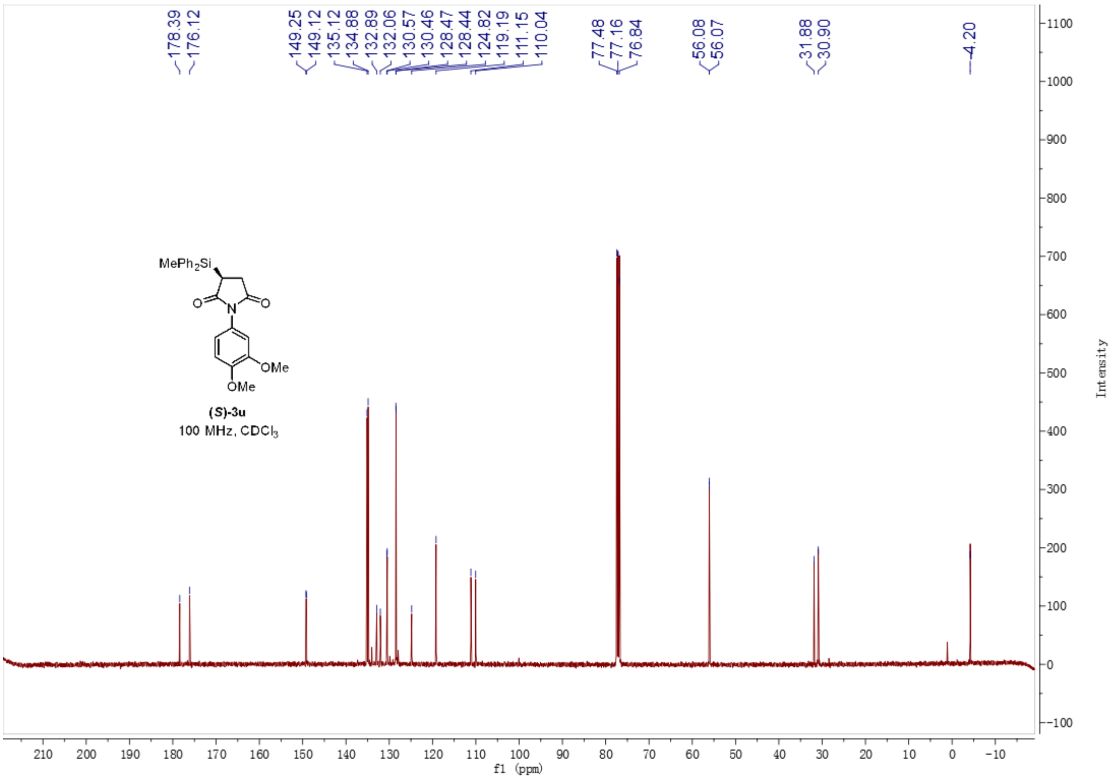

Supplementary Figure 125. ^{13}C NMR spectrum for **3u**

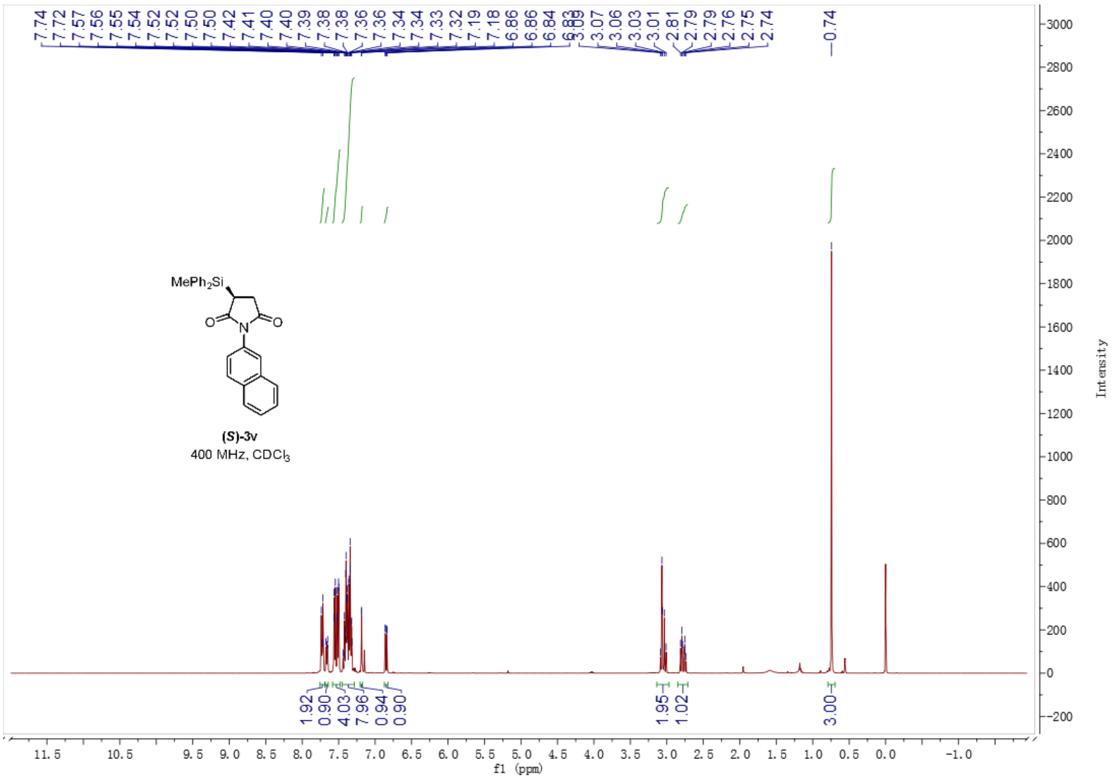

Supplementary Figure 126. ^{1}H NMR spectrum for **3v**

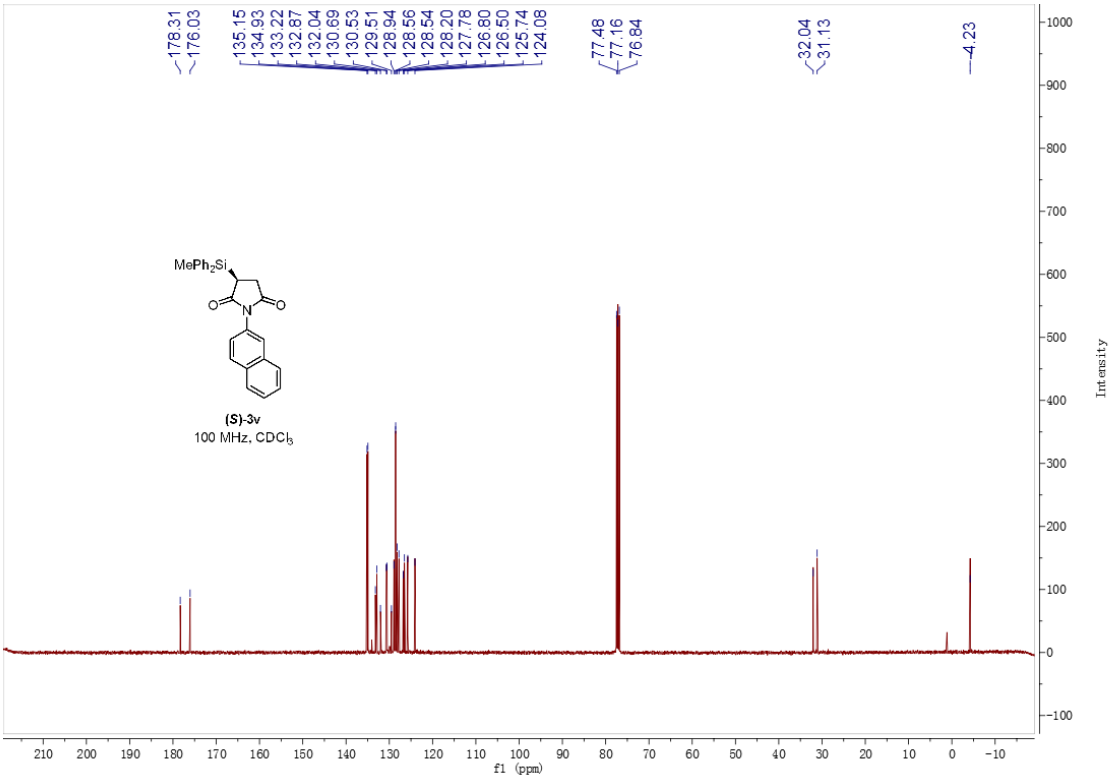

Supplementary Figure 127. ^{13}C NMR spectrum for **3v**

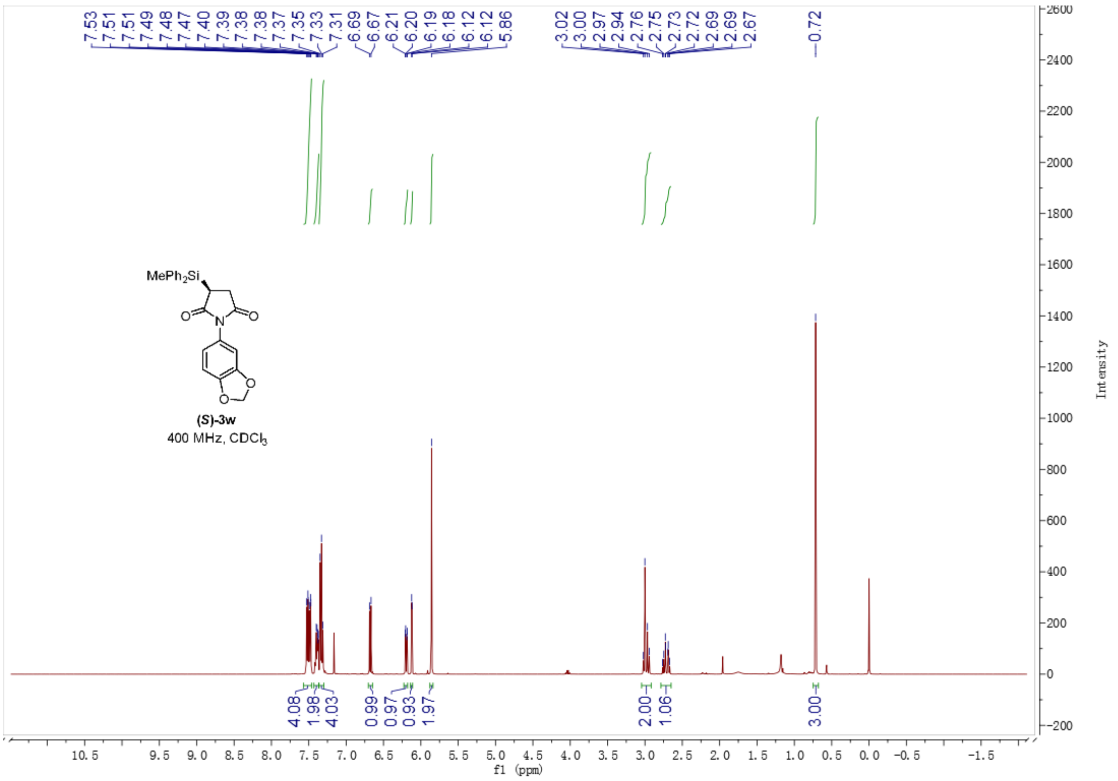

Supplementary Figure 128. ^{1}H NMR spectrum for **3w**

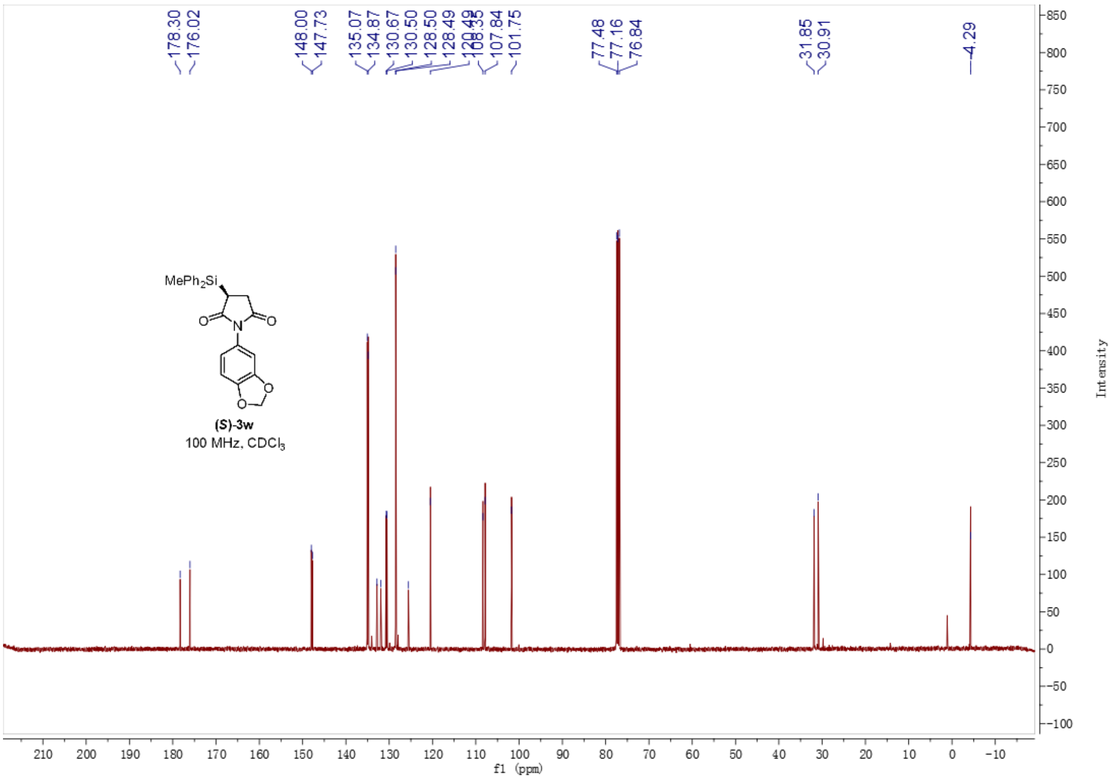

Supplementary Figure 129. ^{13}C NMR spectrum for **3w**

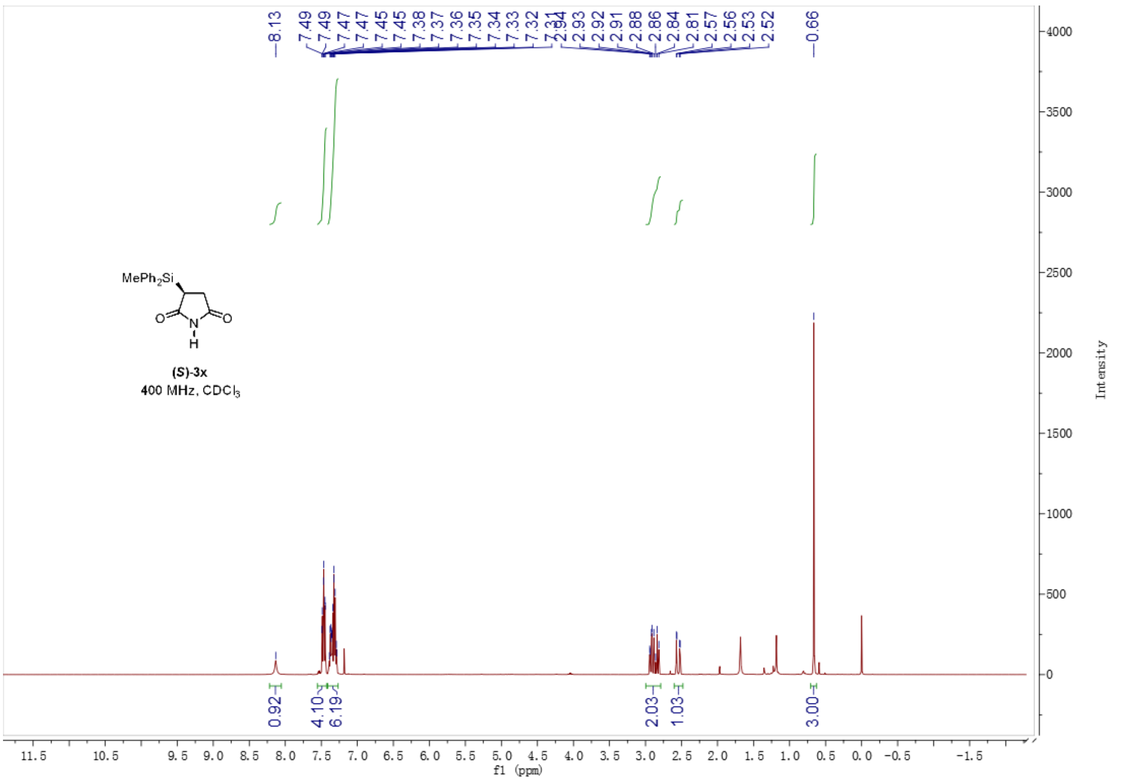

Supplementary Figure 130. ^{1}H NMR spectrum for **3x**

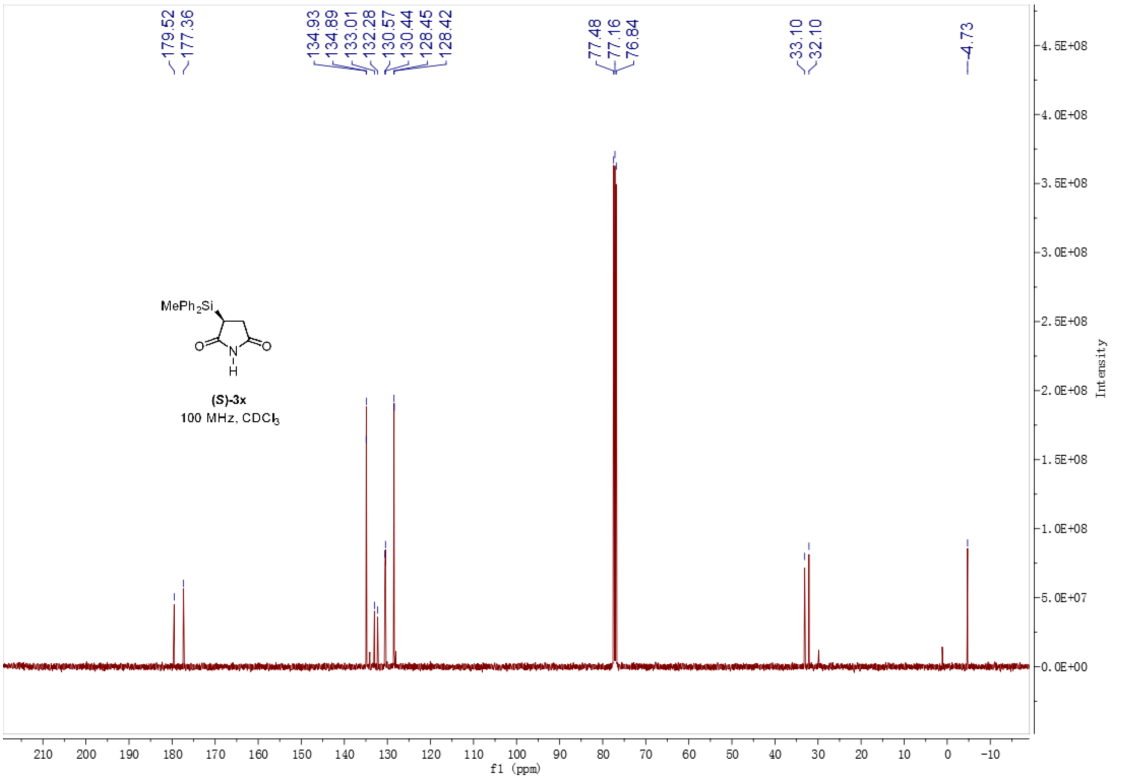

Supplementary Figure 131. ^{13}C NMR spectrum for **3x**

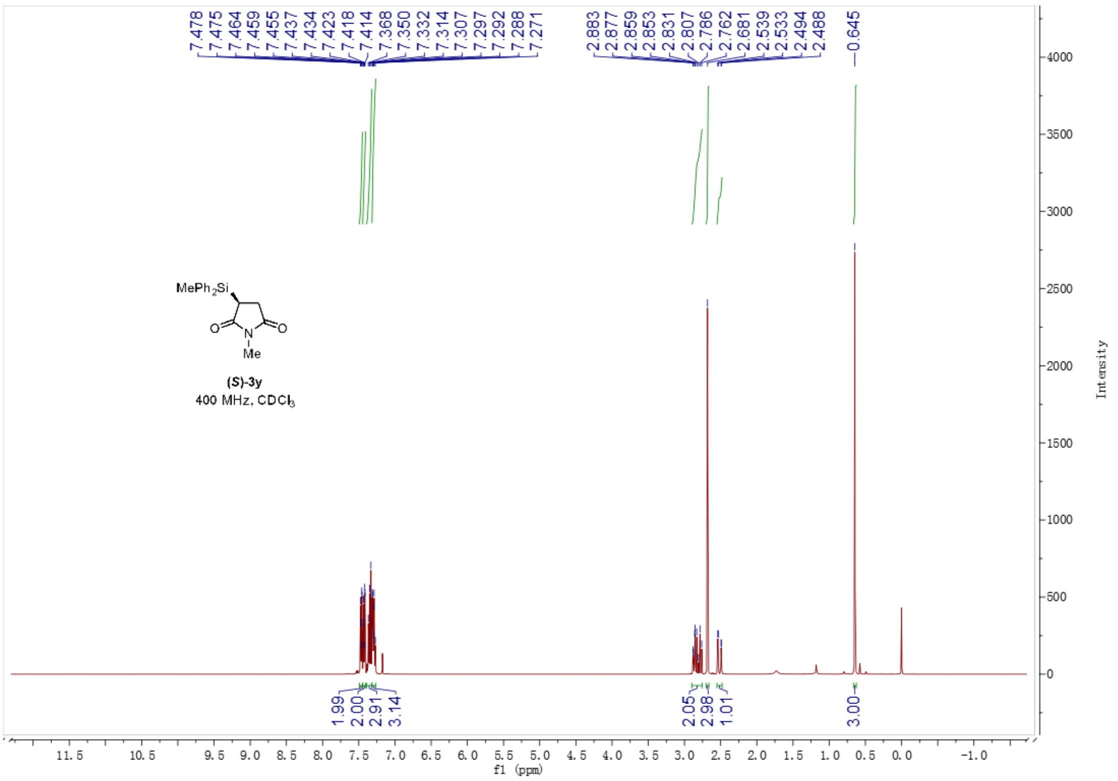

Supplementary Figure 132. ^{1}H NMR spectrum for **3y**

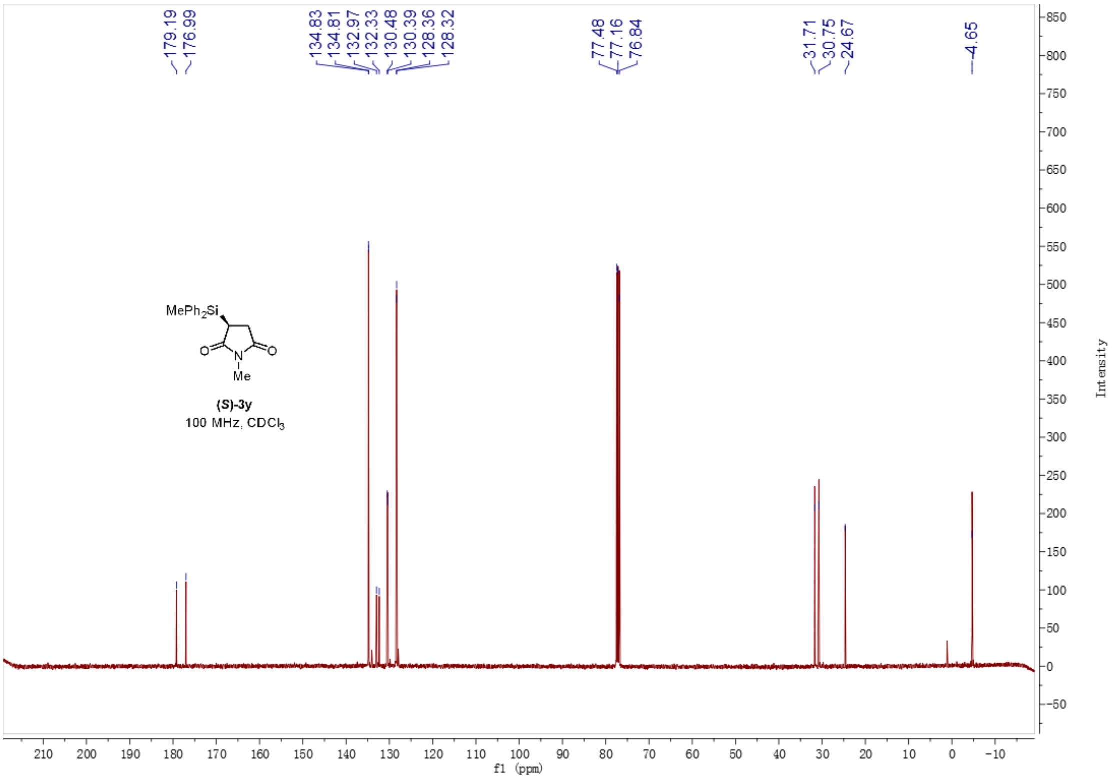

Supplementary Figure 133. ^{13}C NMR spectrum for **3y**

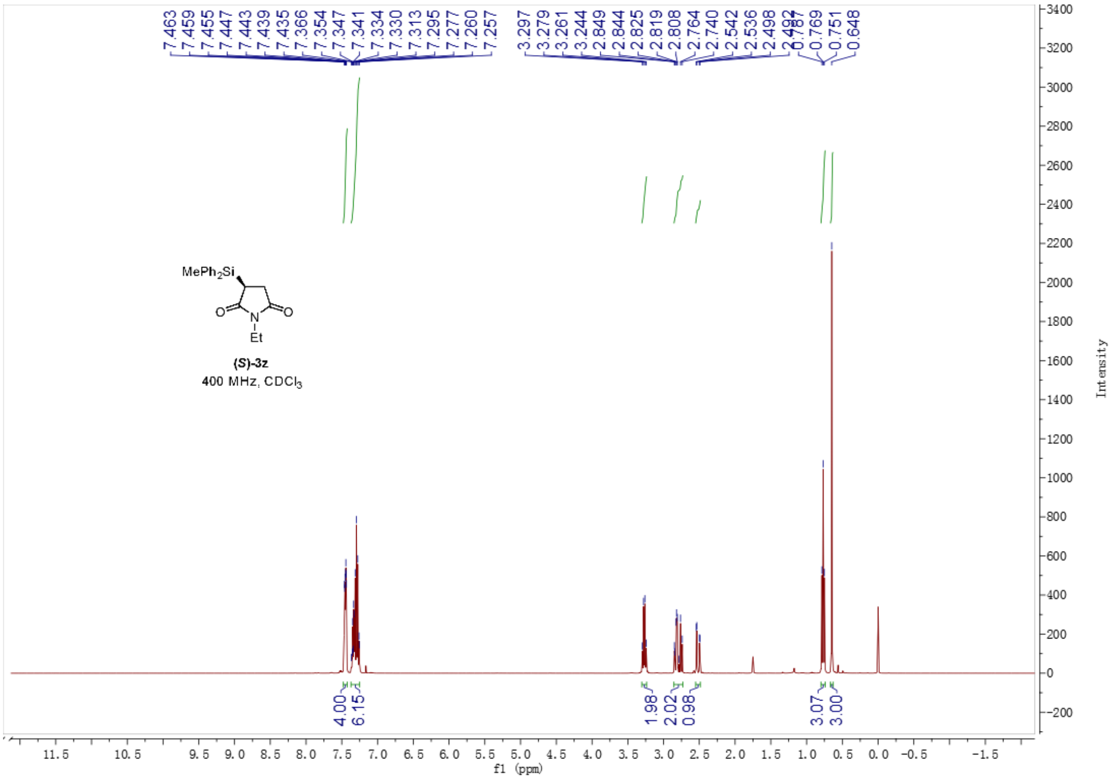

Supplementary Figure 134. ^{1}H NMR spectrum for **3z**

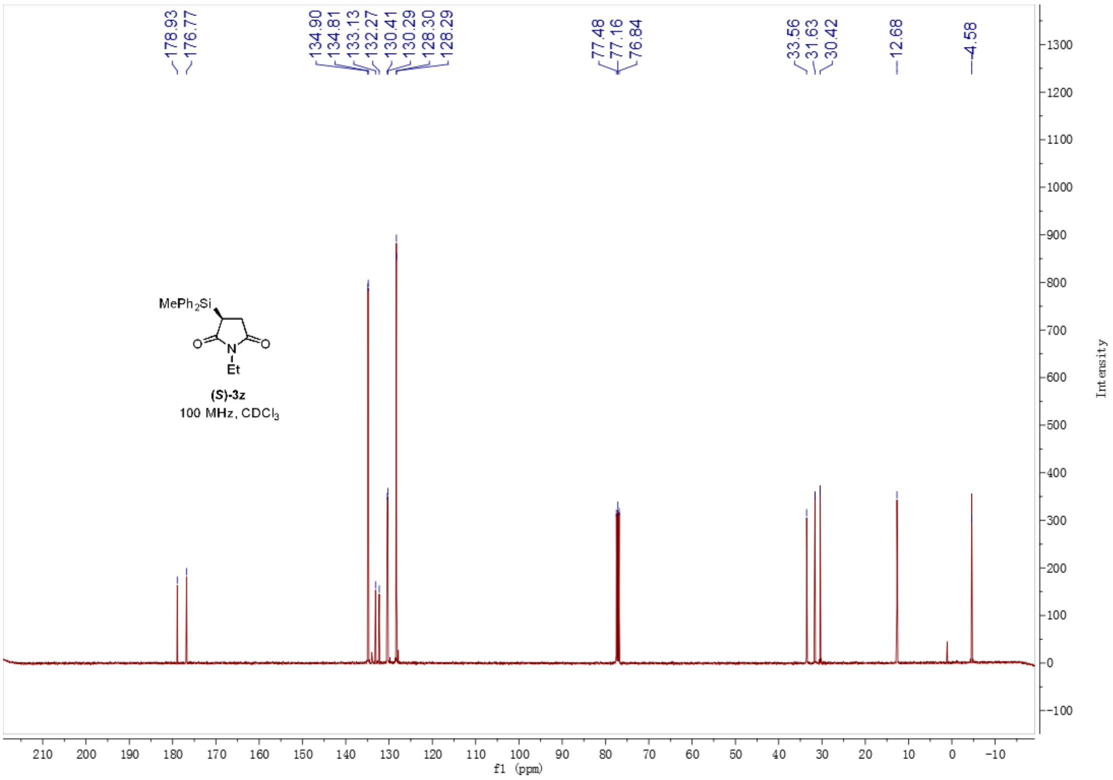

Supplementary Figure 135. ^{13}C NMR spectrum for **3z**

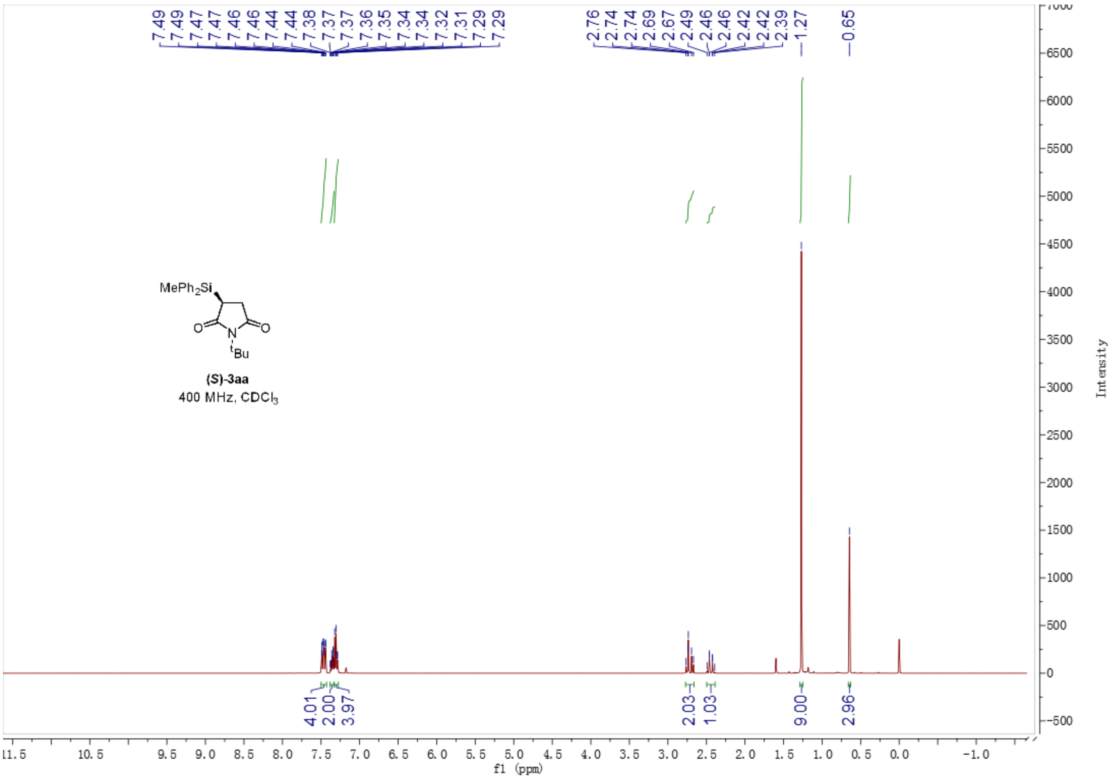

Supplementary Figure 136. ^{1}H NMR spectrum for **3aa**

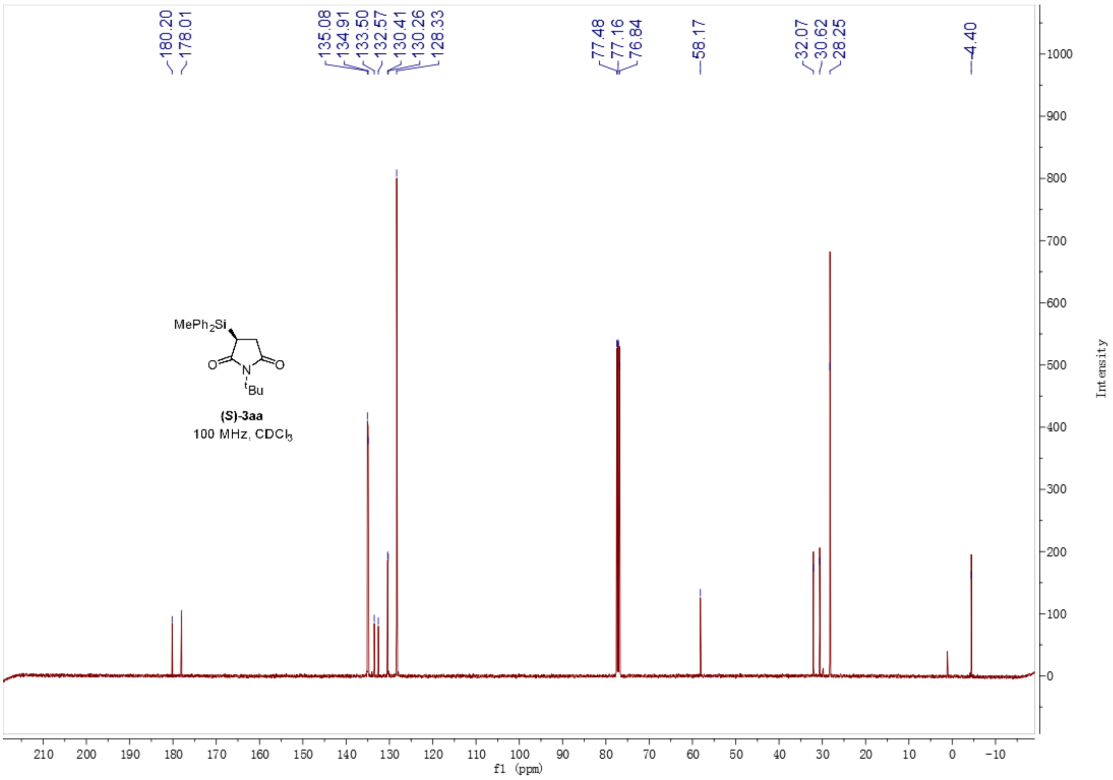

Supplementary Figure 137. ^{13}C NMR spectrum for **3aa**

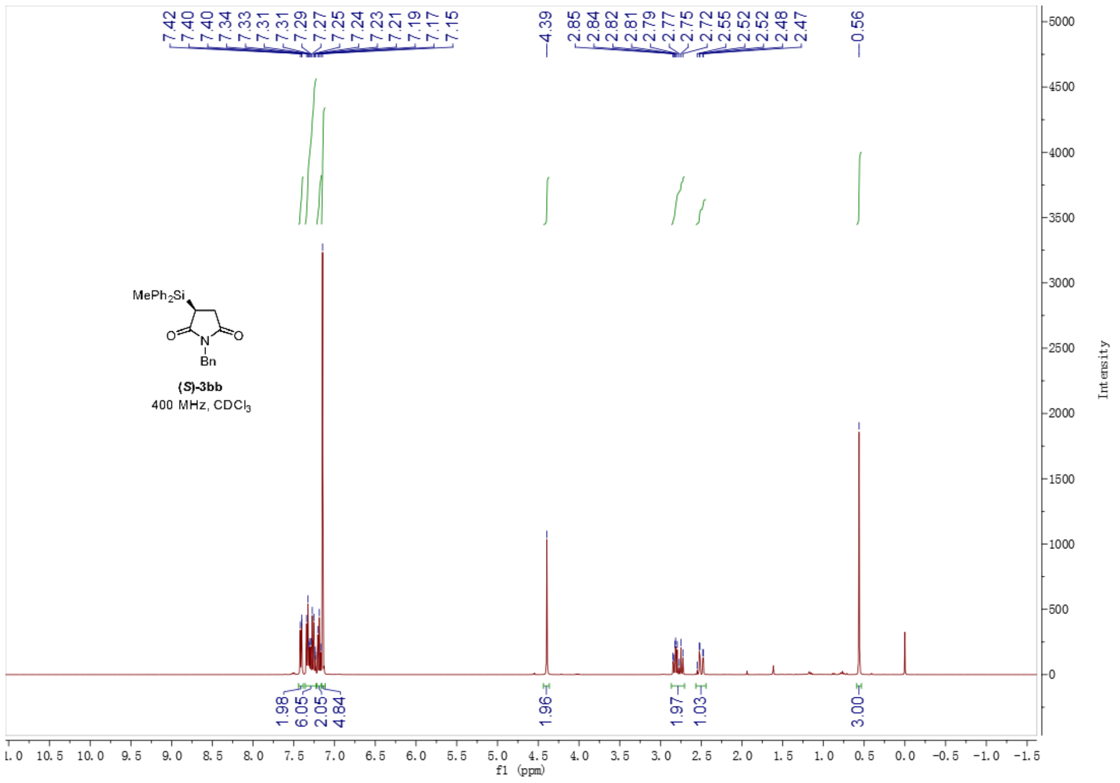

Supplementary Figure 138. ^{1}H NMR spectrum for **3bb**

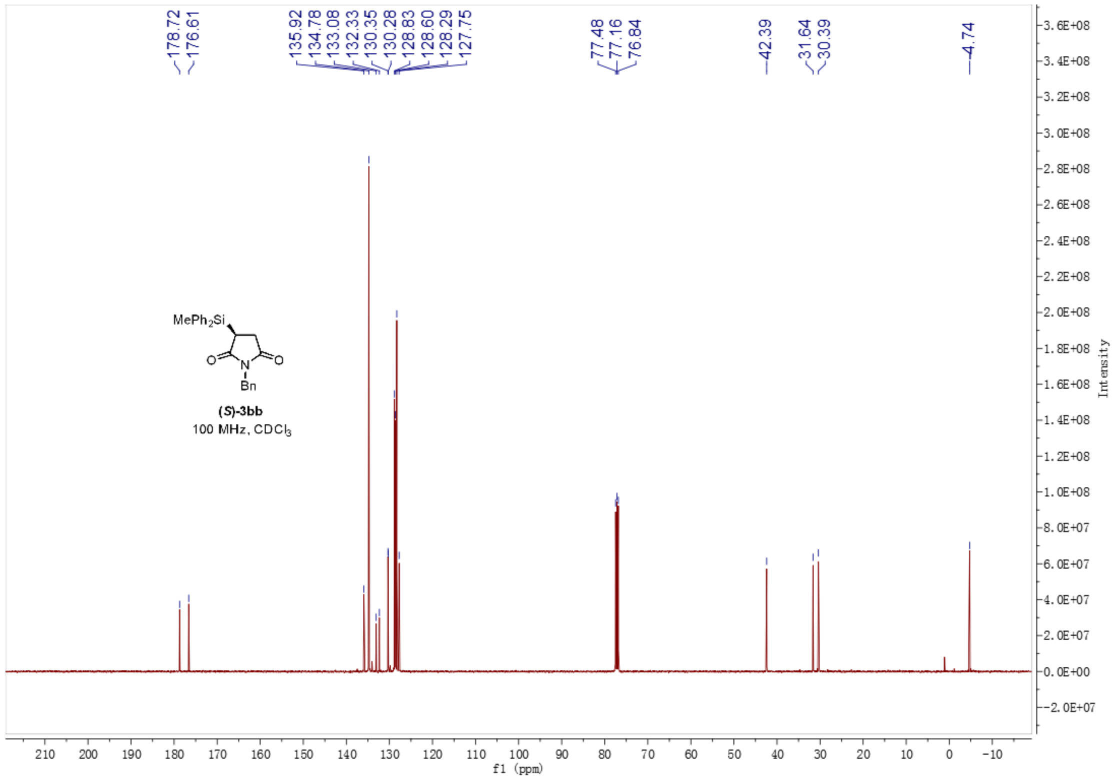

Supplementary Figure 139. ^{13}C NMR spectrum for **3bb**

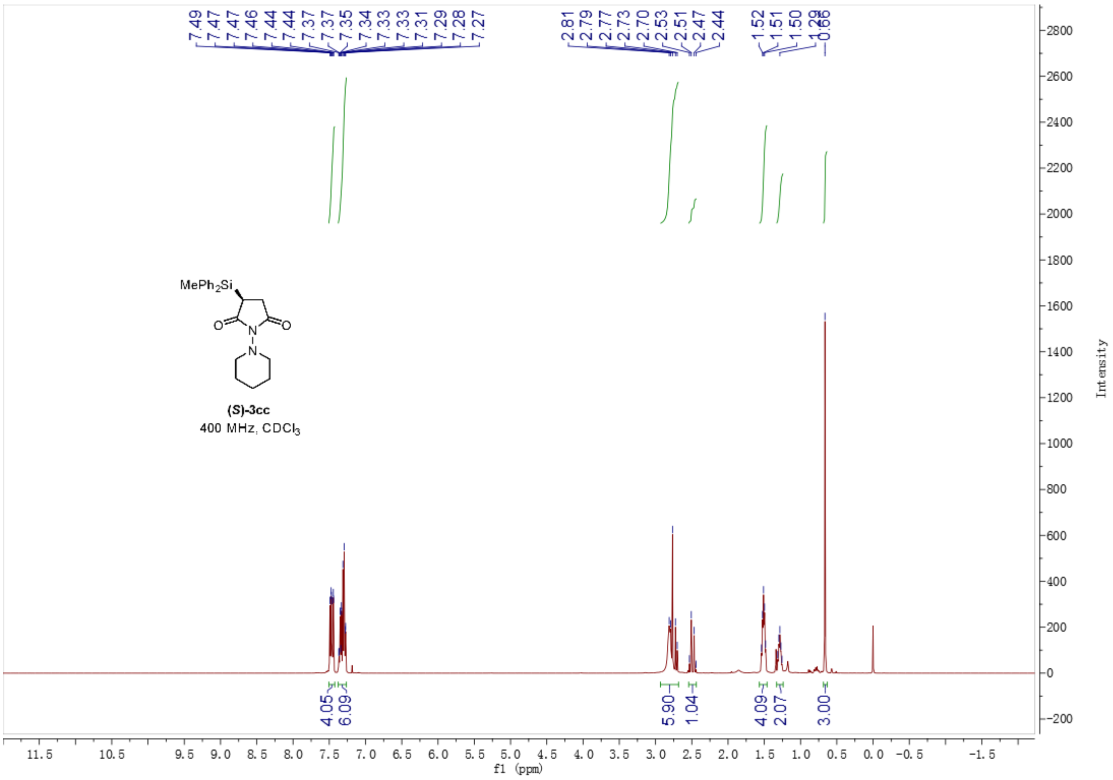

Supplementary Figure 140. ^{1}H NMR spectrum for **3cc**

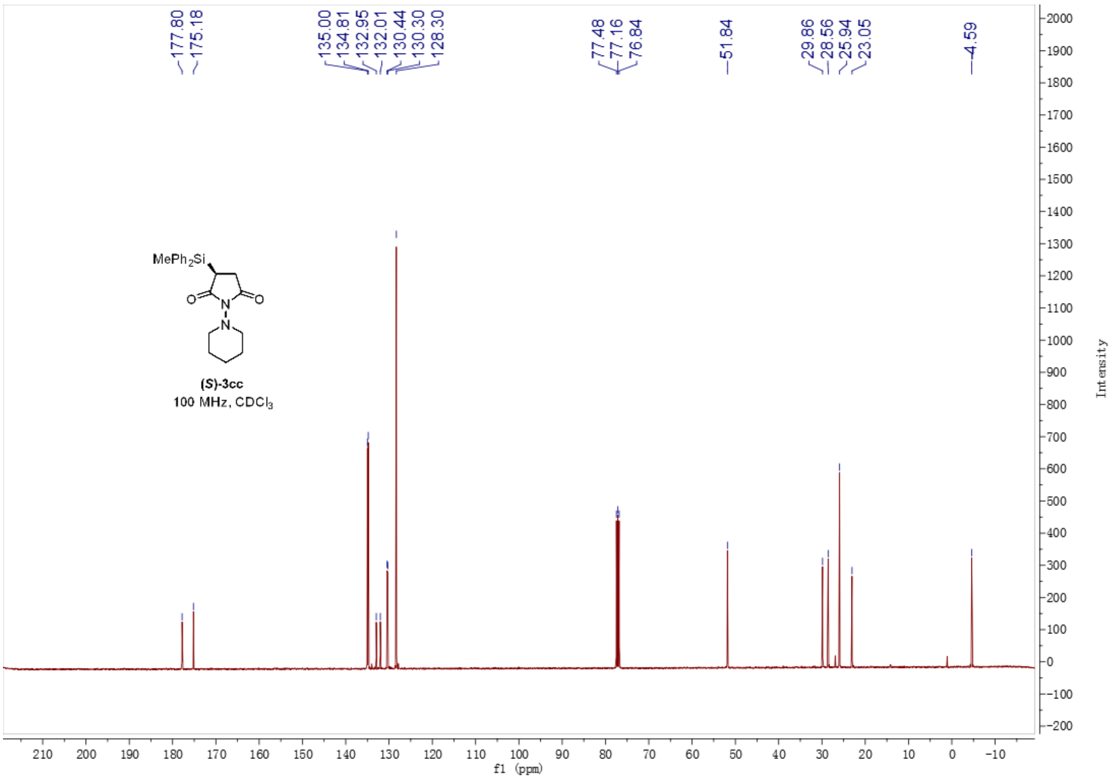

Supplementary Figure 141. ^{13}C NMR spectrum for **3cc**

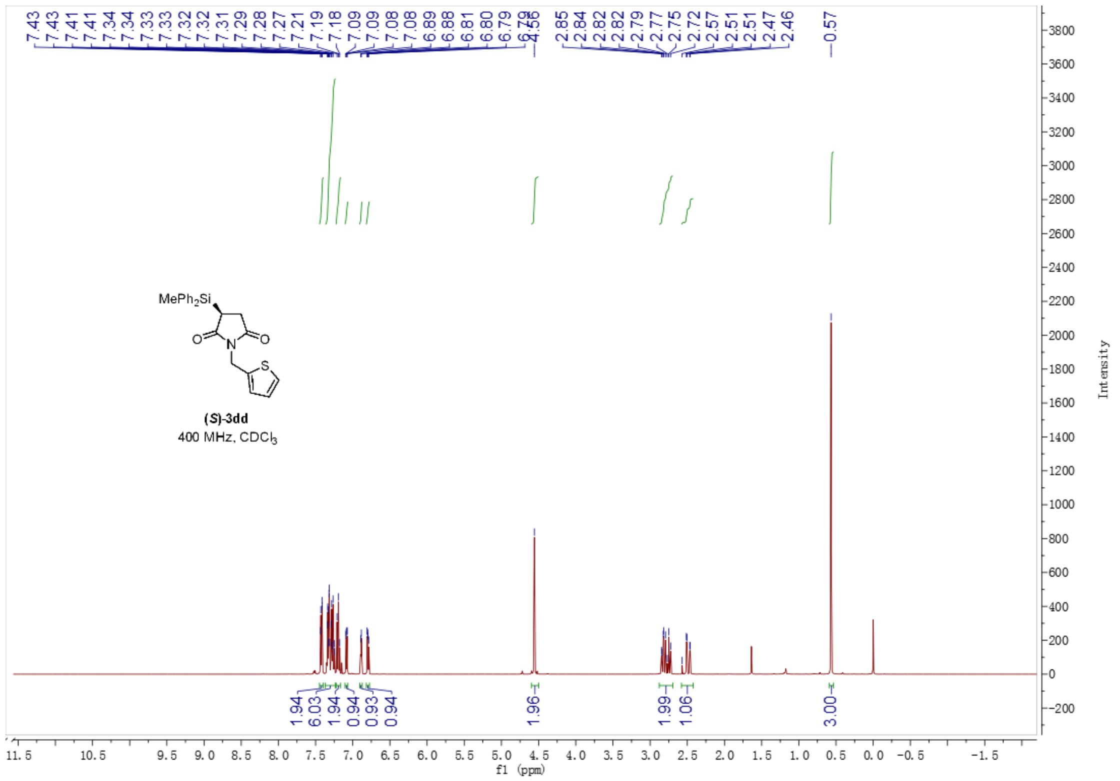

Supplementary Figure 142. ^{1}H NMR spectrum for **3dd**

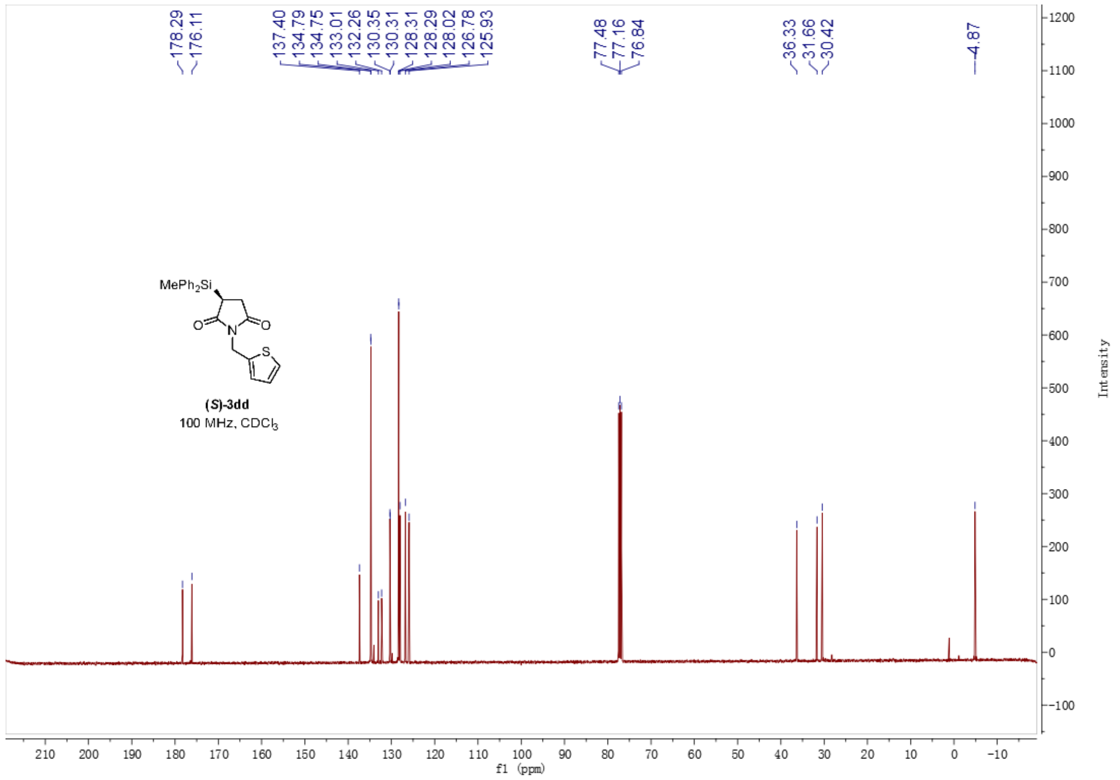

Supplementary Figure 143. ^{13}C NMR spectrum for **3dd**

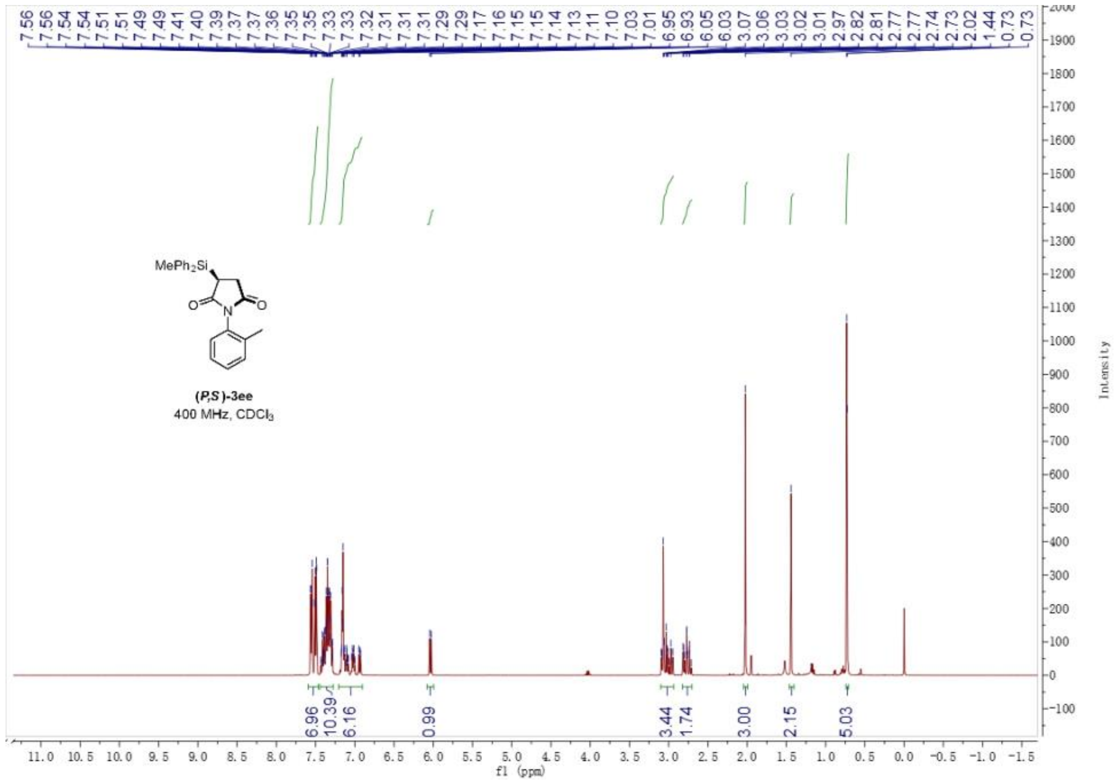

Supplementary Figure 144. ^{1}H NMR spectrum for **3ee**

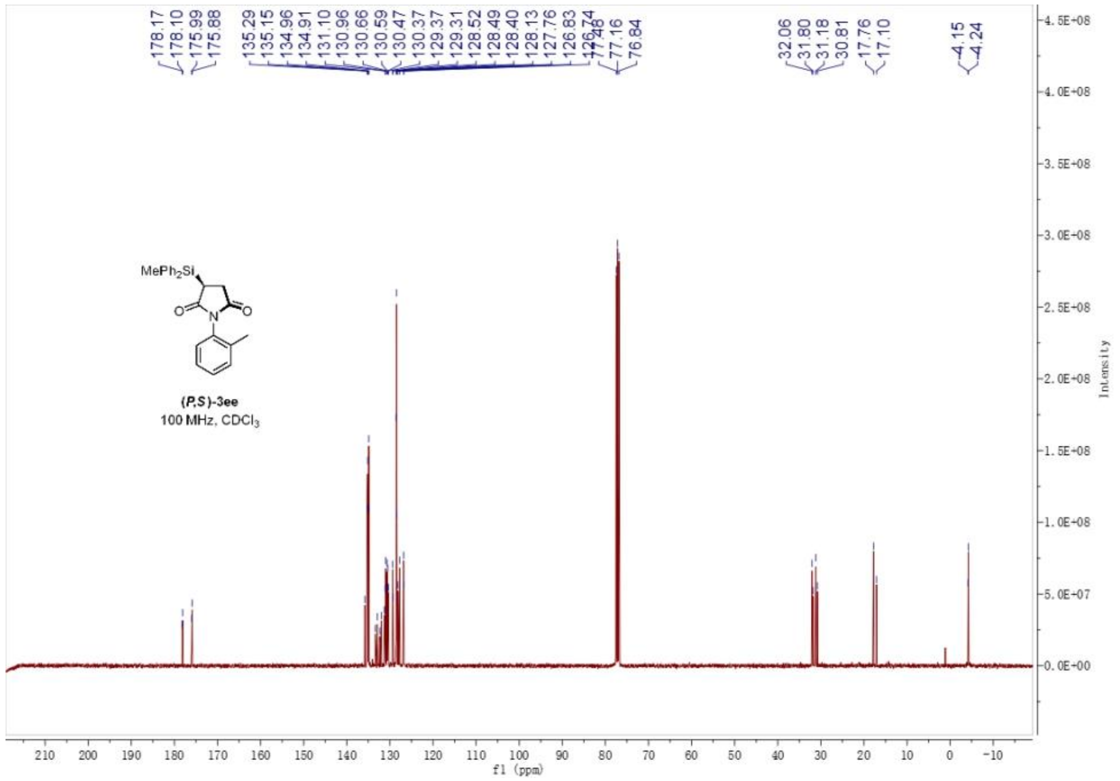

Supplementary Figure 145. ^{13}C NMR spectrum for **3ee**

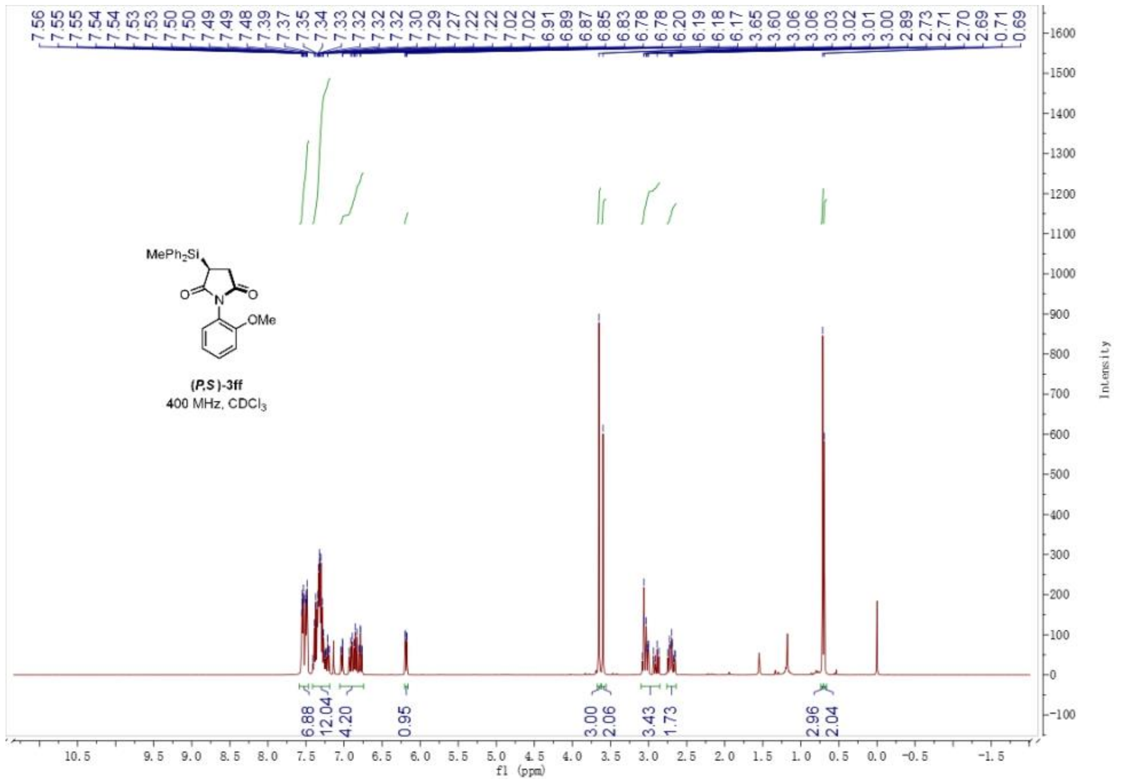

Supplementary Figure 146. ^{1}H NMR spectrum for **3ff**

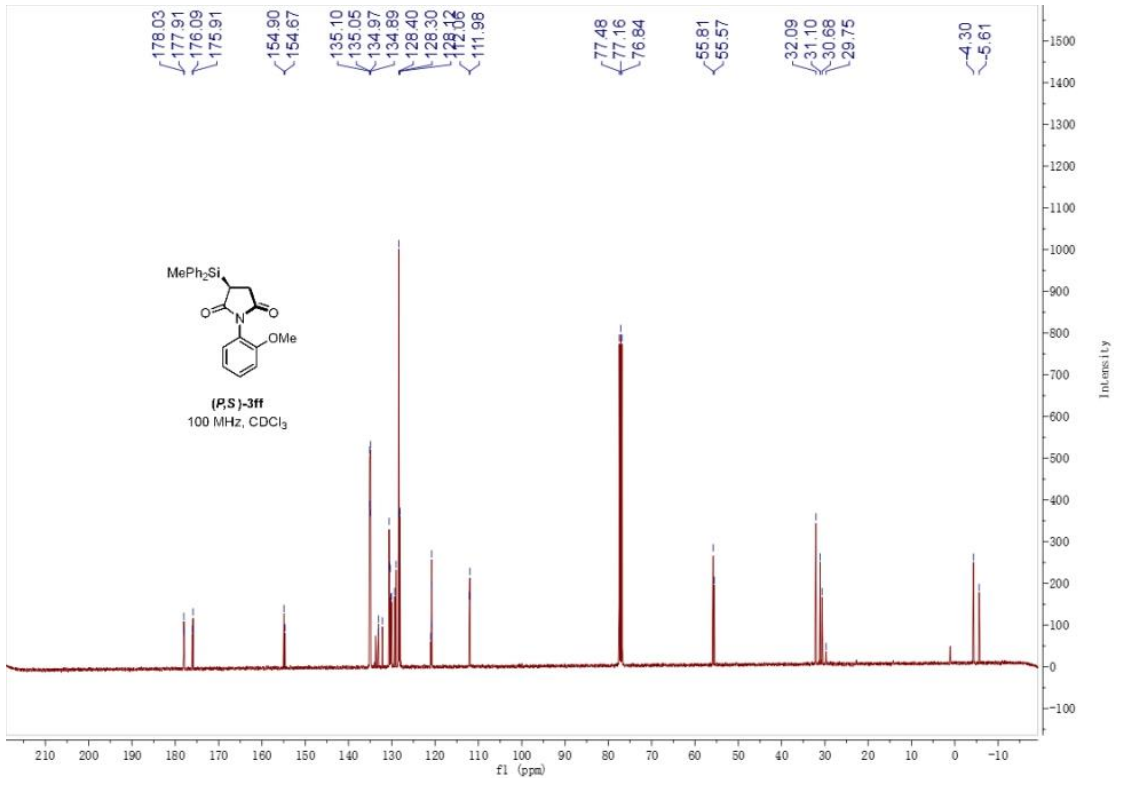

Supplementary Figure 147. ^{13}C NMR spectrum for **3ff**

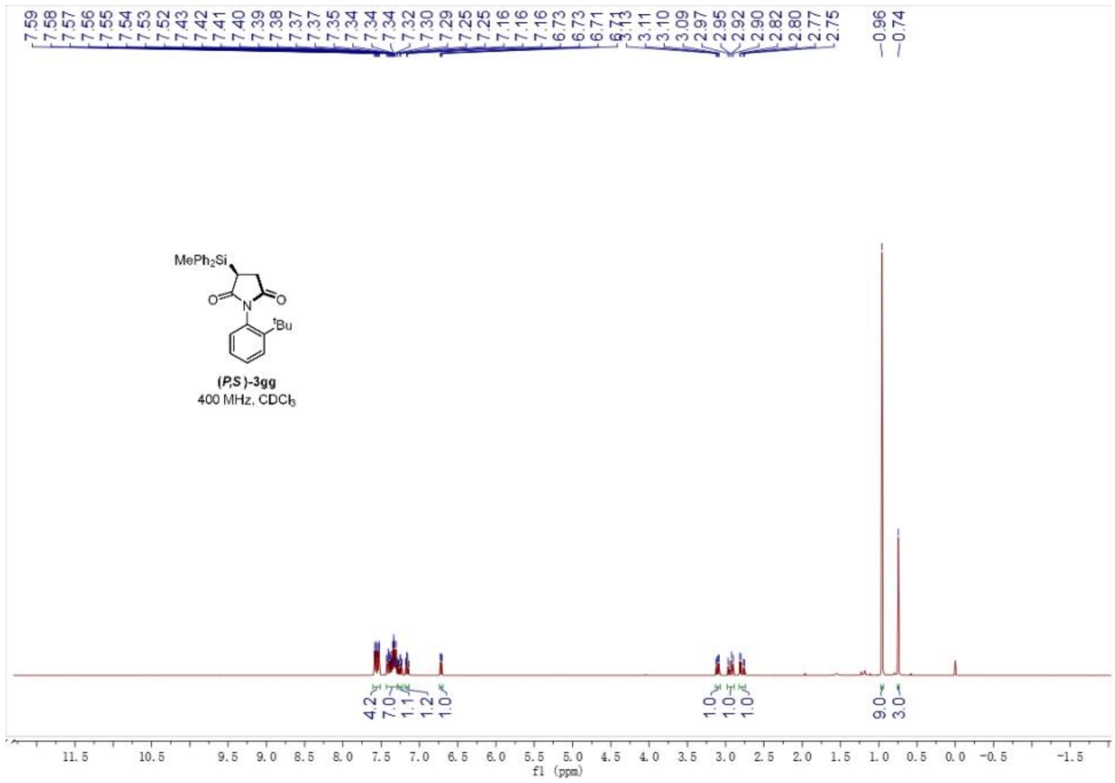

Supplementary Figure 148. ^{1}H NMR spectrum for **3gg**

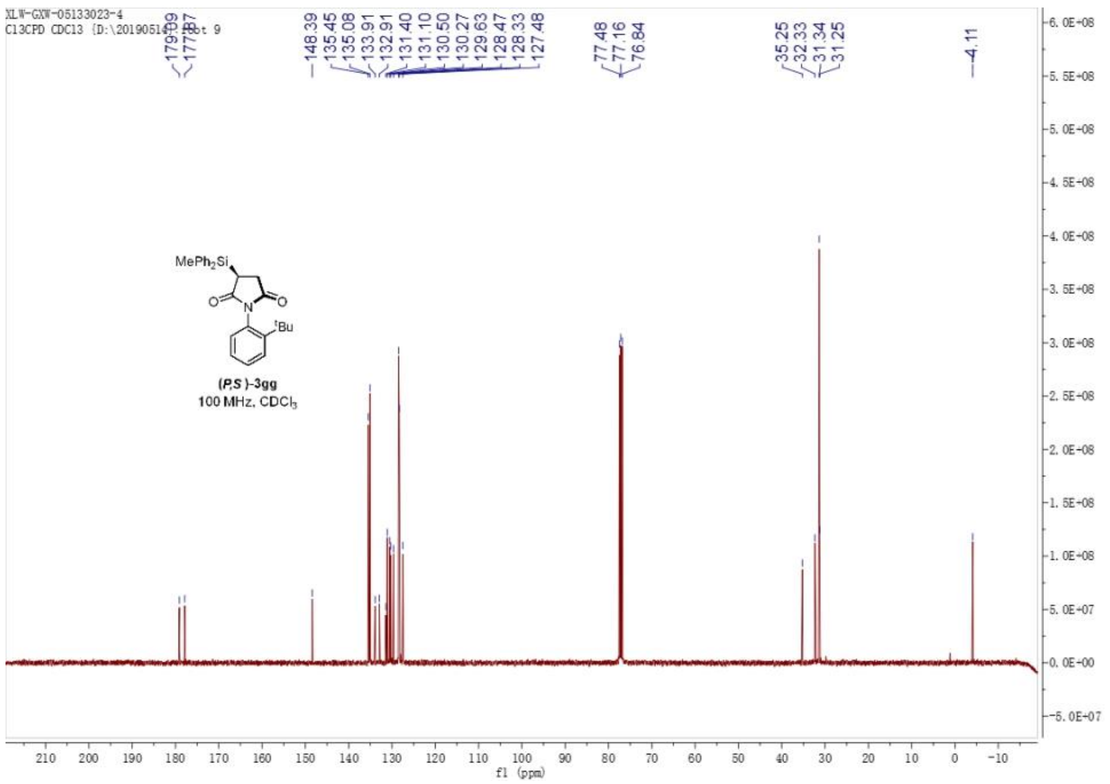

Supplementary Figure 149. ^{13}C NMR spectrum for **3gg**

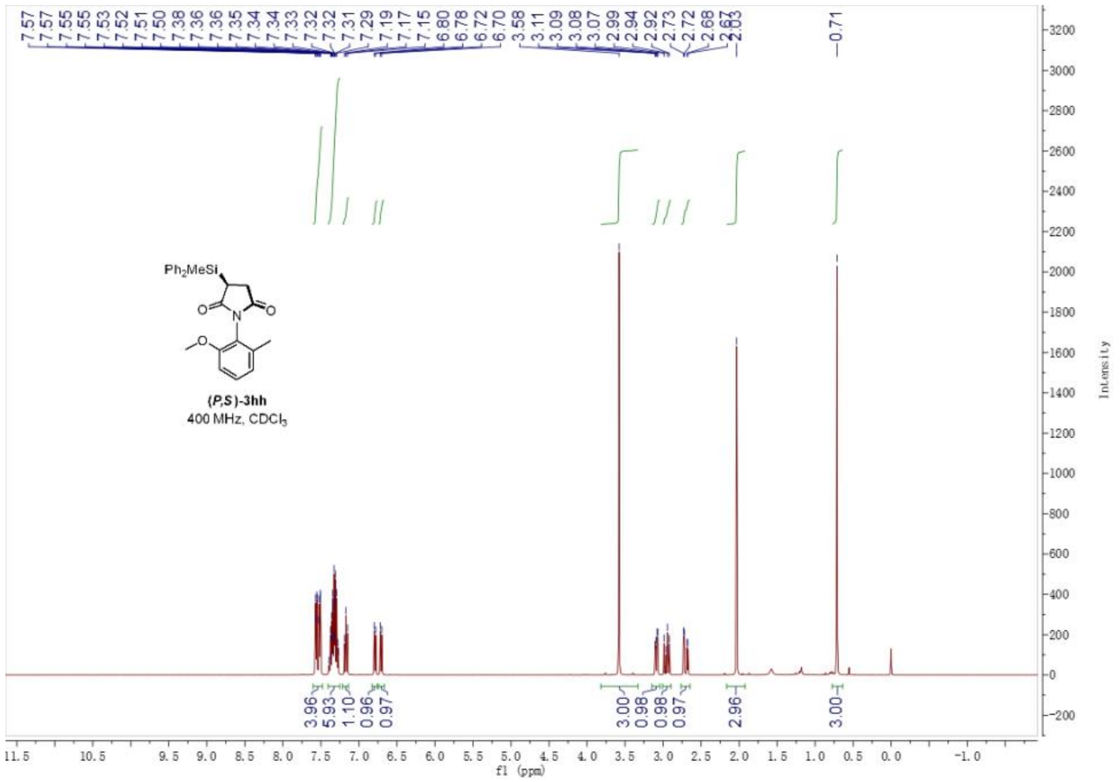

Supplementary Figure 150. ^{1}H NMR spectrum for **3hh**

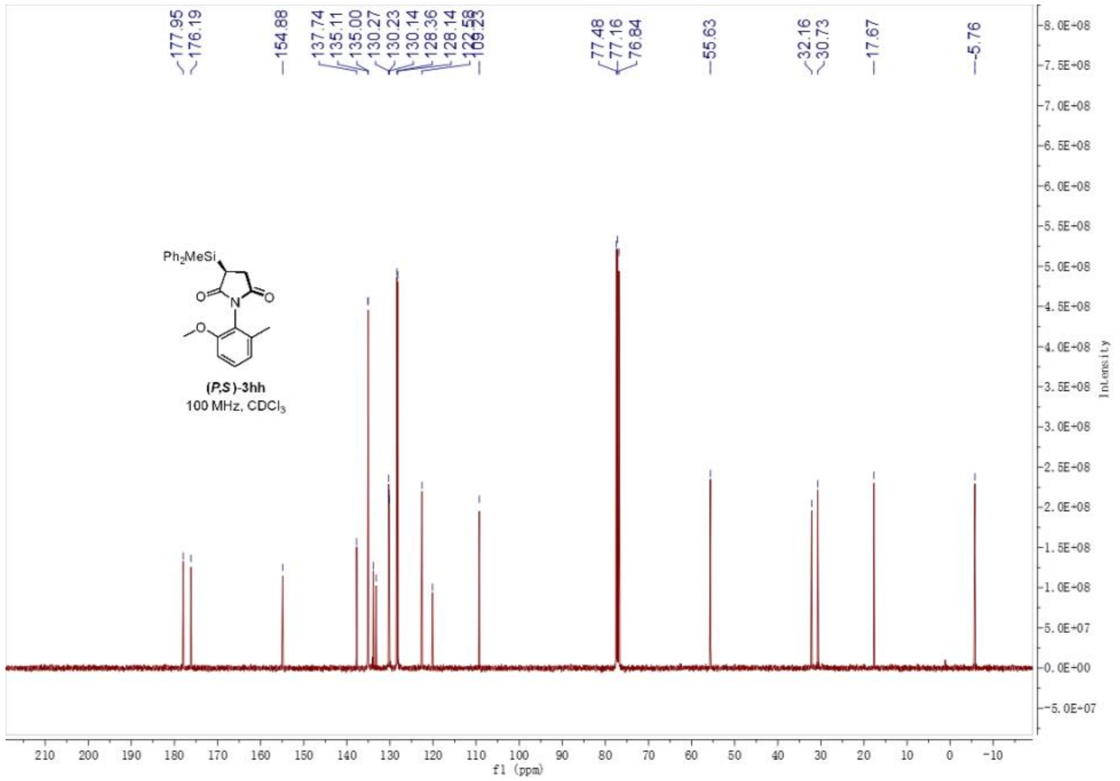

Supplementary Figure 151. ^{13}C NMR spectrum for **3hh**

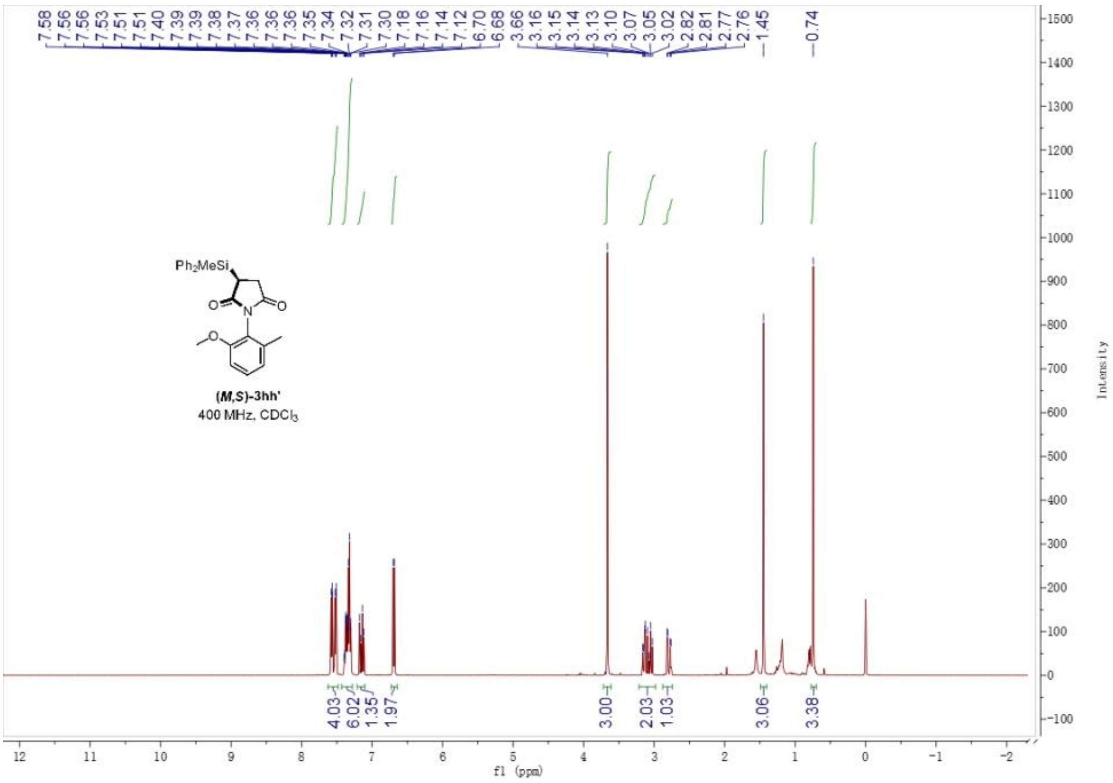

Supplementary Figure 152. ^{1}H NMR spectrum for **3hh'**

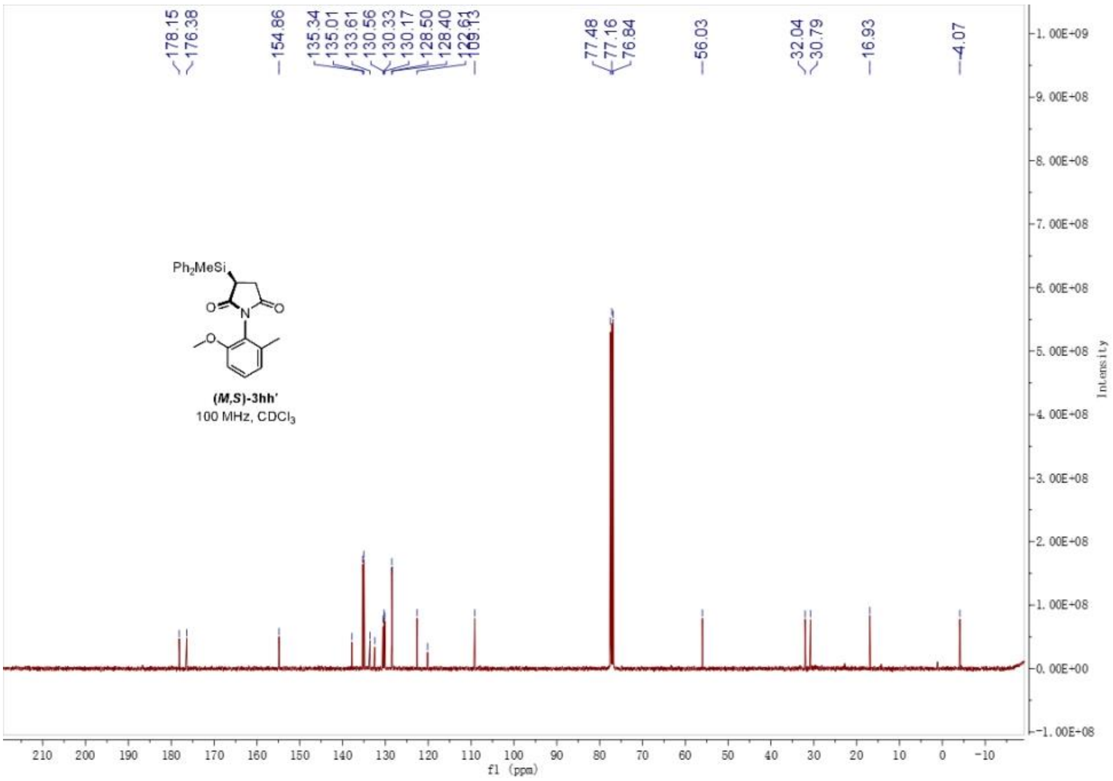

Supplementary Figure 153. ^{13}C NMR spectrum for **3hh'**

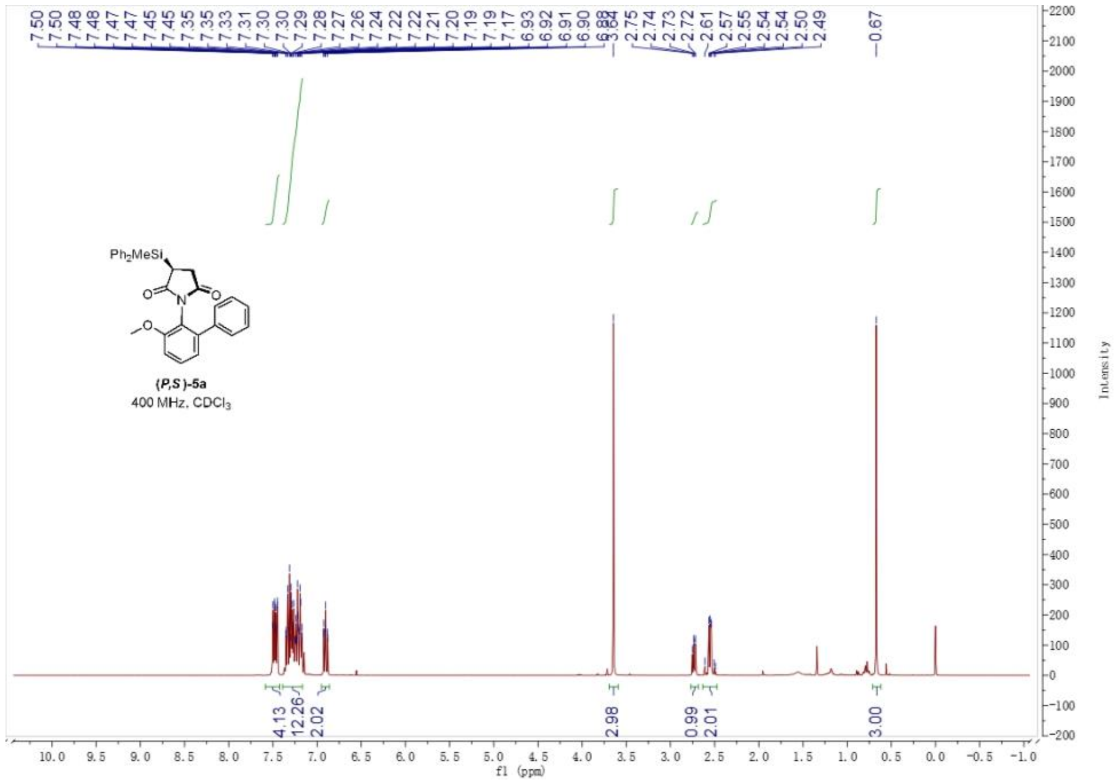

Supplementary Figure 154. ^{1}H NMR spectrum for **5a**

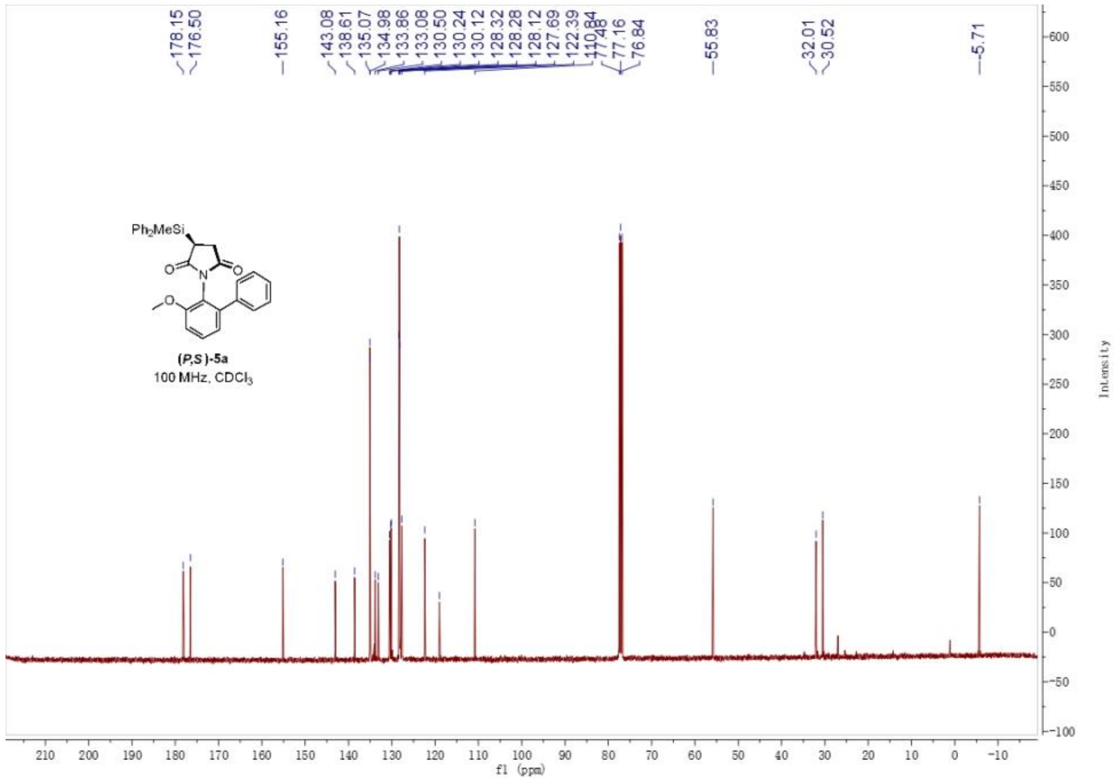

Supplementary Figure 155. ^{13}C NMR spectrum for **5a**

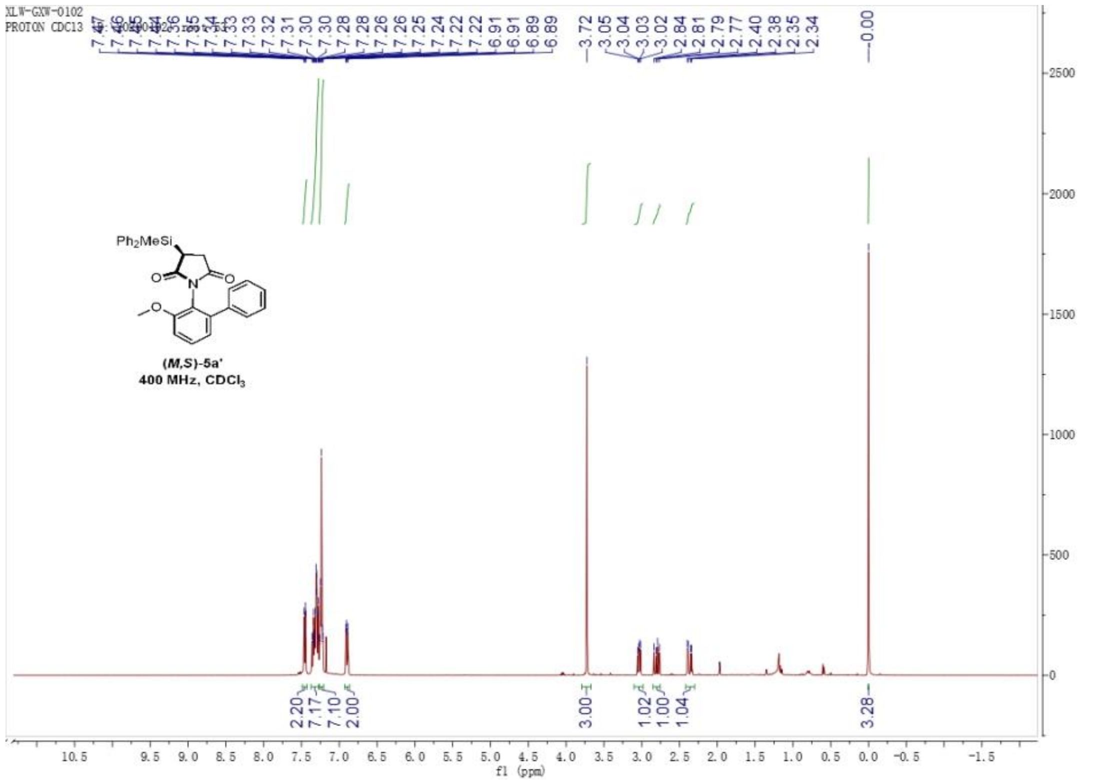

Supplementary Figure 156. ^{1}H NMR spectrum for **5a'**

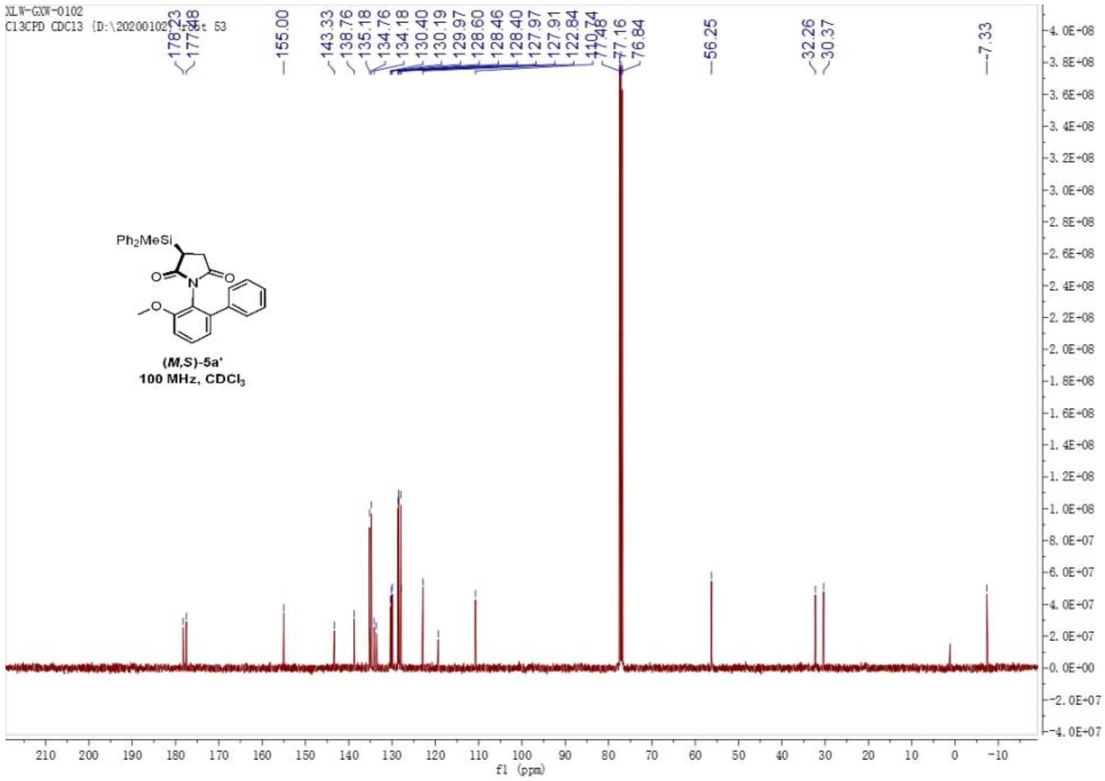

Supplementary Figure 157. ^{13}C NMR spectrum for **5a'**

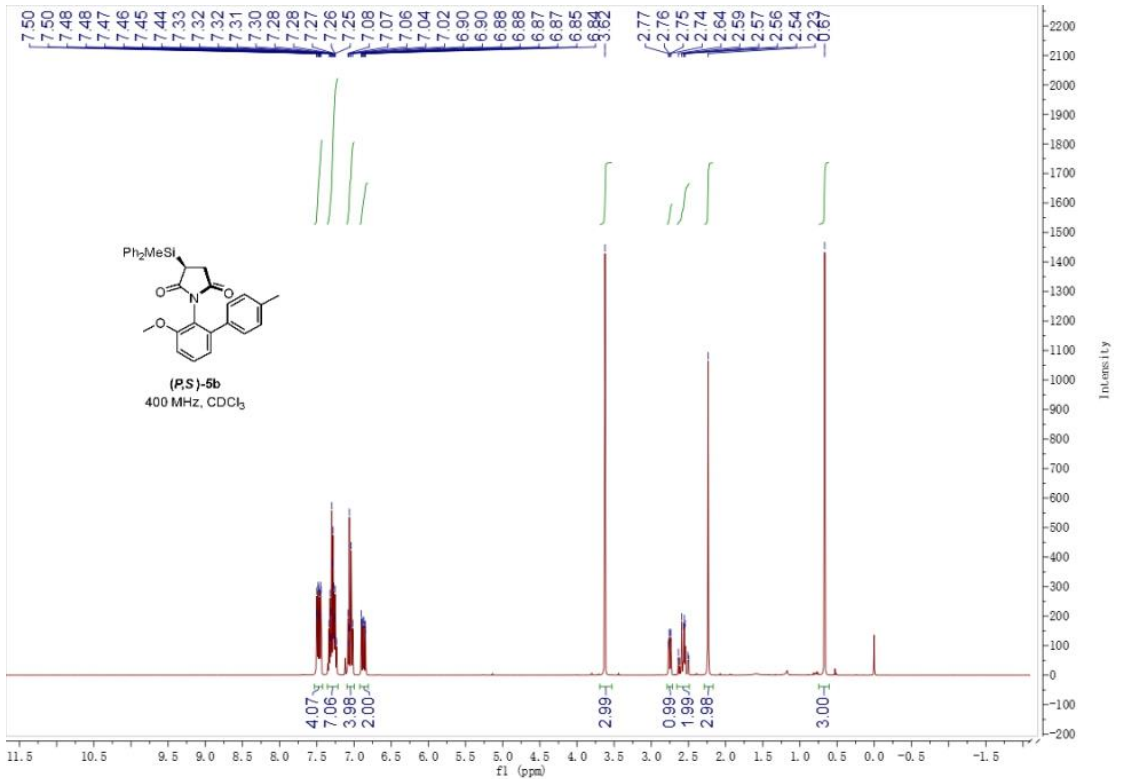

Supplementary Figure 158. ^{1}H NMR spectrum for **5b**

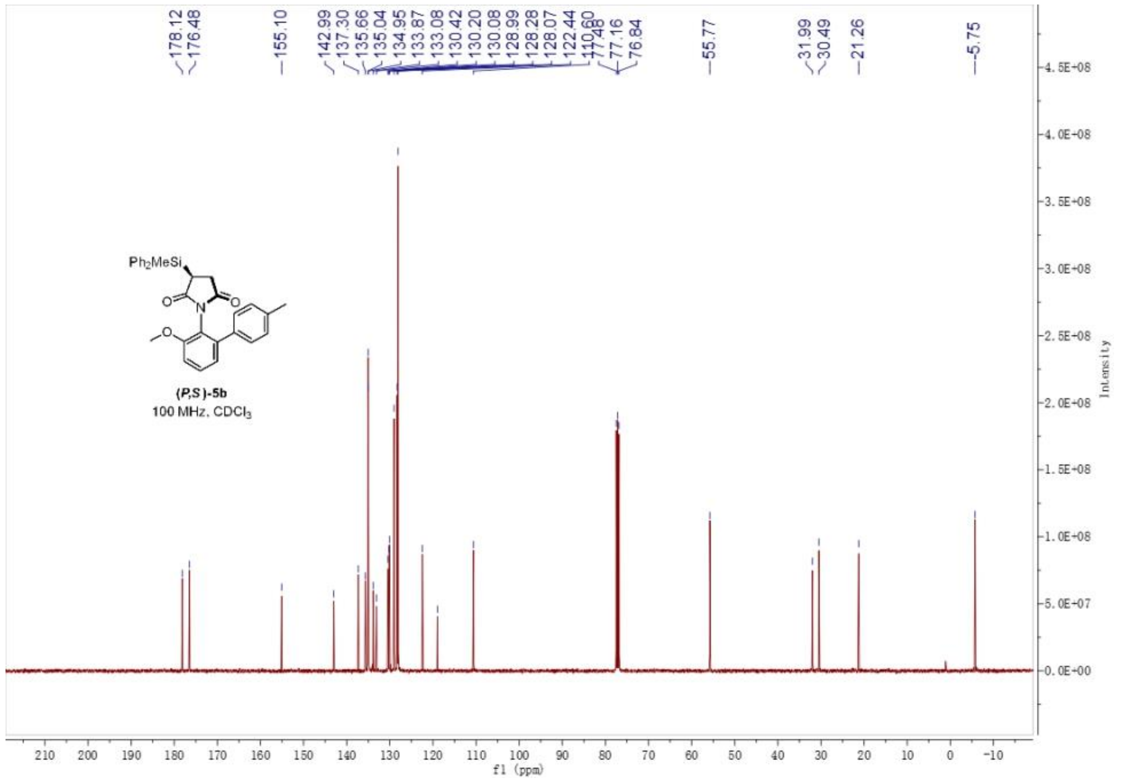

Supplementary Figure 159. ^{13}C NMR spectrum for **5b**

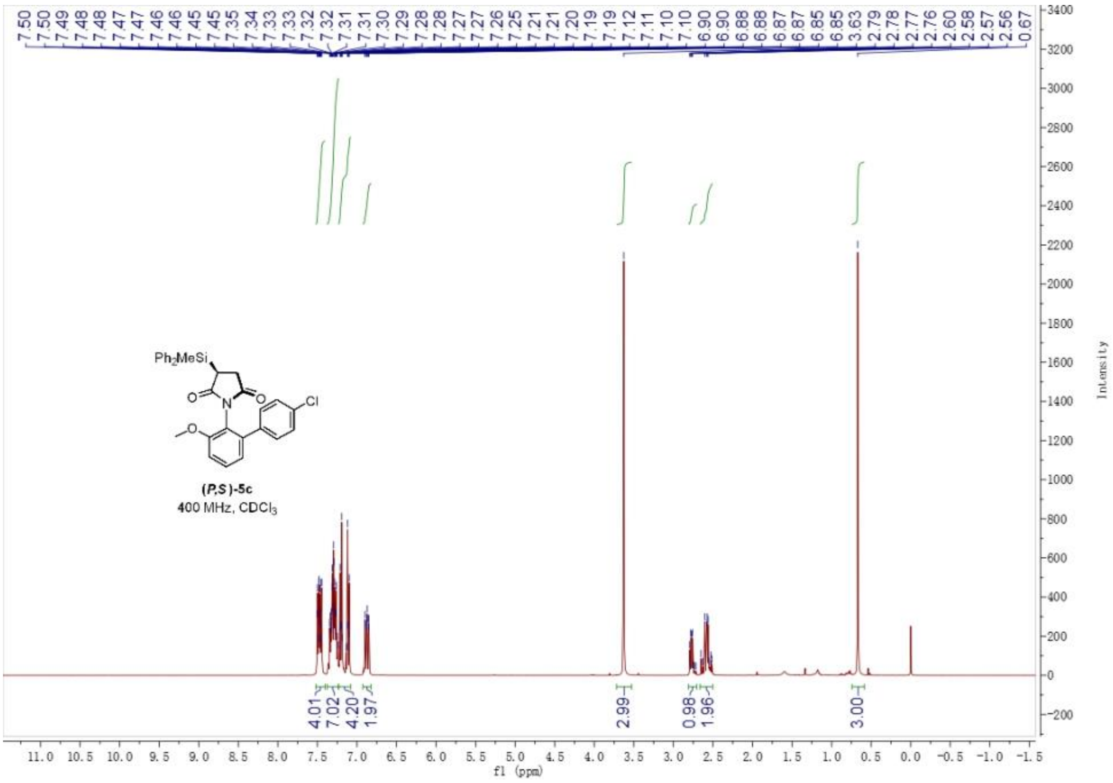

Supplementary Figure 160. ^{1}H NMR spectrum for **5c**

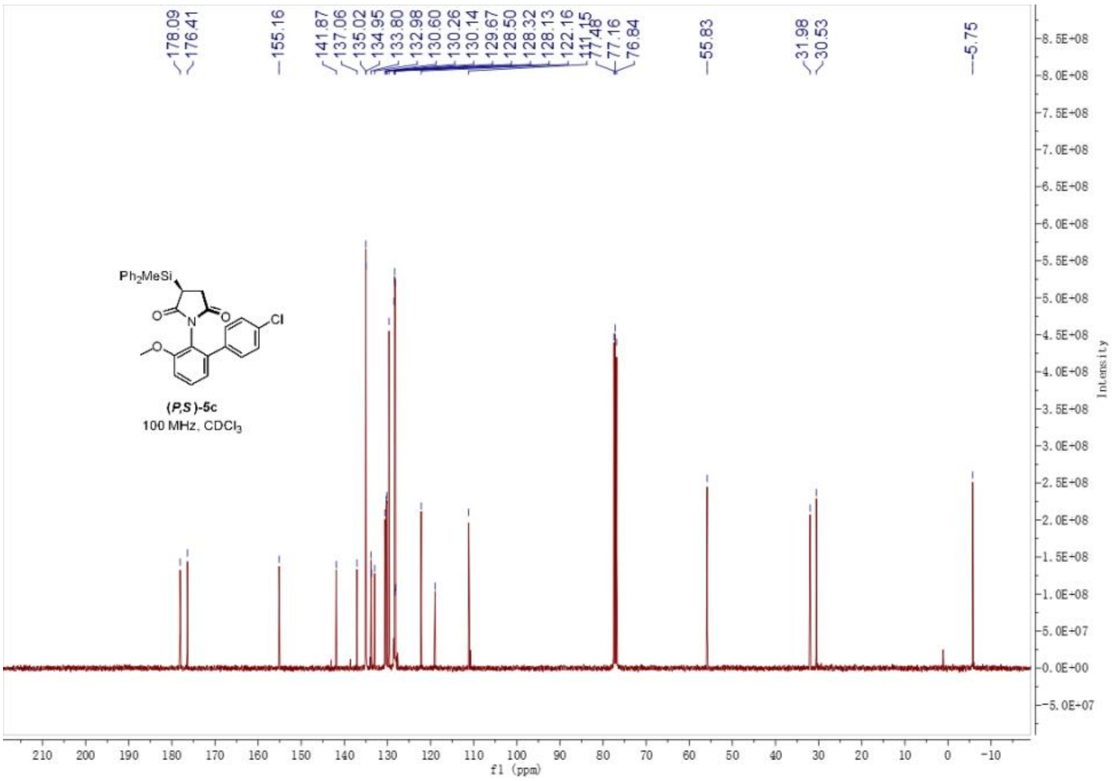

Supplementary Figure 161. ^{13}C NMR spectrum for **5c**

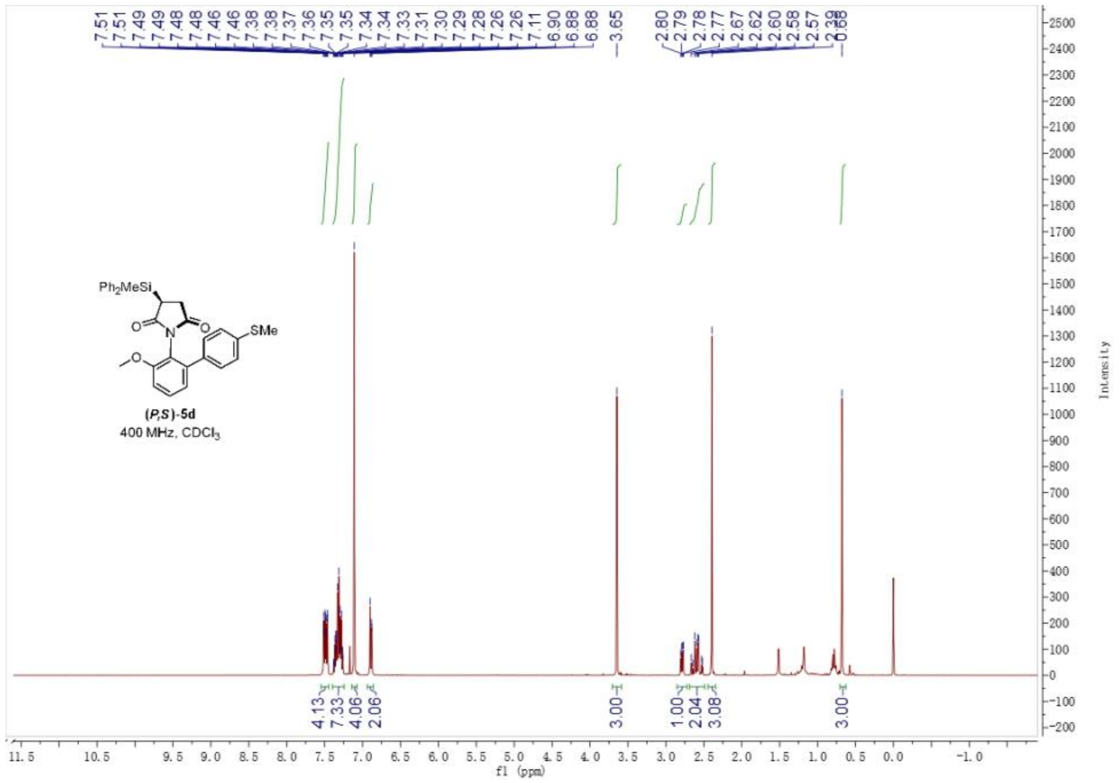

Supplementary Figure 162. ^{1}H NMR spectrum for **5d**

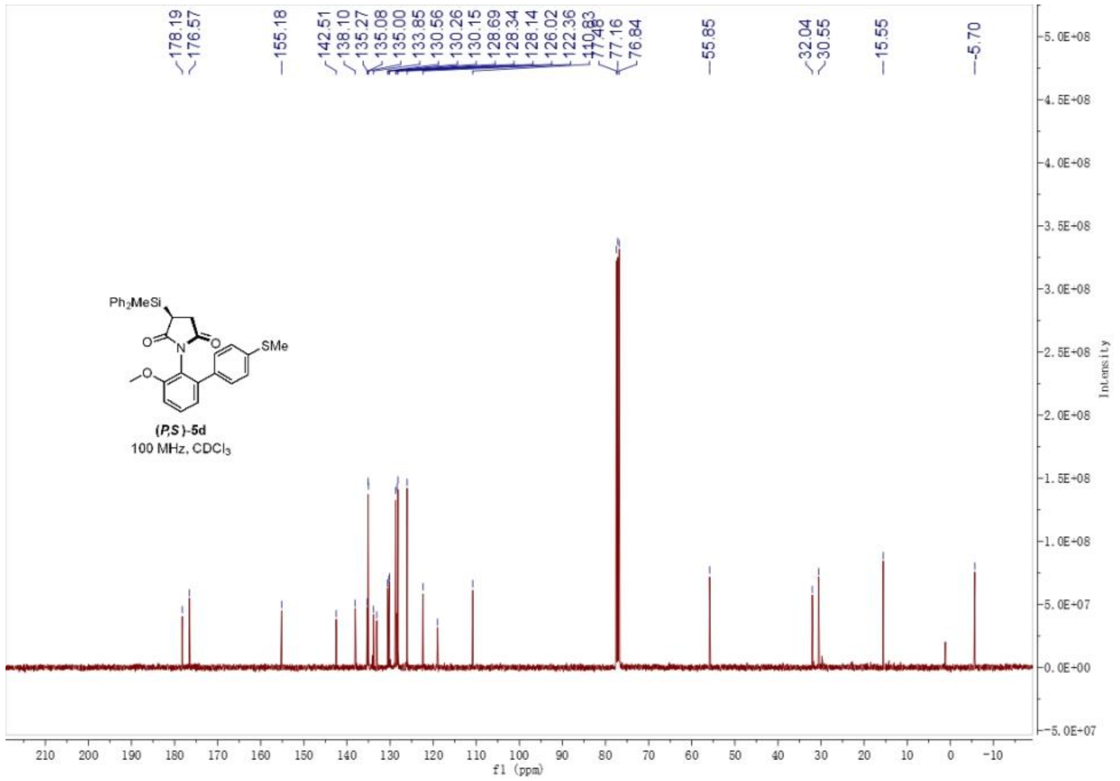

Supplementary Figure 163. ^{13}C NMR spectrum for **5d**

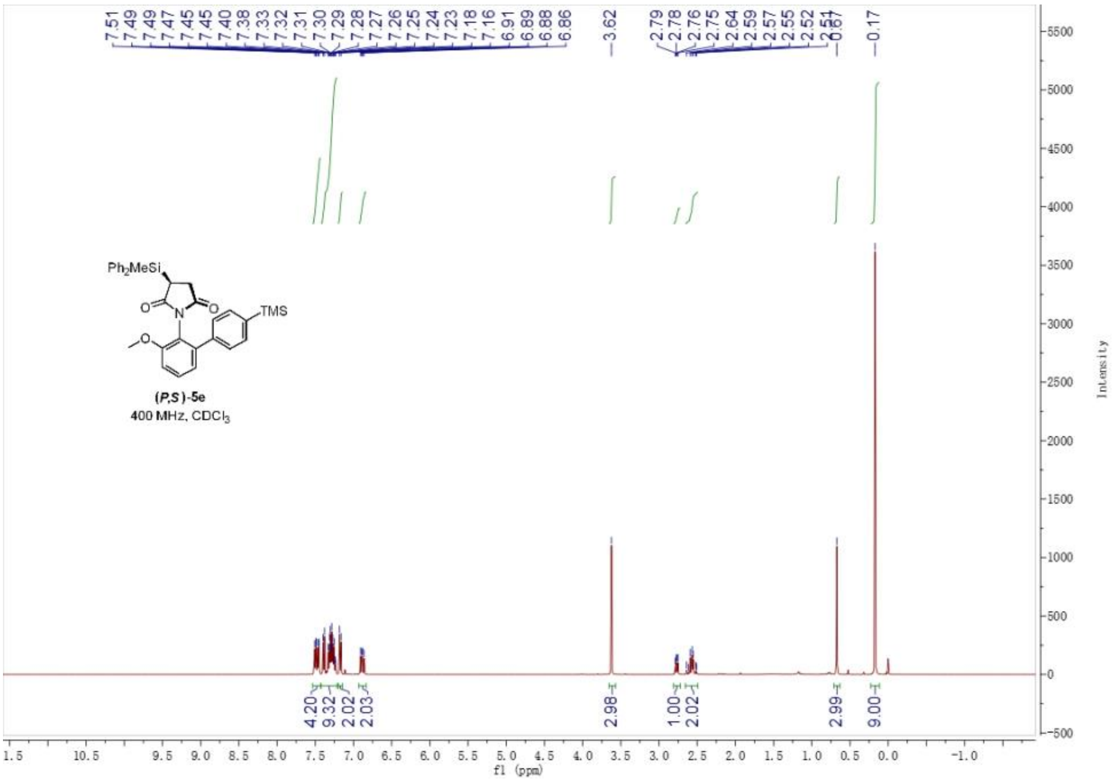

Supplementary Figure 164. ^{1}H NMR spectrum for **5e**

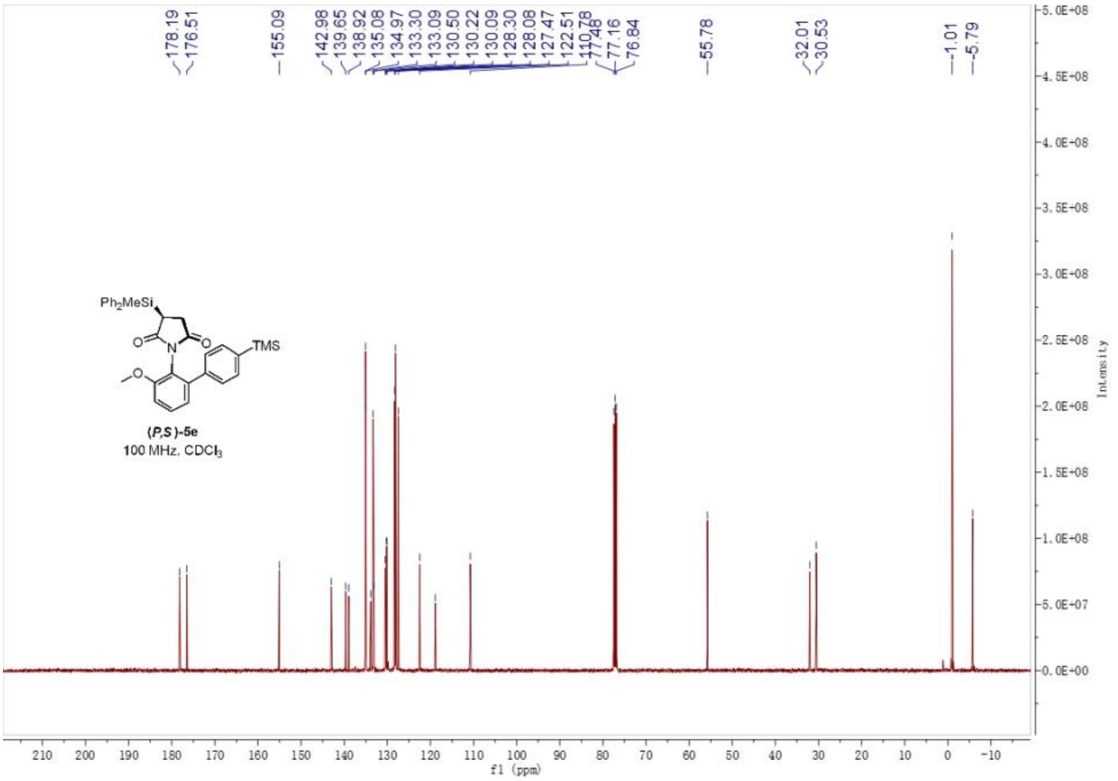

Supplementary Figure 165. ^{13}C NMR spectrum for **5e**

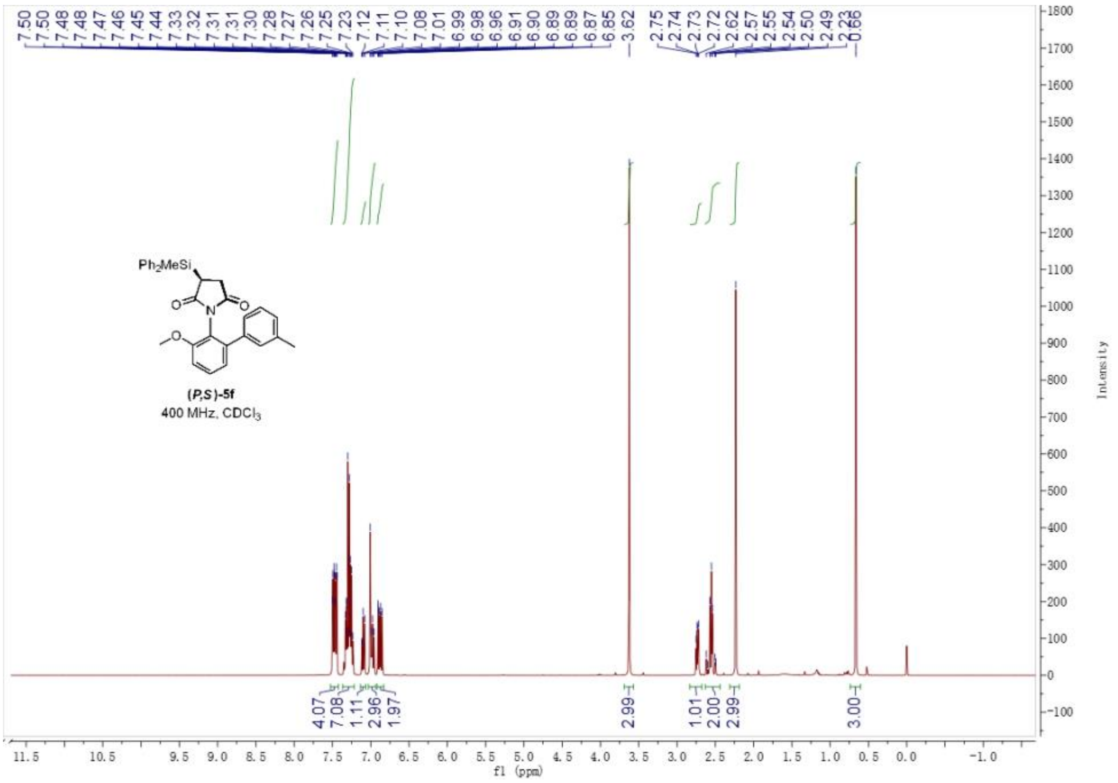

Supplementary Figure 166. ^{1}H NMR spectrum for **5f**

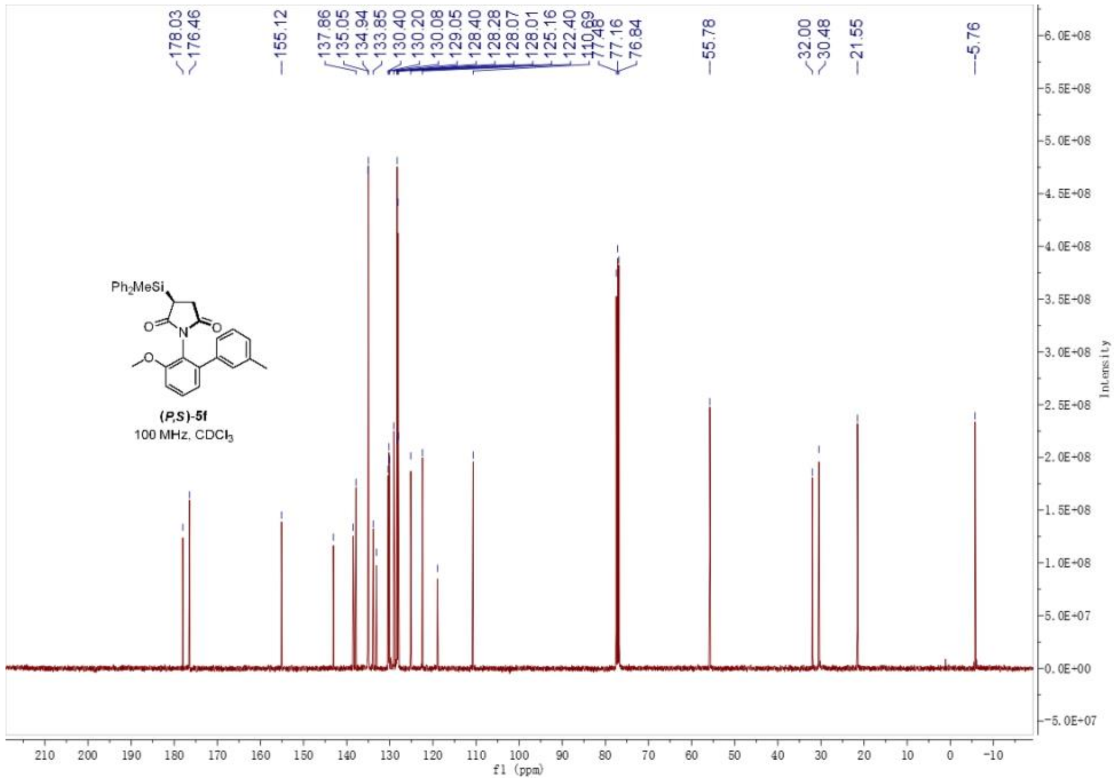

Supplementary Figure 167. ^{13}C NMR spectrum for **5f**

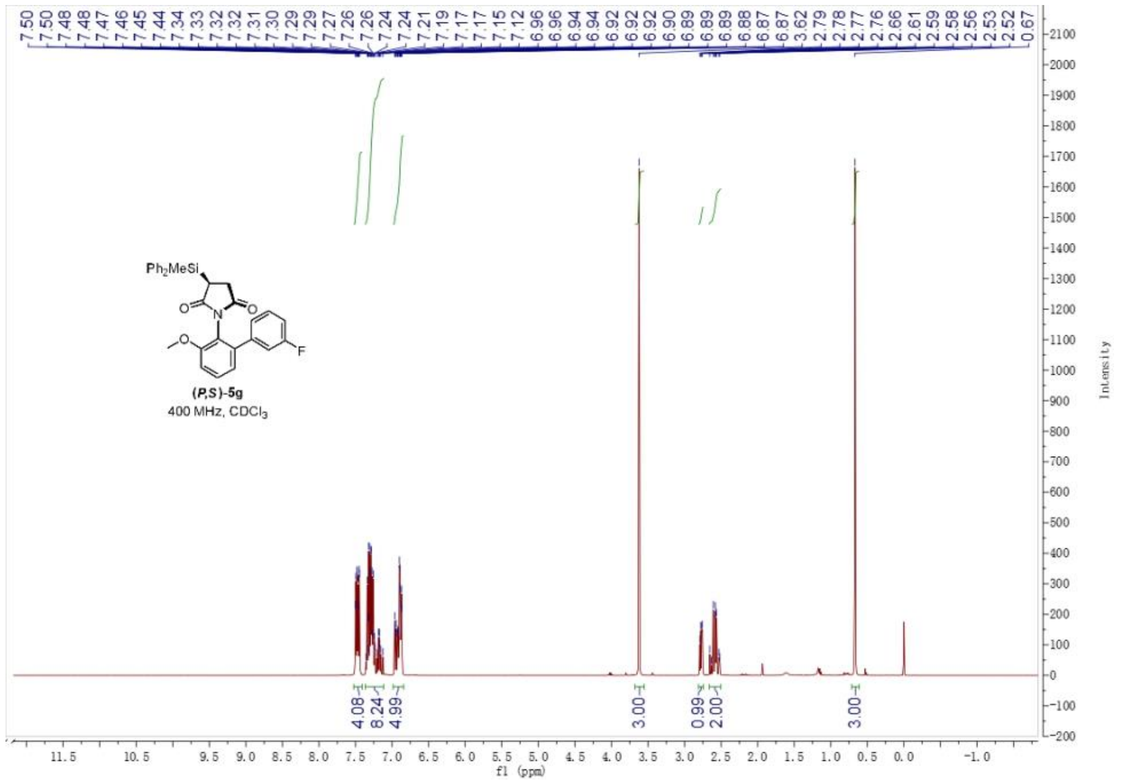

Supplementary Figure 168. ^{1}H NMR spectrum for **5g**

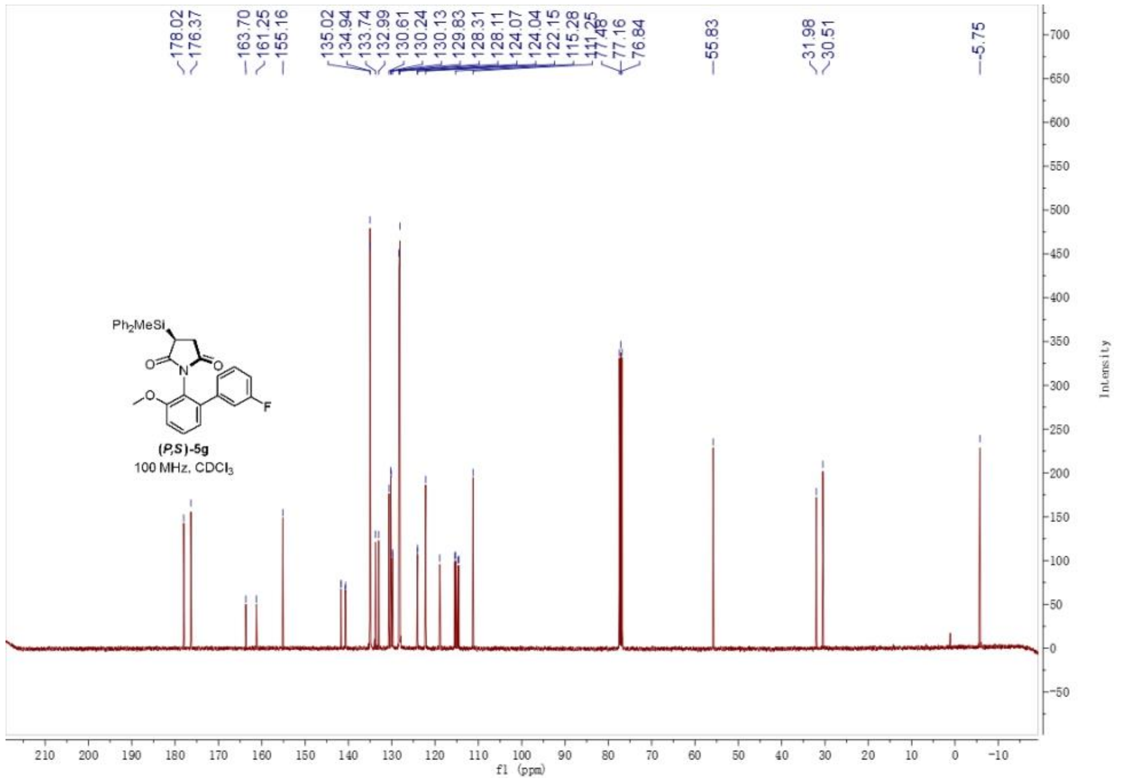

Supplementary Figure 169. ^{13}C NMR spectrum for **5g**

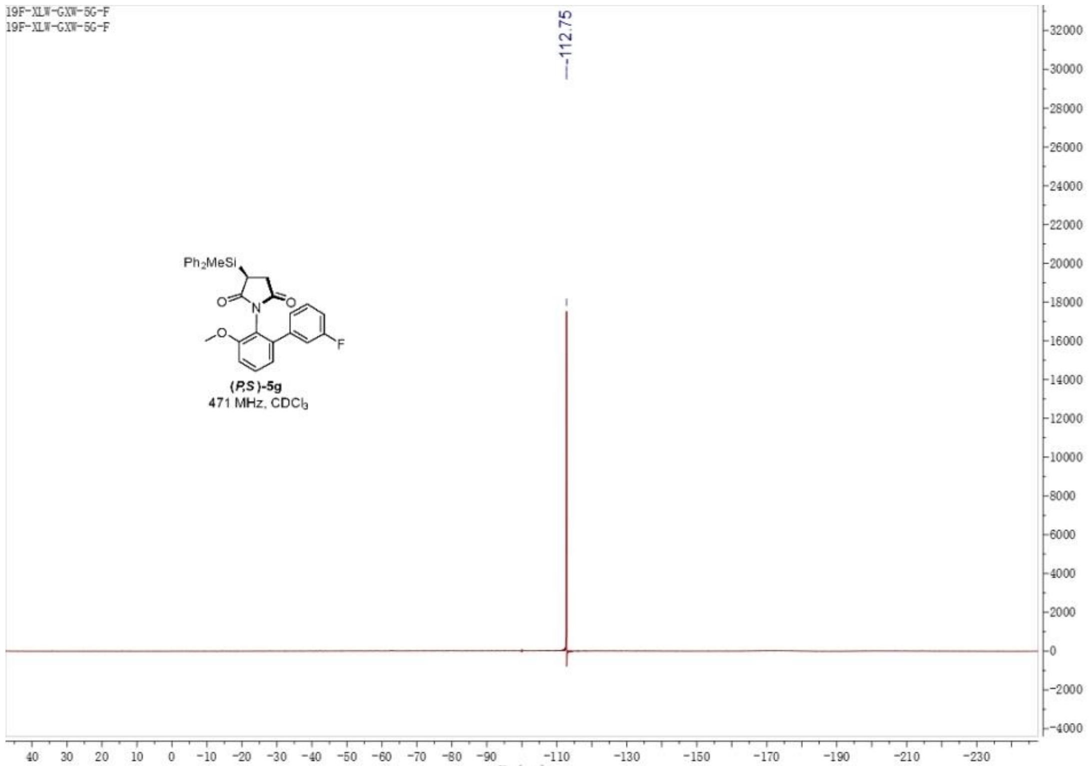

Supplementary Figure 170. ^{19}F NMR spectrum for **5g**

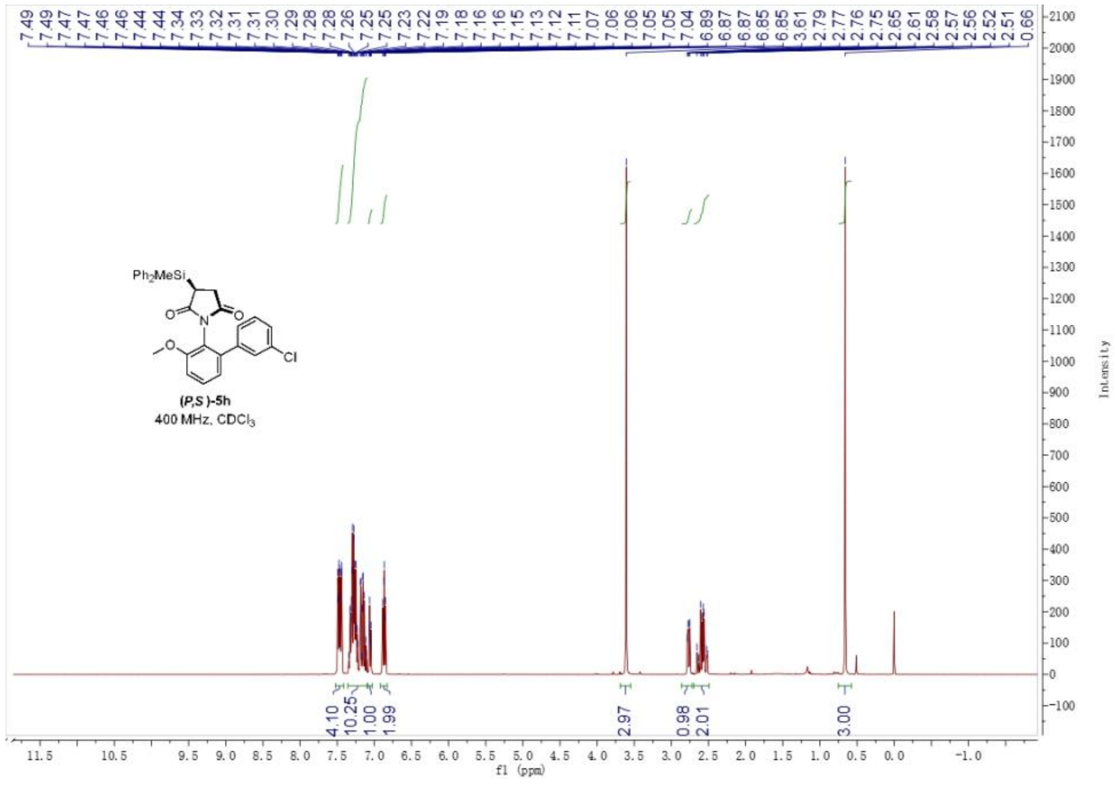

Supplementary Figure 171. ^{1}H NMR spectrum for **5h**

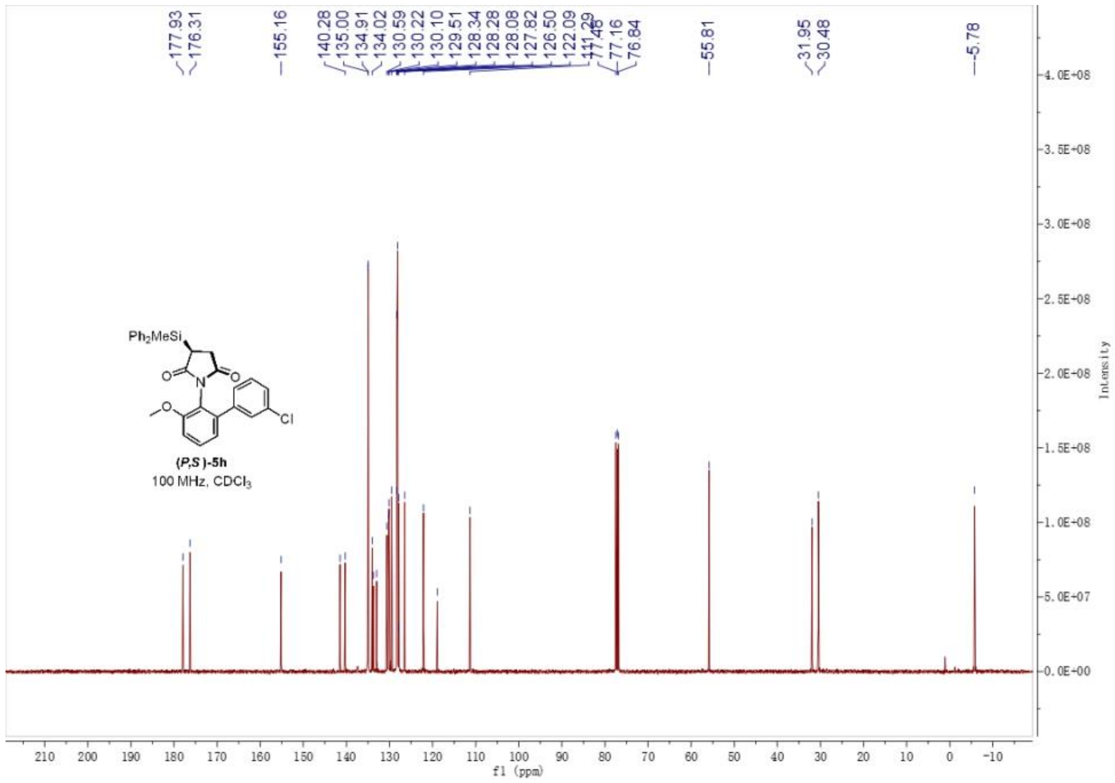

Supplementary Figure 172. ^{13}C NMR spectrum for **5h**

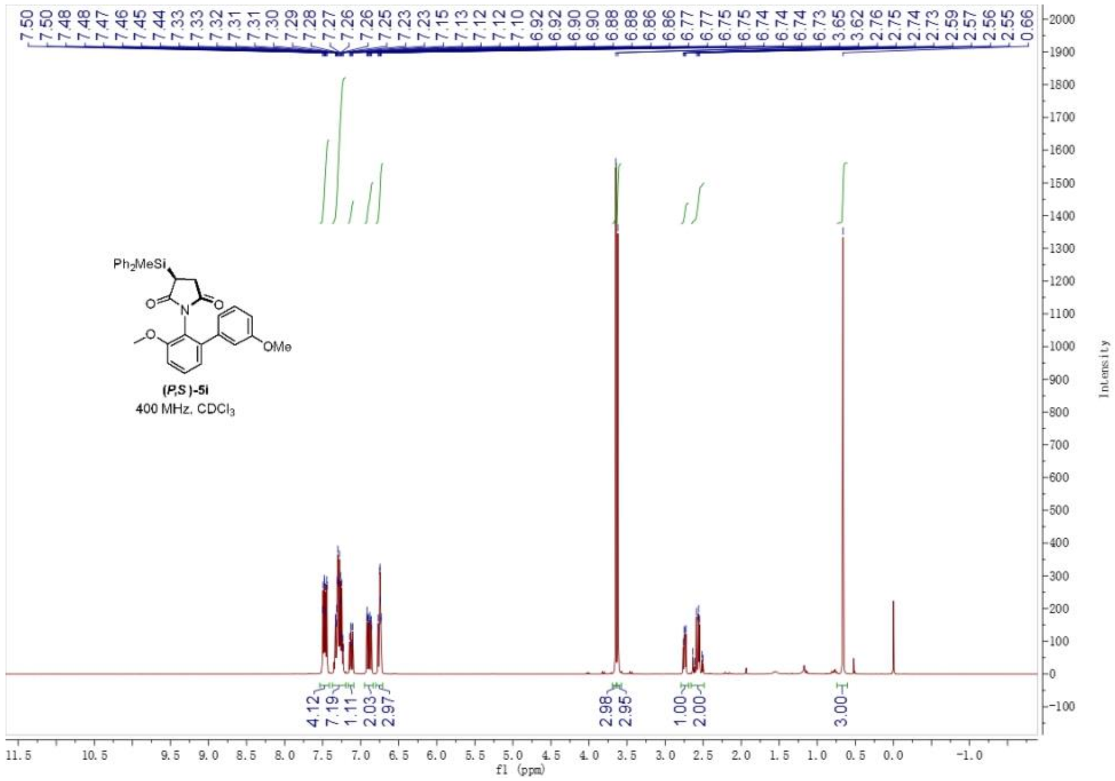

Supplementary Figure 173. ^{1}H NMR spectrum for **5i**

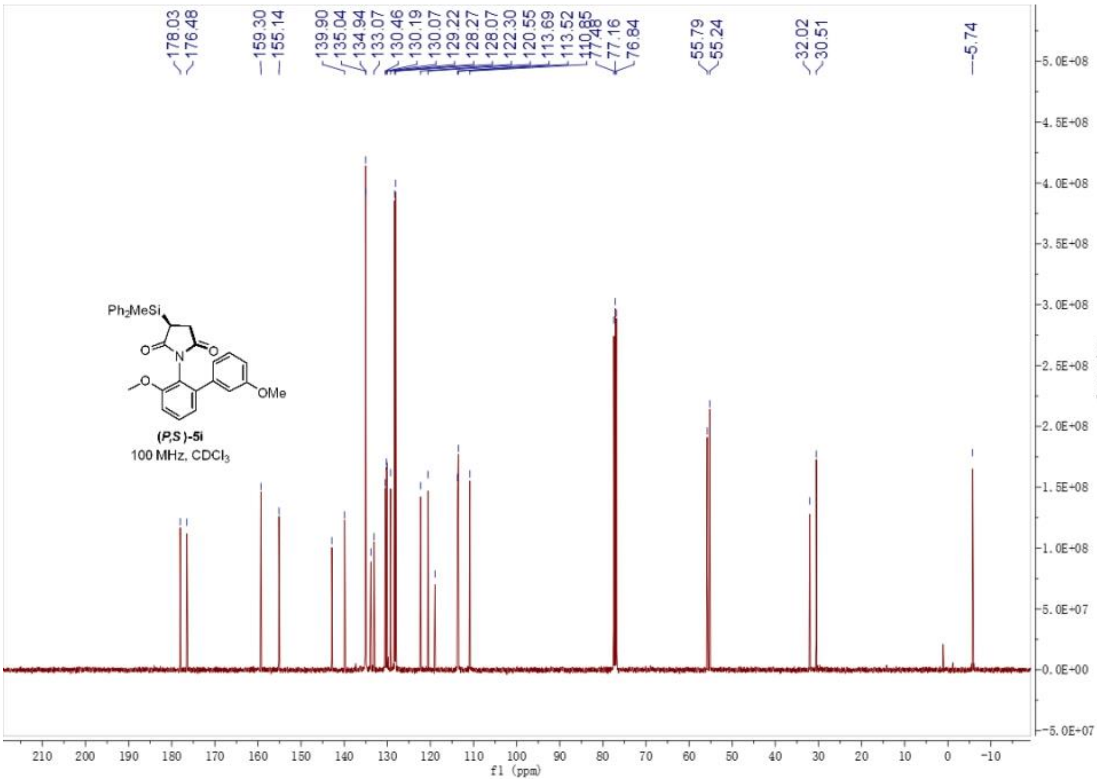

Supplementary Figure 174. ^{13}C NMR spectrum for **5i**

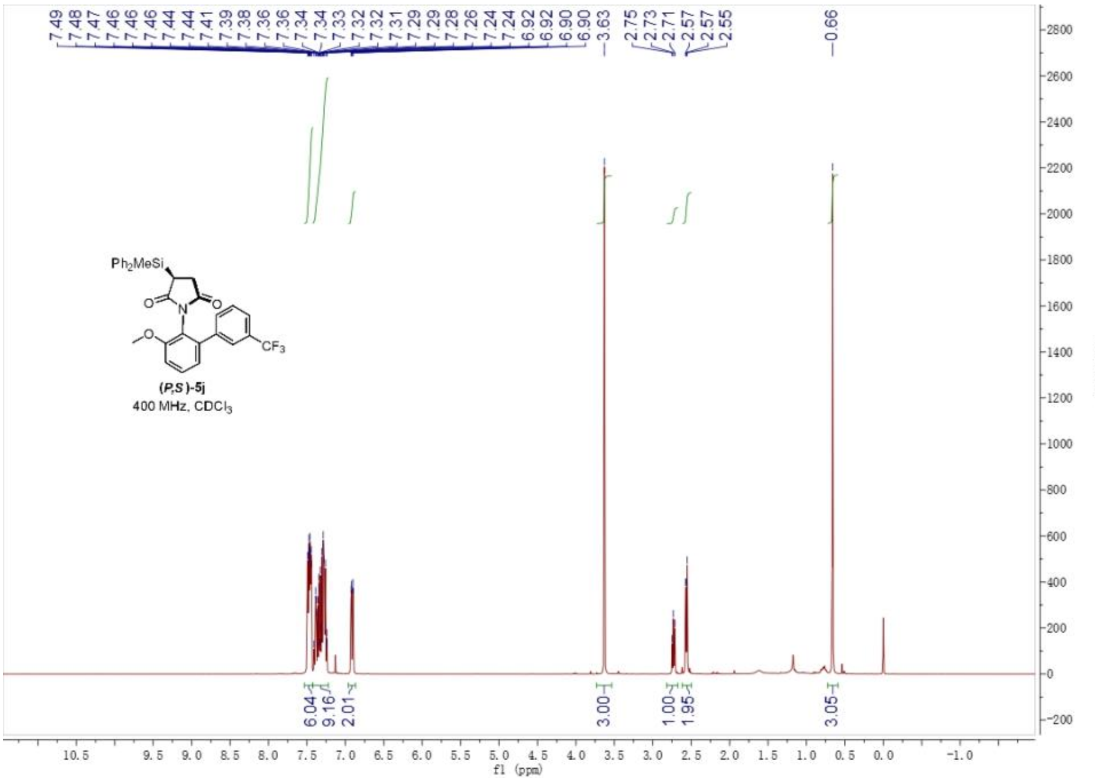

Supplementary Figure 175. ^{1}H NMR spectrum for **5j**

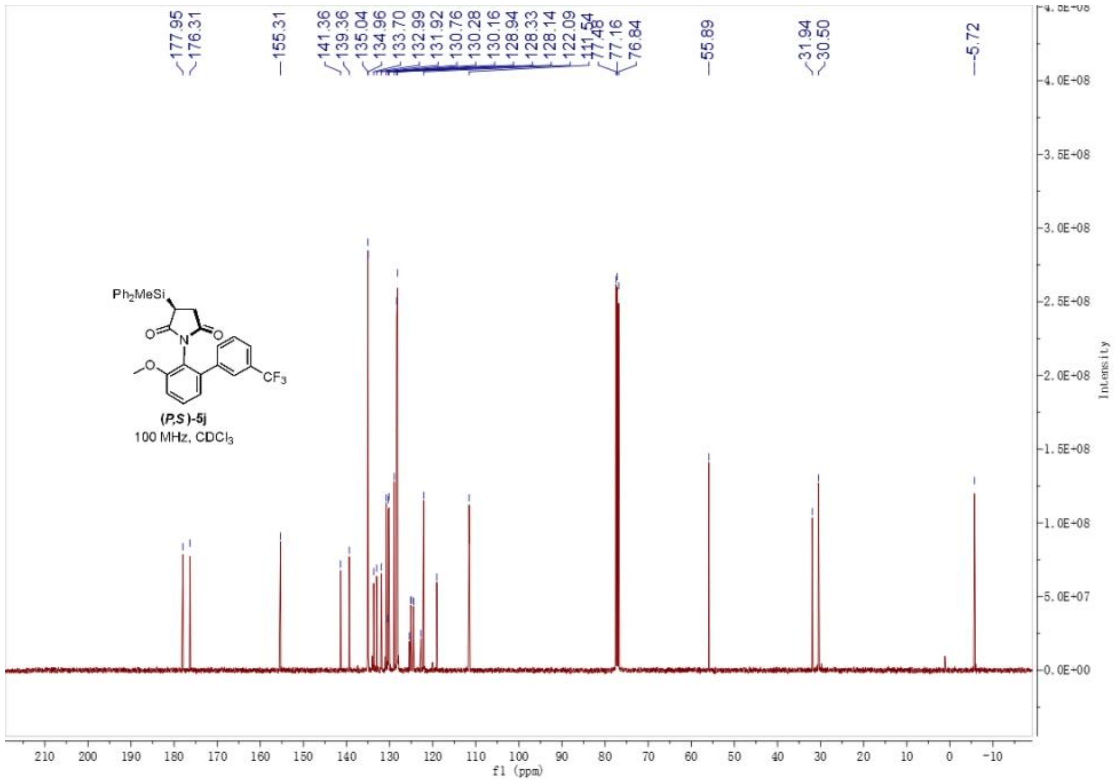

Supplementary Figure 176. ^{13}C NMR spectrum for **5j**

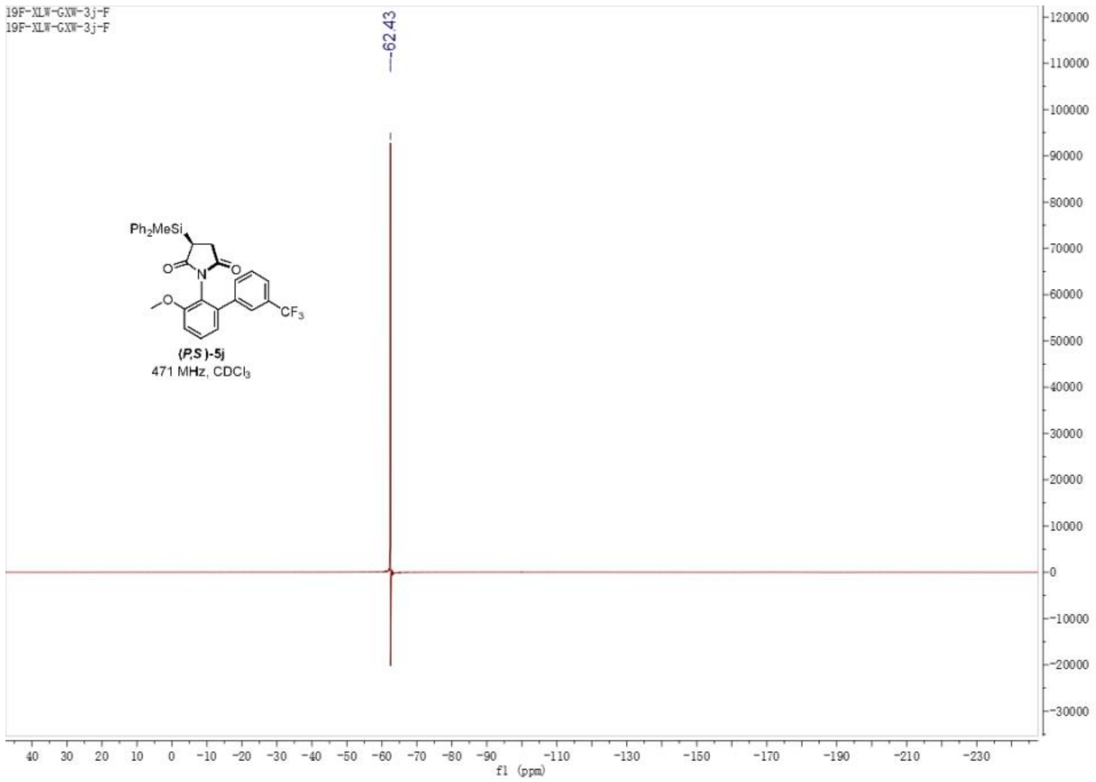

Supplementary Figure 177. ^{19}F NMR spectrum for **5j**

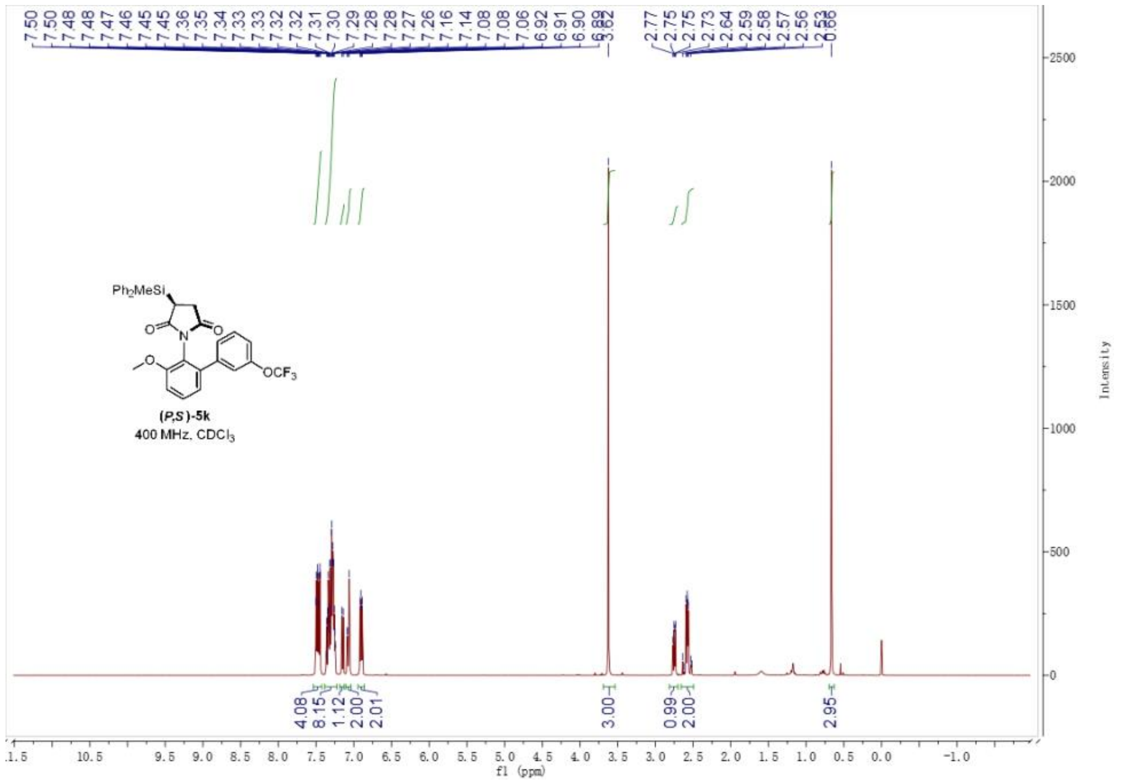

Supplementary Figure 178. ^{1}H NMR spectrum for **5k**

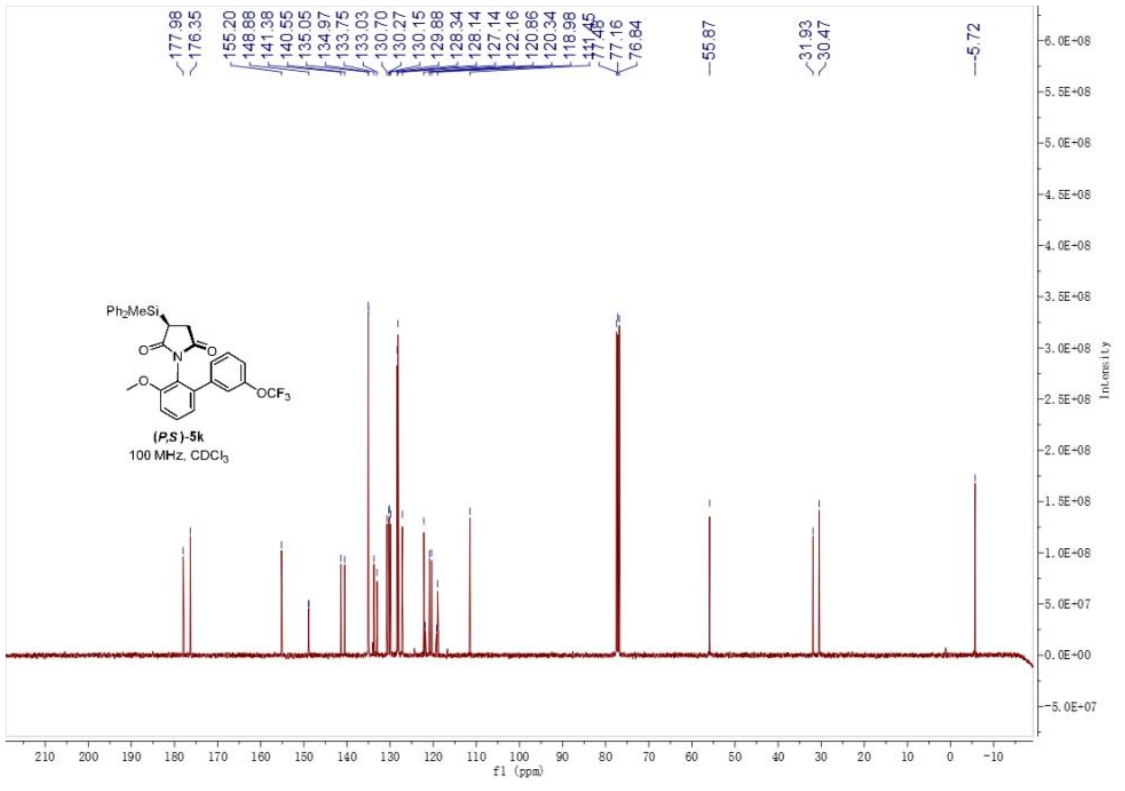

Supplementary Figure 179. ^{13}C NMR spectrum for **5k**

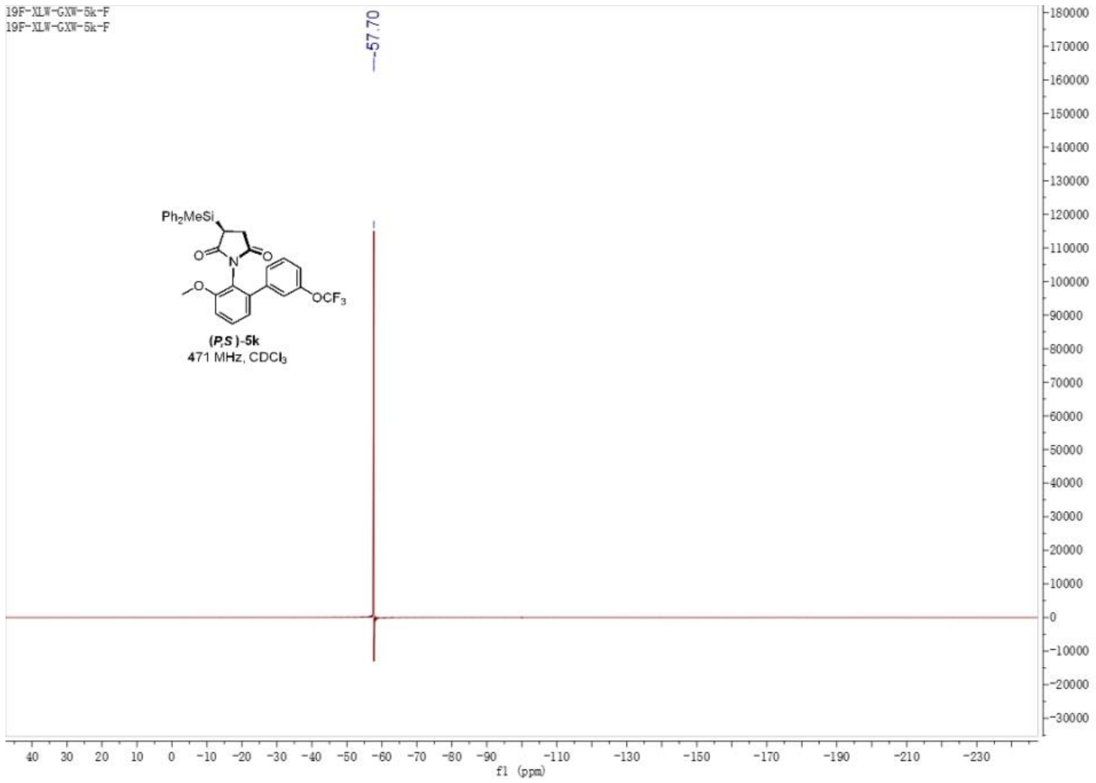

Supplementary Figure 180. ^{19}F NMR spectrum for **5k**

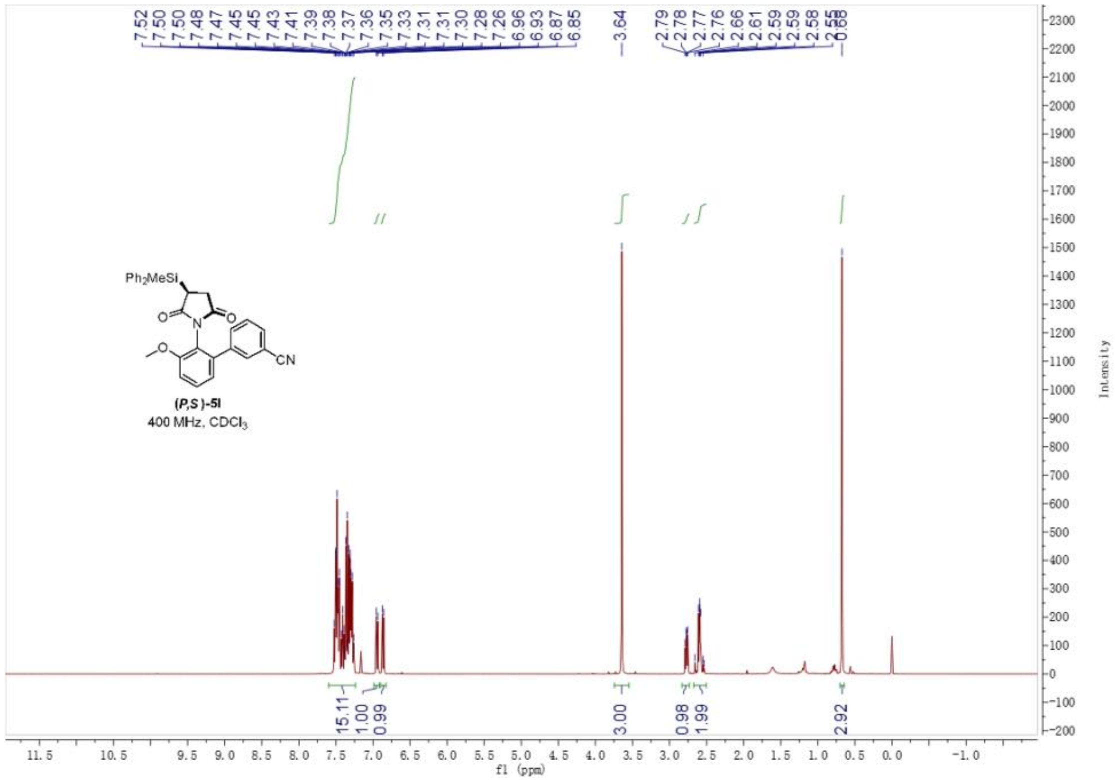

Supplementary Figure 181. ^{1}H NMR spectrum for **5l**

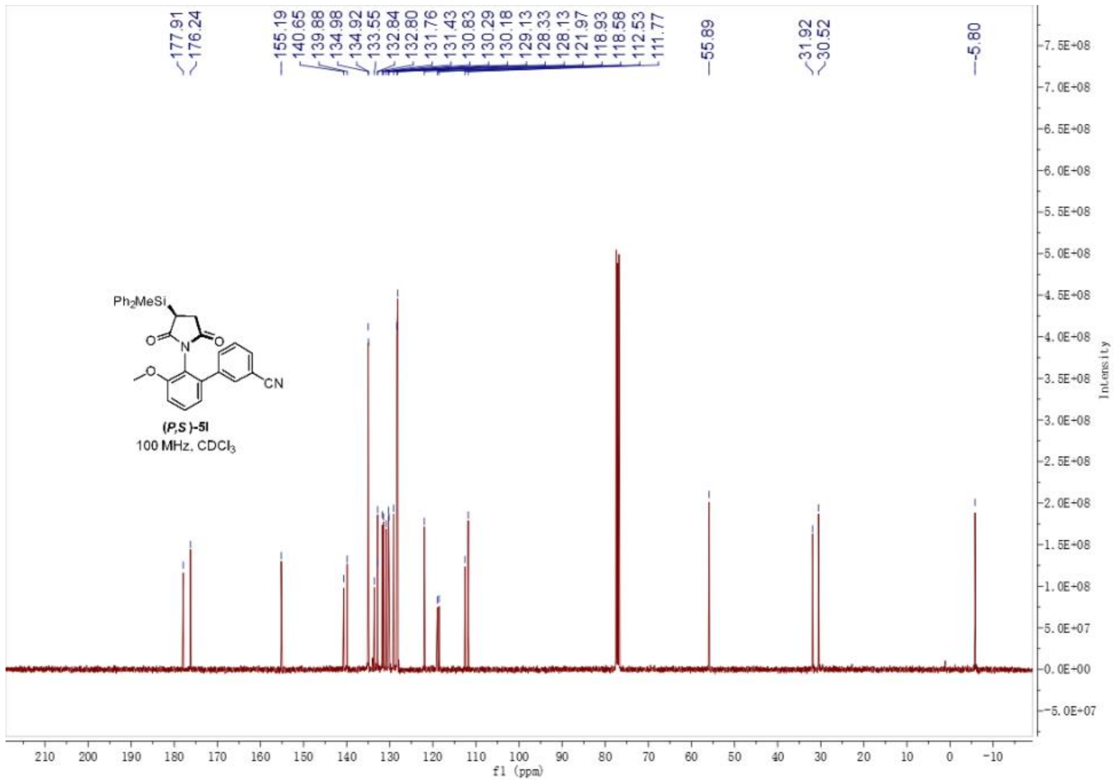

Supplementary Figure 182. ^{13}C NMR spectrum for **5l**

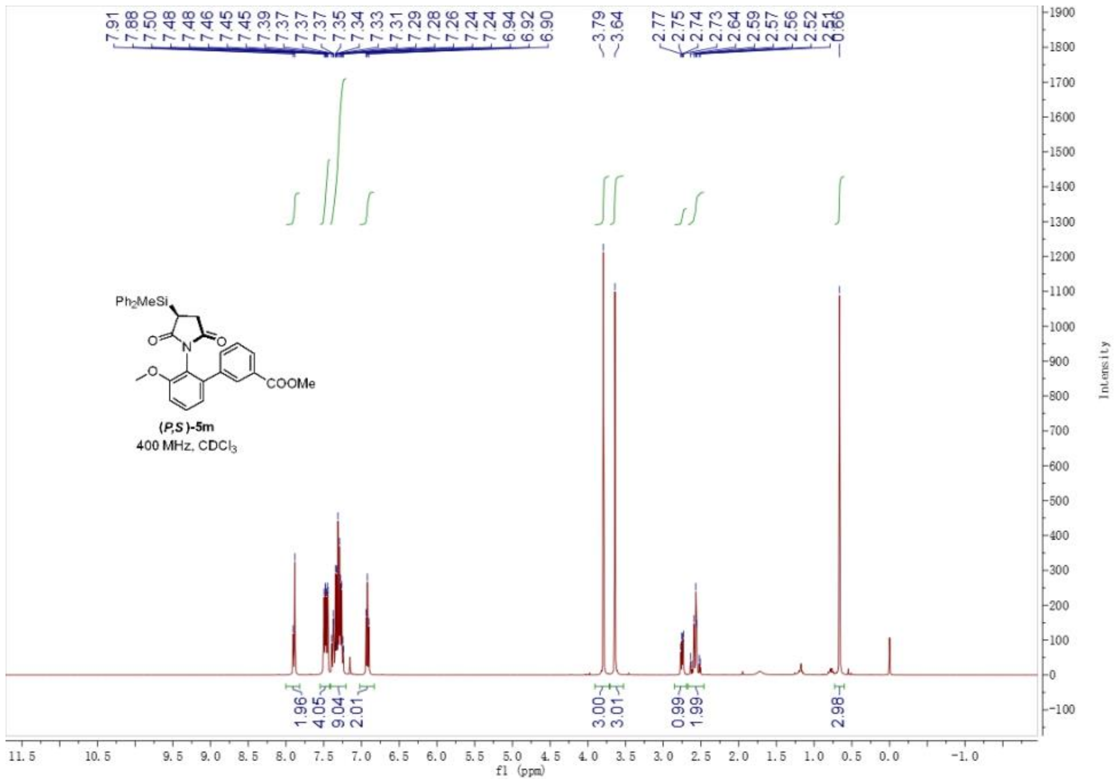

Supplementary Figure 183. ^{1}H NMR spectrum for **5m**

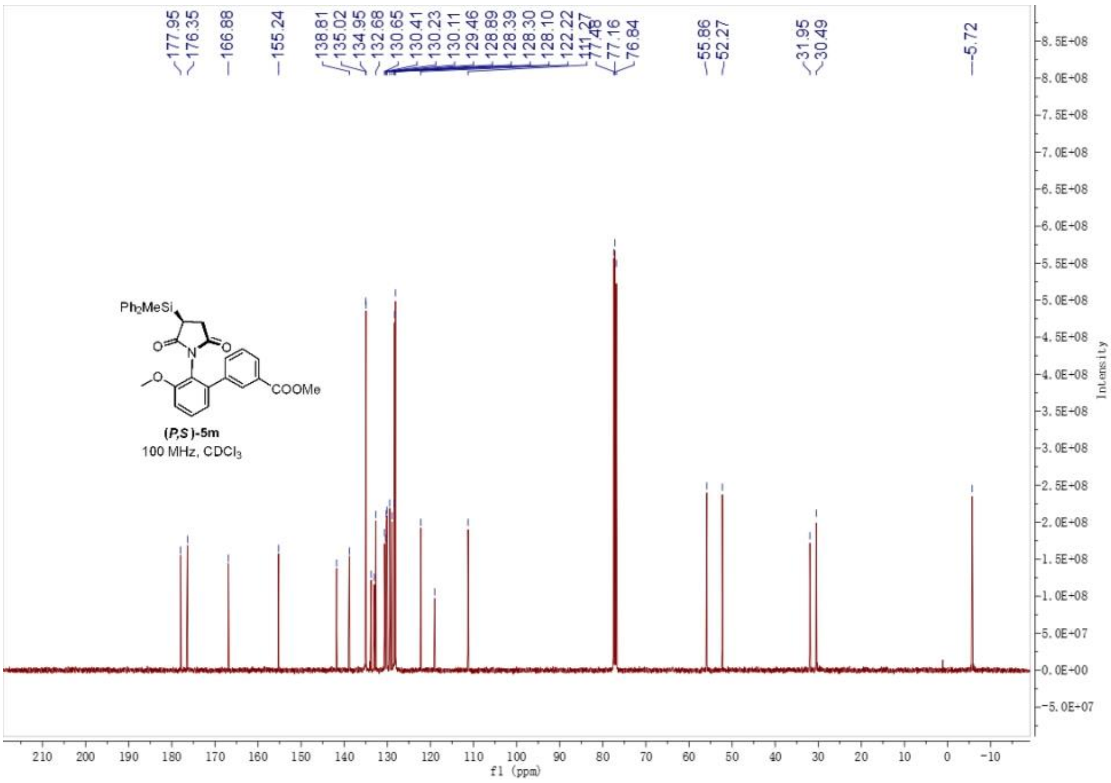

Supplementary Figure 184. ^{13}C NMR spectrum for **5m**

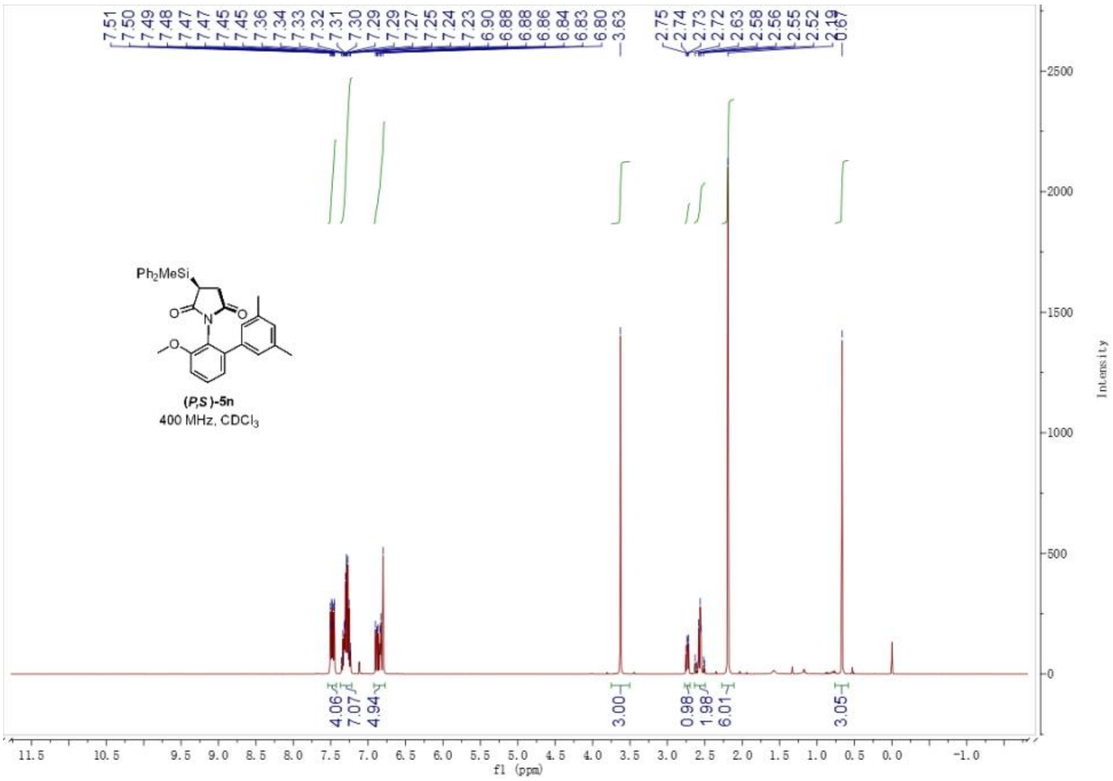

Supplementary Figure 185. ^{1}H NMR spectrum for **5n**

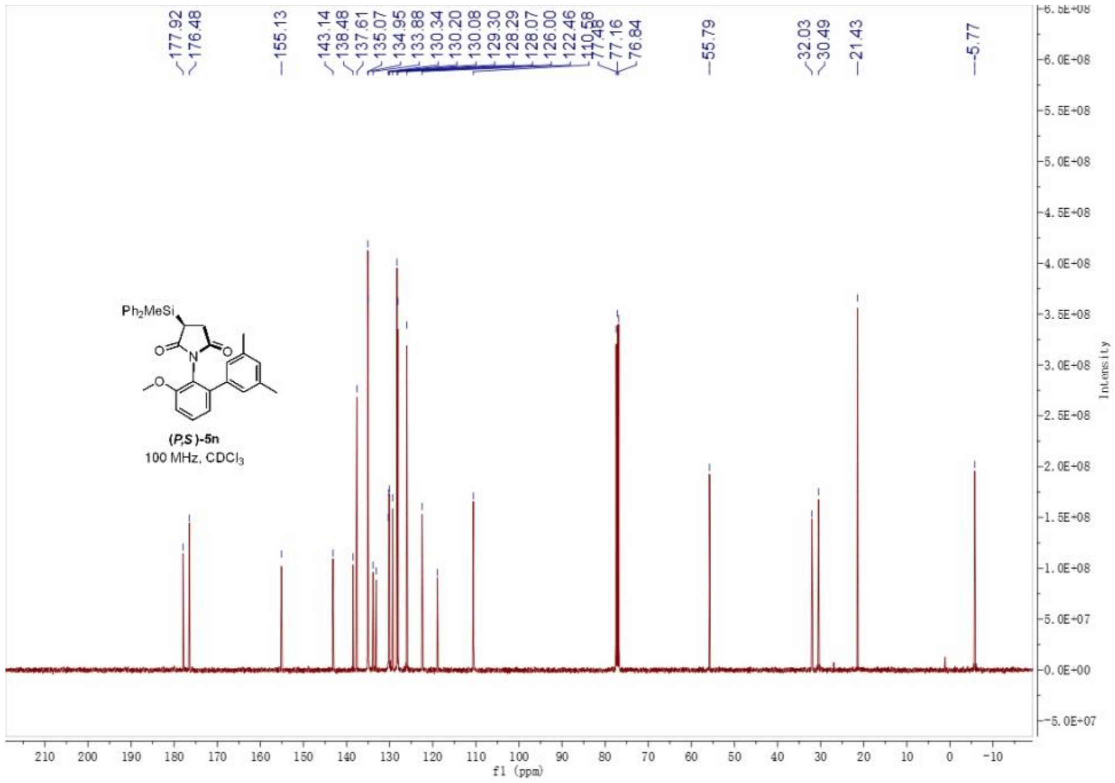

Supplementary Figure 186. ^{13}C NMR spectrum for **5n**

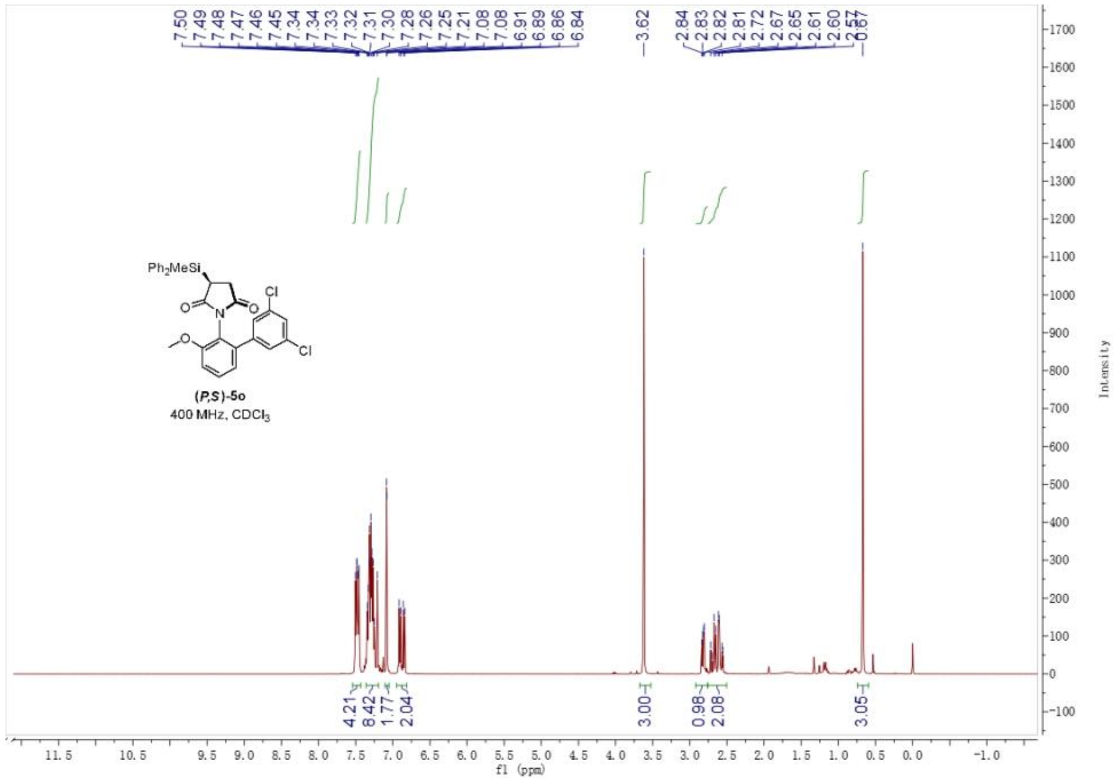

Supplementary Figure 187. ^{1}H NMR spectrum for **5o**

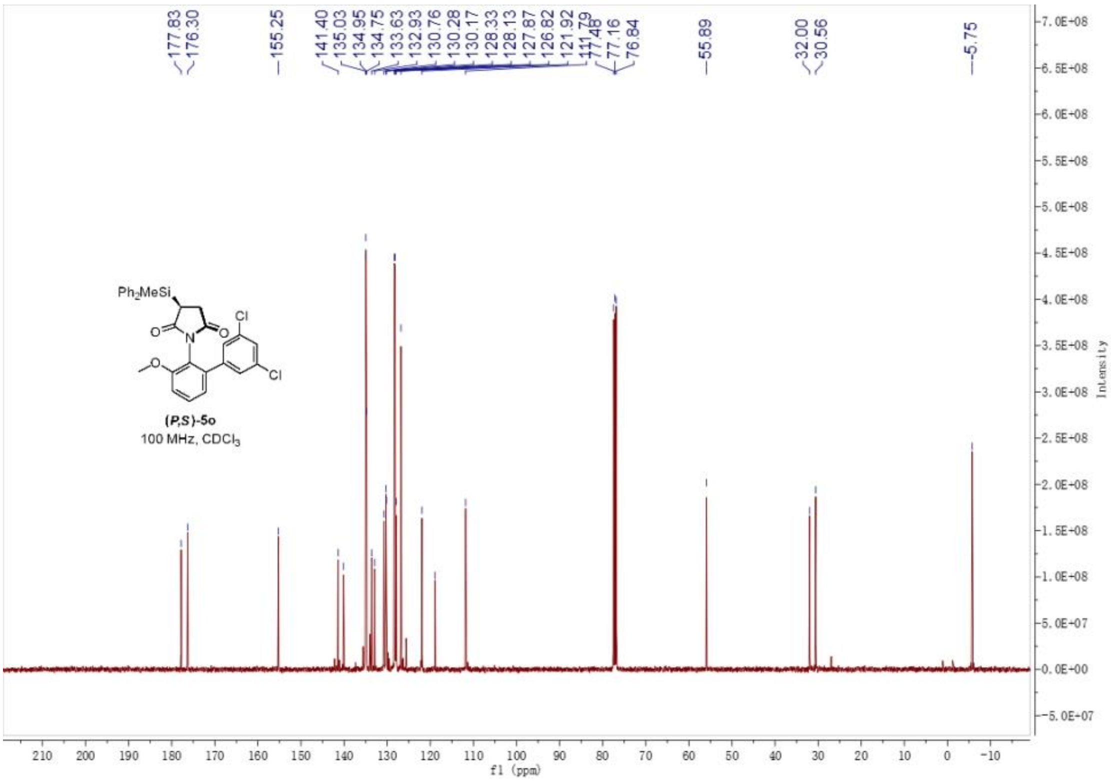

Supplementary Figure 188. ^{13}C NMR spectrum for **5o**

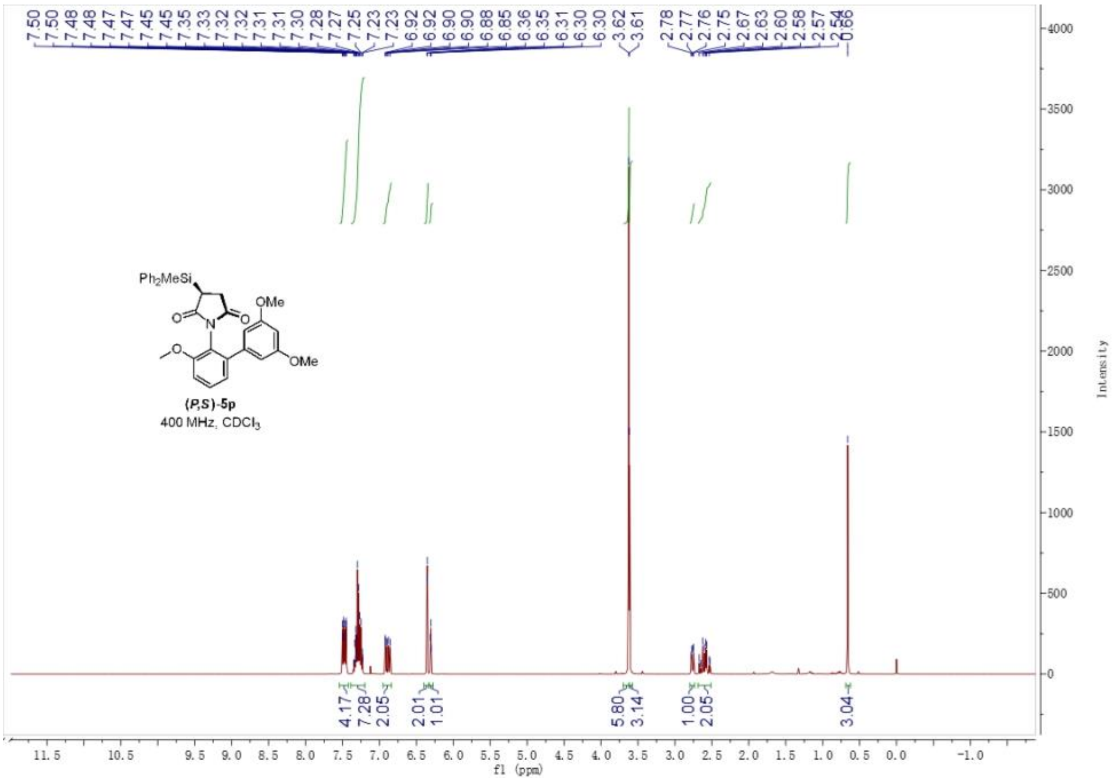

Supplementary Figure 189 ^{1}H NMR spectrum for **5p**

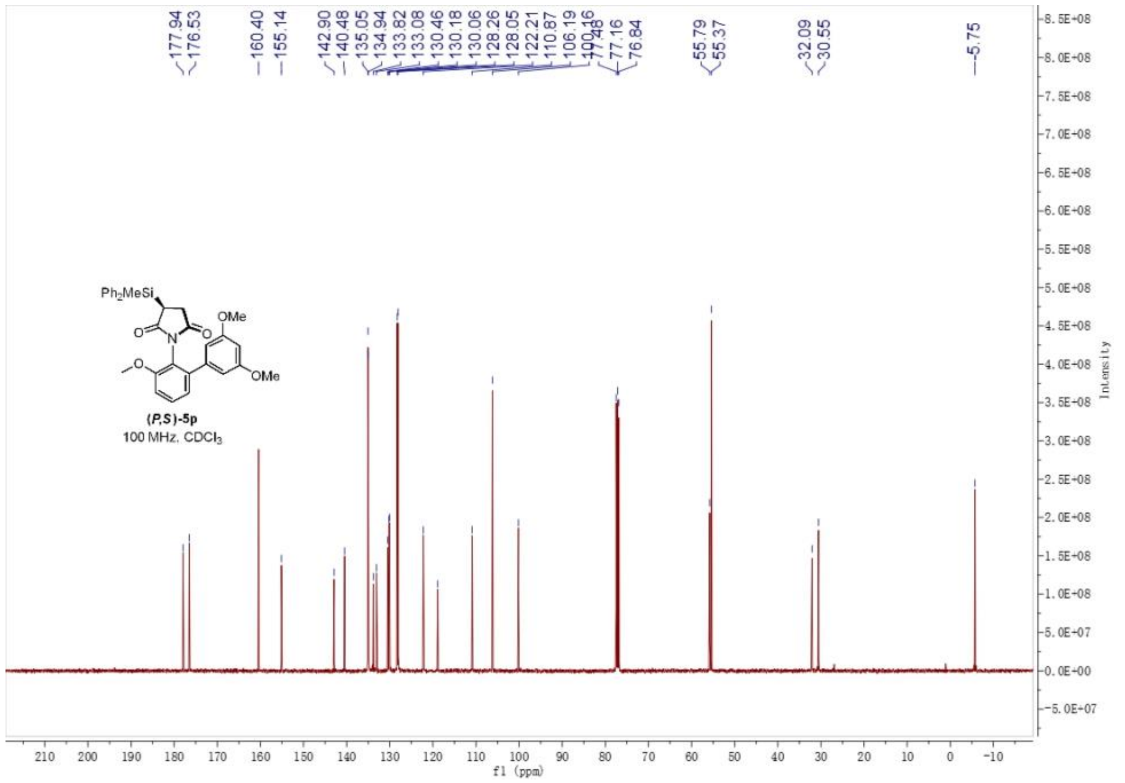

Supplementary Figure 190. ^{13}C NMR spectrum for **5p**

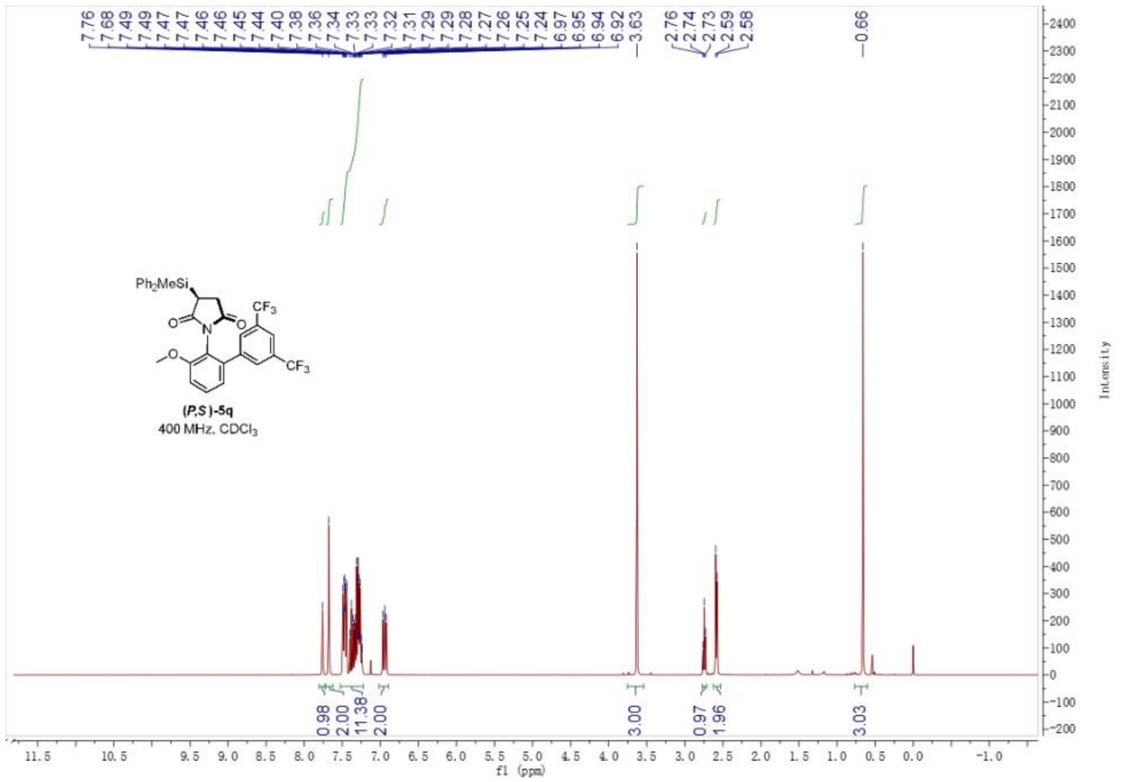

Supplementary Figure 191. ^{1}H NMR spectrum for **5q**

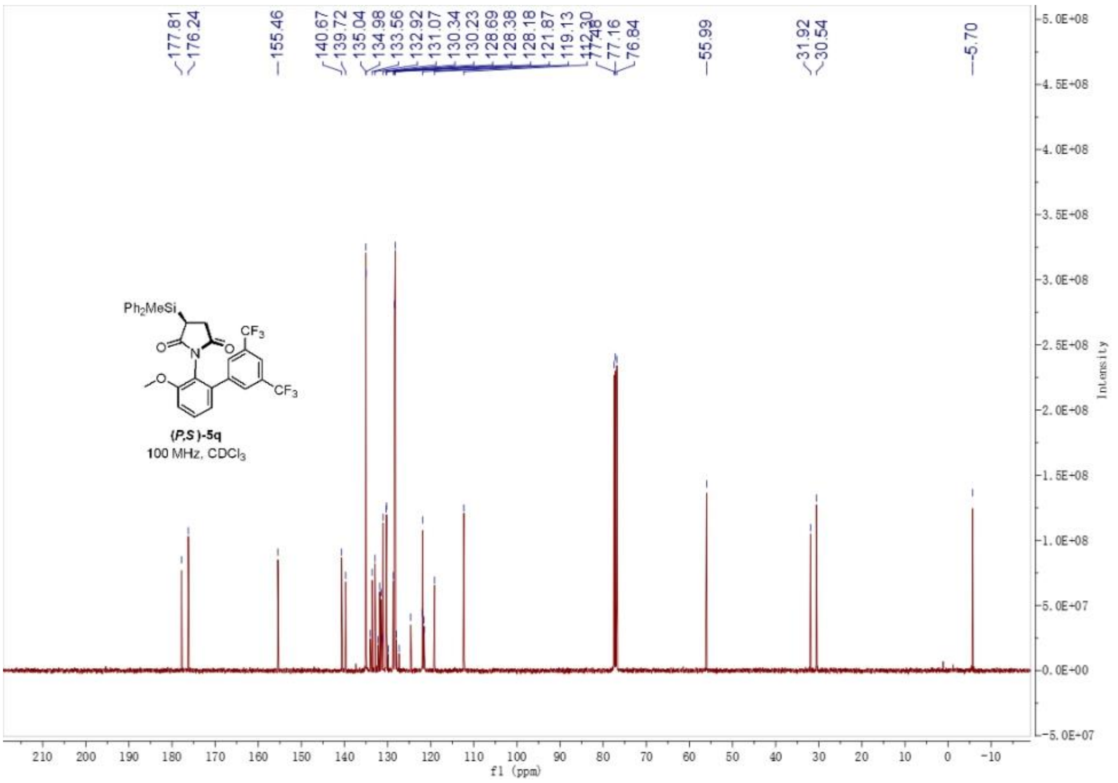

Supplementary Figure 192. ^{13}C NMR spectrum for **5q**

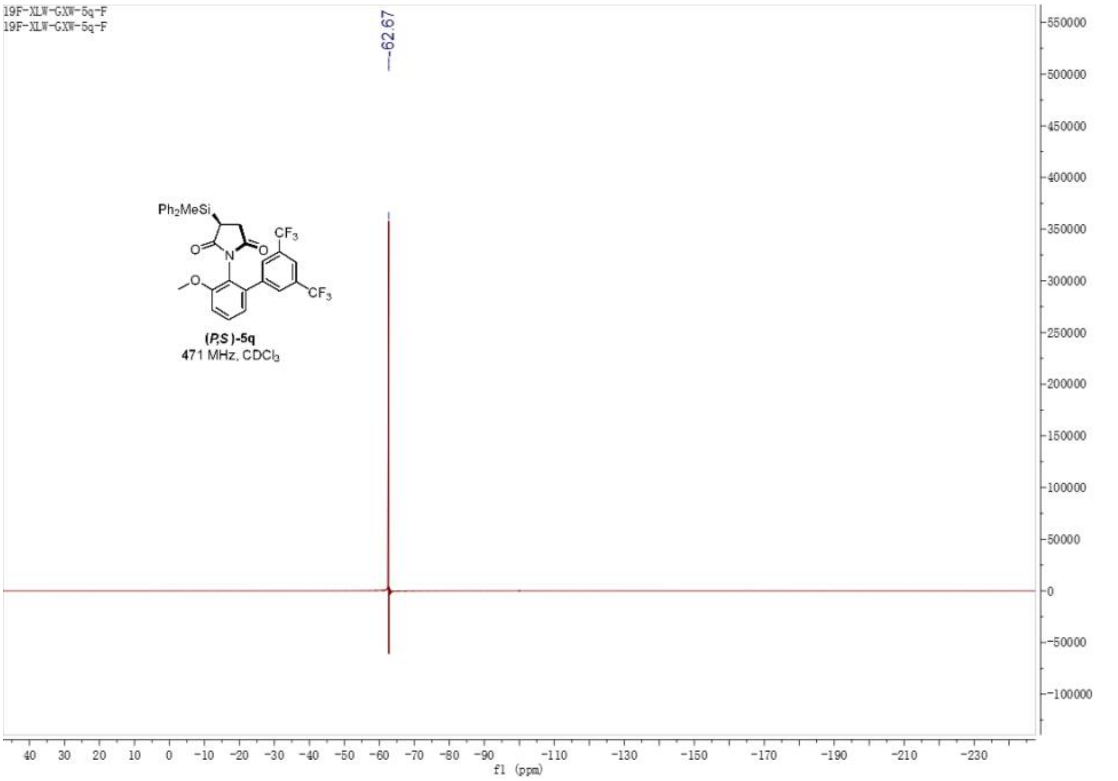

Supplementary Figure 193. ^{19}F NMR spectrum for **5q**

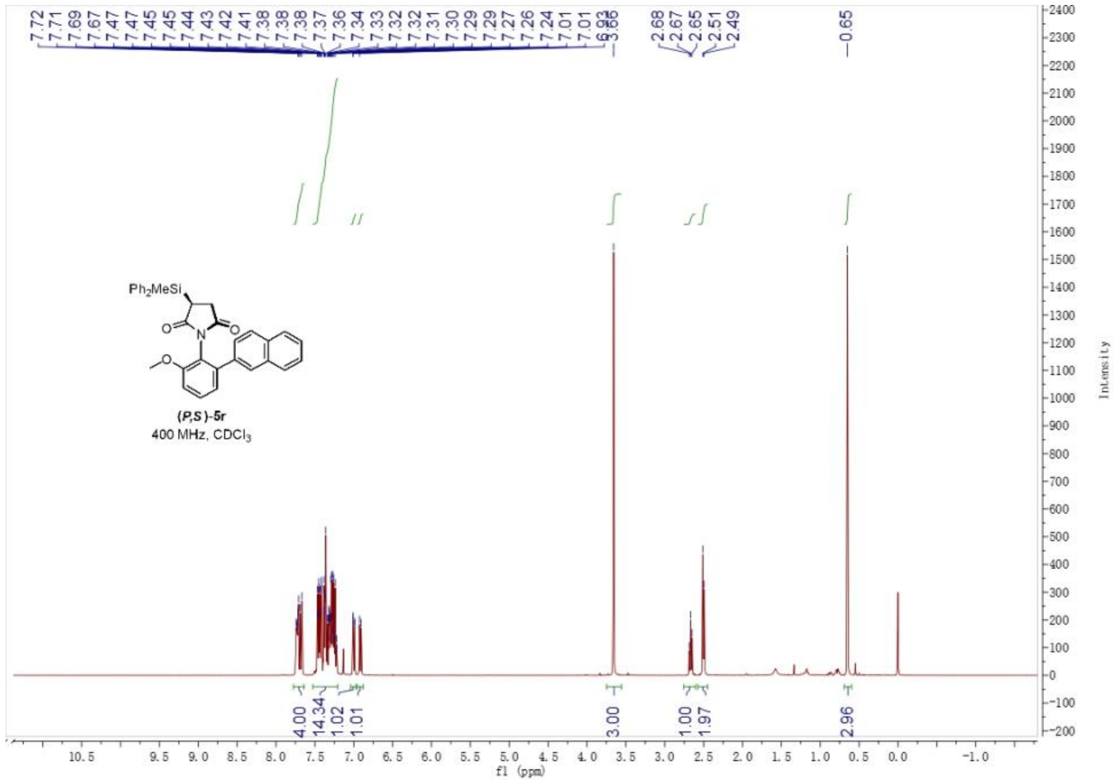

Supplementary Figure 194. ^{1}H NMR spectrum for **5r**

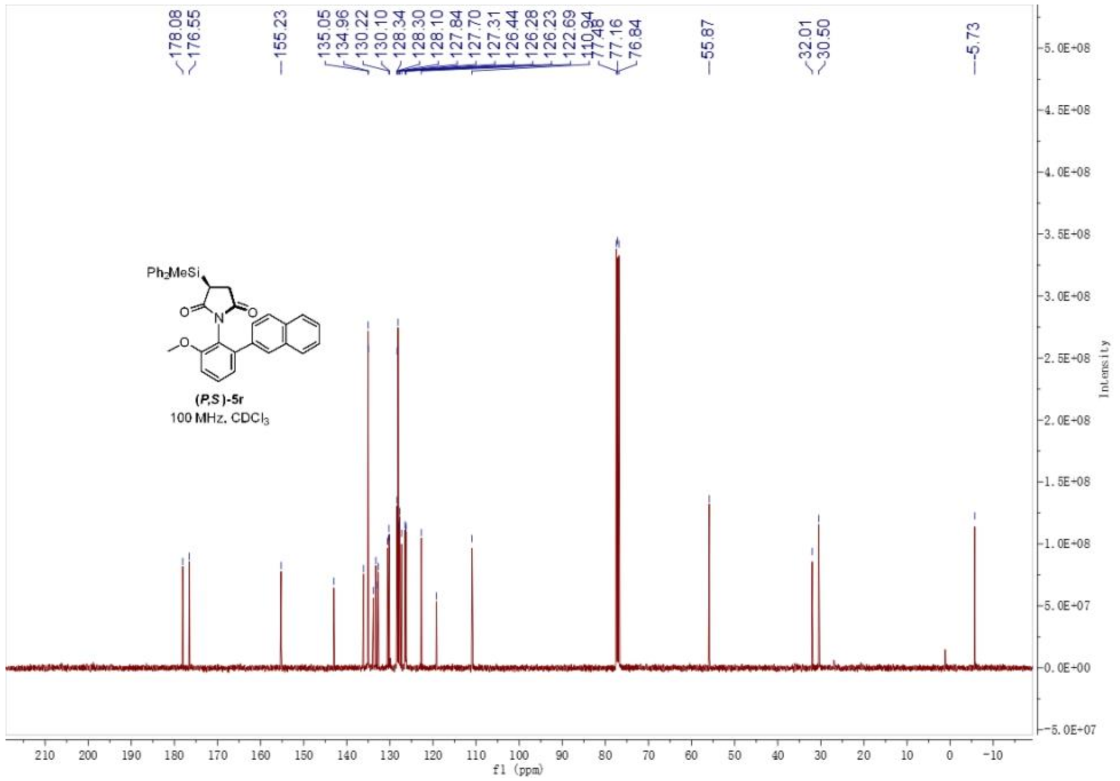

Supplementary Figure 195. ^{13}C NMR spectrum for **5r**

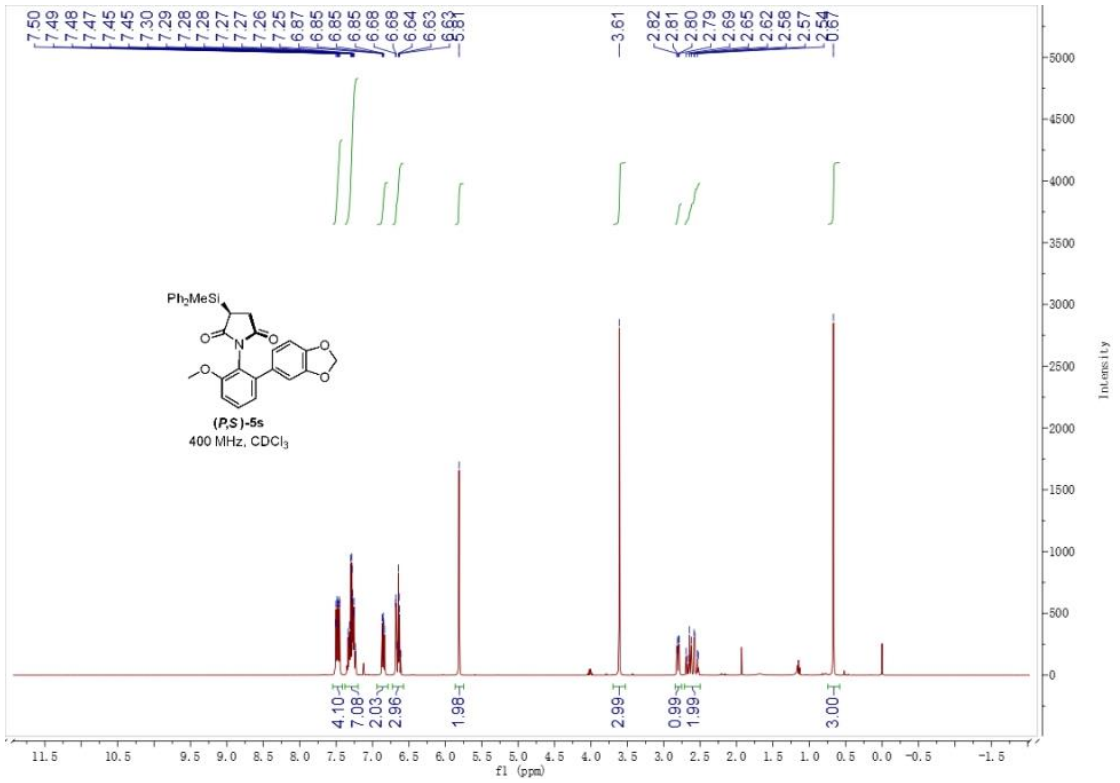

Supplementary Figure 196. ^{1}H NMR spectrum for **5s**

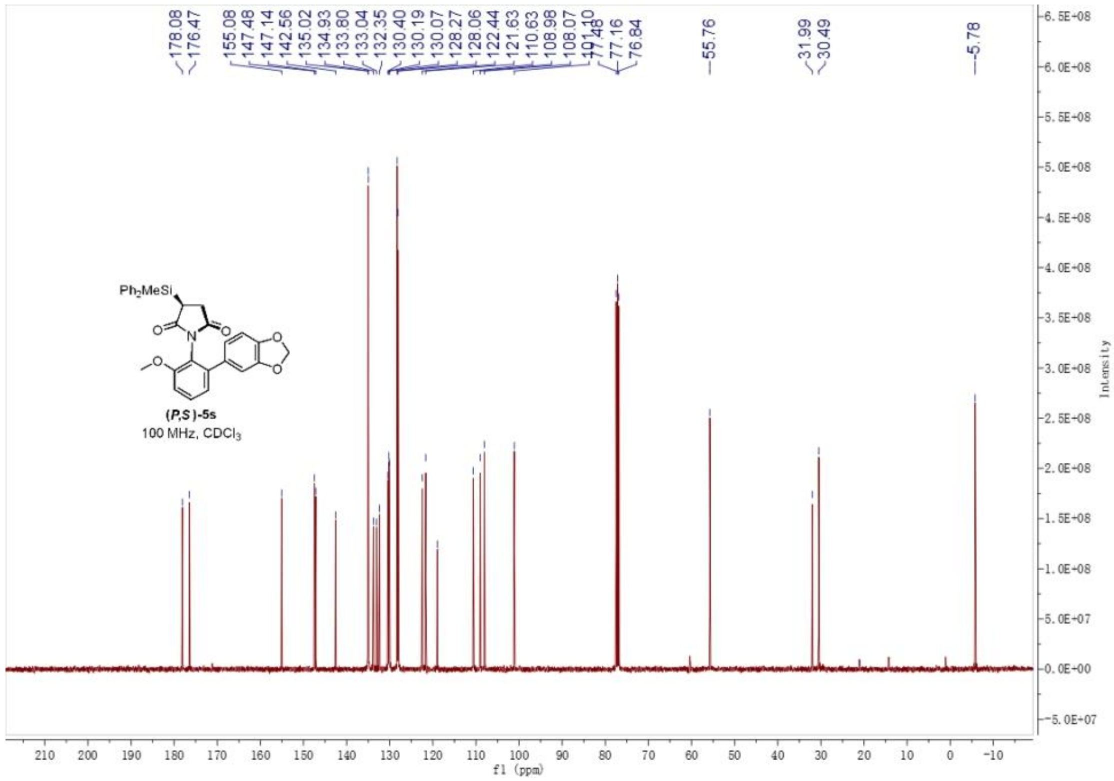

Supplementary Figure 197. ^{13}C NMR spectrum for **5s**

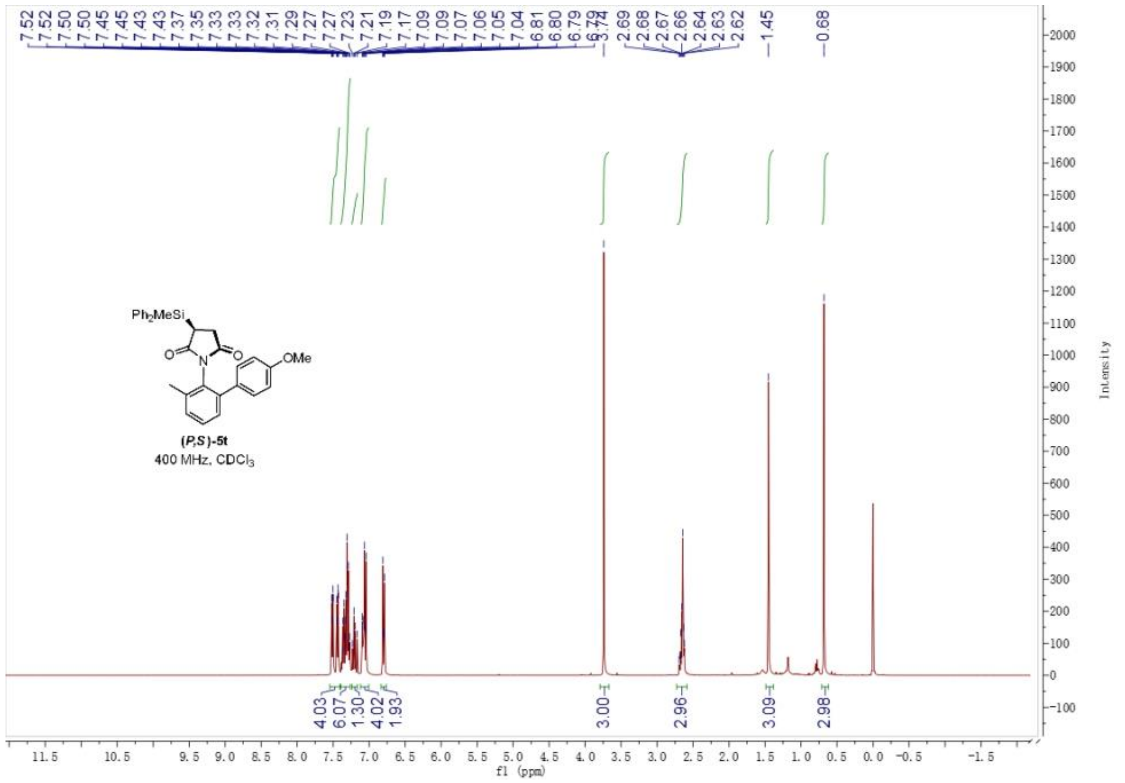

Supplementary Figure 198. ^{1}H NMR spectrum for **5t**

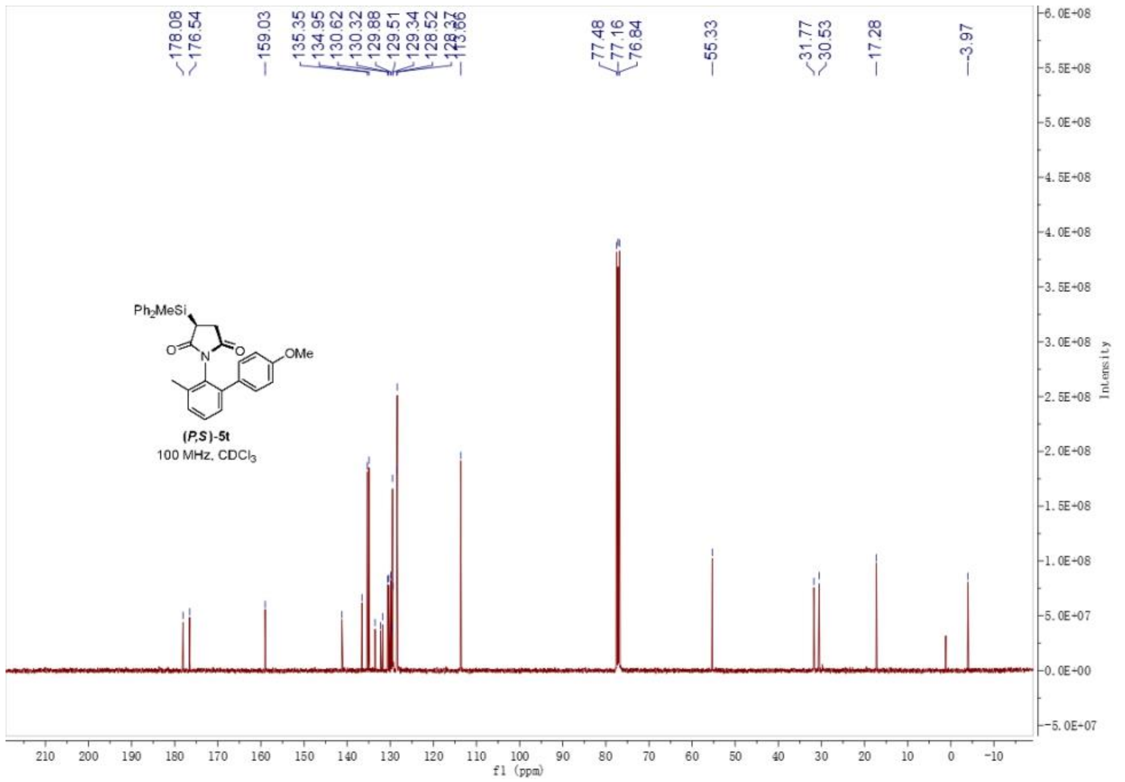

Supplementary Figure 199. ^{13}C NMR spectrum for **5t**

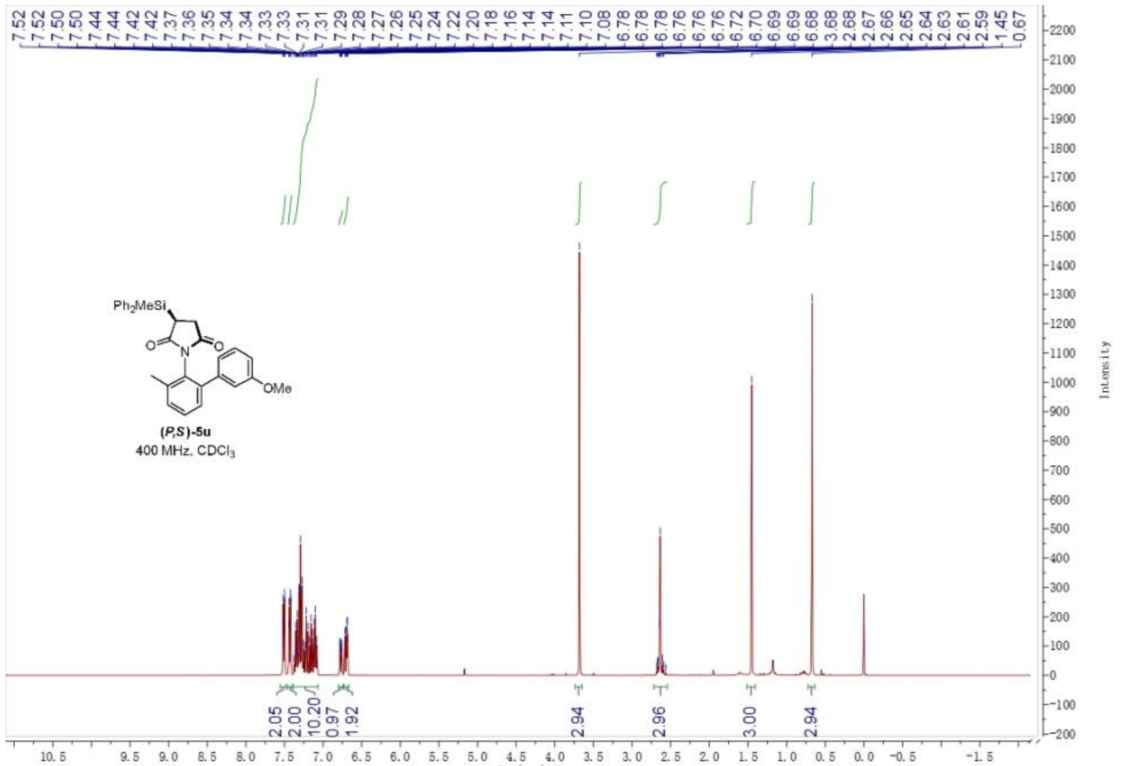

Supplementary Figure 200. ^{1}H NMR spectrum for **5u**

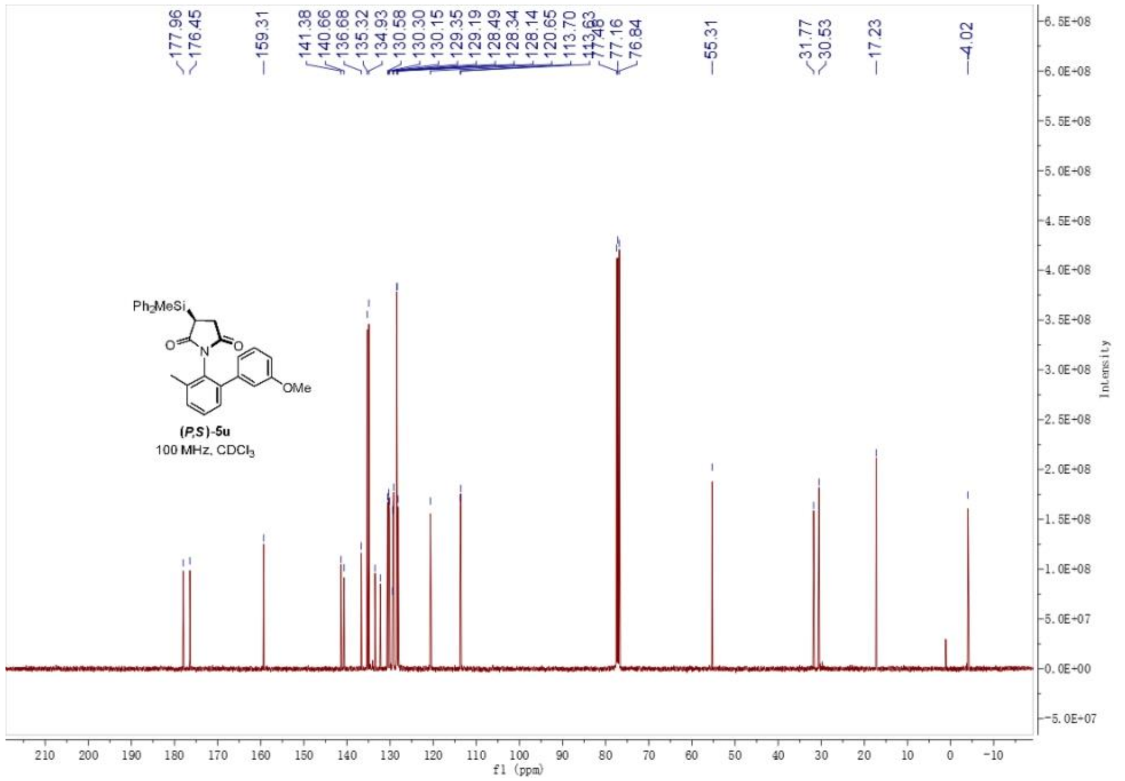

Supplementary Figure 201. ^{13}C NMR spectrum for **5u**

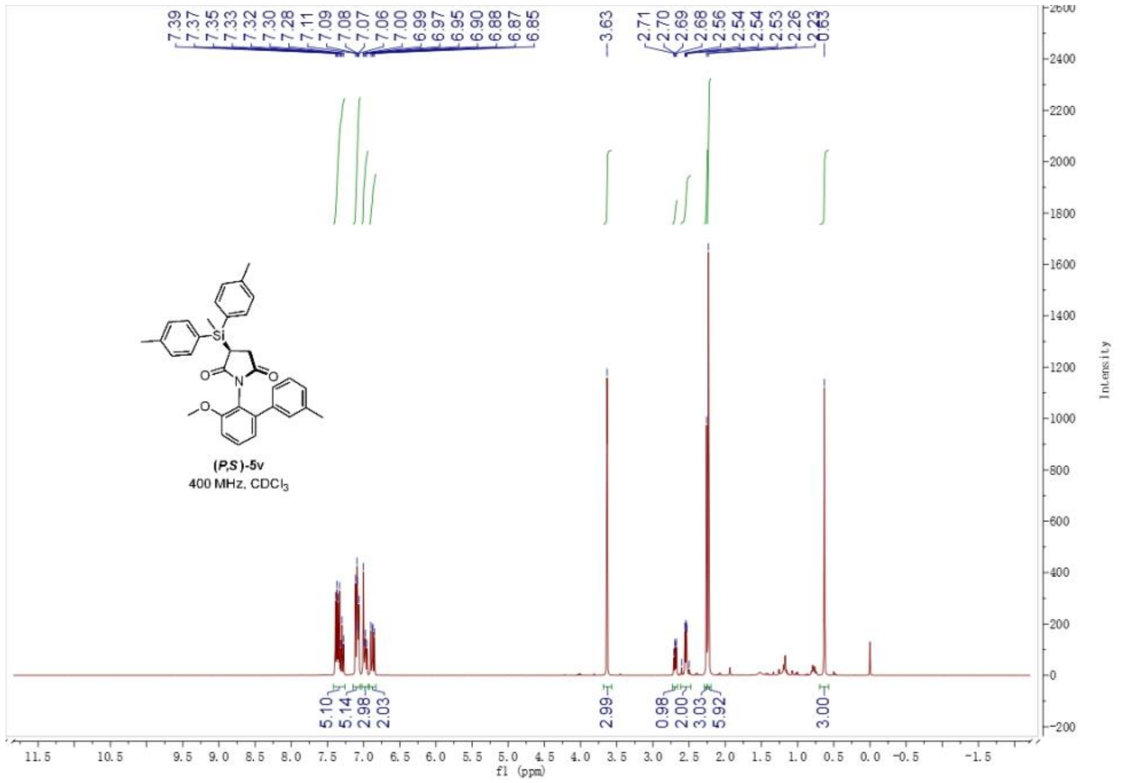

Supplementary Figure 202. ^{1}H NMR spectrum for **5v**

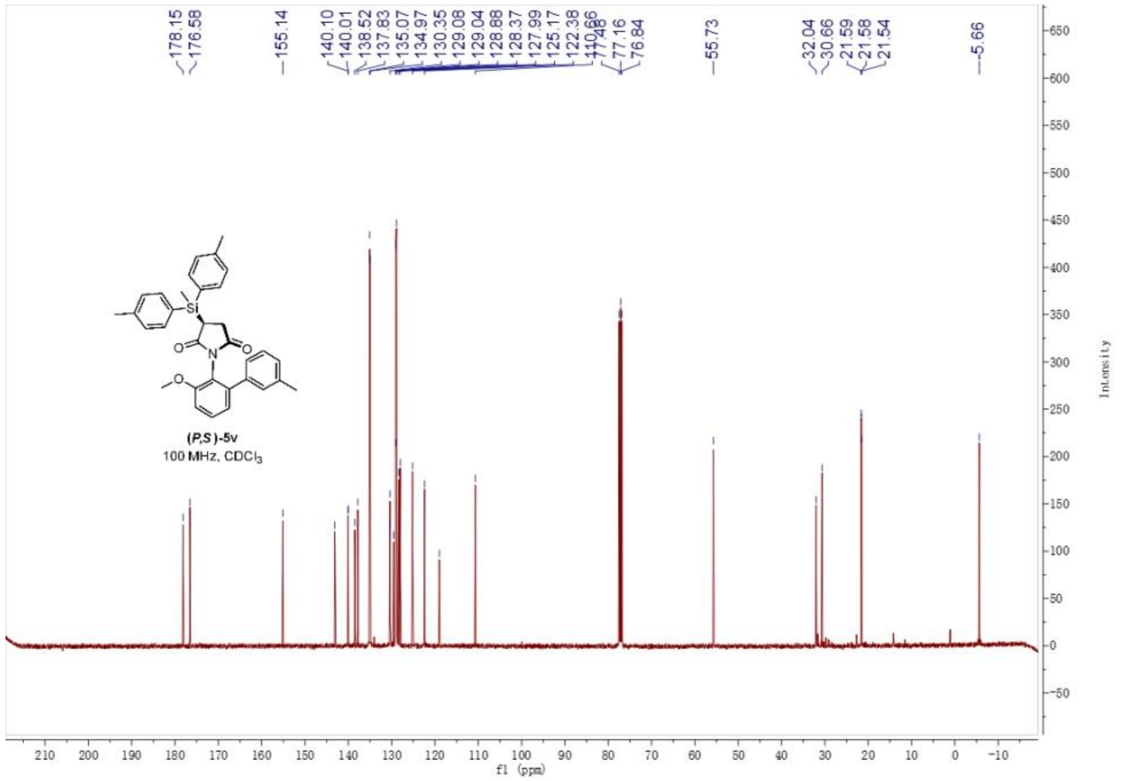

Supplementary Figure 203. ^{13}C NMR spectrum for **5v**

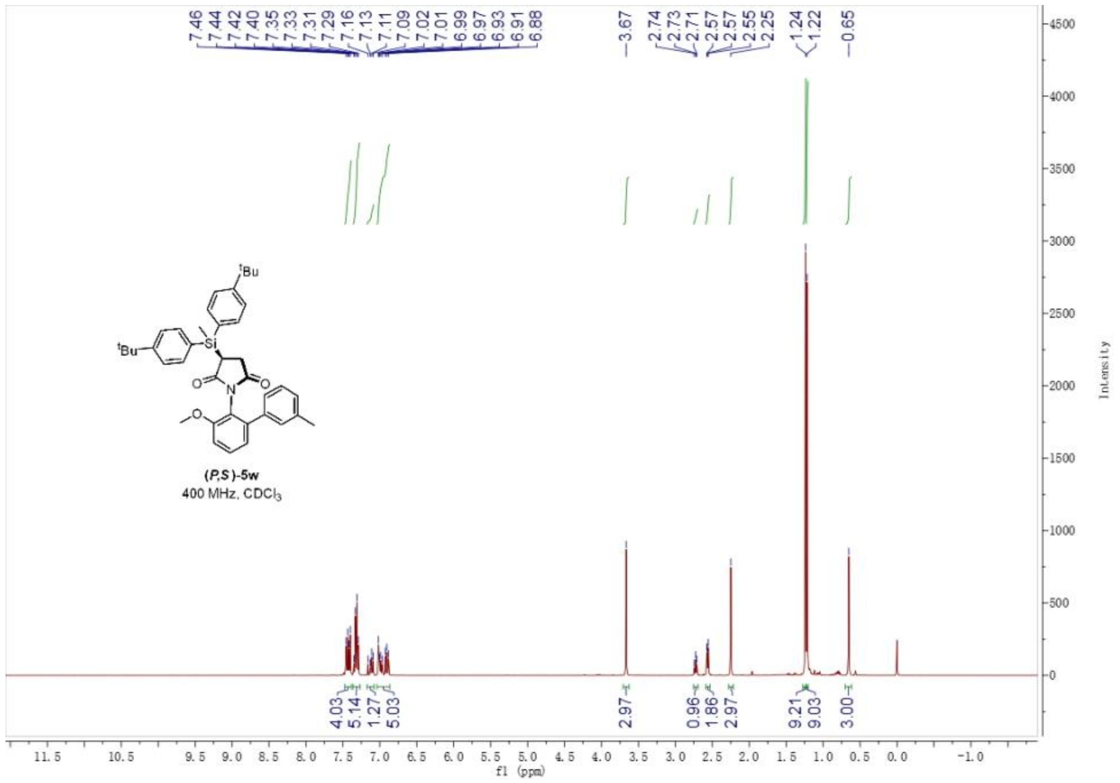

Supplementary Figure 204. ^{1}H NMR spectrum for **5w**

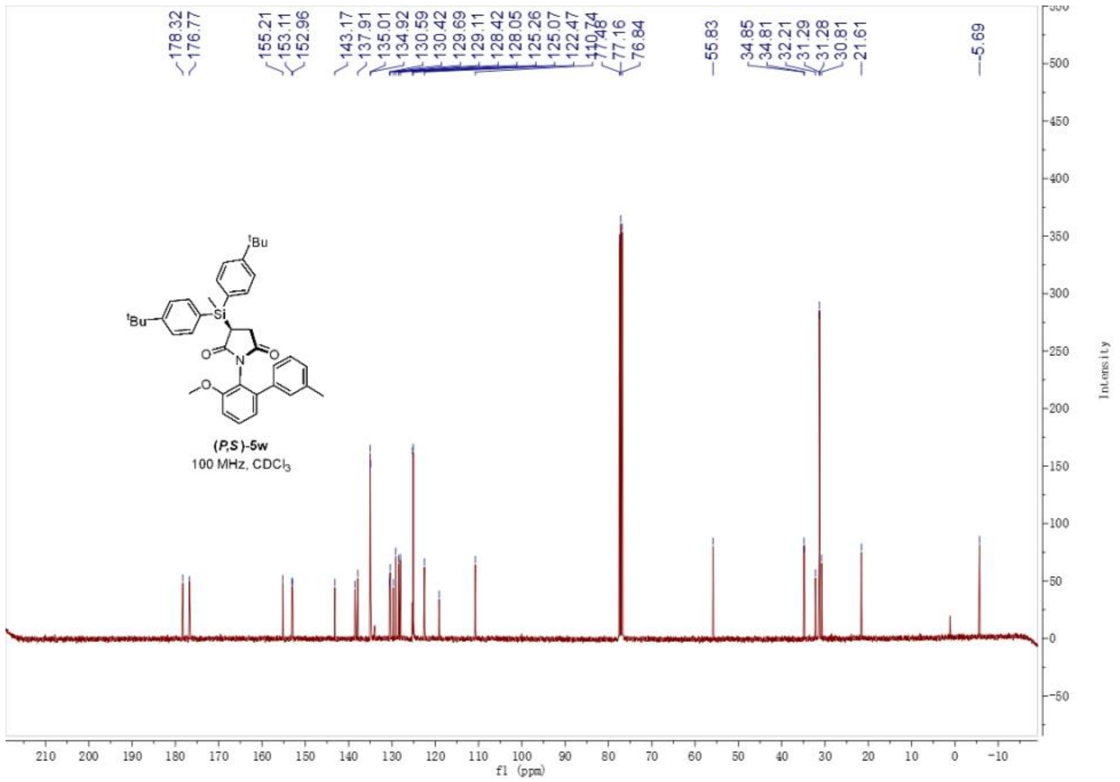

Supplementary Figure 205. ^{13}C NMR spectrum for **5w**

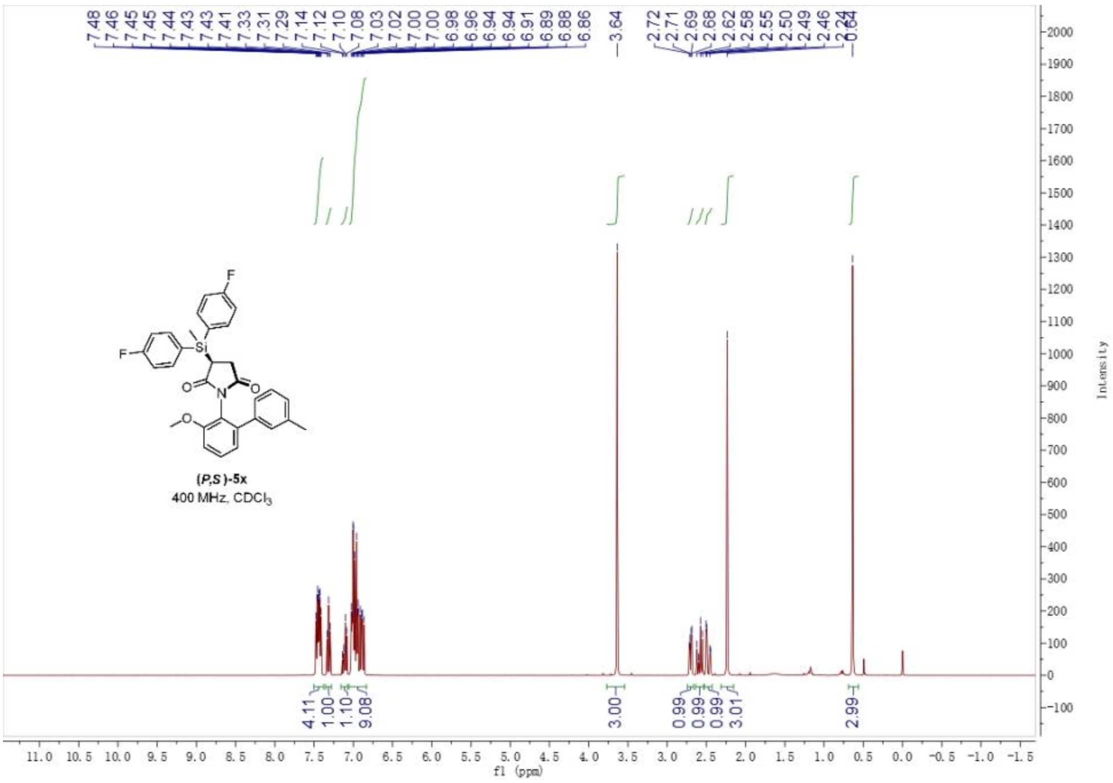

Supplementary Figure 206. ^{1}H NMR spectrum for **5x**

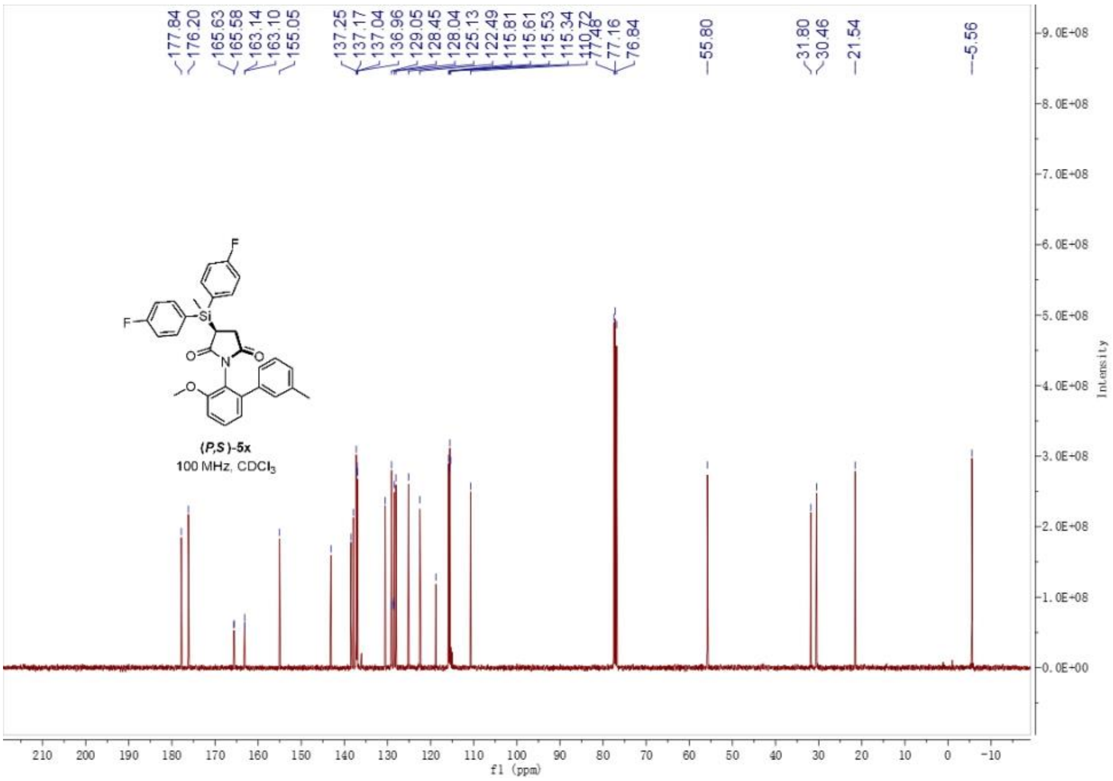

Supplementary Figure 207. ^{13}C NMR spectrum for **5x**

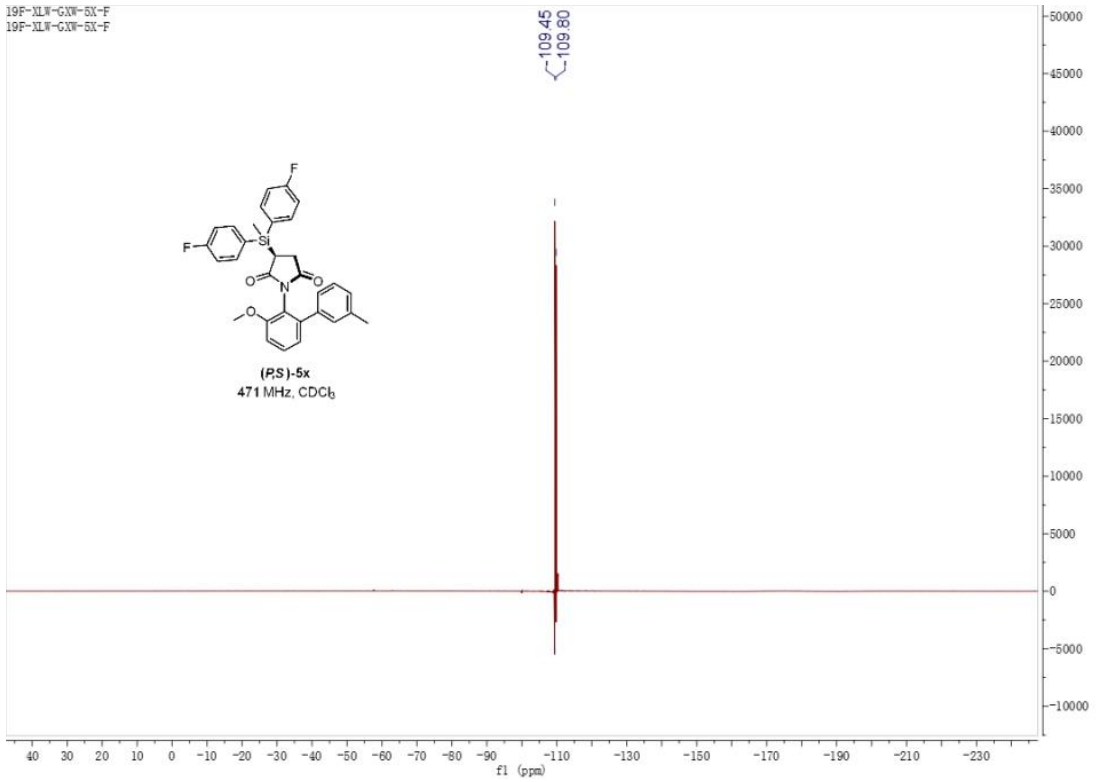

Supplementary Figure 208. ^{19}F NMR spectrum for **5x**

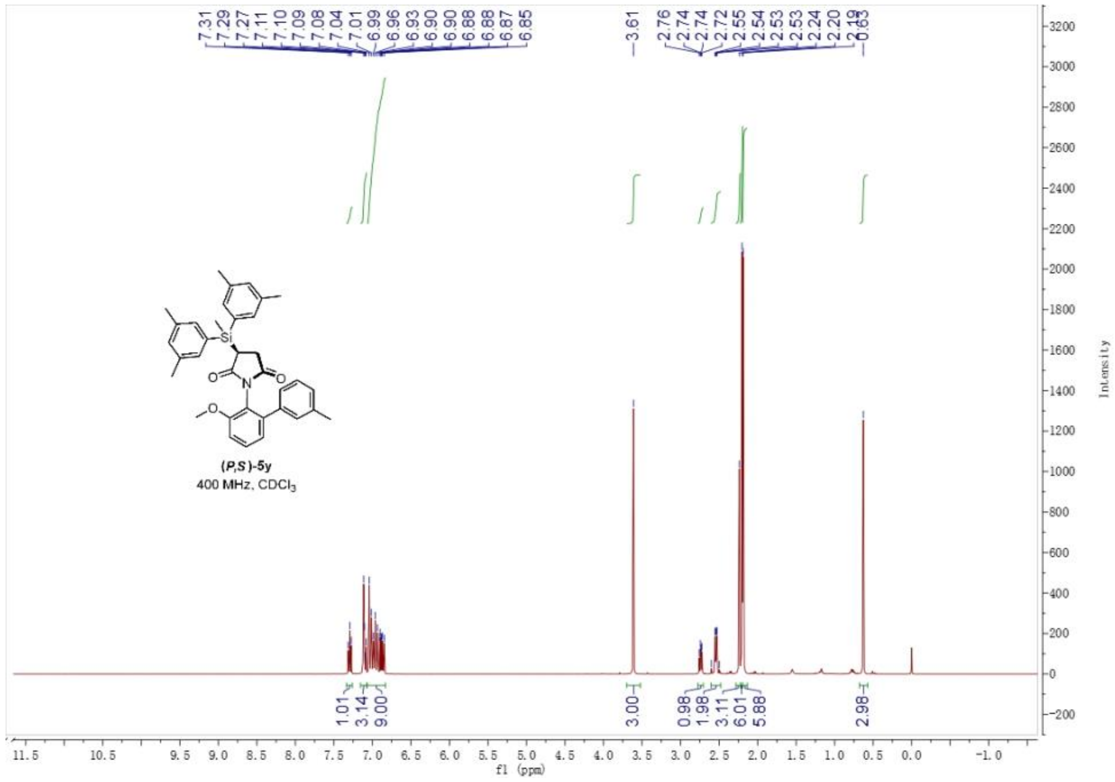

Supplementary Figure 209. ^{1}H NMR spectrum for **5y**

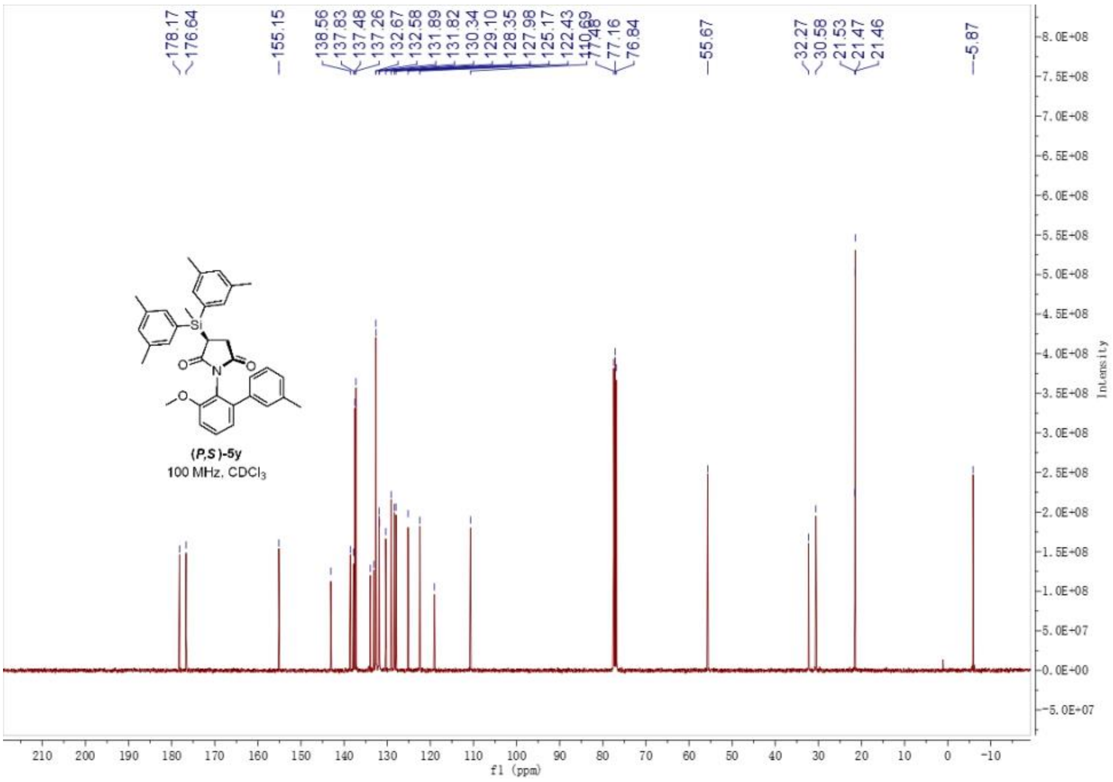

Supplementary Figure 210. ^{13}C NMR spectrum for **5y**

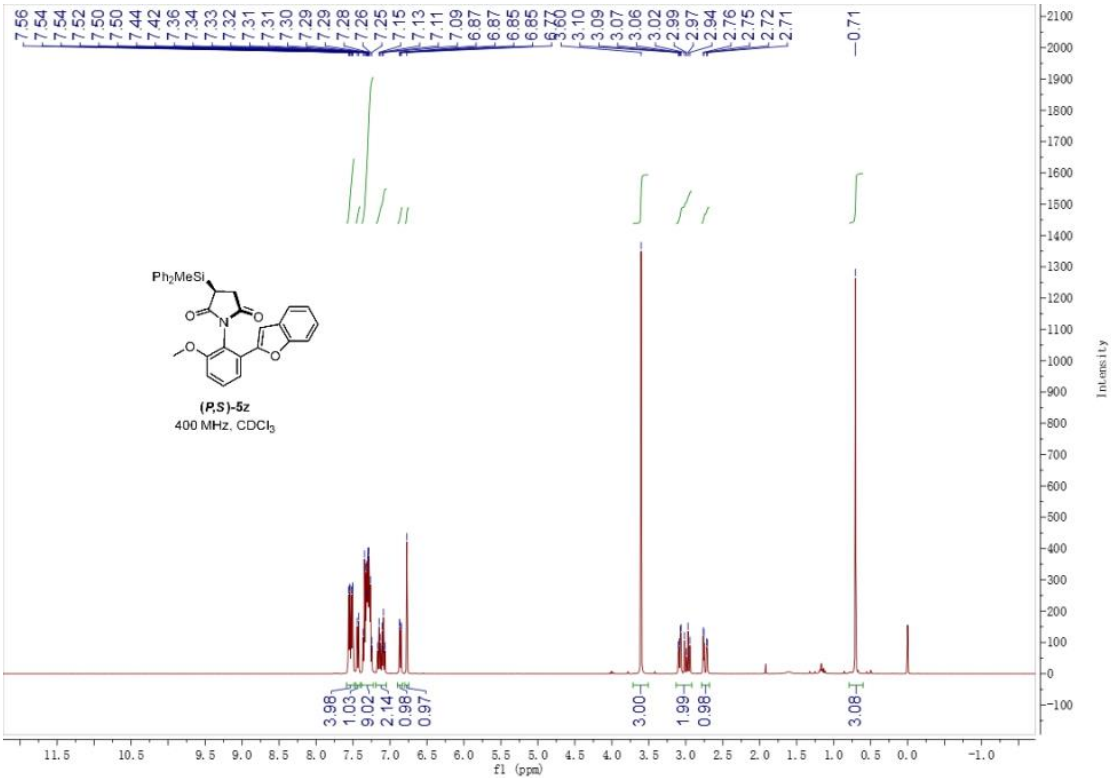

Supplementary Figure 211. ^{1}H NMR spectrum for **5z**

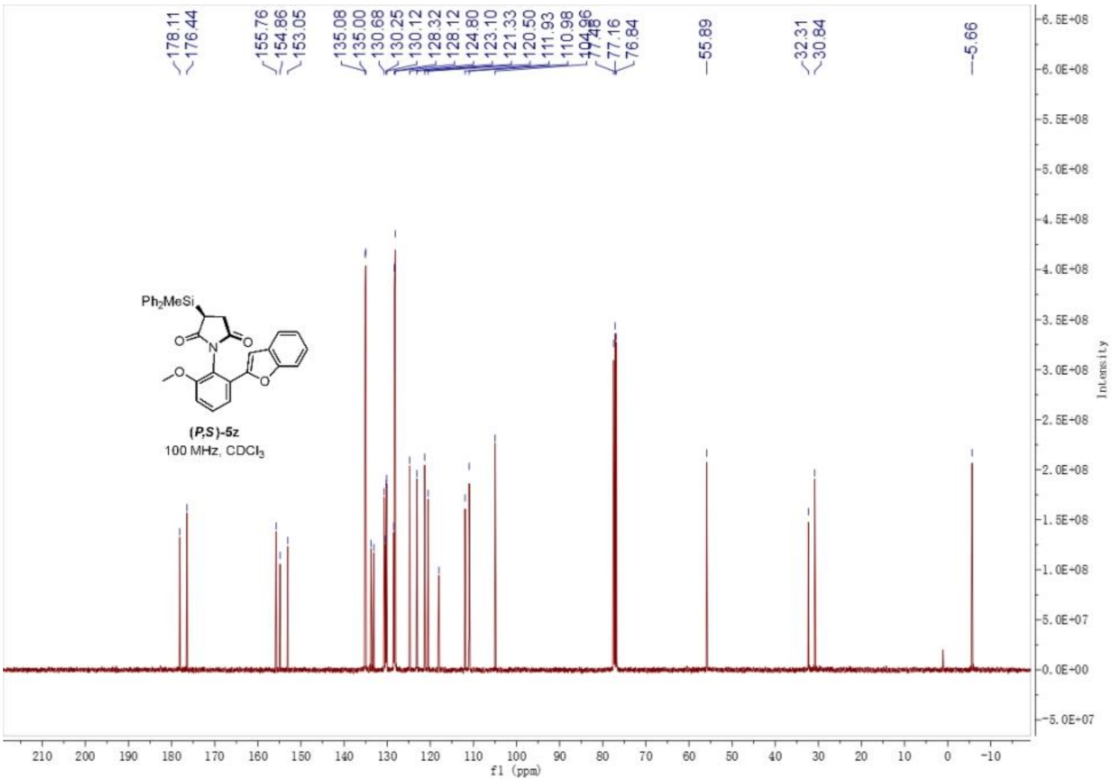

Supplementary Figure 212. ^{13}C NMR spectrum for **5z**

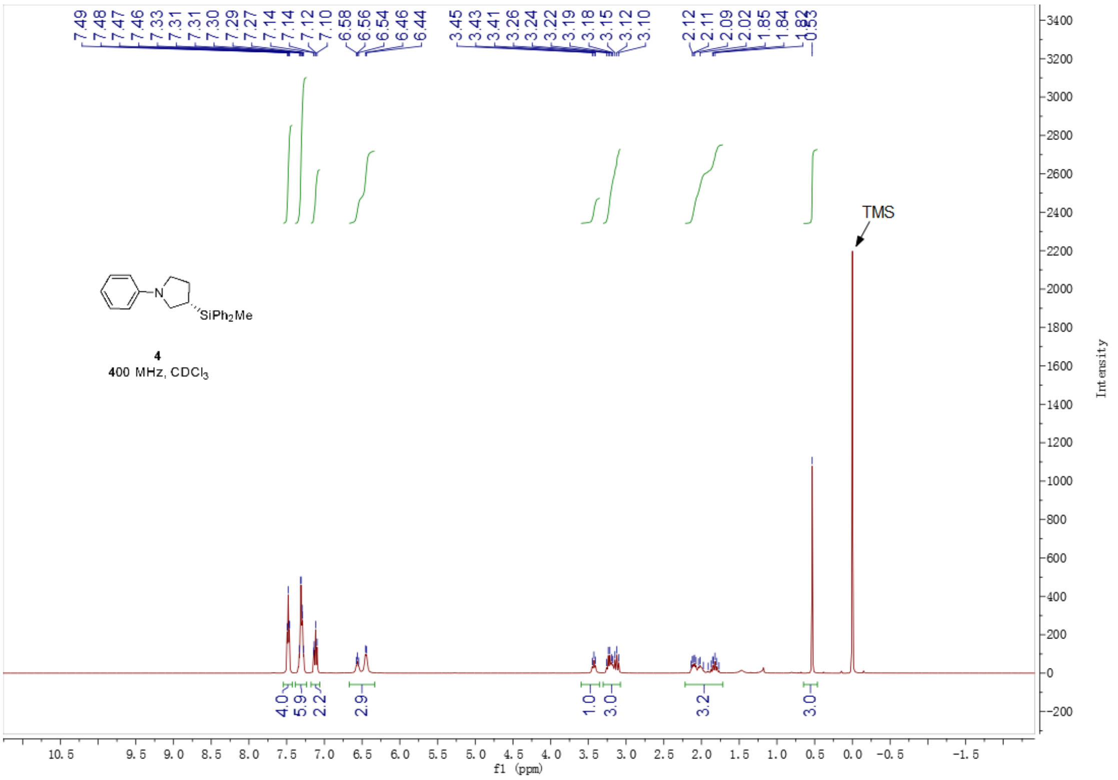

Supplementary Figure 213. ^{1}H NMR spectrum for **6**

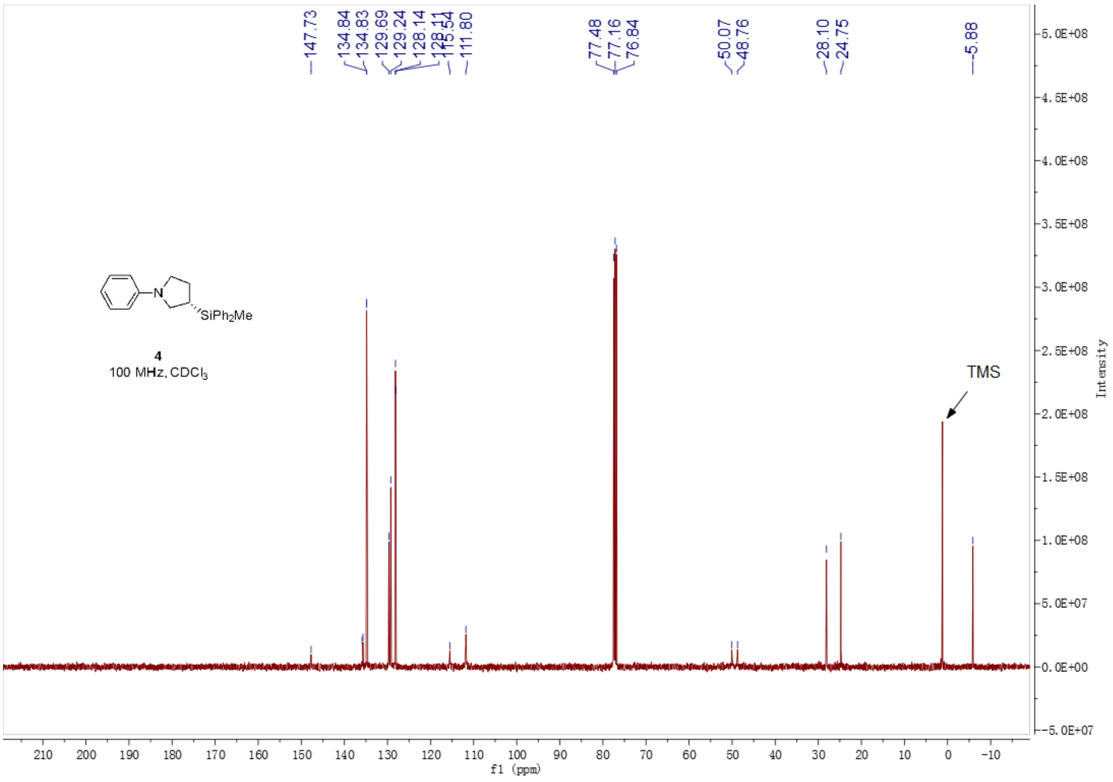

Supplementary Figure 214. ^{13}C NMR spectrum for **6**

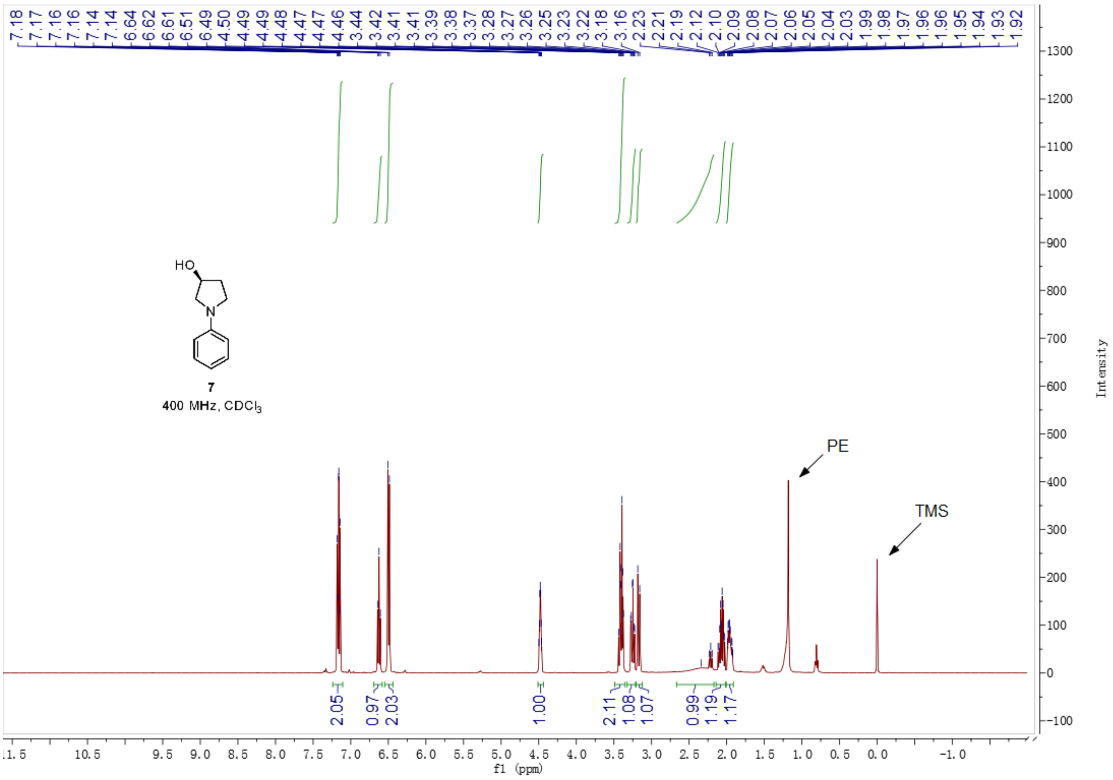

Supplementary Figure 215. ^{1}H NMR spectrum for **7**

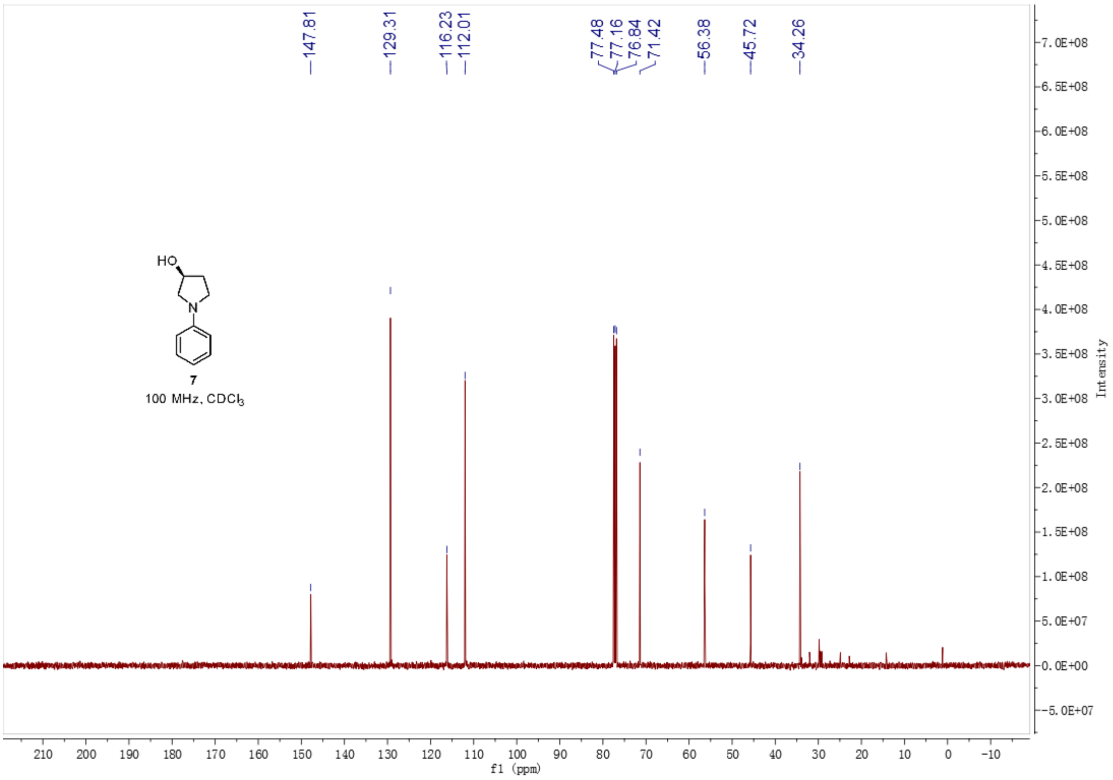

Supplementary Figure 216. ^{13}C NMR spectrum for **7**

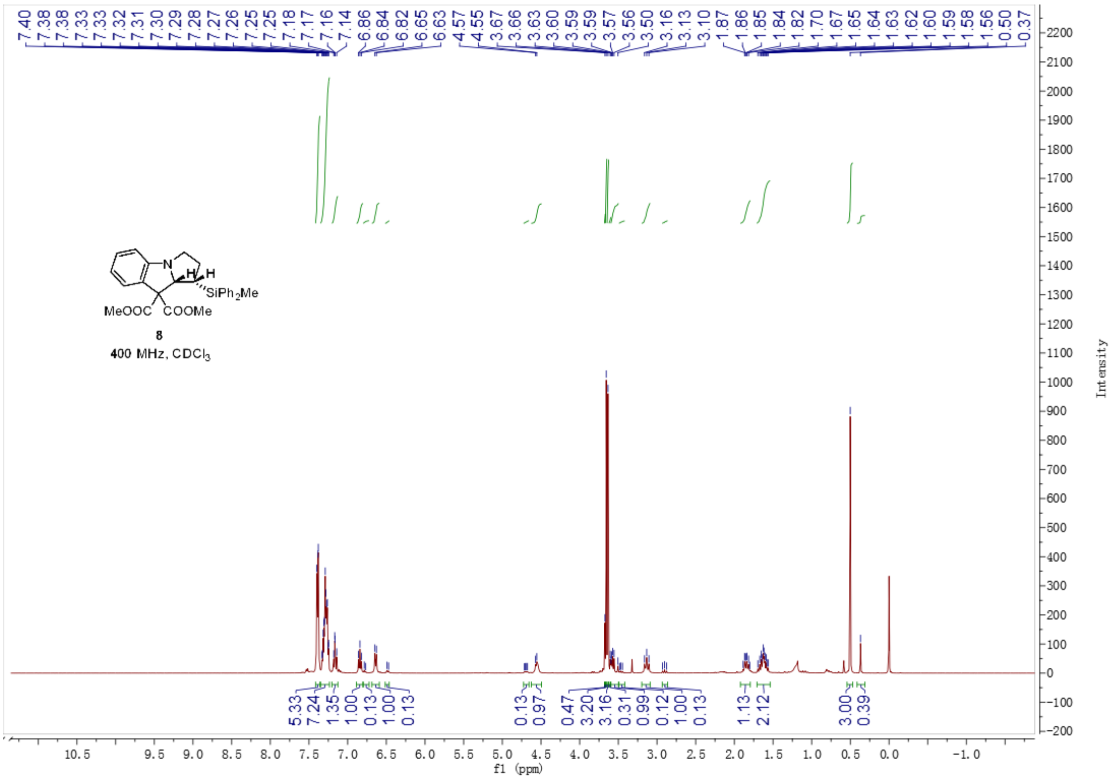

Supplementary Figure 217. ^{1}H NMR spectrum for **8**

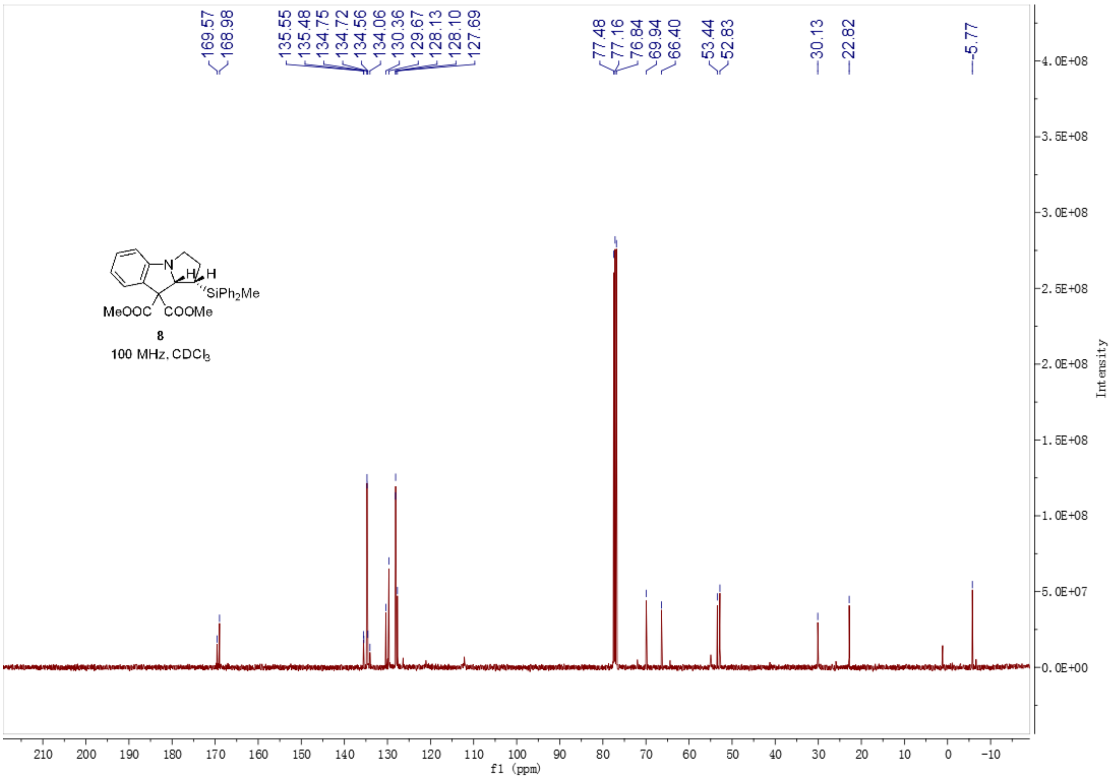

Supplementary Figure 218. ^{13}C NMR spectrum for **8**

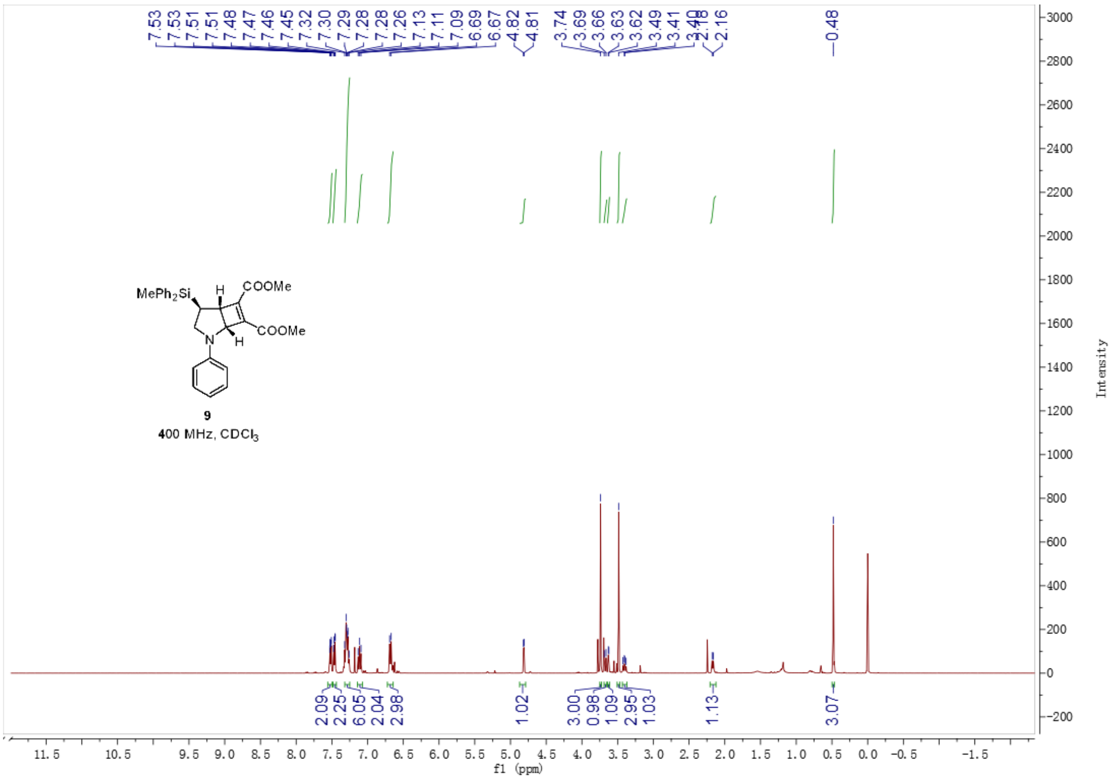

Supplementary Figure 219. ^{1}H NMR spectrum for **9**

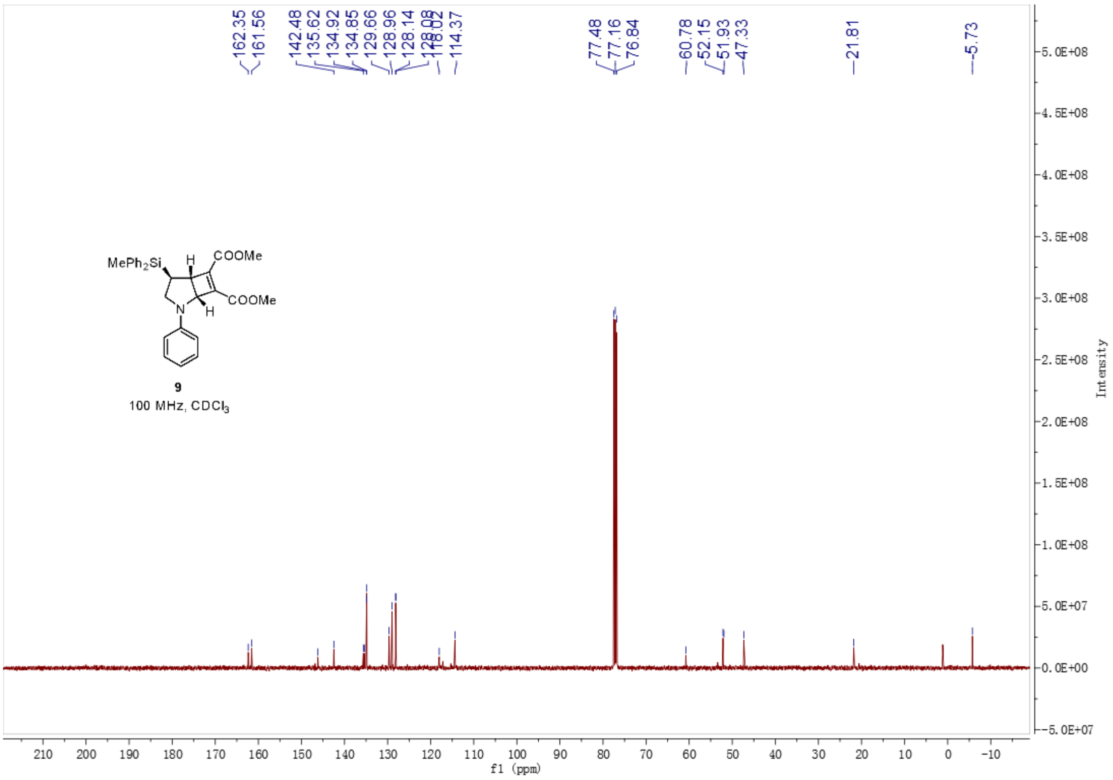

Supplementary Figure 220. ^{13}C NMR spectrum for **9**

HPLC Spectra

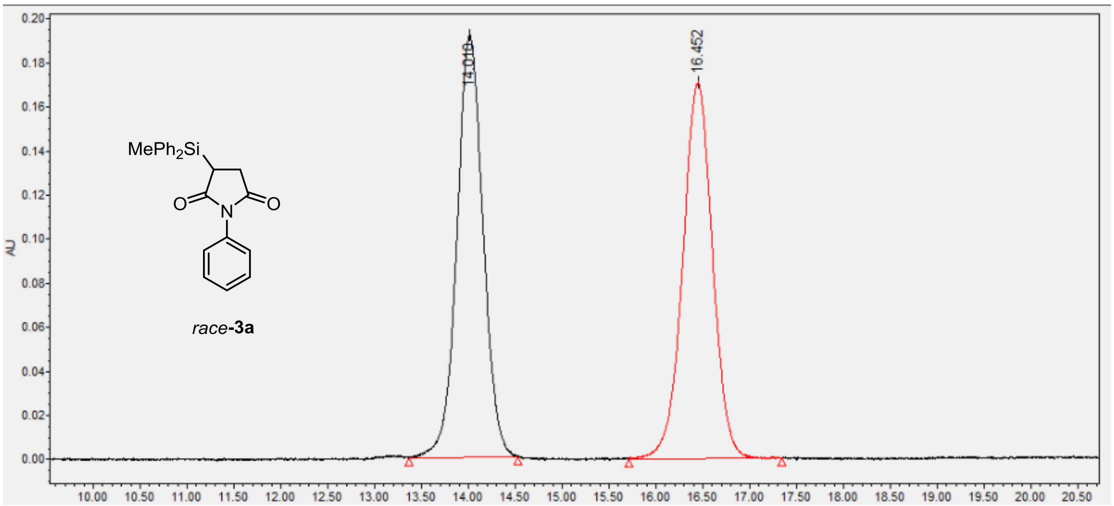

	Time/min	Area	Height	Area%
1	14.010	3713844	191353	49.74
2	16.452	3752755	170952	50.26

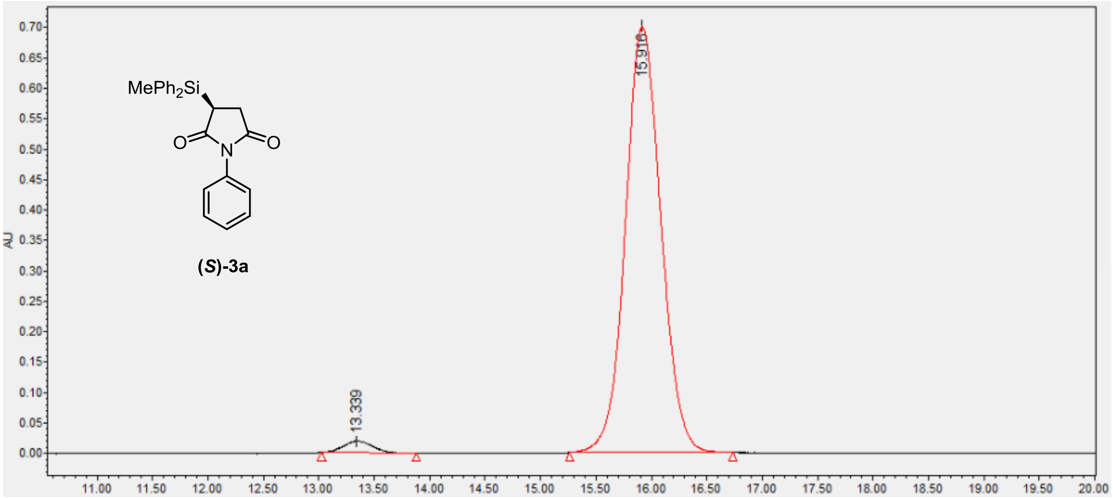

	Time/min	Area	Height	Area%
1	13.339	351266	19352	2.20
2	15.916	15603246	700885	97.80

Supplementary Figure 221. HPLC spectra for **3a**

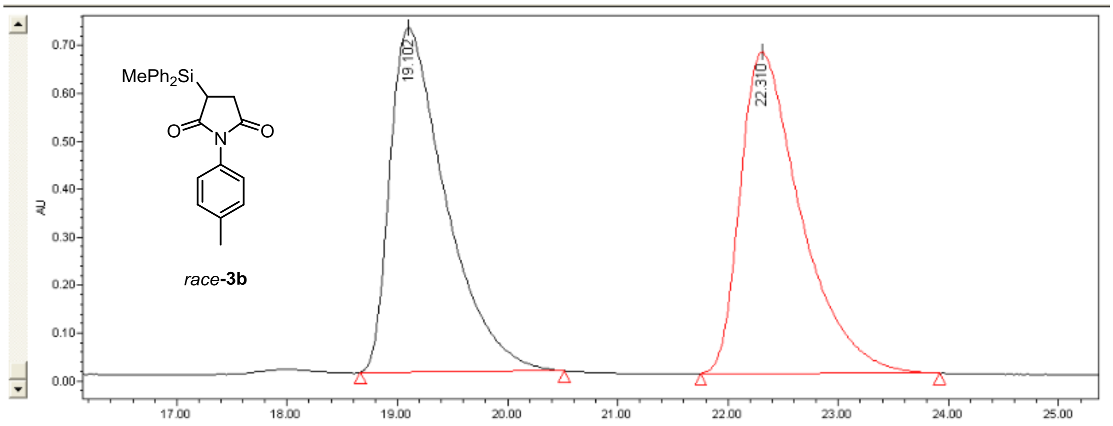

	Time/min	Area	Height	Area%
1	19.102	25374614	715010	49.76
2	22.310	25615042	669386	50.24

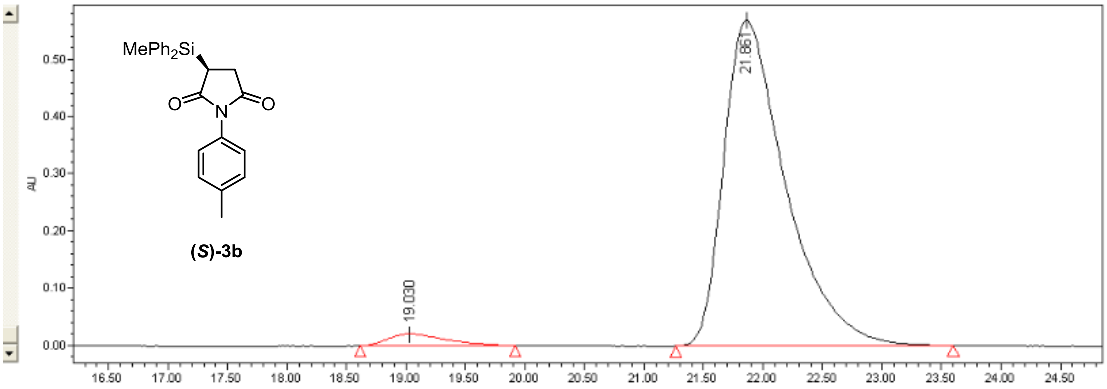

	Time/min	Area	Height	Area%
1	19.030	693205	20970	3.25
2	21.861	20648216	569346	96.75

Supplementary Figure 222. HPLC spectra for **3b**

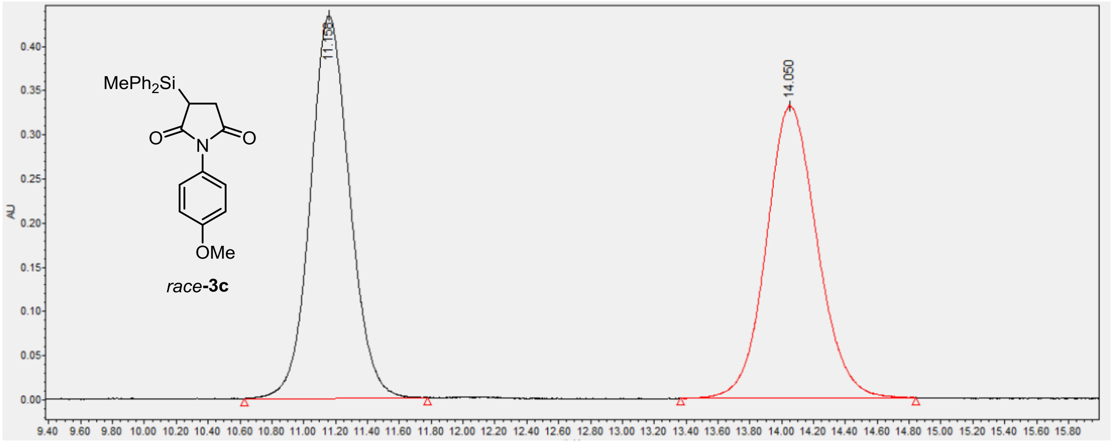

	Time/min	Area	Height	Area%
1	11.158	7428488	432817	50.39
2	14.050	7313758	331196	49.61

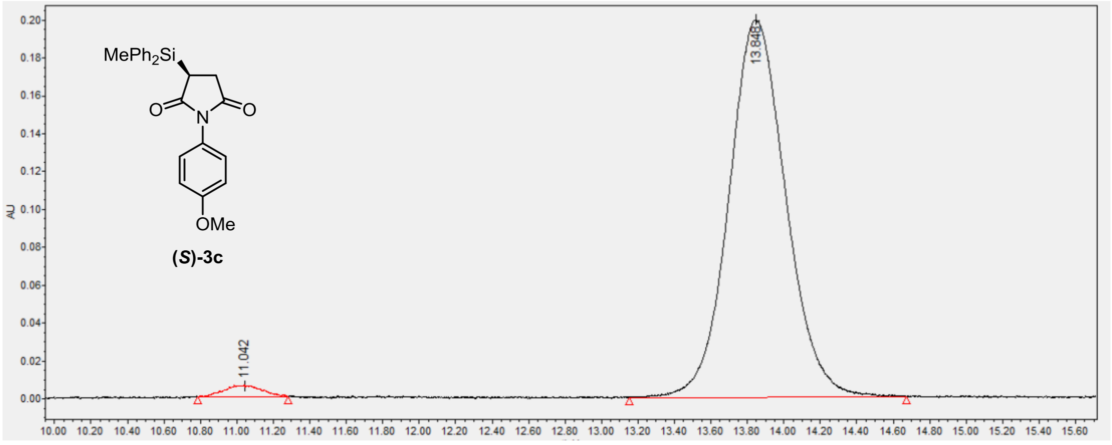

	Time/min	Area	Height	Area%
1	11.042	90055	6143	2.04
2	13.848	4323874	199291	97.96

Supplementary Figure 223. HPLC spectra for **3c**

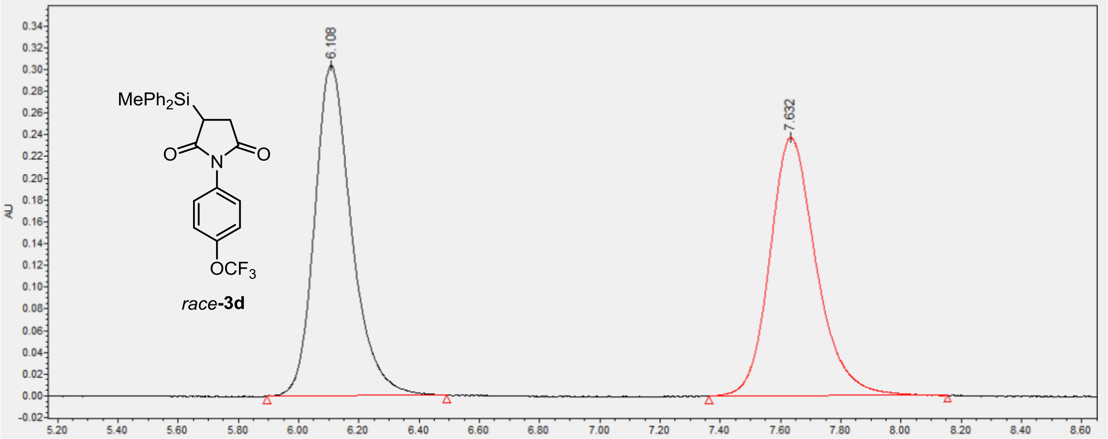

	Time/min	Area	Height	Area%
1	6.108	2595240	303867	50.46
2	7.632	2547992	237610	49.54

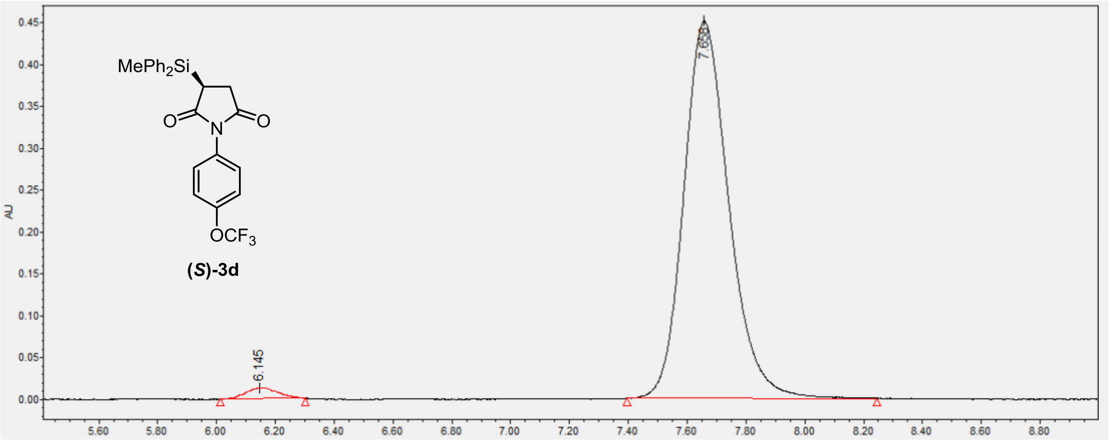

	Time/min	Area	Height	Area%
1	6.145	99452	13110	2.04
2	7.658	4785595	451114	97.96

Supplementary Figure 224. HPLC spectra for **3d**

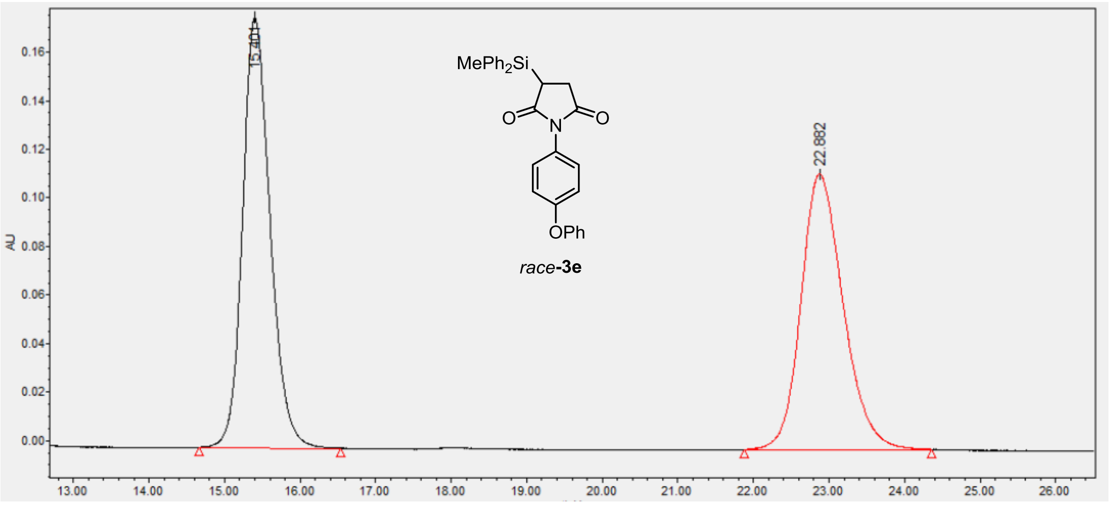

	Time/min	Area	Height	Area%
1	15.401	4483531	177091	50.44
2	22.882	4404835	113582	49.56

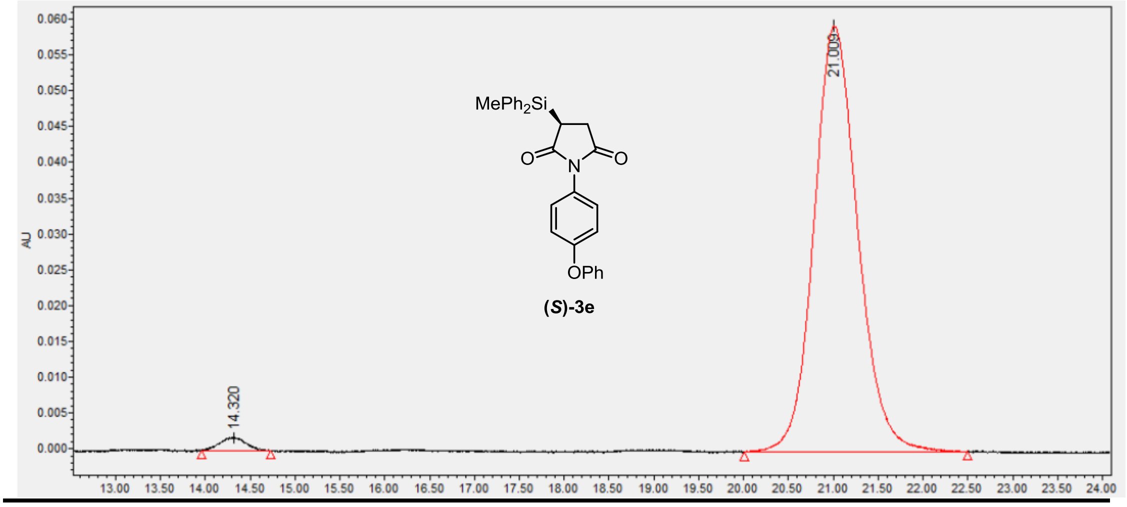

	Time/min	Area	Height	Area%
1	14.320	36453	1822	1.77
2	21.009	2024547	59535	98.23

Supplementary Figure 225. HPLC spectra for **3e**

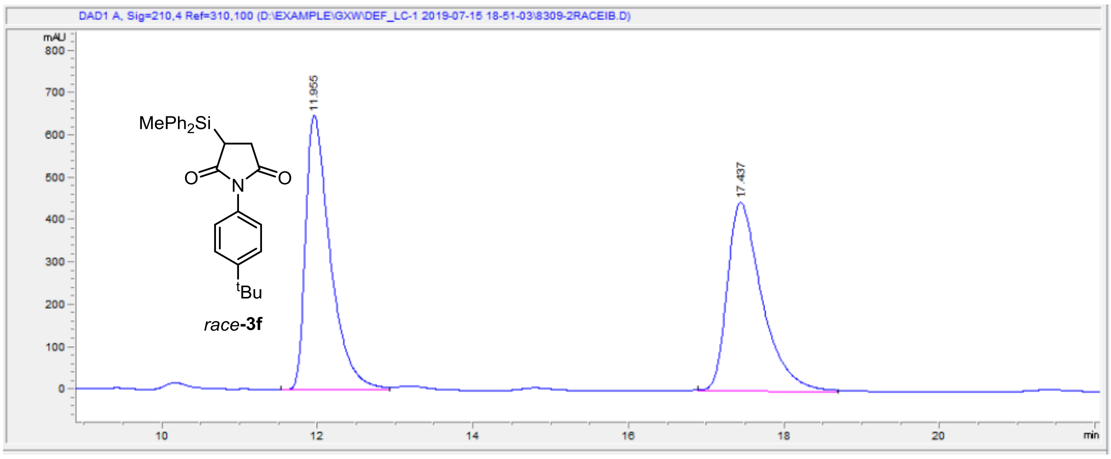

	Time/min	Area	Height	Area%
1	11.955	14330.4	647.7	50.732
2	17.437	13916.7	445.5	49.268

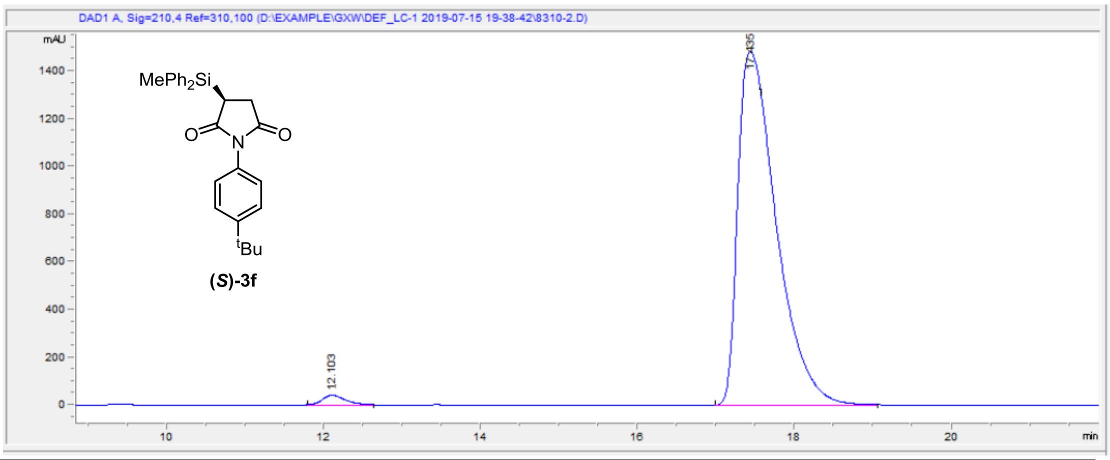

	Time/min	Area	Height	Area%
1	12.103	921.9	42.4	1.789
2	17.435	50592.9	1484.9	98.211

Supplementary Figure 226. HPLC spectra for **3f**

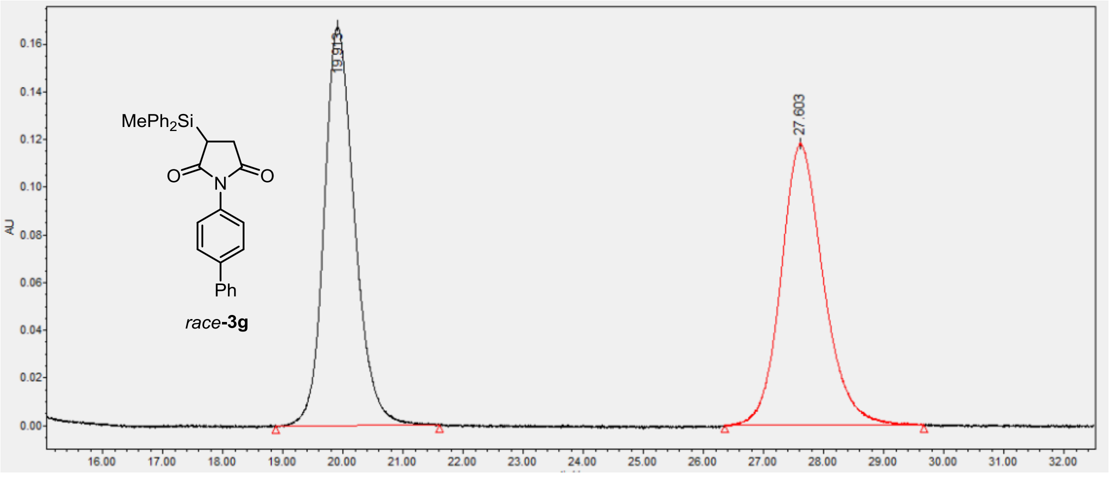

	Time/min	Area	Height	Area%
1	19.913	5871038	167574	50.54
2	27.603	5745665	118193	49.46

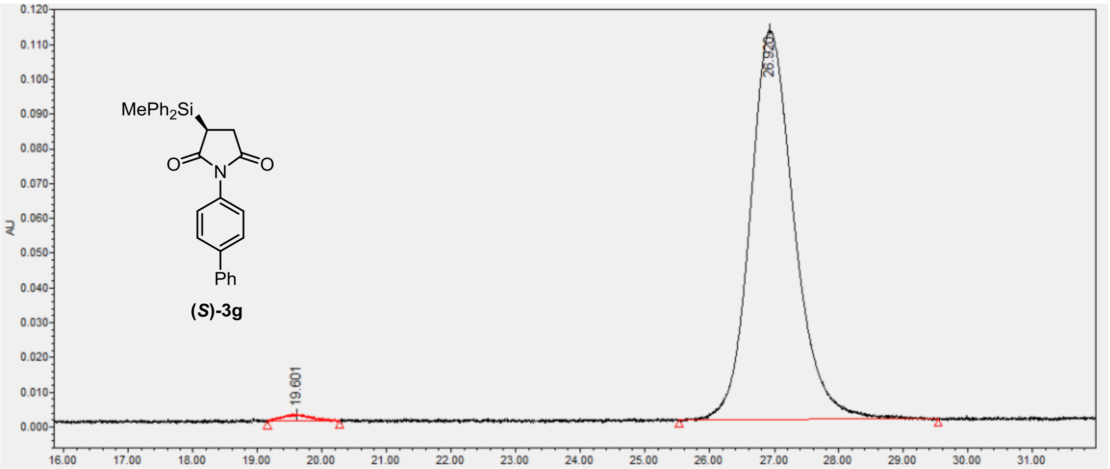

	Time/min	Area	Height	Area%
1	19.601	54705	2083	1.02
2	26.920	5311986	112188	98.98

Supplementary Figure 227. HPLC spectra for **3g**

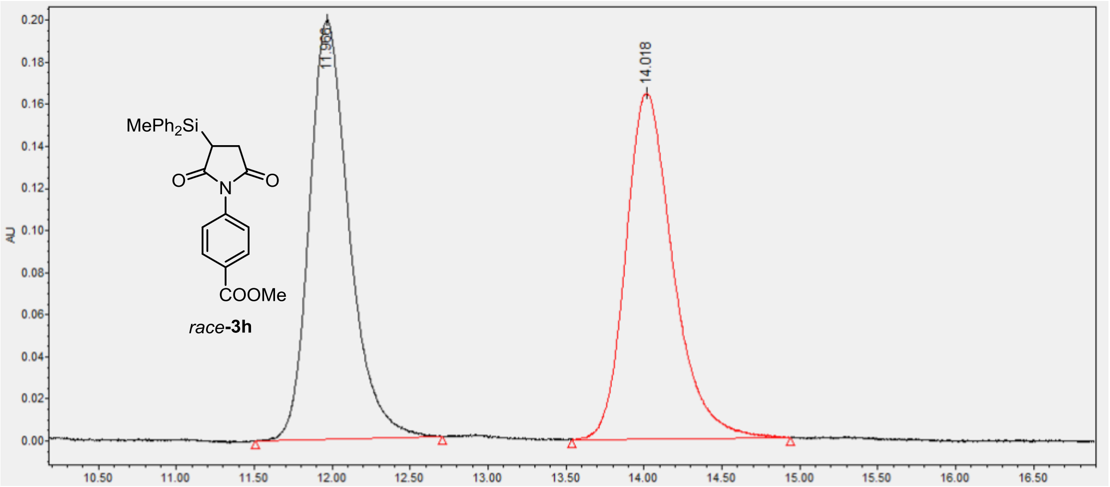

	Time/min	Area	Height	Area%
1	11.966	3528083	198949	50.37
2	14.081	3476069	164585	49.63

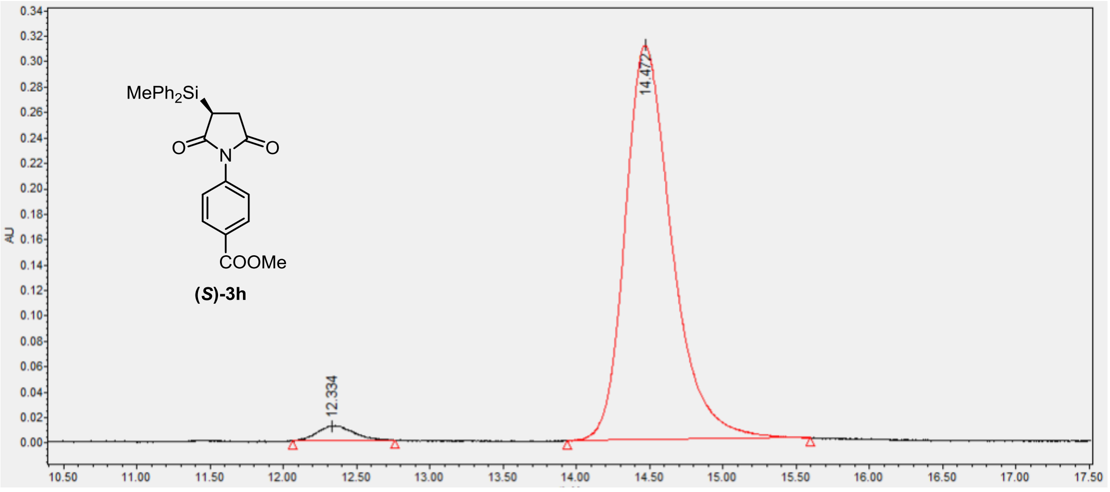

	Time/min	Area	Height	Area%
1	12.334	209103	11935	2.94
2	14.472	6896573	310256	97.06

Supplementary Figure 228. HPLC spectra for **3h**

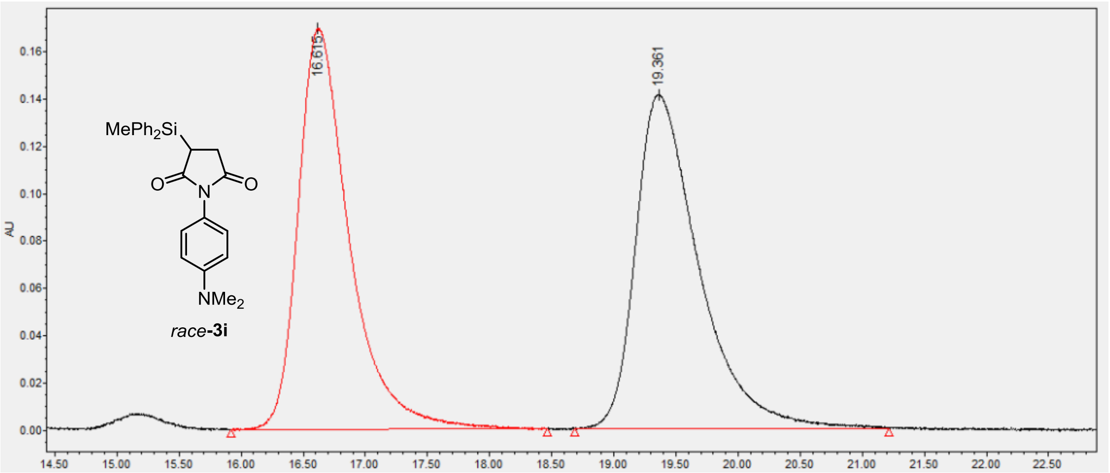

	Time/min	Area	Height	Area%
1	16.615	4863194	169596	49.28
2	19.361	5004966	141389	50.72

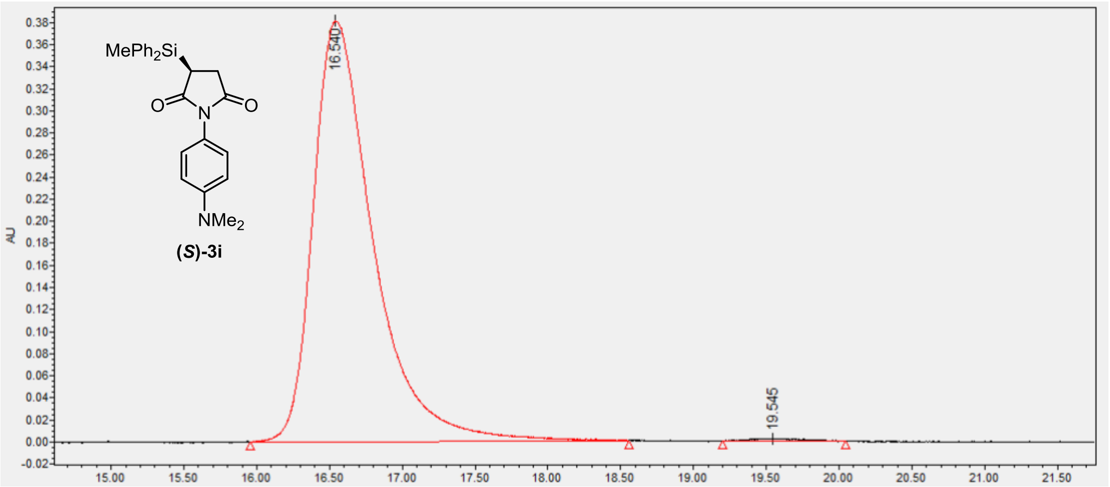

	Time/min	Area	Height	Area%
1	16.540	10945521	381228	99.59
2	19.545	44625	2167	0.41

Supplementary Figure 229. HPLC spectra for **3i**

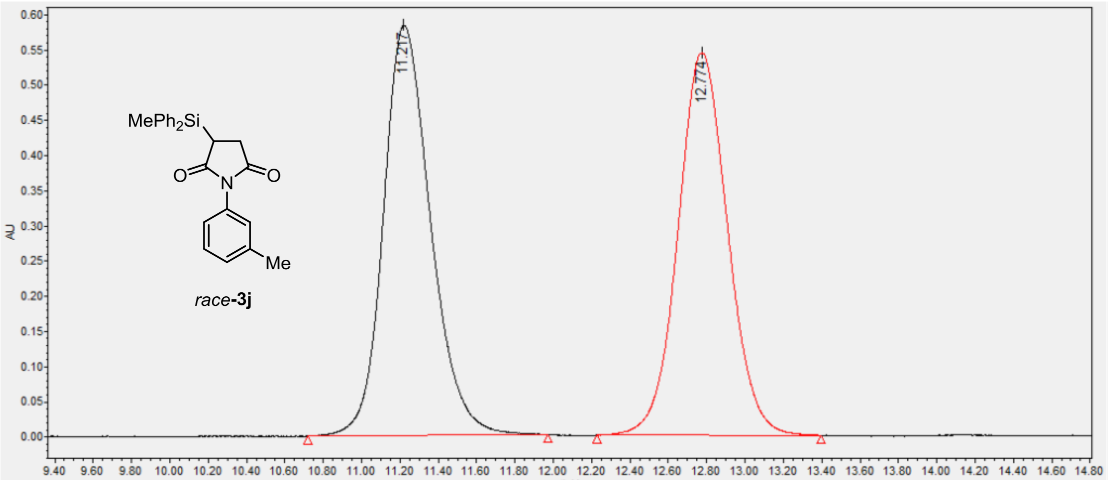

	Time/min	Area	Height	Area%
1	11.217	9993042	583516	50.57
2	12.774	9769479	542849	49.43

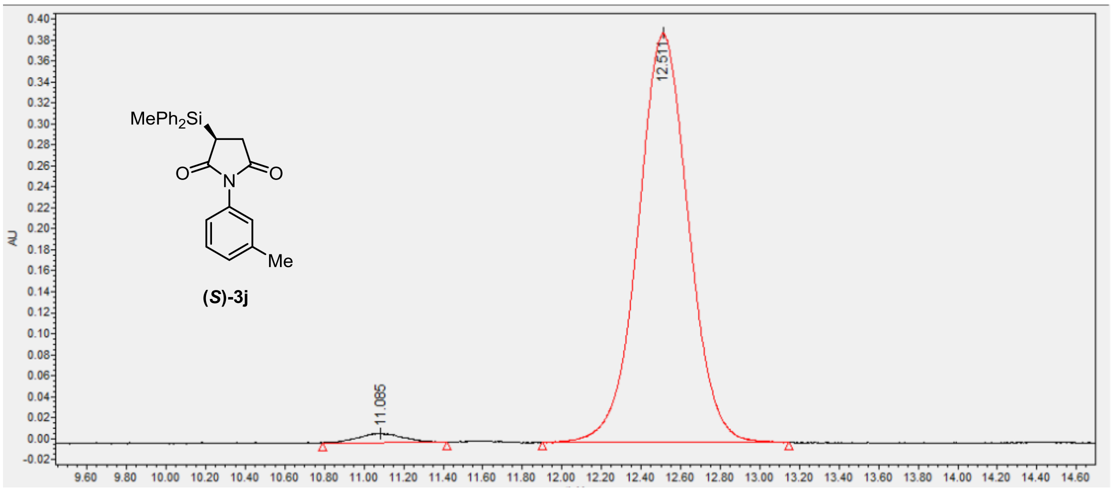

	Time/min	Area	Height	Area%
1	11.085	140600	8886	2.00
2	12.511	6882418	390185	98.00

Supplementary Figure 230. HPLC spectra for **3j**

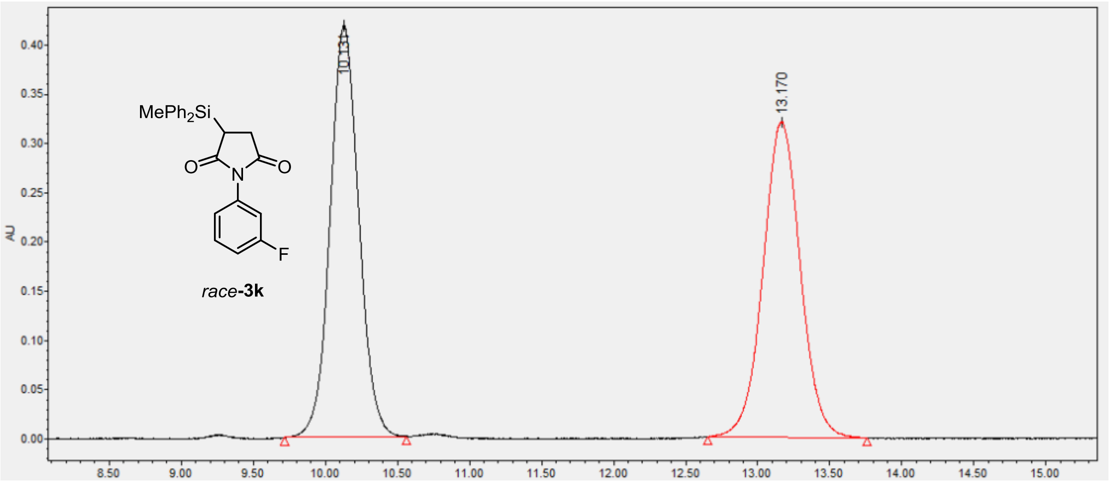

	Time/min	Area	Height	Area%
1	10.131	5786559	417134	50.04
2	13.170	5777548	320661	49.96

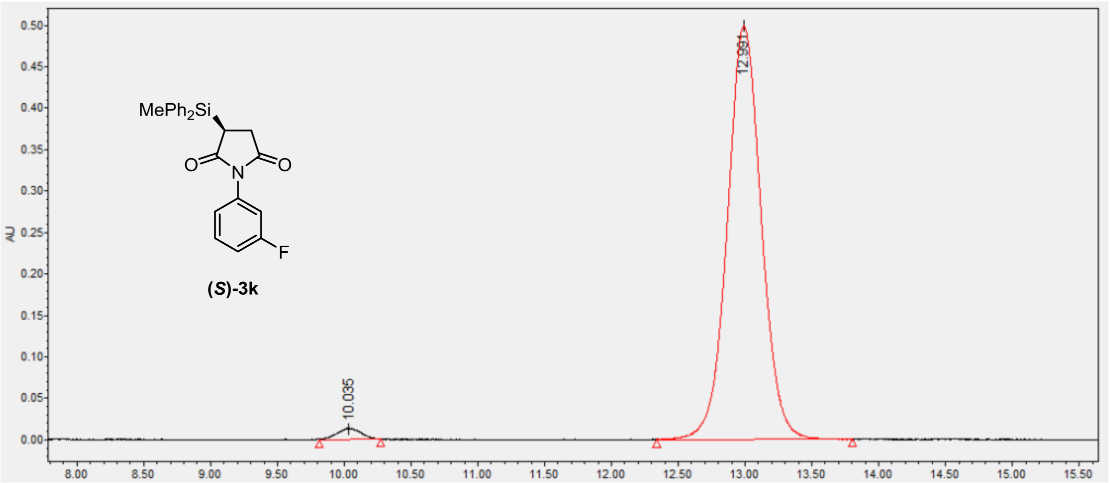

	Time/min	Area	Height	Area%
1	10.035	160580	12978	1.79
2	12.991	8800588	498565	98.21

Supplementary Figure 231. HPLC spectra for **3k**

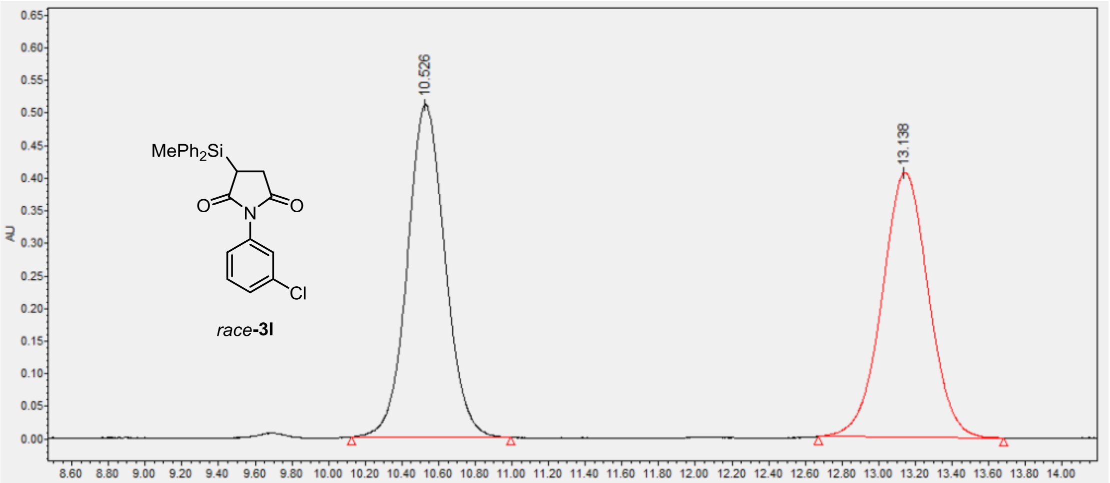

	Time/min	Area	Height	Area%
1	10.526	7286045	510708	50.52
2	13.138	7136494	406025	49.48

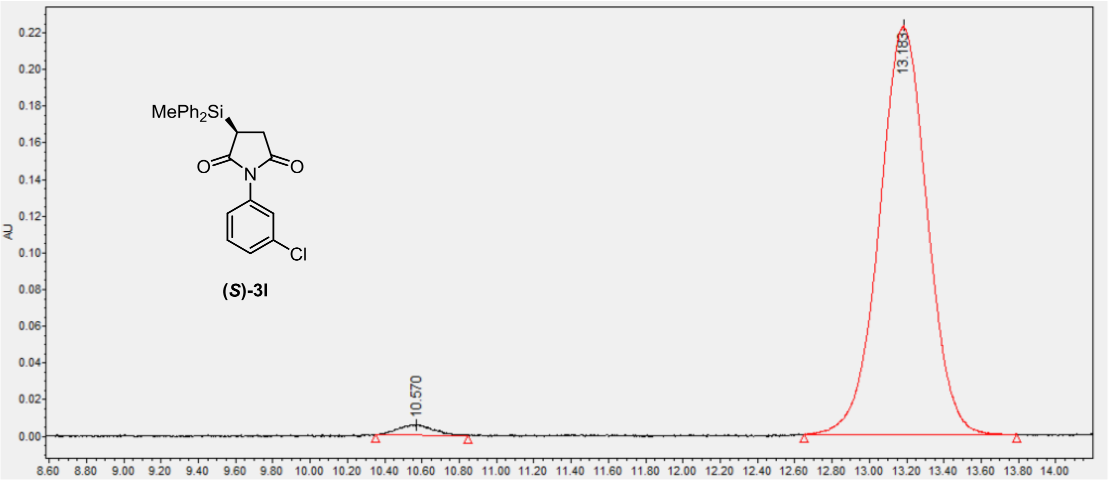

	Time/min	Area	Height	Area%
1	10.570	71784	5611	1.78
2	13.183	3960845	222996	98.22

Supplementary Figure 232. HPLC spectra for **3l**

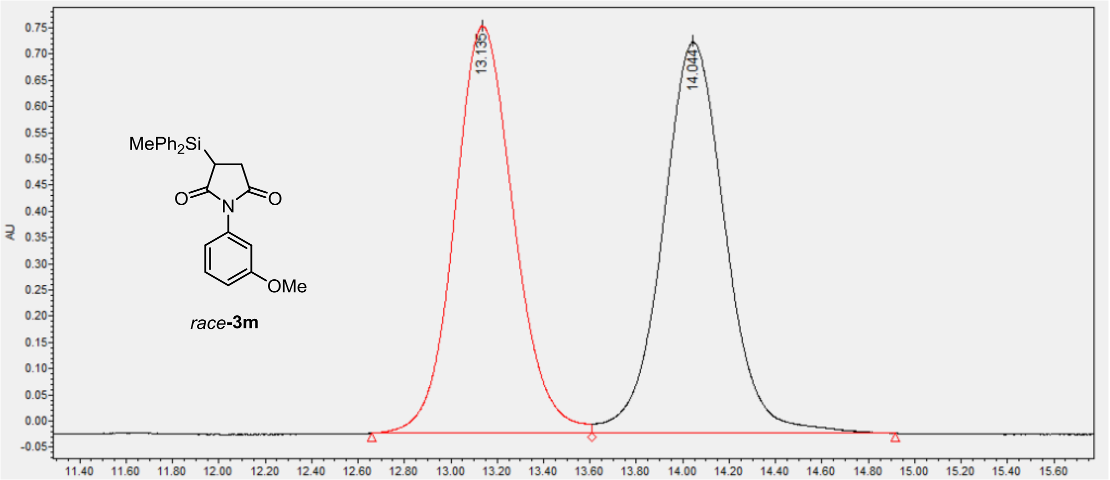

	Time/min	Area	Height	Area%
1	13.135	13783416	777259	49.58
2	14.044	14016914	747803	50.42

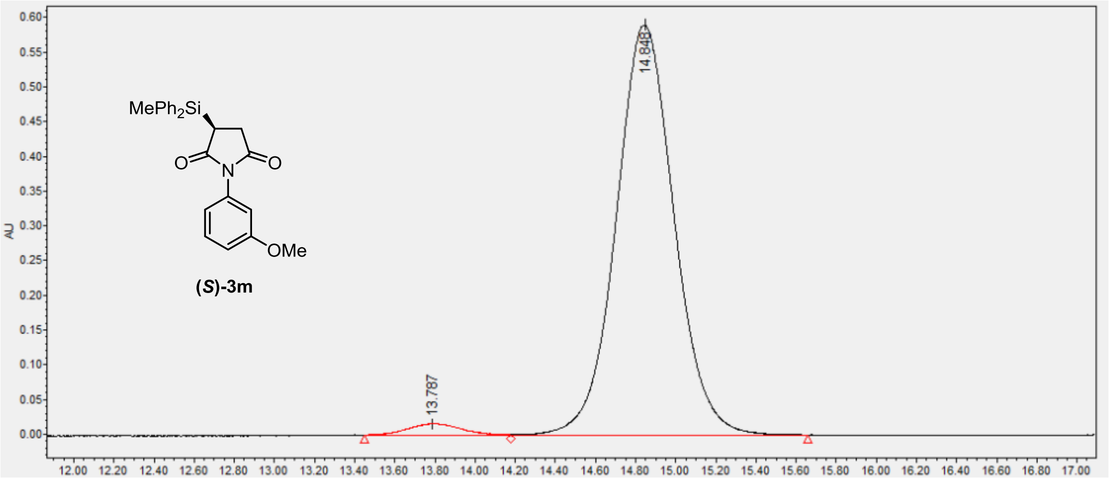

	Time/min	Area	Height	Area%
1	13.787	294217	16315	2.42
2	14.848	11847782	590571	97.58

Supplementary Figure 233. HPLC spectra for **3m**

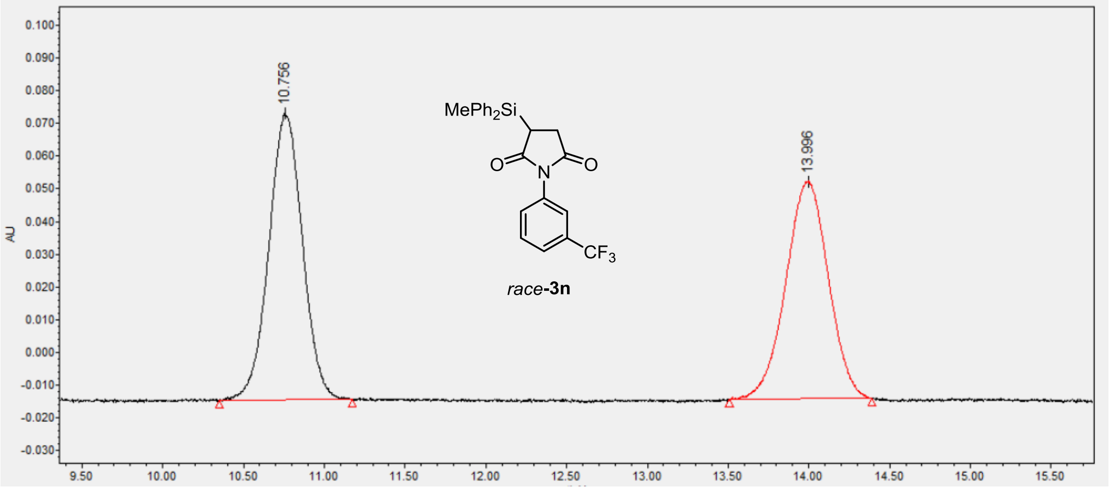

	Time/min	Area	Height	Area%
1	10.756	1267873	87793	51.00
2	13.996	1218163	66521	49.00

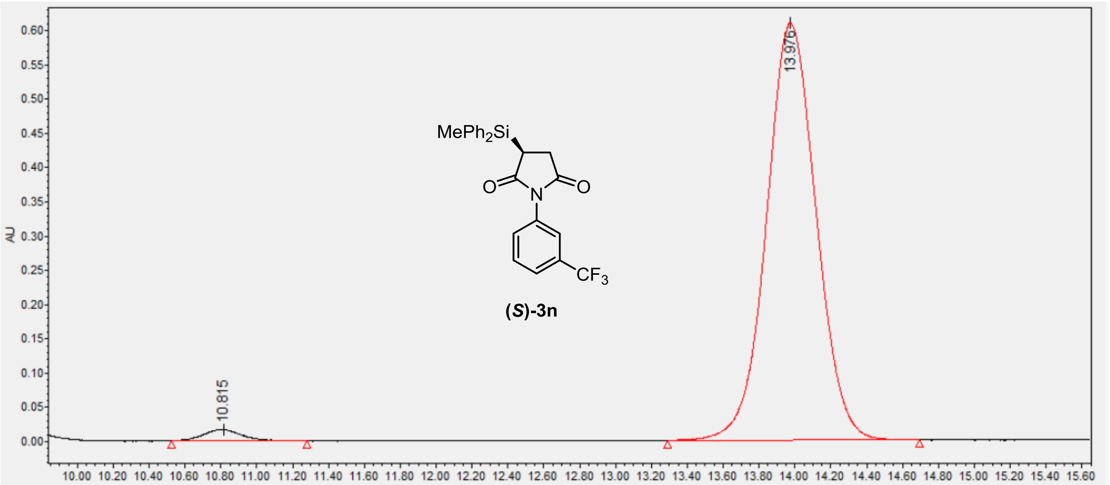

	Time/min	Area	Height	Area%
1	10.815	235609	16344	1.98
2	13.976	11681925	608503	98.02

Supplementary Figure 234. HPLC spectra for **3n**

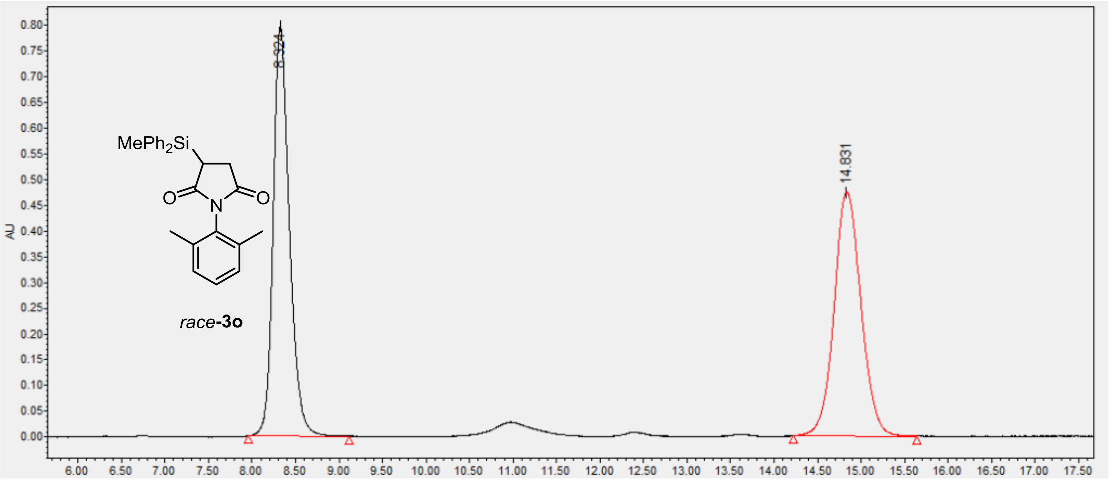

	Time/min	Area	Height	Area%
1	8.324	10305961	795064	50.36
2	14.831	10159537	473679	49.64

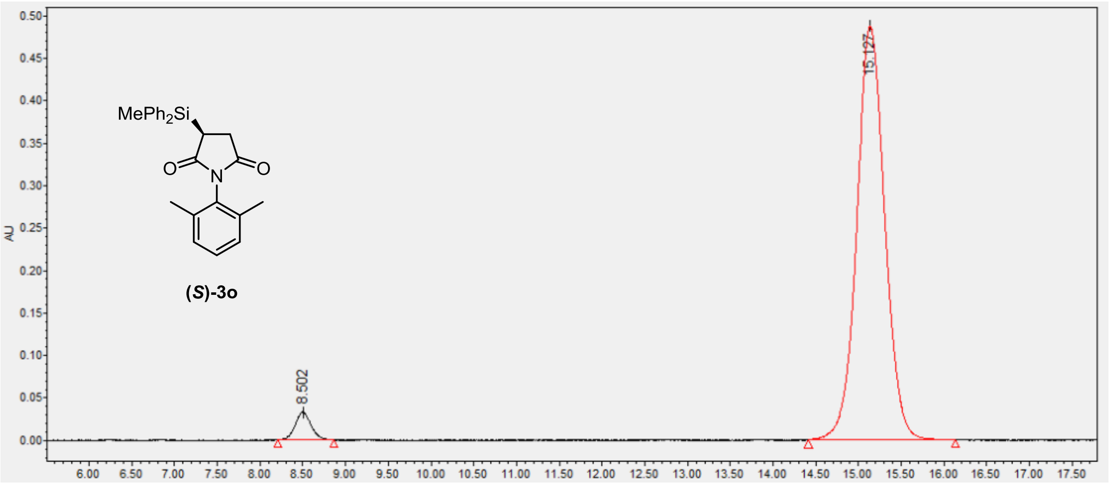

	Time/min	Area	Height	Area%
1	8.502	419906	32991	3.65
2	15.127	11074168	487397	96.35

Supplementary Figure 235. HPLC spectra for **3o**

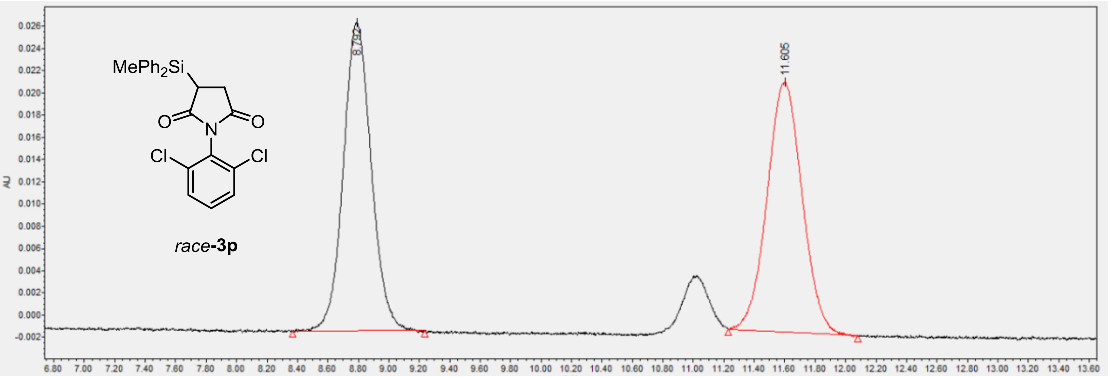

	Time/min	Area	Height	Area%
1	8.792	354846	27733	49.63
2	11.605	360091	22545	50.37

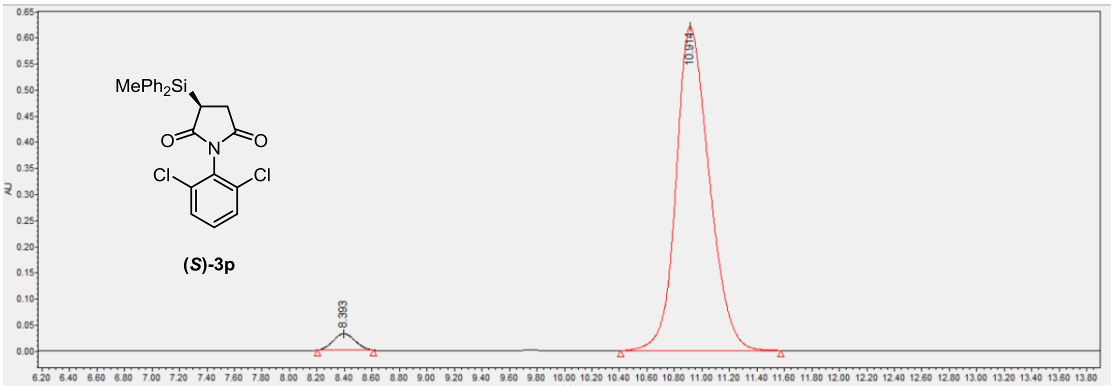

	Time/min	Area	Height	Area%
1	8.393	345038	31392	3.21
2	10.914	10415921	620322	96.79

Supplementary Figure 236. HPLC spectra for **3p**

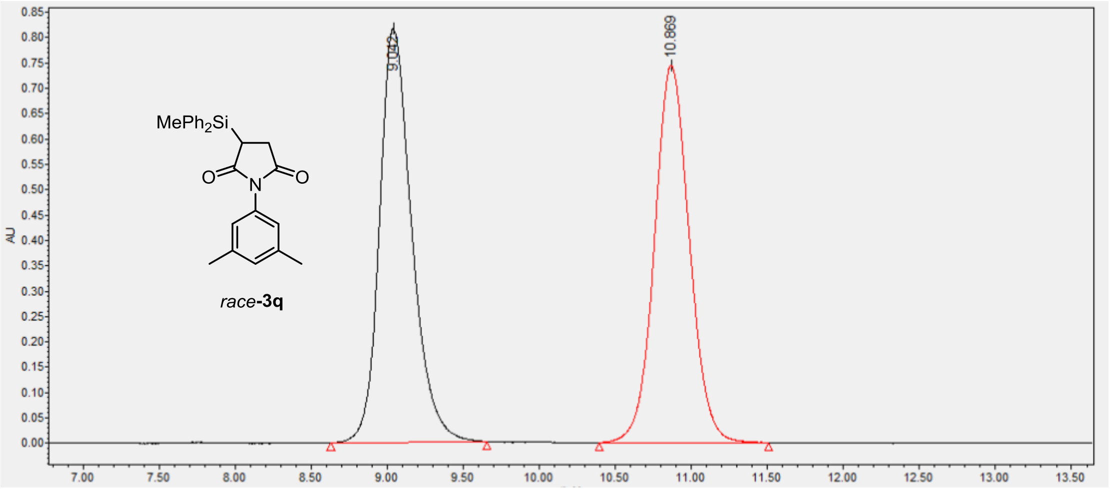

	Time/min	Area	Height	Area%
1	9.042	12023805	815885	50.31
2	10.869	11877757	744267	49.69

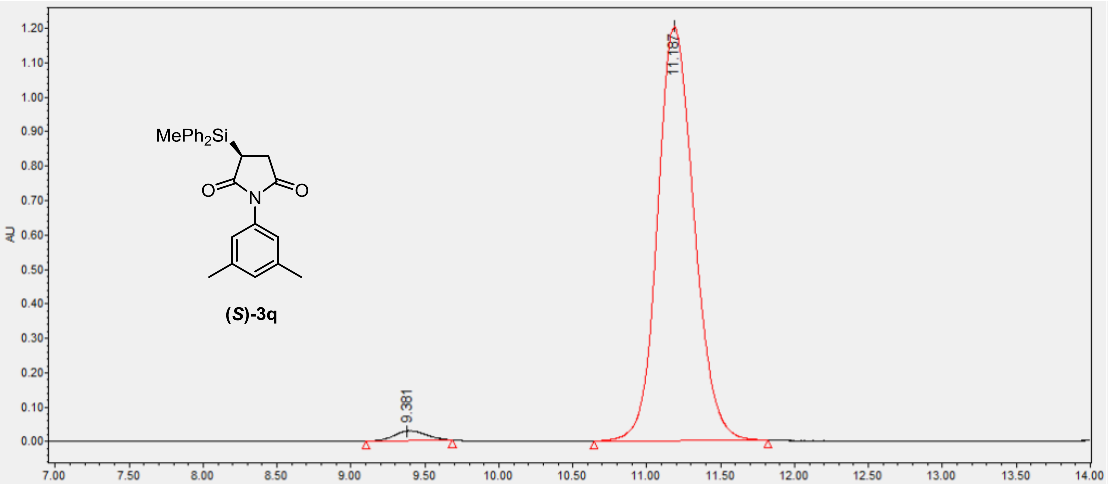

	Time/min	Area	Height	Area%
1	9.381	410831	28183	1.95
2	11.187	20676096	1202827	98.05

Supplementary Figure 237. HPLC spectra for **3q**

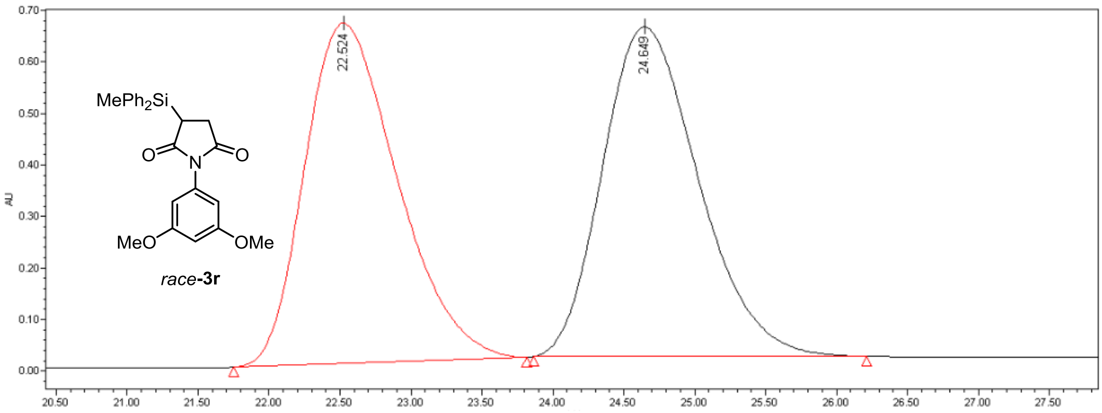

	Time/min	Area	Height	Area%
1	22.524	29963799	660152	49.86
2	24.649	30127664	639795	50.14

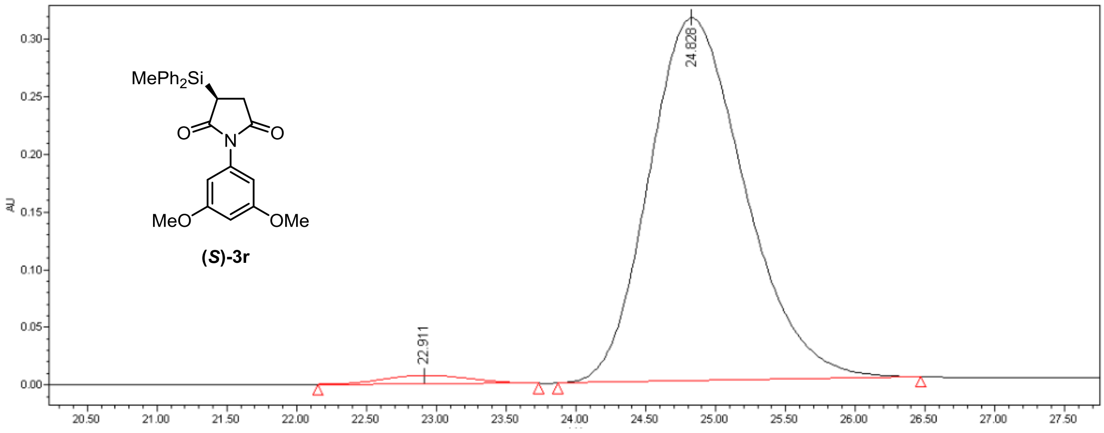

	Time/min	Area	Height	Area%
1	22.911	311382	7383	2.03
2	24.828	15042195	315125	97.97

Supplementary Figure 238. HPLC spectra for **3r**

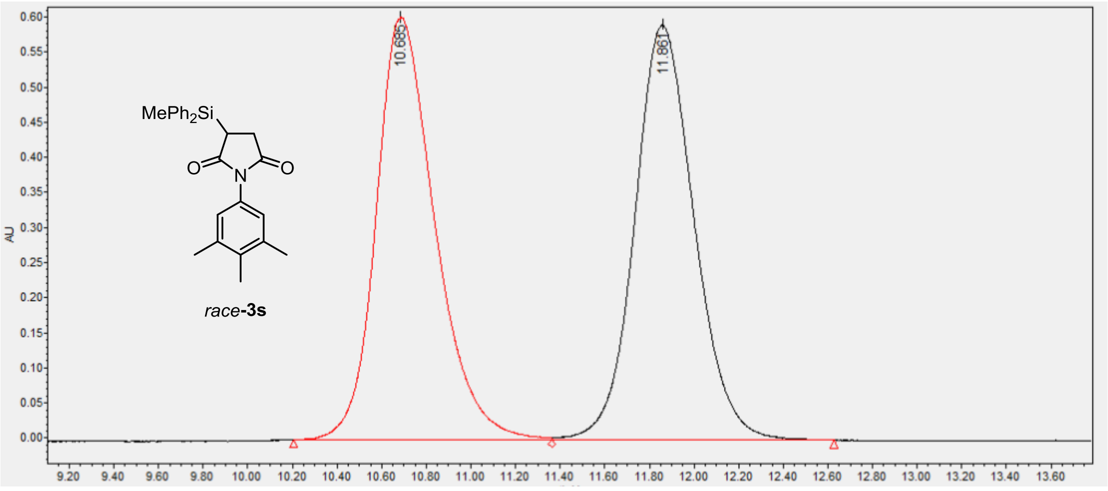

	Time/min	Area	Height	Area%
1	10.685	11010810	602011	50.17
2	11.861	10934558	591854	49.83

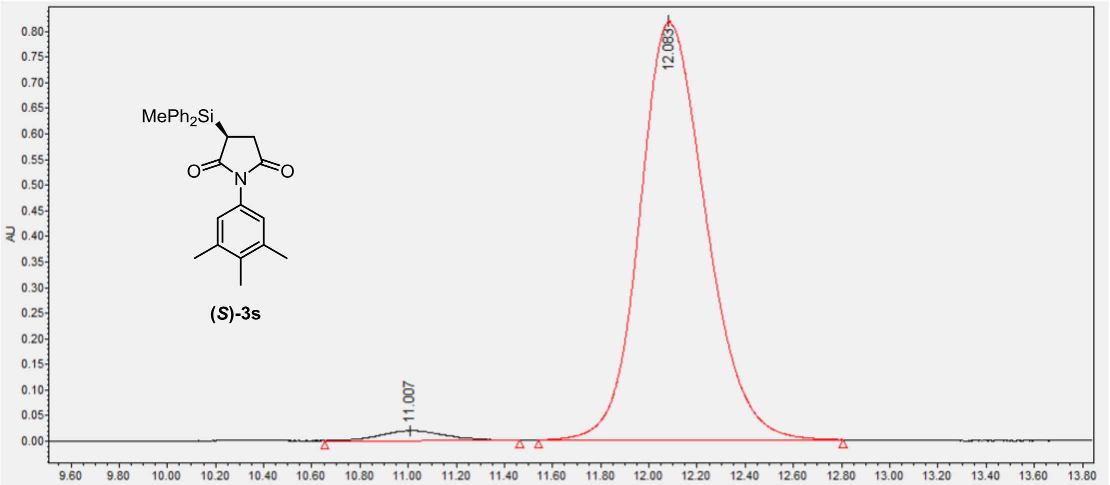

	Time/min	Area	Height	Area%
1	11.007	355334	19537	2.23
2	12.083	15610583	817230	97.77

Supplementary Figure 239. HPLC spectra for **3s**

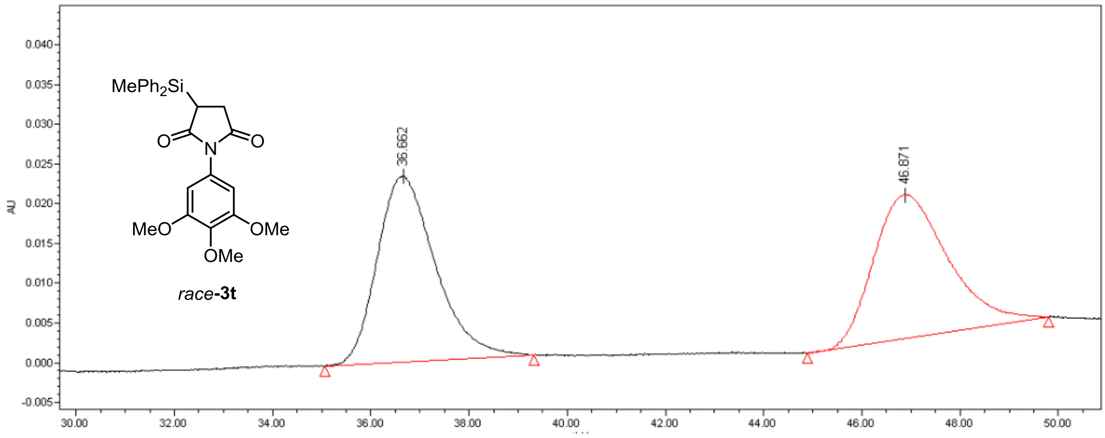

	Time/min	Area	Height	Area%
1	36.662	1920333	23358	50.29
2	46.871	1898043	18063	49.71

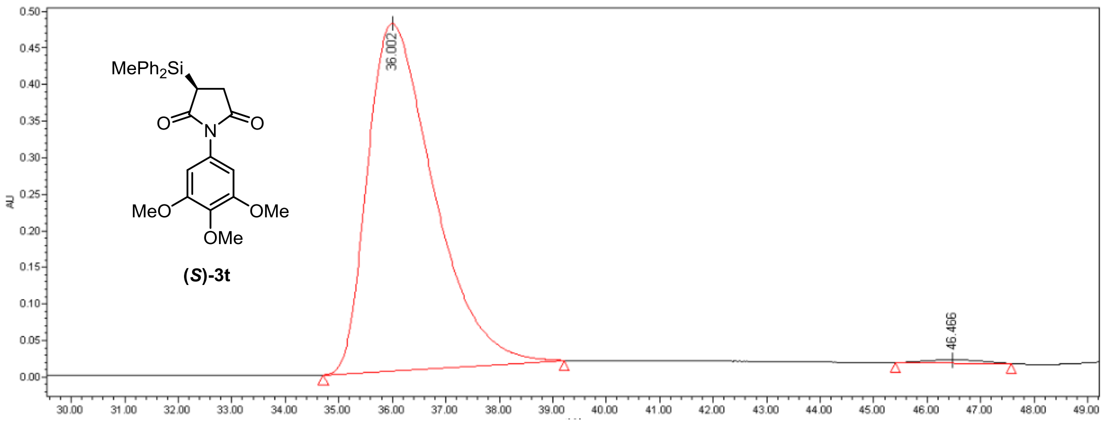

	Time/min	Area	Height	Area%
1	36.002	40847655	474833	99.22
2	46.466	319833	4350	0.78

Supplementary Figure 240. HPLC spectra for **3t**

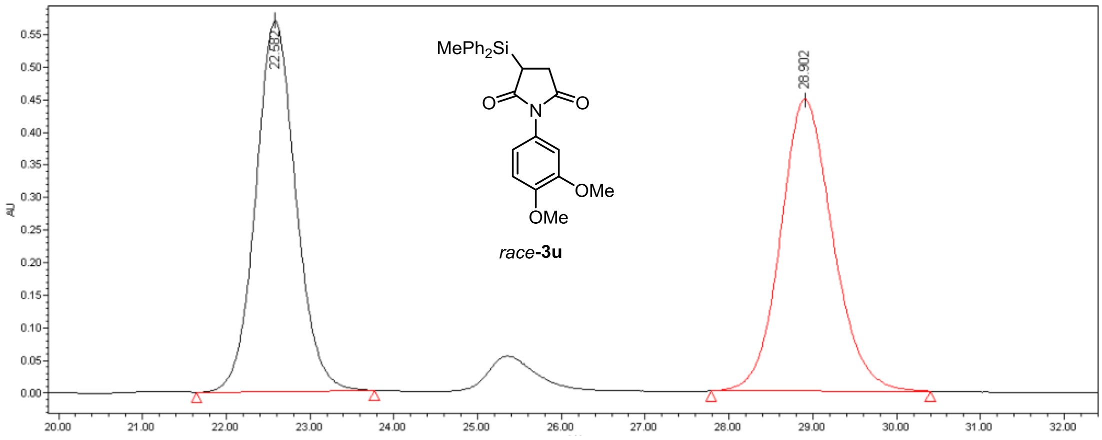

	Time/min	Area	Height	Area%
1	22.582	18912725	568829	49..42
2	28.902	19353020	447721	50.58

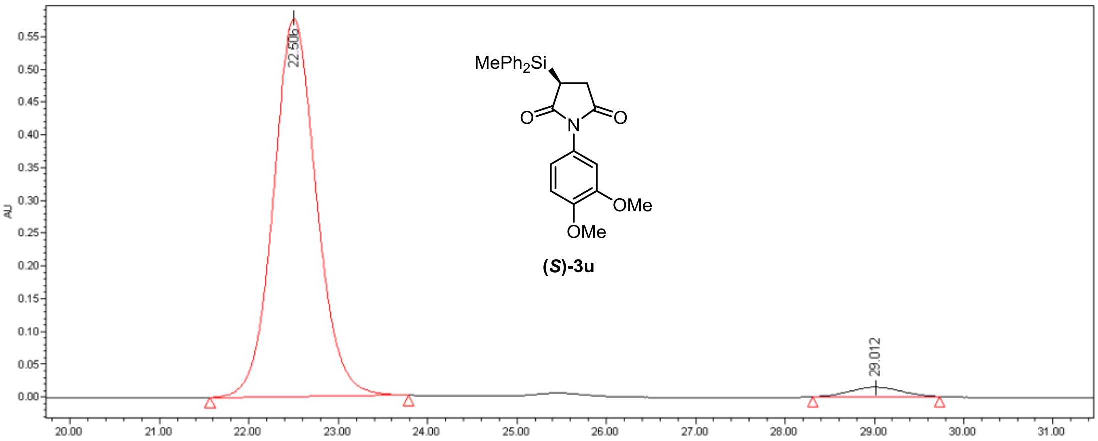

	Time/min	Area	Height	Area%
1	22.506	19331018	576921	97.07
2	29.012	582953	14510	2.93

Supplementary Figure 241. HPLC spectra for **3u**

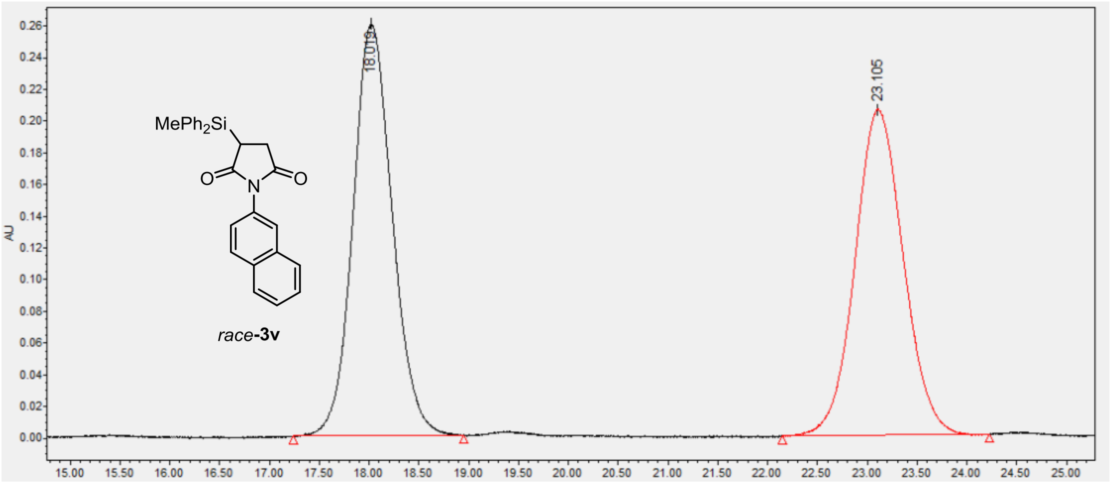

	Time/min	Area	Height	Area%
1	18.019	7121839	259729	50.70
2	23.105	6925607	205629	49.30

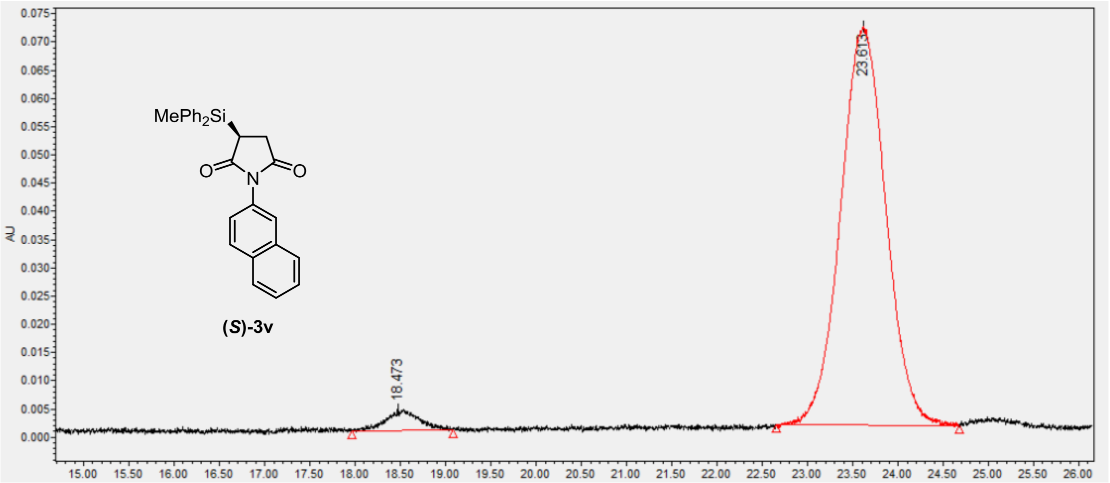

	Time/min	Area	Height	Area%
1	18.473	92565	3783	3.64
2	23.613	2450270	70532	96.36

Supplementary Figure 242. HPLC spectra for **3v**

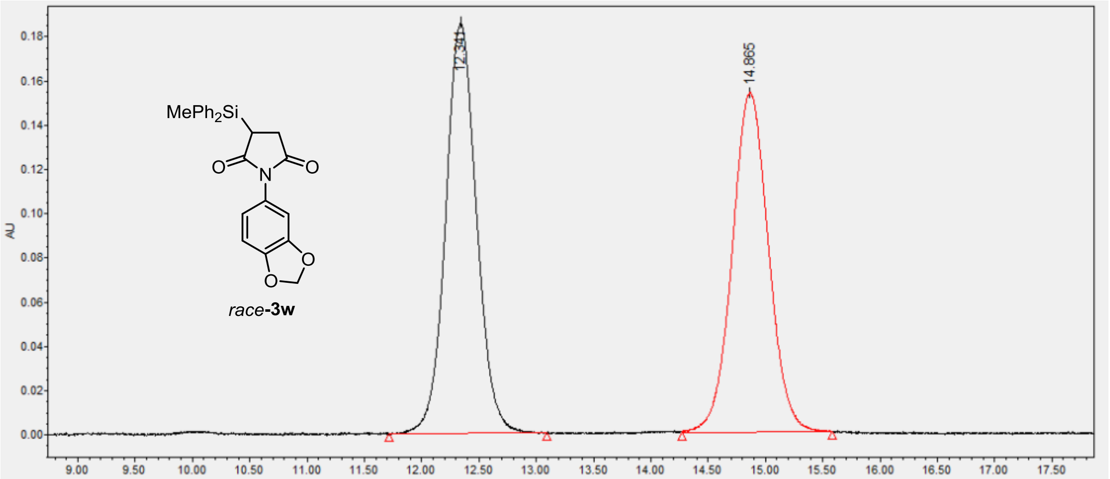

	Time/min	Area	Height	Area%
1	12.341	3476122	185620	50.71
2	14.865	3379313	153598	49.29

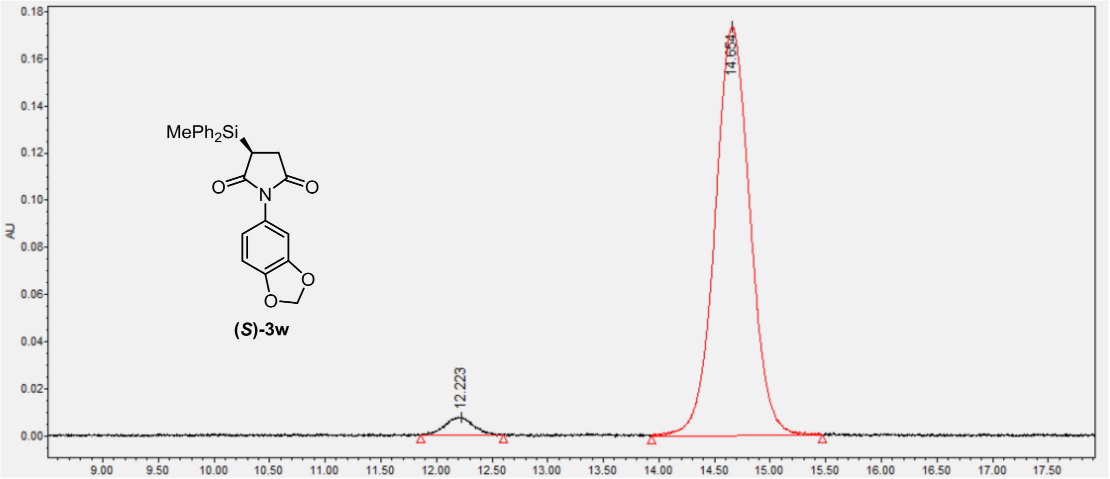

	Time/min	Area	Height	Area%
1	12.223	138380	7799	3.52
2	14.654	3788396	173156	96.48

Supplementary Figure 243. HPLC spectra for **3w**

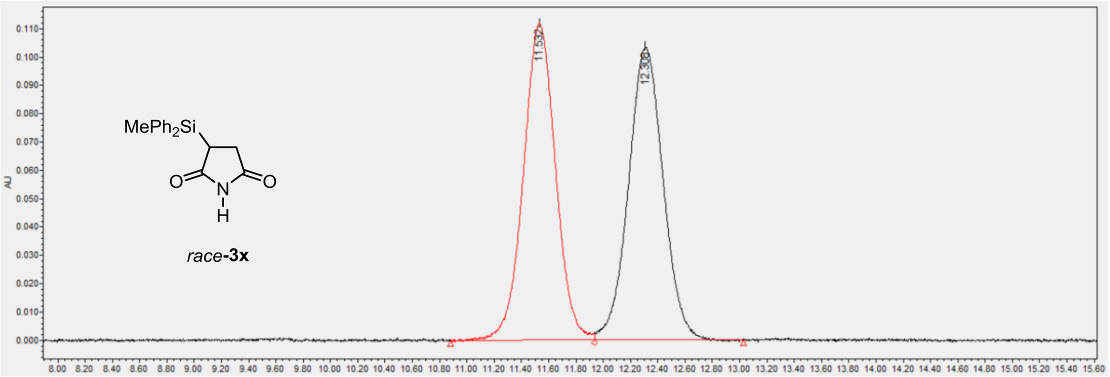

	Time/min	Area	Height	Area%
1	11.532	1835246	111852	50.12
2	12.308	1826563	103631	49.88

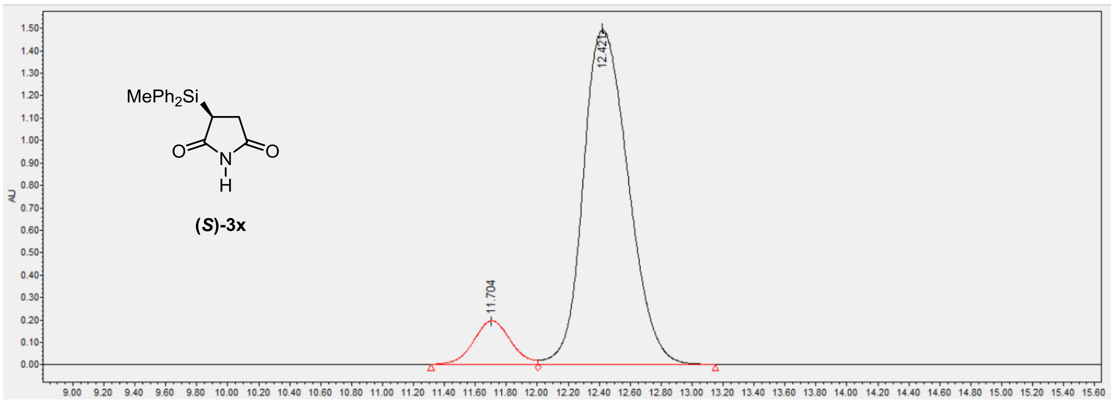

	Time/min	Area	Height	Area%
1	11.704	3200955	194026	9.61
2	12.421	30103403	1496967	90.39

Supplementary Figure 244. HPLC spectra for **3x**

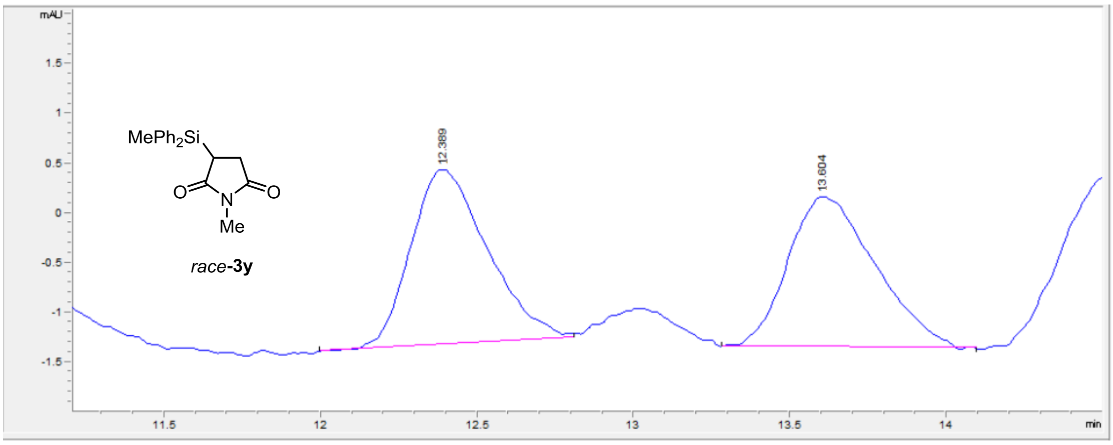

	Time/min	Area	Height	Area%
1	12.389	29.9	1.8	50.418
2	13.604	29.4	1.5	49.582

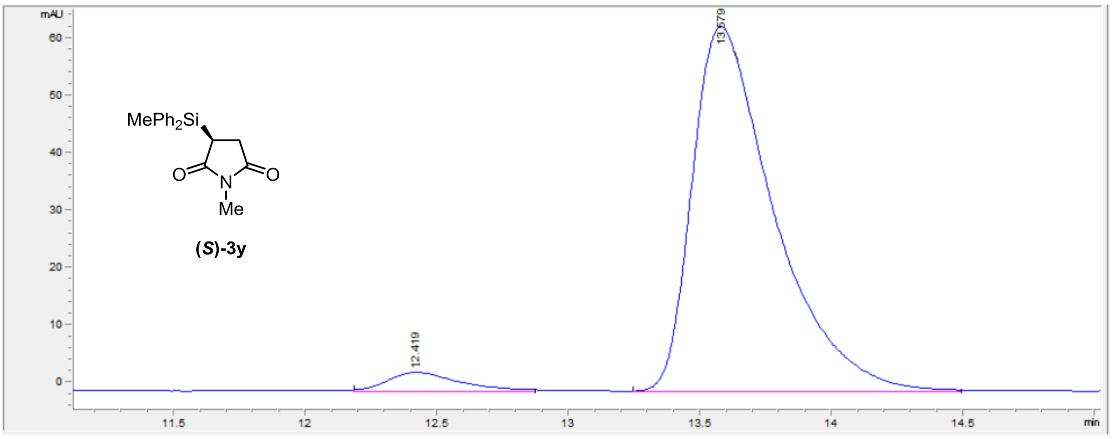

	Time/min	Area	Height	Area%
1	12.419	64.2	3.3	4.465
2	13.579	1374.7	63.6	95.535

Supplementary Figure 245. HPLC spectra for **3y**

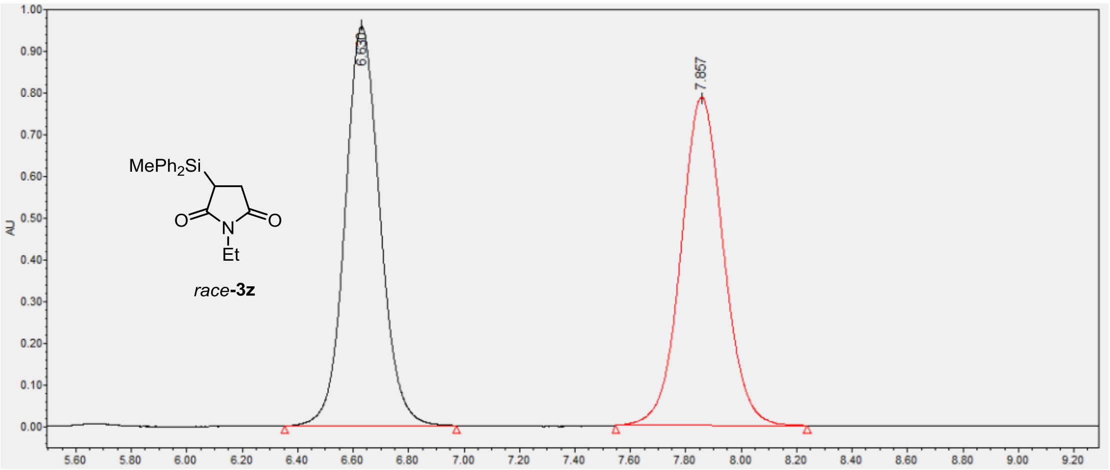

	Time/min	Area	Height	Area%
1	6.630	8366274	957976	50.00
2	7.857	8366551	785141	50.00

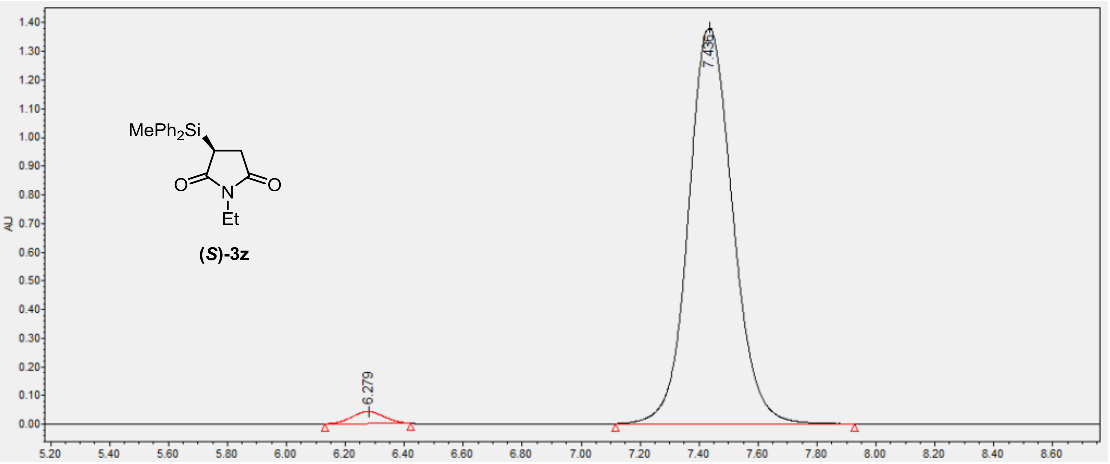

	Time/min	Area	Height	Area%
1	6.279	317856	41930	2.17
2	7.436	14350751	1380853	97.83

Supplementary Figure 246. HPLC spectra for **3z**

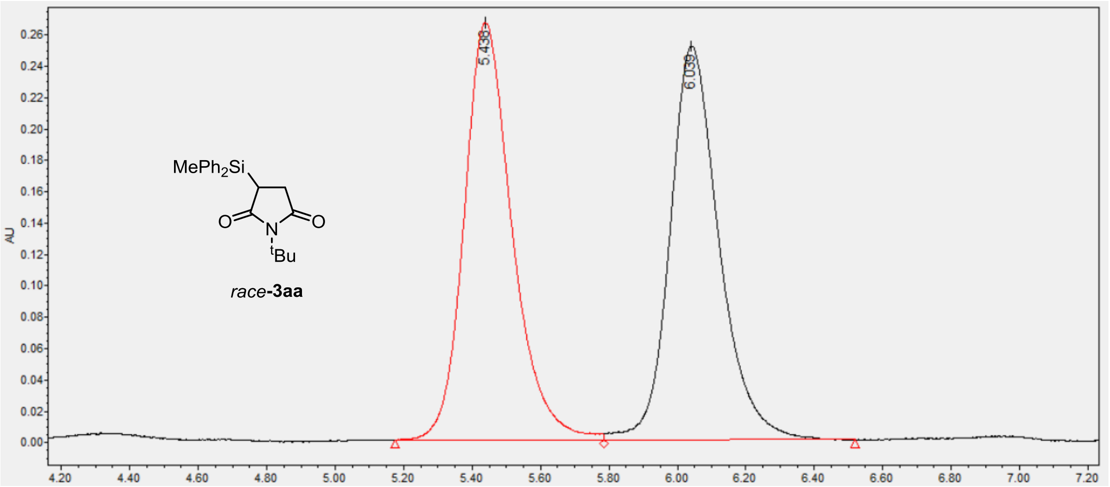

	Time/min	Area	Height	Area%
1	5.438	2603252	266057	50.92
2	6.039	2509206	250780	49.08

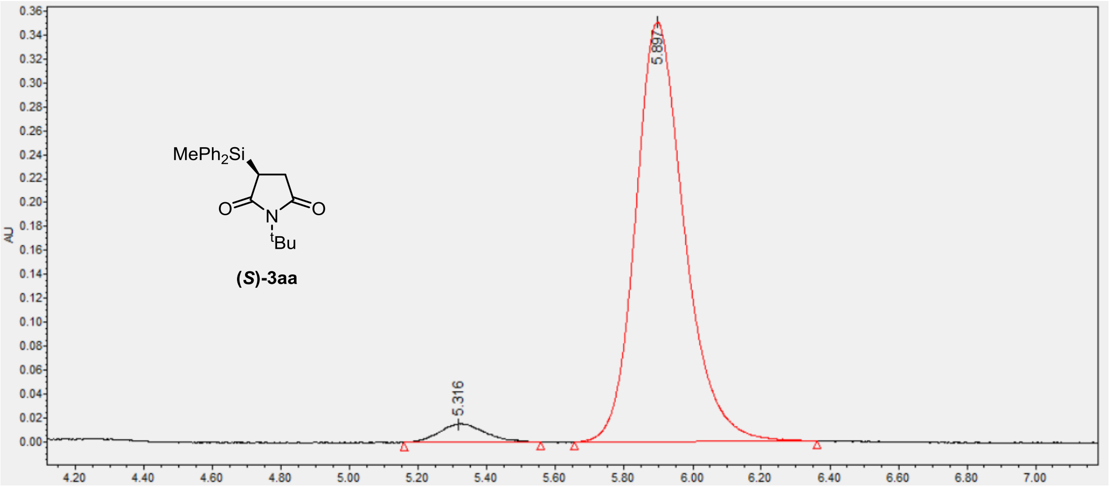

	Time/min	Area	Height	Area%
1	5.316	145283	15409	4.04
2	5.897	3453768	350042	95.96

Supplementary Figure 247. HPLC spectra for **3aa**

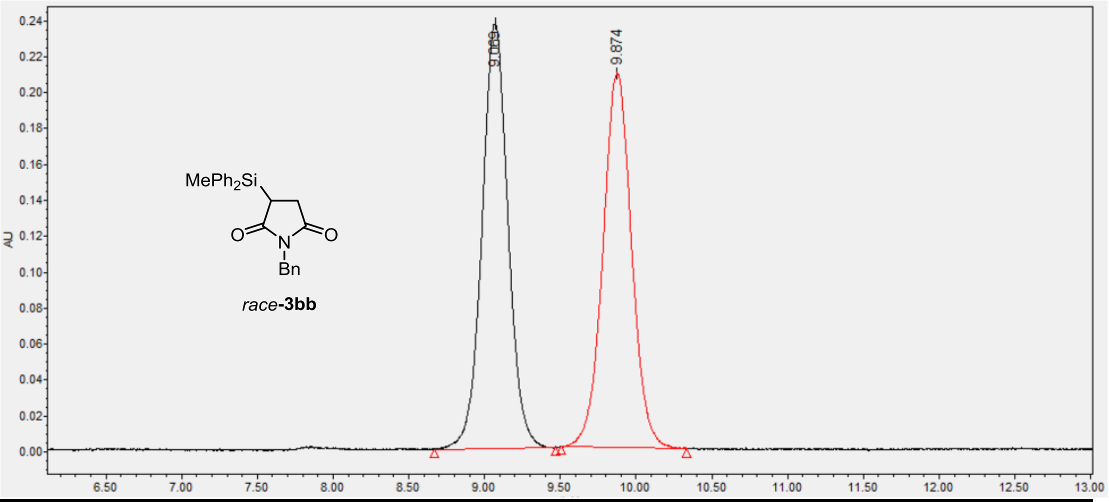

	Time/min	Area	Height	Area%
1	9.069	2818716	236410	50.67
2	9.874	2744666	208597	49.33

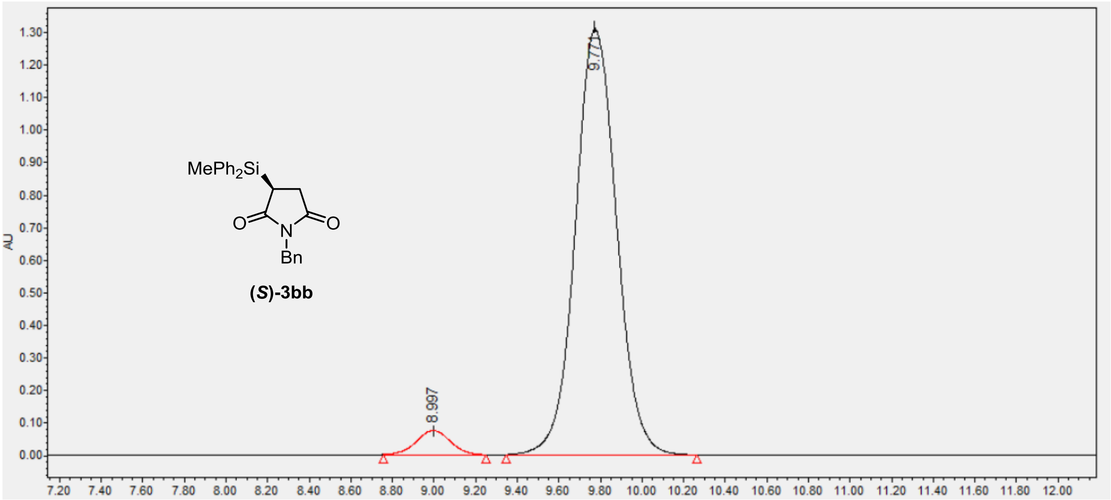

	Time/min	Area	Height	Area%
1	8.997	836793	74157	4.43
2	9.771	18064884	1313519	95.57

Supplementary Figure 248. HPLC spectra for **3bb**

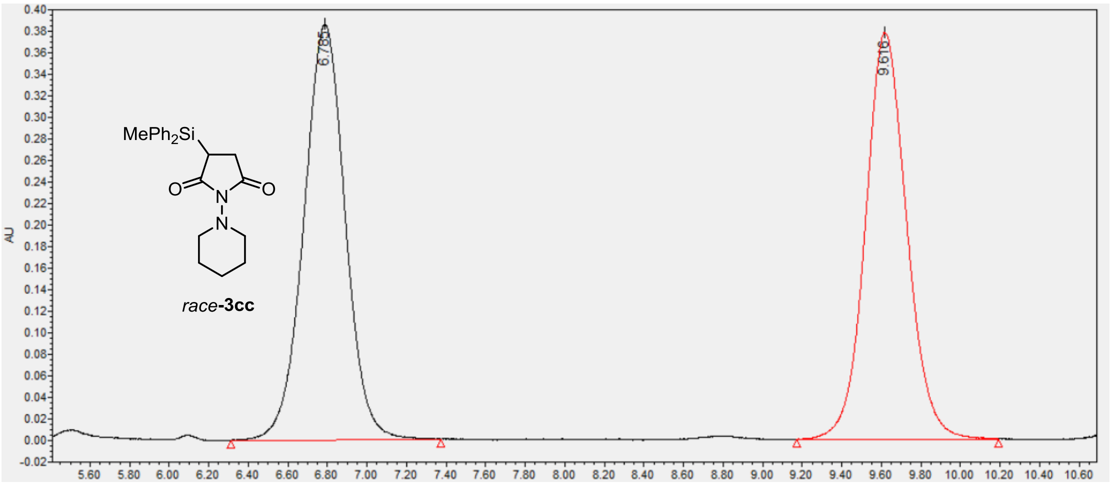

	Time/min	Area	Height	Area%
1	6.785	5625217	386406	50.66
2	9.616	5478882	377267	49.34

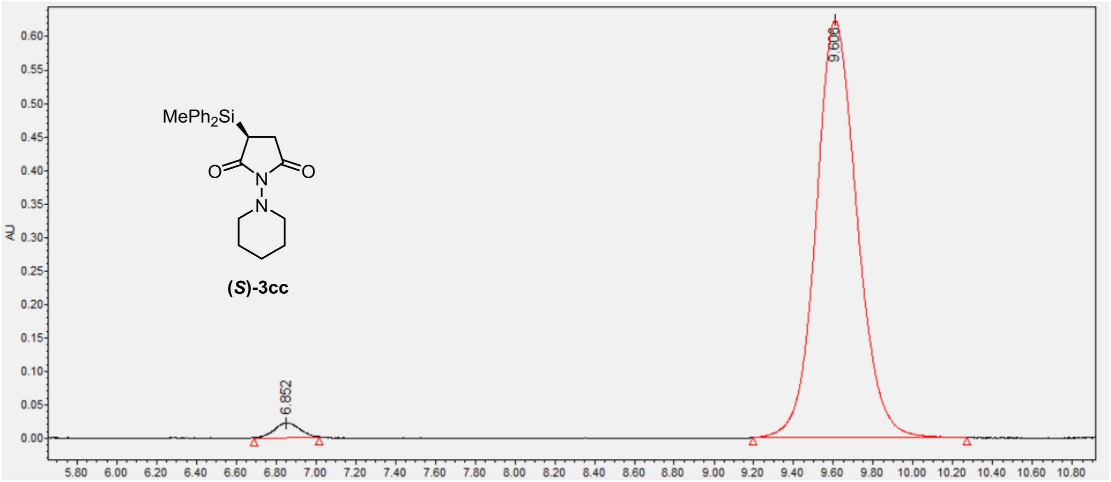

	Time/min	Area	Height	Area%
1	6.852	186469	21770	2.05
2	9.606	8915430	623526	97.95

Supplementary Figure 249. HPLC spectra for **3cc**

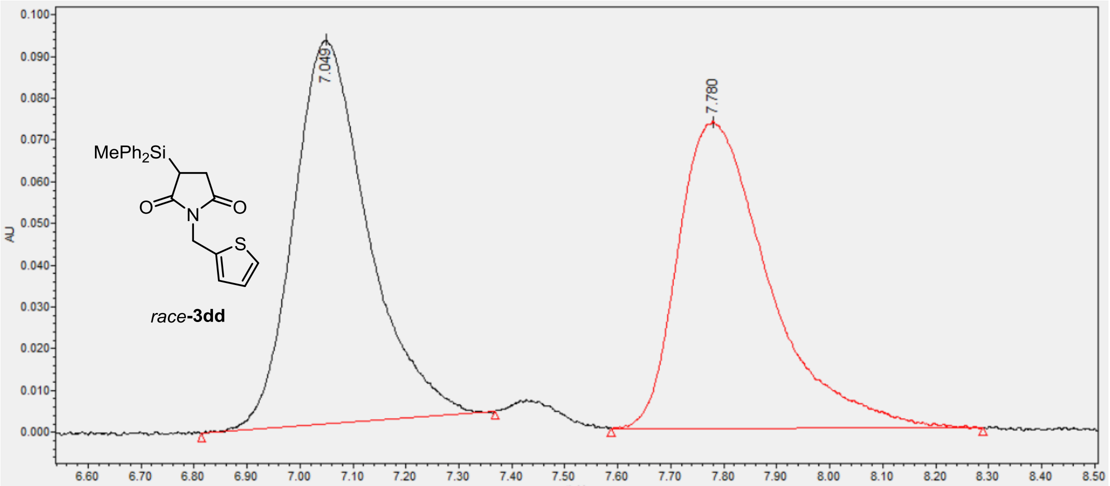

	Time/min	Area	Height	Area%
1	7.049	898209	91956	50.72
2	7.780	872682	73484	49.28

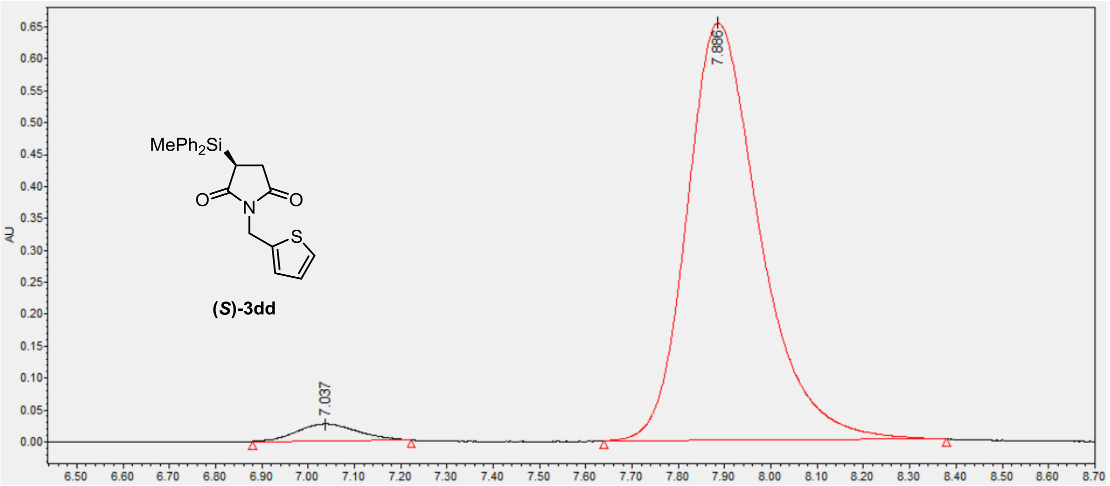

	Time/min	Area	Height	Area%
1	7.037	242026	26509	3.27
2	7.886	7150508	653591	96.73

Supplementary Figure 250. HPLC spectra for **3dd**

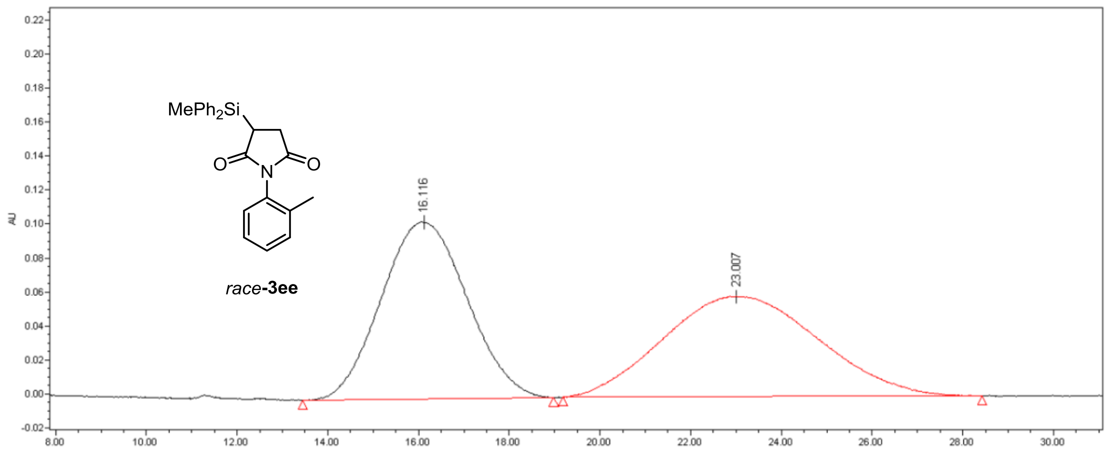

	Time/min	Area	Height	Area%
1	16.116	13719958	104272	49.49
2	23.007	14003570	59134	50.51

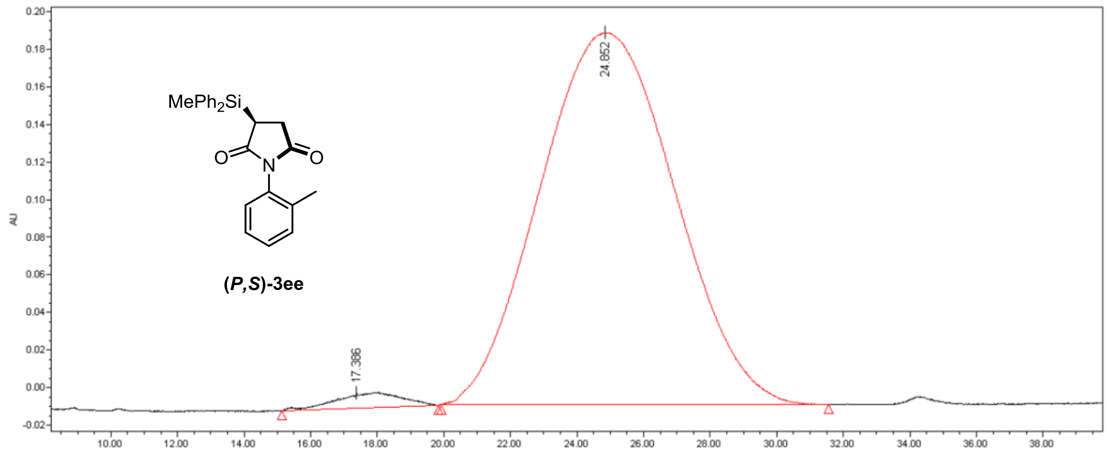

	Time/min	Area	Height	Area%
1	17.386	1070072	7792	1.88
2	24.852	55719688	197597	98.12

Supplementary Figure 251. HPLC spectra for **3ee**

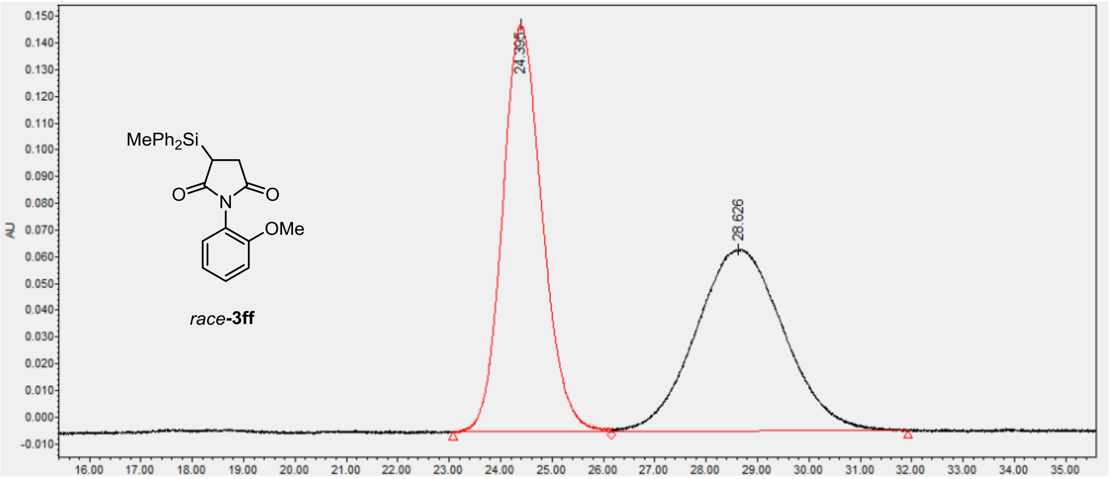

	Time/min	Area	Height	Area%
1	24.395	8165607	152124	50.37
2	28.626	8044979	68035	49.63

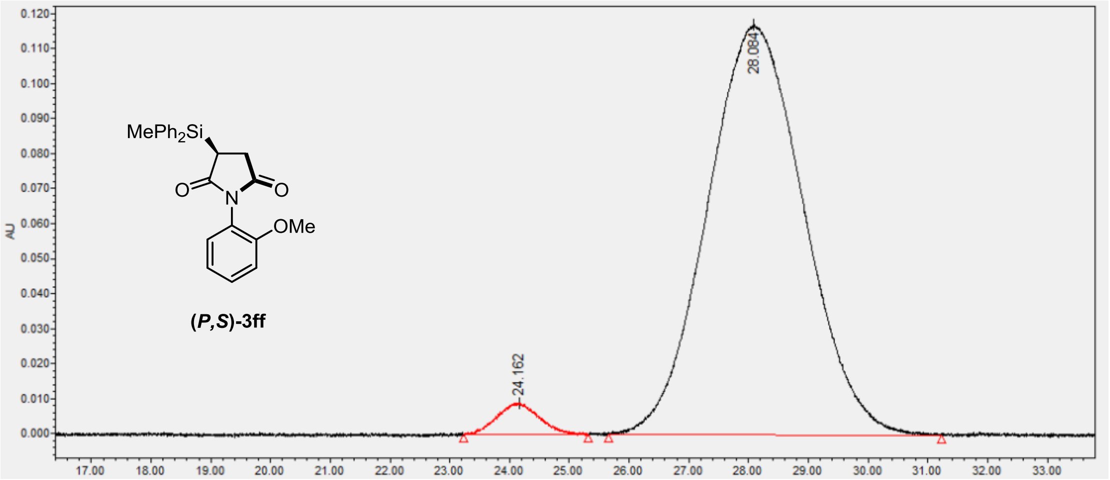

	Time/min	Area	Height	Area%
1	24.162	437011	9017	3.22
2	28.084	13117254	117170	96.78

Supplementary Figure 252. HPLC spectra for **3ff**

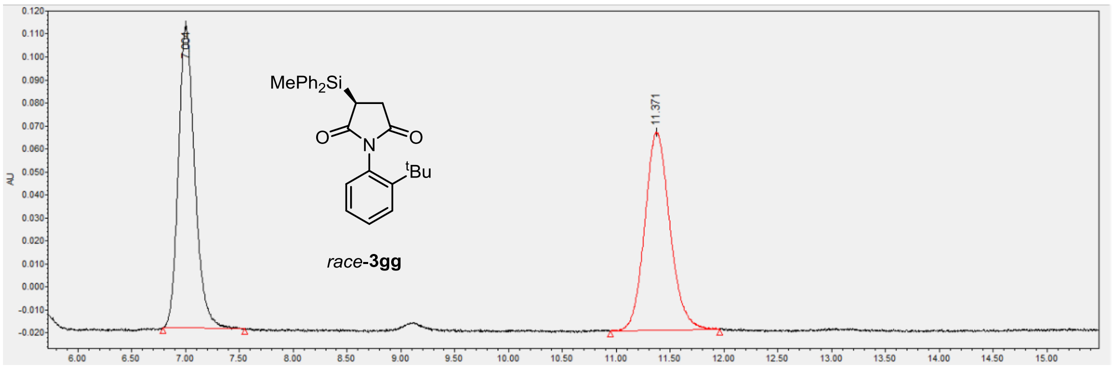

	Time/min	Area	Height	Area%
1	7.004	1387884	131477	49.83
2	11.371	1397183	86258	50.17

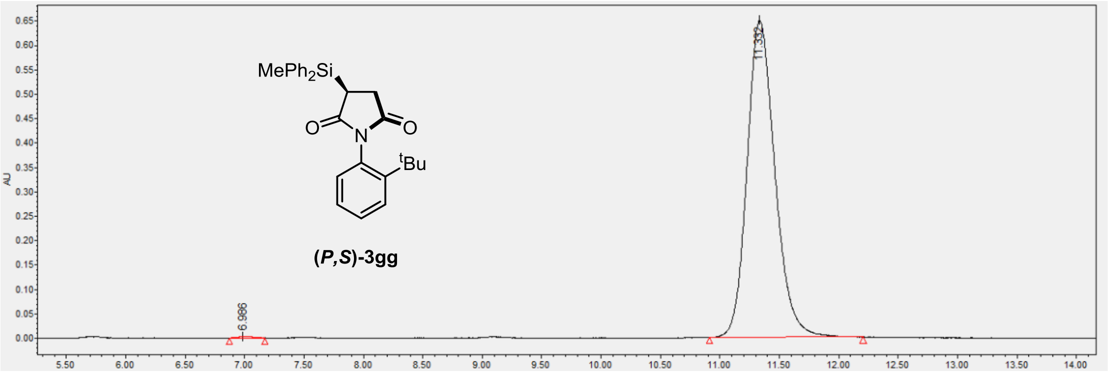

	Time/min	Area	Height	Area%
1	6.986	28430	3468	0.27
2	11.332	10596007	649601	99.73

Supplementary Figure 253. HPLC spectra for **3gg**

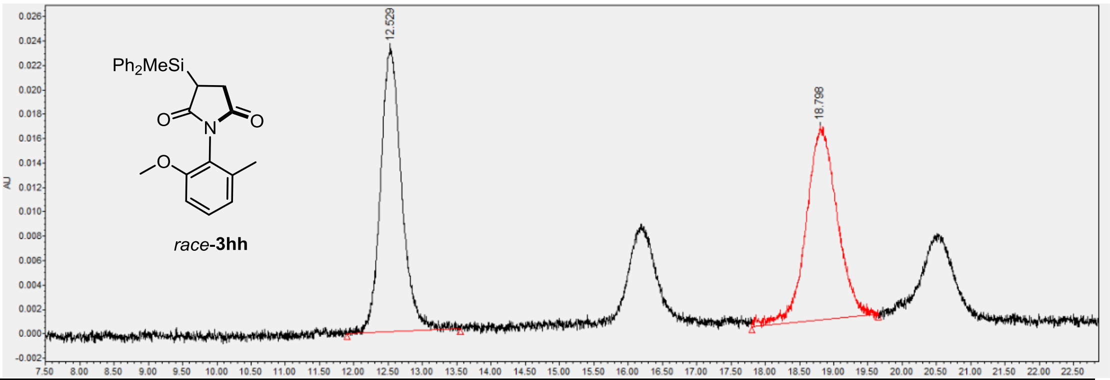

	Time/min	Area	Height	Area%
1	12.529	497223	23302	50.04
2	18.798	496410	15916	49.96

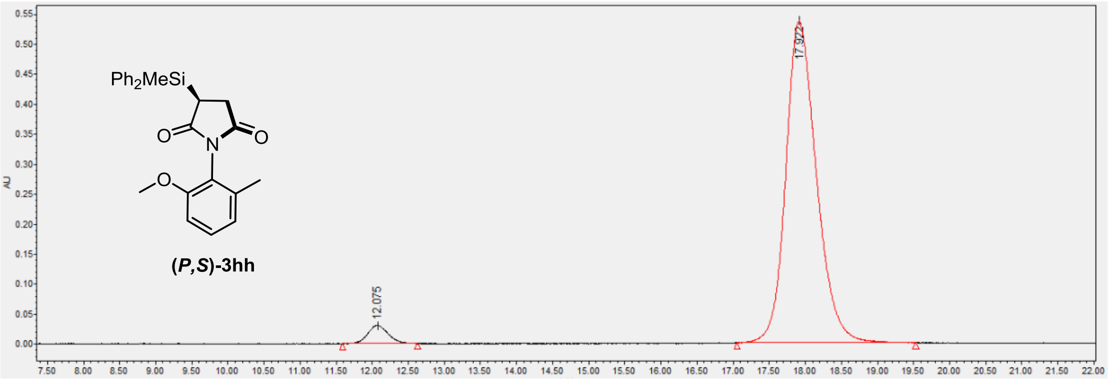

	Time/min	Area	Height	Area%

1	12.075	582824	30190	3.55
2	17.922	15828883	536533	96.45

Supplementary Figure 254. HPLC spectra for **3hh**

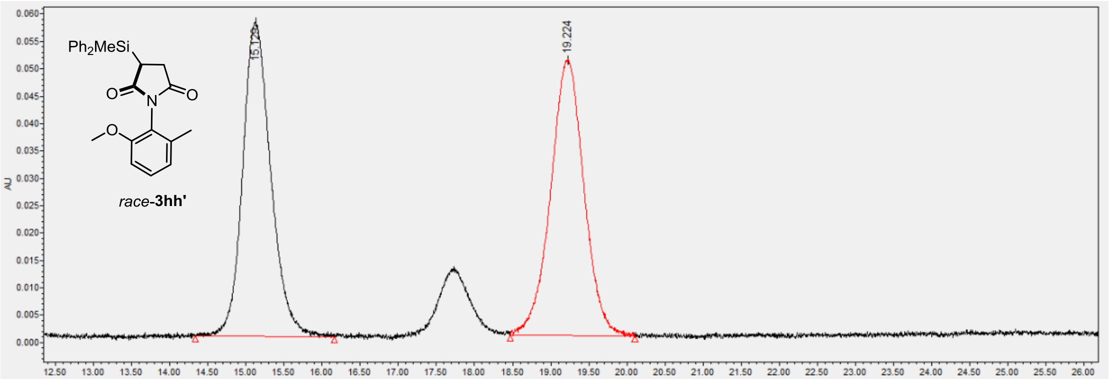

	Time/min	Area	Height	Area%
1	15.129	1477456	57307	49.01
2	19.224	1537074	50360	50.99

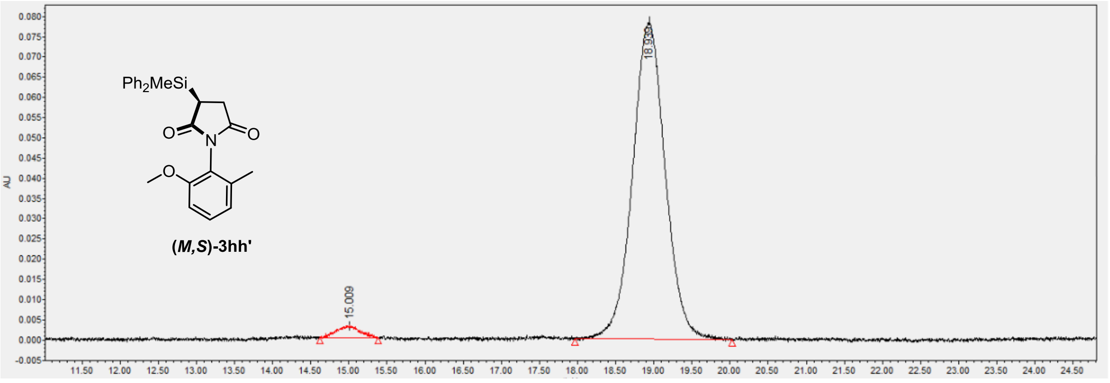

	Time/min	Area	Height	Area%
1	15.009	62004	3037	2.58
2	18.939	2342976	78682	97.42

Supplementary Figure 255. HPLC spectra for **3hh'**

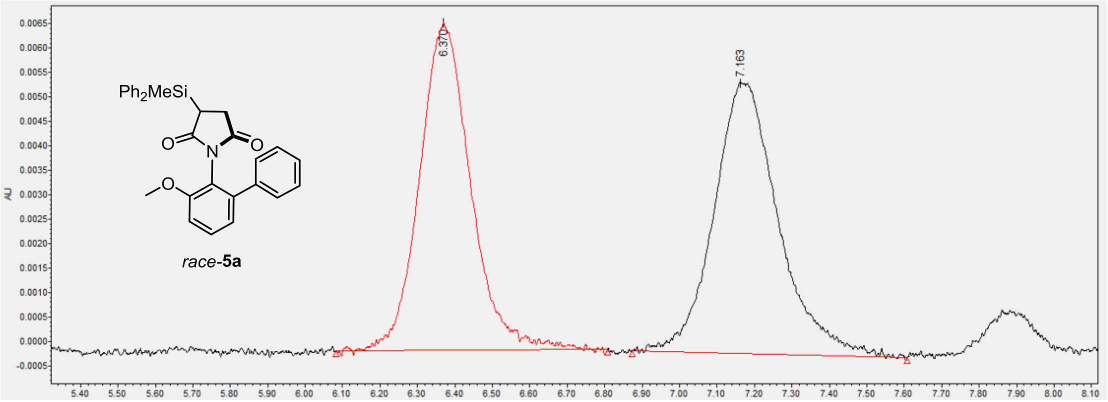

	Time/min	Area	Height	Area%
1	6.370	63553	6694	48.89
2	7.163	66444	5544	51.11

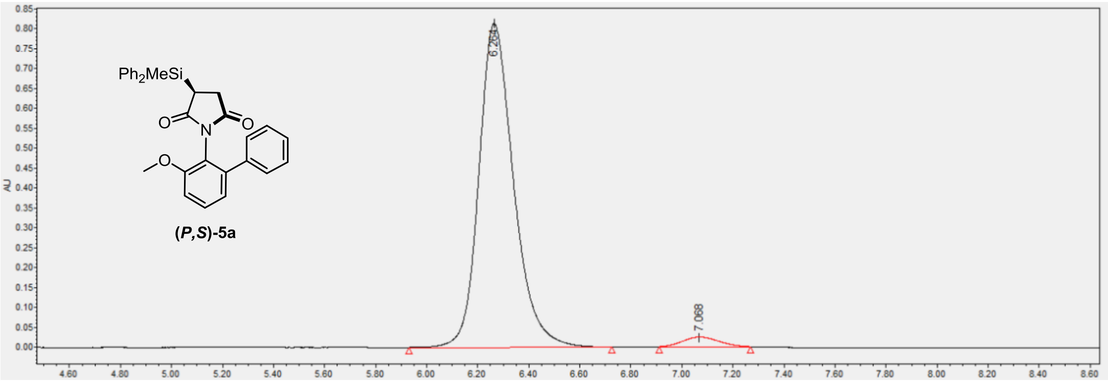

	Time/min	Area	Height	Area%
1	6.264	7689131	812789	97.01
2	7.068	237365	24242	2.99

Supplementary Figure 256. HPLC spectra for **5a**

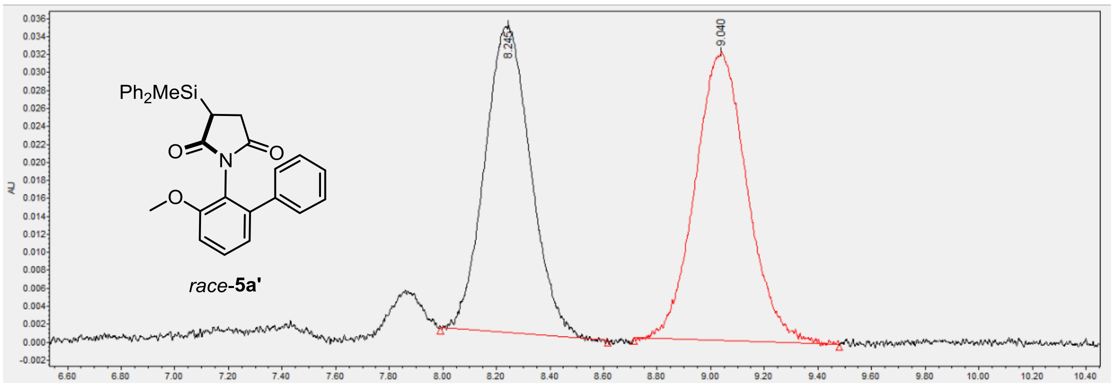

	Time/min	Area	Height	Area%
1	8.245	408388	34229	48.39
2	9.040	435517	32158	51.61

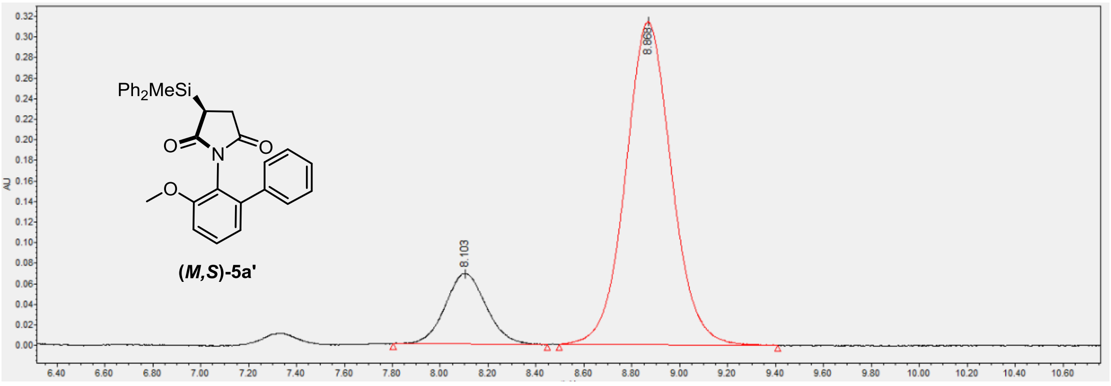

	Time/min	Area	Height	Area%
1	8.103	810447	68388	16.19
2	8.868	4196702	314246	83.81

Supplementary Figure 257. HPLC spectra for **5a'**

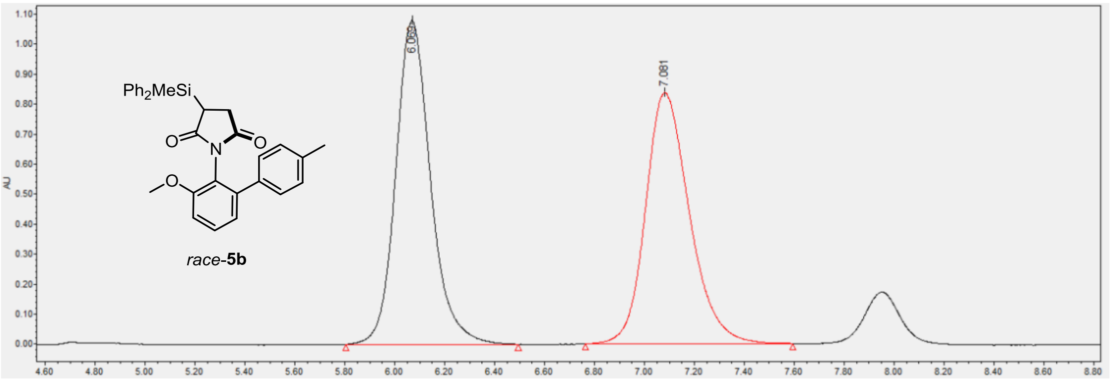

	Time/min	Area	Height	Area%
1	6.069	10430949	1079773	50.55
2	7.081	10204579	838638	49.45

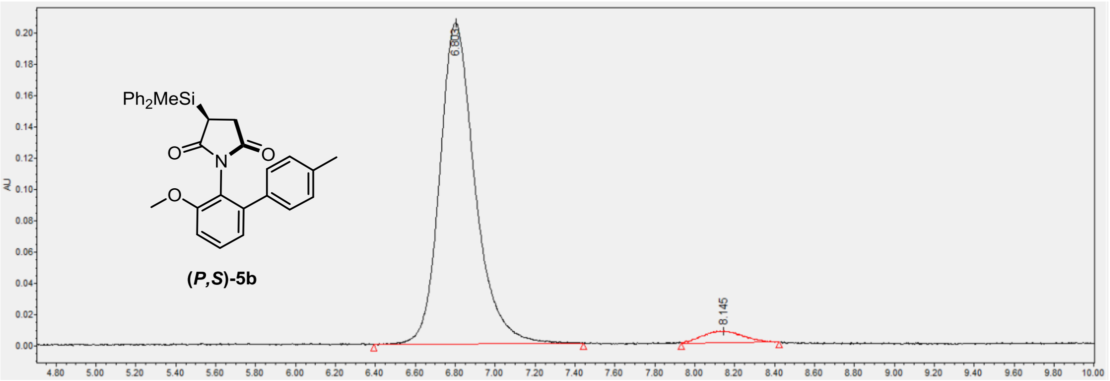

	Time/min	Area	Height	Area%
1	6.803	2527625	205396	96.04

2	8.145	104183	7569	3.96

Supplementary Figure 258. HPLC spectra for **5b**

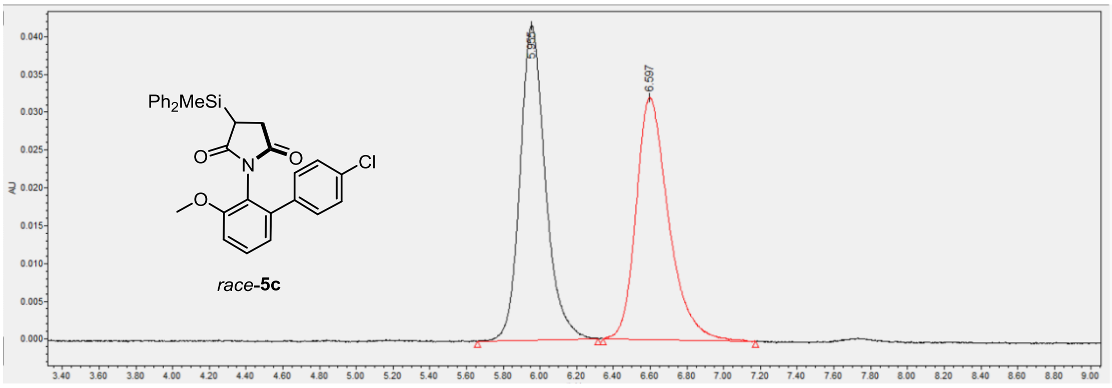

	Time/min	Area	Height	Area%
1	5.955	397979	41513	50.21
2	6.597	394606	32044	49.79

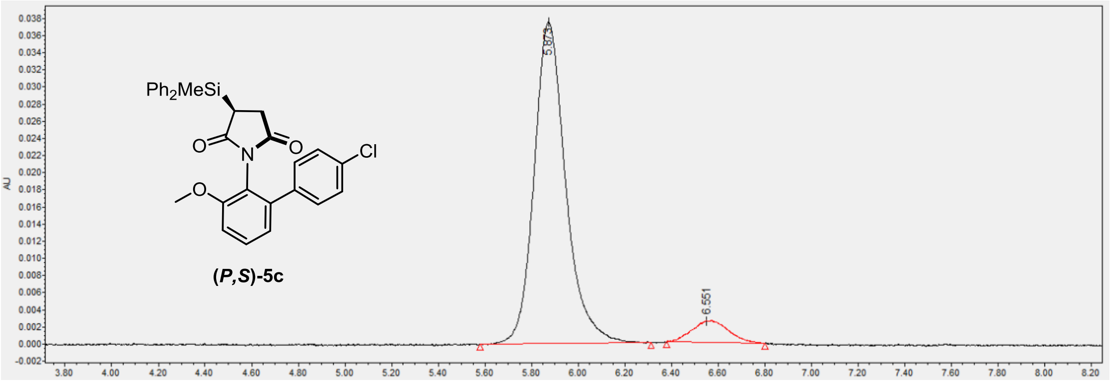

	Time/min	Area	Height	Area%
1	5.873	352859	37499	92.72
2	6.551	27721	2556	7.28

Supplementary Figure 259. HPLC spectra for **5c**

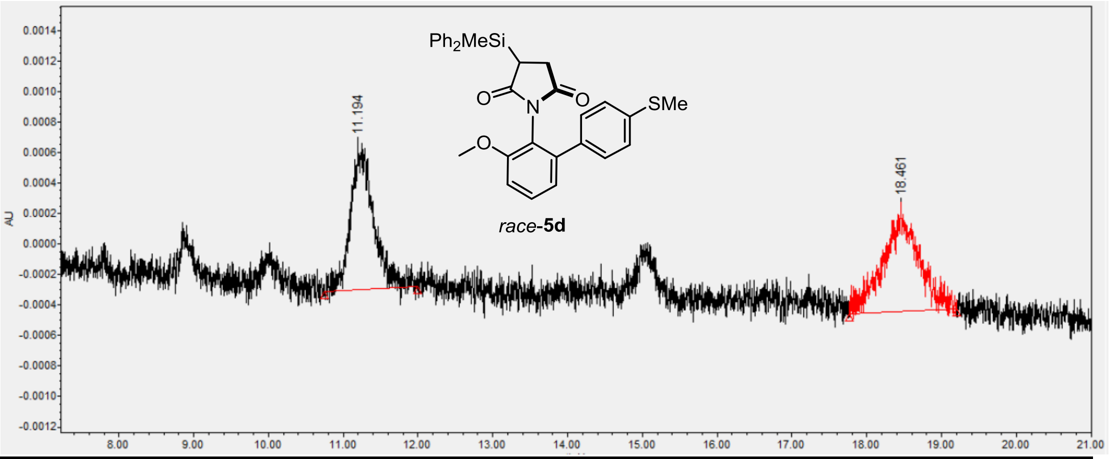

	Time/min	Area	Height	Area%
1	11.194	20406	975	48.43
2	18.461	21732	713	51.57

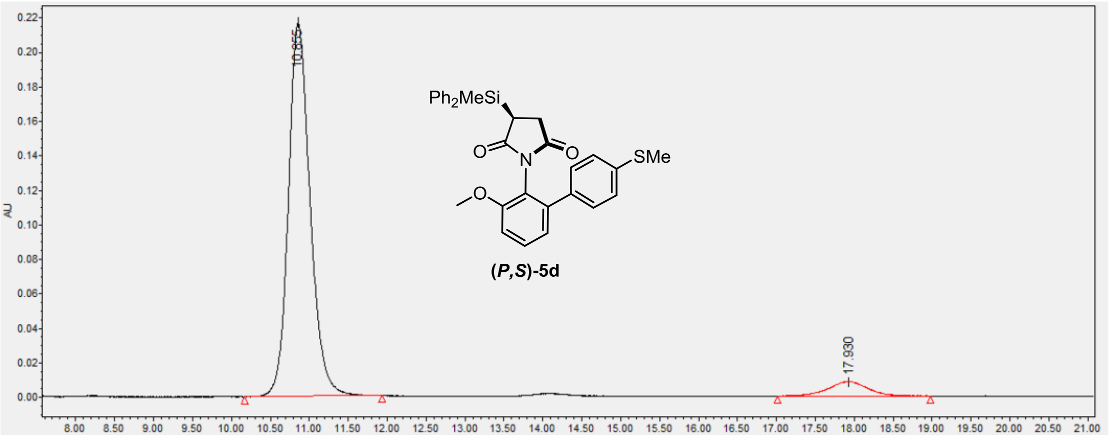

	Time/min	Area	Height	Area%
1	10.855	4203115	216572	93.35
2	17.930	299306	8425	6.65

Supplementary Figure 260. HPLC spectra for **5d**

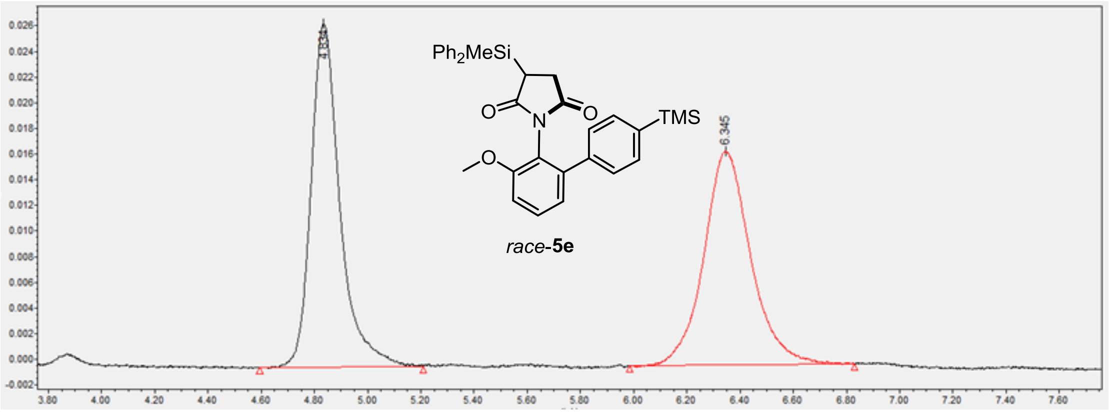

Time/min	Area	Height	Area%

1	4.834	201232	26752	49.79
2	6.345	202950	16637	50.21

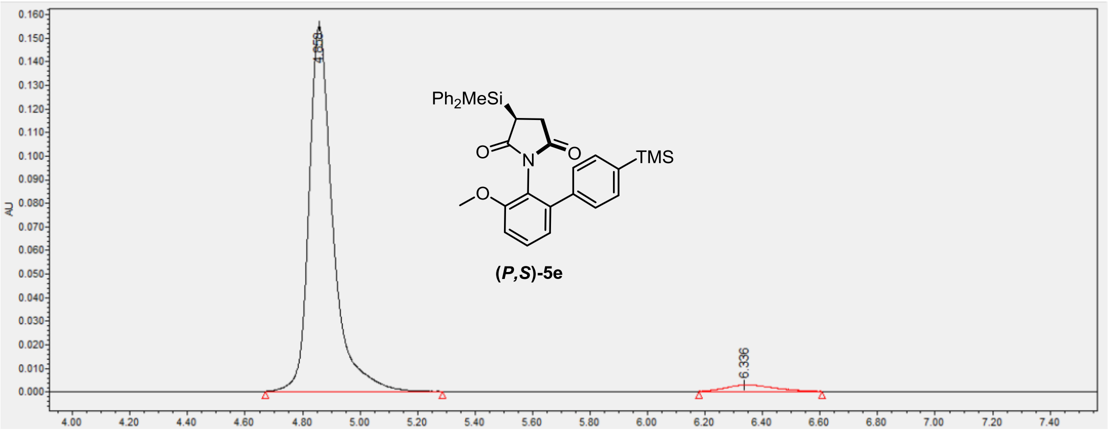

	Time/min	Area	Height	Area%
1	4.858	896076	154694	96.63
2	6.336	31212	2726	3.37

Supplementary Figure 261. HPLC spectra for **5e**

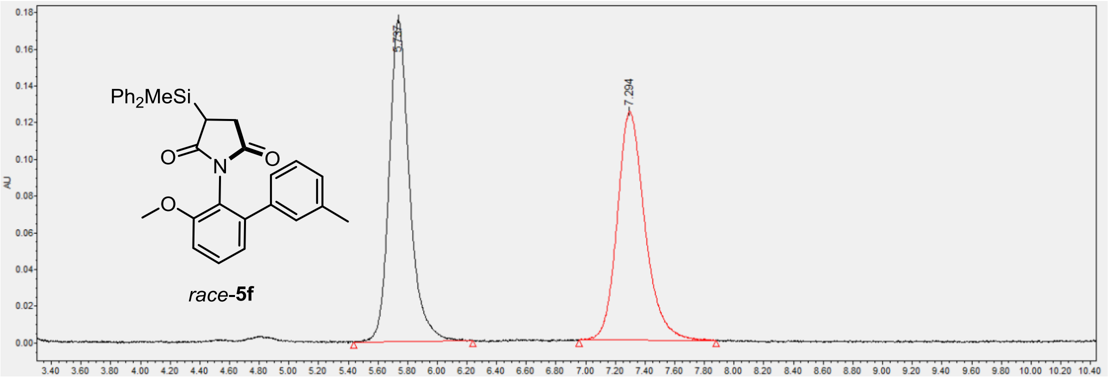

	Time/min	Area	Height	Area%
1	5.737	1669648	175639	50.76
2	7.294	1619910	124860	49.24

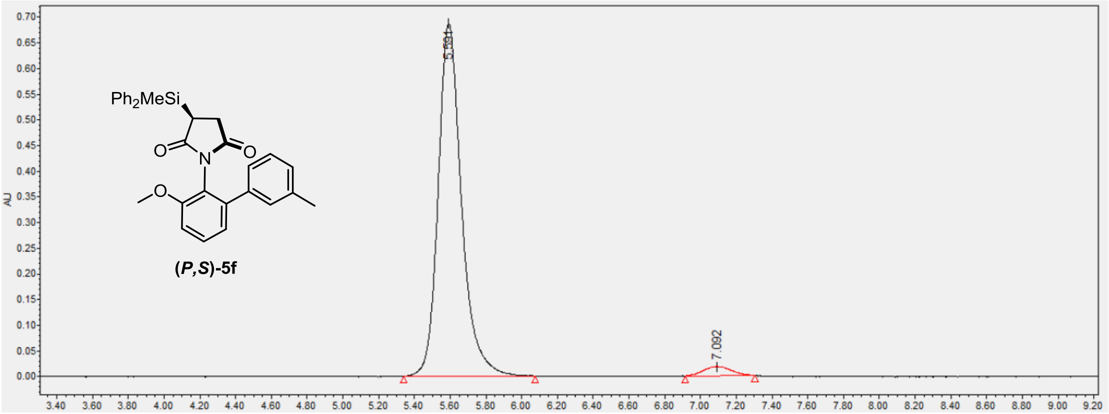

	Time/min	Area	Height	Area%
1	5.591	6218715	686866	96.91
2	7.092	198416	18367	3.09

Supplementary Figure 262. HPLC spectra for **5f**

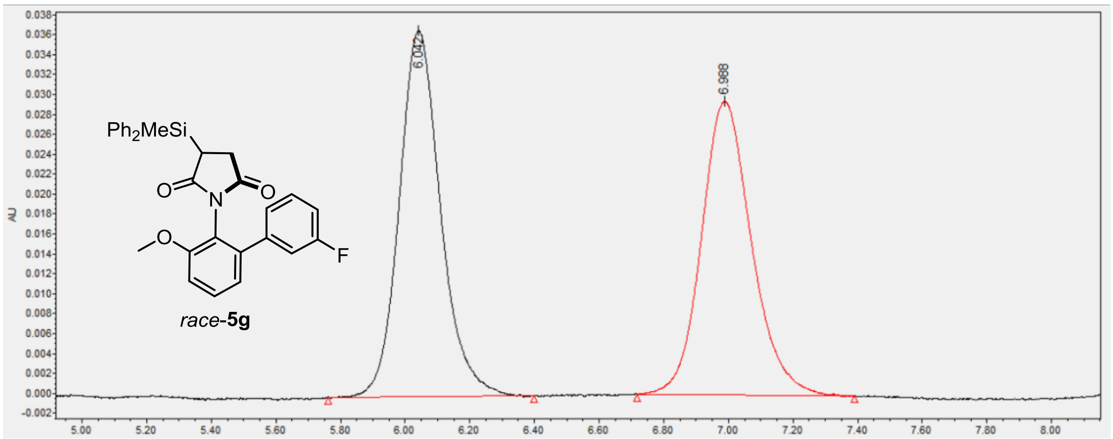

	Time/min	Area	Height	Area%
1	6.042	326130	36704	50.72
2	6.988	316865	29474	49.28

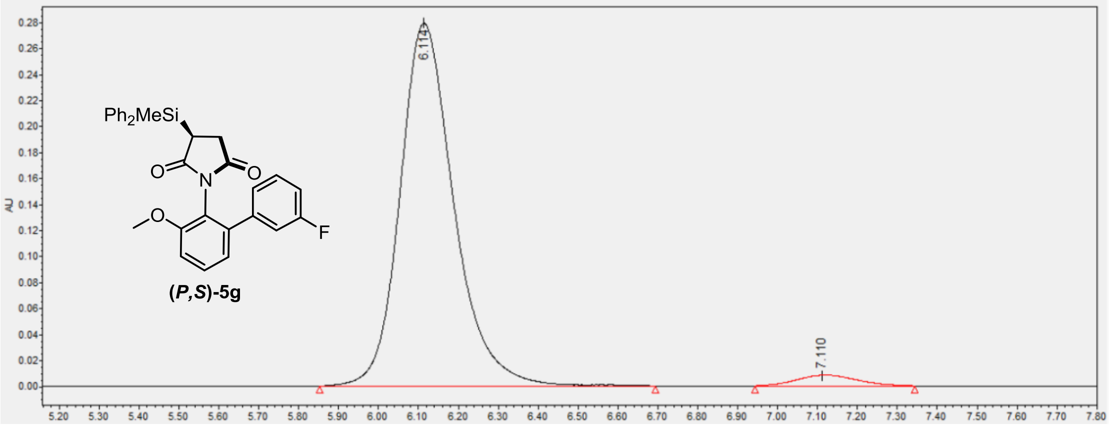

	Time/min	Area	Height	Area%

1	6.114	2616748	279092	96.81
2	7.110	86088	8270	3.19

Supplementary Figure 263. HPLC spectra for **5g**

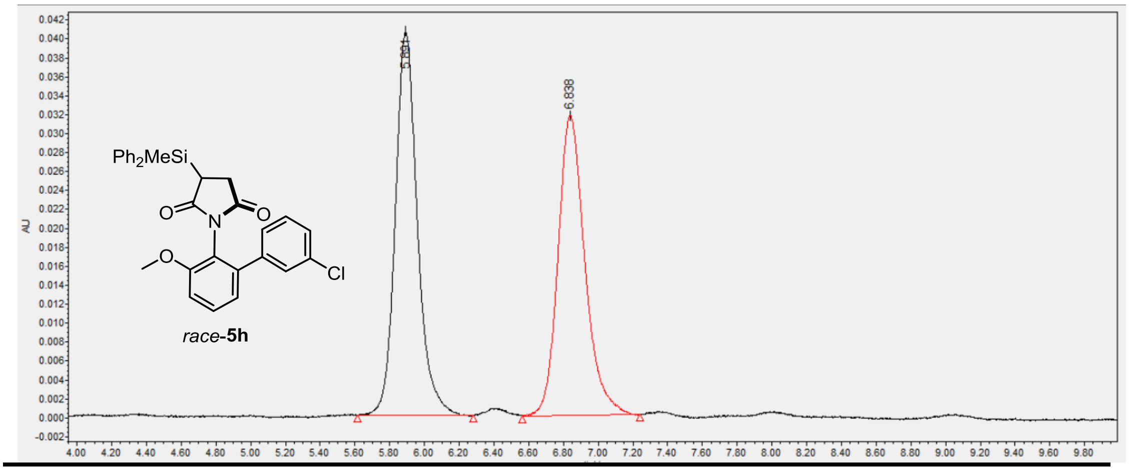

	Time/min	Area	Height	Area%
1	5.891	356255	40477	51.13
2	6.838	340463	31643	48.87

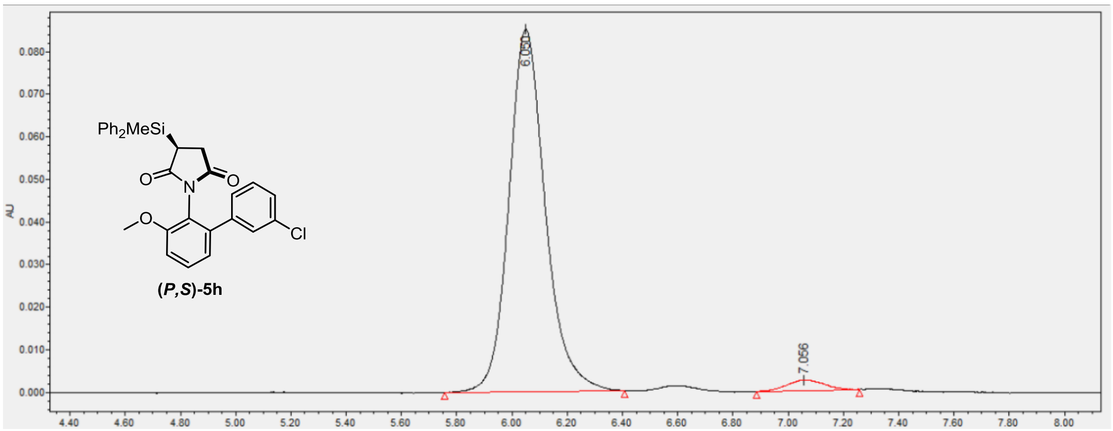

	Time/min	Area	Height	Area%
1	6.050	778003	85012	97.23
2	7.056	22156	2423	2.77

Supplementary Figure 264. HPLC spectra for **5h**

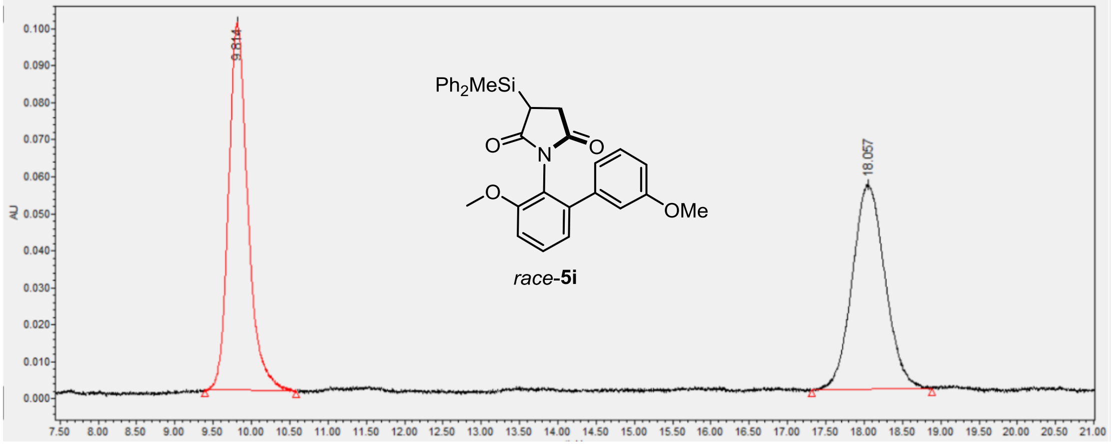

	Time/min	Area	Height	Area%
1	9.814	1732218	99270	51.35
2	18.057	1640832	55391	48.65

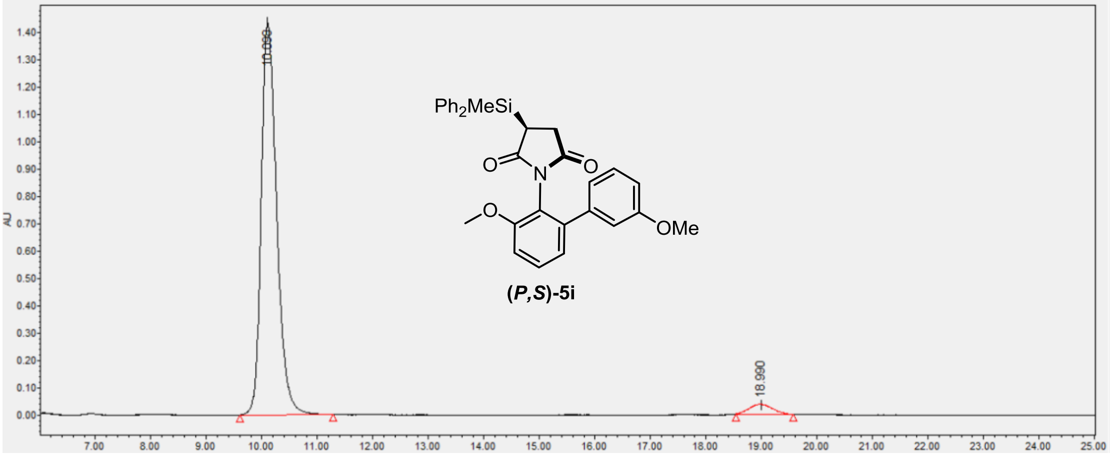

	Time/min	Area	Height	Area%
1	10.098	27704482	1432514	96.37
2	18.990	1043999	36359	3.63

Supplementary Figure 265. HPLC spectra for **5i**

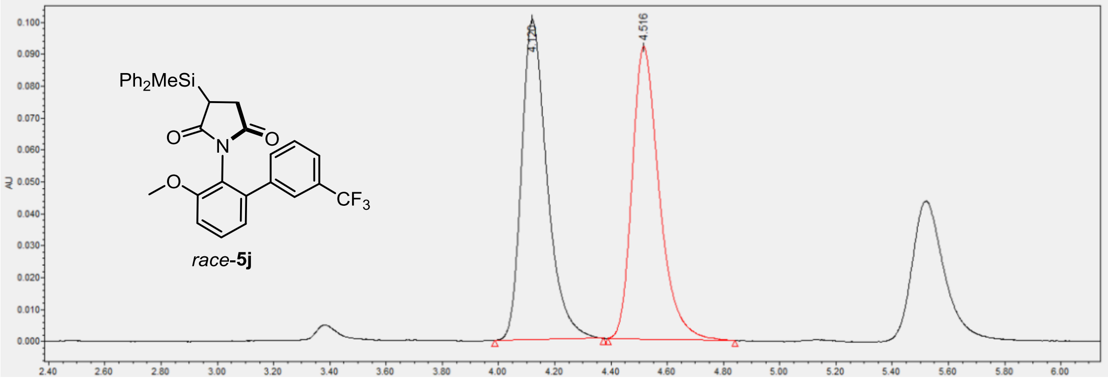

Time/min	Area	Height	Area%

1	4.120	631161	100296	50.70
2	4.516	613729	91620	49.30

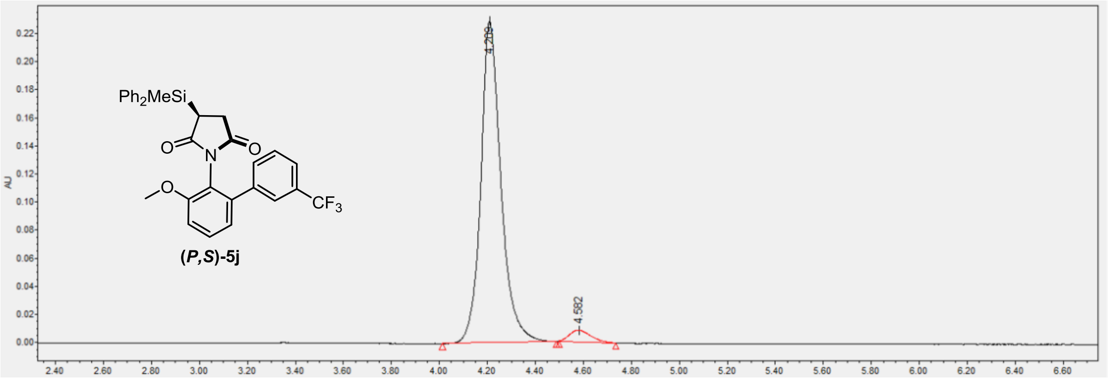

	Time/min	Area	Height	Area%
1	4.209	1333461	229090	96.39
2	4.582	49895	8193	3.61

Supplementary Figure 266. HPLC spectra for **5j**

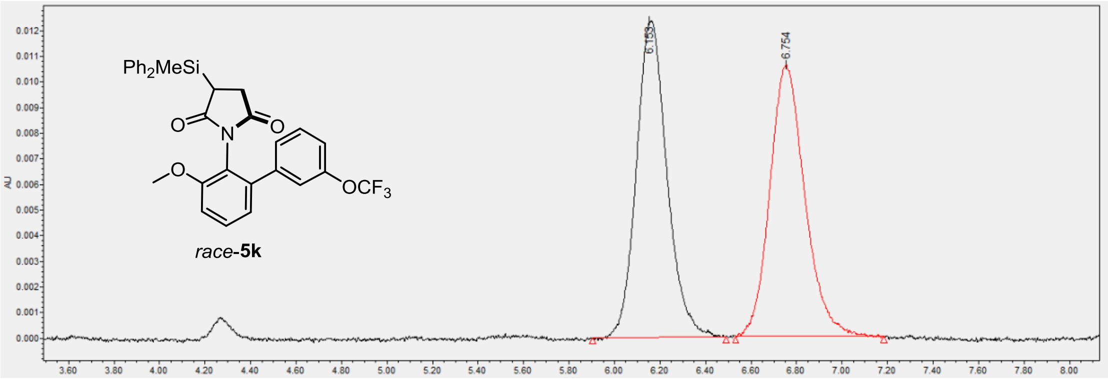

	Time/min	Area	Height	Area%
1	6.153	115224	12335	50.35
2	6.754	113635	10606	49.65

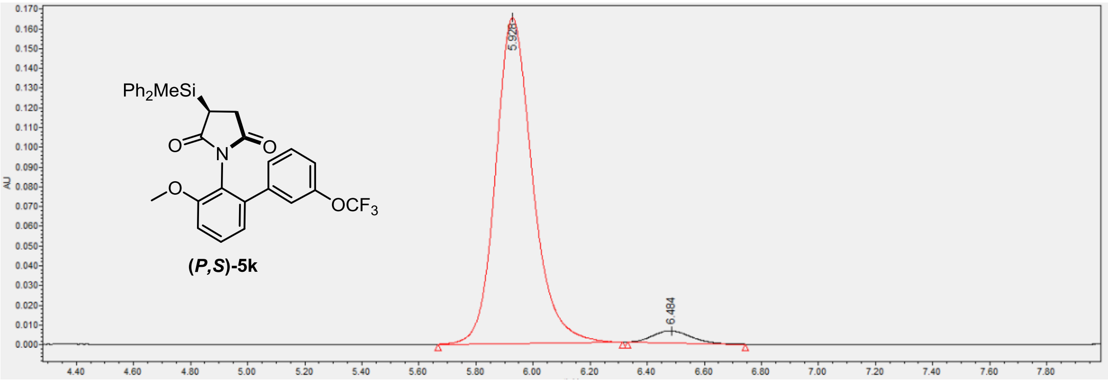

	Time/min	Area	Height	Area%
1	5.928	1482378	165030	96.29
2	6.484	57119	6175	3.71

Supplementary Figure 267. HPLC spectra for **5k**

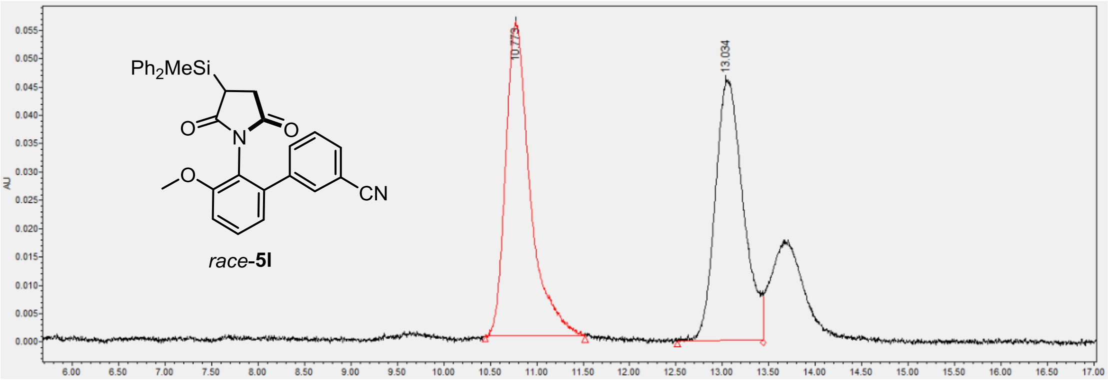

	Time/min	Area	Height	Area%
1	10.773	1017170	55531	51.48
2	13.034	958835	46122	48.52

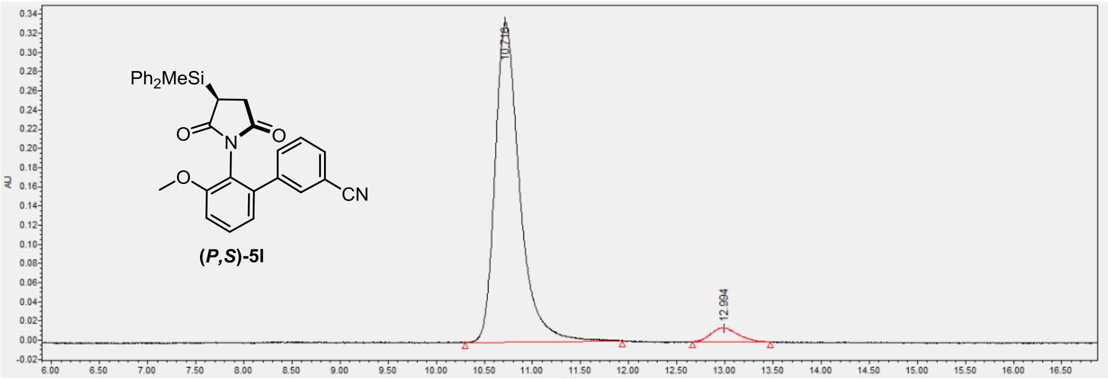

	Time/min	Area	Height	Area%
1	10.716	5931474	334161	95.52
2	12.994	278357	14463	4.48

Supplementary Figure 268. HPLC spectra for **5l**

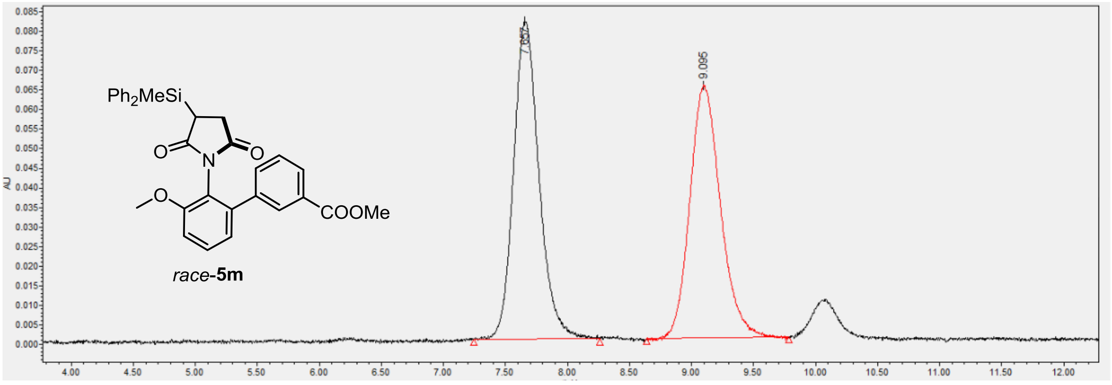

	Time/min	Area	Height	Area%
1	7.657	1160529	81340	51.07
2	9.095	1112077	64705	48.93

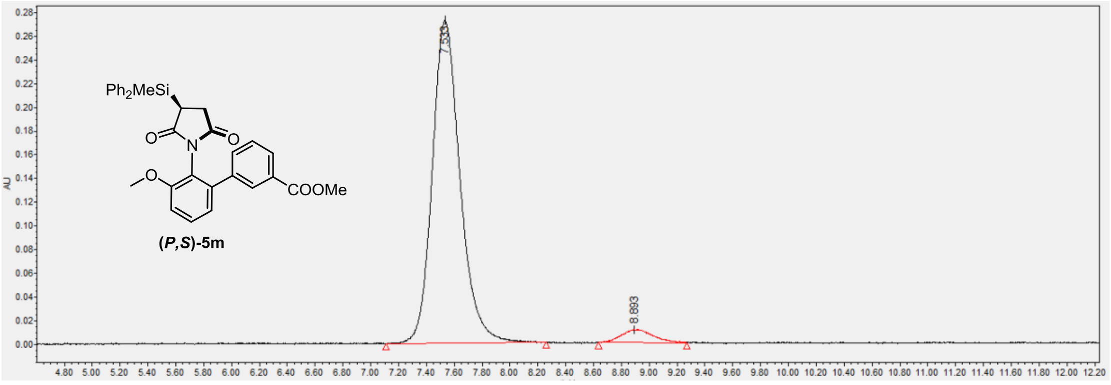

	Time/min	Area	Height	Area%
1	7.533	3760856	271786	95.69
2	8.893	169565	11018	4.31

Supplementary Figure 269. HPLC spectra for **5m**

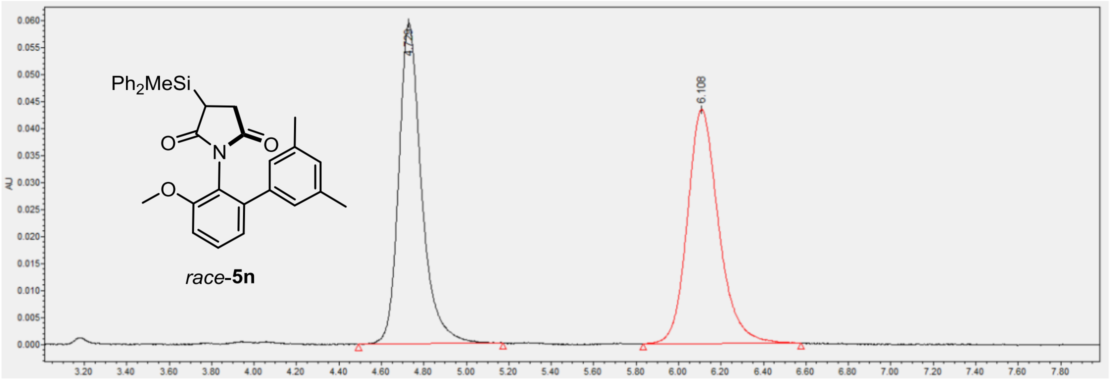

	Time/min	Area	Height	Area%
1	4.729	445122	59343	49.98
2	6.108	445464	43396	50.02

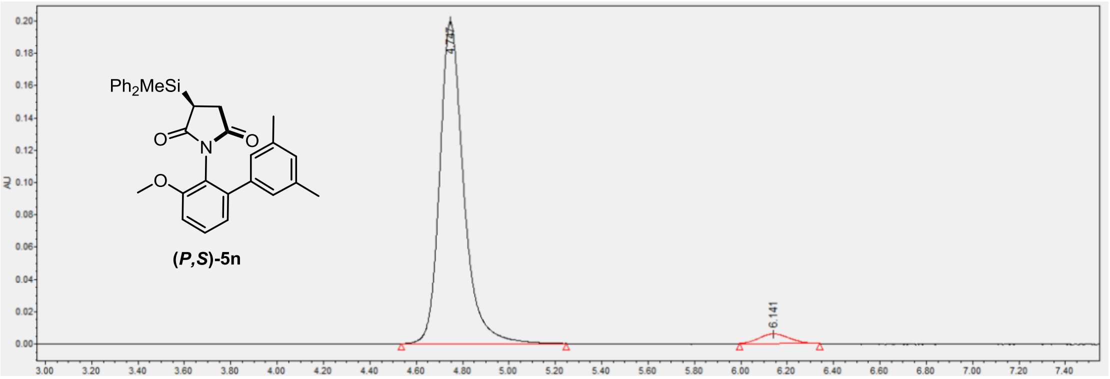

	Time/min	Area	Height	Area%
1	4.747	1439034	199753	96.40
2	6.141	53751	5967	3.60

Supplementary Figure 270. HPLC spectra for **5n**

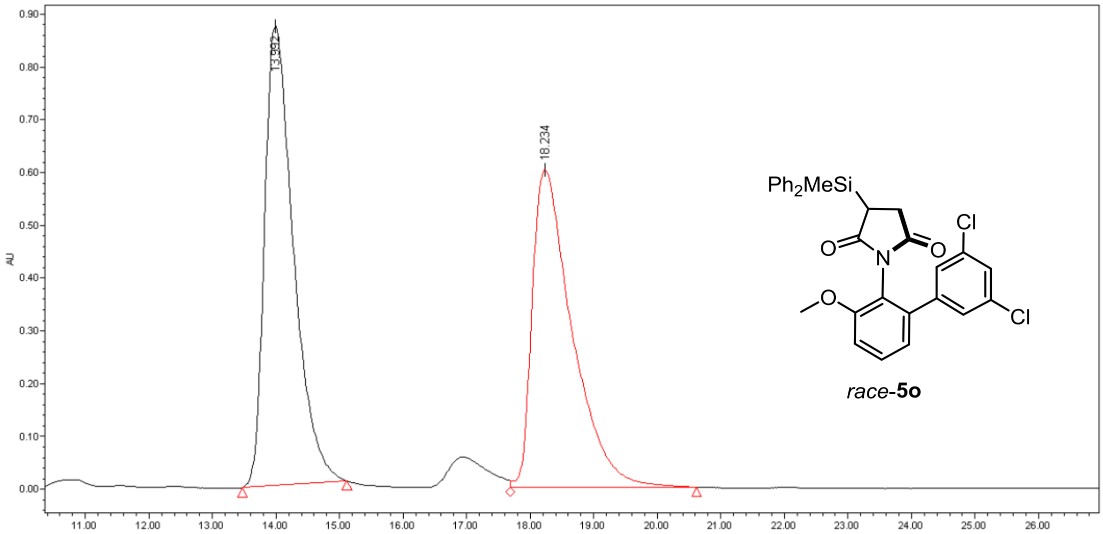

	Time/min	Area	Height	Area%
1	13.992	27523840	870093	51.24
2	18.234	26188128	601538	48.76

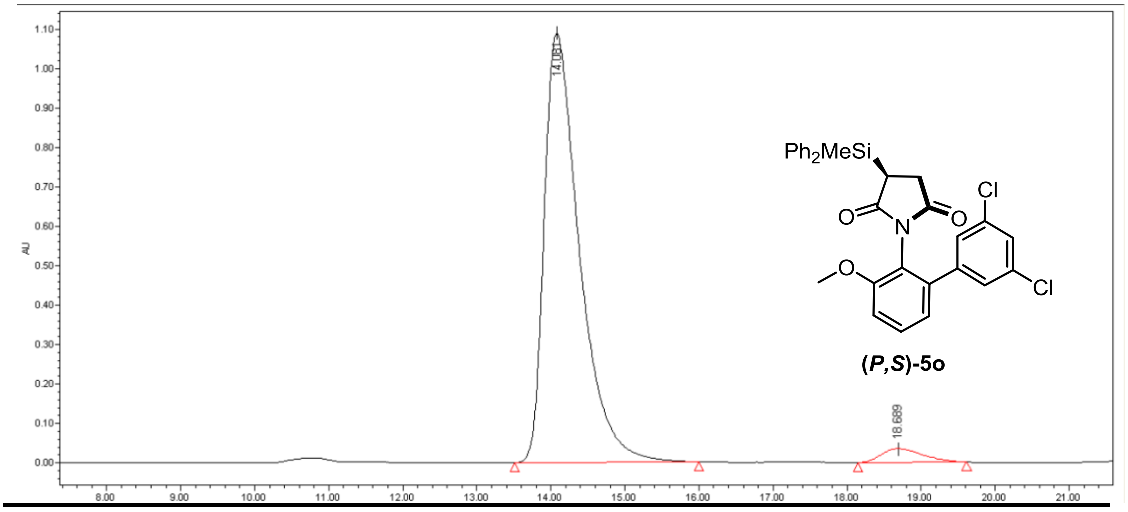

	Time/min	Area	Height	Area%
1	14.081	35948490	1089113	96.43
2	18.689	1330913	34691	3.57

Supplementary Figure 271. HPLC spectra for **5o**

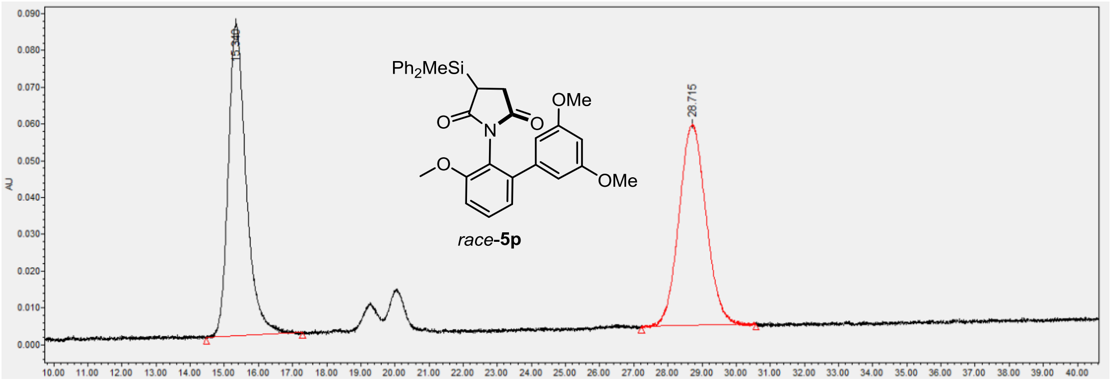

	Time/min	Area	Height	Area%
1	15.340	3089539	85072	50.73
2	28.715	3000488	54851	49.27

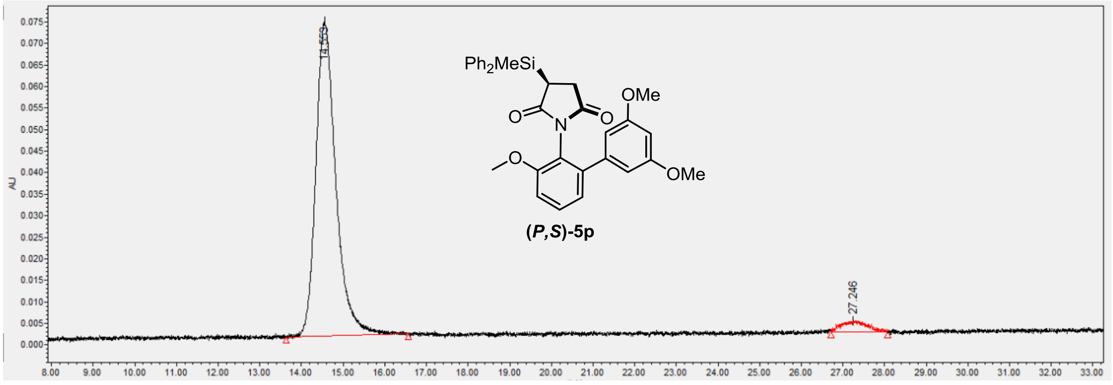

	Time/min	Area	Height	Area%

1	14.553	2432717	73096	95.96
2	27.246	102478	2580	4.04

Supplementary Figure 272. HPLC spectra for **5p**

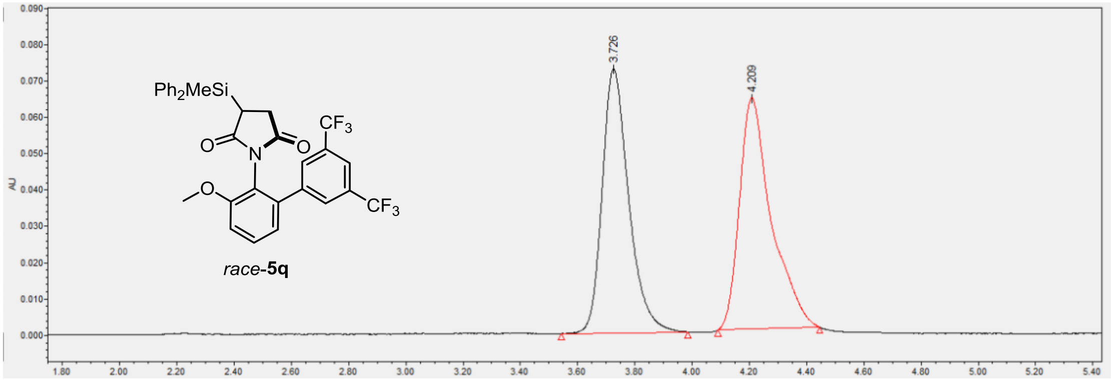

	Time/min	Area	Height	Area%
1	3.726	468930	72675	48.77
2	4.209	492592	63531	51.23

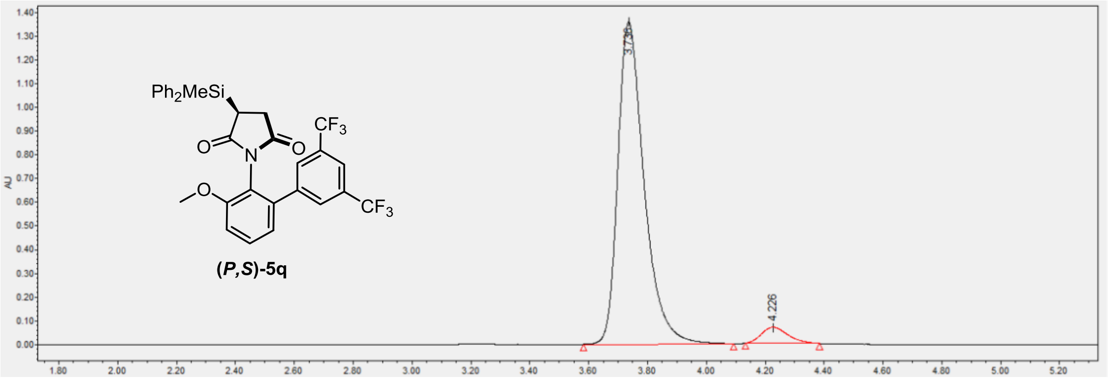

	Time/min	Area	Height	Area%
1	3.736	8428852	1359293	95.21
2	4.226	423663	67790	4.79

Supplementary Figure 273. HPLC spectra for **5q**

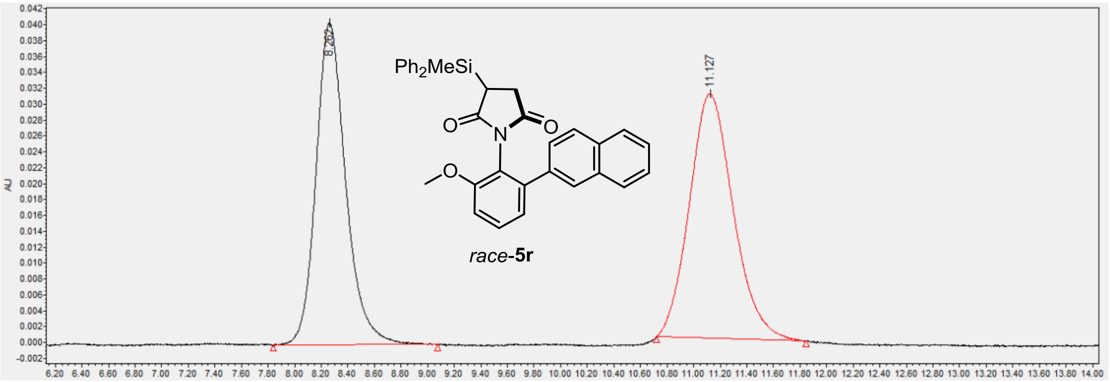

	Time/min	Area	Height	Area%
1	8.262	630045	40471	47.17
2	11.127	705597	30846	52.83

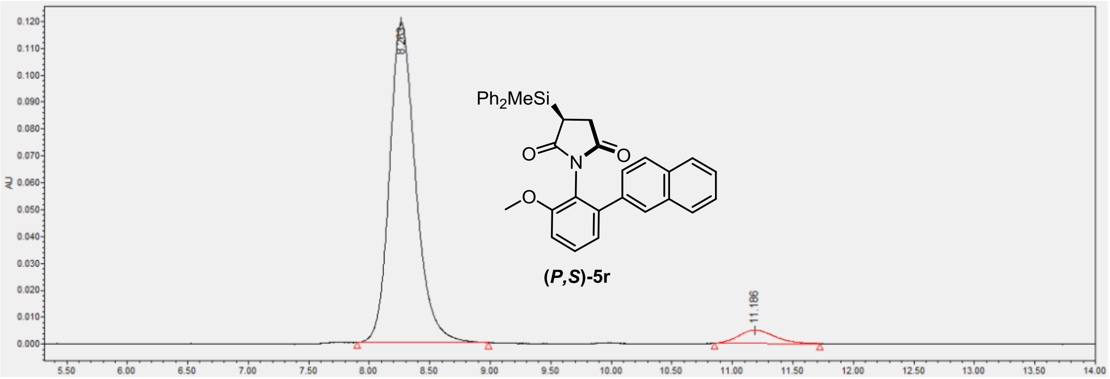

	Time/min	Area	Height	Area%
1	8.263	1788731	119272	94.66
2	11.186	100990	4968	5.34

Supplementary Figure 274. HPLC spectra for **5r**

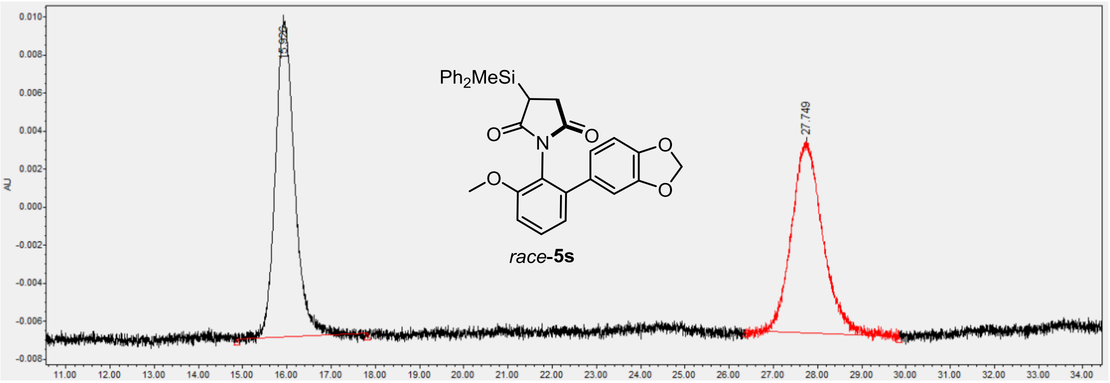

	Time/min	Area	Height	Area%
1	15.926	510850	16684	50.63
2	27.749	498115	10062	49.37

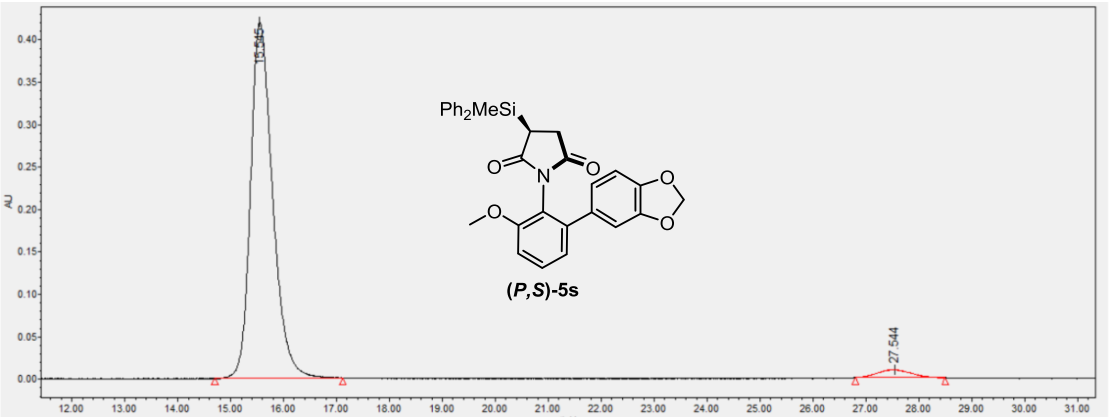

	Time/min	Area	Height	Area%
1	15.545	12122944	419704	96.79
2	27.544	401675	9413	3.21

Supplementary Figure 275. HPLC spectra for **5s**

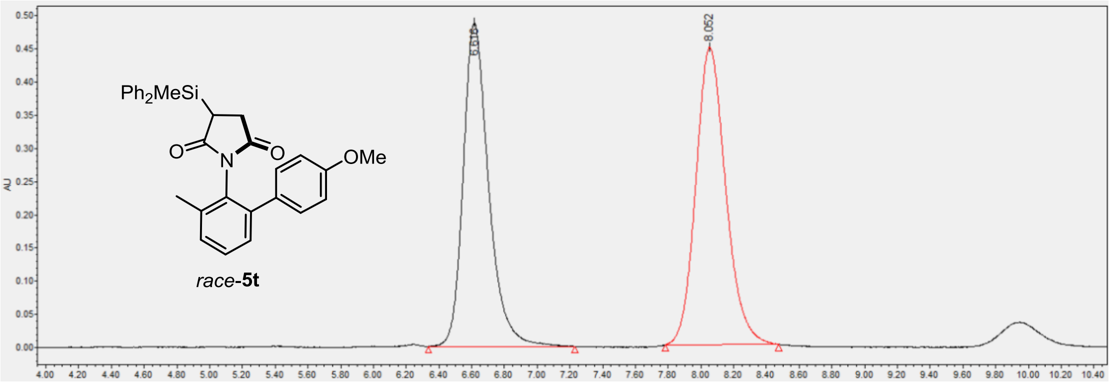

	Time/min	Area	Height	Area%
1	6.616	5256647	487842	48.00
2	8.052	5693922	448267	52.00

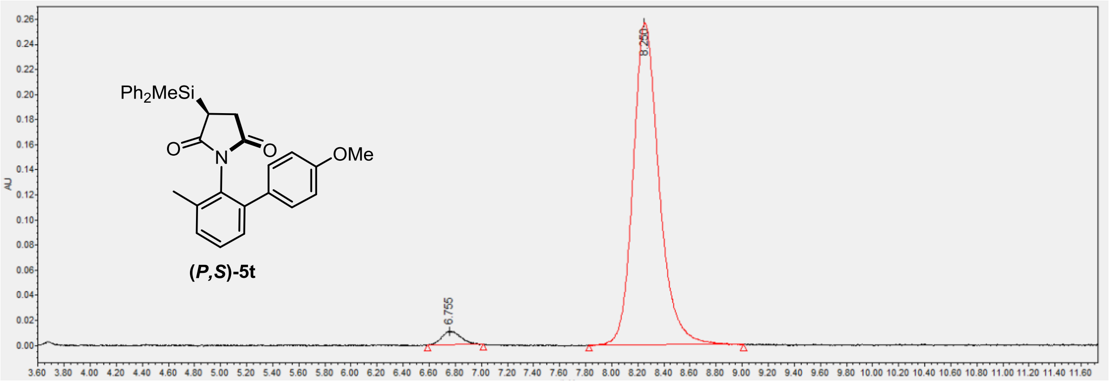

	Time/min	Area	Height	Area%

1	6.755	109356	10866	2.98
2	8.250	3562411	256647	97.02

Supplementary Figure 276. HPLC spectra for **5t**

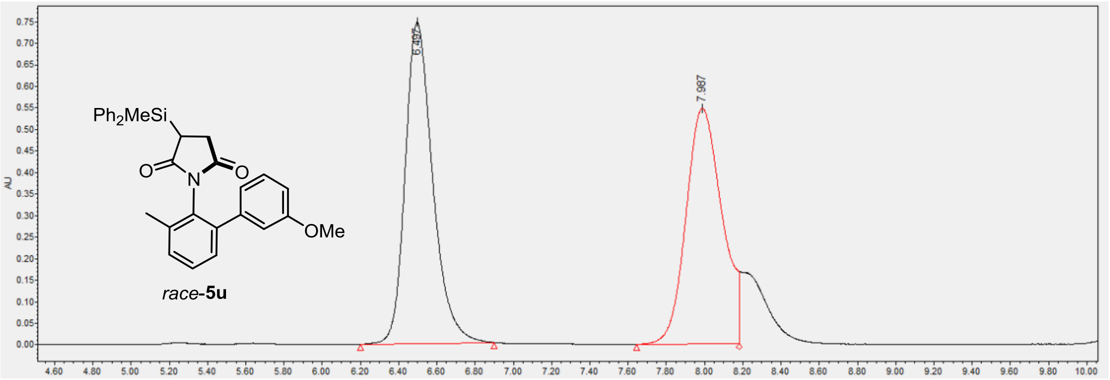

	Time/min	Area	Height	Area%
1	6.497	7456815	745734	51.06
2	7.987	7146542	547934	48.94

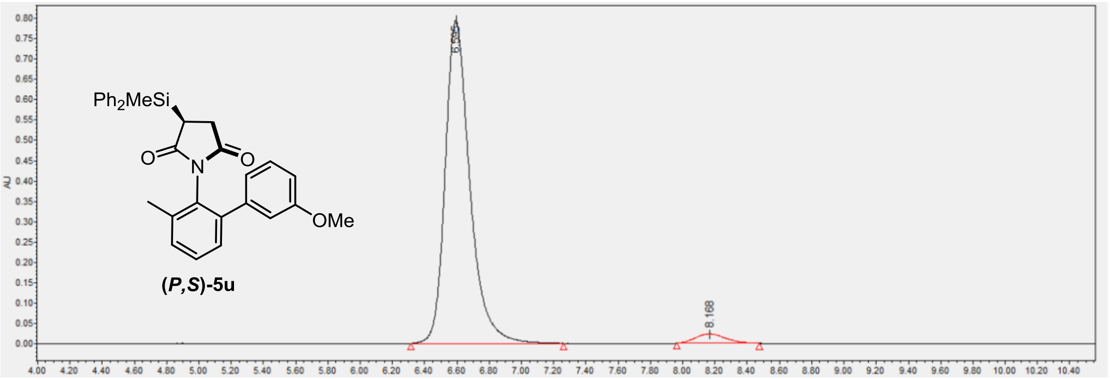

	Time/min	Area	Height	Area%
1	6.595	8428251	793413	96.64
2	8.168	292633	22664	3.36

Supplementary Figure 277. HPLC spectra for **5u**

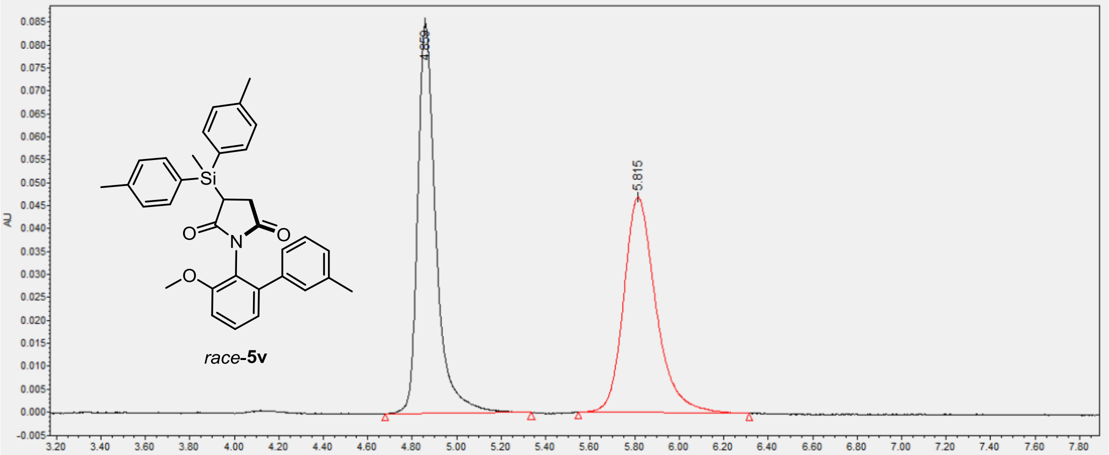

	Time/min	Area	Height	Area%
1	4.859	485936	84965	50.71
2	5.815	472386	46953	49.29

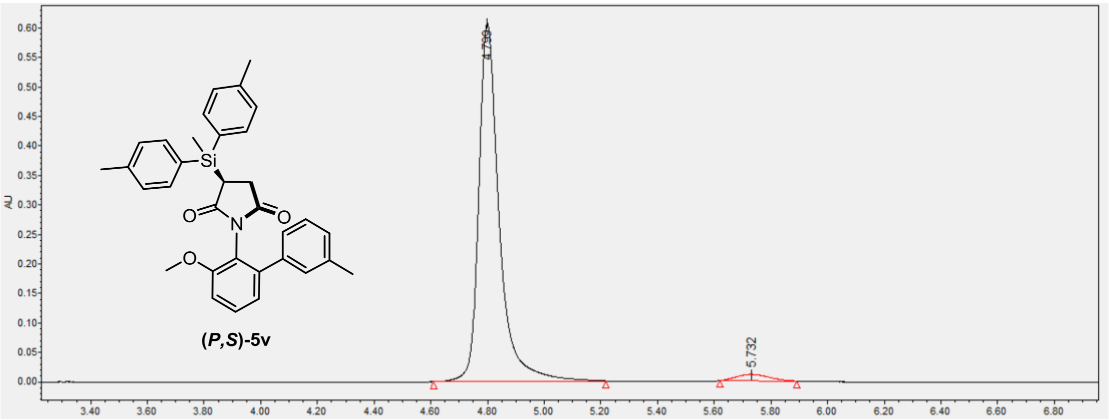

	Time/min	Area	Height	Area%
1	4.799	3060654	606911	97.22
2	5.732	87410	10986	2.78

Supplementary Figure 278. HPLC spectra for **5v**

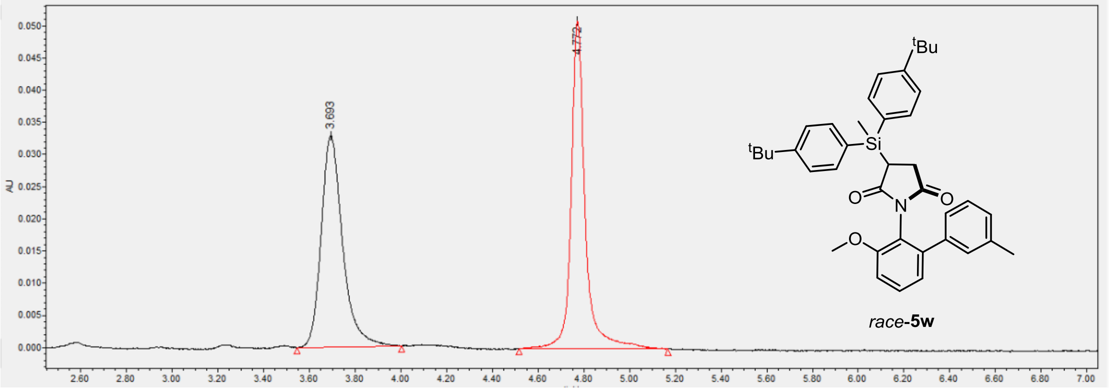

	Time/min	Area	Height	Area%

1	3.693	215030	32919	49.79
2	4.772	216870	50916	50.21

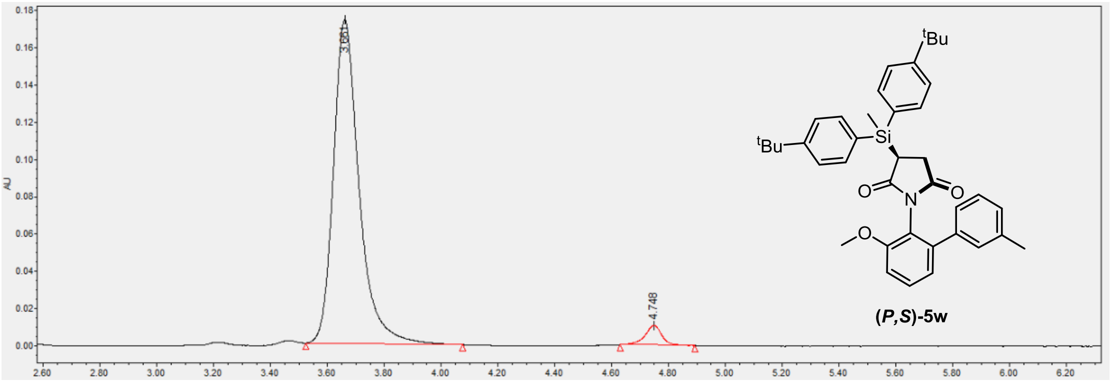

	Time/min	Area	Height	Area%
1	3.661	1091303	173642	96.16
2	4.748	43613	10560	3.84

Supplementary Figure 279. HPLC spectra for **5w**

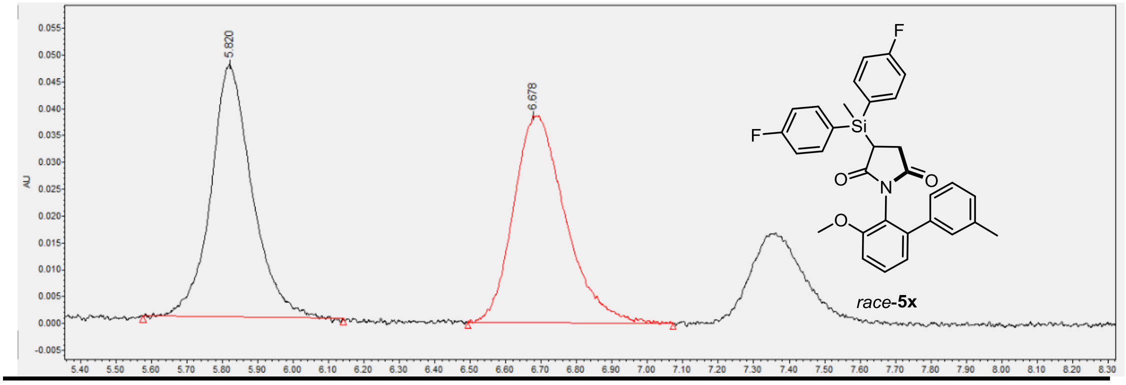

	Time/min	Area	Height	Area%
1	5.820	383410	47213	49.37
2	6.678	393149	38605	50.63

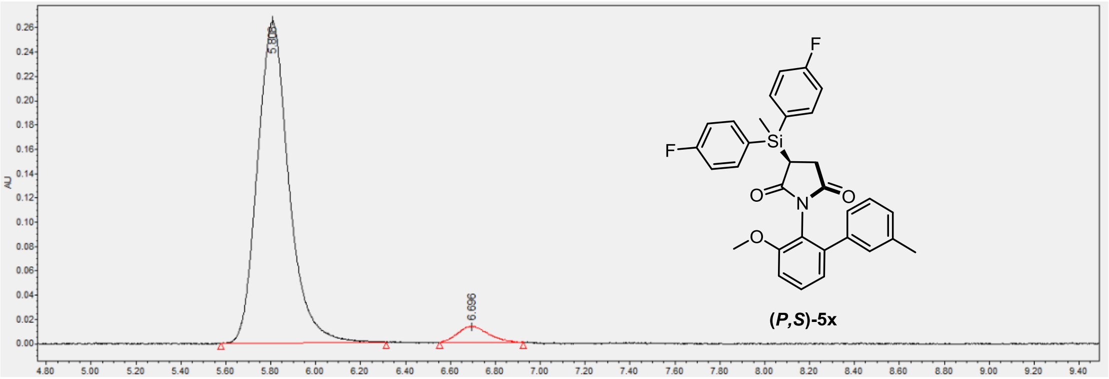

	Time/min	Area	Height	Area%

1	5.808	2575040	265148	95.47
2	6.696	122163	12918	4.53

Supplementary Figure 280. HPLC spectra for **5x**

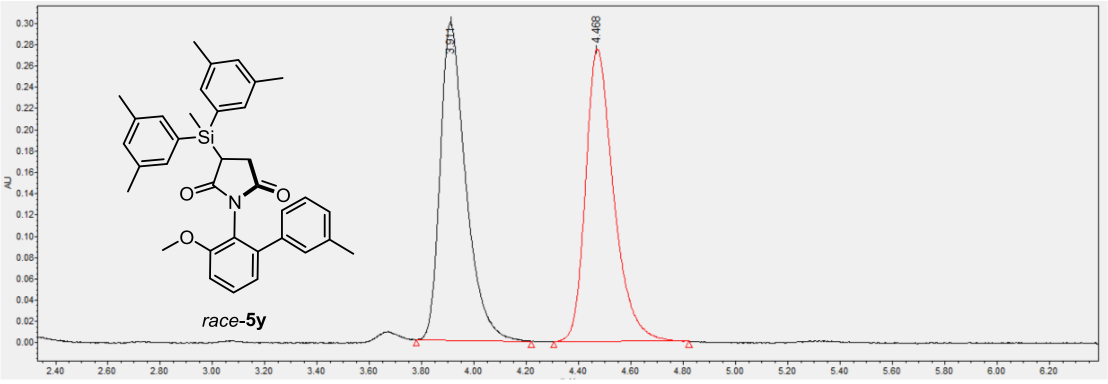

	Time/min	Area	Height	Area%
1	3.911	2023103	299636	49.74
2	4.468	2044468	274723	50.26

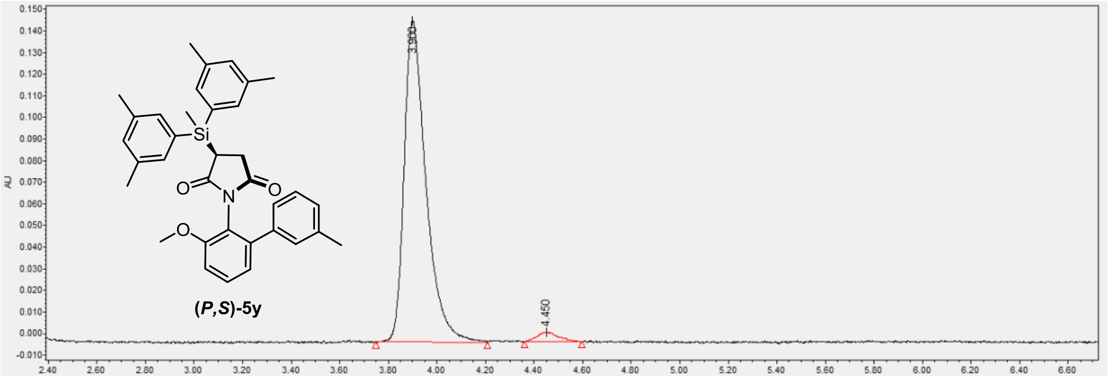

	Time/min	Area	Height	Area%
1	3.900	927818	148507	97.26
2	4.450	26158	4351	2.74

Supplementary Figure 281. HPLC spectra for **5y**

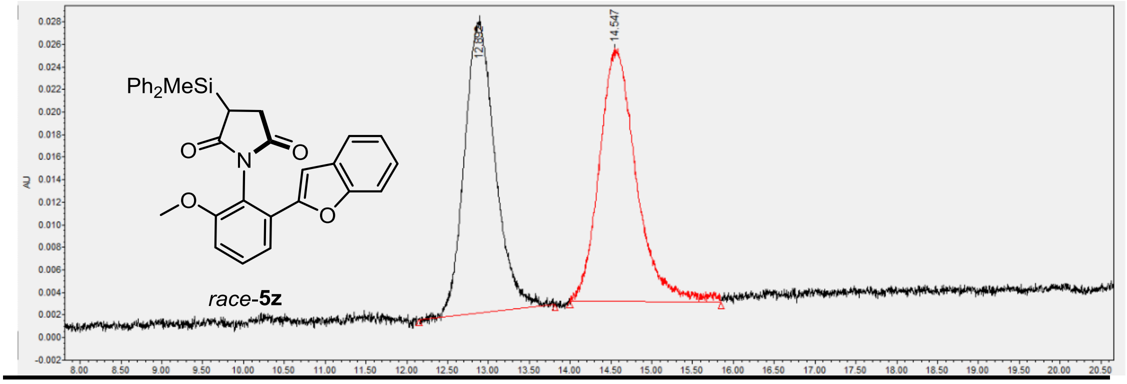

	Time/min	Area	Height	Area%
1	12.892	678983	25969	48.85
2	14.547	710937	22522	51.15

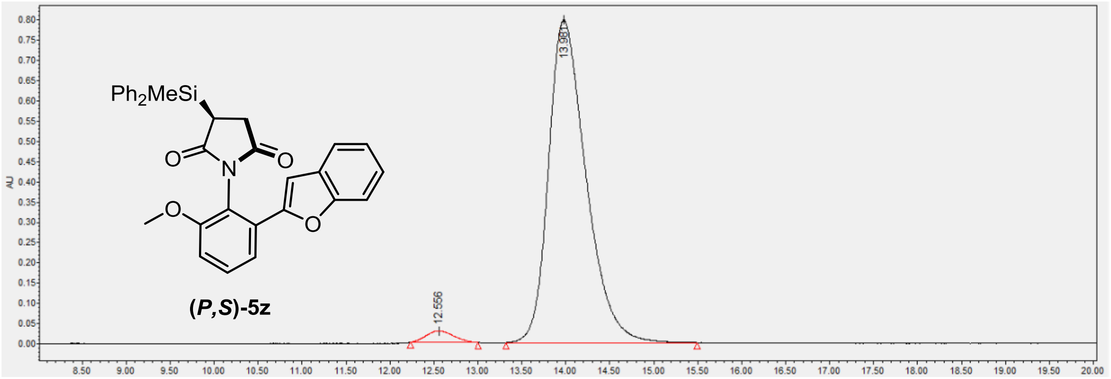

	Time/min	Area	Height	Area%
1	12.556	621540	28563	2.56
2	13.981	23621263	796156	97.44

Supplementary Figure 282. HPLC spectra for **5z**

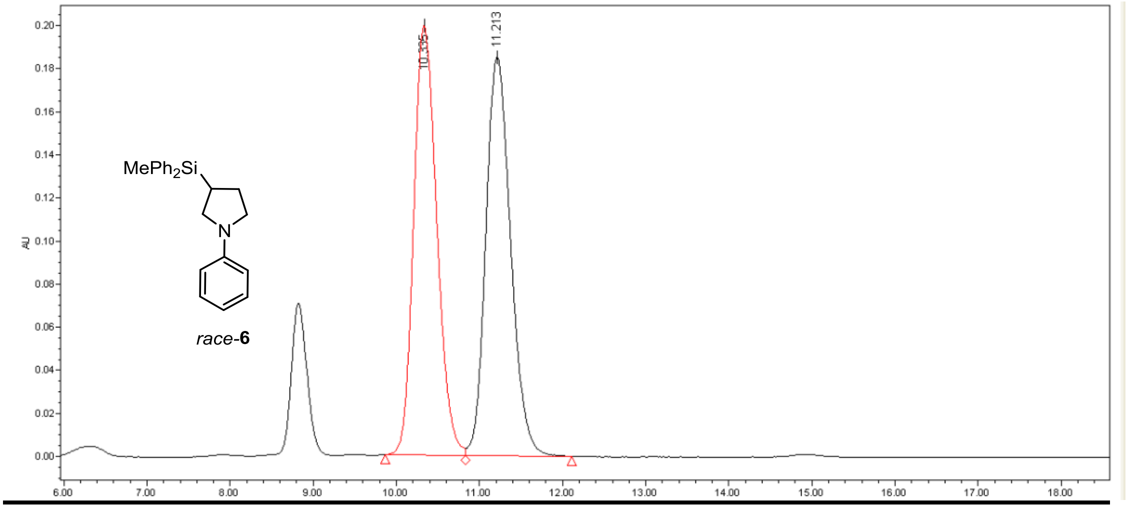

	Time/min	Area	Height	Area%
1	10.335	3828924	199300	50.03

2	11.213	3823952	185182	49.97

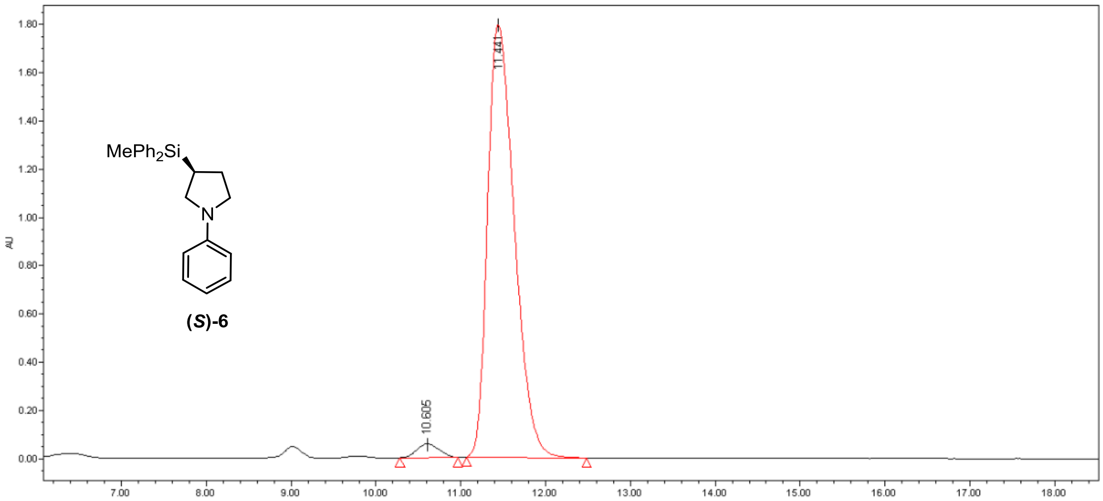

	Time/min	Area	Height	Area%
1	10.605	1037297	57149	2.57
2	11.441	39299781	39299781	97.43

Supplementary Figure 283. HPLC spectra for **6**

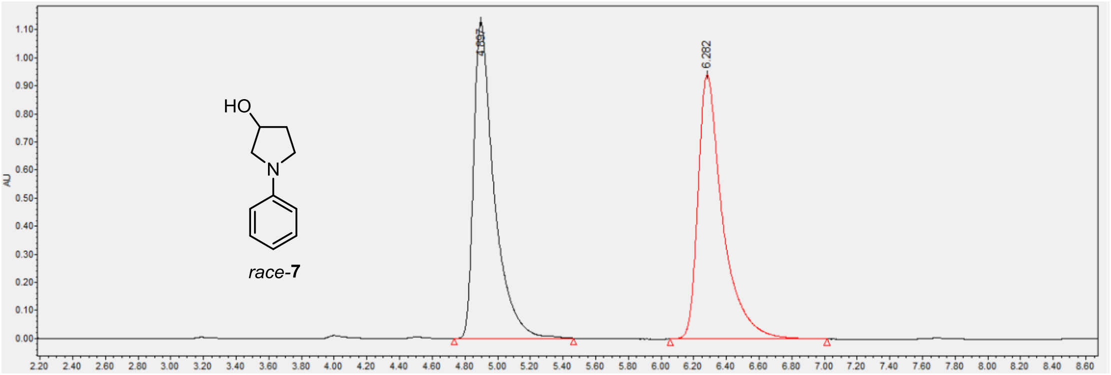

	Time/min	Area	Height	Area%
1	4.897	9834289	1128475	49.88
2	6.282	9882643	942887	50.12

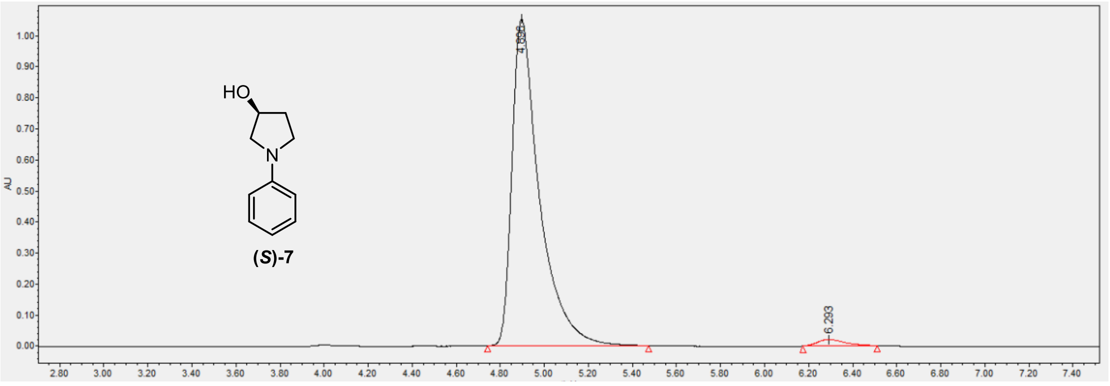

	Time/min	Area	Height	Area%
1	4.896	9098798	1052377	98.05
2	6.293	180872	20634	1.95

Supplementary Figure 284. HPLC spectra for **7**

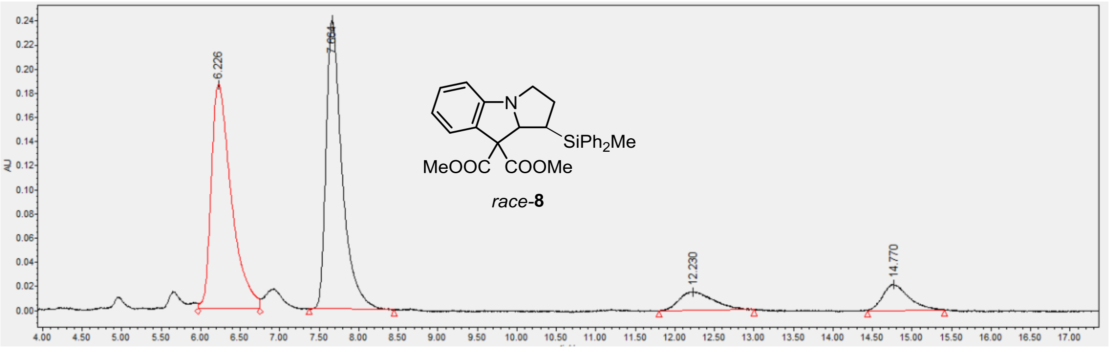

	Time/min	Area	Height	Area%
1	6.226	3282476	185138	43.15
2	7.664	3371073	239386	44.31
3	12.230	459106	15649	6.04
4	14.770	494090	21966	6.50

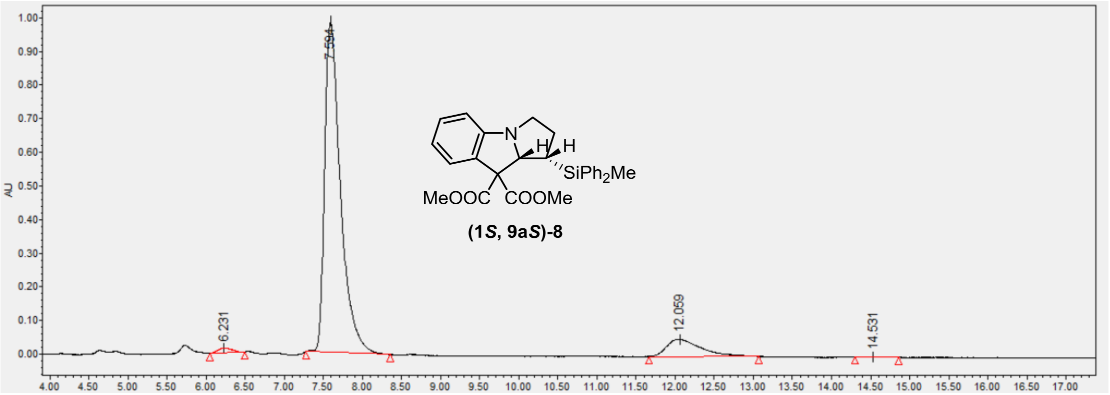

	Time/min	Area	Height	Area%
1	6.231	176715	14155	1.13
2	7.594	13835654	983780	88.64
3	12.059	1560608	51185	10.00
4	14.531	36708	2270	0.23

Supplementary Figure 285. HPLC spectra for **8**

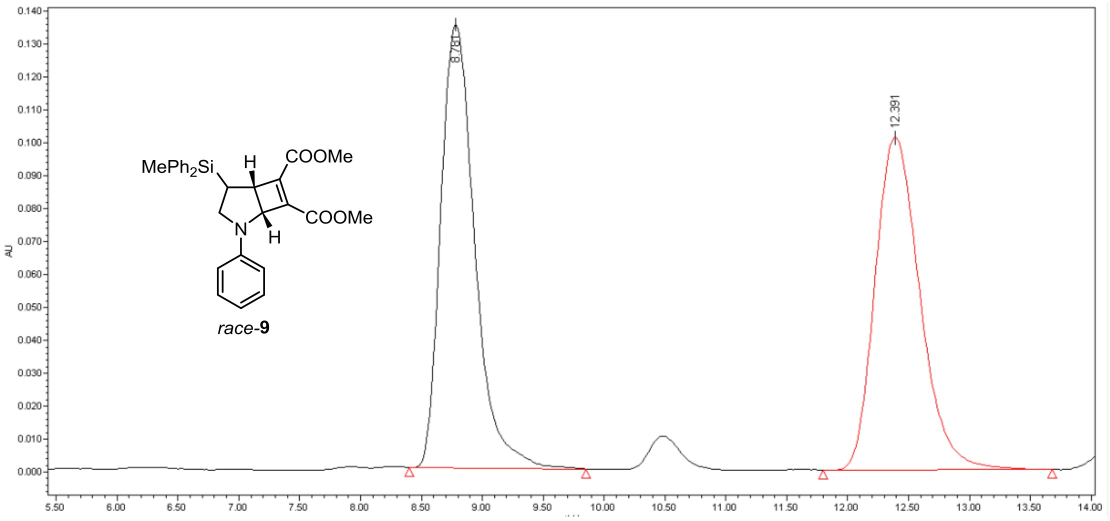

	Time/min	Area	Height	Area%
1	8.781	2613766	134741	50.31
2	12.391	2581633	101103	49.69

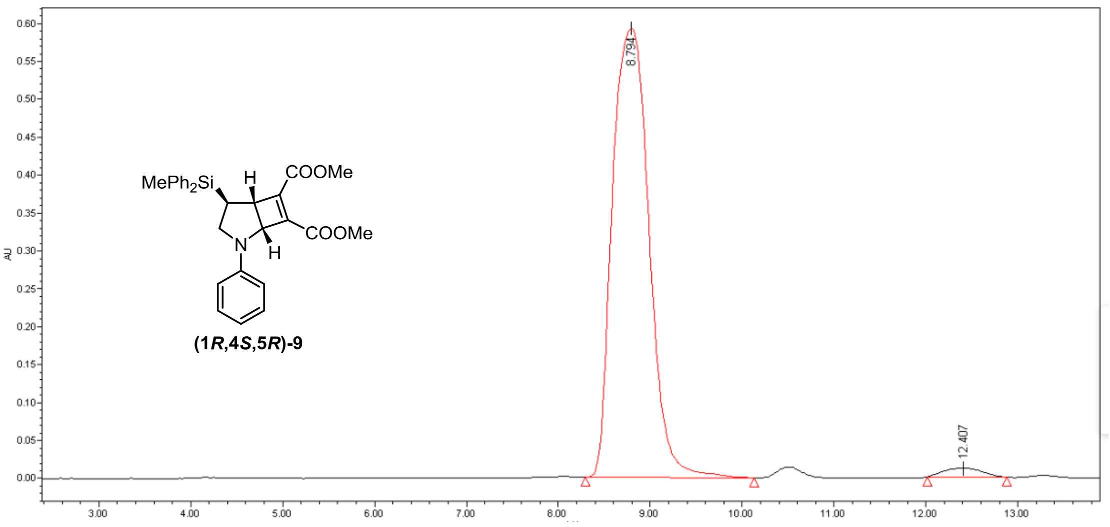

	Time/min	Area	Height	Area%
1	8.794	16364581	592064	97.99
2	12.407	334840	11638	2.01

Supplementary Figure 286. HPLC spectra for **9**

Supplementary References

1. Wan, W.; Ma, G. B.; Li, J. L.; Chen, Y. R.; Hu, Q. Y.; Li, M. J.; Jiang, H. Z.; Deng, H. M.; Hao, J. Silver-catalyzed oxidative decarboxylation of difluoroacetates: efficient access to C–CF_2 bond formation. *Chem. Commun.* **52**, 1598-1602 (2016).
2. Mandal, R.; Emayavaramban, B.; Sundararaju, B. Cp*Co(III)-Catalyzed C−H Alkylation with Maleimides Using Weakly Coordinating Carbonyl Directing Groups. *Org. Lett.* **20**, 2835 - 2838 (2018).
3. Sun, H. Y.; Kubota, K.; Hall, D. G. Reaction Optimization, Scalability, and Mechanistic Insight on the Catalytic Enantioselective Desymmetrization of 1,1-Diborylalkanes via Suzuki–Miyaura Cross-Coupling. *Chem. Eur. J.* 21, 19186 – 19194 (2015).
4. Sun, C. R.; Potter, B.; Morken, J. P. A Catalytic Enantiotopic-Group-Selective Suzuki Reaction for the Construction of Chiral Organoboronates. *J. Am. Chem. Soc.* **136**, 6534 – 6537 (2014).
5. Burks, H. E.; Liu, S. B.; Morken, J. P. Development, Mechanism, and Scope of the Palladium-Catalyzed Enantioselective Allene Diboration. *J. Am. Chem. Soc.* **129,** 8766 – 8773 (2007).
6. Dailler, D.; Rocaboy, R.; Baudoin, O. Synthesis of β‐Lactams by Palladium(0)‐Catalyzed C(sp^3)−H Carbamoylation. *Angew. Chem. Int. Ed.* 56, 7218 - 7222 (2017)
7. Dalton, D. M.; Rappe, A. K.; Rovis, T. Perfluorinated Taddol phosphoramidite as an L,Z-ligand on Rh(I) and Co(−I): evidence for bidentate coordination via metal–C_6F_5 interaction. *Chem. Sci.*, 4, 2062 - 2070 (2013)
8. Kurihara, Y.; Nishikawa, M.; Yamanoi, Y.; Nishihara, H. Synthesis of optically active tertiary silanes via Pd-catalyzed enantioselective arylation of secondary silanes. *Chem. Commun.*, **48**, 11564 - 11566 (2012).

9. Venuti, M. C.; Ort, O. Borane-Methyl Sulfide Reductive Cyclization of ω-Ester Alkylamides: A Convenient Synthesis of N-Substituted Cyclic Amines. *Synthesis* **1988**, 985 – 988 (1988).
10. Shin, K.; Joung, S.; Kim, Y.; Chang, S. Selective Synthesis of Silacycles by Borane‐Catalyzed Domino Hydrosilylation of Proximal Unsaturated Bonds: Tunable Approach to 1,n‐Diols. *Adv. Synth. Catal.* **359**, 3428 – 3436 (2017).
11. Zhao, Z. G.; Luo, Y. R.; Liu, S. Y.; Zhang, L.; Feng, L.; Wang, Y. Direct Cyclization of Tertiary Aryl Amines with Iodonium Ylides. *Angew. Chem. Int. Ed.* **57**, 3792 - 3796 (2018).
12. Xu, G. Q.; Xu, J. T.; Feng, Z. T.; Liang, H.; Wang, Z. Y.; Qin, Y.; Xu, P. F. Dual C(sp^3)–H Bond Functionalization of N‐Heterocycles through Sequential Visible‐Light Photocatalyzed Dehydrogenation/[2+2] Cycloaddition Reactions. *Angew. Chem. Int. Ed.* **57**, 5110 - 5114 (2018).
13. Frisch, M., Trucks, G., Schlegel, H., Scuseria, G., Robb, M., Cheeseman, J., Scalmani, G., Barone, V., Mennucci, B., Petersson, G., Nakatsuji, H., Caricato, M., Li, X., Hratchian, H., Izmaylov, A., Bloino, J., Zheng, G., Sonnenberg, J., Hada, M., Ehara, M., Toyota, K., Fukuda, R., Hasegawa, J., Ishida, M., Nakajima, T., Honda, Y., Kitao, O., Nakai, H., Vreven, T., Montgomery, J., Peralta, J., Ogliaro, F., Bearpark, M., Heyd, J., Brothers, E., Kudin, K., Staroverov, V., Kobayashi, R., Normand, J., Raghavachari, K., Rendell, A., Burant, J., Iyengar, S., Tomasi, J., Cossi, M., Rega, N., Millam, J., Klene, M., Knox, J., Cross, J., Bakken, V., Adamo, C., Jaramillo, J., Gomperts, R., Stratmann, R., Yazyev, O., Austin, A., Cammi, R., Pomelli, C., Ochterski, J., Martin, R., Morokuma, K., Zakrzewski, V., Voth, G., Salvador, P., Dannenberg, J., Dapprich, S., Daniels, A., Farkas, Foresman, J., Ortiz, J., Cioslowski, J., Fox, D., 2009. Gaussian 09, Revision C.01, Gaussian Inc Wallingford CT.
14. Ramírez-López, P.; Ros, A.; Estepa, B.; Fernández, R.; Fiser, B.; Gómez-Bengoa, E.; Lassaletta, J. M. A Dynamic Kinetic C–P Cross–Coupling for the Asymmetric Synthesis of Axially Chiral P,N Ligands. *ACS Catal.* **6**, 3955 (2016).

15. Iorio, N. D.; Champavert, F.; Erice, A.; Righi, P.; Mazzanti, A.; Bencivenni, G. Targeting remote axial chirality control of N-(2-*tert*-butylphenyl)succinimides by means of Michael addition type reactions. *Tetrahedron* **72**, 5191 (2016).

16. Iorio, N. D.; Soprani, L.; Crotti, S.; Marotta, E.; Mazzanti, A.; Righi, P.; Bencivenni, G. Michael Addition of Oxindoles to N-(2-*tert*-Butylphenyl)maleimides: Efficient Desymmetrization for the Synthesis of Atropisomeric Succinimides with Quaternary and Tertiary Stereocenters. *Synthesis* **49**, 1519-1530 (2017).

17. Eudier, F.; Righi, P.; Mazzanti, A.; Ciogli, Bencivenni, G. Organocatalytic Atroposelective Formal Diels–Alder Desymmetrization of N-Arylmaleimides. *Org. Lett.* **17**, 1728-1731 (2015).

18. Duan, W. L.; Imazaki, Y.; Shintani, R.; Hayashi, T. Asymmetric construction of chiral C–N axes through rhodium-catalyzed 1,4-addition. *Tetrahedron* **63**, 8529-8536 (2007).

19. Ruzziconi, R.; Spizzichino, S.; Lunazzi, L.; Mazzanti, A.; Schlosser, M. B Values as a Sensitive Measure of Steric Effects. *Chem. Eur. J.* **15**, 2645-2652 (2009).
