# Supplementary material for: Stereospecific Si-C coupling and remote control of axial chirality by enantioselective palladium-catalyzed hydrosilylation of maleimides
Source: Nat Commun. 2020 Jun 9;11:2904. doi: 10.1038/s41467-020-16716-5 (PMC7283218; doi:10.1038/s41467-020-16716-5)
Supplement: Supplementary file 3 — Description of Additional Supplementary Files [file 41467_2020_16716_MOESM3_ESM.pdf]

Description of Additional Supplementary Files

File Name: Supplementary Data 1
Description: Cartesian coordinates of all calculated intermediates
