# Supplementary material for: Stereospecific Si-C coupling and remote control of axial chirality by enantioselective palladium-catalyzed hydrosilylation of maleimides
Source: Nat Commun. 2020 Jun 9;11:2904. doi: 10.1038/s41467-020-16716-5 (PMC7283218; doi:10.1038/s41467-020-16716-5)
Supplement: Supplementary file 4 — Supplementary Data 1 [file 41467_2020_16716_MOESM4_ESM.pdf]

Cartesian coordinates of all calculated intermediates

A

C	1.148700	-0.203600	0.001800
C	0.666500	-1.618300	0.004300
C	-0.666400	-1.618300	0.004300
C	-1.148700	-0.203600	0.001900
N	0.000000	0.585800	0.004300
O	2.284200	0.207300	-0.008600
O	-2.284100	0.207200	-0.008600
H	-1.351800	-2.454300	0.002300
H	1.351900	-2.454300	0.002200
C	-0.000100	2.027900	0.004500
H	-0.896200	2.378900	-0.507000
H	0.002900	2.428900	1.021700
H	0.892800	2.379400	-0.512400

B

C	4.450000	0.918700	-0.921500
C	4.433200	-0.366800	-0.183900
C	3.628100	-0.199600	0.949400
C	3.107900	1.201400	0.952200
N	3.640400	1.803900	-0.182200
H	3.646800	-0.781000	1.862800
H	5.198900	-1.114300	-0.343300
C	3.261800	3.113100	-0.645400
H	2.261200	3.100700	-1.090000
H	3.263300	3.816400	0.188600
H	3.983800	3.424500	-1.400200
C	-1.171800	1.435100	0.557500
C	-2.390700	1.004300	-0.262100
C	-2.941500	-0.389400	0.024000
C	-2.488100	-1.523700	-0.905100
H	-2.135100	1.111800	-1.329800
H	-2.701400	-0.684500	1.055400
P	0.158100	-1.022500	0.010300
O	-1.022600	-1.622600	-0.925300
O	-0.056600	0.575200	0.172100
O	-4.350300	-0.240600	-0.103600
O	-3.472300	1.852000	0.083000
C	-4.676000	1.154400	-0.198600
C	-5.158300	1.459300	-1.605400
H	-4.369500	1.262300	-2.334300
H	-6.019000	0.835500	-1.856600
H	-5.451200	2.508300	-1.688800

C	-5.685200	1.503700	0.869000
H	-5.908900	2.572100	0.848300
H	-6.612400	0.950700	0.707800
H	-5.287100	1.245100	1.851500
N	-0.260000	-1.647100	1.571400
C	-0.229600	-3.108200	1.615800
H	-0.713500	-3.456200	2.533600
H	-0.758100	-3.536300	0.765000
H	0.801800	-3.496700	1.608200
C	0.583600	-1.109900	2.642800
H	0.062000	-1.195300	3.601700
H	1.529600	-1.667300	2.722900
H	0.830400	-0.062100	2.468700
C	-2.877900	-1.301500	-2.354700
H	-2.530000	-2.142800	-2.957100
H	-3.962800	-1.229500	-2.447500
H	-2.426100	-0.395400	-2.763200
C	-3.058800	-2.835300	-0.397600
H	-4.145000	-2.826200	-0.512300
H	-2.654500	-3.677000	-0.964800
H	-2.837600	-2.979700	0.661700
C	-0.723200	2.820800	0.132900
H	0.200100	3.080900	0.654500
H	-0.536800	2.851500	-0.943500
H	-1.493600	3.555700	0.374500
C	-1.413100	1.399200	2.055800
H	-0.520500	1.751200	2.577200
H	-2.242500	2.066600	2.299400
H	-1.654700	0.399800	2.418900
Pd	2.375300	-1.094300	-0.536700
O	5.021100	1.217800	-1.946600
O	2.378600	1.747600	1.756500

C

C	-2.350700	-1.723300	0.411400
C	-3.071000	-0.404400	0.698000
C	-3.426800	0.458300	-0.513000
C	-2.461100	1.600700	-0.858200
H	-2.457000	0.162900	1.418100
H	-3.535200	-0.171000	-1.407400
O	-1.084200	1.095900	-0.970200
O	-1.024900	-1.393400	-0.103700
O	-4.694100	1.016300	-0.184500
O	-4.341000	-0.713300	1.249100

C	-5.187300	0.399100	1.015600
C	-5.105000	1.388600	2.165000
H	-4.067600	1.668500	2.360500
H	-5.664500	2.296400	1.928500
H	-5.520500	0.949300	3.074700
C	-6.586500	-0.111000	0.768400
H	-6.961400	-0.638000	1.648200
H	-7.258300	0.720600	0.548300
H	-6.584600	-0.796300	-0.080400
N	-1.261000	-0.873100	-2.681400
C	-0.902600	-0.024600	-3.818400
H	-1.616000	-0.178700	-4.634100
H	-0.921100	1.029100	-3.541000
H	0.104200	-0.257400	-4.200200
C	-1.016500	-2.277900	-3.024400
H	-1.835300	-2.656600	-3.645000
H	-0.081900	-2.388100	-3.593700
H	-0.933300	-2.900100	-2.132100
C	-2.391800	2.663800	0.221800
H	-1.653000	3.419900	-0.055500
H	-3.363200	3.146100	0.340500
H	-2.089500	2.240200	1.180400
C	-2.870300	2.223800	-2.180400
H	-3.834400	2.724000	-2.061000
H	-2.132700	2.965000	-2.501400
H	-2.980100	1.469000	-2.960800
C	-2.061100	-2.454900	1.708100
H	-1.467000	-3.346200	1.495700
H	-1.497700	-1.813600	2.390300
H	-2.995800	-2.749800	2.189500
C	-3.104200	-2.627100	-0.546900
H	-2.551600	-3.560300	-0.675400
H	-4.084800	-2.857000	-0.124200
H	-3.248200	-2.172900	-1.527400
P	-0.454000	-0.385600	-1.227100
Pd	1.820100	-0.780800	-1.090000
C	3.453900	-2.677900	0.383000
C	3.349900	-2.335500	-1.052300
C	2.126200	-2.855600	-1.528300
C	1.454500	-3.558400	-0.404600
N	2.292000	-3.398800	0.701700
H	1.889800	-3.121600	-2.550800
H	4.235800	-2.108800	-1.630700
C	1.933200	-3.821000	2.029200

H	0.999300	-3.344500	2.341300
H	1.802400	-4.904900	2.072100
H	2.742100	-3.526500	2.699300
O	4.329800	-2.410000	1.185100
O	0.401100	-4.165000	-0.385700
Si	2.851200	1.497400	0.386300
C	4.618300	1.202800	0.898700
H	4.811100	0.129500	0.996700
H	5.321300	1.602800	0.164000
H	4.836100	1.665700	1.865400
C	1.670500	0.858800	1.704500
C	1.991100	-0.270700	2.474100
C	0.493300	1.552500	2.016100
C	1.168900	-0.681800	3.517700
H	2.905600	-0.825500	2.274500
C	-0.333500	1.137600	3.054700
H	0.230600	2.442200	1.450300
C	0.005100	0.021200	3.812200
H	1.445100	-1.546400	4.113400
H	-1.233300	1.700900	3.291600
H	-0.630700	-0.295000	4.633900
C	2.453600	3.287900	-0.006100
C	1.313900	3.591000	-0.767700
C	3.247000	4.350900	0.441600
C	0.976200	4.907800	-1.059200
H	0.680200	2.783000	-1.132400
C	2.915500	5.669600	0.145100
H	4.140200	4.150800	1.029300
C	1.778400	5.950100	-0.604400
H	0.089000	5.122300	-1.649000
H	3.545900	6.479700	0.498900
H	1.519900	6.978400	-0.836700
H	2.669800	0.868700	-0.998600

D

Si	-0.034900	1.260800	0.514900
H	-0.133400	1.710700	1.927500
C	-0.037800	2.768200	-0.598300
H	-0.965900	3.333600	-0.479200
H	0.790000	3.446100	-0.373300
H	0.036400	2.483000	-1.651300
C	-1.542000	0.191900	0.161900
C	-2.810200	0.628500	0.572200
C	-1.466300	-1.016700	-0.542800

C	-3.954900	-0.106800	0.290400
H	-2.905900	1.560100	1.127600
C	-2.608700	-1.759200	-0.825200
H	-0.498900	-1.386100	-0.875600
C	-3.855000	-1.305400	-0.409300
H	-4.925500	0.251600	0.619600
H	-2.525600	-2.694000	-1.371400
H	-4.746800	-1.884300	-0.628300
C	1.541100	0.271100	0.261700
C	1.726700	-0.971100	0.888300
C	2.572900	0.745100	-0.558400
C	2.891100	-1.706100	0.703200
H	0.943800	-1.372800	1.528900
C	3.741800	0.014900	-0.746900
H	2.464500	1.702300	-1.063100
C	3.902100	-1.213400	-0.116400
H	3.011500	-2.665100	1.197900
H	4.527500	0.403600	-1.387600
H	4.812200	-1.787000	-0.262400

(*S*)-F

C	-4.612900	-0.462900	-0.832400
C	-3.627900	-0.940200	-1.795000
C	-3.430000	-2.412500	-1.492700
C	-4.025500	-2.603300	-0.107100
N	-4.721400	-1.455500	0.184100
C	-5.434300	-1.248100	1.418200
H	-6.110800	-2.082000	1.613600
H	-4.740000	-1.162600	2.259900
H	-6.000000	-0.321500	1.316500
C	2.761000	-2.070900	-0.921500
C	3.532300	-0.875600	-0.351800
C	3.331400	-0.587900	1.135200
C	2.340300	0.536100	1.466900
H	3.281900	0.012300	-0.959300
H	2.992100	-1.493700	1.656900
P	0.354800	-0.929300	0.067900
O	1.115500	0.351000	0.671400
O	1.336800	-1.746100	-0.927000
O	4.625800	-0.252000	1.613000
O	4.911600	-1.186400	-0.439400
C	5.583700	-0.431900	0.558300
C	6.016300	0.916100	0.007800

H	5.175800	1.443300	-0.448600
H	6.418900	1.542600	0.807100
H	6.789900	0.781700	-0.751500
C	6.735100	-1.255700	1.081500
H	7.452400	-1.459400	0.284000
H	7.248600	-0.720700	1.882100
H	6.363300	-2.202800	1.474600
N	0.187500	-1.957900	1.443900
C	-0.754100	-1.377600	2.415200
H	-0.628700	-1.875900	3.381000
H	-0.574300	-0.309200	2.549300
H	-1.801000	-1.506000	2.097200
C	-0.227900	-3.328300	1.125200
H	-0.119600	-3.949000	2.019100
H	-1.281400	-3.386000	0.810400
H	0.398300	-3.748700	0.339500
C	2.843500	1.903800	1.051900
H	2.131400	2.673800	1.354400
H	3.801900	2.111900	1.529900
H	2.954700	1.975300	-0.032000
C	2.000400	0.495200	2.942800
H	2.898200	0.728700	3.521500
H	1.228200	1.227700	3.187700
H	1.655100	-0.497400	3.238200
C	3.110800	-2.242600	-2.387800
H	2.513800	-3.043500	-2.827800
H	2.910500	-1.321300	-2.939800
H	4.168900	-2.489000	-2.492800
C	3.002100	-3.356500	-0.152000
H	2.503800	-4.189400	-0.652800
H	4.073400	-3.567100	-0.120800
H	2.629500	-3.304000	0.872100
Pd	-1.649700	-0.632000	-0.998400
O	-5.264600	0.566200	-0.794600
O	-3.910600	-3.562900	0.628700
Si	-1.586000	1.709200	-0.952500
C	0.114700	2.335400	-1.503500
C	0.519300	3.642000	-1.193300
C	0.974400	1.563300	-2.297200
C	1.733700	4.152000	-1.644100
H	-0.125300	4.275200	-0.586100
C	2.187200	2.068200	-2.756200
H	0.687900	0.544400	-2.558000
C	2.572400	3.364900	-2.426500

H	2.024400	5.166100	-1.386400
H	2.833000	1.452600	-3.376900
H	3.518300	3.761600	-2.782900
C	-1.932500	2.186200	0.836600
C	-3.245300	2.085700	1.328900
C	-0.930200	2.578000	1.734900
C	-3.534500	2.352200	2.662500
H	-4.054700	1.793100	0.663800
C	-1.219600	2.858100	3.067000
H	0.093600	2.659100	1.381800
C	-2.523200	2.738700	3.537000
H	-4.557100	2.265900	3.019200
H	-0.426100	3.174400	3.739600
H	-2.750900	2.953900	4.576500
C	-2.879500	2.466300	-2.076000
H	-2.689900	2.193300	-3.117200
H	-3.885800	2.131500	-1.812000
H	-2.839400	3.558900	-2.001100
H	-2.374800	-2.764800	-1.466400
H	-3.912000	-3.092000	-2.207600
H	-3.771900	-0.612600	-2.820900

(*S*)-G

C	2.885400	-1.209500	-1.707000
C	4.222900	-1.075200	-1.035300
C	4.623200	-2.511200	-0.682200
C	3.373300	-3.336800	-0.892000
N	2.440700	-2.516300	-1.514000
C	1.120800	-2.969900	-1.887900
H	1.068900	-4.036400	-1.669900
H	0.362200	-2.433900	-1.308500
H	0.946500	-2.793100	-2.951300
O	2.262100	-0.362300	-2.312800
O	3.190000	-4.493200	-0.593900
Si	4.181600	0.153800	0.437600
C	4.025000	1.909200	-0.199300
C	2.810000	2.406000	-0.693300
C	5.140600	2.757900	-0.229900
C	2.714200	3.696900	-1.197200
H	1.928000	1.761900	-0.682200
C	5.049900	4.050600	-0.735600
H	6.098300	2.407800	0.148200
C	3.834900	4.521700	-1.220000
H	1.763900	4.061900	-1.575600

H	5.926400	4.690900	-0.748900
H	3.761100	5.530900	-1.613800
C	2.800300	-0.260400	1.647200
C	2.197100	0.772100	2.424700
C	2.522900	-1.596900	2.057400
C	1.489700	0.482800	3.595600
H	2.379000	1.810500	2.158100
C	1.817700	-1.871700	3.224700
H	2.931100	-2.434000	1.496600
C	1.327600	-0.831700	4.012800
H	1.070600	1.297100	4.178300
H	1.655400	-2.903700	3.518800
H	0.793700	-1.050600	4.931700
H	4.991900	-2.665800	0.335200
H	5.401000	-2.903700	-1.345200
C	5.834200	-0.045300	1.306100
H	6.680200	0.061300	0.619900
H	5.947300	0.704100	2.094500
H	5.917300	-1.025600	1.783300
H	4.911700	-0.605700	-1.746000
C	-3.495900	-1.651100	-0.391100
C	-4.317600	-0.583300	0.341700
C	-4.316900	0.808500	-0.281600
C	-3.351900	1.815300	0.354900
H	-3.968200	-0.544700	1.387300
H	-4.075600	0.747500	-1.350800
P	-1.207400	0.059500	-0.310700
O	-2.031400	1.208200	0.504600
O	-2.081400	-1.323300	-0.281200
O	-5.655300	1.267900	-0.138300
O	-5.680600	-0.972200	0.276500
C	-6.478300	0.201100	0.348600
C	-6.883100	0.486300	1.783700
H	-6.004800	0.531900	2.429800
H	-7.404500	1.444100	1.848800
H	-7.545900	-0.298300	2.155600
C	-7.659000	0.025300	-0.577700
H	-8.272800	-0.819900	-0.259500
H	-8.277800	0.924400	-0.577000
H	-7.305500	-0.157000	-1.593900
N	-1.435700	0.588700	-1.955800
C	-0.661800	1.806300	-2.220000
H	-1.042000	2.293400	-3.124700
H	-0.753900	2.510100	-1.390500

H	0.404900	1.581900	-2.370900
C	-1.099100	-0.405500	-2.978900
H	-1.537100	-0.098900	-3.935500
H	-0.013100	-0.503300	-3.110700
H	-1.506500	-1.381000	-2.718300
C	-3.744700	2.195000	1.771300
H	-3.004000	2.885800	2.178200
H	-4.722900	2.678500	1.780600
H	-3.774900	1.322800	2.427800
C	-3.264700	3.051500	-0.521400
H	-4.233600	3.558300	-0.527600
H	-2.513100	3.746700	-0.139900
H	-3.015700	2.785400	-1.549700
C	-3.631300	-2.975300	0.339800
H	-3.004700	-3.733300	-0.134400
H	-3.313700	-2.872500	1.379500
H	-4.670200	-3.310400	0.321400
C	-3.911200	-1.802800	-1.845000
H	-3.357000	-2.623600	-2.306100
H	-4.976400	-2.038800	-1.896600
H	-3.726600	-0.899200	-2.427200
Pd	0.864000	-0.141600	0.556200

(*S*)-H

C	2.071400	-1.399200	-0.383200
C	1.133700	-0.317500	-0.848700
C	2.029700	0.913000	-1.034600
C	3.375200	0.532700	-0.461300
N	3.306400	-0.815900	-0.122700
O	1.843700	-2.576400	-0.199000
O	4.348300	1.230700	-0.309900
H	2.166500	1.184200	-2.085600
C	4.411400	-1.549700	0.447400
H	5.245900	-0.856800	0.549100
H	4.135300	-1.951300	1.424900
H	4.691800	-2.383400	-0.198700
Si	-0.273700	-0.058100	0.437100
C	0.510000	-0.210900	2.130900
H	0.869300	-1.231500	2.292400
H	1.366200	0.463300	2.242300
H	-0.199600	0.018900	2.929800
C	-1.658300	-1.289400	0.145700
C	-1.408800	-2.591400	-0.314600
C	-2.987500	-0.932400	0.420900

C	-2.448900	-3.497400	-0.491700
H	-0.387600	-2.900900	-0.523100
C	-4.027300	-1.838800	0.246500
H	-3.216500	0.072100	0.771300
C	-3.758800	-3.124200	-0.211300
H	-2.234900	-4.500200	-0.849000
H	-5.048200	-1.540900	0.465700
H	-4.569500	-3.832800	-0.350700
C	-0.930300	1.682400	0.177000
C	-1.728000	1.978500	-0.938700
C	-0.596100	2.737300	1.036600
C	-2.170600	3.271900	-1.187000
H	-2.016300	1.179800	-1.620400
C	-1.038300	4.034300	0.794800
H	0.018600	2.546900	1.913700
C	-1.824500	4.303600	-0.319400
H	-2.787900	3.477200	-2.056100
H	-0.768400	4.835200	1.476300
H	-2.169500	5.314900	-0.510900
H	1.675900	1.822000	-0.538000
H	0.655800	-0.643900	-1.778100

I

C	-2.606100	-2.291600	0.337300
C	-3.443300	-1.012600	0.439800
C	-3.492900	-0.144900	-0.811600
C	-2.541600	1.056800	-0.842100
H	-3.072100	-0.435600	1.304300
H	-3.281900	-0.752400	-1.702000
O	-1.190700	0.634600	-0.446700
O	-1.199700	-1.923300	0.244500
O	-4.838300	0.310100	-0.879600
O	-4.795300	-1.395600	0.627200
C	-5.613800	-0.342400	0.133500
C	-5.940000	0.641400	1.243000
H	-5.026700	0.995700	1.725300
H	-6.471100	1.507300	0.841500
H	-6.568200	0.166400	2.000100
C	-6.839700	-0.954300	-0.500400
H	-7.419500	-1.505400	0.242700
H	-7.472600	-0.174400	-0.927300
H	-6.540300	-1.638900	-1.295500
N	-0.582600	-1.174600	-2.206700
C	0.130200	-0.229300	-3.077100

H	-0.228400	-0.342100	-4.105100
H	-0.055500	0.798600	-2.762200
H	1.215000	-0.411400	-3.074000
C	-0.170300	-2.541400	-2.545000
H	-0.646900	-2.839700	-3.484800
H	0.917900	-2.617800	-2.677600
H	-0.482600	-3.238400	-1.767400
C	-2.889300	2.130900	0.170600
H	-2.145400	2.929700	0.112800
H	-3.875500	2.549800	-0.035700
H	-2.880300	1.738100	1.190900
C	-2.530300	1.626300	-2.247600
H	-3.525400	2.017800	-2.476500
H	-1.808900	2.440000	-2.335800
H	-2.297100	0.851600	-2.980400
C	-2.705000	-3.060300	1.643000
H	-2.028900	-3.917200	1.626200
H	-2.428100	-2.421300	2.484700
H	-3.726100	-3.415300	1.794700
C	-3.012000	-3.169600	-0.833800
H	-2.463200	-4.113500	-0.800000
H	-4.079700	-3.392000	-0.768000
H	-2.812500	-2.697400	-1.796300
P	-0.369300	-0.761100	-0.539700
Pd	1.735900	-0.924600	0.403100
C	3.595300	-1.828300	-1.677400
C	2.874800	-2.473300	-0.542900
C	3.558100	-2.138000	0.641000
C	4.714300	-1.295300	0.283600
N	4.662500	-1.128500	-1.110500
H	3.542800	-2.679800	1.578300
H	2.275500	-3.358300	-0.718000
C	5.599300	-0.331400	-1.859300
H	5.497400	0.731400	-1.621200
H	6.623300	-0.635200	-1.633400
H	5.393500	-0.487800	-2.918300
O	3.357100	-1.881600	-2.868500
O	5.573300	-0.811400	0.993500
Si	2.243700	2.539600	1.373500
C	3.730900	1.906300	0.441900
H	4.239500	2.725000	-0.075400
H	4.464100	1.414200	1.085100
H	3.421900	1.173500	-0.313600
C	1.115300	3.475200	0.199900

C	1.085700	3.158600	-1.165100
C	0.328600	4.549700	0.636800
C	0.315900	3.893200	-2.059400
H	1.683500	2.329300	-1.539200
C	-0.455100	5.280800	-0.250600
H	0.339100	4.830600	1.688100
C	-0.458200	4.956200	-1.603100
H	0.320800	3.638200	-3.115700
H	-1.054300	6.111500	0.110300
H	-1.058000	5.532800	-2.300900
C	1.323700	1.122900	2.211200
C	-0.062300	1.201300	2.451800
C	2.018100	0.013600	2.739100
C	-0.725800	0.223300	3.174400
H	-0.621400	2.041600	2.047700
C	1.337900	-0.987300	3.447200
H	3.102200	-0.043600	2.656900
C	-0.026500	-0.881700	3.664700
H	-1.793400	0.314100	3.359600
H	1.890600	-1.836900	3.836900
H	-0.547100	-1.652800	4.223700
H	2.655300	3.495800	2.433300

(*S*)-J

C	2.784800	0.664900	1.599000
C	3.809700	-0.283600	0.965700
C	4.382800	0.142500	-0.382600
C	3.754000	-0.516500	-1.617000
H	3.342800	-1.281300	0.895500
H	4.298400	1.230300	-0.508100
O	2.291700	-0.404000	-1.548600
O	1.578500	0.655800	0.780100
O	5.757200	-0.211200	-0.313800
O	4.944100	-0.307300	1.813000
C	6.082600	-0.621100	1.021800
C	6.350600	-2.114700	1.041100
H	5.458000	-2.669200	0.745300
H	7.155400	-2.366700	0.346800
H	6.640900	-2.436300	2.043700
C	7.245900	0.202200	1.521200
H	7.480500	-0.053700	2.556300
H	8.129600	0.017600	0.907900
H	6.995400	1.262800	1.466600
N	1.906400	2.170400	-1.348900

C	1.656100	2.346900	-2.785500
H	2.246500	3.190400	-3.155700
H	1.945500	1.456400	-3.344200
H	0.594700	2.555900	-2.991600
C	1.312800	3.313700	-0.637700
H	1.863500	4.219700	-0.908700
H	0.253100	3.461600	-0.890900
H	1.393000	3.180500	0.439900
C	4.012100	-2.009600	-1.684800
H	3.489600	-2.429100	-2.547100
H	5.080200	-2.206700	-1.788400
H	3.644700	-2.522400	-0.793000
C	4.262600	0.173600	-2.868200
H	5.335600	-0.009300	-2.967800
H	3.761600	-0.213000	-3.758800
H	4.109400	1.252700	-2.811300
C	2.329800	0.099900	2.932000
H	1.516000	0.705800	3.337400
H	1.968100	-0.923300	2.809300
H	3.157600	0.095200	3.643600
C	3.319800	2.075600	1.768700
H	2.584700	2.695000	2.287500
H	4.228500	2.047800	2.373500
H	3.551900	2.549700	0.814200
P	1.287800	0.639800	-0.819300
Si	-2.971200	-1.072400	1.251600
C	-4.471000	-1.343700	0.150100
C	-5.161900	-0.346000	-0.548700
C	-4.942500	-2.660100	0.032400
C	-6.276200	-0.646900	-1.324500
H	-4.821300	0.683700	-0.502700
C	-6.054000	-2.969100	-0.743600
H	-4.428700	-3.466200	0.553700
C	-6.725700	-1.958800	-1.423100
H	-6.793700	0.146100	-1.855200
H	-6.396500	-3.996800	-0.816000
H	-7.595500	-2.193500	-2.028800
C	-1.502600	-1.992700	0.506300
C	-1.506400	-2.415200	-0.837800
C	-0.352500	-2.249100	1.262600
C	-0.404900	-3.058600	-1.395700
H	-2.399400	-2.258200	-1.439800
C	0.750700	-2.893800	0.708300
H	-0.312000	-1.935800	2.304500

C	0.726000	-3.299100	-0.621000
H	-0.431700	-3.378400	-2.432900
H	1.627100	-3.093300	1.320800
H	1.581400	-3.807600	-1.053700
C	-2.415200	2.736500	0.142400
C	-1.646800	1.519500	0.522600
C	-2.500400	0.767100	1.531900
C	-3.751700	1.598200	1.683300
N	-3.652100	2.666200	0.804800
H	-2.007400	0.741600	2.514600
H	-0.658500	1.782300	0.903100
C	-4.688500	3.658600	0.657800
H	-5.581100	3.220400	0.202900
H	-4.967900	4.064500	1.631900
H	-4.296500	4.449000	0.018200
O	-2.091500	3.676100	-0.554900
O	-4.703800	1.396400	2.408000
C	-3.338600	-1.765500	2.953600
H	-4.134400	-1.180100	3.422100
H	-3.662800	-2.808400	2.912100
H	-2.462100	-1.718000	3.606900
Pd	-1.082300	0.383000	-1.152200
H	-2.656000	0.499600	-1.340800

(*S*)-K

C	3.720200	1.324700	-1.174700
C	4.364300	0.538400	-0.029200
C	4.046900	-0.951600	0.037800
C	2.921100	-1.367000	0.989800
H	4.091900	1.038800	0.915200
H	3.796600	-1.330600	-0.963400
O	1.703300	-0.619700	0.695700
O	2.284400	1.383900	-0.940300
O	5.262200	-1.550500	0.472700
O	5.769700	0.558000	-0.221800
C	6.311900	-0.574400	0.442600
C	6.711300	-0.221900	1.864400
H	5.872400	0.226200	2.400900
H	7.022600	-1.118400	2.405400
H	7.539800	0.490000	1.862200
C	7.459100	-1.102800	-0.385900
H	8.250800	-0.355000	-0.465200
H	7.874300	-2.001900	0.073300
H	7.105800	-1.350800	-1.388100

N	1.266600	-0.844400	-1.869800
C	0.405600	-2.009000	-1.668600
H	0.638600	-2.771700	-2.418200
H	0.555800	-2.431100	-0.676200
H	-0.660200	-1.756200	-1.781000
C	0.911000	-0.218000	-3.144600
H	1.192900	-0.881500	-3.968100
H	-0.173100	-0.030100	-3.222900
H	1.433000	0.730600	-3.268000
C	3.216100	-1.050000	2.445700
H	2.362600	-1.354400	3.055400
H	4.104100	-1.587500	2.782800
H	3.366400	0.019700	2.604200
C	2.658600	-2.854200	0.824400
H	3.524100	-3.415800	1.184600
H	1.781800	-3.156300	1.401900
H	2.504600	-3.118300	-0.223600
C	4.168200	2.773500	-1.097400
H	3.660200	3.364000	-1.861900
H	3.925800	3.198000	-0.120700
H	5.246900	2.842200	-1.251600
C	4.043500	0.743100	-2.540900
H	3.655700	1.396800	-3.325400
H	5.127700	0.678300	-2.657100
H	3.614800	-0.248500	-2.681400
P	1.148800	0.280900	-0.540800
Pd	-0.846300	1.184700	-0.049900
Si	-3.752600	0.575900	0.892900
C	-2.653700	-0.690000	1.747100
C	-2.028600	-1.730900	1.042900
C	-2.412600	-0.609800	3.126100
C	-1.203200	-2.646700	1.682000
H	-2.179100	-1.828500	-0.029400
C	-1.564600	-1.507600	3.767600
H	-2.895100	0.166400	3.716500
C	-0.958600	-2.529500	3.046300
H	-0.745500	-3.450600	1.112500
H	-1.387800	-1.416500	4.835000
H	-0.305200	-3.238100	3.547000
C	-2.782900	2.158800	0.558500
C	-1.957000	2.724600	1.570100
C	-2.942500	2.919700	-0.627200
C	-1.427200	4.010600	1.437700
H	-1.800900	2.180600	2.499200

C	-2.413000	4.199400	-0.750400
H	-3.533500	2.515300	-1.445800
C	-1.673200	4.755200	0.291400
H	-0.821200	4.423700	2.237900
H	-2.575400	4.763100	-1.663700
H	-1.268000	5.757200	0.196400
C	-3.410500	-1.626600	-2.392900
C	-3.361500	-0.189900	-1.937400
C	-4.373500	-0.076400	-0.793800
C	-4.959200	-1.460000	-0.654900
N	-4.340300	-2.283500	-1.594200
H	-5.206000	0.590700	-1.051800
H	-2.324700	0.046400	-1.631100
C	-4.603500	-3.700100	-1.679600
H	-4.313200	-4.193500	-0.748500
H	-5.666100	-3.881500	-1.847700
H	-4.018000	-4.094100	-2.509700
O	-2.758800	-2.152700	-3.266200
O	-5.801300	-1.845500	0.123900
C	-5.230400	0.957100	1.972200
H	-5.820300	0.055000	2.150900
H	-4.922400	1.367000	2.938400
H	-5.878700	1.699100	1.497600
H	-3.572100	0.464100	-2.787900

$TS_{(\mathit{S})\text{-CF}}$

C	2.288500	-3.144700	-0.693900
C	3.090000	-2.107800	-1.347200
C	3.974500	-1.544400	-0.384400
C	3.699900	-2.255200	0.929800
N	2.654200	-3.125000	0.674700
C	2.053800	-3.957000	1.684700
H	1.343400	-3.390600	2.295800
H	1.529300	-4.770900	1.184400
H	2.829300	-4.355800	2.340100
O	1.482900	-3.925900	-1.159700
O	4.262300	-2.115900	1.990000
H	4.996700	-1.252800	-0.599900
H	3.242200	-2.114100	-2.417400
C	-1.752800	-2.305900	-0.381600
C	-2.707000	-1.266500	0.211300
C	-3.112000	-0.102900	-0.696200
C	-2.336200	1.208700	-0.512000
H	-2.252000	-0.889600	1.143400

H	-3.025400	-0.397600	-1.752700
P	0.022200	-0.246700	-1.157100
O	-0.889900	0.940900	-0.593300
O	-0.450100	-1.659600	-0.544600
O	-4.482000	0.118400	-0.393500
O	-3.940100	-1.913500	0.470900
C	-4.944100	-0.910800	0.497500
C	-5.102800	-0.348200	1.899100
H	-4.141800	-0.020000	2.300800
H	-5.780000	0.508800	1.891500
H	-5.512200	-1.109600	2.566700
C	-6.217800	-1.499200	-0.058100
H	-6.560900	-2.326400	0.566300
H	-7.001000	-0.739900	-0.091100
H	-6.041100	-1.867500	-1.069700
N	-0.410700	-0.359600	-2.814600
C	-0.169200	0.860900	-3.583100
H	-0.743700	0.823100	-4.513900
H	-0.472700	1.743000	-3.020700
H	0.894300	0.975500	-3.846000
C	0.186600	-1.514100	-3.491300
H	-0.388900	-1.742700	-4.393600
H	1.225300	-1.308900	-3.790700
H	0.192800	-2.391300	-2.844100
C	-2.530800	1.837900	0.853500
H	-1.926000	2.744100	0.921000
H	-3.580700	2.101100	0.992700
H	-2.217700	1.173800	1.661300
C	-2.703700	2.187500	-1.609800
H	-3.739600	2.507500	-1.473100
H	-2.055900	3.067500	-1.569800
H	-2.625300	1.727700	-2.597300
C	-1.487000	-3.401300	0.632600
H	-0.711000	-4.069100	0.253000
H	-1.148800	-2.973400	1.580000
H	-2.400400	-3.970100	0.815800
C	-2.236800	-2.899600	-1.691300
H	-1.559700	-3.695300	-2.009500
H	-3.229100	-3.330700	-1.540300
H	-2.297700	-2.159600	-2.489500
Pd	2.232200	-0.170300	-0.526600
Si	1.943300	1.775400	0.833100
C	0.807100	1.327500	2.288100
C	0.528400	2.258800	3.299400

C	0.242500	0.050800	2.422300
C	-0.306300	1.948700	4.368600
H	0.979800	3.248600	3.262100
C	-0.583000	-0.273100	3.495800
H	0.454800	-0.705500	1.667000
C	-0.870000	0.680600	4.467000
H	-0.509300	2.693700	5.132300
H	-1.001400	-1.273600	3.577700
H	-1.515000	0.432600	5.304400
C	1.179800	3.174600	-0.204400
C	1.496300	3.265300	-1.566200
C	0.327100	4.155100	0.320300
C	0.985200	4.277300	-2.372200
H	2.150200	2.512500	-2.007300
C	-0.193200	5.169900	-0.477900
H	0.049500	4.124300	1.371600
C	0.132300	5.233000	-1.829400
H	1.248200	4.317500	-3.425900
H	-0.857200	5.912900	-0.044700
H	-0.273800	6.023200	-2.453700
C	3.501100	2.515200	1.602100
H	4.001800	1.788900	2.248700
H	4.212000	2.826200	0.831500
H	3.266000	3.399000	2.205300
H	3.723100	-0.004800	0.137200

$TS_{(S)\text{-FG}}$

C	-4.563900	0.532700	0.074200
C	-3.668500	0.635100	1.269600
C	-3.385300	2.153700	1.350500
C	-3.626500	2.659100	-0.056200
N	-4.364400	1.679400	-0.703300
C	-4.848400	1.809100	-2.055800
H	-4.073000	1.519100	-2.772300
H	-5.706800	1.149200	-2.177400
H	-5.127600	2.846000	-2.243200
O	-5.318600	-0.364400	-0.235900
O	-3.300500	3.717200	-0.544800
H	-2.405800	2.497100	1.720000
H	-4.118100	2.627300	2.018100
H	-4.181000	0.282800	2.164700
C	3.271000	1.360700	1.091300
C	3.796200	0.121900	0.354500
C	3.646600	0.128200	-1.164800

C	2.445400	-0.644100	-1.721600
H	3.315300	-0.767900	0.798700
H	3.572700	1.161000	-1.532900
P	0.736500	1.022500	-0.151500
O	1.245700	-0.287700	-0.962500
O	1.816700	1.379300	1.005400
O	4.856100	-0.441500	-1.648300
O	5.202200	0.084800	0.538000
C	5.759100	-0.637500	-0.550000
C	5.849900	-2.116300	-0.218200
H	4.880800	-2.497900	0.109800
H	6.162400	-2.686600	-1.095700
H	6.574200	-2.282000	0.582500
C	7.093000	-0.020400	-0.894800
H	7.780100	-0.099000	-0.050000
H	7.535700	-0.531100	-1.751900
H	6.957500	1.033000	-1.144700
N	0.969700	2.286600	-1.324800
C	-0.001200	2.131800	-2.416500
H	0.266600	2.804200	-3.237100
H	-0.002800	1.108600	-2.794400
H	-1.025800	2.375400	-2.089400
C	0.794200	3.620500	-0.740000
H	1.051200	4.374500	-1.489500
H	-0.246300	3.799700	-0.422500
H	1.447200	3.753500	0.121400
C	2.557500	-2.145400	-1.539300
H	1.657800	-2.627200	-1.930200
H	3.426400	-2.533400	-2.072800
H	2.638400	-2.410100	-0.483400
C	2.253100	-0.291600	-3.184500
H	3.117900	-0.641200	-3.754700
H	1.357700	-0.771200	-3.588000
H	2.171200	0.788500	-3.319100
C	3.556600	1.220200	2.575200
H	3.108000	2.051600	3.122200
H	3.133300	0.289500	2.959200
H	4.634200	1.218300	2.750500
C	3.848400	2.657000	0.549300
H	3.548900	3.492600	1.186000
H	4.939400	2.600400	0.550100
H	3.508400	2.865700	-0.466000
Pd	-1.328200	0.834800	0.716300
Si	-2.583400	-1.283000	1.070000

C	-0.949600	-1.609400	1.969800
C	0.089200	-2.357800	1.391200
C	-0.751200	-1.116000	3.271600
C	1.271600	-2.594300	2.083400
H	-0.028800	-2.752800	0.385100
C	0.434700	-1.340700	3.958000
H	-1.537000	-0.535500	3.753000
C	1.452800	-2.082400	3.364900
H	2.055400	-3.193600	1.625900
H	0.565700	-0.940700	4.959000
H	2.378100	-2.268000	3.902500
C	-2.645100	-1.995300	-0.670600
C	-3.724300	-2.805300	-1.053900
C	-1.639200	-1.758000	-1.619800
C	-3.798000	-3.354200	-2.329400
H	-4.531200	-2.996600	-0.353900
C	-1.705900	-2.313500	-2.893100
H	-0.791000	-1.134600	-1.353300
C	-2.788600	-3.110100	-3.253200
H	-4.649300	-3.969700	-2.603500
H	-0.912300	-2.120000	-3.610300
H	-2.846100	-3.536600	-4.250300
C	-3.888800	-2.159400	2.098500
H	-3.842900	-1.832200	3.141800
H	-4.900000	-1.980000	1.726100
H	-3.699000	-3.237300	2.090500

TS$_{(S)\text{-IJ}}$

C	-4.563900	0.532700	0.074200
C	-3.668500	0.635100	1.269600
C	-3.385300	2.153700	1.350500
C	-3.626500	2.659100	-0.056200
N	-4.364400	1.679400	-0.703300
C	-4.848400	1.809100	-2.055800
H	-4.073000	1.519100	-2.772300
H	-5.706800	1.149200	-2.177400
H	-5.127600	2.846000	-2.243200
O	-5.318600	-0.364400	-0.235900
O	-3.300500	3.717200	-0.544800
H	-2.405800	2.497100	1.720000
H	-4.118100	2.627300	2.018100
H	-4.181000	0.282800	2.164700
C	3.271000	1.360700	1.091300
C	3.796200	0.121900	0.354500

C	3.646600	0.128200	-1.164800
C	2.445400	-0.644100	-1.721600
H	3.315300	-0.767900	0.798700
H	3.572700	1.161000	-1.532900
P	0.736500	1.022500	-0.151500
O	1.245700	-0.287700	-0.962500
O	1.816700	1.379300	1.005400
O	4.856100	-0.441500	-1.648300
O	5.202200	0.084800	0.538000
C	5.759100	-0.637500	-0.550000
C	5.849900	-2.116300	-0.218200
H	4.880800	-2.497900	0.109800
H	6.162400	-2.686600	-1.095700
H	6.574200	-2.282000	0.582500
C	7.093000	-0.020400	-0.894800
H	7.780100	-0.099000	-0.050000
H	7.535700	-0.531100	-1.751900
H	6.957500	1.033000	-1.144700
N	0.969700	2.286600	-1.324800
C	-0.001200	2.131800	-2.416500
H	0.266600	2.804200	-3.237100
H	-0.002800	1.108600	-2.794400
H	-1.025800	2.375400	-2.089400
C	0.794200	3.620500	-0.740000
H	1.051200	4.374500	-1.489500
H	-0.246300	3.799700	-0.422500
H	1.447200	3.753500	0.121400
C	2.557500	-2.145400	-1.539300
H	1.657800	-2.627200	-1.930200
H	3.426400	-2.533400	-2.072800
H	2.638400	-2.410100	-0.483400
C	2.253100	-0.291600	-3.184500
H	3.117900	-0.641200	-3.754700
H	1.357700	-0.771200	-3.588000
H	2.171200	0.788500	-3.319100
C	3.556600	1.220200	2.575200
H	3.108000	2.051600	3.122200
H	3.133300	0.289500	2.959200
H	4.634200	1.218300	2.750500
C	3.848400	2.657000	0.549300
H	3.548900	3.492600	1.186000
H	4.939400	2.600400	0.550100
H	3.508400	2.865700	-0.466000
Pd	-1.328200	0.834800	0.716300

Si	-2.583400	-1.283000	1.070000
C	-0.949600	-1.609400	1.969800
C	0.089200	-2.357800	1.391200
C	-0.751200	-1.116000	3.271600
C	1.271600	-2.594300	2.083400
H	-0.028800	-2.752800	0.385100
C	0.434700	-1.340700	3.958000
H	-1.537000	-0.535500	3.753000
C	1.452800	-2.082400	3.364900
H	2.055400	-3.193600	1.625900
H	0.565700	-0.940700	4.959000
H	2.378100	-2.268000	3.902500
C	-2.645100	-1.995300	-0.670600
C	-3.724300	-2.805300	-1.053900
C	-1.639200	-1.758000	-1.619800
C	-3.798000	-3.354200	-2.329400
H	-4.531200	-2.996600	-0.353900
C	-1.705900	-2.313500	-2.893100
H	-0.791000	-1.134600	-1.353300
C	-2.788600	-3.110100	-3.253200
H	-4.649300	-3.969700	-2.603500
H	-0.912300	-2.120000	-3.610300
H	-2.846100	-3.536600	-4.250300
C	-3.888800	-2.159400	2.098500
H	-3.842900	-1.832200	3.141800
H	-4.900000	-1.980000	1.726100
H	-3.699000	-3.237300	2.090500

(*S*)-3a

C	-1.335300	0.470700	-0.873400
C	-0.044300	-0.255000	-1.120000
C	-0.447100	-1.726000	-1.234700
C	-1.856500	-1.804200	-0.699400
N	-2.318600	-0.485100	-0.556700
O	-1.532000	1.664300	-0.882200
O	-2.491600	-2.795600	-0.445700
H	-0.463000	-2.076300	-2.271700
Si	1.183300	0.038900	0.332200
C	0.207800	-0.207300	1.911900
H	-0.564100	0.561200	2.014200
H	-0.291800	-1.181800	1.940500
H	0.852100	-0.143000	2.792800
C	2.005400	1.721500	0.225200

C	1.423500	2.825900	-0.413800
C	3.258100	1.899400	0.835300
C	2.069500	4.057500	-0.438200
H	0.449200	2.725500	-0.882800
C	3.902100	3.130500	0.814600
H	3.742800	1.058900	1.328500
C	3.307300	4.213400	0.175300
H	1.601700	4.900200	-0.938000
H	4.869800	3.244200	1.293400
H	3.809000	5.176200	0.155500
C	2.518400	-1.273900	0.167600
C	3.517400	-1.129500	-0.806800
C	2.538200	-2.431700	0.956200
C	4.494500	-2.100700	-0.987900
H	3.538000	-0.233800	-1.425000
C	3.516400	-3.406400	0.782300
H	1.782100	-2.576600	1.724800
C	4.494700	-3.242200	-0.191800
H	5.259300	-1.967200	-1.746700
H	3.515300	-4.293800	1.407300
H	5.258100	-4.001300	-0.329500
H	0.187600	-2.431200	-0.689800
H	0.420800	0.145500	-2.026200
C	-3.631600	-0.144200	-0.125500
C	-4.335600	0.861800	-0.785200
C	-4.202700	-0.814300	0.954800
C	-5.611100	1.199300	-0.353900
H	-3.879400	1.379900	-1.618300
C	-5.484000	-0.473500	1.369200
H	-3.650700	-1.601500	1.452700
C	-6.190100	0.534000	0.720600
H	-6.155600	1.986000	-0.865400
H	-5.929800	-0.999200	2.207100
H	-7.189100	0.798300	1.051600

3a-TS-Rot-*anti*

C	-1.339100	0.531000	-0.543000
C	-0.049500	-0.196300	-0.847200
C	-0.429800	-1.679500	-1.017500
C	-1.910500	-1.787800	-0.690600
N	-2.384100	-0.459600	-0.433000
O	-1.557100	1.713900	-0.432700
O	-2.596200	-2.776200	-0.647200
H	-0.240800	-2.036800	-2.049500

Si	1.320300	0.078100	0.470500
C	0.557300	0.065300	2.163800
H	-0.143600	0.912700	2.276800
H	-0.002000	-0.856100	2.382300
H	1.328200	0.185200	2.944400
C	2.146900	1.718500	0.144400
C	1.380500	2.860500	-0.145400
C	3.543600	1.845700	0.239400
C	1.998700	4.099700	-0.344100
H	0.285500	2.801200	-0.220100
C	4.161100	3.085700	0.041300
H	4.169000	0.979300	0.469700
C	3.389000	4.213700	-0.251800
H	1.393600	4.976000	-0.569300
H	5.242900	3.171800	0.116700
H	3.869000	5.178400	-0.406300
C	2.559600	-1.306900	0.239200
C	3.298700	-1.375500	-0.955900
C	2.766300	-2.289700	1.219400
C	4.220200	-2.406100	-1.165100
H	3.167400	-0.620700	-1.735600
C	3.689800	-3.321600	1.010500
H	2.213400	-2.265200	2.160700
C	4.416200	-3.381300	-0.181500
H	4.787100	-2.449400	-2.093200
H	3.841500	-4.077800	1.778500
H	5.133600	-4.183900	-0.344600
H	0.149700	-2.370300	-0.365900
H	0.389900	0.217100	-1.797600
C	-3.760900	-0.143800	-0.106400
C	-4.668300	0.126100	-1.141800
C	-4.157300	-0.114900	1.239200
C	-5.992800	0.428700	-0.815500
H	-4.345500	0.100500	-2.180600
C	-5.486400	0.189900	1.544000
H	-3.441900	-0.327200	2.030700
C	-6.402300	0.460900	0.521800
H	-6.708600	0.640300	-1.609100
H	-5.808800	0.215500	2.584200
H	-7.436000	0.697300	0.767500

3a-TS-Rot-*syn*

C	-1.339100	0.531500	-0.544400
C	-0.049100	-0.195100	-0.848800

C	-0.429100	-1.678000	-1.021700
C	-1.909700	-1.787300	-0.694800
N	-2.384100	-0.459400	-0.437100
O	-1.557400	1.714200	-0.432600
O	-2.594900	-2.776000	-0.651600
H	-0.240200	-2.033400	-2.054400
Si	1.319700	0.077600	0.470300
C	0.555500	0.061900	2.163100
H	-0.146300	0.908600	2.276600
H	-0.003200	-0.860300	2.380000
H	1.325700	0.181500	2.944300
C	2.146100	1.718700	0.147400
C	1.379600	2.861000	-0.140800
C	3.542700	1.846000	0.243100
C	1.997700	4.100700	-0.337000
H	0.284600	2.801700	-0.216000
C	4.160100	3.086300	0.047400
H	4.168300	0.979200	0.472000
C	3.387900	4.214700	-0.244000
H	1.392500	4.977300	-0.560900
H	5.241900	3.172500	0.123300
H	3.867800	5.179800	-0.396600
C	2.559400	-1.306800	0.237700
C	3.299000	-1.373700	-0.957200
C	2.766100	-2.290700	1.216800
C	4.220900	-2.403700	-1.167400
H	3.167800	-0.617900	-1.736000
C	3.690000	-3.322100	1.006900
H	2.212800	-2.267600	2.157900
C	4.416800	-3.380100	-0.184900
H	4.788100	-2.445700	-2.095300
H	3.841600	-4.079200	1.774000
H	5.134600	-4.182200	-0.348800
H	0.150700	-2.369900	-0.371400
H	0.390900	0.219800	-1.798300
C	-3.760500	-0.144600	-0.107900
C	-4.160800	-0.141100	1.236800
C	-4.663500	0.150600	-1.140300
C	-5.489500	0.163200	1.543800
H	-3.448800	-0.373000	2.025900
C	-5.987600	0.452600	-0.811800
H	-4.337500	0.145400	-2.178400
C	-6.401100	0.459200	0.524700
H	-5.815000	0.168900	2.583400

H	-6.700000	0.683700	-1.602900
H	-7.434500	0.695100	0.772200

(*P*,*S*)-**3ee**

C	-1.210900	0.557300	-0.638000
C	0.084300	-0.083600	-1.051100
C	-0.287400	-1.526300	-1.396300
C	-1.692600	-1.720600	-0.878000
N	-2.178000	-0.450500	-0.520300
O	-1.417500	1.721700	-0.375700
O	-2.310300	-2.749000	-0.778800
H	-0.296100	-1.713300	-2.474800
Si	1.349900	0.053300	0.396200
C	0.381700	-0.199100	1.978600
H	-0.344600	0.606800	2.117900
H	-0.173900	-1.143300	1.961300
H	1.032000	-0.213500	2.856700
C	2.252600	1.695300	0.305100
C	1.620600	2.870400	-0.130100
C	3.602100	1.775900	0.683000
C	2.313700	4.074500	-0.187300
H	0.573100	2.841300	-0.418900
C	4.294800	2.979900	0.627800
H	4.124500	0.882000	1.017300
C	3.651200	4.132000	0.189500
H	1.806800	4.972200	-0.528400
H	5.338800	3.017700	0.923200
H	4.191700	5.072400	0.141100
C	2.598400	-1.331100	0.163200
C	3.523200	-1.267100	-0.890300
C	2.628900	-2.461900	0.989400
C	4.442600	-2.285700	-1.106900
H	3.532900	-0.396900	-1.544600
C	3.550100	-3.484100	0.780000
H	1.927400	-2.547700	1.816400
C	4.458000	-3.396800	-0.268800
H	5.151000	-2.213600	-1.926500
H	3.558700	-4.348900	1.436000
H	5.177600	-4.192700	-0.433000
H	0.363600	-2.292000	-0.963800
H	0.503300	0.473300	-1.895200
C	-3.473100	-0.235500	0.039300
C	-4.373900	0.647900	-0.568700
C	-3.810800	-0.937500	1.193400

C	-5.615600	0.818400	0.048500
C	-5.055100	-0.756800	1.778000
H	-3.092500	-1.629300	1.619000
C	-5.957400	0.132900	1.205900
H	-6.329300	1.500300	-0.405200
H	-5.316600	-1.306400	2.675700
H	-6.932900	0.285800	1.655800
C	-4.049500	1.377800	-1.833000
H	-3.471200	2.282600	-1.628100
H	-3.448000	0.766200	-2.510900
H	-4.961400	1.666300	-2.358800

3ee-TS-Rot-*anti*

C	-1.106200	0.639900	-0.901100
C	0.160500	-0.162700	-1.060800
C	-0.311100	-1.610200	-1.214400
C	-1.741200	-1.614800	-0.696900
N	-2.248200	-0.259900	-0.747400
O	-1.204600	1.843300	-0.976300
O	-2.312900	-2.591000	-0.288400
H	-0.284800	-1.945800	-2.271000
Si	1.408400	0.064300	0.385100
C	0.476200	0.110700	1.989700
H	-0.167700	1.007400	2.044300
H	-0.171700	-0.765600	2.144800
H	1.170400	0.168000	2.846100
C	2.357900	1.651300	0.137200
C	1.740900	2.805800	-0.372300
C	3.709500	1.718000	0.520300
C	2.461500	3.998100	-0.504000
H	0.684400	2.794900	-0.673100
C	4.429000	2.910600	0.389400
H	4.219400	0.839600	0.925300
C	3.805600	4.051900	-0.124300
H	1.971300	4.885000	-0.901500
H	5.474200	2.950100	0.688500
H	4.365200	4.979900	-0.226700
C	2.588500	-1.386300	0.288500
C	3.511300	-1.454900	-0.770900
C	2.567800	-2.421600	1.236000
C	4.391100	-2.536400	-0.879800
H	3.558100	-0.660500	-1.520700
C	3.449300	-3.504200	1.127700
H	1.866300	-2.399600	2.072900

C	4.360500	-3.562900	0.070000
H	5.101800	-2.579100	-1.703000
H	3.424100	-4.300800	1.869000
H	5.045700	-4.404800	-0.014700
H	0.299500	-2.357600	-0.661400
H	0.718700	0.193400	-1.971000
C	-3.550700	0.184800	-0.269700
C	-4.632800	-0.685700	0.024400
C	-3.707600	1.558300	0.020000
C	-5.745600	-0.176500	0.721000
C	-4.840800	2.045400	0.667500
H	-2.941100	2.289700	-0.262100
C	-5.853600	1.169200	1.060500
H	-6.552600	-0.859500	0.989300
H	-4.925200	3.111400	0.872900
H	-6.721300	1.533300	1.605200
C	-4.775300	-2.086500	-0.471500
H	-4.278000	-2.243300	-1.437700
H	-4.339200	-2.814400	0.235400
H	-5.827200	-2.368700	-0.612900

3ee-TS-Rot-*syn*

C	-1.114100	0.622000	-1.160600
C	0.176500	-0.147400	-1.303300
C	-0.257000	-1.571900	-1.644000
C	-1.748500	-1.631600	-1.357800
N	-2.233200	-0.307100	-1.001700
O	-1.252700	1.810900	-1.316900
O	-2.425300	-2.623700	-1.460700
H	-0.059700	-1.824800	-2.705900
Si	1.245000	0.037500	0.284000
C	0.100300	0.199400	1.740500
H	-0.592000	1.059100	1.618000
H	-0.523400	-0.693900	1.900400
H	0.669100	0.379100	2.668900
C	2.272500	1.585100	0.105400
C	1.718400	2.753900	-0.444900
C	3.598500	1.615900	0.569800
C	2.477400	3.925600	-0.533800
H	0.684200	2.768900	-0.813500
C	4.356800	2.788800	0.480900
H	4.058100	0.725200	1.006400
C	3.797000	3.944300	-0.071800
H	2.038000	4.824200	-0.963500

H	5.382700	2.801000	0.842900
H	4.386600	4.856800	-0.141600
C	2.354700	-1.462200	0.397800
C	3.350400	-1.651600	-0.578200
C	2.216900	-2.415200	1.418700
C	4.186000	-2.771700	-0.533700
H	3.487900	-0.923300	-1.382000
C	3.054400	-3.536800	1.463500
H	1.457300	-2.297800	2.194500
C	4.038000	-3.716300	0.487600
H	4.953400	-2.909100	-1.293400
H	2.938000	-4.269500	2.260000
H	4.688700	-4.588700	0.521800
H	0.268300	-2.361100	-1.061800
H	0.800700	0.298300	-2.126500
C	-3.389000	-0.136500	-0.136300
C	-3.755400	1.087100	0.482000
C	-4.291700	-1.221300	-0.049600
C	-5.005600	1.180200	1.123500
C	-5.507300	-1.112300	0.620500
H	-4.069500	-2.184400	-0.525400
C	-5.882900	0.102400	1.196700
H	-5.292100	2.129500	1.578200
H	-6.169500	-1.975100	0.675500
H	-6.843600	0.205300	1.695600
C	-2.871900	2.281000	0.619100
H	-1.864800	2.011700	0.980600
H	-2.749200	2.805800	-0.347600
H	-3.269500	3.022600	1.324000

(*P*,*S*)-**3ff**

C	-1.072800	0.454900	-0.492900
C	0.227700	-0.093600	-1.015000
C	-0.084900	-1.543800	-1.389500
C	-1.420600	-1.849100	-0.753000
N	-1.946700	-0.627200	-0.309500
O	-1.339400	1.595300	-0.191900
O	-1.959700	-2.919000	-0.632300
H	-0.191500	-1.682400	-2.470100
Si	1.562400	0.057300	0.365600
C	0.690600	-0.301000	1.985100
H	-0.066700	0.461400	2.190000
H	0.182700	-1.271600	1.963500
H	1.388000	-0.310600	2.826600
C	2.367500	1.751200	0.322600

C	1.637600	2.909100	0.012600
C	3.725500	1.895600	0.645600
C	2.245000	4.159900	0.026100
H	0.580800	2.829000	-0.230600
C	4.332800	3.146400	0.659700
H	4.320400	1.017200	0.886300
C	3.592400	4.281400	0.348500
H	1.662900	5.043800	-0.216100
H	5.385200	3.234600	0.912200
H	4.065200	5.258900	0.357500
C	2.862200	-1.261500	0.043600
C	3.791900	-1.129400	-0.998900
C	2.909800	-2.430900	0.814100
C	4.731600	-2.120500	-1.256600
H	3.788000	-0.230200	-1.612500
C	3.848400	-3.426300	0.562000
H	2.204700	-2.567700	1.630700
C	4.761400	-3.271500	-0.474300
H	5.443900	-1.995600	-2.066400
H	3.867700	-4.322000	1.174800
H	5.496100	-4.045300	-0.673500
H	0.651700	-2.282000	-1.060200
H	0.558500	0.515800	-1.861400
C	-3.219100	-0.494900	0.305600
C	-4.208400	0.277100	-0.323700
C	-3.482000	-1.126100	1.511000
C	-5.443300	0.436300	0.304300
C	-4.722600	-0.983900	2.123400
H	-2.702200	-1.730200	1.962300
C	-5.691000	-0.194400	1.519900
H	-6.216100	1.040300	-0.155200
H	-4.923500	-1.479400	3.066200
H	-6.659100	-0.065300	1.992900
O	-3.878800	0.800400	-1.526700
C	-4.806000	1.682700	-2.125200
H	-5.011200	2.543500	-1.479300
H	-4.341900	2.027600	-3.047200
H	-5.749300	1.177200	-2.364000

3ff-TS-Rot-*anti*

C	-0.849400	0.849400	-0.765600
C	0.342500	-0.052100	-0.981900
C	-0.248300	-1.459400	-1.133900
C	-1.654100	-1.361300	-0.559800

N	-2.044300	0.036700	-0.623700
O	-0.857600	2.060600	-0.774400
O	-2.276600	-2.267000	-0.077400
H	-0.290900	-1.776400	-2.195500
Si	1.659300	0.048200	0.414300
C	0.799200	0.135600	2.057300
H	0.228800	1.077800	2.149300
H	0.092000	-0.691600	2.223200
H	1.528000	0.124200	2.885900
C	2.722400	1.560100	0.155300
C	2.172400	2.774000	-0.288600
C	4.093900	1.509700	0.462400
C	2.977400	3.909800	-0.430900
H	1.103200	2.854300	-0.528900
C	4.898000	2.645700	0.321000
H	4.553500	0.582600	0.815500
C	4.340300	3.847100	-0.127300
H	2.538400	4.843600	-0.777200
H	5.957800	2.594500	0.560900
H	4.965700	4.731000	-0.238000
C	2.718900	-1.487400	0.246800
C	3.579900	-1.613400	-0.858300
C	2.666800	-2.528900	1.186200
C	4.368700	-2.756800	-1.019600
H	3.649200	-0.816500	-1.603500
C	3.457300	-3.673500	1.025400
H	2.011000	-2.463600	2.057100
C	4.307700	-3.788800	-0.077200
H	5.032000	-2.843600	-1.878000
H	3.408500	-4.474300	1.760900
H	4.922000	-4.678800	-0.202700
H	0.324700	-2.258700	-0.616000
H	0.889500	0.270000	-1.911200
C	-3.332300	0.573500	-0.248200
C	-4.478600	-0.248100	-0.009300
C	-3.482300	1.950700	-0.019500
C	-5.654400	0.281600	0.535800
C	-4.672400	2.484300	0.487400
H	-2.659000	2.645500	-0.230300
C	-5.745900	1.651500	0.793400
H	-6.503500	-0.361900	0.745000
H	-4.745500	3.557700	0.650300
H	-6.660200	2.062300	1.218100
O	-4.384400	-1.536100	-0.471400

C	-5.387300	-2.494700	-0.051800
H	-6.347900	-2.259100	-0.515900
H	-4.964200	-3.427700	-0.450400
H	-5.449200	-2.535600	1.038600

3ff-TS-Rot-*syn*

C	1.046000	0.367300	1.231200
C	-0.307500	-0.290100	1.342500
C	-0.006600	-1.754700	1.671900
C	1.464700	-1.959800	1.351800
N	2.074300	-0.674100	1.062500
O	1.317000	1.523800	1.428900
O	2.050400	-3.013400	1.376200
H	-0.205500	-1.987700	2.737600
Si	-1.311000	0.003000	-0.271600
C	-0.133700	0.025900	-1.704900
H	0.648100	0.802100	-1.560100
H	0.393600	-0.928200	-1.855600
H	-0.652900	0.274400	-2.645900
C	-2.172800	1.652500	-0.114400
C	-1.491700	2.765500	0.408500
C	-3.493400	1.815800	-0.565500
C	-2.121900	4.012100	0.485100
H	-0.456900	2.676000	0.764600
C	-4.122800	3.063500	-0.489700
H	-4.049300	0.971600	-0.982100
C	-3.437800	4.162400	0.036700
H	-1.585900	4.866000	0.894500
H	-5.145800	3.177900	-0.841700
H	-3.927100	5.132800	0.096600
C	-2.579400	-1.367000	-0.393800
C	-3.588900	-1.457700	0.581900
C	-2.547100	-2.318000	-1.425100
C	-4.541400	-2.479800	0.527400
H	-3.645600	-0.727500	1.393500
C	-3.501600	-3.341500	-1.480200
H	-1.779800	-2.274900	-2.201200
C	-4.497900	-3.423800	-0.504300
H	-5.318300	-2.541100	1.287000
H	-3.466200	-4.073600	-2.284800
H	-5.239500	-4.219800	-0.546300
H	-0.621500	-2.484400	1.100000
H	-0.909500	0.196800	2.157100
C	3.178600	-0.580600	0.140800

C	3.589100	0.651300	-0.438200
C	4.025800	-1.689700	-0.053100
C	4.807000	0.778600	-1.117300
C	5.222600	-1.570700	-0.761800
H	3.766400	-2.668000	0.371700
C	5.631100	-0.335800	-1.271500
H	5.091400	1.742000	-1.534200
H	5.849700	-2.450900	-0.898100
H	6.580900	-0.243600	-1.794800
O	2.643000	1.658600	-0.500600
C	3.080700	2.980000	-0.085100
H	3.819200	3.371100	-0.786400
H	2.139900	3.544000	-0.121400
H	3.457900	2.935000	0.941400

(*P*,*S*)-**3gg**

C	-0.744200	0.543200	-0.267300
C	0.481600	-0.155600	-0.792200
C	0.056900	-1.618000	-0.950600
C	-1.260500	-1.737000	-0.222800
N	-1.719500	-0.429800	-0.000600
O	-0.898700	1.724600	-0.055900
O	-1.843800	-2.737700	0.106000
H	-0.115900	-1.891400	-1.997400
Si	1.952100	0.077600	0.428000
C	1.240200	-0.080800	2.153300
H	0.548600	0.741900	2.357100
H	0.689600	-1.020500	2.275800
H	2.020200	-0.053100	2.918500
C	2.817800	1.714000	0.127400
C	2.126600	2.862500	-0.287300
C	4.199100	1.822600	0.352700
C	2.792800	4.069700	-0.468100
H	1.054000	2.811400	-0.455300
C	4.864900	3.029800	0.174500
H	4.766200	0.948900	0.667100
C	4.161400	4.156500	-0.237500
H	2.239500	4.947200	-0.788600
H	5.934000	3.090800	0.354500
H	4.679700	5.100100	-0.379000
C	3.161100	-1.323500	0.101800
C	3.968700	-1.316600	-1.045200
C	3.255000	-2.428400	0.958300
C	4.834500	-2.367100	-1.324300

H	3.928000	-0.467500	-1.725800
C	4.122000	-3.481900	0.686200
H	2.643800	-2.468900	1.857000
C	4.912700	-3.452400	-0.456500
H	5.452000	-2.339500	-2.216600
H	4.180100	-4.326400	1.365900
H	5.590000	-4.273100	-0.671300
H	0.759900	-2.357400	-0.557300
H	0.772300	0.316500	-1.736600
C	-2.923100	-0.137500	0.723300
C	-4.150500	0.131800	0.089900
C	-2.797300	-0.136200	2.109700
C	-5.224800	0.406600	0.946600
C	-3.883200	0.137200	2.924700
C	-5.104600	0.412400	2.330300
H	-6.197000	0.625500	0.525000
H	-3.773800	0.134400	4.003600
H	-5.974500	0.632800	2.940600
H	-1.824900	-0.356600	2.539100
C	-4.353300	0.123000	-1.434400
C	-4.081100	-1.277500	-2.004200
H	-3.032500	-1.563900	-1.932200
H	-4.665100	-2.041900	-1.484400
H	-4.353500	-1.305000	-3.064000
C	-5.794300	0.487400	-1.808100
H	-6.069400	1.489600	-1.467200
H	-5.895300	0.472900	-2.896200
H	-6.520200	-0.223700	-1.404600
C	-3.438400	1.145500	-2.125500
H	-3.523200	2.136100	-1.671300
H	-2.387800	0.858600	-2.093100
H	-3.716600	1.231300	-3.180600

3gg-TS-Rot-*anti*

C	0.431800	-0.936000	-0.977000
C	-0.711500	0.039200	-1.108300
C	-0.038600	1.405600	-1.254700
C	1.359100	1.193300	-0.693000
N	1.701200	-0.207400	-0.851200
O	0.355400	-2.139700	-1.055800
O	2.014000	2.047400	-0.159300
H	0.014300	1.732300	-2.312600
Si	-1.951900	-0.020300	0.361300

C	-1.002000	-0.210700	1.944400
H	-0.484900	-1.187300	1.977400
H	-0.237000	0.567200	2.092400
H	-1.678900	-0.182700	2.815900
C	-3.121900	-1.454600	0.124900
C	-2.677200	-2.687400	-0.380900
C	-4.467700	-1.327000	0.512800
C	-3.560500	-3.765700	-0.504600
H	-1.631000	-2.828100	-0.684900
C	-5.350000	-2.405700	0.389800
H	-4.845900	-0.383600	0.915800
C	-4.897200	-3.626200	-0.120500
H	-3.202900	-4.715000	-0.899100
H	-6.389000	-2.294900	0.692600
H	-5.583400	-4.465600	-0.216700
C	-2.915700	1.583900	0.295200
C	-3.834300	1.794600	-0.749100
C	-2.734700	2.595200	1.251600
C	-4.553200	2.991000	-0.834300
H	-4.003800	1.023500	-1.505300
C	-3.455200	3.792900	1.166900
H	-2.031100	2.464700	2.076800
C	-4.363800	3.992100	0.124200
H	-5.262100	3.143300	-1.645900
H	-3.306500	4.569500	1.914900
H	-4.923800	4.923400	0.058000
H	-0.550300	2.235600	-0.720500
H	-1.328800	-0.223800	-2.011300
C	2.902600	-0.844500	-0.329200
C	3.995800	-0.197600	0.309300
C	2.865900	-2.260500	-0.285100
C	4.664600	-0.929200	1.312800
C	3.647700	-2.981400	0.612500
C	4.479300	-2.296400	1.501000
H	5.389700	-0.406500	1.941200
H	3.585700	-4.067500	0.635600
H	5.004200	-2.823800	2.292200
H	2.201300	-2.821800	-0.950700
C	4.740700	1.071800	-0.150400
C	4.613400	2.185600	0.899800
H	3.560300	2.434500	1.092900
H	5.056100	1.897800	1.856900
H	5.104900	3.104100	0.565500
C	6.247100	0.697300	-0.282100

H	6.382900	-0.162100	-0.946200
H	6.819400	1.533600	-0.695300
H	6.704000	0.449500	0.680100
C	4.312600	1.558300	-1.549200
H	3.917900	0.747000	-2.166400
H	3.561900	2.354900	-1.496500
H	5.157400	1.990600	-2.097700

3gg-TS-Rot-*syn*

C	-0.772500	-0.097400	-1.113400
C	0.639300	-0.635900	-1.156500
C	0.459700	-2.152700	-1.219500
C	-0.980700	-2.412200	-0.808400
N	-1.680900	-1.147900	-0.668000
O	-1.123200	0.971200	-1.545700
O	-1.453900	-3.512000	-0.667300
H	0.642300	-2.550000	-2.239600
Si	1.722700	-0.068300	0.319900
C	0.774800	-0.326300	1.897800
H	-0.176300	0.247200	1.883500
H	0.511200	-1.379300	2.077100
H	1.349100	0.026800	2.770600
C	2.170900	1.732300	0.117000
C	1.465000	2.623500	-0.705200
C	3.260200	2.217200	0.865700
C	1.839300	3.970500	-0.780400
H	0.606900	2.286200	-1.299900
C	3.633200	3.562700	0.790500
H	3.833900	1.548600	1.514200
C	2.922400	4.441300	-0.033800
H	1.282300	4.651200	-1.421900
H	4.476900	3.926700	1.373100
H	3.212500	5.488900	-0.092500
C	3.284300	-1.096900	0.255000
C	4.261900	-0.798800	-0.711000
C	3.503100	-2.172500	1.130300
C	5.429500	-1.563500	-0.801400
H	4.126600	0.039200	-1.400500
C	4.672100	-2.937500	1.040400
H	2.768800	-2.427600	1.897000
C	5.635300	-2.634600	0.074000
H	6.179800	-1.323800	-1.552600
H	4.831200	-3.768800	1.724700
H	6.544200	-3.229900	0.003500

H	1.150600	-2.728600	-0.565400
H	1.166800	-0.243500	-2.069700
C	-2.975200	-1.034200	-0.013000
C	-3.806300	0.116500	0.055600
C	-3.518800	-2.262300	0.446800
C	-5.196300	-0.117100	0.153700
C	-4.879300	-2.428100	0.677200
C	-5.747500	-1.361700	0.436300
H	-5.877400	0.730300	0.024500
H	-5.260400	-3.390500	1.013800
H	-6.823900	-1.493200	0.496300
H	-2.883100	-3.138000	0.612600
C	-3.412500	1.597700	0.213200
C	-3.520100	2.299600	-1.149700
H	-3.159400	3.330700	-1.099800
H	-2.923000	1.784400	-1.916200
H	-4.551500	2.319700	-1.512800
C	-4.413500	2.297100	1.180500
H	-4.461400	1.781500	2.144300
H	-4.109000	3.332300	1.365300
H	-5.429200	2.338900	0.772800
C	-2.026500	1.778000	0.867700
H	-1.294800	2.190200	0.159700
H	-2.055900	2.491900	1.699300
H	-1.622700	0.846900	1.283100

(*P*,*S*)-**3hh**

C	-0.914500	0.799300	-0.757800
C	0.352000	0.097600	-1.167600
C	-0.100500	-1.290100	-1.627700
C	-1.561000	-1.396200	-1.255600
N	-1.953800	-0.136800	-0.777000
O	-1.053700	1.955400	-0.423400
O	-2.286100	-2.351100	-1.353900
H	-0.011700	-1.429600	-2.709200
Si	1.584400	0.049300	0.306800
C	0.594000	-0.340700	1.844500
H	-0.103400	0.470600	2.071600
H	-0.003100	-1.248300	1.710200
H	1.240600	-0.481300	2.714900
C	2.549000	1.654900	0.414800
C	2.041900	2.881700	-0.037100
C	3.831900	1.643000	0.984300
C	2.791000	4.048900	0.075400

H	1.042200	2.925100	-0.461300
C	4.579000	2.809100	1.102700
H	4.258900	0.704500	1.332900
C	4.059000	4.015400	0.645100
H	2.380700	4.988800	-0.280900
H	5.569400	2.776500	1.546600
H	4.642700	4.926900	0.731200
C	2.796500	-1.345900	-0.039500
C	3.800800	-1.192100	-1.007100
C	2.711700	-2.578700	0.620500
C	4.681200	-2.225500	-1.303900
H	3.903600	-0.241700	-1.528500
C	3.591200	-3.617000	0.329800
H	1.947800	-2.733100	1.379300
C	4.577100	-3.441400	-0.634500
H	5.452400	-2.083300	-2.054500
H	3.508100	-4.562700	0.856700
H	5.265400	-4.249100	-0.862800
H	0.442400	-2.126200	-1.175300
H	0.833500	0.678800	-1.961300
C	-3.265600	0.133300	-0.299000
C	-4.123700	0.970700	-1.010000
C	-3.668000	-0.478100	0.898200
C	-5.405800	1.188400	-0.498900
C	-4.954300	-0.256600	1.385000
C	-5.811400	0.579200	0.677800
H	-6.087000	1.835900	-1.041800
H	-5.286100	-0.723300	2.303800
H	-6.813200	0.752000	1.057800
C	-3.680000	1.611900	-2.286400
H	-2.930300	2.384900	-2.093800
H	-3.223800	0.884600	-2.964500
H	-4.520600	2.074800	-2.805100
O	-2.734000	-1.248900	1.507800
C	-3.112500	-1.909200	2.697800
H	-2.238500	-2.472300	3.021600
H	-3.393000	-1.197000	3.482900
H	-3.944100	-2.601400	2.524500

3hh-TS-Rot-*anti*

C	0.675700	-0.937600	-1.196700
C	-0.456500	0.058000	-1.305300
C	0.243100	1.396000	-1.549300
C	1.629000	1.180900	-0.969400

N	1.958100	-0.252600	-1.056800
O	0.578400	-2.128800	-1.386800
O	2.301100	2.035600	-0.453700
H	0.302200	1.642500	-2.628700
Si	-1.569100	0.003800	0.260300
C	-0.487600	-0.378900	1.723700
H	0.029200	-1.352700	1.598100
H	0.292000	0.379900	1.896400
H	-1.087700	-0.453300	2.646800
C	-2.837200	-1.347500	0.035000
C	-2.497800	-2.559800	-0.589600
C	-4.138300	-1.186400	0.542000
C	-3.442100	-3.585000	-0.710900
H	-1.488600	-2.724800	-0.991200
C	-5.081800	-2.212500	0.420900
H	-4.433300	-0.257500	1.037600
C	-4.734600	-3.412500	-0.206800
H	-3.167400	-4.518600	-1.198500
H	-6.086100	-2.076400	0.816300
H	-5.468400	-4.210800	-0.301900
C	-2.420500	1.662500	0.400700
C	-3.419400	2.003400	-0.529400
C	-2.076200	2.590600	1.395700
C	-4.057900	3.245700	-0.464500
H	-3.713700	1.299900	-1.313100
C	-2.716200	3.834400	1.461100
H	-1.305100	2.358800	2.133800
C	-3.706300	4.162900	0.531600
H	-4.829800	3.499300	-1.188400
H	-2.440700	4.546100	2.237200
H	-4.203700	5.130100	0.582100
H	-0.250500	2.269900	-1.073700
H	-1.130800	-0.220800	-2.160800
C	3.075100	-0.688500	-0.234100
C	4.156600	0.242500	-0.057800
C	3.077000	-1.818600	0.613500
C	5.089100	0.139300	0.976200
C	4.080800	-1.956700	1.598800
C	5.039900	-0.976200	1.813700
H	5.854700	0.895800	1.111800
H	4.079900	-2.847500	2.224900
H	5.764500	-1.081300	2.620400
O	4.332000	1.098200	-1.121400
C	5.081400	2.320000	-0.893800

H	6.120100	2.088900	-0.647800
H	5.001500	2.810900	-1.871600
H	4.585500	2.914000	-0.118500
C	2.060600	-2.907500	0.583300
H	1.069000	-2.551500	0.913100
H	1.936100	-3.321600	-0.436400
H	2.324300	-3.755500	1.228500

3hh-TS-Rot-*syn*

C	0.921800	0.223000	1.441400
C	-0.383000	-0.523800	1.354300
C	0.034600	-1.986000	1.466400
C	1.508400	-2.010500	1.079600
N	2.087900	-0.659700	1.144100
O	1.079100	1.319400	1.917200
O	2.087800	-3.028800	0.805300
H	-0.101500	-2.380400	2.493700
Si	-1.368600	-0.093400	-0.232000
C	-0.221200	-0.071300	-1.688500
H	0.533800	0.732600	-1.554400
H	0.335000	-1.010700	-1.826600
H	-0.757000	0.142100	-2.627400
C	-2.164600	1.585800	-0.022400
C	-1.641900	2.575400	0.824800
C	-3.306000	1.883400	-0.789300
C	-2.249100	3.833900	0.908300
H	-0.749600	2.382300	1.434800
C	-3.911500	3.141600	-0.706700
H	-3.738900	1.134700	-1.458800
C	-3.383800	4.118300	0.143700
H	-1.837000	4.590300	1.573400
H	-4.794400	3.360100	-1.303700
H	-3.856100	5.096700	0.209900
C	-2.706000	-1.393500	-0.391200
C	-3.838100	-1.331000	0.440300
C	-2.595700	-2.452000	-1.306900
C	-4.836100	-2.307700	0.358500
H	-3.957000	-0.515000	1.158300
C	-3.594800	-3.429300	-1.389300
H	-1.731600	-2.528600	-1.970500
C	-4.714800	-3.358400	-0.556500
H	-5.708400	-2.249400	1.006500
H	-3.498600	-4.245100	-2.103200
H	-5.491500	-4.118600	-0.620000

H	-0.538900	-2.681100	0.815800
H	-1.058700	-0.217800	2.200800
C	3.014100	-0.304800	0.074700
C	3.103700	1.051300	-0.381100
C	4.163300	-1.098900	-0.191800
C	4.132300	1.531300	-1.194900
C	5.144700	-0.645200	-1.101700
C	5.147300	0.655900	-1.583000
H	4.148100	2.564100	-1.525300
H	5.950000	-1.322400	-1.383500
H	5.940700	1.000300	-2.245300
O	1.966000	1.790400	-0.146300
C	2.002700	3.224300	-0.354600
H	2.100900	3.446800	-1.419600
H	1.015000	3.518600	0.031000
H	2.801300	3.673400	0.242300
C	4.495000	-2.387600	0.481700
H	4.155200	-2.410700	1.528800
H	4.019100	-3.248400	-0.020300
H	5.575400	-2.582000	0.498500

(*P*,*S*)-**5a**

C	0.603100	0.420000	-0.187000
C	-0.636100	0.290000	0.657700
C	-0.280000	-0.765400	1.709400
C	1.034000	-1.364000	1.269100
N	1.493300	-0.591900	0.199100
O	0.834600	1.210400	-1.072700
O	1.623500	-2.300300	1.747600
H	-0.127900	-0.330900	2.702600
Si	-2.163800	-0.201300	-0.398000
C	-1.604000	-1.536100	-1.584000
H	-0.871300	-1.136100	-2.290400
H	-1.120100	-2.362300	-1.052800
H	-2.439800	-1.941600	-2.160600
C	-2.909900	1.290000	-1.259000
C	-2.124500	2.330000	-1.778800
C	-4.300800	1.365300	-1.432600
C	-2.710700	3.399300	-2.448000
H	-1.044600	2.296900	-1.660400
C	-4.886500	2.432600	-2.104100
H	-4.938700	0.580800	-1.030300
C	-4.090500	3.452400	-2.613700

H	-2.085800	4.195200	-2.842000
H	-5.964800	2.470500	-2.225800
H	-4.545500	4.288400	-3.136300
C	-3.425900	-0.889100	0.813800
C	-4.027200	-0.040700	1.755800
C	-3.759200	-2.248900	0.856000
C	-4.920000	-0.529200	2.701600
H	-3.798500	1.023900	1.743900
C	-4.655900	-2.744300	1.798500
H	-3.312300	-2.936700	0.141600
C	-5.235500	-1.884800	2.724100
H	-5.373100	0.145900	3.421100
H	-4.901200	-3.801800	1.811000
H	-5.933400	-2.268800	3.461200
H	-1.017200	-1.563300	1.841100
H	-0.853700	1.270900	1.093600
C	2.746700	-0.785700	-0.442200
C	3.829500	0.044000	-0.146400
C	2.840200	-1.771200	-1.435500
C	5.017200	-0.124400	-0.867500
C	4.038300	-1.941900	-2.125600
C	5.115700	-1.110700	-1.835200
H	5.866100	0.511100	-0.638900
H	4.131800	-2.705400	-2.887600
H	6.046500	-1.244100	-2.377100
C	3.728800	1.087700	0.896400
C	4.035000	2.416500	0.590900
C	3.339800	0.768800	2.202200
C	3.943400	3.405800	1.562700
H	4.320100	2.672300	-0.425300
C	3.251200	1.758800	3.173100
H	3.136100	-0.267100	2.458600
C	3.549100	3.080600	2.855800
H	4.174700	4.434800	1.306800
H	2.960000	1.494700	4.185100
H	3.477300	3.853000	3.614700
O	1.709000	-2.482400	-1.659300
C	1.746700	-3.465800	-2.673700
H	1.985700	-3.028100	-3.649900
H	2.473100	-4.253400	-2.443800
H	0.747900	-3.898200	-2.709400

5a-TS-Rot-*anti*

C	-0.580400	0.155800	-1.209900

C	0.778100	-0.495200	-1.312700
C	0.462100	-1.969500	-1.575800
C	-0.915000	-2.143700	-0.958900
N	-1.628900	-0.862900	-1.057600
O	-0.828100	1.319200	-1.399100
O	-1.294800	-3.140200	-0.402200
H	0.443300	-2.202600	-2.659300
Si	1.837500	-0.186400	0.260200
C	0.730700	-0.279300	1.751600
H	-0.020500	0.536600	1.743700
H	0.179600	-1.229800	1.825000
H	1.316500	-0.162000	2.679400
C	2.609000	1.509300	0.146800
C	1.867700	2.608900	-0.318500
C	3.928100	1.717900	0.586000
C	2.437100	3.886600	-0.351200
H	0.830100	2.489700	-0.664000
C	4.496200	2.996100	0.553500
H	4.530500	0.885700	0.959700
C	3.751200	4.081800	0.083300
H	1.852200	4.728400	-0.717700
H	5.518300	3.145000	0.894900
H	4.193300	5.076000	0.056500
C	3.171700	-1.498700	0.272300
C	4.192700	-1.451800	-0.694300
C	3.170800	-2.546400	1.206100
C	5.189600	-2.432000	-0.724400
H	4.223900	-0.645900	-1.432400
C	4.169600	-3.527600	1.176400
H	2.392800	-2.613900	1.969800
C	5.178700	-3.471500	0.211700
H	5.975500	-2.385900	-1.475800
H	4.158500	-4.335100	1.906100
H	5.955100	-4.234300	0.188200
H	1.186000	-2.684800	-1.131400
H	1.359500	-0.033700	-2.157200
C	-2.825600	-0.696000	-0.255700
C	-3.569600	-1.873400	0.116300
C	-3.192100	0.492400	0.403700
C	-4.437400	-1.909700	1.207100
C	-4.141100	0.463300	1.453500
C	-4.709400	-0.722800	1.893300
H	-4.920100	-2.836600	1.501200
H	-4.407200	1.403200	1.939300

H	-5.384800	-0.731000	2.747500
C	-2.689800	1.863600	0.132900
C	-1.716700	2.429300	0.974100
C	-3.285800	2.638200	-0.874700
C	-1.338400	3.762400	0.799400
H	-1.246200	1.820200	1.747500
C	-2.900200	3.971200	-1.041100
H	-4.032500	2.194200	-1.530200
C	-1.931900	4.535700	-0.204200
H	-0.578000	4.198700	1.444500
H	-3.354700	4.570600	-1.827900
H	-1.637500	5.573900	-0.336600
O	-3.545200	-2.868600	-0.833000
C	-3.860300	-4.220900	-0.409700
H	-4.902400	-4.285000	-0.090100
H	-3.688700	-4.783800	-1.335800
H	-3.157400	-4.535200	0.369100

5a-TS-Rot-*syn*

C	0.038800	-0.857100	-1.290900
C	-0.906600	0.316700	-1.301100
C	0.025400	1.520300	-1.394700
C	1.338800	1.025500	-0.808700
N	1.419500	-0.449300	-0.897900
O	-0.190600	-1.943900	-1.758200
O	2.167400	1.749400	-0.328300
H	0.166500	1.858400	-2.441000
Si	-2.060500	0.285600	0.231400
C	-1.095200	-0.320900	1.695900
H	-0.722600	-1.349400	1.511300
H	-0.216800	0.300200	1.930300
H	-1.723800	-0.364800	2.600800
C	-3.478600	-0.879800	-0.122400
C	-3.298600	-2.044400	-0.887000
C	-4.744300	-0.621600	0.432400
C	-4.363700	-2.926800	-1.099300
H	-2.322700	-2.283300	-1.329600
C	-5.808400	-1.505200	0.221300
H	-4.917000	0.274100	1.035500
C	-5.619200	-2.658300	-0.546300
H	-4.213200	-3.823000	-1.697900
H	-6.783700	-1.294500	0.654900
H	-6.447200	-3.344900	-0.712600
C	-2.712200	2.024200	0.459500

C	-3.667400	2.529000	-0.441000
C	-2.251000	2.853900	1.493600
C	-4.149000	3.835300	-0.309200
H	-4.050100	1.905800	-1.253700
C	-2.733600	4.161700	1.625900
H	-1.510000	2.493700	2.210700
C	-3.681800	4.653300	0.724900
H	-4.888300	4.216000	-1.011100
H	-2.368100	4.795900	2.431300
H	-4.056500	5.670300	0.827200
H	-0.325100	2.419700	-0.844000
H	-1.595300	0.253000	-2.187900
C	2.105300	-1.088000	0.215300
C	1.812200	-2.444200	0.580000
C	3.377300	-0.626100	0.644800
C	2.580400	-3.182300	1.484100
C	4.101200	-1.330200	1.632700
C	3.717500	-2.598900	2.044300
H	2.304500	-4.197600	1.750800
H	5.018600	-0.890500	2.026300
H	4.307900	-3.146400	2.777700
C	4.161500	0.483200	0.039600
C	4.547400	0.379900	-1.308400
C	4.640300	1.546800	0.824500
C	5.398400	1.338400	-1.863400
H	4.174400	-0.443300	-1.916600
C	5.486100	2.502700	0.258000
H	4.329100	1.636600	1.862700
C	5.870100	2.398900	-1.083400
H	5.694400	1.257900	-2.907500
H	5.845300	3.333100	0.862600
H	6.531000	3.143900	-1.519300
O	0.584500	-2.884700	0.144900
C	0.262700	-4.292700	0.265100
H	0.112100	-4.554600	1.314900
H	-0.677800	-4.339400	-0.303700
H	1.032400	-4.905000	-0.212100
